# Photoredox Annulation of Polycyclic Aromatic Hydrocarbons

**DOI:** 10.1021/jacsau.3c00438

**Published:** 2023-11-14

**Authors:** Davide Zanetti, Oliwia Matuszewska, Giuliana Giorgianni, Cristofer Pezzetta, Nicola Demitri, Davide Bonifazi

**Affiliations:** † Institute of Organic Chemistry, Faculty of Chemistry, University of Vienna, Währinger Straße 38, 1090 Vienna, Austria; ‡ School of Chemistry, Cardiff University, Main Building, Park Place, Cardiff CF10 3AT, U.K.; § Elettra—Sincrotrone Trieste, S.S. 14 Km 163.5 in Area Science Park, 34149 Basovizza, Trieste, Italy

**Keywords:** radical, aryl−aryl coupling, polycyclic aromatic hydrocarbons, nanographenes, photoredox, PXX

## Abstract

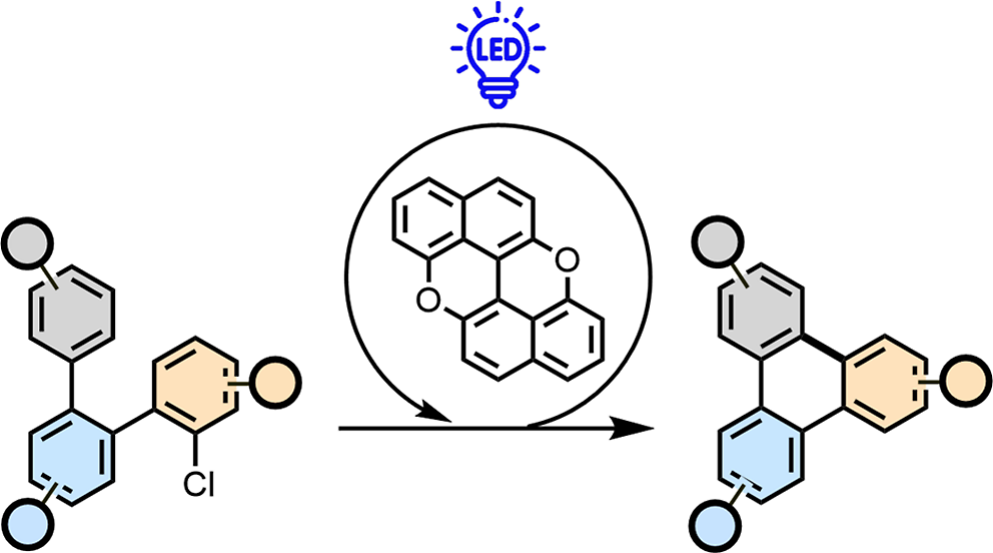

The rise of interest in using polycyclic aromatic hydrocarbons (PAHs) and molecular graphenoids in optoelectronics has recently stimulated the growth of modern synthetic methodologies giving access to intramolecular aryl–aryl couplings. Here, we show that a radical-based annulation protocol allows expansion of the planarization approaches to prepare functionalized molecular graphenoids. The enabler of this reaction is *peri*-xanthenoxanthene, the photocatalyst which undergoes photoinduced single electron transfer with an *ortho*-oligoarylenyl precursor bearing electron-withdrawing and nucleofuge groups. Dissociative electron transfer enables the formation of persistent aryl radical intermediates, the latter undergoing intramolecular C–C bond formation, allowing the planarization reaction to occur. The reaction conditions are mild and compatible with various electron-withdrawing and -donating substituents on the aryl rings as well as heterocycles and PAHs. The method could be applied to induce double annulation reactions, allowing the synthesis of π-extended scaffolds with different edge peripheries.

## Introduction

**Scheme 1:**
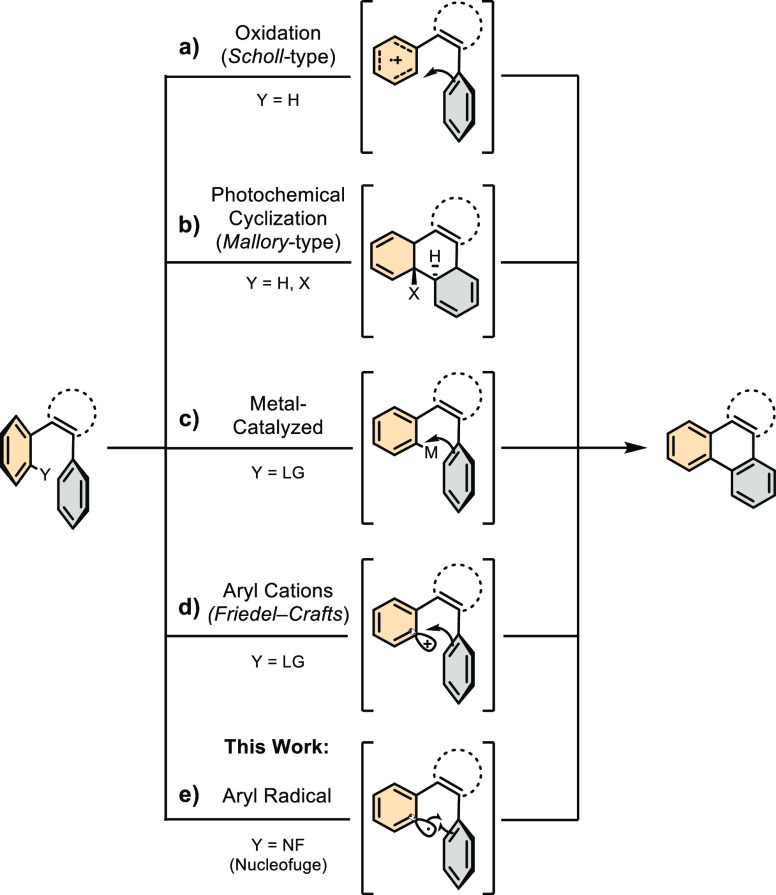
Annulation Approaches Enabling the Formation of PAHs and Molecular Graphenoid Fragments

In recent years, molecular graphenoids have emerged as the organic alternative to inorganic semiconductors.^1−3^ Solution-phase organic synthesis has proven to be very effective for preparing polycyclic aromatic hydrocarbons (PAHs) and molecular graphenoids with tailored properties through the control of edge configurations, size, peripheral moieties, and heteroatom doping.^4−6^ The typical synthetic approach relies on Scholl-type transformations (Scheme 1a),^7−13^ in which oligophenylene precursors are reacted in the presence of a Lewis acid (e.g., AlCl_3_ or FeCl_3_)^14−19^ and an oxidant (e.g., O_2_, CuCl_2_, or DDQ)^16,20−24^ to afford π-type radical carbocation intermediate undergoing intramolecular C–C bond formation and consequent fusion. Other approaches consist of using light to generate reactive intermediates (Scheme 1b). Seminal examples include the Mallory^25−30^ and cyclodehydrochlorination (CDHC)^31−35^ reactions. In these transformations, a sequence of photoinduced (UV) electrocyclization and oxidation (Mallory) or elimination (CDHC) steps occurs to yield annulated products. Recently, a solid-state version of the Mallory and CDHC reactions has also been developed using a photomechanochemical protocol.^36^ Intramolecular Pd-catalyzed direct C–H arylation^37−45^ (Scheme 1c) and Friedel–Crafts-type^46−53^ (Scheme 1d) reactions have also been exploited as annulation protocols. In all approaches, either very reactive species (e.g., silylium ions, strong oxidants, or Lewis acids), transition metals, or deep-UV irradiation (λ = between 254 and 300 nm with 450 W power lamps or equivalent) are required to trigger the formation of the reactive intermediates leading to the C–C bond formation. Although these methods have been revealed to be powerful for preparing a variety of unsubstituted PAHs and nanographenes, they are often incompatible with functional groups or heteroatom-doped frameworks. For instance, typical Scholl-type conditions are unsuitable when the oligoarylenyl precursors bear electron-withdrawing (EW) groups. The same applies to silylium-triggered Friedel–Crafts transformations and photoinduced electrocyclizations on substrates bearing either heteroatoms or peripheral functional groups. While looking for alternative protocols with mild experimental conditions, conceptually, we noticed that the annulation reaction could also be achieved through a radical route (Scheme 1e), exploiting a reactive sp^2^-type aryl radical intermediate that, through intramolecular C–C bond formation with a suitable radical acceptor moiety, undergoes planarization. At the synthetic planning level, this consideration guided us to contemplate the photoredox approach^54^ to mildly generate the given aryl radical intermediate. Our group first reported the use of *peri*-xanthenoxanthene (**PXX**) as a strong and low-cost photoreducer (E^1/2^(PXX^•+^/PXX*) = −2.00 V vs SCE; E^1/2^(PXX*/PXX^•–^) = +0.61 V vs SCE upon irradiation at 450 nm) to generate reactive radical species from electron-poor aryl and alkyl halides.^55−57^ Considering that **PXX** could enable a variety of different transformations (e.g., dehalogenation;^56^ C–C, C–S, and C–N bond formation;^57^ iododifluoromethylation of alkenes;^58^ polymerization of olefins;^59^ and fluorosulfonylation reactions^60−63^), we hypothesized that **PXX** could also be used as a photocatalyst to activate the reduction of a suitable electron-deficient aryl moiety into a radical intermediate that would undergo intramolecular C–C bond formation with a neighboring aryl substituent (Scheme 1e).

Based on this hypothesis, herein we report, to the best of our knowledge, the first radical-based annulation protocol to prepare substituted PAH structures. To show the versatility, functional group tolerance, and potentialities of this approach, the syntheses of substituted triphenylenes (including variants bearing five- and six-membered heterocycles), tetrabenzo-anthracenes, and dibenzopyrenes were selected as archetypal frameworks.

## Results and Discussion

Our studies commenced with a screening of the reaction conditions enabling the radical annulation reaction to take place (Table 1). *ortho*-Terphenyl educt **1**, bearing nitrile and chloride moieties as the electron-withdrawing (EW) and nucleofuge (NF) groups, respectively, served as a model substrate for the reaction optimization and mechanistic studies. The reaction conditions developed by our group to dehalogenate *p*-bromobenzaldehyde (entry 1, **PXX** 2 mol %, 1.4 equiv of DIPEA in CH_3_CN at rt overnight) were initially used.^56^ Precursor **1** could thus be converted into triphenylene **2** in 52% yield. Higher conversion was obtained with DMF but with similar yield (entry 2). Performing the reaction in DMSO led to high conversion and a high yield (75%, entry 5). Apolar solvents such as C_6_H_6_ and CHCl_3_ either gave lower conversions or no product. Lessening of the annulation yield was observed when different amounts of **PXX** (entries 6–10), DIPEA (entries 11–12), and longer reaction times (entries 13–14) were used. Under air, the reaction yielded inferior quantities of the triphenylene derivative (entry 15).

**Table 1:**
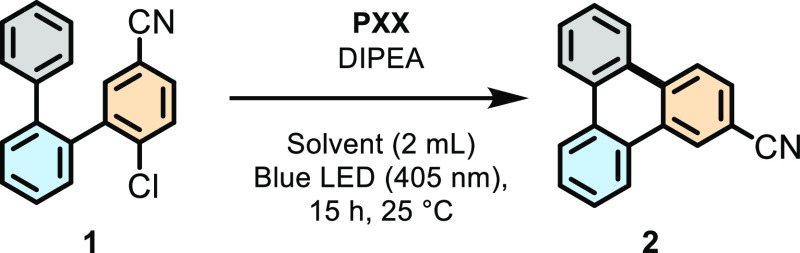
Optimization of the Reaction Conditions for the Annulation of *ortho*-Terphenyl **1** into Triphenylene **2** as a Model Substrate^a^

entry^b^	solvent^c^	PXX (mol %)	base (equiv)	conv (%)^d^	yield (%)^d^
1	CH_3_CN	2	1.4	89	52
2	DMF	2	1.4	>95	49
3	CHCl_3_	2	1.4	18	0
4	C_6_H_6_	2	1.4	26	6
**5**	**DMSO**	**2**	**1.4**	**>95**	**75/72**^e^
6	DMSO	0.1	1.4	55	39
7	DMSO	0.5	1.4	66	48
8	DMSO	1	1.4	>95	51
9	DMSO	5	1.4	>95	57
10	DMSO	10	1.4	>95	47
11	DMSO	2	2	>95	59
12	DMSO	2	1	>95	53
13^f^	DMSO	2	1.4	>95	51
14^g^	DMSO	2	1.4	>95	40
15	DMSO^h^	2	1.4	80	57
16^i^	DMSO	2	1.4	75	47
17	DMSO	–	1.4	70	0
18	DMSO	2	–	68	0
19^j^	DMSO	2	1.4	60	0
20^k^	DMSO	–	1.4	<5	0
21^l^	DMSO	2	1.4	0	0

a Conversions and yields were measured by ^1^H NMR using 1,3,5-trimethoxybenzene as an internal standard.

b Reaction was performed on 0.25 mmol of the starting material.

c Degassed solvent.

d Determined by ^1^H NMR analysis using 1,3,5-trimethoxybenzene as an internal standard.

e Isolated yield.

f 48 h.

g 72 h.

h Air-equilibrated.

i Cs_2_CO_3_ instead of DIPEA.

j Reaction was performed in the dark.

k Irradiation at 285 nm.

l Reaction was performed with reference molecule **1a** (Supporting Information) lacking the nitrile group.

Notably, when DIPEA is replaced by Cs_2_CO_3_ (entry 16), triphenylene is also obtained in a good yield (47%). This suggests that DIPEA intervenes in the mechanism solely as a base. As shown by the control experiments (entries 17–19), no product formation could be observed in the absence of either **PXX**, DIPEA, or light. At last, reactions performed at 285 nm in the absence of **PXX** (entry 20) and with reference terphenyl precursor **1a** (entry 21) gave no product, suggesting that no photoinduced electrocyclizations are possible under our conditions and that the presence of the EW nitrile group is instrumental for the annulation to occur.

**Scheme 2:**
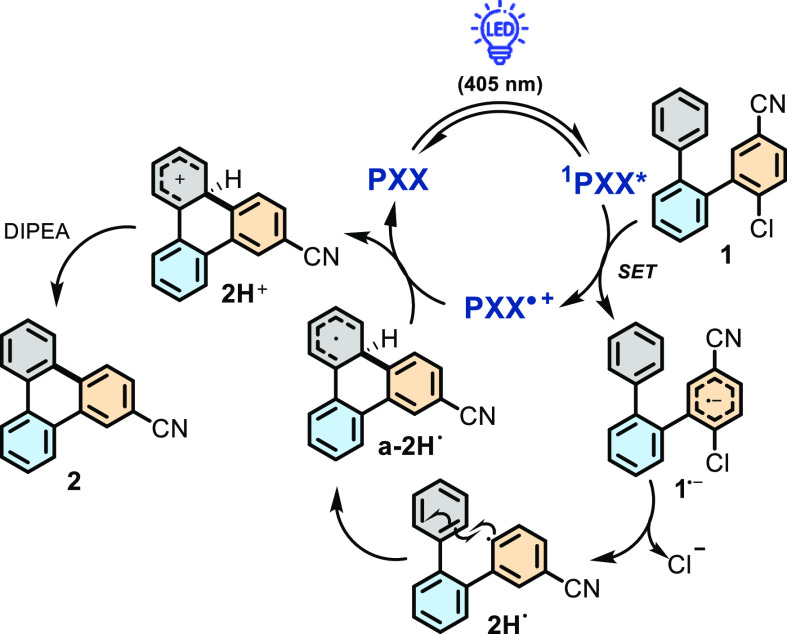
Proposed Photooxidative Mechanism for the Annulation Reaction through C–C Bond Formation Triggered by Light

The mechanism of the reaction was subsequently assessed (Scheme 2). Building on the estimated reductive and oxidative properties (see above) of ^**1**^PXX*, we conjectured that ^**1**^PXX* can reduce the electron-deficient 1-chloro-4-cyano-aryl moiety.^56^ Stern–Volmer investigations (Figure S220, Supporting Information) reflect these considerations, showing that ^**1**^PXX* is effectively quenched by precursor **1** (2.2 × 10^10^ M^–1^ s^–1^) in DMSO as opposed to substrate **1a** (no quenching was observed, Figure S221). Considering that the product yields increase with the polarity of the medium, one can realistically hypothesize that a photoinduced charge transfer occurs from ^**1**^PXX* to the terphenyl substrate, forming **PXX**^**•+**^ and radical anion **1**^**•–**^ as intermediates (Scheme 2).

The aryl radical anion likely undergoes fragmentation by dissociative electron transfer, giving a Cl^–^ anion and persistent aryl radical intermediate **2H**^**•**^ (Scheme 2). The latter reacts intramolecularly with the radical acceptor phenyl ring (gray aromatic ring) to form the new C–C bond and give intermediate **a-2H**^**•**^. Oxidation of the resultant radical conjugate by **PXX**^**•+**^ and subsequent deprotonation by DIPEA of cationic intermediate **2H**^**+**^ gives the final product, triphenylene **2** (a radical chain propagation mechanism propelled by a direct reduction of **1** by the radical species a-2H^•^ cannot be completely excluded). The existence of the persistent radical intermediate **2H**^**•**^ was confirmed by a radical-trapping experiment. When the annulation of **1** was attempted in the presence of a large excess of TEMPO, the relevant adduct (**2HTEMPO**) was unambiguously observed by HRMS-MALDI-TOF analysis of the reaction mixture (Figure S230). Unfortunately, the lack of any phosphorescence emission and the photodegradation of **PXX** under laser excitation at 355 nm prevented us from gathering any mechanistic information on the role of the dark ^**3**^PXX* excited state.^56^

Having in hand the optimized reaction conditions and the fundamental mechanistic insights, attention was then focused on examining the generality of our photoredox annulation protocol to prepare differently substituted molecular graphenoids from the given *ortho*-oligoarylenyl precursors. The variation of the substituents on the aryl radical acceptor (gray ring), donor (light mocha ring), and linker (sky blue ring) moieties (Table 2), the π-extension of the radical acceptor aryl ring (Table 3), and the number of annulations (Table 4) were studied. As none of the oligoarylene precursors (**3**, **5**, **7**, **9**, **10**, **12**, and **13**) were commercially available, they were all prepared through sequential Suzuki coupling reactions except precursor **3r**, for which a synthetic strategy recently reported by our group was employed^64^ (see Supporting Information for the synthetic routes and details). As outlined in Table 2, a variety of triphenylenes could be prepared in good to excellent yields. Moving the nitrile group from the 4-(**1**) to 6-(**3a**) and 5-position (**3b**) on the radical donor aryl moiety of the terphenyl precursors, the relevant nitrile-bearing triphenylenes could be obtained in excellent yields (>70%), suggesting that the dissociative electron transfer leading to the aryl radical intermediate occurs independently on the substitutional pattern between the EW and NF groups. Lower annulation yields (33%) were registered when two nitrile groups were installed on the radical donor ring (**3c**). When the nitrile group was switched to a methyl ester moiety, the corresponding triphenylenes were also obtained in excellent yield (>70%). As one can expect, lower yields (36–38%) of the corresponding annulated products were obtained when either a nitrile (**3f**) or an ester (**3g**) group was placed on the radical acceptor ring. Interestingly, any precursors bearing the nitrile group on to the linker ring (**3h**, **i**) gave no conversion and only starting materials were recovered (see Supporting Information for a discussion). On the other hand, precursor **3j**, featuring an ester group in the linker ring, could also be annulated in good yield (66%).

**Table 2:**
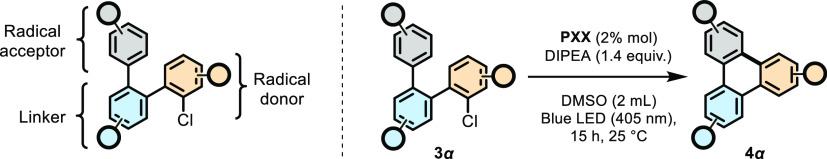

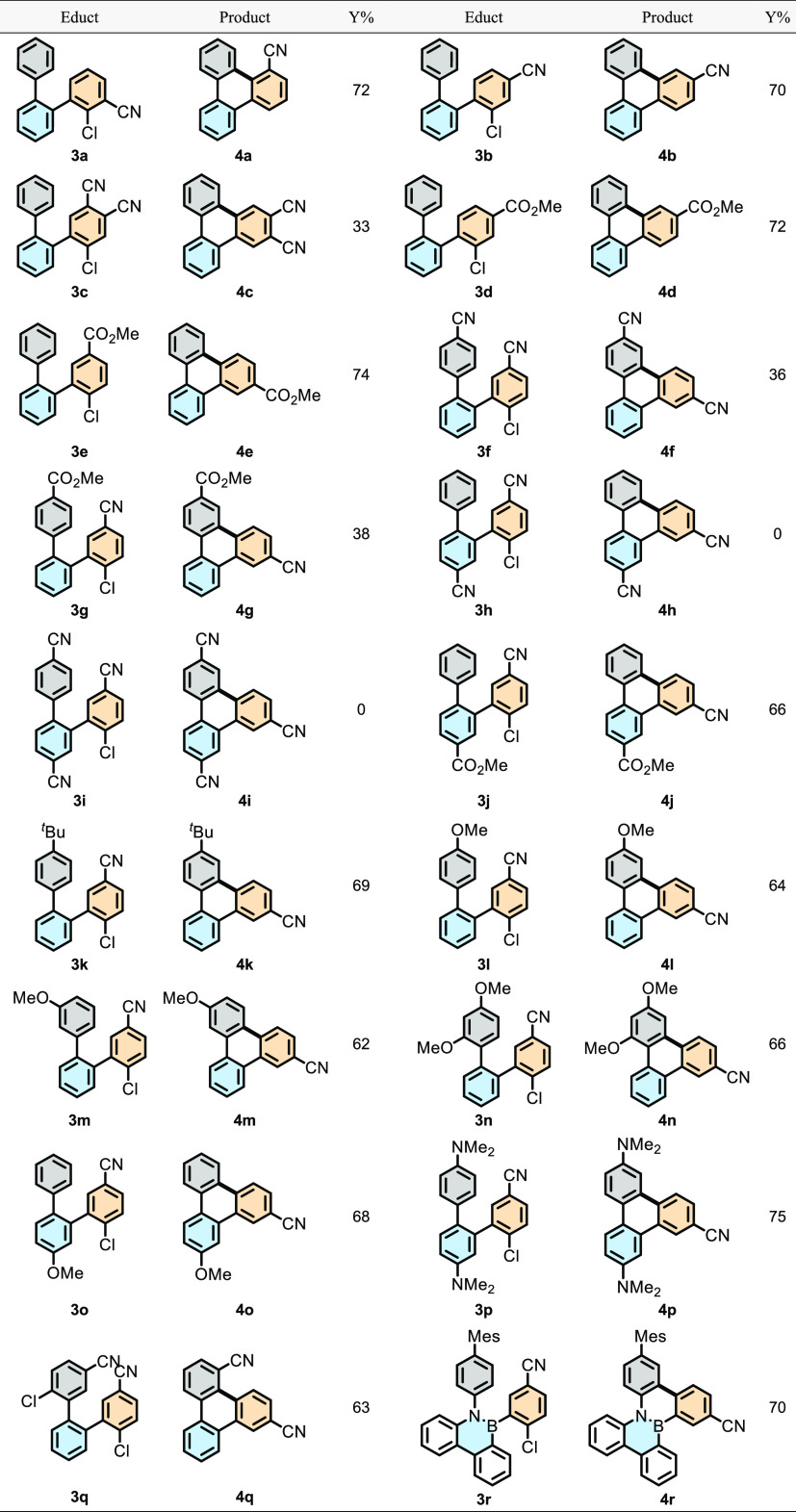
Photoredox Planarization Reaction on Various Substituted *ortho*-Terphenyl Educts Was Used to Prepare Substituted Triphenylenes^a^

a Reactions were performed on a 0.25 mmol scale. Yields reported are isolated.

**Table 3:**
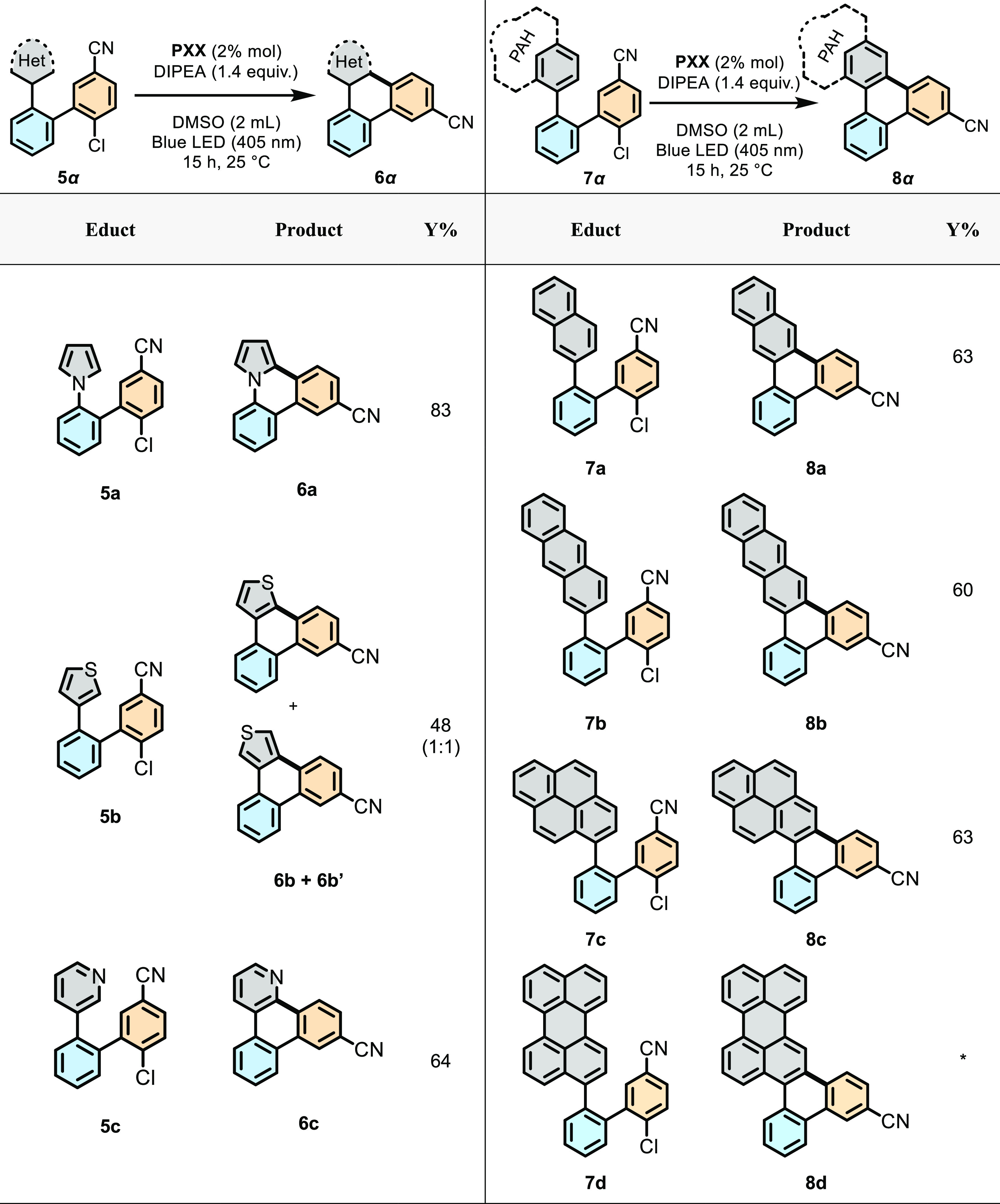
Variation of the π-Structure: Molecular Graphenoids from the Annulation of Heterocycles (Left) and PAH Moieties (Right)^a^

a Reactions were performed on a 0.25 mmol scale. Yields reported are isolated.

* Traces.

**Table 4:**
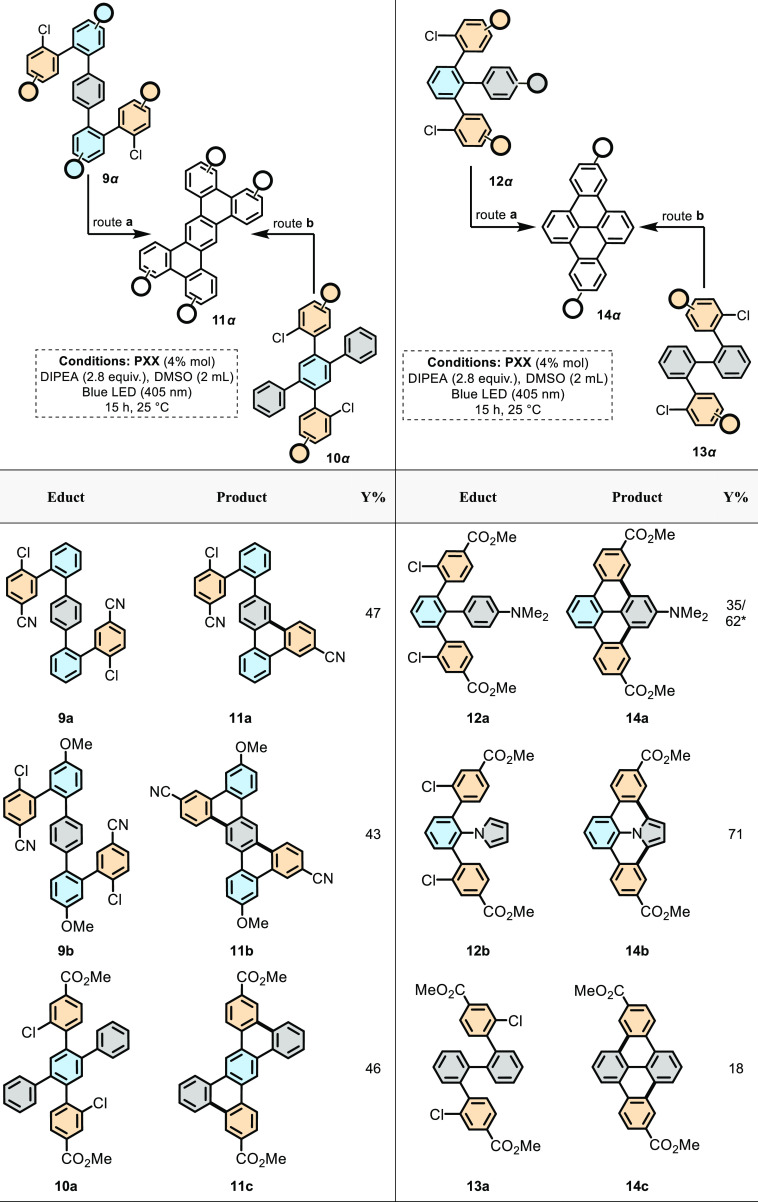
Extension of the p-Structure: Molecular Tetrabenzoanthracene and Dibenzopyrene Graphenoids Synthesized through Double Photoredox Annulation Reactions^a^

a Reaction was performed on a 0.25 mmol scale. Yields reported are isolated.

* 45 h reaction time.

As expected, the installation of electron-donating groups (EDGs) on either the radical acceptor (**3k**–**n**), linking (**3o**), or both (**3p**) rings did not affect the reaction outcome, and the relevant triphenylenes could be formed in good to excellent yields (62–75%). When terphenyl derivative **3q** bearing two 1-chloro-4-cyano aryl moieties was reacted, dicyano triphenylene **4q** was obtained with a good yield (63%). In this case, we hypothesized that radical addition followed either by an elimination reaction or by a photoinduced dehalogenation occurs. At last, when the linking aryl moiety was replaced with a BN-isostere of phenanthrene (**3r**), the corresponding annulated product could be prepared in 70% yield. Notably, the annulation of this BN-containing compound could not be achieved with either Scholl or CDHC-type approaches (see also literature reports about other BN-containing PAHs).^65−68^ This suggests that our protocol can also be applied to heteroatom-doped substrates such as those containing boron–nitrogen bonds. When the radical acceptor ring was substituted by a heterocycle (Table 3), i.e., pyrrole (**5a**), thiophene (**5b**), and pyridine (**5c**), the corresponding annulated products could be obtained in good to excellent yields (83, 48, and 64% for **6a**, **6b** + **6b′**, and **6c**, respectively). Notably, two isomers were obtained in a 1:1 ratio with the thienophenyl derivative (**6b-b′**). When swapping the phenyl ring to naphthyl, anthracenyl, pyrenyl, and perylenyl moieties, the related annulation could be achieved with good yields (63, 60, and 63% for **8a**, **8b**, and **8c**, respectively), only with the exception of **8d**, which was detected in traces in HRMS analysis when the catalyst charge was raised to 20% mol/mol (Table 3). Considering the higher potential of the first reductive event for perylene (*E*^1/2^(per/per^•–^) = −1.63 V vs SCE in THF)^69,70^ when compared to that of the 1-chloro-2-cyano-aryl moiety (*E*^1/2^(CNArCl/CNArCl^•–^) = −1.88 V vs SCE in DMF),^71^ one can envisage that, in the case of **7d**, the reductive photoinduced SET predominantly takes place on the perylene moiety. Under these circumstances, no effective photoinduced SET can take place with the 1-chloro-4-cyano-aryl moiety to give a persistent aryl radical, and the annulation cannot occur.

At last, we have investigated the possibility of triggering two C–C bond formations to allow the formation of graphene fragments such as the tetrabenzo-anthracene (Table 4, left) and dibenzopyrene (Table 4, right) scaffolds depicting cove and armchair peripheries, respectively. To prepare tetrabenzoanthracenes, one can easily notice that the molecule can be built from quinquephenyl precursors, bearing two radical donor moieties at the *para*-position of the radical acceptor aryl ring. When two 1-chloro-4-cyano-aryl substituents were used, the **PXX**-photocatalyzed reaction afforded only monoannulated derivative **11a**, and the bis annulation could not be achieved (even at longer times or at higher concentration of **PXX**). As the double annulation reaction likely proceeds stepwise, we conjectured that the cyano-triphenylene intermediate (*E*^1/2^red = −1.83 V vs SCE in CH_2_Cl_2,_ Figure S223, Supporting Information), formed after the first cyclization, undergoes faster photoreduction than the lingering 1-chloro-4-cyano-aryl group, preventing the second annulation from occurring. To deactivate the electron acceptor capabilities of the intermediate cyano-triphenylene moiety, a decision was taken to install an electron-donating methoxy group on the linking aryl rings.

**Figure 1:**
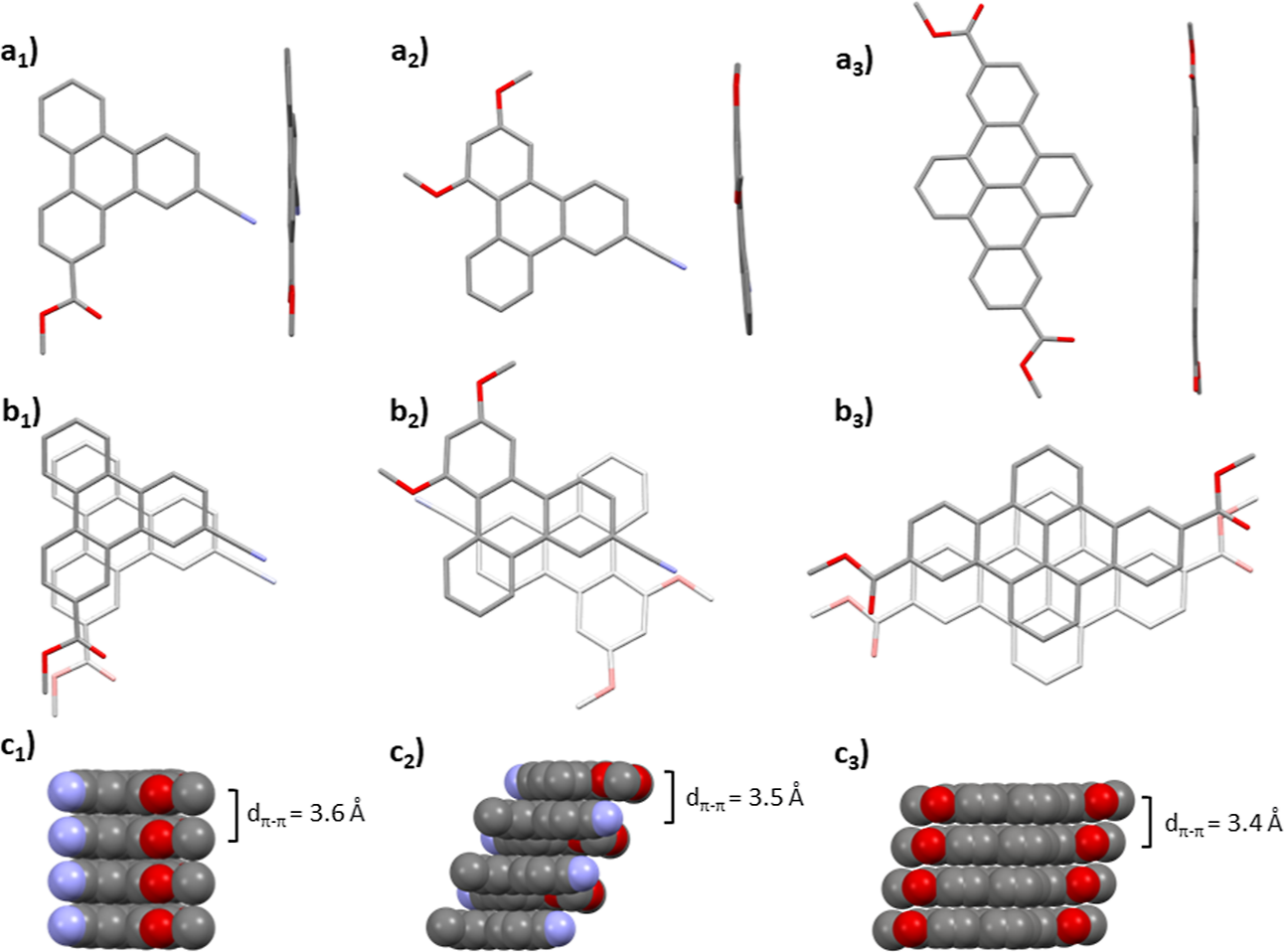
Selected X-ray structures of the PAHs prepared in this work. First row: top- and side-view of crystal structures **4j** (a_1_), **4n** (a_2_), and **14c** (a_3_). Second row: offset (b_1_, **4j** and b_3_, **14c**) and *anti*-offset (b_2_, **4n**) π–π stacking arrangements. Third row: solid-state columnar π–π stacks (c_1_, c_2_, and c_3_). The PXX molecule in the cocrystal of product **14c** was removed for clarity (see complete structure in Figure S226).

Thus, when precursor quinquephenyl **9b** was irradiated in the presence of **PXX**, doubly annulated tetrabenzoanthracene derivative **11b** could be obtained in 43% yield (66% per C–C bond). Tetrabenzoanthracene scaffolds could also be obtained if the radical acceptor and donor aryl moieties were all installed around a tetrasubstituted benzene ring. Thus, precursor **10a**, bearing EW ester groups, was prepared. When reacted with **PXX** under 405 nm light, quaterphenylene **10a** underwent double cyclization to afford tetrabenzoanthracene **11c**. Notably, the use of an ester moiety as an EW group allowed us to perform the double cyclization without the need to introduce an additional electron-donating group. We believe that the moderate EW characteristics of the ester compared to those of the nitrile group allowed the photoreduction to mainly occur with the 1-chloro-3-methyloxycarbonyl-aryl group (i.e., with the ester-bearing triphenylene intermediate being a weaker electron acceptor). Then, we moved our gaze toward the construction of the dibenzopyrene scaffolds. Considering the good yields obtained for the tetrabenzoanthracene derivative from the corresponding ester-based precursor, the syntheses were conducted only with methyl ester as the EW group. In this instance, we first planned to obtain the target molecule starting from a quaterphenylene precursor in which a trisubstituted linking unit bears two lateral radical donor and one central radical acceptor aryl moieties. Thus, we first prepared quaterphenylene **12a** that, bearing a *N*,*N*-dimethylanilino moiety as a radical acceptor ring, could be transformed into doubly annulated product **14a** in 35% in 15 h. To our gratification, when reacted for 45 h, molecule **14a** could be obtained in 62% yield (79% yield per C–C bond). When switching the aryl acceptor ring to a pyrrol-1-yl moiety, as in quaterphenylene **12b**, the cyclization reaction afforded the final product **14b** in 71% yield (85% yield per C–C bond). Finally, the same dibenzopyrene structure could be prepared starting from quaterphenylene precursor **13a**, in which the two radical acceptor moieties are linked in the *ortho* position. In this case, the photocatalyzed annulation gave relevant dibenzopyrene derivative **14a** in 18% yield under classical reaction conditions (unoptimized for this product). To further confirm the structure of the annulated products, suitable crystals for X-ray diffraction (Figure 1) were prepared by slow evaporation from CHCl_3_ (**4j**) and vapor diffusion of MeOH in CHCl_3_ (**4n** and **14c**, the latter obtained only when cocrystallized with **PXX**) of exemplary products. As expected, all X-ray structures confirmed the formation of the annulated products in which either one (**4j**, **4n**) or two (**14c**) C–C bonds were formed. All the products showed a nearly flat shape with offset (**4j** and **14c**) and *anti*-offset (**4n**) π–π stacking arrangements in the third dimension. As expected, all the molecules form columnar architectures in the solid state governed by π–π stacking interactions. Specifically, molecule **4j** undergoes face-to-face arrangements with negligible offset, which is a rather unprecedented packing characteristic for these types of PAHs.

## Conclusions

In summary, we have developed a general, mild, and metal-free radical-based annulation method using visible light to synthesize peripherally substituted PAHs from *ortho*-oligoarylenyl precursors. The reaction mechanism involves a photoinduced single electron transfer from the excited **PXX** photocatalyst to the oligoarylenyl substrate, followed by a dissociative electron transfer generating a persistent radical undergoing an annulation reaction. The mild reaction conditions are tolerant to a large variety of functional groups on the aryl rings, as well as polycyclic aromatic hydrocarbons, heterocycles, and even boron–nitrogen-containing substituents. In this first study, we enabled the formation of triphenylene and other substituted or heteroatom-containing PAHs possessing different edge peripheries, such as tetrabenzoanthracenes and dibenzopyrene, as a proof of concept of the π-conjugated framework. The work demonstrates, for the first time, the versatility and power of the photoredox approach for the construction of PAHs.

## Methods

### Synthesis

Chemicals were purchased from BLDpharm, Sigma-Aldrich, Acros Organics, TCI, Apollo Scientific, Fluorochem, and ABCR and used without any further purification. Solvents were purchased from Sigma-Aldrich, while deuterated solvents were purchased from Eurisotop. Anhydrous conditions were achieved by drying Schlenk tubes or round-bottom flasks in an oven at 120 °C for at least 4 h, followed by evacuation under vacuum and purging with argon. An inert atmosphere was maintained using argon-filled balloons equipped with a syringe and needle that were used to pierce the silicon stoppers used to close the flasks’ necks. All batch photocatalytic studies were performed in a custom-built photoreactor setup featuring a single high-power blue LED (1260 mW, 4.4 mm × 4.4 mm, 405 nm, LED Engin) as the light source used for illuminating the reaction vessel from the bottom. The LED was passively cooled by the attached aluminum heatsink, while the reaction temperature was held constant by circulating a cooling fluid through the custom-made aluminum support connected to a chiller as designed by the Melchiorre’s research group.^72^ The details of all synthetic procedures to prepare both precursors and annulated products are described in full detail in the Supporting Information.

### Instrumentation

Thin layer chromatography (TLC) was conducted on precoated aluminum sheets with 0.20 mm *Machevery-Nagel* Alugram SIL G/UV254 with fluorescent indicator UV254. Column chromatography was carried out using *Merck Gerduran* silica gel 60 (particle size of 63–200 mm). Melting points (mp) were measured on a Gallenkamp apparatus in open capillary tubes and have not been corrected. Nuclear magnetic resonance (NMR): (i) ^1^H and ^13^C spectra were obtained using a Bruker Fourier 300 MHz spectrometer equipped with a dual (^13^C, ^1^H) probe at Cardiff University. (ii) ^1^H, ^11^B, and ^13^C spectra were obtained using a Bruker AVANCE III HD 400 MHz NMR spectrometer equipped with a broadband multinuclear (BBFO) SmartProbe, a Bruker AVANCE III HD 500 MHz spectrometer equipped with a broadband multinuclear (BBO) Prodigy CryoProbe, or a Bruker AV III HDX 700 NMR spectrometer (Bruker BioSpin, Rheinstetten, Germany) at Vienna University. Chemical shifts were reported in parts per million according to tetramethylsilane using the solvent residual signal as an internal reference (CDCl_3_: δ_H_ = 7.26 ppm, δ_C_ = 77.16 ppm). Coupling constants (*J*) are given in Hz. Resonance multiplicity was described as *s* (singlet), *d* (doublet), *t* (triplet), *dd* (doublet of doublets), *dt* (doublet of triplets), *q* (quartet), *m* (multiplet), and *br* (broad signal). Carbon spectra were acquired with complete decoupling for the proton. Infrared spectra (IR) were recorded (i) on a Shimadzu IR Affinity 1S FTIR spectrometer in ATR mode with a diamond monocrystal at Cardiff University and (ii) on a BRUKER VERTEX FTIR spectrometer in ATR mode at Vienna University. Selected absorption bands are reported in wavenumbers (cm^–1^). UV–vis absorption spectroscopy was recorded on an Agilent Cary 5000 UV–vis–NIR spectrophotometer running in double beam mode with a matched pair of quartz absorbance cuvettes (1 × 1 cm). All absorption measurements were performed at 20 °C. Mass spectrometry: (i) high-resolution ESI mass spectra (HRMS) were obtained on a Waters LCT HR TOF mass spectrometer in the positive or negative ion mode at Cardiff University. (ii) High-resolution mass analyses were performed at the Mass Spectrometry Centre of the University of Vienna. ESI mass spectra were obtained on a Bruker maXis UHR ESI-Qq-S3 TOF mass spectrometer in the positive ion mode, GC mass spectra on an Agilent 7200B GC/Q-TOF mass spectrometer, and LD and MALDI mass spectra on a Bruker Autoflex Speed LD-timsTOF or MALDItimsTOF (matrix: 2-[(2E)-3-(4-*tert*-butylphenyl)-2-methylprop-2-enylidene]malononitrile) mass spectrometer. The sum formulas of the detected ions were determined using Bruker Compass DataAnalysis 4.1 based on the mass accuracy (Δ*m*/*z* ≤ 5 ppm) and isotopic pattern matching (SmartFormula algorithm). HRLDMS spectra were acquired on a timsTOF fleX ESI/MALDI dual source—trapped ion mobility separation—Qq-TOF mass spectrometer (Bruker Daltonics, Bremen, Germany) in the positive ion mode. The sum formulas of the detected ions were determined using Bruker Compass DataAnalysis 5.3 based on the mass accuracy (Δ*m*/*z* ≤ 5 ppm) and isotopic pattern matching (SmartFormula algorithm).

### X-ray

The crystal structures of products **4j** and **4n** were determined at the Centre for X-ray Structure Analysis of the University of Vienna. X-ray intensity data were measured at 100 K on a STOE Stradivari diffractometer equipped with dual radiation sources Mo and Cu Kα and a Dectris EIGER2 R 500 K detector. The structures were solved ab initio and refined by full-matrix least-squares techniques. Hydrogen atoms were inserted at calculated positions using AFIX instructions, while all other atoms were refined with anisotropic displacement parameters. Measurements of the crystal structures of products **14c/PXX** (adduct), **PXXa**, and **PXXb** were performed at the XRD2 beamline of the Elettra Synchrotron, Trieste (Italy). The crystals were dipped in NHV oil (Jena Bioscience, Jena, Germany) and mounted on the goniometer head with Kapton loops (MiTeGen, Ithaca, USA). Complete data sets were collected at 100 K (nitrogen stream supplied through an Oxford Cryostream 700) through the rotating crystal method. Data were acquired using a monochromatic wavelength of 0.620 Å on a Pilatus hybrid-pixel area detector (DECTRIS Ltd., Baden-Daettwil, Switzerland). The diffraction data were indexed and integrated using XDS. Semiempirical absorption corrections and scaling were performed on **14c/PXX** (adduct) data sets, exploiting multiple measures of symmetry-related reflections, using SADABS. The structures were solved by the dual-space algorithm implemented in the SHELXT code. Fourier analysis and refinement were performed by the full-matrix least-squares methods based on F2 implemented in SHELXL (Version 2018/3). The Coot program was used for modeling. Anisotropic thermal motion refinement has been used for all atoms. Hydrogen atoms were included at calculated positions with isotropic *U*_factors_ = 1.2·*U*_eq_ or *U*_factors_ = 1.5·*U*_eq_ (for methyl groups; *U*_eq_ being the equivalent isotropic thermal factor of the bonded nonhydrogen atom). Pictures were prepared using Ortep-36 Mercury 4.07 software. Essential crystal and refinement data are fully reported in the Supporting Information.

## Supporting Information Available

Synthetic procedures, NMR and HRMS spectra, photophysical and electrochemical characterizations, and crystallographic data (PDF)
