# Supplementary material for: Photoredox Annulation of Polycyclic Aromatic Hydrocarbons
Source: JACS Au. 2023 Nov 14;3(11):3045–54. doi: 10.1021/jacsau.3c00438 (PMC10685425; doi:10.1021/jacsau.3c00438)
Supplement: Supplementary file 1 — au3c00438_si_001.pdf [file au3c00438_si_001.pdf]

Photoredox annulation of polycyclic aromatic hydrocarbons

Davide Zanetti, Oliwia Matuszewska, Giuliana Giorgianni, Cristofer Pezzetta, Nicola Demitri and Davide Bonifazi*

* Dr. O. Matuszewska, Dr. C. Pezzetta: School of Chemistry, Cardiff University, Main Building, Park Place, Cardiff CF10 3AT, United Kingdom.

Dr. N. Demitri: Elettra – Sincrotrone Trieste, 34149 Trieste, Italy

D. Zanetti, Dr. G. Giorgianni, Prof. Dr. Davide Bonifazi: Institute of Organic Chemistry, University of Vienna, 1090 Vienna, Austria. E-mail: davide.bonifazi@univie.ac.at

1. GENERAL INFORMATION **5**

INSTRUMENTATION 5

EQUIPMENT 7

2. EXPERIMENTAL PROCEDURES **8**

GENERAL PROCEDURE I 8

Synthesis of methyl 2'-bromo-[1,1'-biphenyl]-4-carboxylate *9*

Synthesis of methyl 2-bromo-[1,1'-biphenyl]-4-carboxylate *9*

Synthesis of 2-bromo-4'-(tert-butyl)-1,1'-biphenyl *10*

Synthesis of 2-bromo-3'-methoxy-1,1'-biphenyl *10*

Synthesis of 2-bromo-4-methoxy-1,1'-biphenyl *11*

Synthesis of 2'-bromo-6-chloro-[1,1'-biphenyl]-3-carbonitrile *11*

Synthesis of 1-(2,6-dibromophenyl)-1H-pyrrole *12*

Synthesis of 2',6'-dibromo-N,N-dimethyl-[1,1'-biphenyl]-4-amine *12*

GENERAL PROCEDURE II 13

Synthesis of 2-chloro-1,1':2',1''-terphenyl (1a) *13*

Synthesis of 6-chloro-[1,1':2',1''-terphenyl]-3-carbonitrile (1) *13*

Synthesis of 2-chloro-[1,1':2',1''-terphenyl]-3-carbonitrile (3a) *14*

Synthesis of 2-chloro-[1,1':2',1''-terphenyl]-4-carbonitrile (3b) *14*

Synthesis of 6-chloro-[1,1':2',1''-terphenyl]-3,4-dicarbonitrile (3c) *15*

Synthesis of methyl 6-chloro-[1,1':2',1''-terphenyl]-4-carboxylate (3d) *15*

Synthesis of methyl 6-chloro-[1,1':2',1''-terphenyl]-3-carboxylate (3e) *16*

Synthesis of 6-chloro-[1,1':2',1''-terphenyl]-3,4''-dicarbonitrile (3f) *16*

Synthesis of methyl 2''-chloro-5''-cyano-[1,1':2',1''-terphenyl]-4-carboxylate (3g) *17*

Synthesis of methyl 2''-chloro-5''-cyano-[1,1':2',1''-terphenyl]-4'-carboxylate (3j) *18*

Synthesis of 4''-(tert-butyl)-6-chloro-[1,1':2',1''-terphenyl]-3-carbonitrile (3k) *18*

Synthesis of 6-chloro-4''-methoxy-[1,1':2',1''-terphenyl]-3-carbonitrile (3l) *19*

Synthesis of 6-chloro-3''-methoxy-[1,1':2',1''-terphenyl]-3-carbonitrile (3m) *19*

Synthesis of 6-chloro-2'',4''-dimethoxy-[1,1':2',1''-terphenyl]-3-carbonitrile (3n) *20*

Synthesis of 6-chloro-5'-methoxy-[1,1':2',1''-terphenyl]-3-carbonitrile (3o) *20*

Synthesis of 6-chloro-4'',5'-bis(dimethylamino)-[1,1':2',1''-terphenyl]-3-carbonitrile (3p) *21*

Synthesis of 6,6''-dichloro-[1,1':2',1''-terphenyl]-3,3''-dicarbonitrile (3q) *21*

Synthesis of 4'-bromo-2,4,6-trimethyl-1,1'-biphenyl *22*

Synthesis of N-(2',4',6'-trimethyl-[1,1'-biphenyl]-4-yl)-[1,1'-biphenyl]-2-amine *22*

Synthesis of 4-chloro-3-(5-(2',4',6'-trimethyl-[1,1'-biphenyl]-4-yl)dibenzo[c,e] [1,2]azaborinin-6(5H)-yl)benzonitrile (3r) *23*

Synthesis of 6-chloro-2'-(1H-pyrrol-1-yl)-[1,1'-biphenyl]-3-carbonitrile (5a) *24*

Synthesis of 6-chloro-2'-(thiophen-3-yl)-[1,1'-biphenyl]-3-carbonitrile (5b) *24*

Synthesis of 6-chloro-2'-(pyridin-3-yl)-[1,1'-biphenyl]-3-carbonitrile (5c) *25*

Synthesis of 6-chloro-2'-(naphthalen-2-yl)-[1,1'-biphenyl]-3-carbonitrile (7a) 25
Synthesis of 2'-(anthracen-2-yl)-6-chloro-[1,1'-biphenyl]-3-carbonitrile (7b) 26
Synthesis of 6-chloro-2'-(pyren-1-yl)-[1,1'-biphenyl]-3-carbonitrile (7c) 27
Synthesis of 6-chloro-2'-(perylen-1-yl)-[1,1'-biphenyl]-3-carbonitrile (7d) 27
Synthesis of 2',5'-dibromo-1,1':4',1''-terphenyl 28
Synthesis of 2,2''-dibromo-4,4''-dimethoxy-1,1':4',1''-terphenyl 29
GENERAL PROCEDURE III 29
Synthesis of 6,6''''-dichloro-[1,1':2',1'':4'',1''':2''',1''''-quinquephenyl]-3,3''''-dicarbonitrile (9a) 30
Synthesis of 2,2''''-dichloro-4''',5'-dimethoxy-[1,1':2',1'':4'',1''':2''',1''''-quinquephenyl]-4,4''''-dicarbonitrile (9b) 30
Synthesis of dimethyl 2,2''-dichloro-2',5'-diphenyl-[1,1':4',1''-terphenyl]-4,4''-dicarboxylate (10a) 31
Synthesis of dimethyl 2,2''-dichloro-2'-(4-(dimethylamino)phenyl)-[1,1':3',1''-terphenyl]-4,4''-dicarboxylate (12a) 31
Synthesis of dimethyl 2,2''-dichloro-2'-(1H-pyrrol-1-yl)-[1,1':3',1''-terphenyl]-4,4''-dicarboxylate (12b) 32
Synthesis of 6,6'''-dichloro-[1,1':2',1'':2'',1'''-quaterphenyl]-3,3'''-dicarbonitrile (13a) 32
GENERAL PROCEDURE IV 33
Synthesis of triphenylene-2-carbonitrile (2) 33
Synthesis of triphenylene-1-carbonitrile (4a) 34
Synthesis of triphenylene-2-carbonitrile (4b) 34
Synthesis of triphenylene-2,3-dicarbonitrile (4c) 35
Synthesis of methyl triphenylene-2-carboxylate (4d) 35
Synthesis of methyl triphenylene-2-carboxylate (4e) 35
Synthesis of triphenylene-2,6-dicarbonitrile (4f) 36
Synthesis of methyl 10-cyanotriphenylene-2-carboxylate (4g) 36
Synthesis of methyl 11-cyanotriphenylene-2-carboxylate (4j) 37
Synthesis of 6-(tert-butyl)triphenylene-2-carbonitrile (4k) 37
Synthesis of 6-methoxytriphenylene-2-carbonitrile (4l) 38
Synthesis of 7-methoxytriphenylene-2-carbonitrile (4m) 38
Synthesis of 6,8-dimethoxytriphenylene-2-carbonitrile (4n) 39
Synthesis of 11-methoxytriphenylene-2-carbonitrile (4o) 39
Synthesis of 6,11-bis(dimethylamino)triphenylene-2-carbonitrile (4p) 40
Synthesis of triphenylene-1,7-dicarbonitrile (4q) 40
Synthesis of 8-mesityldibenzo[c,e]dibenzo[3,4:5,6][1,2]azaborinino[1,2-a] [1,2]azaborinine-12-carbonitrile (4r) 41
Synthesis of pyrrolo[1,2-f]phenanthridine-10-carbonitrile (6a) 41
Synthesis of phenanthro[9,10-b]thiophene-9-carbonitrile (6b) and phenanthro[9,10-c]thiophene-6-carbonitrile (6b') . 42
Synthesis of dibenzo[f,h]quinoline-10-carbonitrile (6c) 42
Synthesis of benzo[f]tetraphene-3-carbonitrile (8a) 43
Synthesis of dibenzo[a,c]tetracene-3-carbonitrile (8b) 43
Synthesis of dibenzo[f,pqr]picene-9-carbonitrile (8c) 44
Synthesis of 6-(2'-chloro-5'-cyano-[1,1'-biphenyl]-2-yl)triphenylene-2-carbonitrile 11a) 44
Synthesis of 6,15-dimethoxytribenzo[f,k,m]tetraphene-3,12-dicarbonitrile (11b) 45
Syntehsis of dimethyl tribenzo[f,k,m]tetraphene-2,11-dicarboxylate (10a) 45

Syntehsis of dimethyl 2-(dimethylamino)dibenzo[fg,op]tetracene-5,13-dicarboxylate (14a)......................................46
Syntehsis of dimethyl benzo[7,8]indolizino[6,5,4,3-def]phenanthridine-4,12-dicarboxylate (14b) 46
Synthesis of dimethyl dibenzo[fg,op]tetracene-5,12-dicarboxylate (14c)..47
Synthesis of peri-Xanthenoxanthene (PXX) 47

CHARACTERIZATION...48

OPTIMIZATION SPECTRA (TABLE 1, MAIN TEXT)..48
CHARACTERIZATION OF CLEAN COMPOUNDS 59

3. PHOTOPHYSICAL CHARACTERIZATIONS AND STERN-VOLMER ANALYSIS 201

4. ELECTROCHEMICAL ANALYSIS 203

5. CRYSTALLOGRAPHIC DATA...204

6. SUPPLEMENTARY DATA 209

7. REFERENCES...212

1. General information

Instrumentation

Thin layer chromatography (TLC) was conducted on pre-coated aluminum sheets with 0.20 mm *Machevery-Nagel* Alugram SIL G/UV254 with fluorescent indicator UV254. **Column chromatography** was carried out using *Merck Gerduran* silica gel 60 (particle size 63-200 mm). **Melting points** (mp) were measured on a Gallenkamp apparatus in open capillary tubes and have not been corrected. **Nuclear magnetic resonance** (NMR): (i) ^{1}H and ^{13}C spectra were obtained a Bruker Fourier 300 MHz spectrometer equipped with a dual (^{13}C, ^{1}H) probe at Cardiff University. (ii) ^{1}H and ^{13}C spectra were obtained a Bruker AVANCE III HD 400MHz NMR spectrometer equipped with a Broadband multinuclear (BBFO) SmartProbe™, a Bruker AVANCE III HD 500MHz Spectrometer equipped with Broadband multinuclear (BBO) Prodigy CryoProbe or a Bruker AV III HDX 700 NMR spectrometer (Bruker BioSpin, Rheinstetten, Germany) at Vienna University. Chemical shifts were reported in ppm according to tetramethylsilane using the solvent residual signal as an internal reference ($CDCl_3$: δ_H = 7.26 ppm, δ_C = 77.16 ppm). Coupling constants (*J*) were given in Hz. Resonance multiplicity was described as *s* (singlet), *d* (doublet), *t* (triplet), *dd* (doublet of doublets), *dt* (doublet of triplets), *q* (quartet), *m* (multiplet) and *br* (broad signal). Carbon spectra were acquired with a complete decoupling for the proton. **Infrared spectra** (IR) were recorded on a Shimadzu IR Affinity 1S FTIR spectrometer in ATR mode with a diamond mono-crystal at Cardiff University. IR spectra were also recorded on a BRUKER Vertex FTIR spectrometer in ATR mode at Vienna University. Selected absorption bands are reported in wavenumbers (cm_{-1}). **UV-Vis Absorption spectroscopy** was recorded on Agilent Cary 5000 UV-Vis-NIR Spectrophotometer running in double beam mode with a matched pair of quartz absorbance cuvettes (1 x 1 cm). All absorption measurements were performed at 20 °C. **Mass spectrometry**: (i) High-resolution ESI mass spectra (HRMS) were performed on a Waters LCT HR TOF mass spectrometer in the positive or negative ion mode at Cardiff University. (ii) High-resolution mass analyses were performed at the Mass Spectrometry Centre of the University of Vienna. ESI mass spectra were obtained on a Bruker maXis UHR ESI-Qq- S3 TOF mass spectrometer in the positive ion mode, GC mass spectra on an Agilent 7200B GC/Q-TOF mass spectrometer, LD and MALDI mass spectra on a Bruker Autoflex Speed LD-timsTOF or MALDItimsTOF (matrix: 2-[(2E)-3-(4-tert-butylphenyl)-2-methylprop-2-enylidene]malononitrile (DCTB)) mass spectrometer. The sum formulas of the detected ions were determined using Bruker Compass DataAnalysis 4.1 based on the mass accuracy ($\Delta m/z \leq 5$ ppm) and isotopic pattern matching (SmartFormula algorithm). HRLDMS spectra were acquired on a timsTOF fleX ESI/MALDI dual source - trapped ion mobility separation - Qq-TOF mass spectrometer (Bruker Daltonics, Bremen, Germany) in the positive ion mode. The sum formulas of the detected ions were determined using Bruker Compass DataAnalysis 5.3 based on the mass accuracy ($\Delta m/z \leq 5$ ppm) and isotopic pattern matching (SmartFormula algorithm).

Synthesis Chemicals were purchased from *BLDpharm, Sigma Aldrich*, *Acros Organics*, *TCI, Apollo Scientific, Fluorochem,* and *ABCR* and were used without any further purification. Solvents were purchased from *Sigma Aldrich*, while deuterated solvents from *Eurisotop*. MeOH, $CHCl_3$ and acetone

were purchased as reagent-grade and used without further purification. 0 °C temperature baths were prepared using ice/H_2O. Anhydrous conditions were achieved by drying Schlenk tubes or round-bottom flasks in an oven at 120 °C for at least 4 h, followed by evacuation under vacuum and purging with argon. Inert atmosphere was maintained using Argon-filled balloons equipped with a syringe and needle that was used to pierce the silicon stoppers used to close the flasks's necks. **X-Ray measurements** Cristal structures of the products 4j and 4n were performed at the Centre for X-ray Structure Analysis of the University of Vienna. X-ray intensity data were measured at 100 K on a STOE Stadivari diffractometer equipped with dual radiation source Mo and Cu Kα, and a Dectris EIGER2 R 500K detector. The structures were solved ab initio and refined by full-matrix least-squares techniques. Hydrogen atoms were inserted at calculated positions using AFIX instructions, while all other atoms were refined with anisotropic displacement parameters. measurements Cristal structures of the products 14c/PXX (adduct), PXXa and PXXb were performed at the XRD2 beamline of the Elettra Synchrotron, Trieste (Italy). The crystals were dipped in NHV oil (Jena Bioscience, Jena, Germany) and mounted on the goniometer head with kapton loops (MiTeGen, Ithaca, USA). Complete datasets were collected at 100 K (nitrogen stream supplied through an Oxford Cryostream 700) through the rotating crystal method. Data were acquired using monochromatic wavelength of 0.620 Å on a Pilatus hybrid-pixel area detector (DECTRIS Ltd., Baden-Daettwil, Switzerland). The diffraction data were indexed and integrated using XDS.[1] Semi-empirical absorption corrections and scaling were performed on 14c/PXX (adduct) datasets, exploiting multiple measures of symmetry-related reflections, using SADABS.[2] The structures were solved by the dual space algorithm implemented in the SHELXT code.[3] Fourier analysis and refinement were performed by the full-matrix least-squares methods based on F2 implemented in SHELXL (Version 2018/3).[4] The Coot program was used for modeling.[5] Anisotropic thermal motion refinement has been used for all atoms. Hydrogen atoms were included at calculated positions with isotropic Ufactors = 1.2•Ueq or Ufactors = 1.5•Ueq (for methyl groups; Ueq being the equivalent isotropic thermal factor of the bonded non hydrogen atom). Pictures were prepared using Ortep-3[6] Mercury 4.0[7] software. Essential crystal and refinement data are reported below.

Equipment

All batch photocatalytic studies were performed in a custom-built photoreactor setup featuring a single high-power blue LED (1260 mW, 4.4 x 4.4 mm, 405 nm, LED Engin) as the light source used for illuminating the reaction vessel from the bottom. The LED was passively cooled by the attached aluminium heatsink, while the reaction temperature was held constant by circulating a cooling fluid through the aluminium support connected to a chiller. The inspiration came from the custom-made setup reported by Melchiorre´s research group.[8]

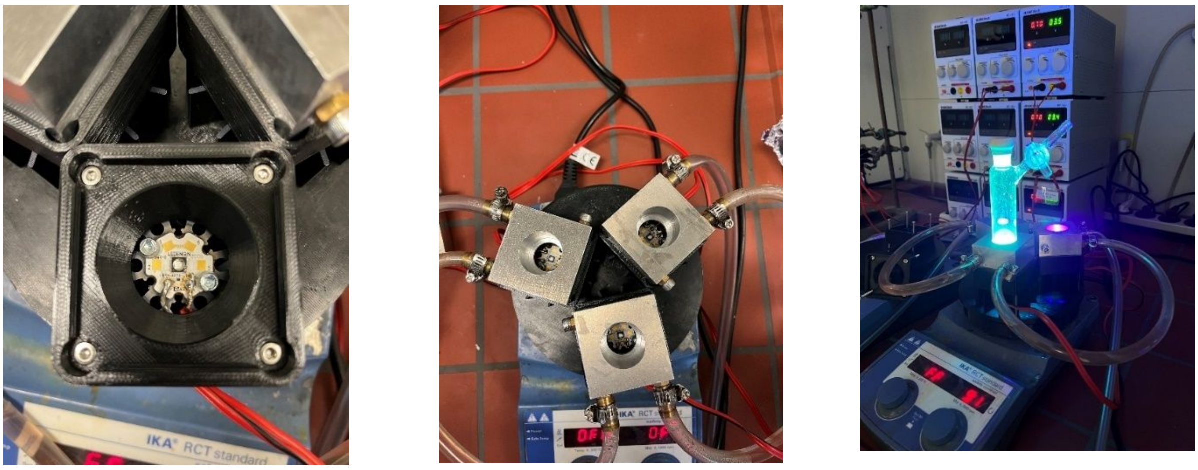

Figure S1. Reaction setup for the photocyclization reactions.

2. Experimental procedures

General procedure I

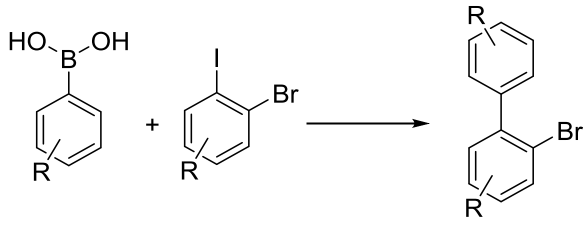

The mixture of the relevant boronic acid (1 eq), aryl iodine (1.2 eq) and base (2-2.5 eq) in the given solvent is degassed with Ar bubbling for 15 min. The given Pd catalyst (0.02-0.1 eq) is added, and the mixture is degassed for additional 5 min out. After the given reaction time, the mixture is cooled to rt, water is added, and the mixture is extracted with EtOAc (3x10 mL). The organic phase is dried over $MgSO_4$, filtered, and concentrated in vacuo. The product was isolated by flash chromatography on SiO_2 using a mixture of heptane/EtOAc or heptane/CH_2Cl_2 as eluent.

Synthesis of 2'-bromo-[1,1'-biphenyl]-4-carbonitrile

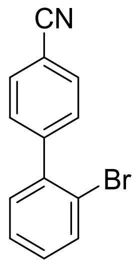

Synthesized in accordance with *general procedure I,* using (4-(methoxycarbonyl)phenyl)boronic acid (432 mg, 2.4 mmol), 1-bromo-2-iodobenzene (566 mg, 2.0 mmol), [Pd(dppf)Cl_2] (73 mg, 0.1 mmol), K_2CO_3 (553 mg, 4.0 mmol), toluene (15 mL) and MeOH (8 mL). The reaction mixture was stirred at 110 °C for 24 h. The desired product was purified by flash chromatography heptane/EtOAc 30:10. The pure compound was obtained as a white powder (431 mg, 1.5 mmol, yield: 74%). ^{1}H-NMR (300 MHz, $CDCl_3$) δ 7.77 – 7.70 (m, 3H), 7.57 – 7.53 (m, 2H), 7.45 (ddd, J = 8.0, 7.1, 2.1 Hz, 1H), 7.33 – 7.26 (m, 2H); ^{13}C-NMR (75 MHz, $CDCl_3$) δ 145.7, 140.8, 133.5, 132.0, 131.1, 130.4, 129.9, 127.8, 122.2, 118.9, 111.6. LRMS (APCI$^+$): *m/z* calcd for $C_{13}H_8BrN$ [M+H]$^+$: 258.98; found: 258.98. Spectroscopic characterization in accordance with literature.[9]

Synthesis of methyl 2'-bromo-[1,1'-biphenyl]-4-carboxylate

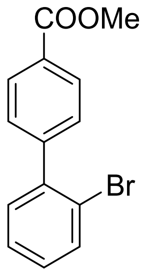

Synthesized in accordance with *general procedure I,* using (4-(methoxycarbonyl)phenyl)boronic acid (432 mg, 2.4 mmol), 1-bromo-2-iodobenzene (566 mg, 2.0 mmol), [Pd(dppf)Cl_2] (73 mg, 0.1 mmol), K_2CO_3 (553 mg, 4.0 mmol), toluene (15 mL) and MeOH (8 mL). The reaction mixture was stirred at 110 °C for 24 h. The desired product was purified by flash chromatography heptane/EtOAc 30:10. The pure compound was obtained as a white powder (431 mg, 1.5 mmol, yield: 74%). ^{1}H-NMR (300 MHz, $CDCl_3$) δ 8.03 – 8.00 (m, 2H), 7.59 (ddd, J = 8.0, 1.2, 0.4 Hz, 1H), 7.42 – 7.38 (m, 2H), 7.29 – 7.22 (m, 2H), 7.14 (ddd, J = 8.0, 7.1, 2.1 Hz, 1H), 3.85 (s, 3H); ^{13}C-NMR (75 MHz, $CDCl_3$) δ 167.0, 145.7, 141.7, 133.4, 131.1, 129.6, 129.4, 129.4, 127.6, 122.3, 52.3 (1 carbon peak is missing due to overlap). HRMS (APCI): *m/z* calcd for $C_{14}H_{11}BrO_2$ [M+H]$^+$: 291.0021; found: 291.0028. Spectroscopic characterization in accordance with literature.[10]

Synthesis of methyl 2-bromo-[1,1'-biphenyl]-4-carboxylate

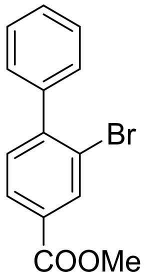

Phenylboronic acid (128 mg, 1.1 mmol), methyl 3-bromo-4-iodobenzoate (340 mg, 1.0 mmol), [Pd(PPh_3)$_2$$Cl_2$] (14 mg, 0.02 mmol), Et_3N (0.4 mL, 3.0 mmol) and Kolliphor (2% w/w) were dissolved in 0.5 mL of toluene and 4.5 mL of H_2O. After sonicating for 30 seconds, the mixture was stirred at 60 °C for 2 h. Later the mixture was cooled down to room temperature and filtered over a silica plug using CH_2Cl_2 as eluent. The desired product was purified by flash chromatography using heptane. The pure compound was obtained as white solid (275 mg, 0.9 mmol, yield: 95%), m.p. 101 – 103 °C. ^{1}H-NMR (700 MHz, $CDCl_3$) δ 8.35 (d, J = 1.7 Hz, 1H), 8.01 (dd, J = 8.0, 1.7 Hz, 1H), 7.48 – 7.35 (m, 6H), 3.94 (s, 3H). ^{13}C-NMR (176 MHz, $CDCl_3$) δ 156.7, 147.0, 140.2, 134.3, 131.2, 130.6, 129.2, 128.5, 128.2, 128.1, 122.6, 52.4. IR (ATR): ν (cm^{-1}) = 1598, 1463, 1440, 1288, 1254, 1032, 1004, 880, 816, 762, 695, 580, 538, 457 cm^{-1}. HRMS (APCI) *m/z* calcd for $C_{14}H_{11}BrO_2$ [M+H]$^+$: 291.0015; found: 291.0018. Spectroscopic characterization in accordance with literature.[10]

Synthesis of 2-bromo-4'-(tert-butyl)-1,1'-biphenyl

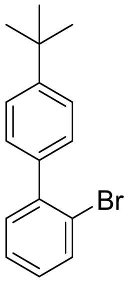

Synthesized in accordance with *general procedure I,* using (4-(tert-butyl)phenyl)boronic acid (320 mg, 1.8 mmol), 1-bromo-2-iodobenzene (424 mg, 1.5 mmol), [Pd(PPh_3)$_2$$Cl_2$] (53 mg, 0.08 mmol), K_2CO_3 (415 mg, 3.0 mmol), toluene (10 mL) and MeOH (5 mL). The reaction mixture was stirred at 110 °C for 24 h. The desired product was purified by flash chromatography using heptane. The pure compound was obtained as a white powder (413 mg, 1.4 mmol, yield: 95%). ^{1}H-NMR (300 MHz, $CDCl_3$) δ 7.68 – 7.65 (m, 1H), 7.47 – 7.43 (m, 2H), 7.39 – 7.32 (m, 4H), 7.22 – 7.16 (m, 1H), 1. 38 (s, 9H). ^{13}C-NMR (75 MHz, $CDCl_3$) δ 150.6, 142.6, 138.2, 133.3, 131.6, 129.1, 128.6, 127.5, 125.0, 122.8, 34.8, 31.5. LRMS (APCI): *m/z* calcd for $C_{16}H_{17}Br$ $[M]^+$: 288.05; found: 288.05. Spectroscopic characterization in accordance with literature.[9]

Synthesis of 2-bromo-3'-methoxy-1,1'-biphenyl

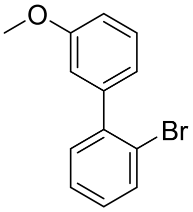

Synthesized in accordance with *general procedure I,* using (3-methoxyphenyl)boronic acid (380 mg, 2.5 mmol), 1-bromo-2-iodobenzonitrile (0.4 mL, 3.0 mmol), [Pd(PPh_3)$_4$] (144 mg, 0.13 mmol), K_2CO_3 (864 mg, 6.3 mmol), THF (8 mL) and H_2O (2 mL). The mixture was stirred at 110 °C for 24 h. The desired product was purified by flash chromatography using a mixture of heptane/EtOAc whit gradient from 100% to 95:5. The pure compound was obtained as dense colourless oil (633 mg, 2.4 mmol, yield: 96%). ^{1}H-NMR (700 MHz, $CDCl_3$) δ 7.71 (d, J = 7.9 Hz, 1H), 7.41 – 7.35 (m, 3H), 7.23 (ddd, J = 8.1, 5.7, 3.4 Hz, 1H), 7.04 – 6.98 (m, 3H), 3.88 (s, 3H). ^{13}C-NMR (176 MHz, $CDCl_3$) δ 159.1, 142.4, 142.3, 133.1, 131.1, 128.9, 128.7, 127.3, 122.5, 121.8, 115.0, 113.2, 55.2. MS (GC-MS): *m/z* calcd for $C_{13}H_{11}BrO$ $[M]^+$: 262.01; found: 262.00. Spectroscopic characterization in accordance with literature.[10]

Synthesis of 2-bromo-4-methoxy-1,1'-biphenyl

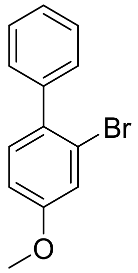

Synthesized in accordance with *general procedure I,* using phenylboronic acid (366 mg, 3.0 mmol), 2-bromo-1-iodo-4-methoxybenzene (0.56 mL, 3.6 mmol), [Pd_2(dba)$_3$] (275 mg, 0.3 mmol), K_2CO_3 (1.0 g, 7.5 mmol), toluene (5 mL) and H_2O (5 mL). The mixture was stirred at 110 °C for 24 h. The desired product was purified by flash chromatography using heptane. The pure compound was obtained as white solid (255 mg, 1.0 mmol, yield: 54%), m.p. 101 – 103 °C. ^{1}H-NMR (700 MHz, $CDCl_3$) δ 7.47 – 7.41 (m, 4H), 7.41 – 7.37 (m, 1H), 7.30 – 7.24 (m, 2H), 6.94 (dd, J = 8.5, 2.2 Hz, 1H), 3.86 (s, 3H). ^{13}C-NMR (176 MHz, $CDCl_3$) δ 159.2, 140.8, 135.0, 131.6, 129.6, 127.9, 127.2, 122.7, 118.2, 113.5, 55.5. IR (ATR): υ (cm^{-1}) = 1598, 1463, 1440, 1288, 1254, 1032, 1004, 880, 816, 762, 695, 580, 538, 457 cm^{-1}. HRMS (ESI): *m/z* calcd for $C_{13}H_{11}BrO$ [*M*+H]$^+$: 263.0975; found: 263.0066.

Synthesis of 2'-bromo-6-chloro-[1,1'-biphenyl]-3-carbonitrile

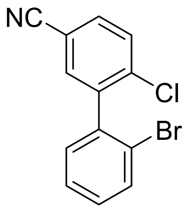

(2-chloro-5-cyanophenyl)boronic acid (147 mg, 1 mmol), 3-bromo-4-iodobenzonitrile (370 mg, 0,9 mmol), [$PdCl_2$(dtbpf)] (13 mg, 0.02 mmol), NEt_3 (0.418 mL, 3.0 mmol) and Kolliphor (2% w/w) were dissolved in 0.5 mL of toluene and 4.5 mL of H_2O. After sonicating for 30 seconds, the mixture was stirred at 60 °C for 2 h. Later the mixture was cooled down to room temperature and filtered over a silica plug using CH_2Cl_2 as eluent. The desired product was purified by flash chromatography using a mixture of heptane/EtOAc whit gradient from 100% to 90:10. The pure compound was obtained as white solid (277 mg, 0.95 mmol, yield: 95%), m. p. 135 - 137 °C. ^{1}H-NMR (400 MHz, $CDCl_3$) δ 7.70 (dd, J = 8.0, 1.1 Hz, 1H), 7.64 (dd, J = 8.3, 1.8 Hz, 1H), 7.59 (d, J = 8.3 Hz, 1H), 7.58 – 7.55 (d, J = 1.8 Hz, 1H), 7.41 (td, J = 7.5, 1.2 Hz, 1H), 7.31 (td, J = 7.5, 1.8 Hz, 1H), 7.23 (dd, J = 7.5, 1.7 Hz, 1H). ^{13}C-NMR (101 MHz, $CDCl_3$) δ 149.4, 145.0, 133.8, 131.0 129.2, 128.6, 128.5, 127.6, 126.6, 123.5, 123.5, 123.4, 112.2. IR (ATR): υ (cm^{-1}) = 2213, 1641, 1476, 1399, 1245, 1055, 755, 740, 578, 554, 443. HRMS (ESI): m/z calcd for $C_{13}H_7BrClN$ [*M*+Na]$^+$: 315.9321; found: 315.9318.

Synthesis of 1-(2,6-dibromophenyl)-1H-pyrrole

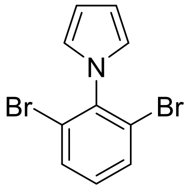

To a 200 mL round bottom flask was added 2,6-dibromoaniline (502 mg, 2.0 mmol), dichloroethane (5.0 mL), acetic acid (5.0 mL), and 2,5-dimethoxytetrahydrofuran (0.4 mL, 3.0 mmol). The mixture was heated to reflux for 2 hours. The mixture was diluted with CH_2Cl_2, rinsed with H_2O, and then rinsed with saturated aqueous K_2CO_3. The organic layer was dried over $MgSO_4$ and filtered through a pad of SiO_2 with CH_2Cl_2 as eluent. The oil was place under high vacuum overnight to remove the excess 2,5-dimethoxytetrahydrofuran. During this time 1-(2,6 dibromophenyl)-1H-pyrrole, changes to a white solid (524 mg, 1.7 mmol, yield: 84%). ^{1}H-NMR (600 MHz, $CDCl_3$) δ 7.65 (d, *J* = 8.1 Hz, 2H), 7.15 (t, *J* = 8.1 Hz, 1H), 6.69 (t, *J* = 2.1 Hz, 2H), 6.38 (t, *J* = 2.1 Hz, 2H).^{13}C-NMR (151 MHz, $CDCl_3$) δ 132.4, 130.5, 124.2, 121.5, 109.3 (1 carbon peak is missing due to overlap). HRMS (ESI): m/z calcd for $C_{10}H_7Br_2N$ [*M*]+: 301.8998; found: 301.8999. Spectroscopic characterization in accordance with literature.[11]

Synthesis of 2',6'-dibromo-N,N-dimethyl-[1,1'-biphenyl]-4-amine

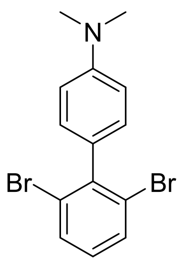

Synthesized in accordance with *general procedure I,* using 4-(dimethylamino)phenylboronic acid pinacol ester (257 mg, 1.0 mmol), 1,3-dibromo-2-iodo-benzene (470 mg, 1.3 mmol), [Pd(PPh$_3$)$_4$] (75 mg, 0.1 mmol), Na_2CO_3 (482 mg, 4.6 mmol), DME (40 mL), EtOH (5 mL) and H_2O (15 mL). The mixture was stirred at 100 °C for 24 h. The desired product was purified by flash chromatography using a mixture of heptane/EtOAc whit gradient from 100% to 95:5. The pure compound was obtained as dense colorless oil (633 mg, 2.4 mmol, yield: 96%). The desired product was purified by flash chromatography using a mixture of petroleum ether/ethyl acetate whit gradient from 100% to 90:10. The pure compound was obtained as white solid (120 mg, 0.3 mmol, yield: 42%). ^{1}H-NMR (600 MHz, $CDCl_3$) δ 7.61 (d, *J* = 8.0 Hz, 2H), 7.13 – 7.05 (m, 2H), 7.01 (t, *J* = 8.0 Hz, 1H), 6.82 – 6.74 (m, 2H), 3.03 (s, 6H). ^{13}C-NMR (151 MHz, $CDCl_3$) δ 149.8, 143.3, 131.8, 130.0, 129.3, 125.6, 111.3, 40.3 (1 carbon peak is missing due to overlap). HRMS (ESI): m/z calcd for $C_{14}H_{13}Br_2N$ [*M*]$^+$: 355.9468; found: 355.9470. Spectroscopic characterization in accordance with literature.[12]

General procedure II

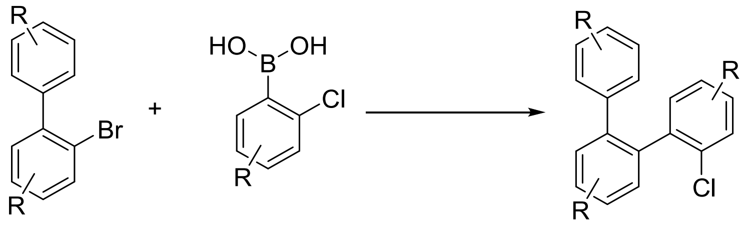

The mixture of the relevant boronic acid (1-3 eq), aryl iodine/bromine (1-1.2 eq), base (2-2.5 eq) in the given solvent is degassed with Ar bubbling for 15 min. The given Pd catalyst (0.04-0.1 eq) is added, and the mixture is degassed for additional 5 min out. After the given reaction time, the mixture is cooled to rt, water is added, and the mixture is extracted with EtOAc (3x10 mL). The organic phase is dried over $MgSO_4$, filtered, and concentrated in vacuo. The product was isolated by flash chromatography on SiO_2 using a mixture of heptane/EtOAc or heptane/CH_2Cl_2 as eluent.

Synthesis of 2-chloro-1,1':2',1"-terphenyl (1a)

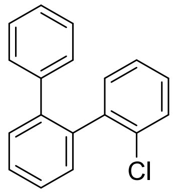

2-Bromobiphenyl (233.1 mg, 1.0 mmol), 2-Chlorophenylboronic acid (234.6 mg, 1.5 mmol), [$PdCl_2$(dtbpf)] (13.0 mg, 0.02 mmol), NEt_3 (0.42 mL, 3.0 mmol) and Kolliphor (2% w/w) were dissolved in 1 mL of toluene and 9.5 mL of H_2O. After sonicating for 30 seconds, the mixture was stirred at 60 °C for 2 h. Later the mixture was cooled down to room temperature and filtered over a silica plug using CH_2Cl_2 as eluent. The desired product was purified by flash chromatography using a mixture of heptane/EtOAc whit gradient from 100% to 90:10. The pure compound was obtained as a colorless oil (232 mg, 0.88 mmol, yield: 88%), m.p. 100-102 °C. ^{1}H-NMR (600 MHz, $CDCl_3$) δ 7.50 – 7.45 (m, 2H), 7.43 (m, 1H), 7.38 – 7.31 (m, 2H), 7.22 – 7.07 (m, 8H). ^{13}C-NMR (151 MHz, $CDCl_3$) δ 141.4, 141.1, 140.0, 137.9, 133.4, 132.2, 130.8, 130.0, 129.4, 129.3, 128.3, 128.2, 127.7, 126.9, 126.6, 126.2. HRMS (APCI): m/z calcd for $C_{18}H_{13}Cl$ [M]$^+$: 264.0700; found: 264.0694. Spectroscopic characterization in accordance with literature.[13]

Synthesis of 6-chloro-[1,1':2',1"-terphenyl]-3-carbonitrile (1)

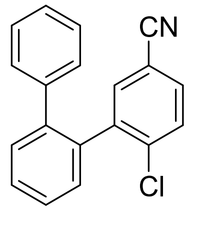

Synthesized in accordance with *general procedure II,* using [1,1'-biphenyl]-2-ylboronic acid (915 mg, 4.6 mmol), 3-bromo-4-chlorobenzonitrile (1 g, 4.6 mmol), [Pd(PPh_3)$_4$] (266 mg, 0.23 mmol), K_2CO_3 (766 mg, 5.5 mmol), toluene (23 mL) and water (3 mL). The mixture was stirred at 100 °C for 16 h. The desired product was purified by flash chromatography using a mixture of heptane/EtOAc 30:1. The pure compound was obtained as a white powder (305 mg, 0.8 mmol, yield: 23%), m.p. 98 - 100 °C. ^{1}H-NMR (300 MHz, $CDCl_3$): δ 7.52 – 7.40 (m, 6H), 7.31 – 7.29 (d, J = 7.5 Hz, 1H), 7.21 – 7.19 (m, 3H), 7.10 - 7.07 (m, 2H). ^{13}C-NMR (75 MHz, $CDCl_3$) δ 142.1, 141.5, 140.3, 138.9, 135.8, 135.6, 131.6, 130.5, 130.4, 130.4, 129.3, 129.1, 128.1, 127.3, 127.1, 118.0, 110.5. IR (ATR) υ (cm^{-1}): 3069, 3025, 2225, 1467, 1431, 1387, 1082, 1053, 1009, 903, 829, 780, 768, 743, 729, 703, 628, 611, 553, 529, 512, 486. HRMS (APCI): *m/z* calcd for $C_{19}H_{12}NCl$ [M+H]$^+$: 290.0737; found: 290.0746.

Synthesis of 2-chloro-[1,1':2',1''-terphenyl]-3-carbonitrile (3a)

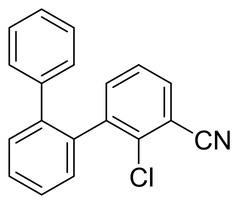

Synthesized in accordance with *general procedure II,* using [1,1'-biphenyl]-2-ylboronic acid (297 mg, 1.5 mmol), 3-bromo-2-chlorobenzonitrile (325 mg, 1.5 mmol), [Pd(PPh_3)$_4$] (87 mg, 0.075 mmol), K_2CO_3 (249 mg, 1.8 mmol), toluene (8 mL) and water (1 mL). The mixture was stirred at 100 °C for 16 h. The desired product was purified by flash chromatography using a mixture of heptane/EtOAc 40:1. The pure compound was obtained as a white powder (130 mg, 0.5 mmol, yield: 30%), m.p. 100 – 102 °C. ^{1}H-NMR (300 MHz, $CDCl_3$) δ 7.50 – 7.35 (m, 4H), 7.26 – 7.22 (m, 2H), 7.16 – 7.11 (m, 4H), 7.03 – 6.99 (m, 2H). ^{13}C-NMR (75 MHz, $CDCl_3$) δ 142.3, 141.4, 140.4, 136.5, 136.2, 136.0, 132.9, 130.5, 130.4, 129.3, 129.0, 128.1, 127.3, 127.1, 126.7, 116.4, 114.0. IR (ATR) υ (cm^{-1}): 3067, 3025, 2233, 1481, 1454, 1409, 1271, 805, 799, 776, 767, 761, 744, 733, 720, 703, 543, 436, 412. HRMS (APCI): *m/z* calcd for $C_{19}H_{12}NCl$ [M]$^+$: 289.0658; found: 289.0670.

Synthesis of 2-chloro-[1,1':2',1''-terphenyl]-4-carbonitrile (3b)

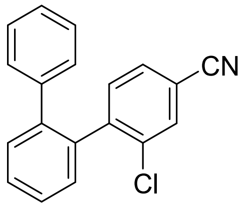

2-Bromobiphenyl (73.7 mg, 0.32 mmol), 2-Chloro-4-cyanophenylboronic acid pinacol ester (125 mg, 0.47 mmol), [$PdCl_2$(dtbpf)] (4.12 mg, 0.01 mmol), NEt_3 (0.13 mL, 0.95 mmol) and Kolliphor (2% w/w) were

dissolved in 0.5 mL of toluene and 4.5 mL of H_2O. After sonicating for 30 seconds, the mixture was stirred at 60 °C for 2 h. Later the mixture was cooled down to room temperature and filtered over a silica plug using CH_2Cl_2 as eluent. The desired product was purified by flash chromatography using a mixture of heptane/EtOAc whit gradient from 100% to 90:10. The pure compound was obtained as a colorless oil (90 mg, 0.31 mmol, yield: 98%), m.p. 100-102 °C. ^{1}H-NMR (700 MHz, $CDCl_3$) δ 7.64 (d, J = 1.6 Hz, 1H), 7.56 – 7.50 (m, 2H), 7.47 (td, J = 7.4, 1.7 Hz, 1H), 7.41 (dd, J = 7.9, 1.6 Hz, 1H), 7.35 (dd, J = 7.6, 0.7 Hz, 1H), 7.24 (m, 4H), 7.16 – 7.10 (m, 2H). ^{13}C-NMR (176 MHz, $CDCl_3$) δ 145.5, 141.0, 136.1, 132.8, 132.7, 130.2, 130.0, 129.6, 129.1, 128.9, 127.9, 127.1, 126.9, 117.4, 112.1 (2 carbon peaks are missing due to overlap). IR (ATR) υ (cm^{-1}): 3068, 3022, 2240, 1481, 1454, 1409, 1279, 804, 788, 776, 761, 760, 744, 730, 723, 703, 436, 412. HRMS (GC-MS): m/z calcd for $C_{19}H_{12}NCl$ [M]$^+$: 289.0658; found: 289.0644.

Synthesis of 6-chloro-[1,1':2',1''-terphenyl]-3,4-dicarbonitrile (3c)

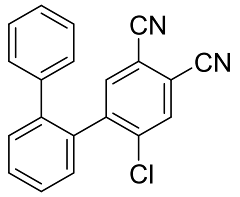

Synthesized in accordance with *general procedure II,* using [1,1'-biphenyl]-2-ylboronic acid (109 mg, 0.6 mmol), 4,5-dichlorophthalonitrile (99 mg, 1.0 mmol), [Pd(dppf)Cl_2] (26 mg, 0.04 mmol), K_2CO_3 (173 mg, 1.3 mmol), dioxane (4 mL) and H_2O (1 mL). The mixture was stirred at 40 °C for 24 h. The desired product was purified by flash chromatography using a mixture of heptane/EtOAc 80:20. The pure compound was obtained as white solid (40 mg, 0.5 mmol, yield: 25%), m.p. 106 – 109 °C. ^{1}H-NMR (600 MHz, $CDCl_3$) δ 7.68 (s, 1H), 7.48 (dd, J = 7.6, 1.3 Hz, 1H), 7.45 – 7.40 (m, 3H), 7.22 (dd, J = 7.7, 0.9 Hz, 1H), 7.18 (m, 3H), 6.99 – 6.97 (m, 2H). ^{13}C-NMR (151 MHz, $CDCl_3$) δ 146.7, 141.3, 139.6, 139.4, 136.8, 134.4, 134.2, 130.6, 129.9, 129.8, 129.11, 128.37, 127.6, 127.5, 115.2, 114.6, 114.0, 113.3. IR (ATR) υ (cm^{-1}): 2206, 2089, 2055, 1956, 611, 557, 473, 454, 431, 409. HRMS (APCI): *m/z* calcd for $C_{20}H_{11}ClN_2$ [M+Na]$^+$: 337.0703; found: 337.0501.

Synthesis of methyl 6-chloro-[1,1':2',1''-terphenyl]-4-carboxylate (3d)

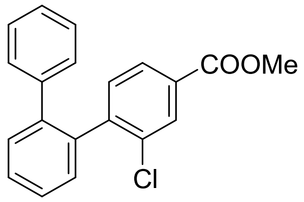

2-Bromobiphenyl (90.6 mg, 0.39 mmol), 2-Chloro-5-(methoxycarbonyl)phenylboronic acid (125 mg, 0.58 mmol), [PdCl2(dtbpf)] (5 mg, 0.01 mmol), NEt_3 (0.16 mL, 1.8 mmol) and Kolliphor (2% w/w) were dissolved in 0.5 mL of toluene and 4.5 mL of H_2O. After sonicating for 30 seconds, the mixture was stirred

at 60 °C for 2 h. Later the mixture was cooled down to room temperature and filtered over a silica plug using CH_2Cl_2 as eluent. The desired product was purified by flash chromatography using a mixture of heptane/EtOAc whit gradient from 100% to 90:10. The pure compound was obtained as a colorless Soil (115 mg, 0.36 mmol, yield: 91%), m.p. 135 – 138 °C. ^{1}H-NMR (600 MHz, $CDCl_3$) δ 7.92 (d, J = 2.1 Hz, 1H), 7.86 (dd, J = 8.3, 2.1 Hz, 1H), 7.52 – 7.43 (m, 3H), 7.36 (t, J = 8.0 Hz, 2H), 7.21 – 7.16 (m, 5H), 3.88 (s, 3H). ^{13}C-NMR (151 MHz, $CDCl_3$) δ 166.2, 141.5, 140.8, 140.8, 138.5, 137.1, 133.3, 130.6, 130.2, 129.5, 129.4, 129.4, 128.6, 128.4, 127.9, 127.2, 126.8, 52.3. IR (ATR) υ (cm^{-1}): 3070, 3020, 2947, 1730, 1701, 1428, 1397, 1277, 1262, 1240, 1234, 1107, 1079, 1023, 923, 850, 771, 758, 746, 699, 691, 550, 529. HRMS (GC-MS): m/z calcd for $C_{20}H_{15}O_2Cl$ [*M*]$^+$: 322.0755; found: 322.0748.

Synthesis of methyl 6-chloro-[1,1':2',1''-terphenyl]-3-carboxylate (3e)

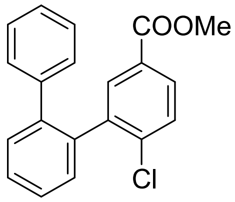

Synthesized in accordance with *general procedure II,* using [1,1'-biphenyl]-2-ylboronic acid (198 mg, 1.0 mmol), methyl 3-bromo-4-chlorobenzoate (250 mg, 1.0 mmol), [$Pd(PPh_3)_4$] (58 mg, 0.05 mmol), K_2CO_3 (276 mg, 2 mmol), toluene (5 mL) and water (1 mL). The mixture was stirred at 110 °C for 24 h. The desired product was purified by flash chromatography using a mixture of heptane/EtOAc 30:1. The pure compound was obtained as a white powder (102 mg, 0.3 mmol, yield: 32%), m.p. 134 – 136 °C. ^{1}H-NMR (300 MHz, $CDCl_3$) δ 7.90 (d, J = 2.2 Hz, 1H), 7.86 (dd, J = 8.3, 2.3 Hz, 1H), 7.55 – 7.43 (m, 3H), 7.39 – 7.33 (m, 2H), 7.21 – 7.13 (m, 5H), 3.89 (s, 3H). ^{13}C-NMR (75 MHz, $CDCl_3$) δ 166.3, 141.6, 140.9, 138.6, 137.2, 133.3, 130.6, 130.3, 129.6, 129.5, 129.4, 128.7, 128.4, 127.9, 127.2, 126.9, 52.4 (1 carbon peak is missing due to overlap). IR (ATR) υ (cm^{-1}): 3071, 3025, 2952, 1722, 1700, 1428, 1396, 1276, 1266, 1241, 1231, 1107, 1079, 1022, 909, 852, 770, 757, 746, 699, 691, 552, 529. HRMS (APCI): *m/z* calcd for $C_{20}H_{15}O_2Cl$ [*M*]$^+$: 322.0755; found: 322.0754.

Synthesis of 6-chloro-[1,1':2',1''-terphenyl]-3,4''-dicarbonitrile (3f)

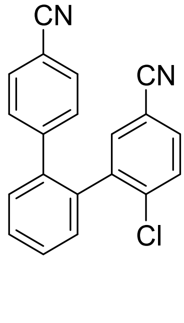

2'-bromo-6-chloro-[1,1'-biphenyl]-3-carbonitrile (282 mg, 1 mmol), (4-cyanophenyl)boronic acid (176.4 mg, 1.2 mmol), [PdCl2(dtbpf)] (13 mg, 0.02 mmol), NEt_3 (0.418 mL, 3 mmol) and Kolliphor (2% w/w) were dissolved in 0.5 mL of toluene and 4.5 mL of H_2O. After sonicating for 30 seconds, the mixture was stirred at 60 °C for 2 h. Later the mixture was cooled down to room temperature and filtered over a silica plug using CH_2Cl_2 as eluent. The desired product was purified by flash chromatography using a mixture of heptane/EtOAc whit gradient from 100% to 90:10. The resulting material was further recrystallised from hexane to give the pure compound as an off-white powder (234 mg, 0.743 mmol, 74% yield), m.p. 142 – 144 °C. ^{1}H NMR (600 MHz, $CDCl_3$): δ 7.59 – 7.48 (m, 5H), 7.47 – 7.41 (m, 3H), 7.35 – 7.30 (m, 1H), 7.23 – 7.19 (m, 2H). ^{13}C NMR (151 MHz, $CDCl_3$) δ 145.3, 141.3, 139.6, 138.8, 135.8, 135.3, 132.2, 132.0, 130.8, 130.8, 130.1, 130.0, 129.5, 128.6, 118.8, 117.8, 111.1, 111.0. IR (ATR) υ (cm^{-1}): 3067, 2959, 2924, 2851, 2226, 1721, 1609, 1458, 1261, 1099, 826, 764, 613, 571. HRMS (ESI): m/z calcd for $C_{20}H_{11}ClN_2$ [M+Na]$^+$: 337.0703; found: 337.0501.

Synthesis of methyl 2''-chloro-5''-cyano-[1,1':2',1''-terphenyl]-4-carboxylate (3g)

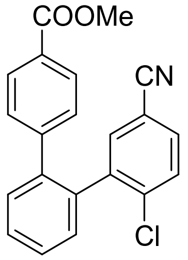

Synthesized in accordance with *general procedure II,* using (2-chloro-5-cyanophenyl)boronic acid (272 mg, 1.5 mmol), methyl 2'-bromo-[1,1'-biphenyl]-4-carboxylate (291 mg, 1.0 mmol), [Pd(dppf)Cl_2] (37 mg, 0.05 mmol), K_2CO_3 (276 mg, 2.0 mmol), dioxane (5 mL) and water (1 mL). The mixture was stirred at 66 °C for 36 h. The desired product was purified by flash chromatography using a mixture of heptane/EtOAc whit gradient from 100% to 5:1. The resulting material was washed with petroleum ether and further recrystallised from hexane to give the pure compound as an off-white powder (141 mg, 0.4 mmol, yield: 40%), m.p. 154 – 156 °C. ^{1}H-NMR (300 MHz, $CDCl_3$) δ 7.89 (d, J = 8.2 Hz, 2H), 7.57 – 7.40 (m, 6H), 7.32 (d, J = 7.8 Hz, 1H), 7.16 (d, J = 8.2 Hz, 2H), 3.89 (s, 3H). ^{13}C-NMR (75 MHz, $CDCl_3$) δ 166.9, 145.1, 141.7, 140.5, 138.9, 135.8, 135.4, 132.0, 130.7, 130.6, 130.2, 129.5, 129.4, 129.3, 128.9, 128.1, 117.9, 110.8, 52.3. IR (ATR) υ (cm^{-1}): 3067, 2955, 2230, 1713, 1605, 1470, 1435, 1393, 1312, 1281, 1192, 1180, 1119, 1103, 1080, 1049, 1022, 907, 860, 833, 748, 729, 706, 613. HRMS (APCI): *m/z* calcd for $C_{21}H_{14}NO_2Cl$ [M+Na]$^+$: 370.0611; found: 370.0615.

Synthesis of methyl 2''-chloro-5''-cyano-[1,1':2',1''-terphenyl]-4'-carboxylate (3j)

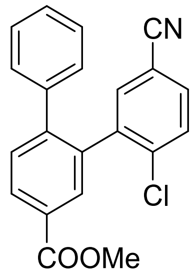

Synthesized in accordance with *general procedure II,* using (2-chloro-5-cyanophenyl)boronic acid (63 mg, 0.4 mmol), methyl 2-bromo-[1,1'-biphenyl]-4-carboxylate (66 mg, 0.2 mmol), [Pd(dtbpf)Cl_2] (3 mg, 0.004 mmol), NEt_3 (141 mg, 1.4 mmol) and Kolliphor (2% w/w) in 0.5 mL of toluene and 4.5 mL of H_2O. After sonicating for 30 seconds, the mixture was at 60 °C for 2 h. Later the mixture was cooled down to room temperature and filtered over a silica plug using CH_2Cl_2 as eluent. The desired product was purified by flash chromatography using a mixture of petroleum ether/ethyl acetate with gradient from 100% to 15:1. The pure compound was obtained as a white powder (56 mg, 0.2 mmol, 59% yield), m.p. 196 - 200 °C. ^{1}H-NMR (600 MHz, $CDCl_3$) δ 8.17 (dd, J = 8.1, 1.6 Hz, 1H), 7.99 (d, J = 1.4 Hz, 1H), 7.56 (d, J = 8.1 Hz, 1H), 7.49 – 7.43 (m, 3H), 7.26 – 7.22 (m, 3H), 7.11 – 7.08 (m, 2H) 3.95 (s, 3H). ^{13}C-NMR (151 MHz, $CDCl_3$) δ 166.5, 146.0, 141.3, 139.4, 138.9, 136.0, 135.5, 132.1, 131.8, 130.7, 130.7, 130.2, 129.3, 129.2, 128.3, 127.9, 117.9, 110.8, 52.5. IR (ATR) υ (cm^{-1}): 3059, 2980, 2970, 2234, 1717, 1472, 1429, 1387, 1310, 1277, 1260, 1240, 1109, 1076, 1042, 1007, 833, 781, 760, 698, 621. HRMS (ESI): *m/z* calcd for $C_{21}H_{14}NO_2Cl$ [M+H]$^+$: 348.0791; found: 348.0798.

Synthesis of 4''-(tert-butyl)-6-chloro-[1,1':2',1''-terphenyl]-3-carbonitrile (3k)

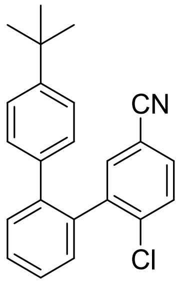

Synthesized in accordance with *general procedure II,* using (2-chloro-5-cyanophenyl)boronic acid (190 mg, 1.1 mmol), 2-bromo-4'-(tert-butyl)-1,1'-biphenyl (202 mg, 0.7 mmol), [Pd(dppf)Cl_2] (77 mg, 0.11 mmol), K_2CO_3 (193 mg, 1.4 mmol), dioxane (5 mL) and water (1 mL). The mixture was stirred at 101 °C for 16 h. The desired product was purified by flash chromatography using a mixture of heptane/EtOAc with gradient from 100% to 25:1. The resulting material purified by heating to 45 °C under a high vacuum (the side product sublimes). The pure compound was obtained as a white powder (163 mg, 0.5 mmol, yield: 67%), m.p. 148 – 149 °C. ^{1}H-NMR (300 MHz, $CDCl_3$) δ 7.51 – 7.36 (m, 6H), 7.30 – 7.20 (m, 3H), 7.0 (d, J = 8.2 Hz, 2H), 1.27 (s, 9H). ^{13}C-NMR (75 MHz, $CDCl_3$) δ 150.1, 142.3, 141.4, 139.1, 137.3,

135.8, 135.7, 131.6, 131.5, 130.6, 130.5, 129.1, 129.0, 127.1, 125.1, 110.4, 34.6, 31.4 (1 carbon peak is missing due to overlap). IR (ATR) υ (cm^{-1}): 3058, 3026, 2964, 2233, 1461, 1444, 1387, 1270, 1110, 1079, 1047, 1024, 905, 838, 826, 772, 767, 743, 642, 624, 613, 583, 563, 543, 49. HRMS (APCI): *m/z* calcd for $C_{23}H_{20}ClN$ [*M*]$^+$: 345.1284; found: 345.1287.

Synthesis of 6-chloro-4''-methoxy-[1,1':2',1''-terphenyl]-3-carbonitrile (3l)

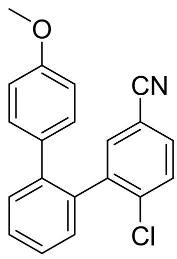

2'-bromo-6-chloro-[1,1'-biphenyl]-3-carbonitrile (282 mg, 1.0 mmol), (4-methoxyphenyl)boronic acid (182.4 mg, 1.2 mmol), [$PdCl_2$(dtbpf)] (13 mg, 0.02 mmol), NEt_3 (0.418 mL, 3.0 mmol) and Kolliphor (2% w/w) were dissolved in 0.5 mL of toluene and 4.5 mL of H_2O. After sonicating for 30 seconds, the mixture was stirred at 60 °C for 2 h. Later the mixture was cooled down to room temperature and filtered over a silica plug using CH_2Cl_2 as eluent. The desired product was purified by flash chromatography using a mixture of heptane/EtOAc whit gradient from 100% to 90:10. The pure compound was obtained as white solid (224 mg, 0.70 mmol, 70% yield), m.p. 142 – 144 °C. ^{1}H NMR (600 MHz, $CDCl_3$) δ 7.53 – 7.36 (m, 6H), 7.32 – 7.24 (m, 1H), 7.03 – 7.02 (m, 1H), 7.01 – 6.99 (m, 1H), 6.77 – 6.75 (m, 1H), 6.75 – 6.73 (m, 1H), 3.77 (s, 3H). ^{13}C NMR (151 MHz, $CDCl_3$) δ 142.3, 141.0, 138.9, 135.7, 135.6, 132.7, 131.5, 130.5, 130.4, 130.3, 129.0, 126.9, 118.0, 113.5, 110.5, 55.16 (2 carbon peaks are missing due to overlap). IR (ATR) υ (cm^{-1}): 2101, 2001, 642, 593, 527, 503, 460. HRMS (ESI): m/z calcd for $C_{20}H_{14}ClNO$ [*M*+H]$^+$: 320.0764; found: 320.0835.

Synthesis of 6-chloro-3''-methoxy-[1,1':2',1''-terphenyl]-3-carbonitrile (3m)

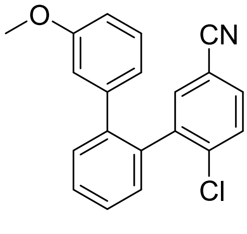

Synthesized in accordance with *general procedure II,* using (2-chloro-5-cyanophenyl)boronic acid (341 mg, 1.9 mmol), 2-bromo-3'-methoxy-1,1'-biphenyl (165 mg, 0.63 mmol), [$Pd(OAc)_2$] (35 mg, 0.16 mmol), K_3PO_4 (532 mg, 2.5 mmol), SPhos (0.13 mmol, 51.5 mg), THF (4 mL) and H_2O (1 mL). The mixture was stirred at 66 °C for 36 h. The desired product was purified by flash chromatography using a mixture of heptane/EtOAc whit gradient from 100% to 90:10. The pure compound was obtained as dense colourless oil (100 mg, 0.31 mmol, yield: 36%). ^{1}H-NMR (600 MHz, $CDCl_3$) δ 7.54 – 7.48 (m, 2H), 7.47 – 7.43 (m,

3H), 7.42 (s, 1H), 7.33 – 7.28 (m, 1H), 7.13 (t, *J* = 7.9 Hz, 1H), 6.76 (dd, *J* = 8.3, 1.0 Hz, 1H), 6.70 (d, *J* = 7.6, Hz, 1H), 6.66 (d, *J* = 1.6 Hz, 1H), 3.68 (s, 3H). ^{13}C-NMR (151 MHz, $CDCl_3$) δ 159.1, 142.1, 141.6, 141.1, 138.8, 135.6, 135.3, 131.5, 130.4, 130.3, 130.1, 129.0, 128.9, 127.2, 121.6, 117.8, 114.6, 112.8, 110.4, 54.9. IR (ATR) υ (cm^{-1}): 2101, 2001, 642, 593, 527, 503, 460. HRMS (ESI): *m/z* calcd for $C_{20}H_{14}ClNO$ [*M*+Na]$^+$: 320.0656; found: 320.0835.

Synthesis of 6-chloro-2'',4''-dimethoxy-[1,1':2',1''-terphenyl]-3-carbonitrile (3n)

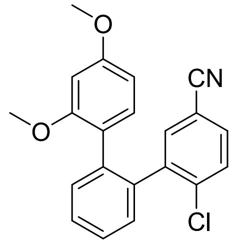

Synthesized in accordance with *general procedure II,* using 2,4-dimethoxyphenylboronic acid (280 mg, 1.5 mmol), 2'-bromo-6-chloro-[1,1'-biphenyl]-3-carbonitrile (300 mg, 1.03 mmol), [Pd(dppf)Cl_2] (28 mg, 0.039 mmol), K_2CO_3 (216 mg, 1.6 mmol), dioxane (5 mL) and water (1 mL). Refluxed overnight. Purified by silica gel chromatography with eluent heptane/EtOAc 20:1. The pure compound was obtained as a white powder (180 mg, 66% yield). m.p. 154 – 156 °C; ^{1}H NMR (600 MHz, $CDCl_3$): δ 7.50 – 7.15 (m, 7H), 6.97 (d, *J* = 8.3 Hz, 1H), 6.35 (d, *J* = 8.3 Hz, 1H), 6.15 (d, *J* = 2.2 Hz, 1H), 3.69 (s, 3H), 3.40 (s, 3H); ^{13}C NMR (151 MHz, $CDCl_3$) δ 160.7, 156.7, 142.3, 138.9, 138.0, 135.3, 131.9, 131.3, 131.2, 130.2, 129.8, 128.8, 127.1, 122.0, 118.2, 109.7, 104.3, 98.1, 55.4, 54.8 (1 carbon peak is missing due to overlap); m.p. 103 - 104 °C; IR (ATR) υ (cm^{-1}): 3065, 2995, 2967, 2942, 2231, 1613, 1511, 1456, 1437, 1298, 1207, 1159, 1135, 1053, 1027, 1021, 899, 840, 823, 800, 763, 636, 625, 612, 576, 561, 508, 482; HRMS (ESI): m/z calcd for $C_{21}H_{16}O_2NCl$ [*M*]$^+$: 349.0864; found: 349.0862.

Synthesis of 6-chloro-5'-methoxy-[1,1':2',1''-terphenyl]-3-carbonitrile (3o)

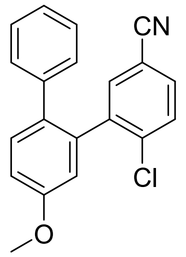

Synthesized in accordance with *general procedure II,* using (2-chloro-5-cyanophenyl)boronic acid (1.6 mmol, 295 mg), 2-bromo-4-methoxy-1,1'-biphenyl (143 mg, 0.5 mmol), [Pd$(OAc)_2$] (31 mg, 0.14 mmol), K_3PO_4 (461 mg, 2.2 mmol), SPhos (45 mg, 0.1 mmol), THF (4 mL) and H_2O (1 mL). The mixture was stirred at 66 °C for 16 h. The desired product was purified by flash chromatography using a mixture of heptane/EtOAc whit gradient from 100% to 90:10. The pure compound was obtained as white solid (116

mg, 0.36 mmol, yield: 67%), m.p. 140 – 143 °C. ^{1}H-NMR (600 MHz, $CDCl_3$) δ 7.44 – 7.40 (m, 4H), 7.18 (m, 3H), 7.05 (m, 3H), 6.83 (d, J = 2.7 Hz, 1H), 3.87 (s, 3H). ^{13}C-NMR (151 MHz, $CDCl_3$) δ 158.6, 142.0, 140.0, 138.8, 136.7, 135.4, 134.0, 131.6, 131.5, 130.5, 129.3, 128.0, 126.7, 117.9, 115.7, 114.6, 110.4, 55.5. IR (ATR) υ (cm^{-1}): 2231, 1605, 1482, 1465, 1442, 1269, 1216, 1075, 1049, 1029, 821, 769, 733, 700. HRMS (ESI): *m/z* calcd for $C_{21}H_{13}ClN_2O$ [*M*+Na]$^+$: 342.0656; found: 342.0640.

Synthesis of 6-chloro-4'',5'-bis(dimethylamino)-[1,1':2',1''-terphenyl]-3-carbonitrile (3p)

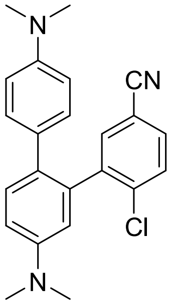

Synthesized in accordance with *general procedure II,* using (2-chloro-5-cyanophenyl)boronic acid (120 mg, 0.66 mmol), 2-bromo-N4,N4,N4',N4'-tetramethyl-[1,1'-biphenyl]-4,4'-diamine (70 mg, 0.22 mmol), $Pd(OAc)_2$ (10 mg, 0.05 mmol), K_3PO_4 (187 mg, 0.88 mmol), Sphos (23 mg, 0.06 mmol), THF (2 mL) and H_2O (0.5 mL). The mixture was stirred at 66 °C for 48 h. The desired product was purified by flash chromatography using a mixture of heptane/EtOAc whit gradient from 100% to 90:10. The pure compound was obtained as dense colourless oil (33 mg, 0.09 mmol, yield: 41%). ^{1}H-NMR (600 MHz, $CDCl_3$) δ 7.42 (d, J = 1.9 Hz, 1H), 7.38 – 7.33 (m, 2H), 7.25 (d, J = 8.6 Hz, 1 H), 6.84 – 6.77 (m, 3H), 6.49 – 6.45 (m, 3H), 2.92 (s, 6H), 2.82 (s, 6H). ^{13}C-NMR (151 MHz, $CDCl_3$) δ 148.9, 148.8, 143.4, 139.0, 136.1, 135.7, 131.2, 130.9, 130.4, 129.9, 128.6, 118.3, 114.0, 113.1, 112.1, 110.3, 40.6, 40.5 (1 carbon peak is missing due to overlap). IR (ATR) υ (cm^{-1}): 3487, 2210, 1546, 1129, 989, 855, 831, 567. HRMS (ESI): *m/z* calcd for $C_{23}H_{22}ClN_3$ [*M*+H]$^+$: 376.1575; found: 376.1568.

Synthesis of 6,6''-dichloro-[1,1':2',1''-terphenyl]-3,3''-dicarbonitrile (3q)

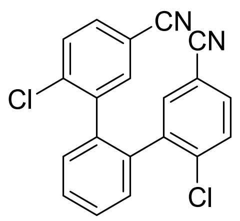

1,2-Diiodobenzene (330 mg, 1 mmol), (2-chloro-5-cyanophenyl)boronic acid (544 mg, 3.0 mmol), [PdCl2(dtbpf)] (13 mg, 0.02 mmol), NEt_3 (0.418 mL, 3.0 mmol) and Kolliphor (2% w/w) were dissolved in 0.5 mL of toluene and 4.5 mL of H_2O. After sonicating for 30 seconds, the mixture was stirred at 60 °C

for 2 h. Later the mixture was cooled down to room temperature and filtered over a silica plug using CH_2Cl_2 as eluent. The desired product was further recrystallised from hexane to give the pure compound as a light-yellow powder (329 mg, 0.942 mmol, 94% yield), m.p. 185 – 188 °C. ^{1}H NMR (600 MHz, $CDCl_3$): δ 7.56 – 7.54 (m, 2H), 7.50 – 7.40 (m, 6H), 7.40 – 7.32 (m, 2H). ^{13}C NMR (151 MHz, $CDCl_3$) δ 145.2, 141.2, 135.7, 135.2, 132.2, 131.9, 130.0, 129.9, 129.4, 118.6. IR (ATR) υ (cm^{-1}): 3067, 2959, 2924, 2851, 2226, 1721, 1609, 1458, 1261, 1099, 826, 764, 613, 571. HRMS (GC-MS): m/z [*M*]$^+$ calcd for ($C_{20}H_{10}Cl_2N_2$): 348.0221; found: 348.0208.

Synthesis of 4'-bromo-2,4,6-trimethyl-1,1'-biphenyl

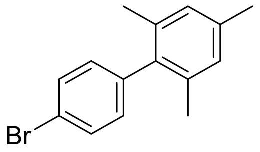

2-Iodomesitylene (1230 mg, 5 mmol), 4-Bromobenzeneboronic acid (1205 mg, 6 mmol), [$PdCl_2$(dtbpf)] (65 mg, 0.1 mmol), NEt_3 (2.1 mL, 15.0 mmol) and Kolliphor (2% w/w) were dissolved in 1 mL of toluene and 9 mL of H_2O. After sonicating for 30 seconds, the mixture was stirred at 60 °C for 2 h. Later, the mixture was cooled down to room temperature and filtered over a silica plug using CH_2Cl_2 as eluent. The desired product was purified by flash chromatography using heptane as eluent. The pure compound was obtained as white solid (1149 mg, 4.2 mmol, 84% yield). ^{1}H NMR (600 MHz, $CDCl_3$) δ 7.58 (dd, J = 7.1, 1.4 Hz, 2H), 7.05 (dd, J = 7.1, 1.4 Hz, 2H), 6.98 (s, 2H), 2.37 (s, 3H), 2.03 (s, 6H). ^{13}C NMR (151 MHz, $CDCl_3$) δ 140.0, 137.8, 137.0, 135.9, 131.7, 131.1, 128.2, 120.7, 21.0, 20.8. HRMS (GC-MS): m/z [*M*+1]$^+$ calcd for ($C_{15}H_{15}Br$): 274.0357; found 274.0347. Spectroscopic characterization in accordance with literature.[14]

Synthesis of N-(2',4',6'-trimethyl-[1,1'-biphenyl]-4-yl)-[1,1'-biphenyl]-2-amine

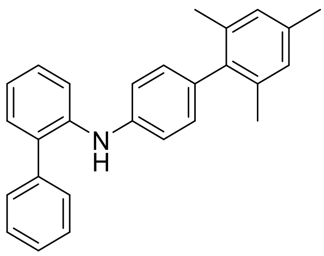

In the glovebox, a 25 mL Schlenk tube containing a stir bar was charged with $Pd_2(dba)_3$ (2.2 mg, 0.002 mmol), *rac*-BINAP (4.4 mg, 0.007 mmol), NaOtBu (170 mg, 1.77 mmol), 2-aminobiphenyl (200 mg, 1.2 mmol), 4'-bromo-2,4,6-trimethyl-1,1'-biphenyl (308 mg, 1.1 mmol) and toluene (5 mL). The screw-cap of the Schlenk tube was closed and the mixture was stirred at 110 °C for 20 h (inside of the glove-box). The mixture was poured onto 40 mL of H_2O and extracted with CH_2Cl_2 (4x20 mL). After drying over $MgSO^4$, the solvent and all volatiles were evaporated *under vacuum*. The residue was sonicated with n-heptane leading to a brownish solution. The desired product was purified by flash chromatography using

heptane/CH_2Cl_2 gradient from 100% to 90:10. The pure compound was obtained as colorless oil (300 mg, 0.825 mmol, 87% yield). ^{1}H NMR (600 MHz, $CDCl_3$) δ 7.48 (m, 5H), 7.38 (t, J = 7.1 Hz, 1H), 7.31 – 7.27 (m, 2H), 7.10 (d, J = 8.4 Hz, 2H), 7.01 (m, 3H), 6.95 (s, 2H), 5.71 (s, 1H), 2.34 (s, 3H), 2.06 (s, 6H). ^{13}C NMR (151 MHz, $CDCl_3$) δ 141.5, 140.5, 139.1, 138.7, 136.4, 136.4, 134.0, 131.05, 130.9, 130.2, 29.4, 128.9, 128.2, 128.0, 127.4, 120.7, 118.6, 116.6, 21.0, 20.8. IR (ATR) υ (cm^{-1}): 3487, 2210, 1546, 1129, 989, 855, 831, 567. HRMS (Maldi-TOF): m/z calcd for $C_{27}H_{25}N$ [M+H]$^+$: 363.1987 found: 364.2049.

Synthesis of 4-chloro-3-(5-(2',4',6'-trimethyl-[1,1'-biphenyl]-4-yl)dibenzo[c,e] [1,2]azaborinin-6(5H)-yl)benzonitrile (3r)

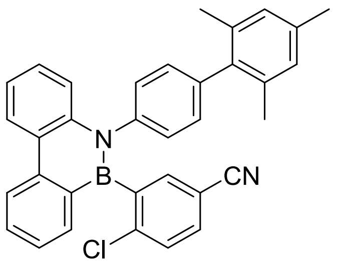

In the glovebox, a 25 mL Schlenk tube containing a stir bar was charged with N-(2',4',6'-trimethyl-[1,1'-biphenyl]-4-yl)-[1,1'-biphenyl]-2-amine (55 mg, 0.15 mmol) and dry o-DCB (2.5 mL). BBr_3 (neat, 0.042 mL) was added to the colorless solution. The Schlenk tube was closed with a glass stopper (with Glindemann ring) and the yellow solution was stirred at 130 °C for 18 h (outside of the glovebox). After allowing the blue solution to reach room temperature, all volatiles were removed *in vacuo* (through Schlenk line, with external trap). In the glove-box, 1 mL THF and (2-chloro-5-cyanophenyl)magnesium bromide (107 mg, 0.45 mmol in dry THF previously prepared by reaction between 3-bromo-4-chlorobenzonitrile (97 mg, 0.45 mmol) and *i*-propylmagnesium chloride (2.5 M in ether, 44.7 mg, 0.45 mmol) in dry THF (2 mL)) were cannulated to the mixture and the resulting yellow solution was stirred at r.t. for 18 h (inside the glove-box). The yellow solution was quenched with MeOH (5 mL) at 0 °C and evaporated *in vacuo*. After adding 1 M HCl (15 mL), the mixture was extracted with CH_2Cl_2 (4 x 15 mL). The combined organic layers were dried over Na_2SO_4 and evaporated *in vacuo*. The desired product was purified by flash chromatography using a mixture of heptane/CH_2Cl_2 whit gradient from 100% to 90:10. The pure compound was obtained as pale yellow solid (53 mg, 0.104 mmol, 70% yield), m.p. 235 – 237 °C. ^{1}H NMR (600 MHz, $CDCl_3$) δ 8.62 – 8.59 (m, 2H), 7.84 – 7.72 (m, 1H), 7.51 – 7.49 (m, 1H), 7.42 (t, J = 7.4 Hz, 1H), 7.36 – 7.34 (m, 4H), 7.30 – 7.24 (m, 1H), 7.18 (dd, J = 8.0, 2.2 Hz, 1H), 7.17 – 7.14 (m, 3H), 7.04 (m, 1H), 6.96 (s, 1H), 6.91 (d, J = 1.0 Hz, 1H), 2.33 (s, 3H), 2.13 (s, 3H), 1.67 (s, 3H). ^{13}C NMR (151 MHz, $CDCl_3$) δ 141.9, 141.8, 141.2, 140.6, 138.0, 137.0, 136.9, 136.2, 136.0, 135.5, 132.0, 131.8, 130.5, 130.1, 129.1, 128.8, 128.1, 128.1, 127.9, 124.4, 124.2, 122.5, 122.3, 118.9, 118.4, 109.7, 21.1, 20.8, 20.1. IR (ATR) υ (cm^{-1}): 3062, 2960, 2920, 2856, 2220, 1604, 1578, 1551, 1482, 1443, 1425, 1373, 1356, 1342, 1319, 1301, 1295, 1271, 1240, 1199, 1166, 1105, 1083, 1030, 993, 961, 922, 849, 802, 786, 752, 725, 659, 623, 594, 564, 524, 434. HRMS (Maldi-TOF): m/z calcd for $C_{34}H_{26}BClN_2$ [M+H]$^+$: 508.1878 found: 508.1877.

Synthesis of 6-chloro-2'-(1H-pyrrol-1-yl)-[1,1'-biphenyl]-3-carbonitrile (5a)

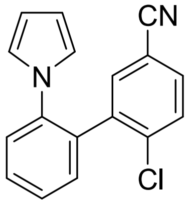

Synthesized in accordance with *general procedure II,* using (2-chloro-5-cyanophenyl)boronic acid (272 mg, 1.7 mmol), 1-(2-bromophenyl)-1H-pyrrole (244 mg, 1.1 mmol), $[Pd(PPh_3)_2Cl_2]$ (77 mg, 0.11 mmol), K_2CO_3 (759 mg, 5.5 mmol), DME (6 mL) and water (2 mL). The mixture was stirred at 110 °C for 24 h. The desired product was purified by flash chromatography using a mixture of heptane/EtOAc with gradient from 100% to 30:1. The pure compound was obtained as a white powder (130 mg, 0.5 mmol, yield: 42%), m.p. 104 – 107 °C. ^{1}H-NMR (300 MHz, $CDCl_3$) δ 7.56 – 7.49 (m, 3H), 7.47 – 7.41 (m, 3H), 7.36 – 7.34 (m, 1H), 6.54 – 6.53 (m, 2H), 6.12 – 6.10 (m, 2H). ^{13}C-NMR (75 MHz, $CDCl_3$) δ 139.8, 139.6, 139.2, 134.5, 132.5, 132.3, 131.4, 130.7, 130.1, 127.1, 126.2, 121.9, 117.8, 111.1, 109.7. IR (ATR) υ (cm^{-1}): 3067, 2230, 1593, 1578, 1501, 1466, 1443, 1393, 1331, 1319, 1069, 1015, 922, 837, 760, 733, 613, 498. HRMS (APCI): *m/z* calcd for $C_{17}H_{12}ClN_2$ $[M+H]^+$: 279.0689; found: 279.0698.

Synthesis of 6-chloro-2'-(thiophen-3-yl)-[1,1'-biphenyl]-3-carbonitrile (5b)

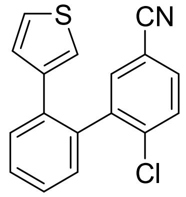

Synthesized in accordance with *general procedure II,* using (2-chloro-5-cyanophenyl)boronic acid (283 mg, 1.6 mmol), 3-(2-bromophenyl)thiophene (249 mg, 1.1 mmol), $[Pd(dppf)Cl_2]$ (39 mg, 0.052 mmol), K_2CO_3 (287 mg, 2.1 mmol), dioxane (8 mL) and water (2 mL). The mixture was stirred at 101 °C for 16 h. The desired product was purified by flash chromatography using a mixture heptane/EtOAc with gradient from 100% to 25:1, followed by further recrystallization from hexane to give the pure compound as an off-white powder (136 mg, 0.5 mmol, yield: 44%), m.p. 108 – 110 °C. ^{1}H-NMR (300 MHz, $CDCl_3$): δ 7.48 – 7.38 (m, 5H), 7.34 (ddd, J = 7.5, 7.2, 1.8 Hz, 1H), 7.21 – 7.18 (m, 1H), 7.08 (dd, J = 5.0, 3.0 Hz, 1H), 6.82 (dd, J = 3.0, 1.3 Hz, 1H), 6.69 (dd, J = 5.0, 1.3 Hz, 1H). ^{13}C-NMR (75 MHz, $CDCl_3$) δ 142.3, 140.8, 139.1, 136.1, 135.7, 135.3, 131.9, 130.7, 130.4, 130.0, 129.2, 128.3, 127.4, 125.4, 123.3, 118.0, 110.8. IR (ATR) υ (cm^{-1}): 3061, 2234, 1466, 1461, 1438, 1390, 1194, 1077, 1022, 912, 828, 824, 793, 770, 765, 753, 715, 647, 624, 612, 497. HRMS (APCI): *m/z* calcd for $C_{17}H_{11}NSCl$ $[M+H]^+$: 296.0301; found: 296.0313.

Synthesis of 6-chloro-2'-(pyridin-3-yl)-[1,1'-biphenyl]-3-carbonitrile (5c)

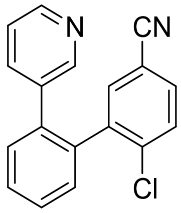

2'-bromo-6-chloro-[1,1'-biphenyl]-3-carbonitrile (282 mg, 1 mmol), pyridin-3-ylboronic acid (370 mg, 0,9 mmol), [PdCl$_2$(dtbpf)] (147.6 mg, 1.2 mmol), NEt$_3$ (0.418 mL, 3 mmol) and Kolliphor (2% w/w) were dissolved in 0.5 mL of toluene and 4.5 mL of H_2O. After sonicating for 30 seconds, the mixture was stirred at 60 °C for 2 h. Later the mixture was cooled down to room temperature and filtered over a silica plug using CH_2Cl_2 as eluent. The desired product was purified by flash chromatography using a mixture of heptane/EtOAc whit gradient from 100% to 90:10. The pure compound was obtained as a colourless oil (421 mg, 1.45 mmol, yield: 46%). ^{1}H-NMR (600 MHz, $CDCl_3$) δ 8.46 (dd, J = 4.8, 1.5 Hz, 1H), 8.38 (d, J = 2.1 Hz, 1H), 7.60 – 7.47 (m, 5H), 7.44 – 7.39 (m, 2H), 7.33 (dd, J = 7.5, 1.1 Hz, 1H), 7.18 – 7.13 (m, 1H). ^{13}C-NMR (151 MHz, $CDCl_3$) δ 149.9, 148.3, 141.3, 138.7, 137.7, 136.3 136.1, 136.0, 135.3, 132.1, 130.7, 130.6, 130.3, 129.4, 128.3, 122.9, 117.7, 110.1. IR (ATR) υ (cm^{-1}): 2101, 2001, 642, 593, 527, 503, 460. HRMS (GC-MS): m/z calcd for $C_{18}H_{11}ClN_2$ [M+Na]$^+$ 290.0611; found:290.0600.

Synthesis of 6-chloro-2'-(naphthalen-2-yl)-[1,1'-biphenyl]-3-carbonitrile (7a)

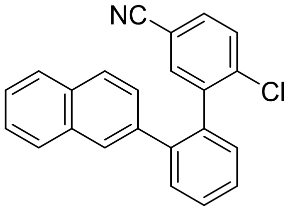

2-bromonapthalene (207 mg, 1 mmol) was dissolved in dry THF (5 mL) and cooled down to –78 °C. BuLi 2.5 M in hexane (0.6 mL, 1.5 mmol) was added dropwise and the solution was left stirring under argon atmosphere for 1 h. Then, trimethyl borate (0.167 mL, 1.5 mmol) was added dropwise and the mixture was allowed to reach room temperature. The mixture was quenched with MeOH (5 mL), washed twice with water (2 times 20 mL) and brine (10 mL). The crude was dried under vacuum and directly used in the following step. 2'-bromo-6-chloro-[1,1'-biphenyl]-3-carbonitrile (263 mg, 0.9 mmol), dimethyl naphthalen-2-ylboronate (216 mg, 1.08 mmol), [PdCl$_2$(dtbpf)] (12 mg, 0.018 mmol), NEt$_3$ (0.376 mL, 2.7 mmol) and Kolliphor (2% w/w) were dissolved in 0.5 mL of toluene and 4.5 mL of H_2O. After sonicating for 30 seconds, the mixture was stirred at 60 °C for 2 h. Later, the mixture was cooled down to room temperature and filtered over a silica plug using CH_2Cl_2 as eluent. The desired product was purified by flash chromatography using a mixture of heptane/EtOAc whit gradient from 100% to 90:10. The pure compound was obtained as white solid (256 mg, 0.753 mmol, 84% yield), m.p. 200 – 202 °C. ^{1}H NMR (600 MHz, $CDCl_3$): δ 7.66 (dd, J = 6.1, 3.4 Hz, 1H), 7.61 (dd, J = 6.0, 3.4 Hz, 1H), 7.55 (d, J = 8.5 Hz,

1H), 7.50 (d, *J* = 1.1 Hz, 1H), 7.49 – 7.41 (m, 2H), 7.40 – 7.36 (m, 4H), 7.34 – 7.21 (m, 3H), 7.09 (dd, *J* = 8.5, 1.7 Hz, 1H). ^{13}C NMR (151 MHz, $CDCl_3$) δ 142.1, 141.4, 138.9, 137.9, 136.0, 135.5, 133.1, 132.2, 131.7, 130.7, 130.6, 130.5, 129.1, 128.4, 128.1, 127.6, 127.5, 127.3, 126.3, 126.2, 117.9, 110.6 (1 carbon peak is missing due to overlap). IR (ATR) υ (cm^{-1}): 3065, 3055, 2959, 2924, 2801, 2220, 1718, 1609, 1514, 1458, 1261, 826, 784, 692, 613, 538.HRMS (GC-MS): m/z [*M*+1]+ calcd for ($C_{23}H_{14}ClN$): 339.0815; found: 339.0795.

Synthesis of 2'-(anthracen-2-yl)-6-chloro-[1,1'-biphenyl]-3-carbonitrile (7b)

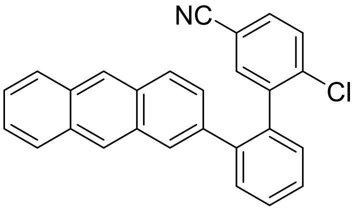

2-bromoanthracene (386 mg, 1.5 mmol) was dissolved in dry THF (5 mL) and cooled down to –78 °C. BuLi 2.5 M in hexane (0.9 mL, 2.3 mmol) was added dropwise and the solution was left stirring under argon atmosphere for 1 h. Then, trimethyl borate (0.251 mL, 2.25 mmol) was added dropwise and the mixture was allowed to reach room temperature. The mixture was quenched with MeOH (5 mL), washed twice with water (2 times 20 mL) and brine (10 mL). The crude was dried under vacuum and directly used in the following step. 2'-bromo-6-chloro-[1,1'-biphenyl]-3-carbonitrile (263 mg, 0.9 mmol), dimethyl anthracen-2-ylboronate (270 mg, 1.08 mmol), [$PdCl_2$(dtbpf)] (12 mg, 0.018 mmol), NEt_3 (0.376 mL, 2.7 mmol) and Kolliphor (2% w/w) were dissolved in 0.5 mL of toluene and 4.5 mL of H_2O. After sonicating for 30 seconds, the mixture was stirred at 60 °C for 2 h. Later, the mixture was cooled down to room temperature and filtered over a silica plug using CH_2Cl_2 as eluent. The desired product was purified by flash chromatography using a mixture of heptane/EtOAc with gradient from 100% to 90:10. The pure compound was obtained as white solid (255 mg, 0.65 mmol, 73% yield), m.p. 202 – 203 °C. ^{1}H NMR (600 MHz, $CDCl_3$): δ 8.22 (s, 1H), 8.15 (s, 1H), 7.88 – 7.81 (m, 2H), 7.70 (d, *J* = 8.8 Hz, 1H), 7.62 (s, 1H), 7.51 (dd, *J* = 7.6, 1.2 Hz, 1H), 7.45 (td, *J* = 7.5, 1.3 Hz, 1H), 7.42 (d, *J* = 1.9 Hz, 1H), 7.37 (td, *J* = 7.5, 1.5 Hz, 1H), 7.33 (m, 2H), 7.28 – 7.19 (m, 3H), 7.07 (dd, *J* = 8.8, 1.6 Hz, 1H).^{13}C NMR (151 MHz, $CDCl_3$) δ 142.1, 141.4, 138.9, 137.2, 136.0, 135.5, 132.0, 131.9, 131.7, 131.2, 130.6, 130.5, 130.4, 129.1, 128.5, 128.2, 128.1, 128.0, 127.5, 127.0, 126.5, 126.0, 125.6, 125.5, 117.9, 110.6 (1 carbon peak is missing due to overlap). IR (ATR) υ (cm^{-1}): 3065, 3055, 2959, 2924, 2801, 2220, 1718, 1609, 1514, 1458, 1261, 826, 784, 692, 613, 538.HRMS (LD-TOF): m/z [*M*+1]$^{+}$ calcd for ($C_{27}H_{16}ClN$): 389.0971; found: 389.0961

Synthesis of 6-chloro-2'-(pyren-1-yl)-[1,1'-biphenyl]-3-carbonitrile (7c)

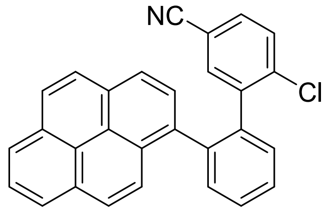

1-bromopyrene (281 mg, 1 mmol) was dissolved in dry THF (5 mL) and cooled down to –78 °C. BuLi 2.5 M in hexane (0.6 mL, 1.5 mmol) was added dropwise and the solution was left stirring under argon atmosphere for 1 h. Then, trimethyl borate (0.167 mL, 1.5 mmol) was added dropwise and the mixture was allowed to reach room temperature. The mixture was quenched with MeOH (5 mL), washed twice with water (2 times 20 mL) and brine (10 mL). The crude was dried under vacuum and directly used in the following step. 2'-bromo-6-chloro-[1,1'-biphenyl]-3-carbonitrile (263 mg, 0.9 mmol), dimethyl pyren-1-ylboronate (296 mg, 1.1 mmol), [$PdCl_2$(dtbpf)] (12 mg, 0.018 mmol), NEt_3 (0.376 mL, 2.7 mmol) and Kolliphor (2% w/w) were dissolved in 0.5 mL of toluene and 4.5 mL of H_2O. After sonicating for 30 seconds, the mixture was stirred at 60 °C for 2 h. Later, the mixture was cooled down to room temperature and filtered over a silica plug using CH_2Cl_2 as eluent. The pure compound was obtained impure and used directly in the next step without full characterisation. HRMS (Maldi-TOF): m/z $[M+1]^+$ calcd for ($C_{29}H_{16}ClN$): 413.0971; found: 413.2656.

Synthesis of 6-chloro-2'-(perylen-1-yl)-[1,1'-biphenyl]-3-carbonitrile (7d)

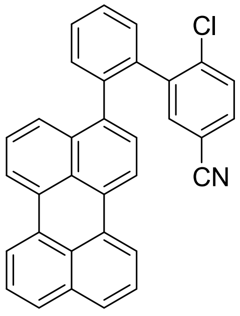

3-bromoperylene (330 mg, 1 mmol) was dissolved in dry THF (10 mL) and cooled down to –78 °C. BuLi 2.5 M in hexane (0.9 mL, 2.3 mmol) was added dropwise and the solution was left stirring under argon atmosphere for 1 h. Then, trimethyl borate (0.251 mL, 2.3 mmol) was added dropwise and the mixture was allowed to reach room temperature. The mixture was quenched with MeOH (5 mL), washed twice with water (2x20 mL) and brine (10 mL). The crude was dried under vacuum and directly used in the following step. 2'-bromo-6-chloro-[1,1'-biphenyl]-3-carbonitrile (263 mg, 0.9 mmol), dimethyl perylen-3-ylboronate (349.9 mg, 1.1 mmol), [$PdCl_2$(dtbpf)] (12 mg, 0.02 mmol), NEt_3 (0.376 mL, 2.7 mmol) and Kolliphor (2% w/w) were dissolved in 0.5 mL of toluene and 4.5 mL of H_2O. After sonicating for 30 seconds, the mixture was stirred at 60 °C for 2 h. Later, the mixture was cooled down to room

temperature and filtered over a silica plug using CH_2Cl_2 as eluent. The desired product was purified by flash chromatography using a mixture of heptane/EtOAc whit gradient from 100% to 90:10. The pure compound was obtained as orange solid (256 mg, 0.55 mmol, 55% yield), m.p. 213 – 215 °C. ^1H NMR (600 MHz, $CDCl_3$) δ 8.22 – 7.97 (m, 4H), 7.68 (s, 1H), 7.66 (s, 1H), 7.61 – 7.31 (m, 9H), 7.28 – 7.26 (m, 1H), 7.25 – 7.17 (m, 2H). ^{13}C NMR (151 MHz, $CDCl_3$) δ 134.7, 131.7, 131.6, 131.4, 131.1, 130.9, 130.7, 130.6, 130.18, 130.0, 128.8, 128.7, 128.6, 128.0, 127.9, 127.9, 127.8, 127.7, 126.7, 126.6, 126.6, 126.5, 126.5, 126.3, 126.2, 125.5, 120.6, 120.4, 120.3, 120.3, 120.1, 119.3, 119.3. IR (ATR) υ (cm^{-1}): 3065, 3051, 2988, 2934, 2924, 2801, 2744, 2223, 1711, 1631, 1614, 1458, 1222, 963, 784, 692, 614, 538. HRMS (Maldi-TOF): m/z $[M+1]^+$ calcd for ($C_{33}H_{18}ClN$): 463.1122; found: 463.1113

Synthesis of 2',5'-dibromo-1,1':4',1''-terphenyl

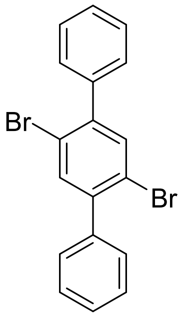

1,4-Dibromo-2,5-diiodobenzene (975 mg, 2 mmol), Phenylboronic acid (488 mg, 2 mmol), [PdCl2(dtbpf)] (26 mg, 0.04 mmol), NEt_3 (0.836 mL, 6 mmol) and Kolliphor (4% w/w) were dissolved in 1 mL of toluene and 9 mL of H_2O. After sonicating for 30 seconds, the mixture was stirred at 60 °C for 2 h. Later the mixture was cooled down to room temperature and filtered over a silica plug using CH_2Cl_2 as eluent. The desired product was purified by flash chromatography using heptane as eluent. The pure compound was obtained as white solid (748 mg, 1.9 mmol, 96% yield). ^1H NMR (600 MHz, $CDCl_3$) δ 7.65 (s, 2H), 7.50 – 7.38 (m, 10H). ^{13}C NMR (151 MHz, $CDCl_3$) δ 142.9, 139.5, 135.2, 129.3, 128.2, 128.1, 121.4. HRMS (GC-MS): m/z calcd for $C_{18}H_{12}Br_2$ $[M+H]^+$: 388.1020; found: 387.9284. Spectroscopic characterization in accordance with literature.[15]

Synthesis of 2,2''-dibromo-4,4''-dimethoxy-1,1':4',1''-terphenyl

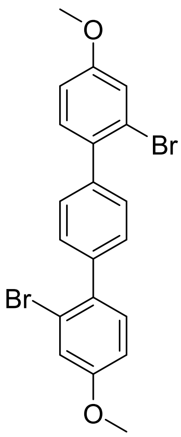

Synthesized in accordance with *general procedure II,* using 3-bromo-4-iodoanisole (2065 mg, 6.6 mmol), Benzene-1,4-diboronic acid (497 mg, 3 mmol), $Pd_2(PPh_3)_4$ (173 mg, 0.15 mmol) and K_2CO_3 (1741 mg, 3 mmol) 10 mL of toluene/H_2O (2:1). The mixture was stirred at 100 °C for 20 h. The desired product was purified by flash chromatography using a mixture of heptane/EtOAc whit gradient from 100% to 90:10. The pure compound was obtained as white solid (583 mg, 1.1 mmol, 65% yield), m.p. 145 – 148 °C. ^{1}H NMR (600 MHz, $CDCl_3$) δ 7.46 (s, 4H), 7.34 (d, J = 8.5 Hz, 2H), 7.26 (d, J = 2.5 Hz, 2H), 6.96 (dd, J = 8.5, 2.5 Hz, 2H), 3.87 (s, 6H). ^{13}C NMR (151 MHz, $CDCl_3$) δ 159.3, 139.6, 134.7, 131.8, 129.2, 122.8, 118.3, 113.6, 55.6 (1 carbon peak is missing due to overlap). IR (ATR) υ (cm^{-1}): 1580, 1462, 1418, 1209, 1178, 1002, 862, 748, 697, 658, 449. HRMS (ESI): m/z calcd for $C_{20}H_{16}Br_2O_2$ $[M+H]^+$: 448.1540 found: 448.1553.

General procedure III

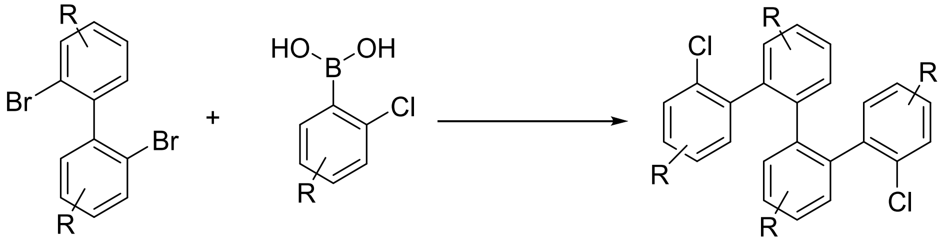

The mixture of the relevant boronic acid (2-3 eq), aryl iodine/bromine (1 eq), base (2-2.5 eq) in the given solvent is degassed with Ar bubbling for 15 min. The given Pd catalyst (0.04-0.1 eq) is added, and the mixture is degassed for additional 5 min out. After the given reaction time, the mixture is cooled to rt, water is added, and the mixture is extracted with EtOAc (3x10 mL). The organic phase is dried over $MgSO_4$, filtered, and concentrated *in vacuo*. The product was isolated by flash chromatography on SiO_2 using a mixture of heptane/EtOAc or heptane/CH_2Cl_2 as eluent.

Synthesis of 6,6''''-dichloro-[1,1':2',1'':4'',1''':2''',1''''-quinquephenyl]-3,3''''-dicarbonitrile (9a)

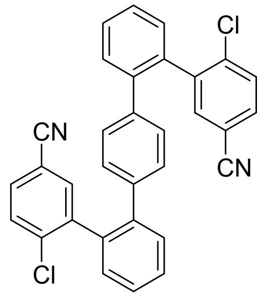

2'-bromo-6-chloro-[1,1'-biphenyl]-3-carbonitrile (564 mg, 2 mmol), 1,4-phenylenediboronic acid (166 mg, 1 mmol), [$PdCl_2$(dtbpf)] (26 mg, 0.04 mmol), NEt_3 (0.836 mL, 6 mmol) and Kolliphor (4% w/w) were dissolved in 1 mL of toluene and 9 mL of H_2O. After sonicating for 30 seconds, the mixture was stirred at 60 °C for 2 h. Later the mixture was cooled down to room temperature and filtered over a silica plug using CH_2Cl_2 as eluent. The desired product was purified by flash chromatography using a mixture of heptane/EtOAc whit gradient from 100% to 70:30. The compound was obtained impure and used directly in the next step without full characterisation. HRMS (ESI): m/z $[M+1]^+$ calcd for ($C_{32}H_{18}N_2Cl_2$): 523.1342; found: 523.0743.

Synthesis of 2,2''''-dichloro-4''',5'-dimethoxy-[1,1':2',1'':4'',1''':2''',1''''-quinquephenyl]-4,4''''-dicarbonitrile (9b)

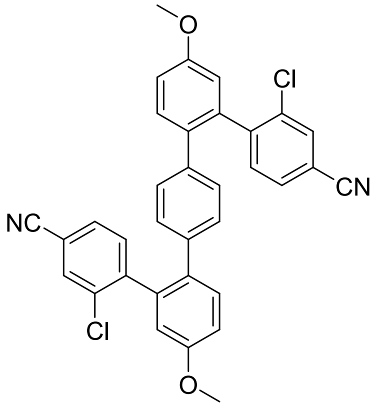

2,2''-dibromo-4,4''-dimethoxy-1,1':4',1''-terphenyl (300 mg, 0.669 mmol), (2-chloro-5-cyanophenyl)boronic acid (267 mg, 1.5 mmol), [$PdCl_2$(dtbpf)] (17.5 mg, 0.027 mmol), NEt_3 (0.56 mL, 4.0 mmol) and Kolliphor (2% w/w) were dissolved in 0.5 mL of toluene and 4.5 mL of H_2O. After sonicating for 30 seconds, the mixture was stirred at 60 °C for 2 h. Later the mixture was cooled down to room temperature and filtered over a silica plug using CH_2Cl_2 as eluent. The desired product was purified by flash chromatography using a mixture of heptane/EtOAc whit gradient from 100% to 90:10. The pure compound was obtained as white solid (243 mg, 0.43 mmol, 65% yield), m.p. 155 – 158 °C. ^1H NMR (600 MHz, $CDCl_3$) δ 7.49 – 7.39 (m, 6H), 7.35 (dd, J = 8.5, 4.6 Hz, 2H), 7.03 (m, 2H), 6.86 (s, 4H), 6.79 (t, J = 3.0 Hz, 2H), 3.86 (s, 6H). ^{13}C NMR (151 MHz, $CDCl_3$) δ 158.7, 141.9, 138.6, 138.4, 136.7, 135.3, 133.5, 131.6, 131.3, 130.5, 128.9, 118.1, 115.7, 115.7, 114.6, 110.5, 55.5. IR (ATR) υ (cm-1) = 2150,

2041, 1968, 996, 751, 661, 613, 569, 529. HRMS (ESI): m/z [M]+ calcd for $C_{34}H_{22}Cl_2N_2O_2$ [M+H]$^+$: 561.1131; found: 561.1133.

Synthesis of dimethyl 2,2''-dichloro-2',5'-diphenyl-[1,1':4',1''-terphenyl]-4,4''-dicarboxylate (10a)

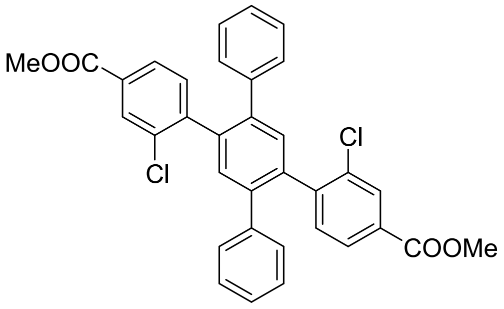

2',5'-dibromo-1,1':4',1''-terphenyl (388 mg, 1 mmol), 2-Chloro-4-(methoxycarbonyl)phenylboronic acid (472 mg, 2.2 mmol), [$PdCl_2$(dtbpf)] (26 mg, 0.04 mmol), NEt_3 (0.836 mL, 6 mmol) and Kolliphor (2% w/w) were dissolved in 0.5 mL of toluene and 4.5 mL of H_2O. After sonicating for 30 seconds, the mixture was stirred at 60 °C for 2 h. Later the mixture was cooled down to room temperature and filtered over a silica plug using CH_2Cl_2 as eluent. The desired product was purified by flash chromatography using a mixture of heptane/EtOAc whit gradient from 100% to 90:10. The pure compound was obtained as white solid (224 mg, 0.70 mmol, 70% yield), m.p. 142 – 144 °C. ^{1}H NMR (600 MHz, $CDCl_3$) δ 8.05 (m, 2H), 7.80 (m, 2H), 7.47 (m, 2H), 7.27 (t, J = 7.2 Hz, 2H), 7.26 – 7.16 (m, 10H), 3.91 (s, 6H). ^{13}C (151 MHz, $CDCl_3$) δ 165.8, 144.5, 139.6, 137.2, 133.8, 132.5, 132.4, 132.2, 130.7, 130.5, 129.3, 128.0, 127.4, 127.1, 52.4. IR (ATR) υ (cm^{-1}): 3071, 3025, 2952, 1722, 1700, 1428, 1396, 1276, 1266, 1241, 1231, 1107, 1079, 1022, 909, 852, 770, 757, 746, 699, 691, 552, 529. HRMS (ESI): m/z [M+Na]$^+$ calcd for ($C_{34}H_{24}Cl_2O_4$): 589.0944; found: 589.0945.

Synthesis of dimethyl 2,2''-dichloro-2'-(4-(dimethylamino)phenyl)-[1,1':3',1''-terphenyl]-4,4''-dicarboxylate (12a)

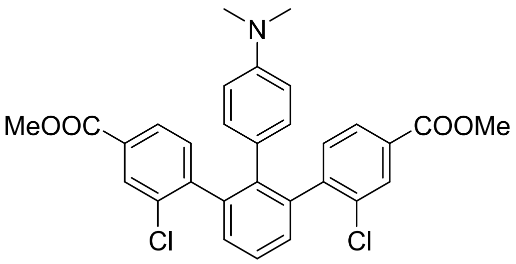

2',6'-dibromo-N,N-dimethyl-[1,1'-biphenyl]-4-amine (120 mg, 0.34 mmol), 2-Chloro-4-(methoxycarbonyl)phenylboronic acid (217.39 mg, 1.0 mmol), [$PdCl_2$(dtbpf)] (11 mg, 0.02 mmol), NEt_3 (0.28 mL, 2.0 mmol) and Kolliphor (2% w/w) were dissolved in 0.5 mL of toluene and 4.5 mL of H_2O. The mixture was stirred at 60 °C for 3 h. Later the mixture was cooled down to room temperature and filtered over a silica plug using CH_2Cl_2 as eluent. The desired product was purified by flash chromatography

using a mixture of heptane/EtOAc whit gradient from 100% to 80:20. The pure compound was obtained as white solid (145 mg, 0.27 mmol, 80% yield), m.p. 148 – 151 °C. ^{1}H NMR (600 MHz, $CDCl_3$) δ 7.93 (m, 2H), 7.65 (m, 2H), 7.42 – 7.26 (m, 3H), 7.06 (m, 2H), 6.60 (m, 2H), 6.16 (dd, J = 8.6, 7.2 Hz, 2H), 3.83 (s, 6H), 2.71 (s, 6H). ^{13}C (151 MHz, $CDCl_3$) δ 166.0, 148.8, 148.7, 145.7, 145.5, 138.8, 132.5, 132.0, 130.8, 130.4, 130.4, 130.2, 129.9, 129.8, 127.0, 127.0, 125.8, 111.1, 52.3, 40.1 (more peaks detected due to partially hindered rotation of the methyl 3-chlorobenzoate moiety). IR (ATR) υ (cm^{-1}): 3071, 3001, 2986, 2951, 1715, 1700, 1678, 1428, 1396, 1276, 1266, 1241, 1231, 1214, 1107, 1079, 1022, 909, 852, 770, 757, 746, 699, 691, 552, 529. HRMS (ESI): m/z [M]$^+$ calcd for ($C_{30}H_{25}Cl_2NO_4$): 534.1233; found: 534.1227.

Synthesis of dimethyl 2,2''-dichloro-2'-(1H-pyrrol-1-yl)-[1,1':3',1''-terphenyl]-4,4''-dicarboxylate (12b)

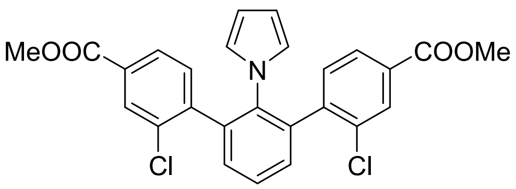

1-(2,6-dibromophenyl)-1H-pyrrole (520 mg, 1.73 mmol), 2-Chloro-4-(methoxycarbonyl)phenylboronic acid (1111 mg, 5.18 mmol), [Pd(dtbpf)Cl_2] (45 mg, 0.07 mmol), NEt_3 (1.44 mL, 10.37 mmol) and Kolliphor (2% w/w) were dissolved in 1 mL of toluene and 9 mL of H_2O. The mixture was stirred at 60 °C for 3 h. Later the mixture was cooled down to room temperature and filtered over a silica plug using CH_2Cl_2 as eluent. The desired product was purified by flash chromatography using a mixture of heptane/EtOAc whit gradient from 100% to 90:10. The pure compound was obtained as white solid (735 mg, 1.53 mmol, 88% yield), m.p. 137 – 140 °C. ^{1}H NMR (600 MHz, $CDCl_3$) δ 7.98 (m, 2H), 7.78 – 7.59 (m, 2H), 7.45 (d, J = 7.7 Hz, 1H), 7.43 – 7.30 (m, 2H), 7.05 (m, 2H), 6.21 (m, 2H), 5.67 (m, 2H), 3.82 (s, 6H). ^{13}C (151 MHz, $CDCl_3$) δ 165.7, 142.2, 141.8, 137.6, 136.7, 133.7, 133.5, 131.3, 130.9, 130.8, 130.5, 127.4, 126.9, 122.8, 122.2, 109.0, 108.4, 52.3 (more peaks detected due to partially hindered rotation of the methyl 3-chlorobenzoate moiety). IR (ATR) υ (cm^{-1}): 3071, 3025, 2952, 2230, 1593, 1578, 1501, 1466, 1331, 1319, 1276, 1266, 1069, 1022, 922, 909, 852, 837, 760, 733, 613, 498. HRMS (ESI): m/z [M+Na]$^+$ calcd for ($C_{26}H_{19}Cl_2NO_4$): 502.0583; found: 502.0586.

Synthesis of 6,6'''-dichloro-[1,1':2',1'':2'',1'''-quaterphenyl]-3,3'''-dicarbonitrile (13a)

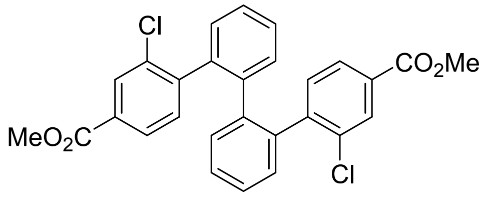

2,2'-dibromo-1,1'-biphenyl (398 mg, 1.28 mmol), 2-chloro-5-cyanophenyl)boronic acid (694 mg, 3.83 mmol), [Pd(dtbpf)Cl_2] (33 mg, 0.051 mmol), NEt_3 (777 mg, 7.7 mmol), and Kolliphor (2% w/w) were dissolved in 1 mL of toluene and 9 mL of H_2O. After sonicating for 30 seconds, the mixture was stirred at 60 °C for 3.5 h. After cooling to r.t. the mixture was diluted with CH_2Cl_2 and filtered through a silica plug. The desired product was purified by flash chromatography using a mixture of petroleum ether/ethyl acetate whit gradient from 100% to 90:10. Resulting material was further recrystallized from EtOH. The pure compound was obtained as white solid (420 mg, 0.99 mmol, yield: 70%), m.p. 244 – 246°C. ^{1}H-NMR (600 MHz, $CDCl_3$, recorded at room temperature) δ 8.10 – 7.86 (m, 2H), 7.73 – 7.27 (m, 8H), 7.25 – 7.20 (m, 2H), 6.91 – 6.84 (m, 2H), 3.93 (s, 6H). ^{13}C-NMR (151 MHz, $CDCl_3$, recorded at 50 °C) δ 165.9, 139.8, 133.3, 131.9, 131.6, 131.2, 130.5, 128.0, 126.9, 52.3 (4 peaks missing due to overlap). IR (ATR) υ (cm^{-1}): 3090, 3062, 2229, 1458, 1434, 1387, 1075, 1022, 901, 822, 779, 768, 756, 611, 403. HRMS (LD-TOF): *m/z* calcd for $C_{26}H_{15}Cl_2N_2$ [*M*+Na]$^+$: 513.0631; found: 513.0622.

General procedure IV

The relevant aryl chloride (1 eq) and PXX (0.02 – 0.04 eq) is placed in a Schlenk tube and set under vacuum for 5 min. After backfilling with Ar and under Ar flow, they are suspended in dry DMSO. DIPEA (1.4 – 3 eq) is added and the mixture freeze pump thawed (3x, frozen with Ar (l), set under vacuum for 5 min and thawed) and finally backfilled with Ar. After the reaction time (15 h unless specified otherwise) under LED irradiation, the mixture is extracted with EtOAc (3x10 mL). The organic phase was washed with brine, dried over $MgSO_4$, and concentrated *in vacuo*. The product is isolated by flash chromatography on SiO_2 using a mixture of heptane/EtOAc or heptane/CH_2Cl_2 as eluent.

Synthesis of triphenylene-2-carbonitrile (2)

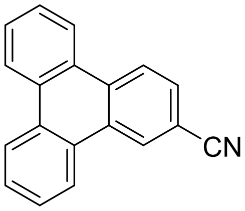

Synthesized in accordance with *general procedure IV* using **1** (58 mg, 0.2 mmol), PXX (1 mg, 0.004 mmol), DIPEA (0.05 mL, 0.28 mmol) and dry DMSO (1 mL). The desired product was purified by flash chromatography using a mixture of heptane/EtOAc 95:5. The pure compound was obtained as yellow solid (36 mg, 0.15 mmol, yield: 72%). ^{1}H-NMR (600 MHz, $CDCl_3$) δ 8.99 (d, *J* = 1.5 Hz, 1H), 8.75 (d, *J* = 8.5 Hz, 1H), 8.73 – 8.69 (m, 2H), 8.67 (dd, *J* = 8.0, 1.4 Hz, 1H), 8.65 – 8.61 (m, 1H), 7.89 (dd, *J* = 8.5, 1.6 Hz, 1H), 7.82 – 7.72 (m, 4H). ^{13}C-NMR (75 MHz, $CDCl_3$) δ 132.9, 130.8, 130.1, 130.0, 129.0, 128.5, 128.4, 128.3, 128.2, 127.9, 127.8, 124.3, 124.0, 123.6, 123.5, 123.3, 119.5, 110.6 (1 carbon peak is missing due to overlap). LRMS (ESI): *m/z* calcd for $C_{19}H_{11}N$ [*M*]$^+$: 253.09; found: 253.08. Spectroscopic characterization in accordance with literature.[16]

Synthesis of triphenylene-1-carbonitrile (4a)

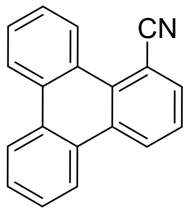

Synthesized in accordance with *general procedure IV,* using **3a** (58 mg, 0.2 mmol), PXX (1 mg, 0.004 mmol), DIPEA (0.05 mL, 0.28 mmol) and dry DMSO (1 mL). The desired product was purified by flash chromatography using a mixture of heptane/EtOAc 95:5. The pure compound was obtained as yellow solid (36 mg, 0.14 mmol, yield: 72%). ^{1}H-NMR (300 MHz, $CDCl_3$) δ 9.52 (dd, J = 7.4, 2.2 Hz, 1H), 8.72 (d, J = 8.4 Hz, 1H), 8.56 (dd, J = 7.3, 2.2 Hz, 1H), 8.51 (dd, J = 7.6, 1.8 Hz, 1H), 8.43 (dd, J = 7.6, 1.8 Hz, 1H), 7.96 (dd, J = 7.4, 1.2 Hz, 1H), 7.73 – 7.56 (m, 5H); ^{13}C-NMR (75 MHz, $CDCl_3$) δ 136.1, 131.2, 130.8, 130.6, 130.0, 128.9, 128.5, 128.4, 128.0, 127.7, 127.5, 127.3, 126.3, 125.9, 123.4, 123.3, 121.8, 107.9 (1 carbon peak is missing due to overlap). HRMS (APCI): *m/z* calcd for $C_{19}H_{12}N$ [M+H]$^+$: 254.0970; found: 254.0972. Spectroscopic characterization in accordance with literature.[16]

Synthesis of triphenylene-2-carbonitrile (4b)

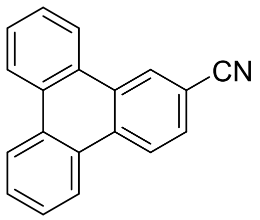

Synthesized in accordance with *general procedure IV,* using **3b** (90 mg, 0.31 mmol), PXX (1.75 mg, 0.01 mmol), DIPEA (0.08 mL, 0.43 mmol) and dry DMSO (2 mL). The desired product was purified by flash chromatography using a mixture of heptane/EtOAc 95:5. The pure compound was obtained as yellow solid (59 mg, 0.23 mmol, yield: 70%) ^{1}H-NMR (600 MHz, $CDCl_3$) δ 8.99 (d, J = 1.5 Hz, 1H), 8.75 (d, J = 8.5 Hz, 1H), 8.73 – 8.69 (m, 2H), 8.67 (dd, J = 8.0, 1.4 Hz, 1H), 8.65 – 8.61 (m, 1H), 7.89 (dd, J = 8.5, 1.6 Hz, 1H), 7.82 – 7.72 (m, 4H). ^{13}C-NMR (75 MHz, $CDCl_3$) δ 132.9, 130.8, 130.1, 130.0, 129.0, 128.5, 128.4, 128.3, 128.2, 127.9, 127.8, 124.3, 124.0, 123.6, 123.5, 123.3, 119.5, 110.6 (1 carbon peak is missing due to overlap). LRMS (ESI): m/z calcd for $C_{19}H_{11}N$ [M]$^+$: 253.09; found: 253.08. Spectroscopic characterization in accordance with literature.[16]

Synthesis of triphenylene-2,3-dicarbonitrile (4c)

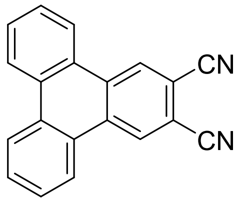

Synthesized in accordance with *general procedure IV,* using **3c** (0.16 mmol, 50 mg), PXX (0.89 mg, 3.1 x 10^{-3} mmol), DIPEA (0.04 mL, 0.22 mmol) and dry DMSO (1 mL). The desired product was purified by precipitation with $CHCl_3$. The pure compound was obtained as white solid (20 mg, 0.07 mmol, yield: 33%), m.p. 223 – 226 °C. ^{1}H-NMR (600 MHz, $CDCl_3$) δ 9.07 (s, 2H), 8.75 (d, *J* = 8.0 Hz, 2H), 8.62 (d, *J* = 8.0 Hz, 2H), 7.85 (m, 4H). ^{13}C-NMR (151 MHz, $CDCl_3$) δ 132.7, 131.2, 130.1, 129.9, 128.5, 126.9, 123.9, 123.8, 116.1, 122.3. IR (ATR) υ (cm^{-1}): 2230, 1716, 1606, 1451, 1395, 1222, 885, 757, 715, 557, 512, 414. HRMS (GC-MS): *m/z* calcd for $C_{20}H_{10}N_2$ [*M*]$^+$: 278.0844; found: 278.0835.

Synthesis of methyl triphenylene-2-carboxylate (4d)

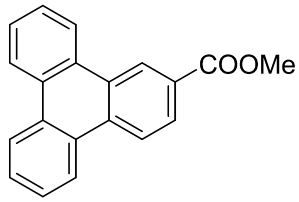

Synthesized in accordance *general procedure IV,* using **3d** (150 mg, 0.46 mmol), PXX (2.62 mg, 0.01 mmol), DIPEA (0.11 mL, 0.65 mmol) and dry DMSO (3 mL). The desired product was purified by flash chromatography using a mixture of heptane/EtOAc 90:10. The pure compound was obtained as yellow solid (100 mg, 0.35 mmol, yield: 72%). ^{1}H-NMR (600 MHz, $CDCl_3$) δ 9.25 (d, *J* = 1.7 Hz, 1H), 8.69 – 8.61 (m, 1H), 8.61 – 8.50 (m, 4H), 8.17 (dd, *J* = 8.6, 1.7 Hz, 1H), 7.70 – 7.55 (m, 4H), 4.03 (s, 3H). ^{13}C-NMR (151 MHz, $CDCl_3$) δ 167.2, 133.1, 130.6, 129.8, 129.4, 129.3, 128.9, 128.9, 128.2, 127.7, 127.5, 127.3, 127.2, 125.4, 123.9, 123.5, 123.4, 123.3, 123.2, 52.3. LRMS (ESI): m/z calcd for $C_{20}H_{15}O_2$ [*M*+H]$^+$: 287.11; found: 287.11. Spectroscopic characterization in accordance with literature.[17]

Synthesis of methyl triphenylene-2-carboxylate (4e)

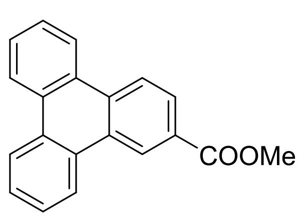

Synthesized in accordance with *general procedure IV,* using **3e** (64 mg, 0.2 mmol), PXX (1 mg, 0.004 mmol), DIPEA (0.05 mL, 0.28 mmol) and dry DMSO (1 mL). The mixture was stirred at r.t. for 16 h. The

desired product was purified by flash chromatography using a mixture heptane/EtOAc 90:10. The pure compound was obtained as yellow solid (41 mg, 0.14 mmol, yield: 74%). ^{1}H-NMR (600 MHz, $CDCl_3$) *δ* 9.25 (d, *J* = 1.7 Hz, 1H), 8.69 – 8.61 (m, 1H), 8.61 – 8.50 (m, 4H), 8.17 (dd, *J* = 8.6, 1.7 Hz, 1H), 7.70 – 7.55 (m, 4H), 4.03 (s, 3H). ^{13}C-NMR (151 MHz, $CDCl_3$) *δ* 167.2, 133.1, 130.6, 129.8, 129.4, 129.3, 128.9, 128.9, 128.2, 127.7, 127.5, 127.3, 127.2, 125.4, 123.9, 123.5, 123.4, 123.3, 123.2, 52.3. LRMS (ESI): *m/z* calcd for $C_{20}H_{15}O_2$ $[M+H]^+$: 287.11; found: 287.11 Spectroscopic characterization in accordance with literature.[17]

Synthesis of triphenylene-2,6-dicarbonitrile (4f)

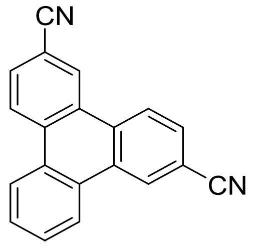

Synthesized in accordance with *general procedure IV*, using **3f** (150 mg, 0.477 mmol), PXX (2.7 mg, 0.009 mmol), DIPEA (0.12 mL, 0.67 mmol) and dry DMSO (2 mL). The desired product was purified by flash chromatography using a mixture of heptane/EtOAc 95:5. The pure compound was obtained as yellow solid (44 mg, 0.20 mmol, yield: 36%) m.p. 236 – 240 °C. ^{1}H-NMR (600 MHz, $CDCl_3$) *δ* 8.91 (d, *J* = 1.4 Hz, 1H), 8.87 (d, *J* = 1.4 Hz, 1H), 8.69 (d, *J* = 8.6 Hz, 1H), 8.64 – 8.51 (m, 3H), 7.87 (m, 2H), 7.79 – 7.70 (m, 2H). ^{13}C-NMR (151 MHz, $CDCl_3$) *δ* 133.9, 131.3, 130.6, 130.4, 129.6, 129.2, 129.1, 128.9, 128.8, 128.6, 128.5, 128.0, 124.7, 124.4, 124.2, 123.6, 118.8, 118.8, 112.1, 111.3. IR (ATR) *υ* (cm^{-1}): 2230, 716, 1606, 1451, 1395, 1222, 885, 757, 715, 557, 512, 414. HRMS (GC-MS): m/z calcd for $C_{20}H_{10}N_2$ $[M]^+$: 278.0844; found: 278.0835.

Synthesis of methyl 10-cyanotriphenylene-2-carboxylate (4g)

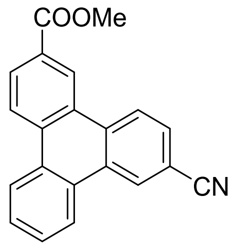

Synthesized in accordance with *general procedure for photocyclization* using **3g** (69 mg, 0.2 mmol), PXX (1 mg, 0.004 mmol), DIPEA (0.05 mL, 0.28 mmol) and dry DMSO (1 mL). The desired product was purified by flash chromatography using a mixture heptane/EtOAc 80:20. The pure compound was obtained as a yellow solid (24 mg, 0.08 mmol, 38% yield), m.p. 248 – 250 °C. ^{1}H-NMR (300 MHz, $CDCl_3$) *δ* 9.13 (d, *J* = 1.5 Hz, 1H), 8.79 (d, *J* = 1.5 Hz, 1H), 8.62 (d, *J* = 8.6 Hz, 1H), 8.59 – 8.54 (m, 2H), 8.48 – 8.45 (m, 1H), 8.26 (dd, *J* = 8.6, 1.6 Hz, 1H), 7.78 (dd, *J* = 8.5, 1.6, Hz, 1H), 7.74 – 7.69 (m, 2H), 4.05 (s,

3H). ^{13}C-NMR (75 MHz, $CDCl_3$) δ 166.7, 134.1, 132.5, 130.1, 129.3, 129.2, 129.0, 128.9, 128.8, 128.7, 128.3, 128.0, 125.9, 124.6, 124.2, 123.7, 123.4, 119.1, 111.2, 52.6 (1 carbon peak is missing due to overlap). IR (ATR) υ (cm^{-1}): 3096, 3035, 2951, 2227, 1715, 1617, 1420, 1286, 1260, 1241, 1190, 1110, 834, 751, 617. HRMS (APCI): *m/z* calcd for $C_{21}H_{14}NO_2$ [*M*+H]$^+$: 312.1025; found: 312.1033.

Synthesis of methyl 11-cyanotriphenylene-2-carboxylate (4j)

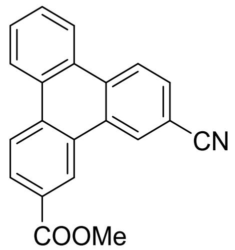

Synthesized in accordance with *general procedure IV,* using **3j** (54 mg, 0.6 mmol), PXX (1 mg, 0.004 mmol), DIPEA (0.05 mL, 0.28 mmol) and dry DMSO (1 mL). The desired product was purified by flash chromatography using a mixture heptane/EtOAc with gradient from 100% to 90:10. The pure compound was obtained as a yellow solid (32 mg, 0.1 mmol, yield: 66%), m.p. 251 – 254 °C. ^{1}H-NMR (600 MHz, $CDCl_3$) δ 9.17 (d, *J* = 1.6 Hz, 1H), 8.93 (d, *J* = 1.6 Hz, 1H), 8.66 – 8.63 (m, 3H), 8.61 – 8.57 (m, 1H), 8.29 (dd, *J* = 8.6, 1.6 Hz, 1H), 7.84 (dd, *J* = 8.6, 1.6 Hz, 1H), 7.79 – 7.73 (m, 2H), 4.07 (s, 3H). ^{13}C-NMR (151 MHz, $CDCl_3$) δ 166.8, 133.5, 130.1, 129.6, 129.3, 129.2, 129.0, 128.6, 128.6, 128.0, 125.3, 124.5, 124.3, 124.2, 123.8, 119.3, 111.1, 52.7 (3 carbon peaks are missing due to overlap). IR (ATR) υ (cm^{-1}): 3048, 3017, 2959, 2222, 1721, 1612, 1485, 1423, 1265, 1238, 1107, 976, 903, 822, 745, 706, 617, 490. HRMS (LD-TOF): *m/z* calcd for $C_{21}H_{14}NO_2$ [*M*+H]$^+$: 312.1025; found: 312.1021.

Synthesis of 6-(tert-butyl)triphenylene-2-carbonitrile (4k)

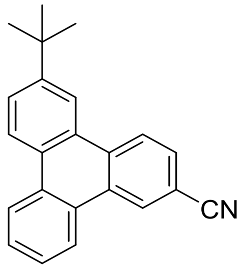

Synthesized in accordance with *general procedure IV,* using **3k** (69 mg, 0.2 mmol), PXX (1 mg, 0.004 mmol), DIPEA (0.05 mL, 0.28 mmol) and dry DMSO (1 mL). The desired product was purified by flash chromatography using a mixture of heptane/EtOAc 90:10. The pure compound was obtained as a yellow solid (43 mg, 0.14 mmol, yield: 69%), m.p. 200 – 202 °C. ^{1}H-NMR (300 MHz, $CDCl_3$) δ 8.92 (s, 1H), 8.72 (d, *J* = 8.7 Hz, 1H), 8.68 – 8.50 (m, 4H), 7.90 – 7.65 (m, 4H), 1.51 (s, 9H). ^{13}C-NMR (75 MHz, $CDCl_3$) δ 150.7, 133.3, 130.2, 130.2, 128.9, 128.6, 128.5, 128.5, 128.1, 128.0, 127.6, 127.2, 124.3, 123.5, 123.4, 123.3, 119.9, 119.6, 110.4, 35.3, 31.5. IR (ATR) υ (cm^{-1}): 3076, 2965, 2906, 2868, 2224, 1492, 1478,

1456, 1408, 1402, 1364, 1264, 909, 887, 828, 777, 761, 749, 718, 631, 611, 477, 424, 420, 405. HRMS (APCI): *m/z* calcd for $C_{23}H_{20}N$ [M+H]$^+$: 310.159; found: 310.1594.

Synthesis of 6-methoxytriphenylene-2-carbonitrile (4l)

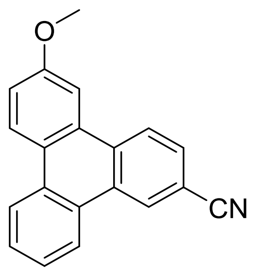

Synthesized in accordance with *general procedure IV,* using **3l** (420 mg, 1.45 mmol), PXX (8 mg, 0.03 mmol), DIPEA (0.35 mL, 2.03 mmol) and dry DMSO (3 mL). The desired product was purified by flash chromatography using a mixture heptane/EtOAc 80:20. The pure compound was obtained as yellow solid (265 mg, 0.93 mmol, yield: 64%), m.p. 196 – 198 °C. ^{1}H-NMR (600 MHz, $CDCl_3$) δ 8.86 (d, J = 1.2 Hz, 1H), 8.57 – 8.47 (m, 4H), 7.92 (d, J = 1.7 Hz, 1H), 7.78 (d, J = 8.5 Hz, 1H), 7.68 (d, J = 8.4 Hz, 1H), 7.63 (d, J = 8.4 Hz, 1H), 7.34 (dd, J = 8.5, 1.2 Hz, 1H), 4.03 (s, 3H). ^{13}C-NMR (151 MHz, $CDCl_3$) δ 159.1, 132.5, 130.3, 130.2, 129.8, 128.7, 128.5, 128.3, 127.1, 126.9, 125.2, 124.7, 124.3, 123.2, 122.9, 119.4, 117.5, 110.6, 106.3, 55.6. IR (ATR) υ (cm^{-1}): 3140, 3113, 3075, 2222, 1609, 1543, 1493, 1447, 1350, 1238, 1099, 880, 833, 760, 745, 710, 610, 420. HRMS (ESI): m/z calcd for $C_{20}H_{13}NO$ [M+H]$^+$: 284.1070; found: 284.1068.

Synthesis of 7-methoxytriphenylene-2-carbonitrile (4m)

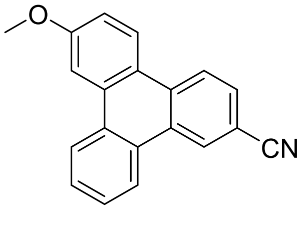

Synthesized in accordance with *general procedure IV,* using **3m** (42 mg, 0.13 mmol), PXX (0.7 mg, 2.6 x 10^{-3} mmol), DIPEA (0.03 mL, 0.18 mmol) and dry DMSO (1 mL). The desired product was purified by flash chromatography using a mixture of heptane/EtOAc whit gradient from 100% to 99:1. The pure compound was obtained as yellow solid (23 mg, 0.08 mmol, yield: 62%), m.p. 197 – 200 °C. ^{1}H-NMR (600 MHz, $CDCl_3$) δ 9.73 (d, J = 8.8 Hz, 1H), 8.93 (d, J = 1.8 Hz, 1H), 8.84 – 8.52 (m, 2H), 8.33 (d, J = 8.1 Hz, 1H), 7.79 (dd, J = 8.8, 1.8 Hz, 1H), 7.72 – 7.64 (m, 3H), 7.24 (d, J = 8.0 Hz, 1H), 4.14 (s, 3H). ^{13}C NMR (151 MHz, $CDCl_3$) δ 159.2, 133.3, 132.8, 130.3, 130.1, 130.0, 128.9, 128.8, 128.4, 128.3, 127.9, 127.4, 124.2, 123.1, 119.6, 119.0, 116.1, 109.9, 109.6, 55.9. IR (ATR) υ (cm^{-1}): 2229, 1598, 1463, 1224, 1210, 1177, 1022, 995, 822, 758, 700, 612,494. HRMS (ESI): m/z calcd for $C_{20}H_{13}NO$ [M+H]$^+$: 284.1070; found: 284.1068.

Synthesis of 6,8-dimethoxytriphenylene-2-carbonitrile (4n)

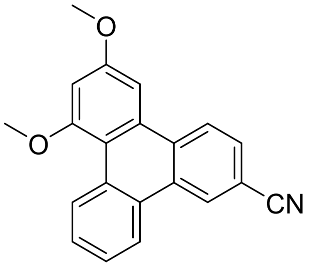

Synthesized in accordance with *general procedure IV,* using **3n** (100 mg, 0.286 mmol), PXX (1.61 mg, 0.006 mmol), DIPEA (0.07 mL, 0.4 mmol) and dry DMSO (1 mL). The desired product was purified by flash chromatography using a mixture of heptane/EtOAc 80:20. The pure compound was obtained as yellow solid (55 mg, 0.170 mmol, yield: 66%), m.p. 213 - 215 °C. ^{1}H-NMR (600 MHz, $CDCl_3$) δ 9.54 – 9.47 (m, 1H), 8.91 (d, J = 1.5 Hz, 1H), 8.58 (d, J = 8.6 Hz, 1H), 8.55 – 8.51 (m, 1H), 7.78 (dd, J = 8.6, 1.5 Hz, 1H), 7.66 – 7.57 (m, 3H), 6.87 (d, J = 2.4 Hz, 1H), 4.08 (s, 3H), 4.02 (s, 3H). ^{13}C-NMR (151 MHz, $CDCl_3$) δ 160.3, 159.0, 132.7, 131.9, 130.8, 130.2, 128.5, 128.4, 128.0, 127.5, 126.3, 125.0, 122.7, 119.4, 115.9, 110.7, 100.9, 98.4, 56.0, 55.5 (1 carbon peak is missing due to overlap). IR (ATR) υ (cm^{-1}): 3151, 3122, 3078, 2224, 1600, 1544, 1493, 1447, 1355, 1238, 1220, 1099, 880, 833, 760, 745, 710, 610, 420. HRMS (GC-MS): m/z calcd for $C_{21}H_{15}NO_2$ [M+H]$^+$: 284.1070; found: 284.1068.

Synthesis of 11-methoxytriphenylene-2-carbonitrile (4o)

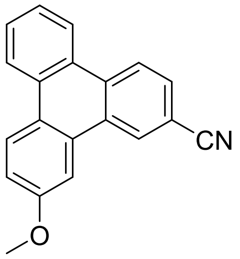

Synthesized in accordance with *general procedure IV,* using **3o** (50 mg, 0.16 mmol), PXX (0.9 mg, 3.1 x 10^{-3} mmol), DIPEA (0.04 mL, 0.22 mmol) and dry DMSO (1 mL). The pure compound was purified by flash chromatography using a mixture of heptane/EtOAc whit gradient from 100% to 99:1. The pure compound as yellow solid (30 mg, 0.11 mmol, yield: 68%), m.p. 190 – 192 °C. ^{1}H-NMR (600 MHz, $CDCl_3$) δ 8.78 (d, J = 1.7 Hz, 1H), 8.63 (d, J = 8.5 Hz, 1H), 8.57 – 8.48 (m, 3H), 7.86 (d, J = 2.6 Hz, 1H), 7.79 (d, J = 8.5, Hz, 1H), 7.71 (ddd, J = 8.2, 7.0, 1.3 Hz, 1H), 7.63 (ddd, J = 8.2, 7.0, 1.3 Hz, 1H), 7.31 (dd, J = 9.0, 2.6 Hz, 1H), 4.04 (s, 3H). ^{13}C-NMR (151 MHz, $CDCl_3$) δ 159.3, 133.3, 130.9, 129.6, 129.5, 128.9, 128.8, 128.3, 127.2, 126.7, 125.1, 124.3, 123.9, 123.8, 122.9, 119.4, 117.4, 110.2, 105.3, 55.6. IR (ATR) υ (cm^{-1}): 2224, 1729, 1614, 1463, 1408, 1128, 1184, 1079, 1034, 963, 710. HRMS (ESI): *m/z* calcd for $C_{20}H_{13}NO$ [M+Na]$^+$: 306.0889; found: 306.0882.

Synthesis of 6,11-bis(dimethylamino)triphenylene-2-carbonitrile (4p)

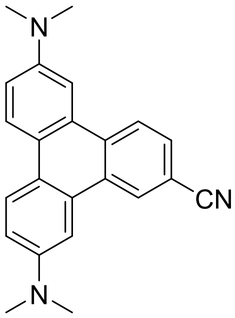

Synthesized in accordance with *general procedure IV,* using **3p** (100 mg, 0.26 mmol), PXX (1.5 mg, 0.005 mmol), DIPEA (0.06 mL, 0.37 mmol) and dry DMSO (1 mL). The desired product was purified by flash chromatography using a mixture of heptane/EtOAc whit gradient from 100% to 90:10. The pure compound was obtained as yellow solid (72 mg, 0.20 mmol, yield: 75%), m.p. 140 – 145 °C. ^{1}H-NMR (600 MHz, $CDCl_3$) δ 8.84 (d, J = 1.6 Hz, 1H), 8.58 (d, J = 8.5 Hz, 1H), 8.35 (dd, J = 9.1, 4.9 Hz, 2H), 7.76 (dd, J = 8.5, 1.7 Hz, 1H), 7.68 (d, J = 2.8 Hz, 1H), 7.62 (d, J = 2.7 Hz, 1H), 7.20 (m, 2H), 3.16 (s, 6H), 3.14 (s, 6H). ^{13}C-NMR (151 MHz, $CDCl_3$) δ 148.8, 148.7, 133.5, 130.4, 128.4, 128.1, 127.8, 127.7, 124.2, 123.6, 123.5, 122.3, 121.4, 119.9, 116.2, 115.6, 109.5, 104.9, 104.2, 40.8. IR (ATR) υ (cm^{-1}): 3487, 3422, 2210, 1546, 1129, 989, 855, 831, 624, 611, 567, 532. HRMS (ESI): *m/z* calcd for $C_{23}H_{21}N_3$ [*M*]$^+$: 339.1730; found: 339.1721.

Synthesis of triphenylene-1,7-dicarbonitrile (4q)

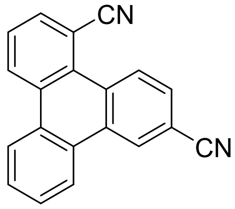

Synthesized in accordance with *general procedure IV,* using **3q** (150 mg, 0.473 mmol), PXX (2.43 mg, 0.01 mmol), DIPEA (0.11 mL, 0.60 mmol) and dry DMSO (2 mL). The mixture was stirred at r.t. for 16 h. The desired product was purified by flash chromatography using a mixture of heptane/EtOAc 95:5. The pure compound was obtained as yellow solid (63 mg, 0.269 mmol, yield: 63%), m.p. 235 – 253 °C. ^{1}H-NMR (600 MHz, $CDCl_3$) δ 9.75 (d, J = 8.7 Hz, 1H), 9.00 (d, J = 1.3 Hz, 1H), 8.96 (d, J = 8.4 Hz, 1H), 8.67 – 8.59 (m, 2H), 8.14 (dd, J = 7.3, 1.2 Hz, 1H), 7.95 (dd, J = 8.7, 1.6 Hz, 1H), 7.87 – 7.78 (m, 3H). ^{13}C-NMR (151 MHz, $CDCl_3$) δ 136.6, 132.3, 131.1, 130.5, 129.1, 129.1, 129.0, 128.9, 128.5, 128.3, 128.1, 128.0, 126.8, 123.6, 123.4, 120.9, 118.7, 112.3, 108.8 (1 carbon peak is missing due to overlap). IR (ATR) υ (cm^{-1}): 2230, 1716, 1606, 1451, 1395, 1222, 885, 757, 715, 557, 512, 414. HRMS (GC-MS): m/z calcd for $C_{20}H_{10}N_2$ [*M*]$^+$: 278.0844; found: 278.0835.

Synthesis of 8-mesityldibenzo[c,e]dibenzo[3,4:5,6][1,2]azaborinino[1,2-a] [1,2]azaborinine-12-carbonitrile (4r)

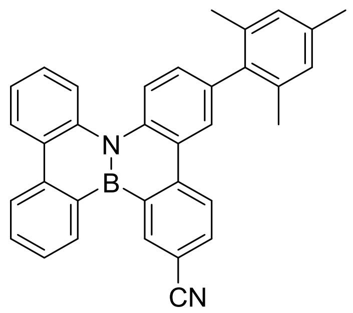

Synthesized in accordance with *general procedure IV,* using **3r** (51 mg, 0.1 mmol), PXX (0.6 mg, 0.002 mmol), DIPEA (0.02 mL, 0.14 mmol) and dry DMSO (2 mL). The desired product was purified by flash chromatography using a mixture of heptane/EtOAc 95:5. The pure compound was obtained as green solid (33 mg, 0.069 mmol, yield: 70%), m.p. 289 – 293 °C. ^{1}H-NMR (600 MHz, $CDCl_3$) δ 8.40 (dd, J = 7.5, 1.2 Hz, 1H), 8.38 (d, J = 8.2 Hz, 1H), 8.33 (dd, J = 7.9, 1.5 Hz, 1H), 7.83 – 7.79 (m, 1H), 7.61 (m 2H), 7.50 – 7.43 (m, 3H), 7.36 (d, J = 8.3 Hz, 1H), 7.35 – 7.31 (m, 3H), 7.17 (dd, J = 8.3, 2.0 Hz, 1H), 7.02 (s, 1H), 6.97 (s, 1H), 2.36 (s, 3H), 2.23 (s, 3H), 2.04 (s, 3H). ^{13}C-NMR (151 MHz, $CDCl_3$) δ 156.2, 141.0, 140.8, 140.6, 139.2, 137.5, 137.1, 136.3, 136.2, 135.9, 132.5, 132.5, 131.9, 131.7, 130.3, 130.2, 128.4, 128.2, 127.7, 127.6, 127.1, 126.9, 125.7, 124.9, 124.6, 122.6, 122.3, 120.2, 118.2, 112.8, 31.9, 22.7, 14.1 (1 carbon peak is missing due to overlap). ^{11}B NMR (193 MHz, $CDCl_3$) δ 34.6. IR (ATR) υ (cm^{-1}): 3064, 2962, 2911, 2855, 2222, 1600, 1569, 1560, 1482, 1442, 1425, 1373, 1356, 1342, 1319, 1361, 1295, 1271, 1250, 1188, 1166, 1105, 1080, 1030, 993, 962, 955, 922, 849, 802, 786, 752, 725, 659, 623, 594, 564, 524, 434. HRMS (LD-TOF): m/z calcd for $C_{34}H_{25}BN_2$ [M+H+Na]$^+$: 496.2085 found: 496.2141.

Synthesis of pyrrolo[1,2-*f*]phenanthridine-10-carbonitrile (6a)

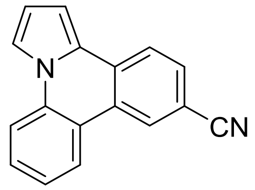

Synthesized in accordance with *general procedure IV,* using **5a** (56 mg, 0.2 mmol), PXX (1 mg, 0.004 mmol), DIPEA (0.05 mL, 0.28 mmol) and dry DMSO (1 mL). The desired product was purified by flash chromatography using a mixture of heptane/EtOAc 80:20. The pure compound was obtained as yellow solid (40 mg, 0.17 mmol, yield: 83%), m.p. 192 – 193 °C. ^{1}H-NMR (300 MHz, $CDCl_3$) δ 8.37 (d, J = 1.5 Hz, 1H), 8.16 (dd, J = 8.2, 1.2 Hz, 1H), 7.92 (d, J = 8.3 Hz, 1H), 7.82 – 7.79 (m, 2H), 7.60 – 7.54 (m, 2H), 7.39 (ddd, J = 8.3, 7.3, 1.2 Hz, 1H), 7.02 (dd, J = 3.9, 1.3 Hz, 1H), 6.77 (dd, J = 3.9, 2.9 Hz, 1H). ^{13}C-NMR (75 MHz, $CDCl_3$) δ 133.2, 130.3, 129.9, 129.3, 127.8, 127.3, 125.0, 124.5, 124.0, 123.3, 120.0, 119.6, 115.2, 115.0, 113.1, 108.7, 105.1. IR (ATR) υ (cm^{-1}): 3140, 3113, 3075, 2222, 1609, 1543, 1493,

1447, 1350, 1238, 1099, 880, 833, 760, 745, 710, 610, 420. HRMS (ESI): *m/z* calcd for $C_{17}H_{10}N_2$ [*M*]$^+$: 242.0839; found: 242.0833.

Synthesis of phenanthro[9,10-b]thiophene-9-carbonitrile (6b) and phenanthro[9,10-c]thiophene-6-carbonitrile (6b')

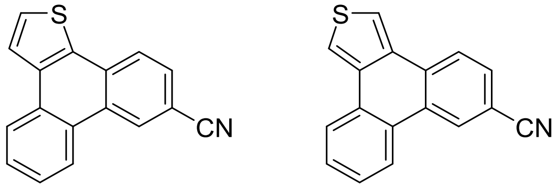

Synthesized in accordance with *general procedure IV,* using **5b** (59 mg, 0.2 mmol), PXX (1 mg, 0.004 mmol), DIPEA (0.05 mL, 0.28 mmol) and dry DMSO (1 mL). The desired product was purified by flash chromatography using a mixture of heptane/EtOAc 80:20. The pure compounds were obtained as yellow solid (25 mg, 0.1 mmol, yield: 48%). **6b**: m.p. 160 – 162 °C. ^{1}H-NMR (300 MHz, $CDCl_3$) δ 8.92 (d, *J* = 1.5 Hz, 1H), 8.58 (dd, *J* = 7.5, 2.0 Hz, 1H), 8.31 (dd, *J* = 7.3, 2.0 Hz, 1H), 8.13 (dd, *J* = 8.3, 0.5 Hz, 1H), 7.98 (d, *J* = 5.4 Hz, 1H), 7.76 (dd, *J* = 8.3, 1.5 Hz, 1H), 7.76 – 7.66 (m, 3H). ^{13}C-NMR (75 MHz, $CDCl_3$) δ 137.3, 135.0, 131.4, 129.1, 129.0, 128.9, 128.4, 127.8, 127.5, 126.9, 125.1, 124.6, 123.5, 119.6, 109.3, (2 carbon peaks are missing due to overlap). IR (ATR) υ (cm^{-1}): 3079, 2961, 2923, 2222, 1610, 1507, 1466, 1446, 1275, 1261, 1236, 1126, 1101, 1097, 1071, 1019, 893, 884, 824, 811, 781, 758, 728, 711, 676, 669, 649, 611, 583, 539, 501, 423, 419. HRMS (ESI) *m/z* calcd for $C_{17}H_{10}NS$ [*M*+H]$^+$: 260.0534; found: 260.0539. **6b'**: m.p. 204 – 206 °C. ^{1}H-NMR (300 MHz, $CDCl_3$) δ 8.62 (d, *J* = 1.4 Hz, 1H), 8.29 (dd, *J* = 7.1, 2.2 Hz, 1H), 8.21 – 8.18 (m, 2H), 8.12 (d, *J* = 3.0 Hz, 1H), 8.07 (d, *J* = 3.0 Hz, 1H), 7.69 (dd, *J* = 8.2, 1.5 Hz, 1H), 7.62 – 7.53 (m, 2H). ^{13}C-NMR (75 MHz, $CDCl_3$) δ 135.3, 133.8, 131.4, 129.9, 129.6, 128.7, 128.5, 128.4, 127.7, 127.5, 125.0, 124.4, 123.5, 119.8, 117.9, 110.2 (1 carbon peak is missing due to overlap). IR (ATR) υ (cm^{-1}): 3107, 3089, 2224, 1608, 1534, 1477, 1468, 1237, 1229, 1187, 1048, 884, 875, 837, 799, 787, 757, 726, 719, 711, 705, 639, 613, 588, 545, 502, 424, 406, 401. HRMS (APCI): *m/z* calcd for $C_{17}H_{10}NS$ [*M*+H]$^+$: 260.0534; found: 260.0545.

Synthesis of dibenzo[f,h]quinoline-10-carbonitrile (6c)

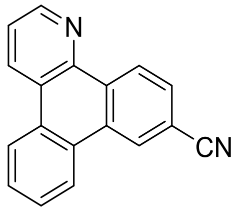

Synthesized in accordance with *general procedure IV,* using **5c** (420 mg, 1.45 mmol), PXX (8 mg, 0.03 mmol), DIPEA (0.35 mL, 2.03 mmol) and dry DMSO (3 mL). The desired product was purified by flash chromatography using a mixture of heptane/EtOAc 80:20. The pure compound was obtained as yellow solid (265 mg, 0.93 mmol, yield: 64%), m.p. 196 – 198 °C. ^{1}H-NMR (600 MHz, $CDCl_3$) δ 9.41 (d, *J* = 8.5

Hz, 1H), 9.00 (dd, *J* = 4.3, 1.6 Hz, 1H), 8.88 (td, *J* = 4.2, 1.5 Hz, 2H), 8.58 (dd, *J* = 6.2, 3.2 Hz, 2H), 7.90 (dd, *J* = 8.4, 1.5 Hz, 1H), 7.76 (dd, *J* = 6.2, 3.3 Hz, 2H), 7.66 (dd, *J* = 8.3, 4.4 Hz, 1H). ^{13}C-NMR (151 MHz, $CDCl_3$) *δ* 149.4, 145.0, 133.8, 131.3, 131.0, 129.2, 129.1, 128.6, 128.5, 128.2, 127.6, 126.6, 125.7, 123.5, 123.5, 123.5, 119.3, 112.2. IR (ATR) *υ* (cm^{-1}): 3140, 3113, 3075, 2222, 1609, 1543, 1493, 1447, 1350, 1238, 1099, 880, 833, 760, 745, 710, 610, 420. HRMS (ESI): m/z calcd for $C_{18}H_{10}N_2$ [*M*+Na]$^+$: 277.0736; found: 277.0739.

Synthesis of benzo[f]tetraphene-3-carbonitrile (8a)

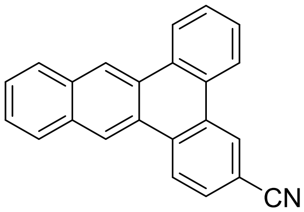

Synthesized in accordance with *general procedure IV,* using **7a** (240 mg, 0.706 mmol), PXX (4 mg, 0.014 mmol, 1.5 mg), DIPEA (0.17 mL, 0.99 mmol) and dry DMSO (2 mL). The desired product was purified by flash chromatography using a mixture of heptane/EtOAc whit gradient from 100% to 90:10. The pure compound was obtained as yellow solid (135 mg, 0.445 mmol, yield: 63%), m.p. 210 – 212 °C. ^{1}H-NMR (600 MHz, $CDCl_3$) *δ* 9.07 (d, *J* = 1.6 Hz, 1H), 9.00 (d, *J* = 8.8 Hz, 1H), 8.85 – 8.80 (m, 1H), 8.73 – 8.67 (m, 2H), 8.65 (d, *J* = 8.8 Hz, 1H), 8.11 (t, *J* = 6.6 Hz, 1H), 8.08 (dd, *J* = 7.4, 1.6 Hz, 1H), 7.89 – 7.78 (m, 3H), 7.73 – 7.64 (m, 2H). ^{13}C-NMR (151 MHz, $CDCl_3$) *δ* 133.6, 132.3, 130.8, 130.1, 130.1, 129.5, 128.6, 128.6, 128.6, 128.5, 127.9, 127.9, 127.7, 126.8, 126.4, 126.1, 124.1, 123.1, 120.6, 119.5, 109.8 (2 carbon peaks are missing due to overlap). IR (ATR) *υ* (cm^{-1}): 2230, 1716, 1606, 1451, 1395, 1222, 885, 757, 715, 557, 512, 414. HRMS (ESI): *m/z* calcd for $C_{23}H_{13}N$ [*M*]$^+$: 303.1043; found: 303.1034.

Synthesis of dibenzo[a,c]tetracene-3-carbonitrile (8b)

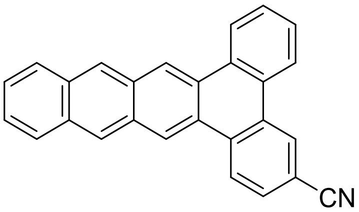

Synthesized in accordance with *general procedure IV,* using **7b** (250 mg, 0.641 mmol), PXX (3.62 mg, 0.013 mmol), DIPEA (0.16 mL, 0.89 mmol) and dry DMSO (2 mL). The desired product was purified by flash chromatography using a mixture of heptane/EtOAc whit gradient from 100% to 90:10. The pure compound was obtained as yellow solid (136 mg, 0.385 mmol, yield: 60%), m.p. 223 – 226 °C. ^{1}H-NMR (600 MHz, $CDCl_3$) *δ* 9.35 (s, 1H), 9.17 (d, *J* = 8.6 Hz, 1H), 9.13 (d, *J* = 1.5 Hz, 1H), 8.72 (ddd, *J* = 8.6, 6.7, 3.7 Hz, 2H), 8.61 – 8.53 (m, 2H), 8.22 (d, *J* = 9.0 Hz, 1H), 8.17 – 8.09 (m, 2H), 7.91 (d, *J* = 8.2 Hz, 1H), 7.88 – 7.80 (m, 2H), 7.66 – 7.59 (m, 2H). ^{13}C-NMR (151 MHz, $CDCl_3$) *δ* 137.1, 134.4, 132.3, 132.0,

131.6, 130.7, 130.0, 130.0, 129.8, 129.5, 128.8, 128.7, 128.7, 128.7, 128.5, 127.9, 127.9, 127.9, 127.5, 126.5, 126.4, 126.2, 124.1, 123.1, 120.6, 106.4 (1 carbon peak is missing due to overlap). IR (ATR) υ (cm^{-1}): 2221, 1718, 1606, 1438, 1400, 1386, 1222, 882, 757, 715, 557, 512, 414. HRMS (LD-TOF): *m/z* calcd for $C_{27}H_{15}N$ $[M]^{+}$: 353.1199; found: 353.1205.

Synthesis of dibenzo[f,pqr]picene-9-carbonitrile (8c)

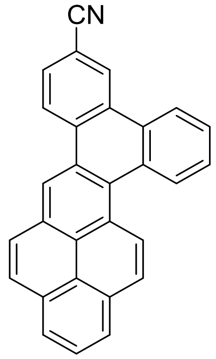

Synthesized in accordance with *general procedure IV,* using **7c** (150 mg, 0.362 mmol), PXX (2.05 mg, 0.007 mmol), DIPEA (0.06 mL, 0.37 mmol) and dry DMSO (2 mL). The desired product was purified by flash chromatography using a mixture of heptane/EtOAc whit gradient from 100% to 90:10. The pure compound was obtained as yellow solid (86 mg, 0.228 mmol, yield: 63%), m.p. 225 – 227 °C. ^{1}H-NMR (600 MHz, $CDCl_3$) δ 9.12 (s, 1H), 9.07 (d, J = 9.3 Hz, 1H), 8.91 (d, J = 1.4 Hz, 1H), 8.86 (m, 2H), 8.60 – 8.56 (m, 1H), 8.18 (m, 4H), 8.08 – 7.99 (m, 2H), 7.85 (dd, J = 8.5, 1.6 Hz, 1H), 7.73 – 7.65 (m, 2H). ^{13}C-NMR (151 MHz, $CDCl_3$) δ 133.4, 131.7, 131.0, 130.5, 130.4, 130.3, 130.3, 129.3, 129.3, 128.6, 128.1, 127.7, 127.6, 127.5, 127.4, 127.4, 127.0, 126.7, 126.4, 126.1, 125.6, 125.6, 125.3, 125.1, 124.6, 123.6, 119.4, 119.0, 110.7. IR (ATR) υ (cm^{-1}): 2220, 1710, 1606, 1438, 1386, 1233, , 1123, 757, 718, 557, 515, 414. HRMS (Maldi-TOF): *m/z* calcd for $C_{29}H_{15}N$ $[M]^{+}$: 377.1199; found: 377.1198.

Synthesis of 6-(2'-chloro-5'-cyano-[1,1'-biphenyl]-2-yl)triphenylene-2-carbonitrile 11a)

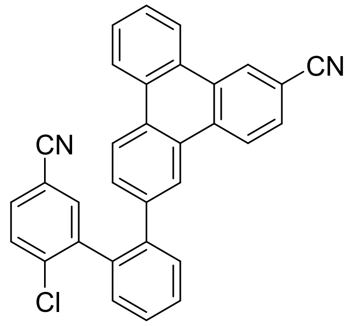

Synthesized in accordance with *general procedure IV,* using **9a** (50 mg, 0.1 mmol), PXX (0.57 mg, 0.002 mmol), DIPEA (0.02 mL, 0.14 mmol) and dry DMSO (1 mL). The desired product was purified by flash chromatography using a mixture of heptane/EtOAc whit gradient from 100% to 90:10. The pure compound was obtained as yellow solid (22 mg, 0.0473 mmol, yield: 47%), m.p. 194 – 196 °C. ^{1}H-NMR

(600 MHz, $CDCl_3$) δ 8.91 (s, 1H), 8.77 (s, 1H), 8.68 – 8.65 (m, 2H), 8.17 (d, J = 8.6 Hz, 1H), 7.86 – 7.77 (m, 4H), 7.72 – 7.59 (m, 3H), 7.54 (d, J = 8.6 Hz, 1H), 7.42 – 7.27 (m, 3H), 7.13 (d, J = 8.8 Hz, 1H). ^{13}C-NMR (151 MHz, $CDCl_3$) δ 142.0, 141.9, 140.9, 140.8, 139.1, 135.8, 135.7, 135.4, 131.6, 131.5, 130.6, 130.5, 130.4, 130.3, 130.3, 130.2, 130.2, 130.2, 129.1, 128.9, 127.4, 125.0, 125.0, 122.2, 122.1, 122.0, 118.0, 110.5, 110.4 (3 carbon peaks are missing due to overlap). IR (ATR) υ (cm^{-1}): 3090, 3062, 2229, 2220, 1458, 1434, 1387, 1075, 1022, 901, 822, 779, 768, 756, 611, 403. HRMS (CI): *m/z* calcd for $C_{32}H_{17}ClN_2$ [*M*+ACN+NH_4]$^+$: 523.1757; found: 523.0731.

Synthesis of 6,15-dimethoxytribenzo[f,k,m]tetraphene-3,12-dicarbonitrile (11b)

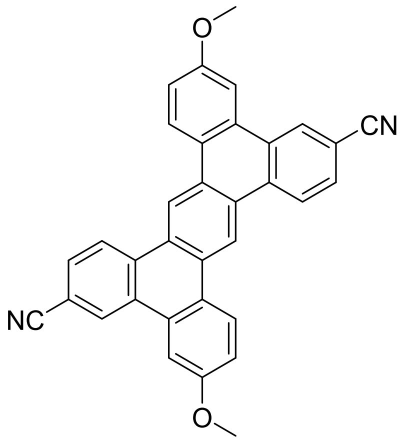

Synthesized in accordance with *general procedure IV,* using **9b** (70 mg, 0.126 mmol), PXX (1.4 mg, 0.005 mmol), DIPEA (0.06 mL, 0.35 mmol) and dry DMSO (1 mL). The desired product was purified by flash chromatography using a mixture of heptane/EtOAc whit gradient from 100% to 90:10. The pure compound was obtained as yellow solid (26 mg, 0.053 mmol, yield: 43%), m.p. 199 – 201 °C. ^{1}H-NMR (600 MHz, $CDCl_3$) δ 9.74 (d, J = 8.9 Hz, 2H), 8.94 (d, J = 1.5 Hz, 2H), 8.64 – 8.56 (m, 4H), 8.33 (d, J = 8.3 Hz, 2H), 7.80 (dd, J = 8.9, 1.7 Hz, 2H), 7.25 (s, 2H), 4.14 (s, 6H). ^{13}C-NMR (151 MHz, $CDCl_3$) δ 156.3, 130.5, 130.0, 128.4, 128.3, 124.5, 124.4, 124.3, 123.5, 120.5, 118.7, 118.1, 115.2, 108.4, 104.3, 55.7, 29.7. IR (ATR) υ (cm^{-1}): 3140, 3113, 3075, 2222, 1609, 1543, 1493, 1447, 1350, 1238, 1099, 880, 833, 760, 745, 710, 610, 420. HRMS (LD-TOF): *m/z* calcd for $C_{34}H_{20}N_2O_2$ [*M*]$^+$: 488.1512; found: 488.1519.

Syntehsis of dimethyl tribenzo[f,k,m]tetraphene-2,11-dicarboxylate (10a)

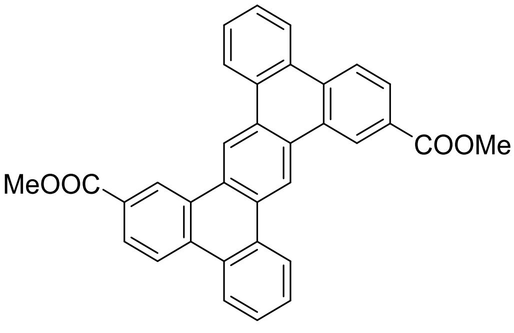

Synthesized in accordance with *general procedure IV,* using **X** (100 mg, 0.176 mmol), PXX (1 mg, 0.004 mmol, 1.5 mg), DIPEA (0.04 mL, 0.247 mmol) and dry DMSO (2 mL). The desired product was purified by flash chromatography using a mixture of heptane/EtOAc whit gradient from 100% to 90:10. The pure compound was obtained as pale yellow solid (40 mg, 0.081 mmol, yield: 46%), m.p. 203 – 205 °C. ^{1}H-NMR (600 MHz, $CDCl_3$) δ 7.97 (m, 2H), 7.74 (t, *J* = 8.6 Hz, 2H), 7.40 (m, 2H), 7.26 – 7.20 (m, 2H), 7.15 – 7.10 (m, 8H), 3.85 (s, 6H). ^{13}C-NMR (151 MHz, $CDCl_3$) δ 165.8, 140.0, 139.9, 139.8, 139.8, 133.8, 132.5, 132.4, 130.7, 130.5, 129.3, 128.0, 127.4, 127.4, 127.0, 99.5, 52.4. IR (ATR) υ (cm^{-1}): 3096, 3035, 2951, 2227, 1715, 1617, 1420, 1286, 1260, 1241, 1190, 1110, 834, 751, 617. HRMS (LD-TOF): *m/z* calcd for $C_{34}H_{22}O_4$ [*M*]$^+$: 494.1513; found: 494.1509.

Syntehsis of dimethyl 2-(dimethylamino)dibenzo[fg,op]tetracene-5,13-dicarboxylate (14a)

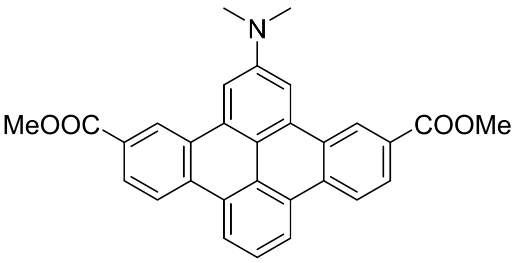

Synthesized in accordance with *general procedure IV,* using **12a** (145 mg, 0.27 mmol), PXX (1.53 mg, 0.01 mmol), DIPEA (0.05 mL, 0.38 mmol) and dry DMSO (2 mL). The desired product was purified by flash chromatography using a mixture of heptane/EtOAc whit gradient from 100% to 90:10. The pure compound was obtained as pale-yellow solid (40 mg, 0.081 mmol, yield: 35% for 15 h reaction time while 77 mg, 0.17 mmol, yield: 62% for 45 h reaction time), m.p. 203 – 205 °C. ^{1}H-NMR (600 MHz, $CDCl_3$) δ 9.44 (d, *J* = 1.3 Hz, 2H), 8.90 (d, *J* = 8.0 Hz, 2H), 8.82 (d, *J* = 8.6 Hz, 2H), 8.35 – 8.29 (m, 4H), 7.96 (t, *J* = 8.0 Hz, 1H), 4.08 (s, 6H), 3.39 (s, 6H). ^{13}C-NMR (151 MHz, $CDCl_3$) δ 167.4, 133.9, 130.5, 129.8, 128.5, 127.5, 127.4, 125.8, 124.9, 124.8, 123.9, 123.3, 106.5, 52.4, 41.1 (2 carbon peaks are missing due to overlap). IR (ATR) υ (cm^{-1}): 3096, 3035, 2951, 2227, 1748, 1715, 1617, 1420, 1286, 1260, 1241, 1190, 1110, 834, 751, 617. HRMS (LD-TOF): m/z calcd for $C_{30}H_{23}NO_4$ [*M*]$^+$: 462.1700; found: 462.1697.

Syntehsis of dimethyl benzo[7,8]indolizino[6,5,4,3-def]phenanthridine-4,12-dicarboxylate (14b)

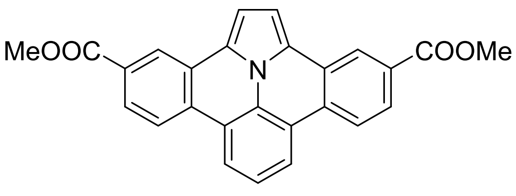

Synthesized in accordance with *general procedure IV,* using **12b** (279 mg, 0.58 mmol), PXX (3.28 mg, 0.01 mmol), DIPEA (0.14 mL, 0.81 mmol) and dry DMSO (3 mL). The desired product was purified by flash chromatography using a mixture of heptane/EtOAc whit gradient from 100% to 90:10. The pure compound was obtained as bright yellow solid (169 mg, 0.41 mmol, yield: 71%), m.p. 213 – 216 °C. ^{1}H-NMR (600 MHz, $CDCl_3$) δ 8.70 (d, J = 1.7 Hz, 2H), 8.34 – 8.25 (m, 4H), 8.03 (dd, J = 8.5, 1.7 Hz, 2H), 7.59 (t, J = 8.0 Hz, 1H), 7.38 (s, 2H), 4.01 (s, 6H). ^{13}C-NMR (151 MHz, $CDCl_3$) δ 166.7, 130.9, 129.9, 128.6, 126.5, 126.2, 125.3, 124.8, 123.9, 123.2, 122.2, 122.0, 104.8, 52.4. IR (ATR) υ (cm^{-1}): 3031, 3025, 2947, 2231, 1750, 1715, 1633, 1420, 1231, 1228, 1206, 1119, 1110, 834, 751, 617. HRMS (LD-TOF): m/z calcd for $C_{26}H_{17}NO_4$ [M]$^+$: 407.1152; found: 407.1140.

Synthesis of dimethyl dibenzo[fg,op]tetracene-5,12-dicarboxylate (14c)

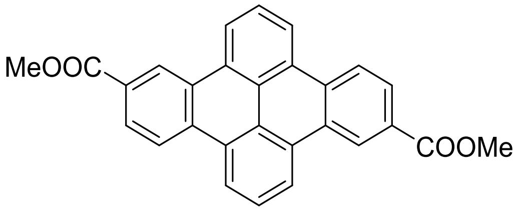

Synthesized in accordance with *general procedure IV,* using **13a** (59 mg, 0.12 mmol), PXX (0.7 mg, 0.002 mmol), DIPEA (0.06 mL, 0.35 mmol) and dry DMSO (2 mL). The desired product as precipitate was filtered and washed with DMSO (15 mL). The pure compound was obtained as yellow solid (9 mg, 0.02 mmol, yield: 18%), m.p. 197 – 200 °C. ^{1}H-NMR (700 MHz, $CDCl_3$) δ 9.55 (d, J = 1.7 Hz, 2H), 9.10 (d, J = 8.0 Hz, 2H), 9.00 (d, J = 8.0 Hz, 2H), 8.89 (d, J = 8.1 Hz, 2H), 8.37 (dd, J = 8.1, 1.7 Hz, 2H), 8.15 (t, J = 8.0 Hz, 2H), 4.08 (s, 6H).^{13}C-NMR couldn't be recorded due to the insolubility of the product. IR (ATR) υ (cm^{-1}): 3096, 3035, 2951, 2227, 1715, 1617, 1420, 1286, 1260, 1241, 1190, 1110, 834, 751, 617. HRMS (LD-TOF): m/z calcd for $C_{28}H_{18}O_4$ [M]+: 441.1097; found: 441.1096.

Synthesis of peri-Xanthenoxanthene (PXX)

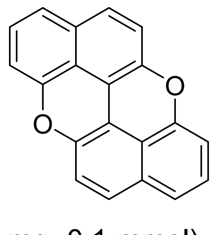

Under ambient air, 1,1'-bi-2-naphthol (28.6 mg, 0.1 mmol), K_2CO_3 (27.6 mg, 0.2 mmol), CuCl (3.0 mg, 0.03 mmol). *m*-xylene was added (0.5 mL), followed by N-methylimidazole (4.8 µL, 0.06 mmol). The

vessel was heated at 120 °C for 20 h. After removal of solvent under reduced pressure, the residue was filtered through silica gel short pad with CHCl3 as the eluent and recrystallized from toluene to give peri-Xanthenoxanthene as yellow solid (18.0 mg, 0.064 mmol, 64% yield). ^{1}H-NMR (400 MHz, $CDCl_3$) *δ* 7.31 (d, *J* = 8.9 Hz, 2H), 7.10 (d, *J* = 4.5 Hz, 4H), 6.93 (d, *J* = 8.9 Hz, 2H), 6.66 (t, *J* = 4.5 Hz, 2H). ^{13}C-NMR (101 MHz, $CDCl_3$) *δ* 152.69, 144.29, 131.30, 127.12, 126.34, 121.59, 120.08, 117.33, 111.51, 108.64. HRMS (LD-TOF): m/z calcd for $C_{20}H_{10}O_2$ $[M+H]^+$: 282.0675; found: 282.0675. Spectroscopic characterization in accordance with literature.[18]

Characterization

Optimization spectra (table 1, main text)

The yield was measured by ^{1}H-NMR spectroscopy using a stock solution of 1,3,5-trimethoxybenzene (0.6 mL, 0.28 mM in CDCl3) as internal standard (IS). The peaks which were used to calculate the yield are the singlet at 8.6 ppm (H_1) and the singlet at 6.0 ppm (H_1) which was normalised. The reactions were carried out on 0.5 mmol scale of stating material and 1/3 of internal standard was used (0.17 mmol, unless specified otherwise). ^{1}H-NMR (400 MHz) were all recorded in $CDCl_3$.

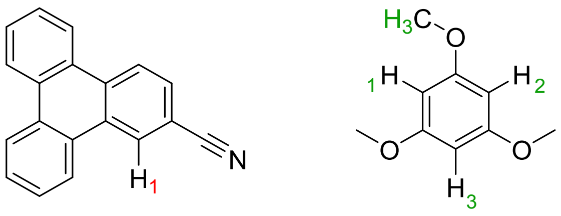

1: 0.5 mmol **IS:** 0.17 mmol

Figure S2. Proton H_1 of **1** and H_1 of IS used to calculate the yield.

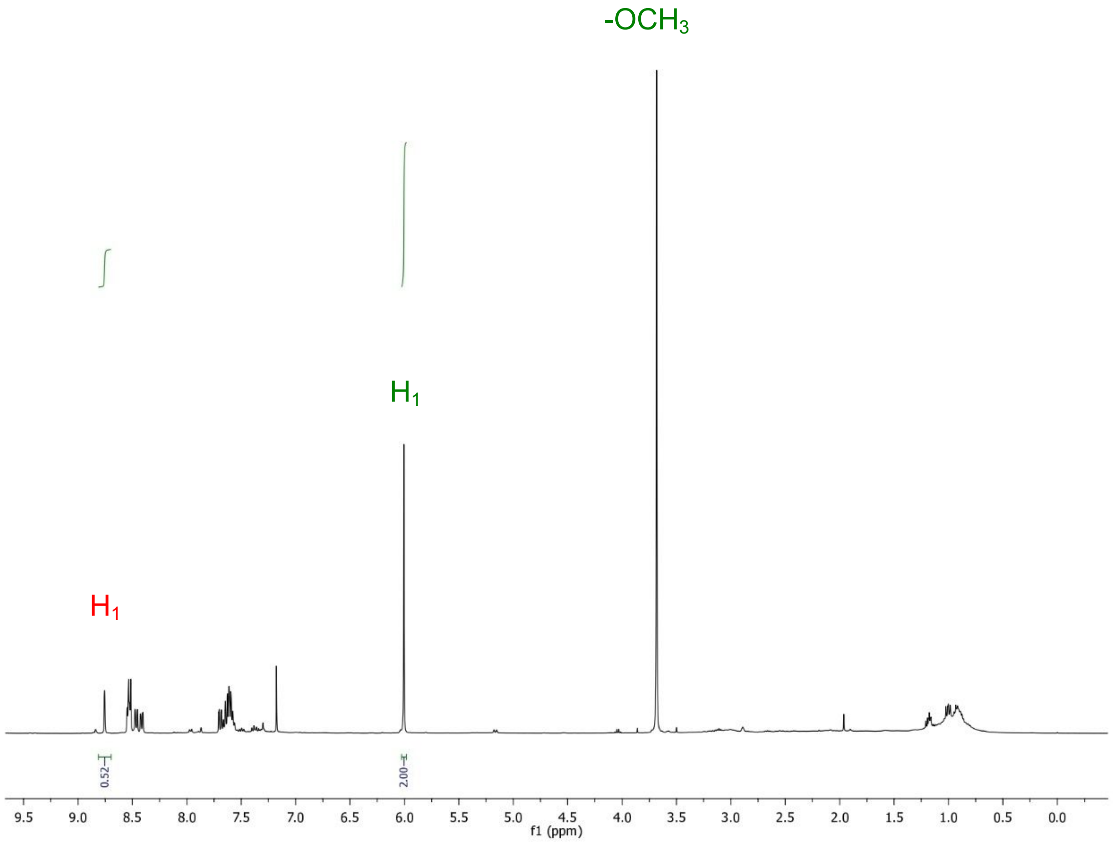

Entry 1 (0.34 mmol of IS were used) – 52% yield

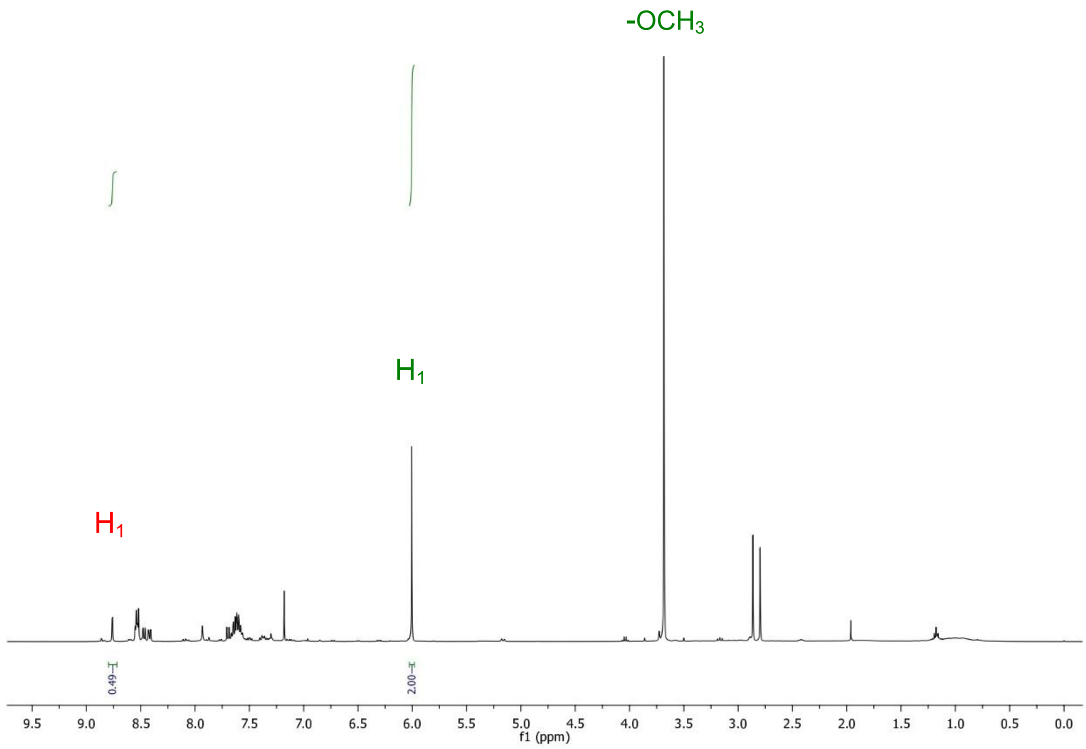

Entry 2 (0.34 mmol of IS were used) – 49% yield

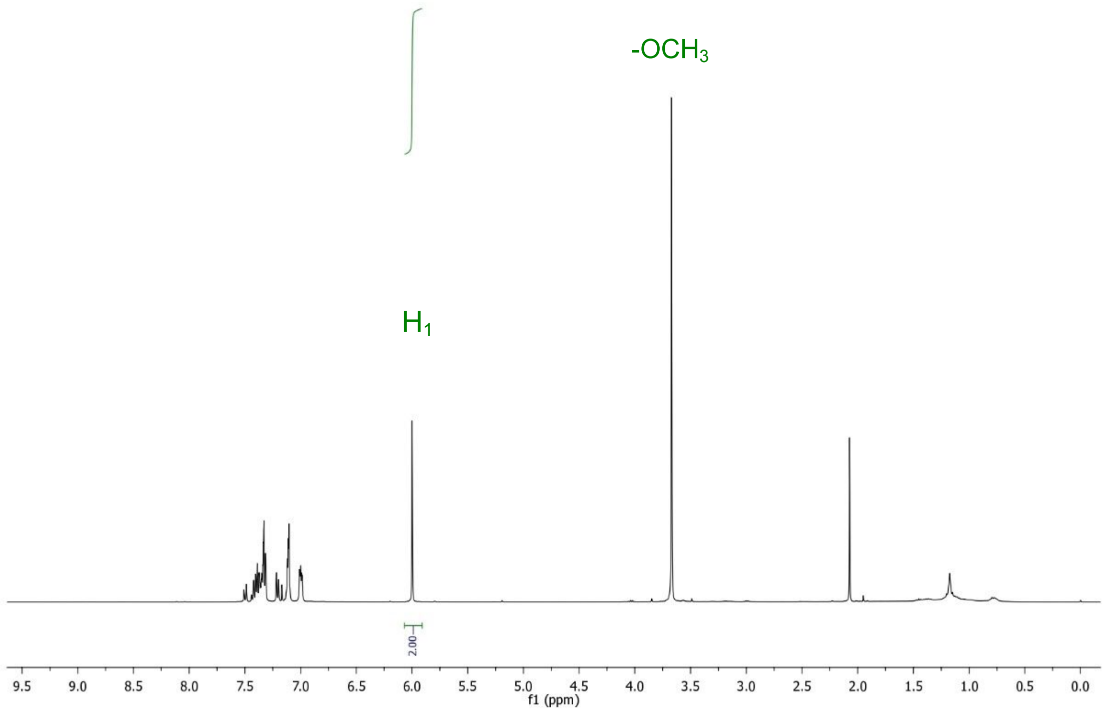

Entry 3 (0.34 mmol of IS were used) – 0% yield

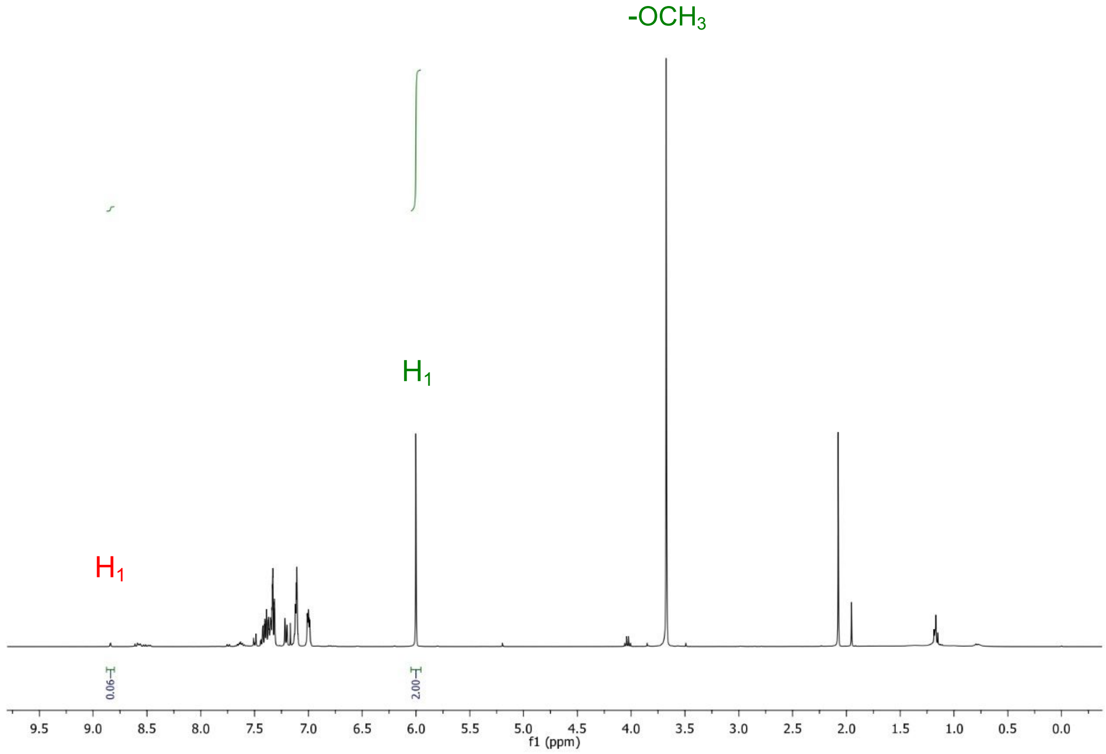

Entry 4 (0.34 mmol of IS were used) – 6% yield

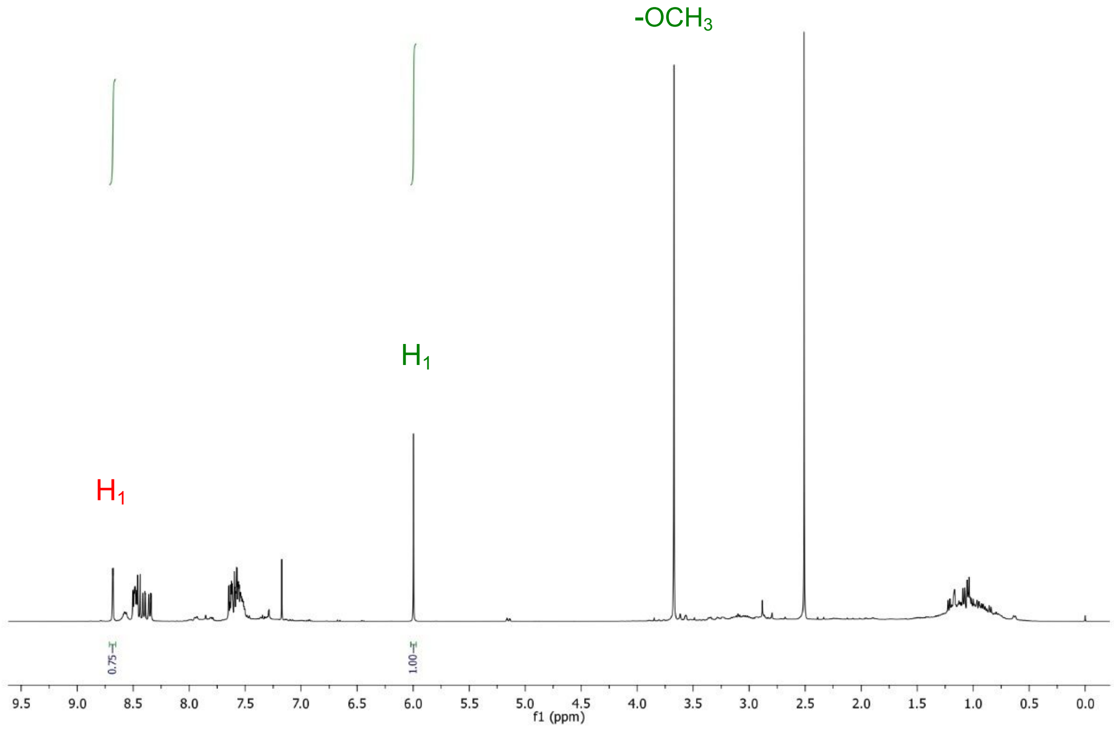

Entry 5 – 75% yield

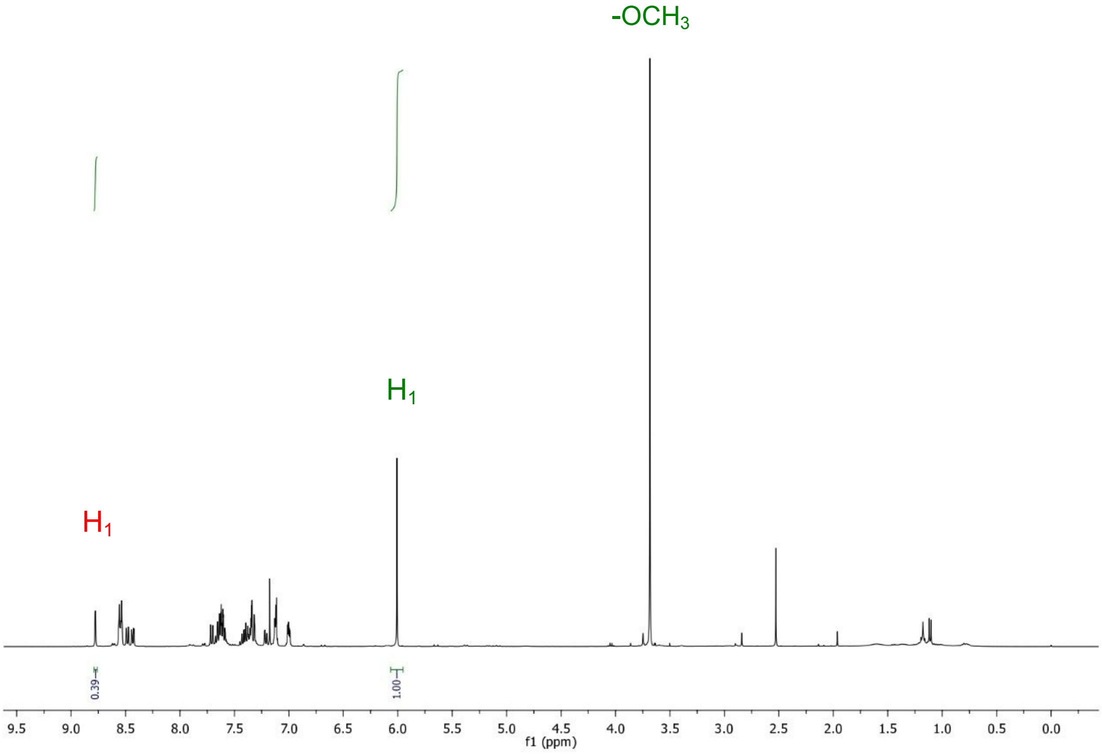

Entry 6 – 39% yield

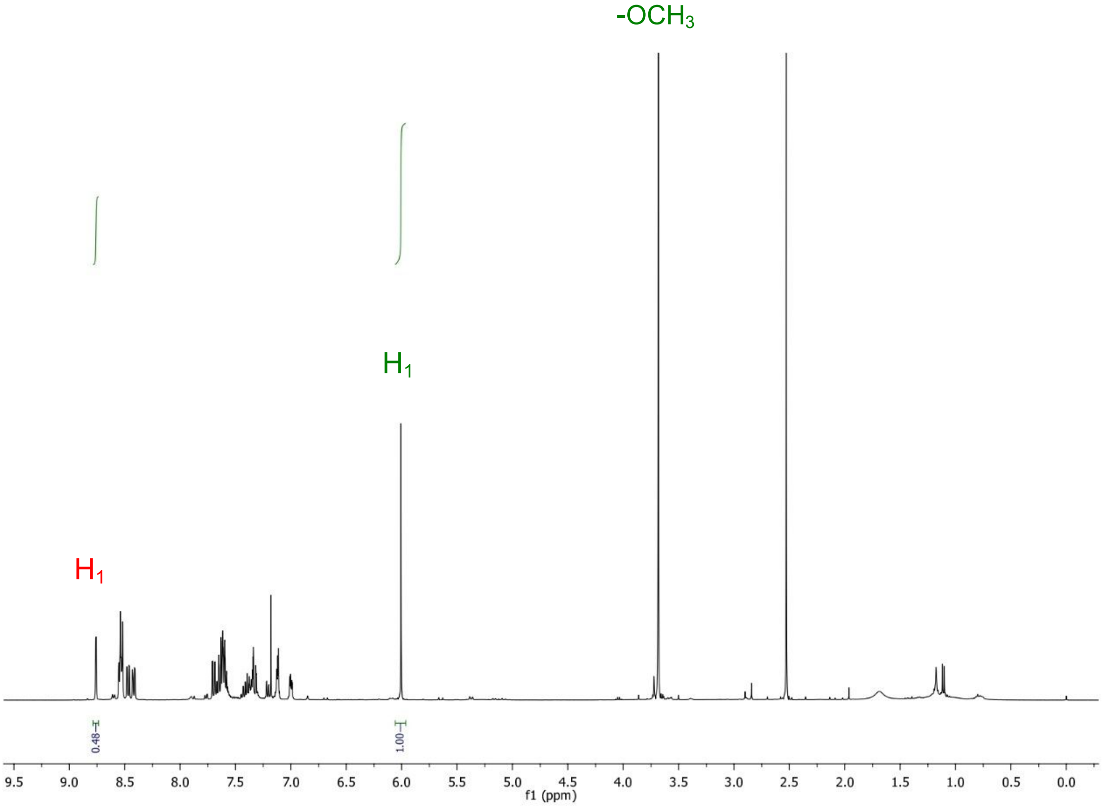

Entry 7 – 48% yield

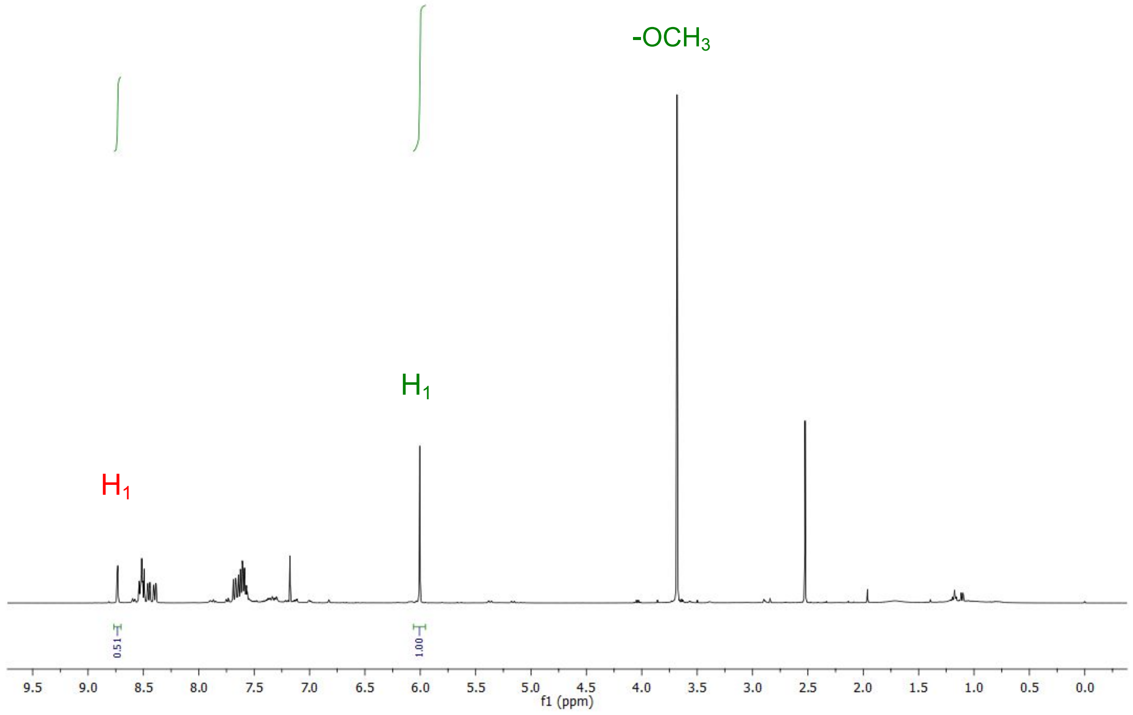

Entry 8 – 51% yield

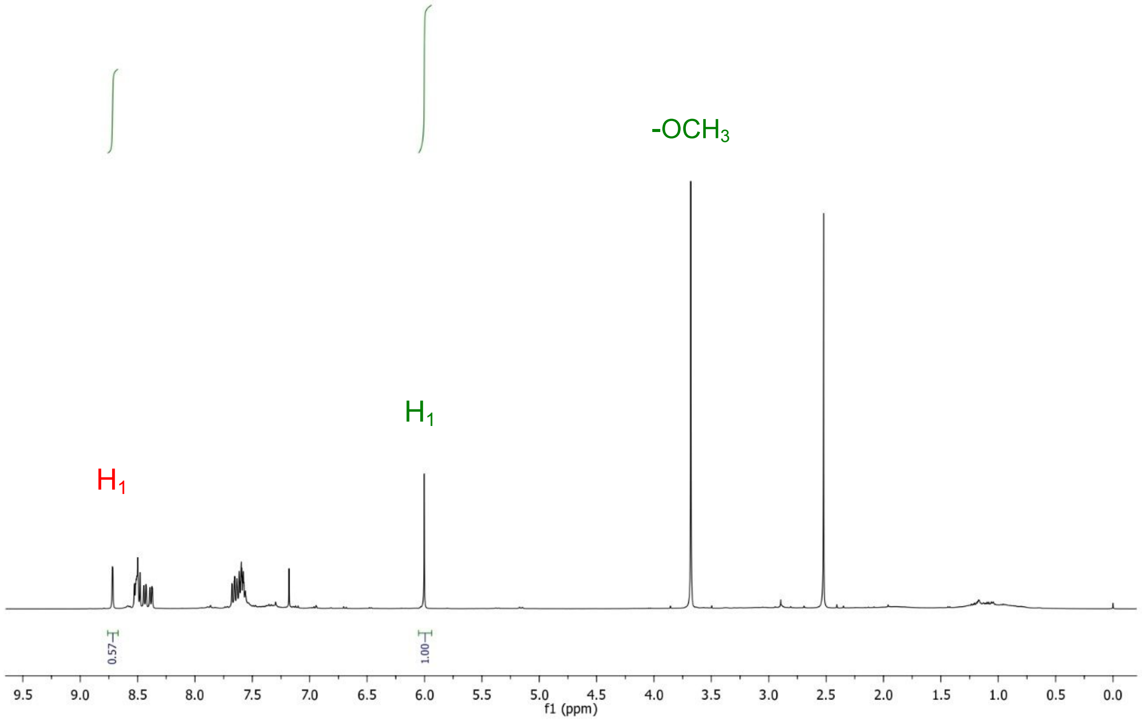

Entry 9 – 57% yield

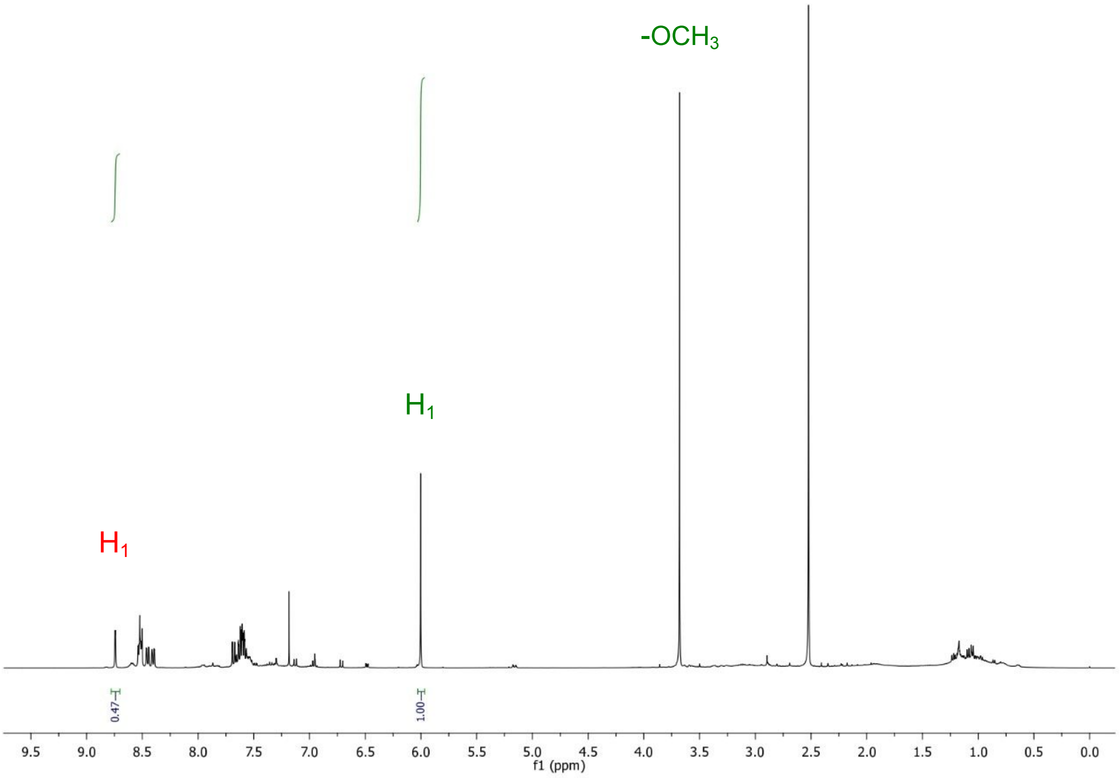

Entry 10 – 47% yield

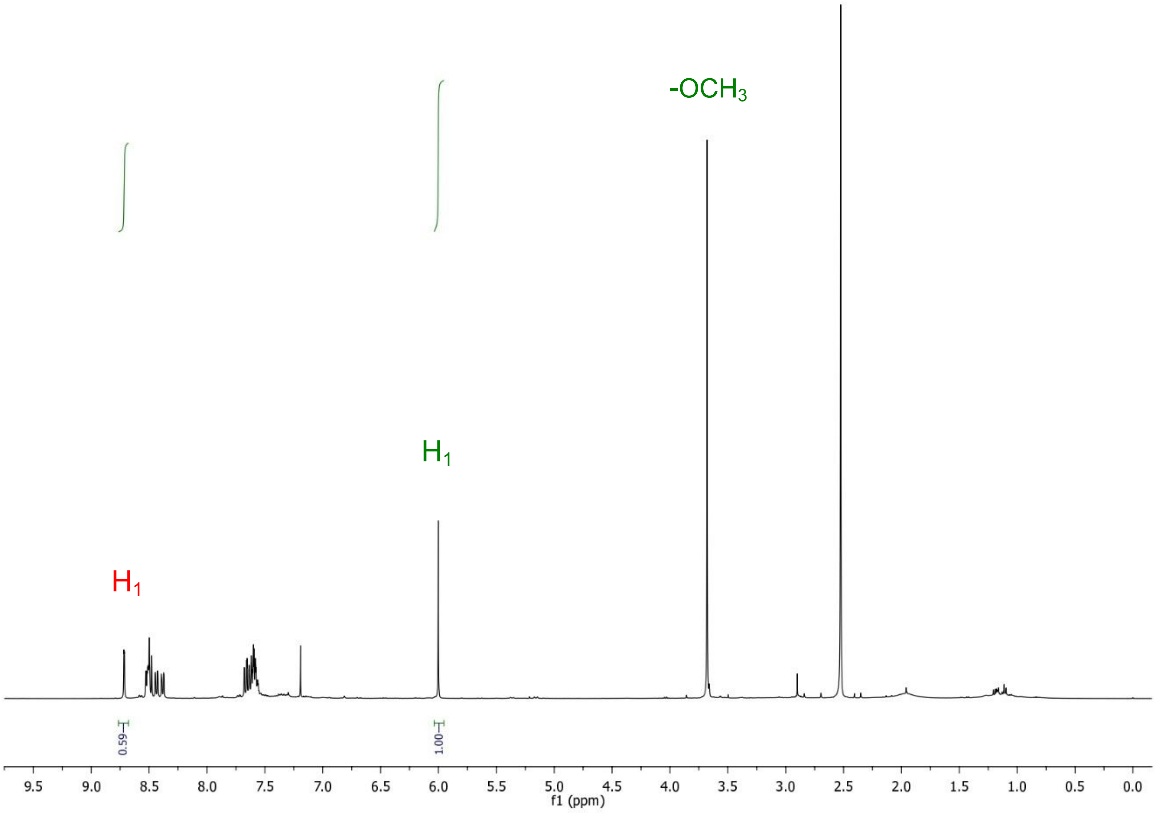

Entry 11 – 59% yield

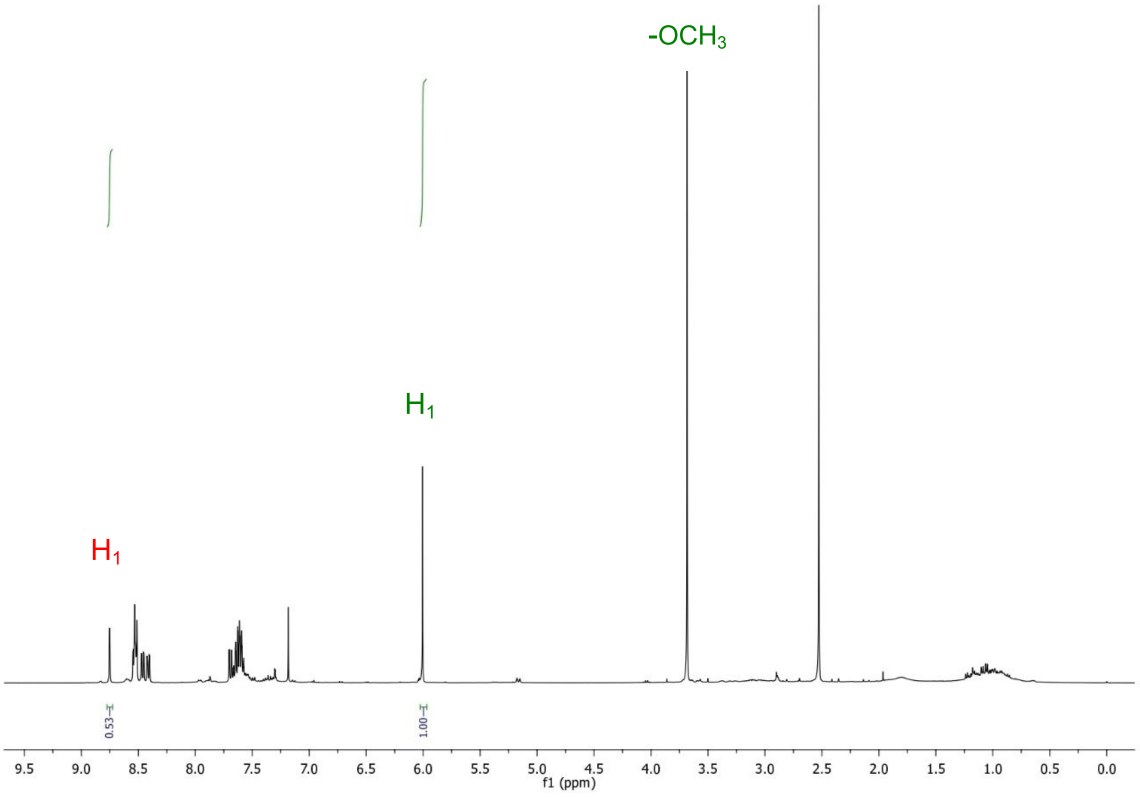

Entry 12 – 53% yield

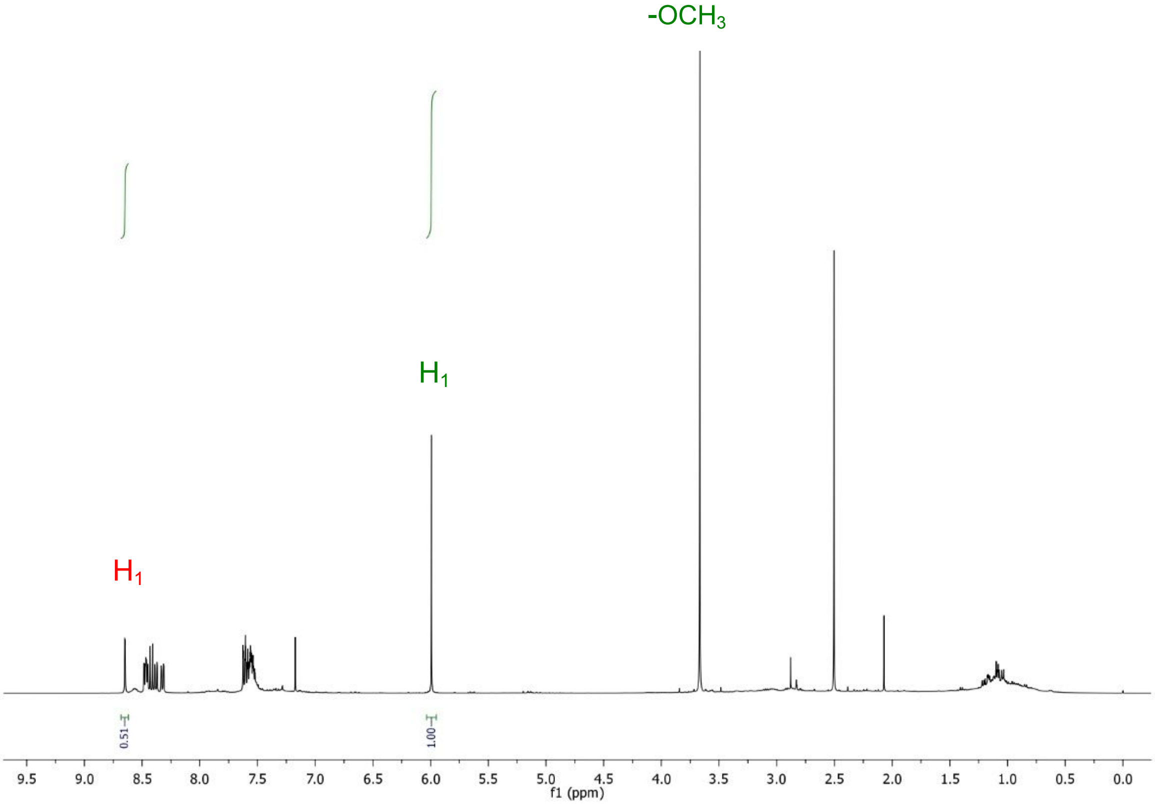

Entry 13 – 51% yield

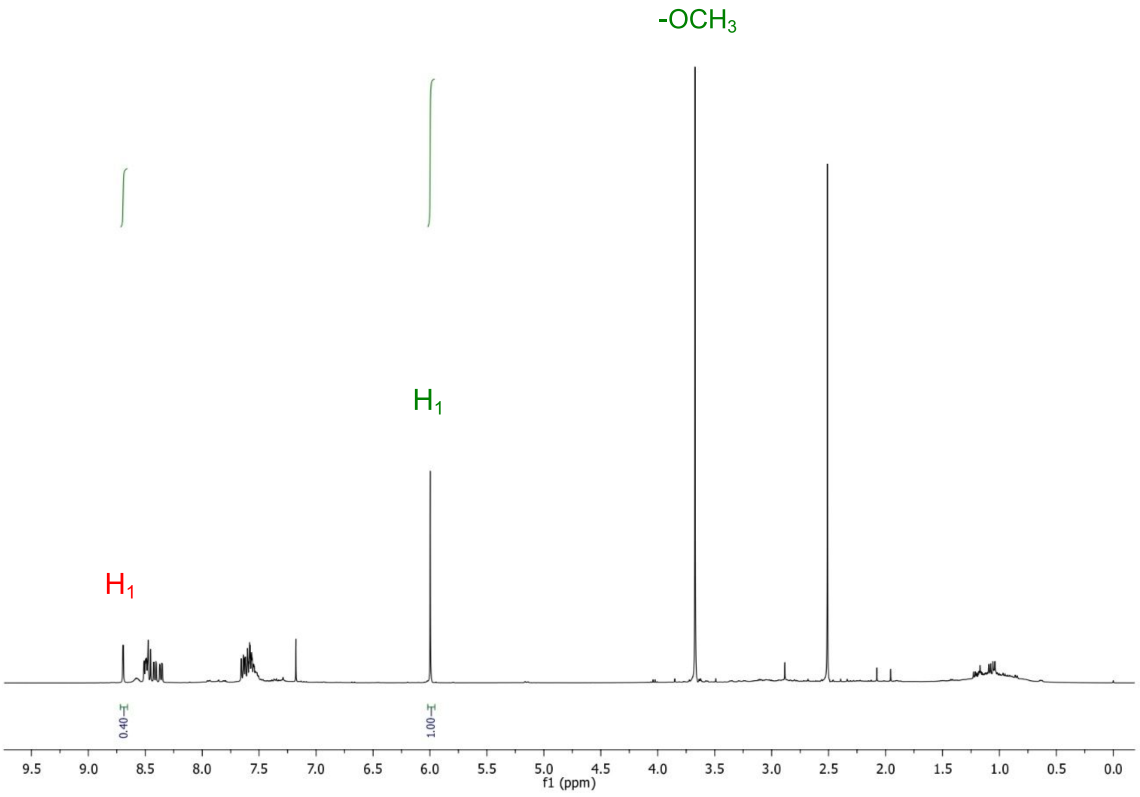

Entry 14 – 40% yield

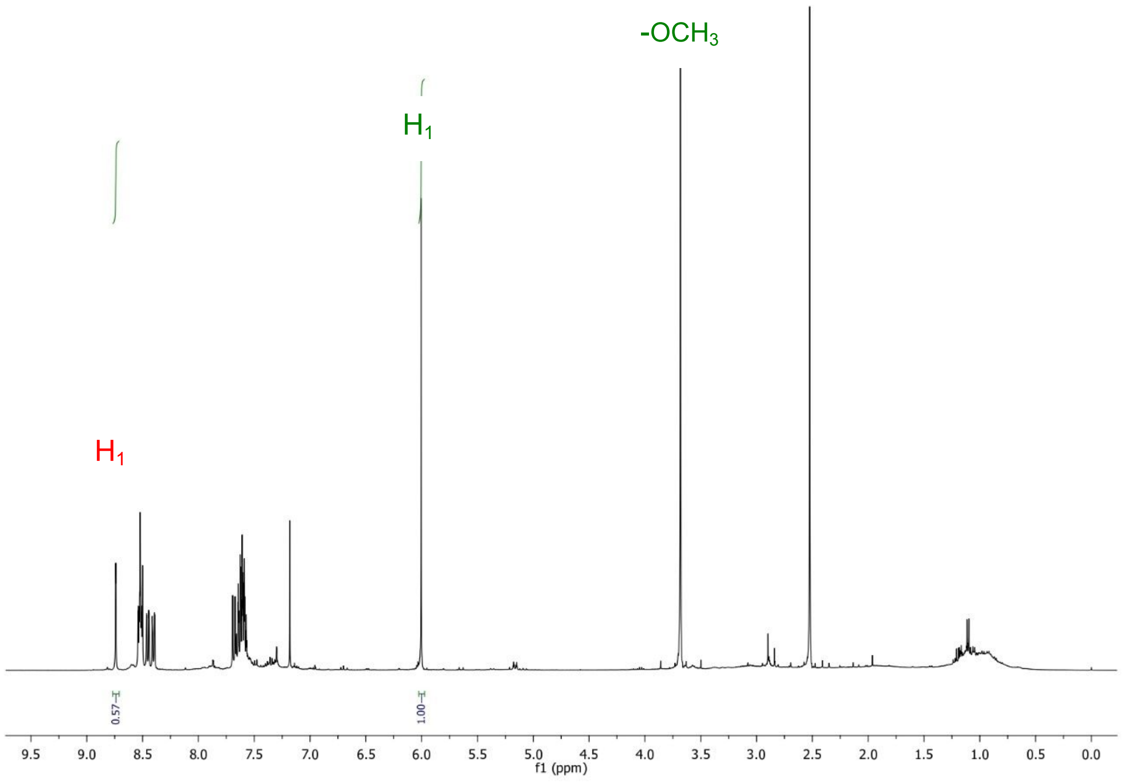

Entry 15 – 57% yield

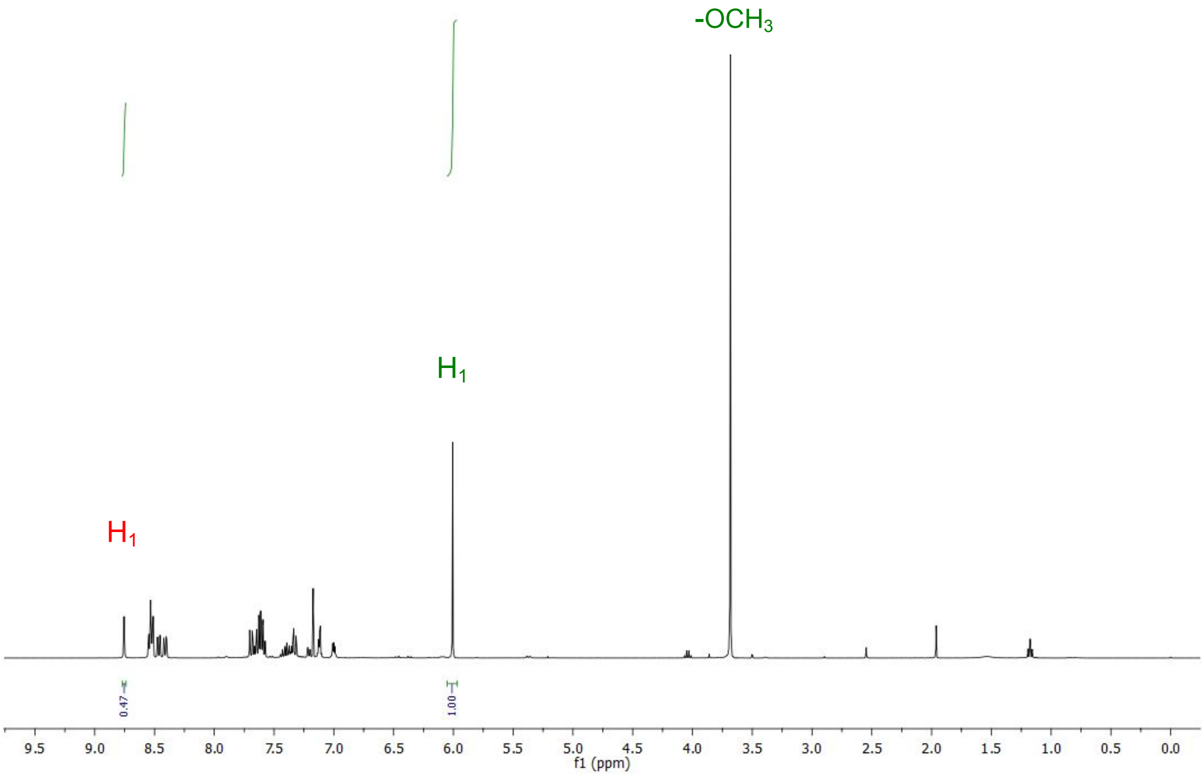

Entry 16 – 47% yield

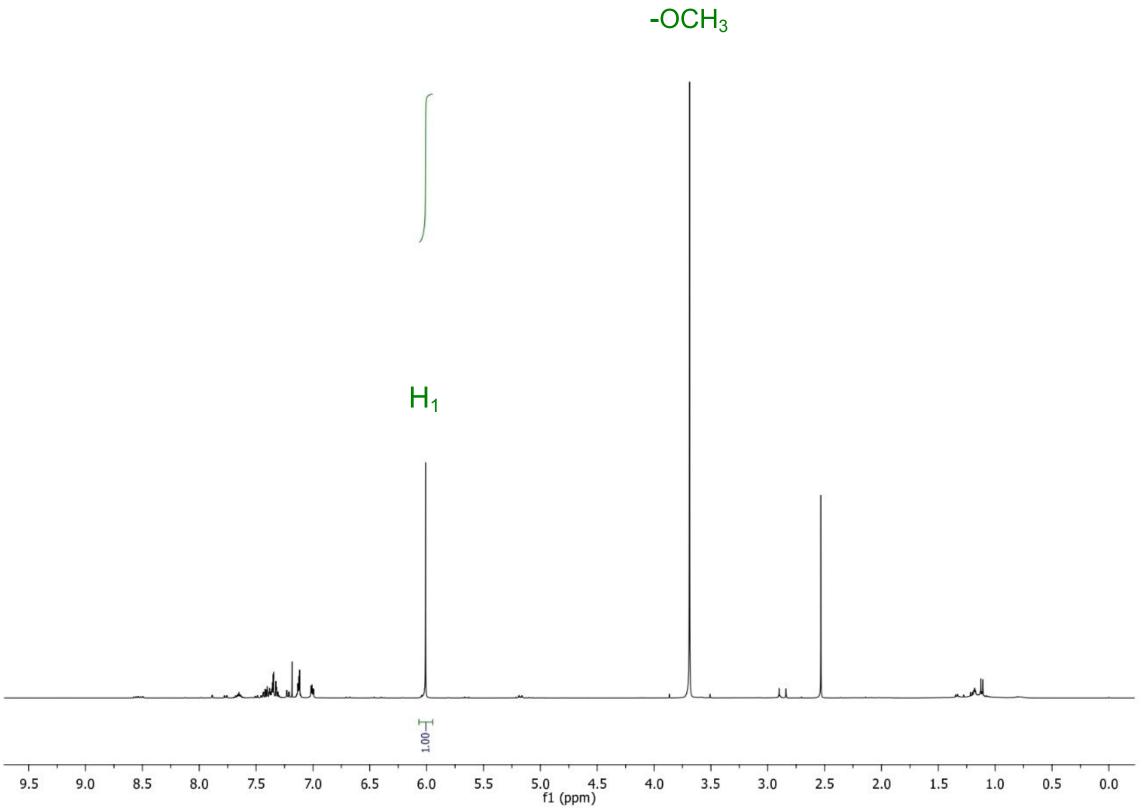

Entry 17 – 0% yield

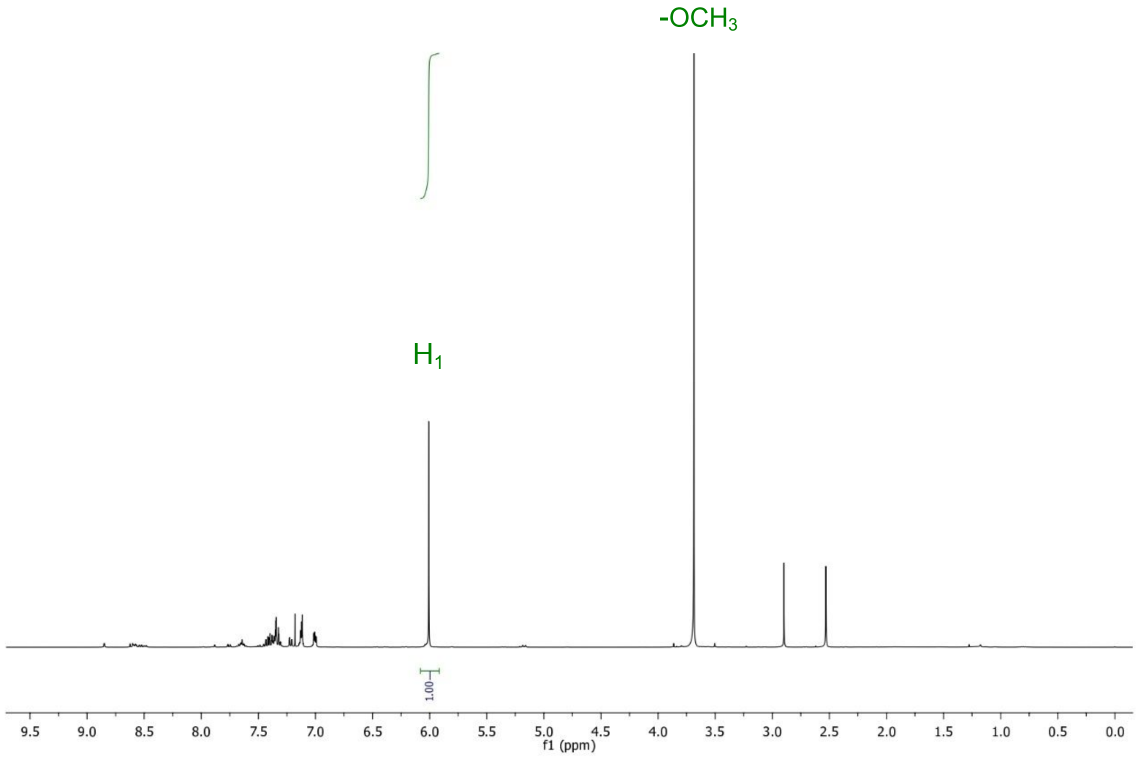

Entry 18 – 0% yield

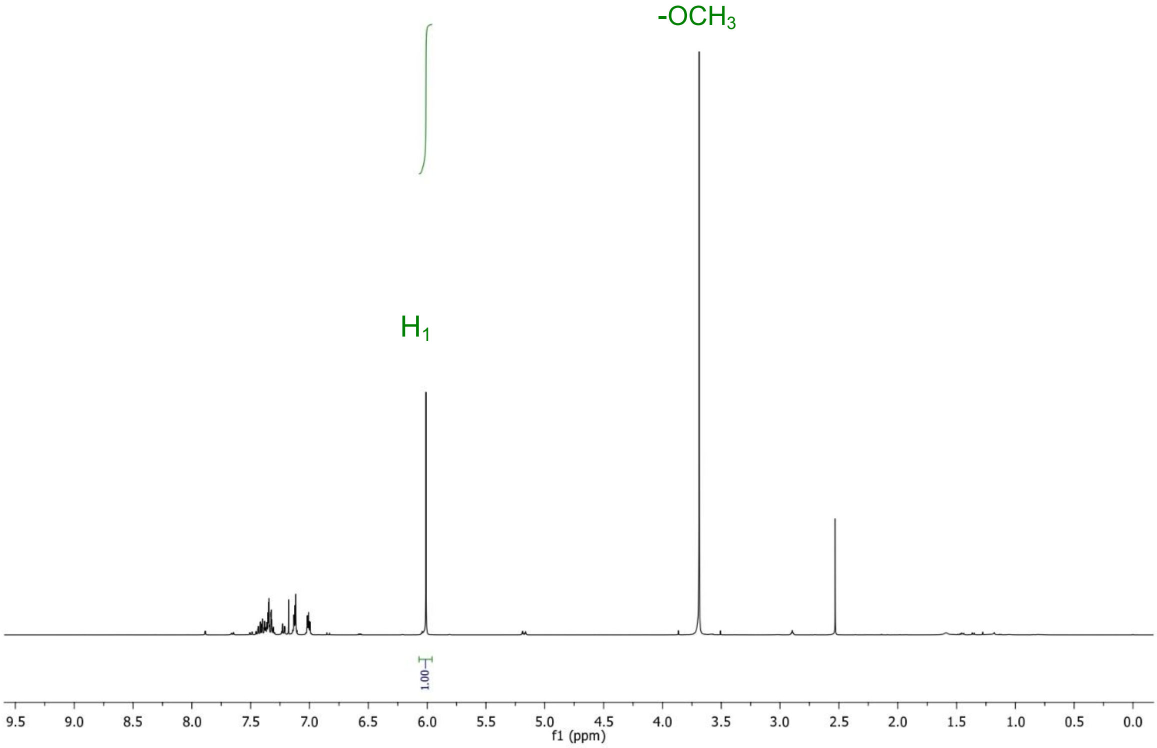

Entry 19 – 0% yield

-OCH$_3$

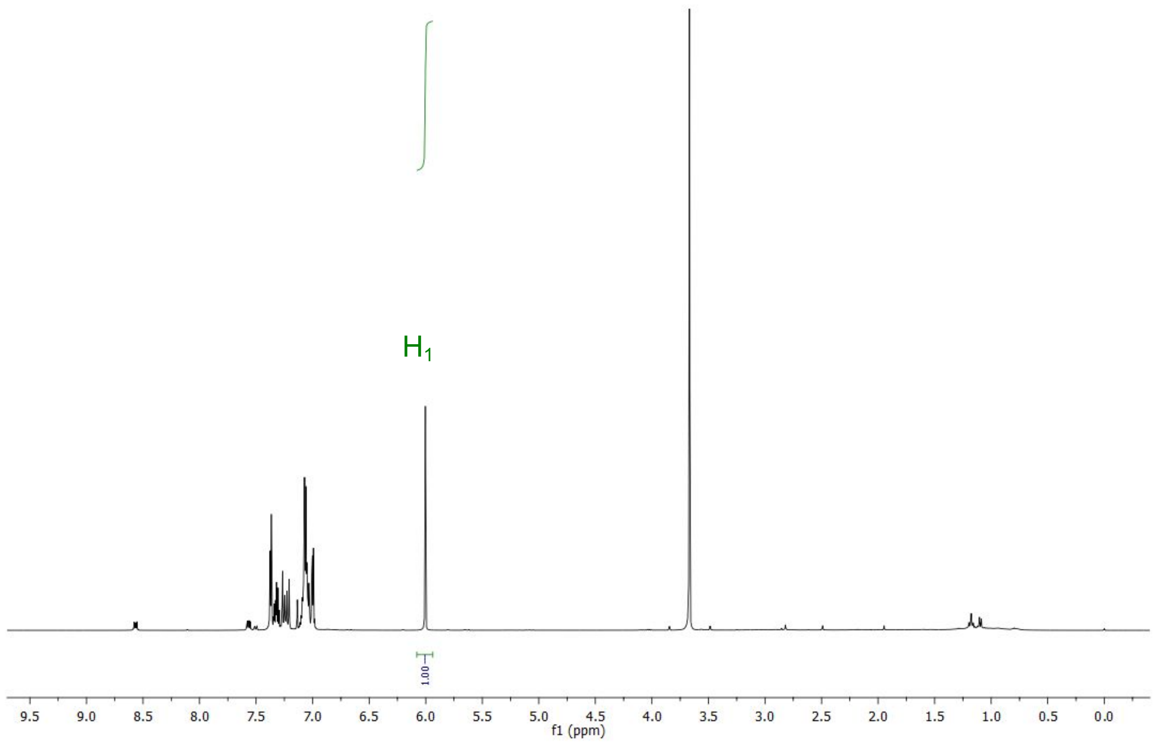

Entry 20 – 0% yield

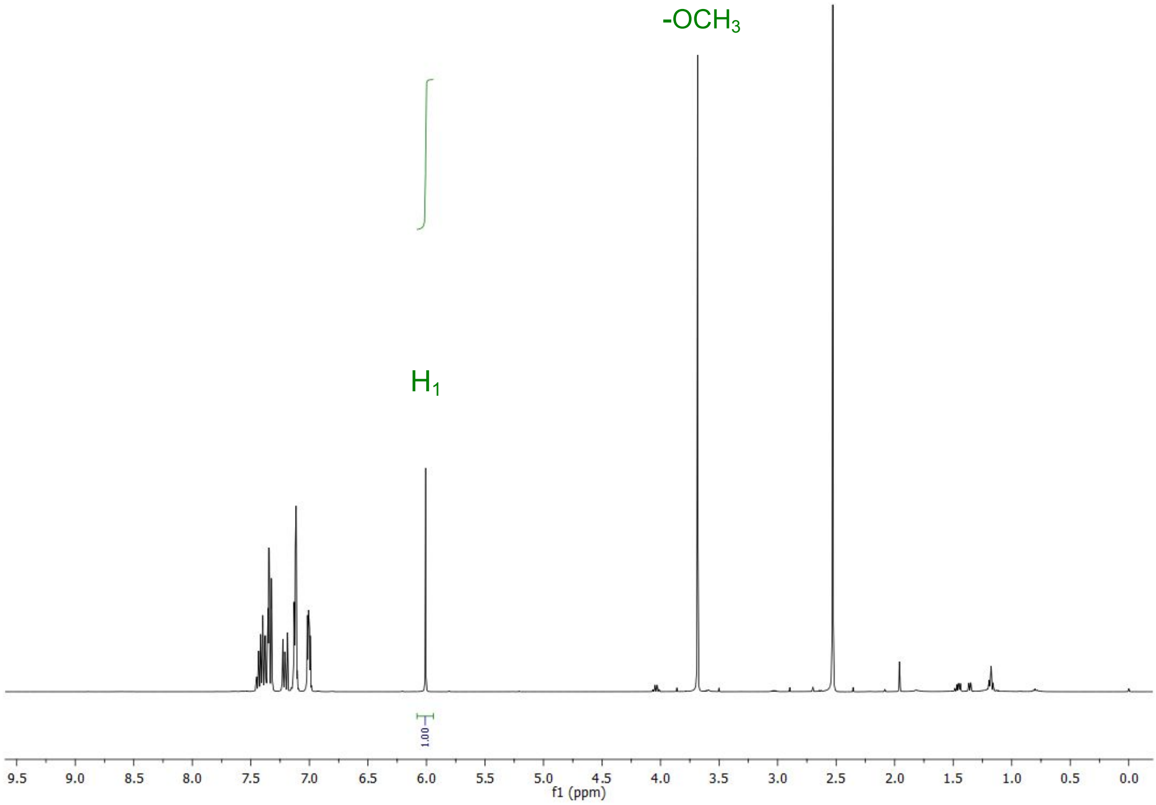

Entry 21 – 0% yield

Characterization of clean compounds

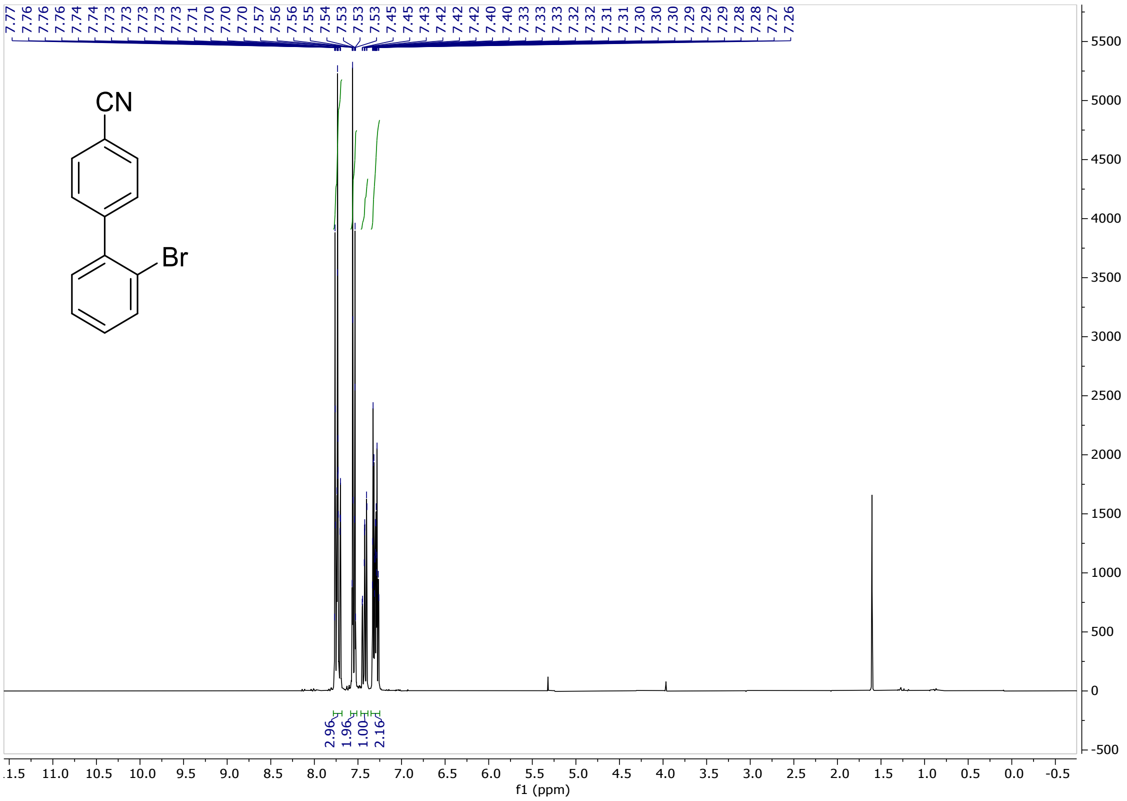

Figure S3. ^{1}H-NMR (300 MHz) spectrum of **2'-bromo-[1,1'-biphenyl]-4-carbonitrile** in $CDCl_3$.

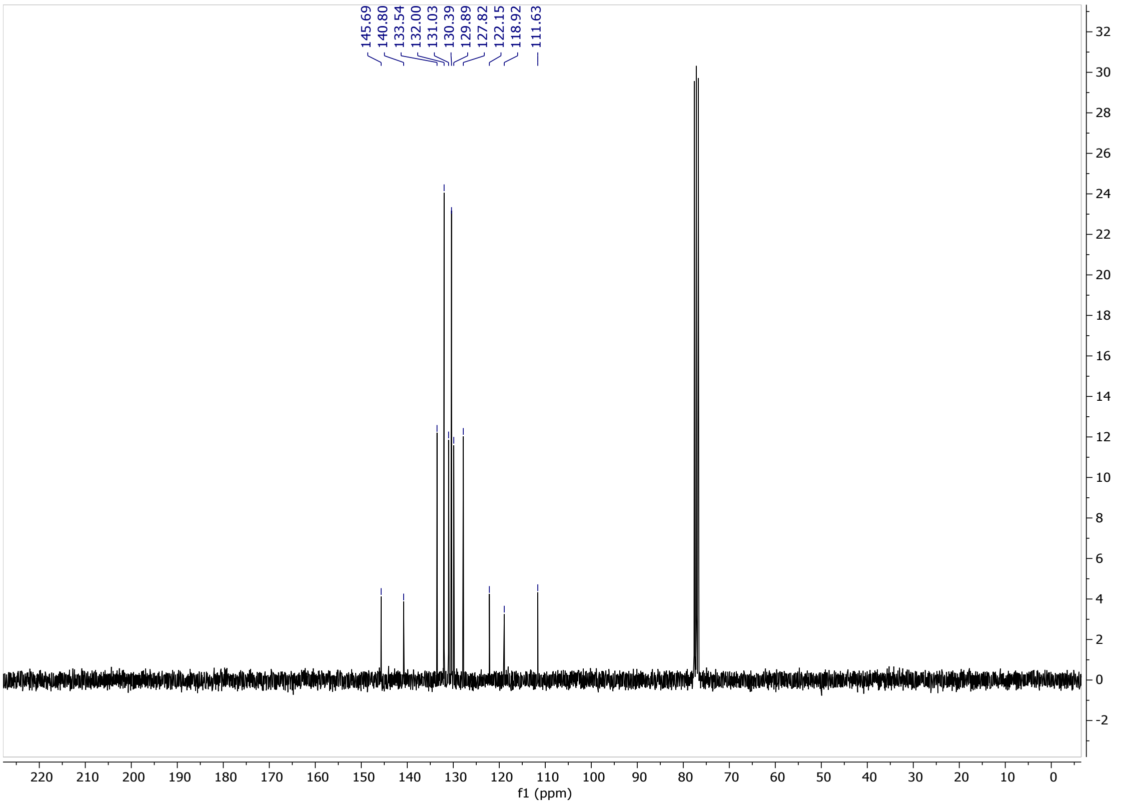

Figure S4. ^{13}C-NMR (75 MHz) spectrum of **2'-bromo-[1,1'-biphenyl]-4-carbonitrile** in $CDCl_3$.

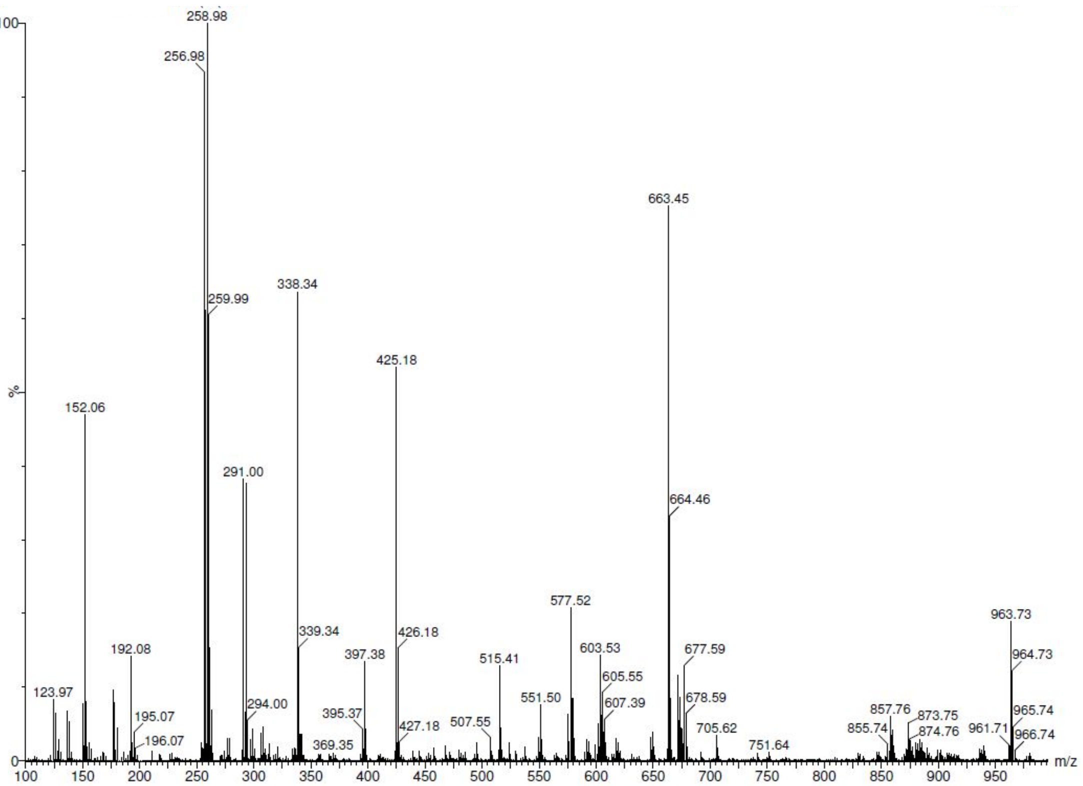

Figure S5. LRMS-APCI TOF mass spectrum of **2'-bromo-[1,1'-biphenyl]-4-carbonitrile**.

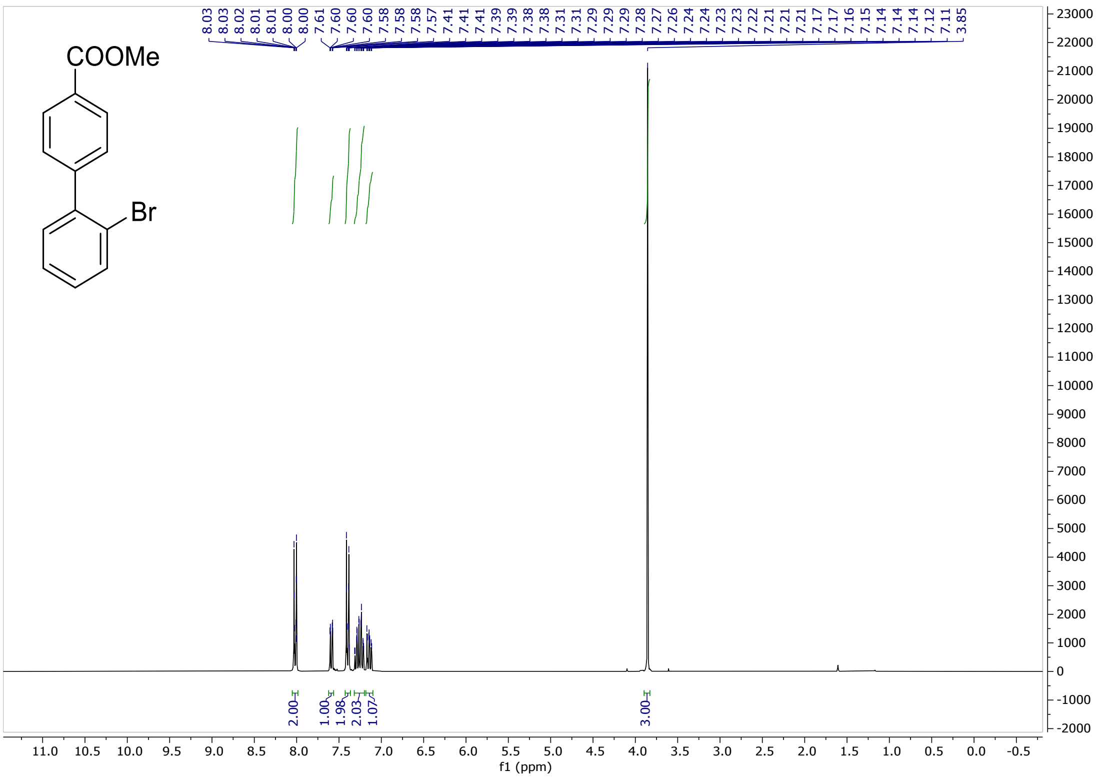

Figure S6. ^{1}H-NMR (300 MHz) spectrum of **2'-bromo-[1,1'-biphenyl]-4-carboxylate** in $CDCl_3$.

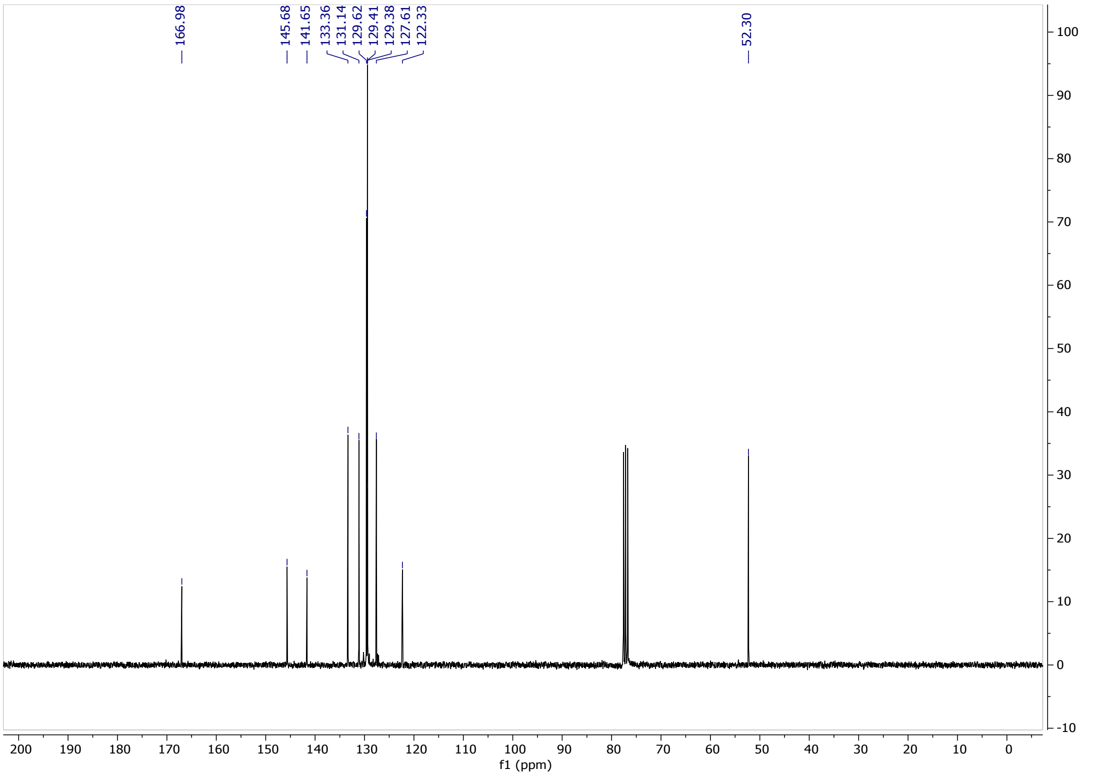

Figure S7. ^{13}C-NMR (75 MHz) spectrum of **2'-bromo-[1,1'-biphenyl]-4-carboxylate** in $CDCl_3$.

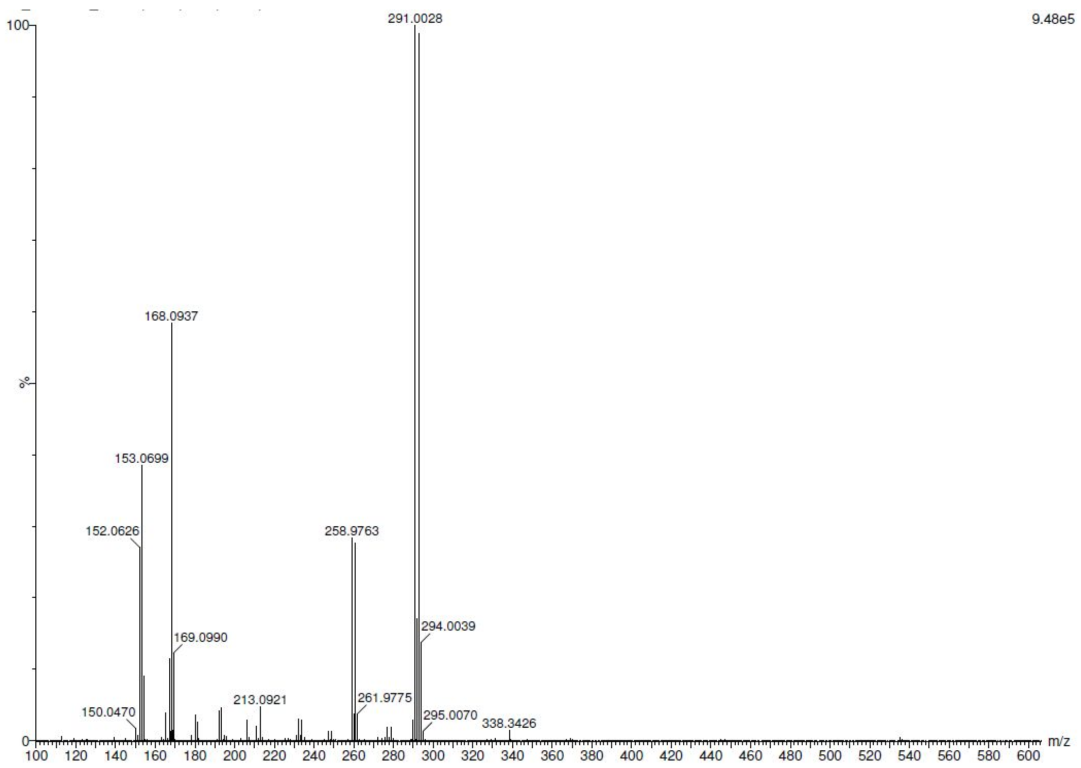

Figure S8. HRMS-APCI TOF mass spectrum of **2'-bromo-[1,1'-biphenyl]-4-carboxylate**.

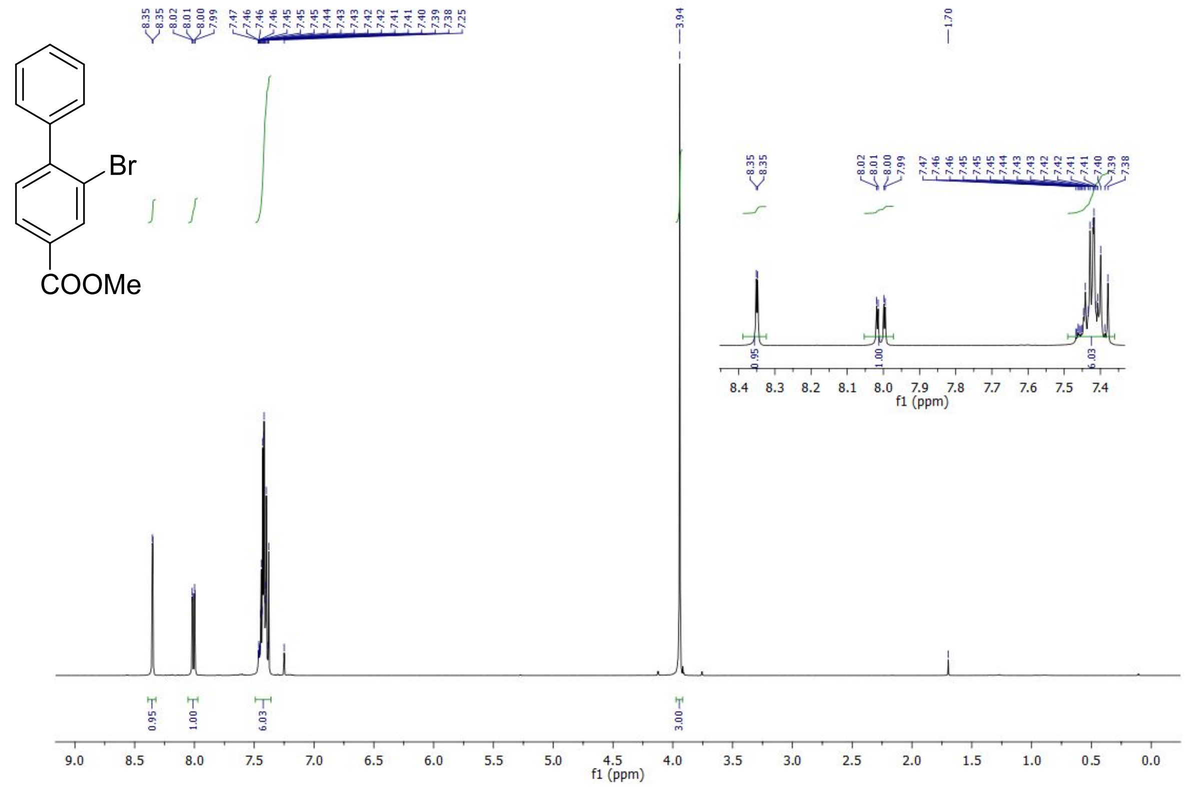

Figure S9. ^{1}H-NMR (700 MHz) spectrum of **methyl 2-bromo-[1,1'-biphenyl]-4-carboxylate** in $CDCl_3$.

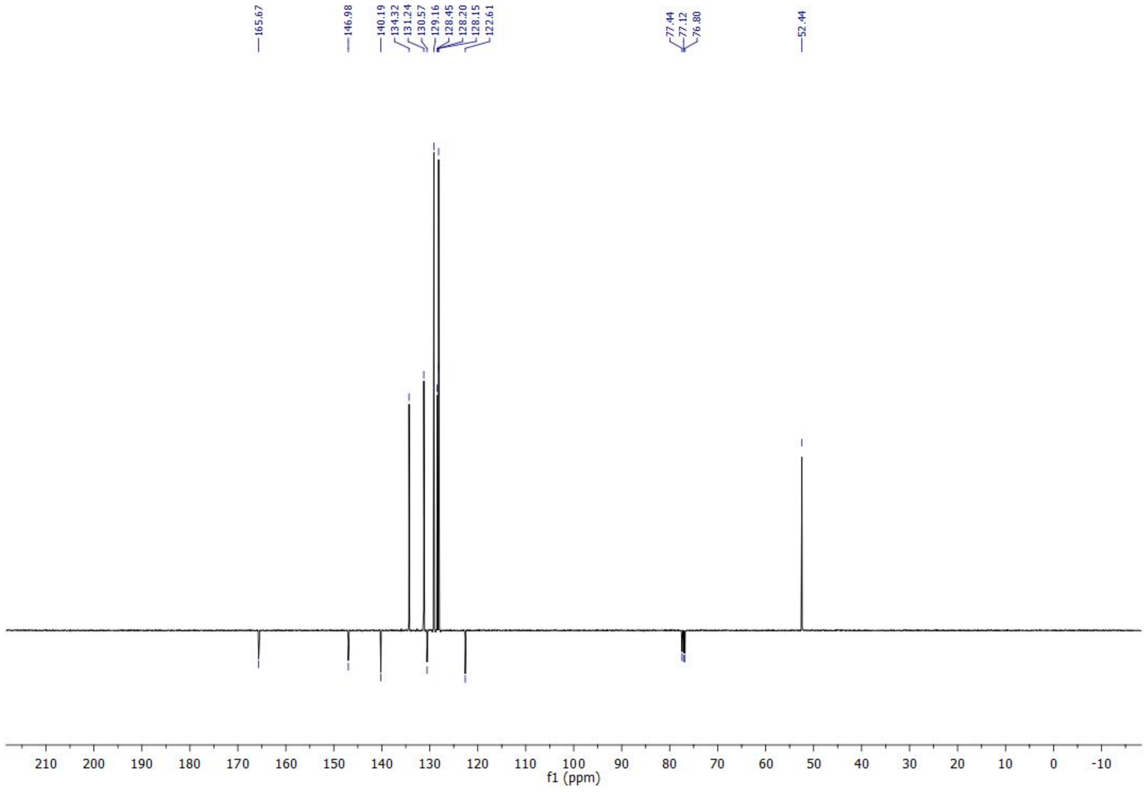

Figure S10. ^{13}C-NMR (176 MHz) spectrum of **methyl 2-bromo-[1,1'-biphenyl]-4-carboxylate** in $CDCl_3$**.**

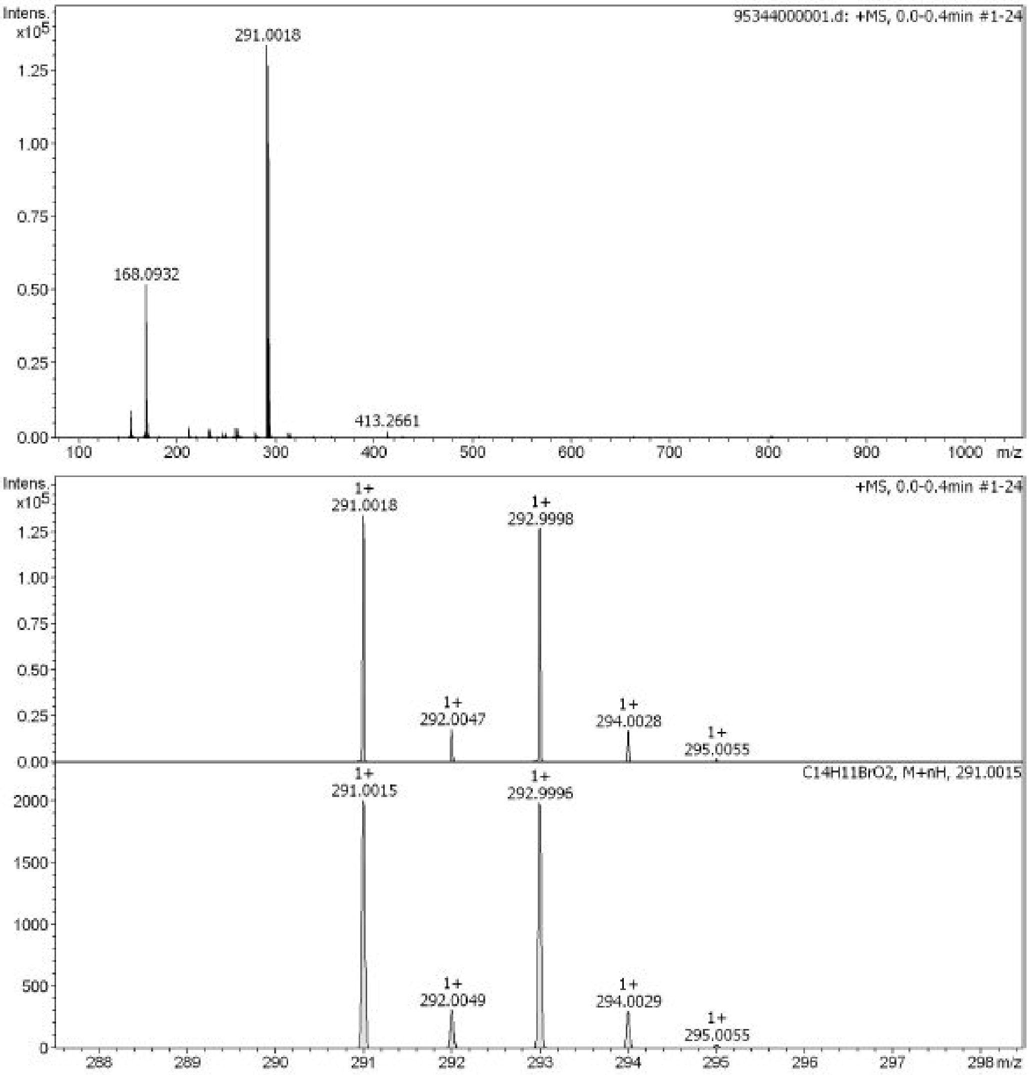

Figure S11. HRMS-ESI mass spectrum of **methyl 2-bromo-[1,1'-biphenyl]-4-carboxylate.**

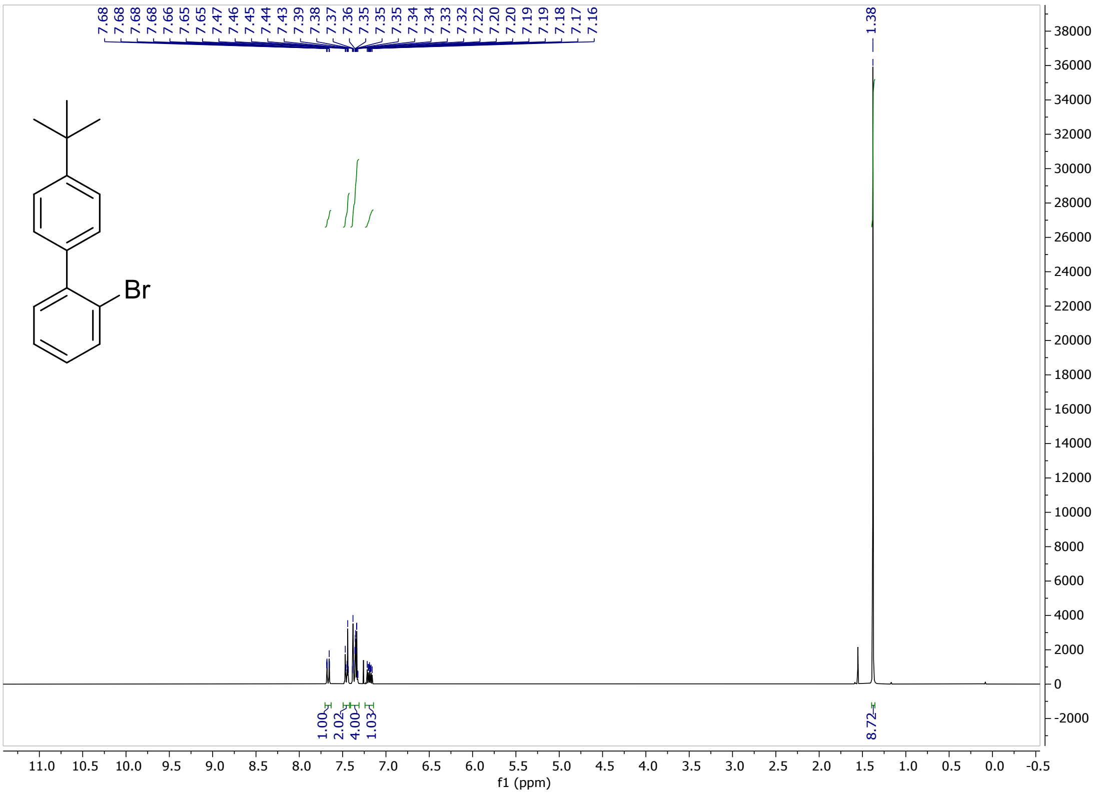

Figure S12. ^{1}H-NMR (300 MHz) spectrum of **2-bromo-4'-(tert-butyl)-1,1'-biphenyl** in $CDCl_3$.

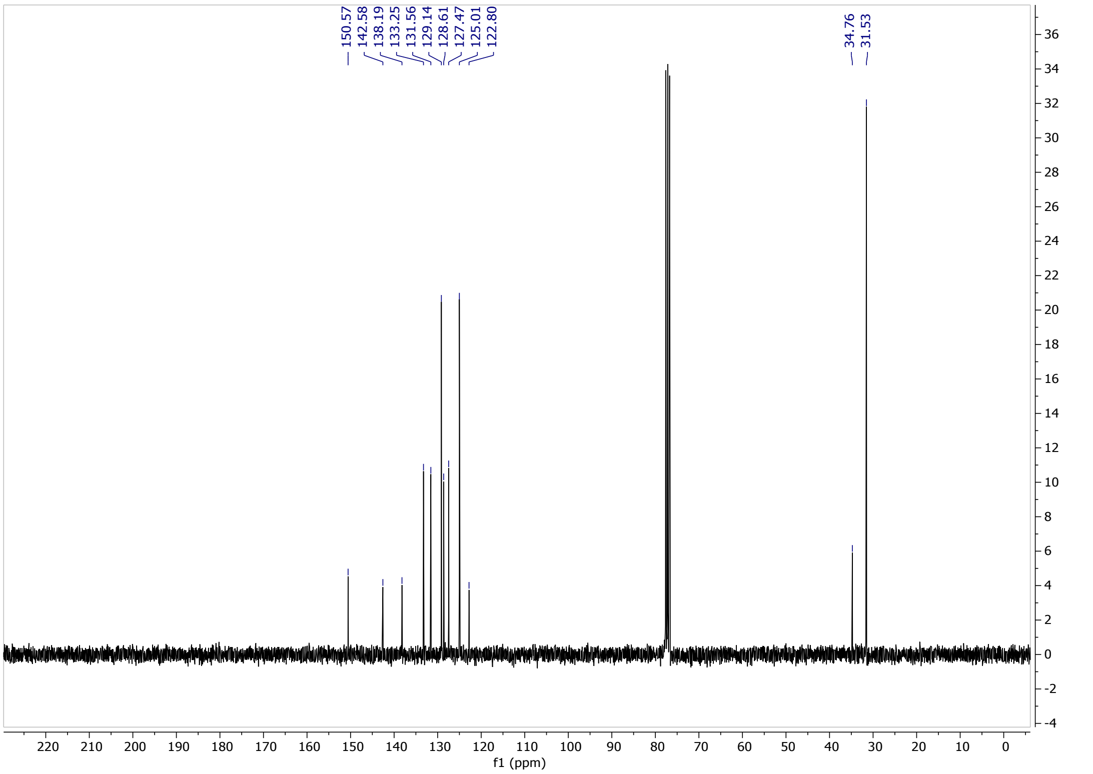

Figure S13. ^{13}C-NMR (75 MHz) spectrum of **2-bromo-4'-(tert-butyl)-1,1'-biphenyl** in $CDCl_3$.

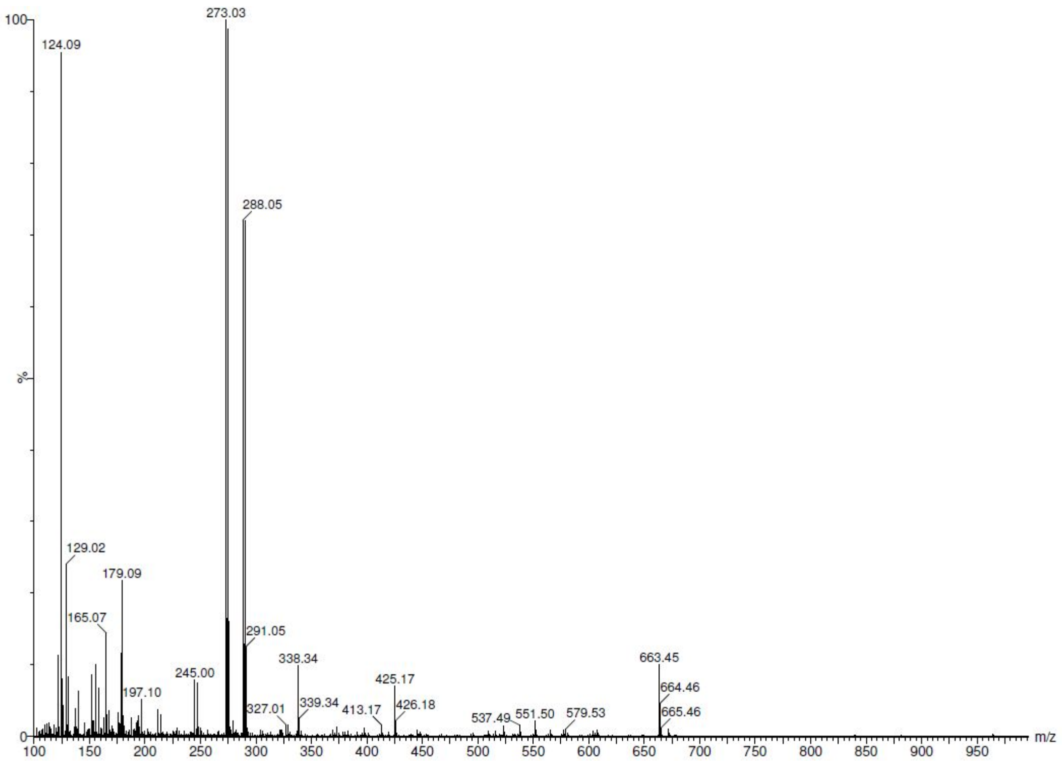

Figure S14. LRMS-APCI TOF mass spectrum of **2-bromo-4'-(tert-butyl)-1,1'-biphenyl.**

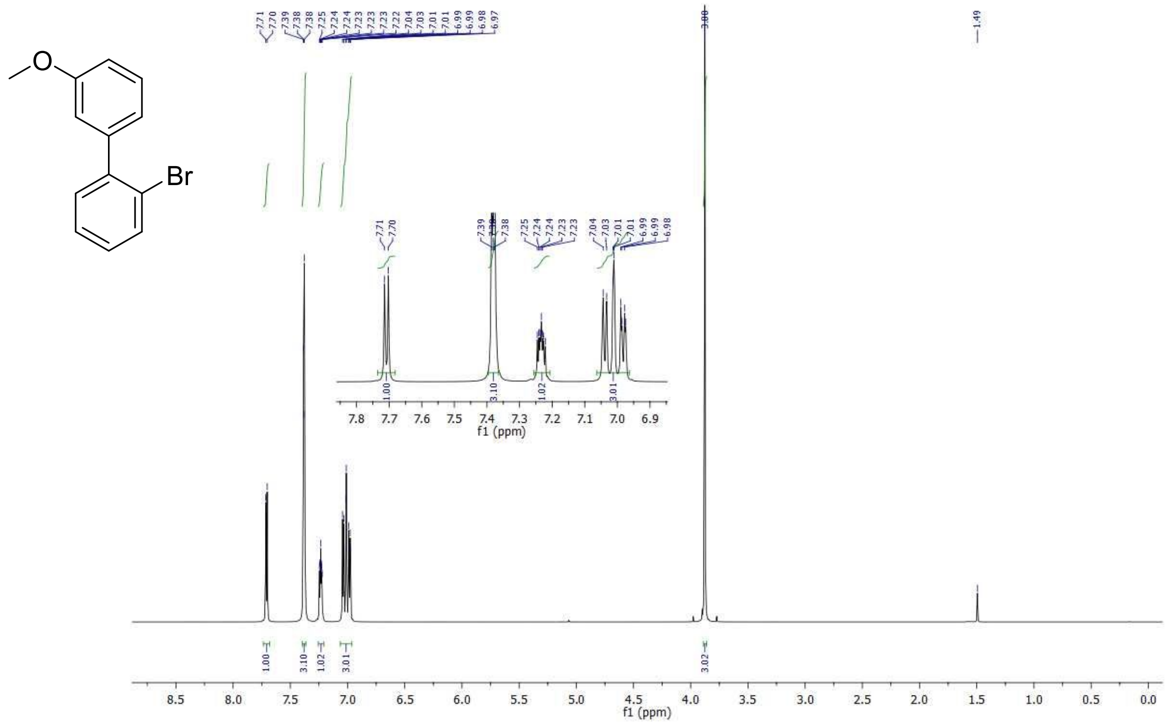

Figure S15. ^{1}H-NMR (700 MHz) spectrum of **2-bromo-3'-methoxy-1,1'-biphenyl** in $CDCl_3$.

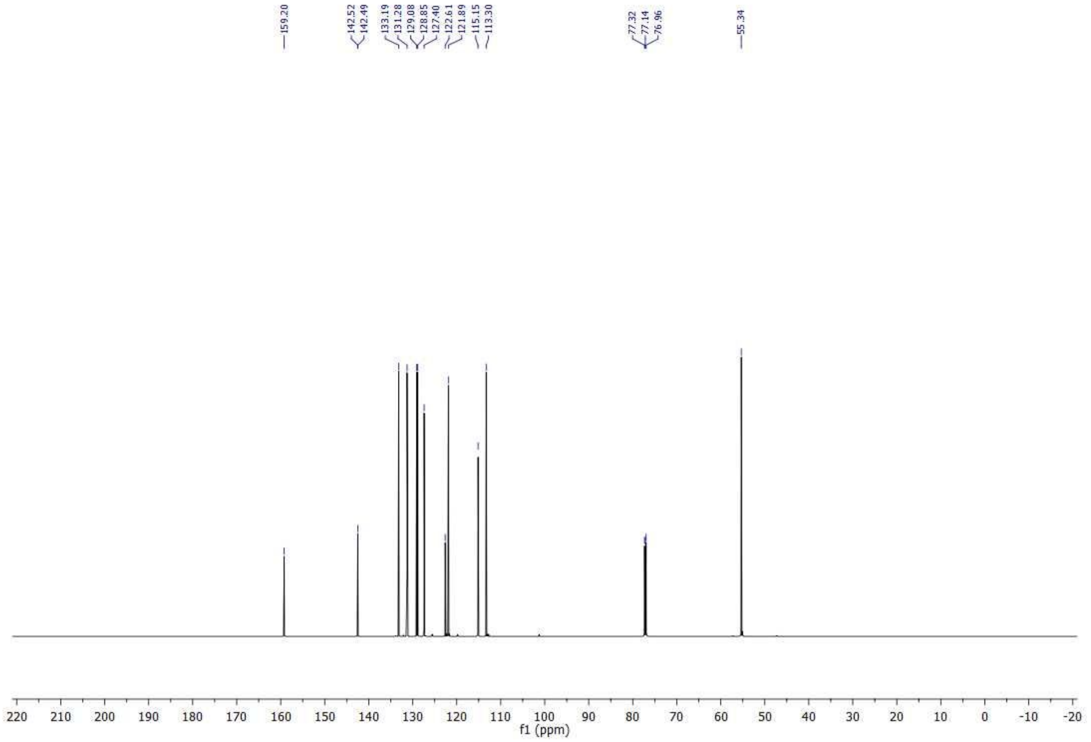

Figure S16. ^{13}C-NMR (176 MHz) spectrum of **2-bromo-3'-methoxy-1,1'-biphenyl** in $CDCl_3$.

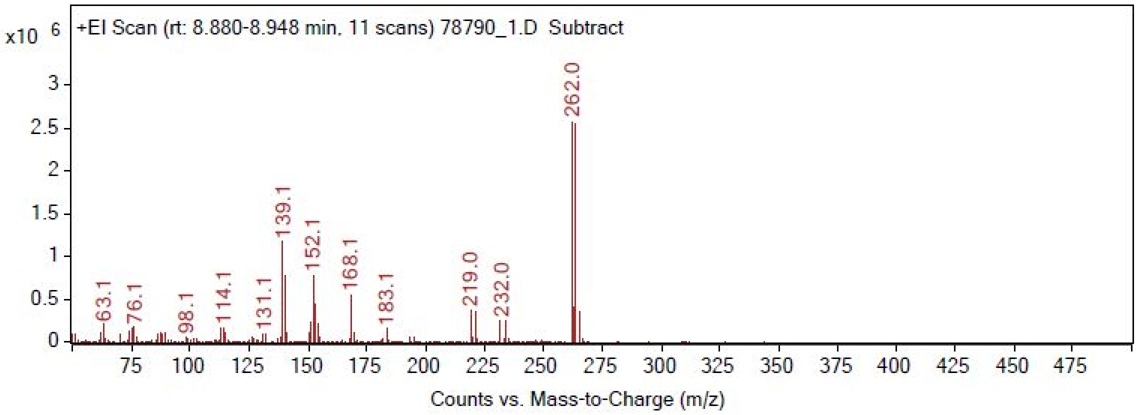

Peak List

m/z	z	Abund
139.1		1164528
140.1	1	760684.06
152.1		769969.44
153.1		439054.66
168.1	1	544277.69
219	1	358152.84
221	1	350645.91
262	1	2577987.75
263.1	1	407560.78
264	1	2551976.25

Figure S17. LRGC-MS mass spectrum of **2-bromo-3'-methoxy-1,1'-biphenyl**.

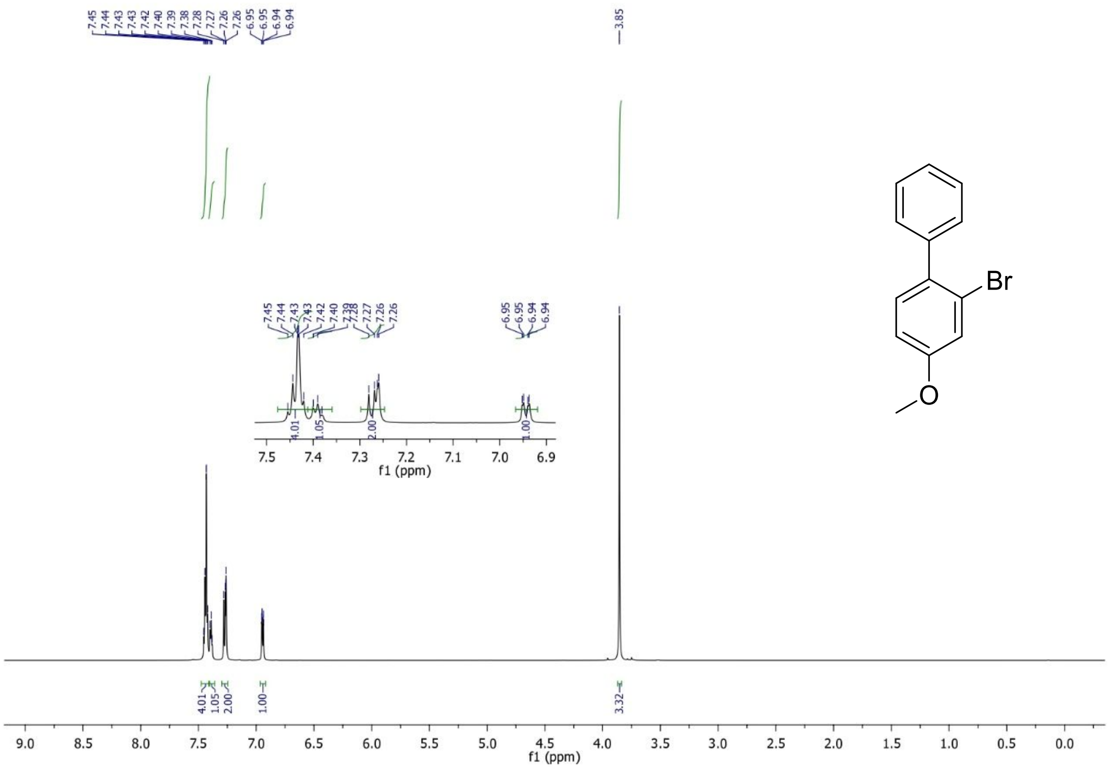

Figure S18. ^{1}H-NMR (700 MHz) spectrum of **2-bromo-4-methoxy-1,1'-biphenyl** in $CDCl_3$.

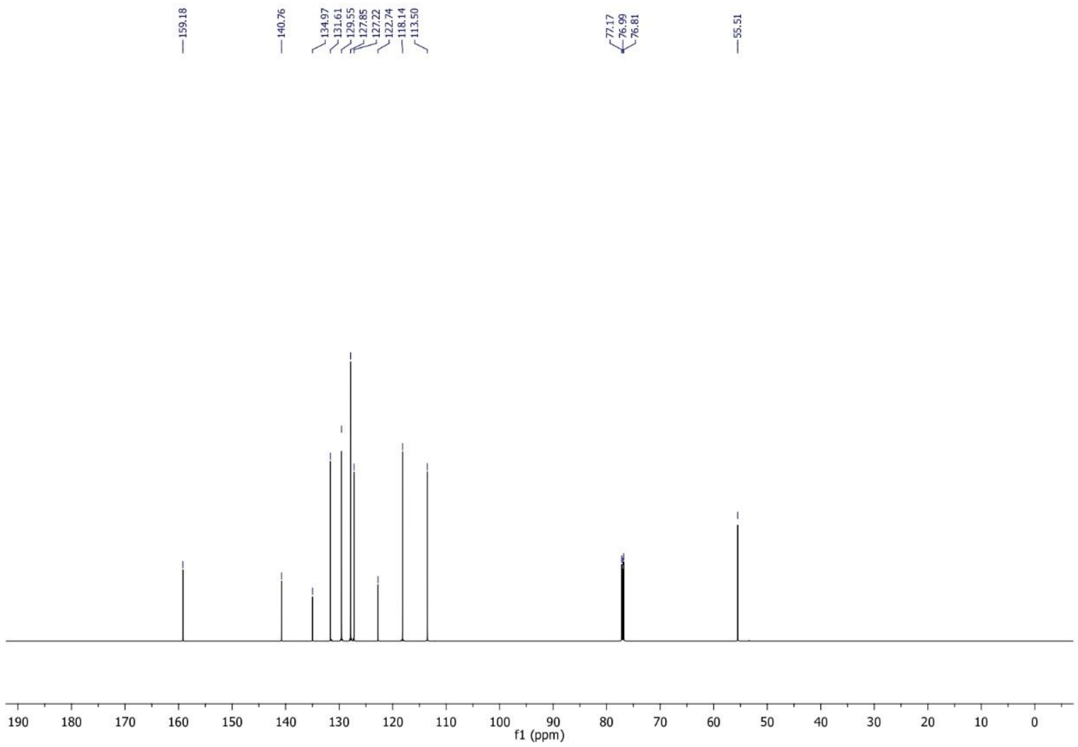

Figure S19. ^{13}C-NMR (176 MHz) spectrum of **2-bromo-4-methoxy-1,1'-biphenyl** in $CDCl_3$.

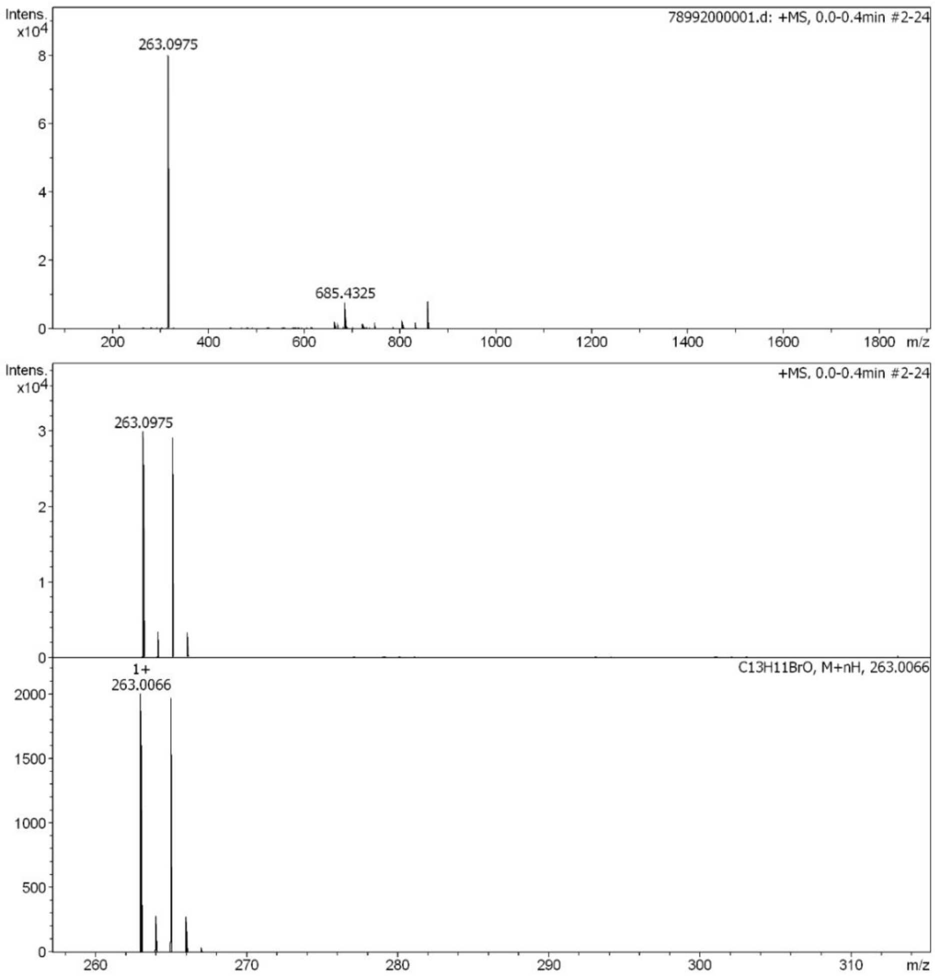

Figure S20. HRMS-ESImass spectrum of **2-bromo-4-methoxy-1,1'-biphenyl**

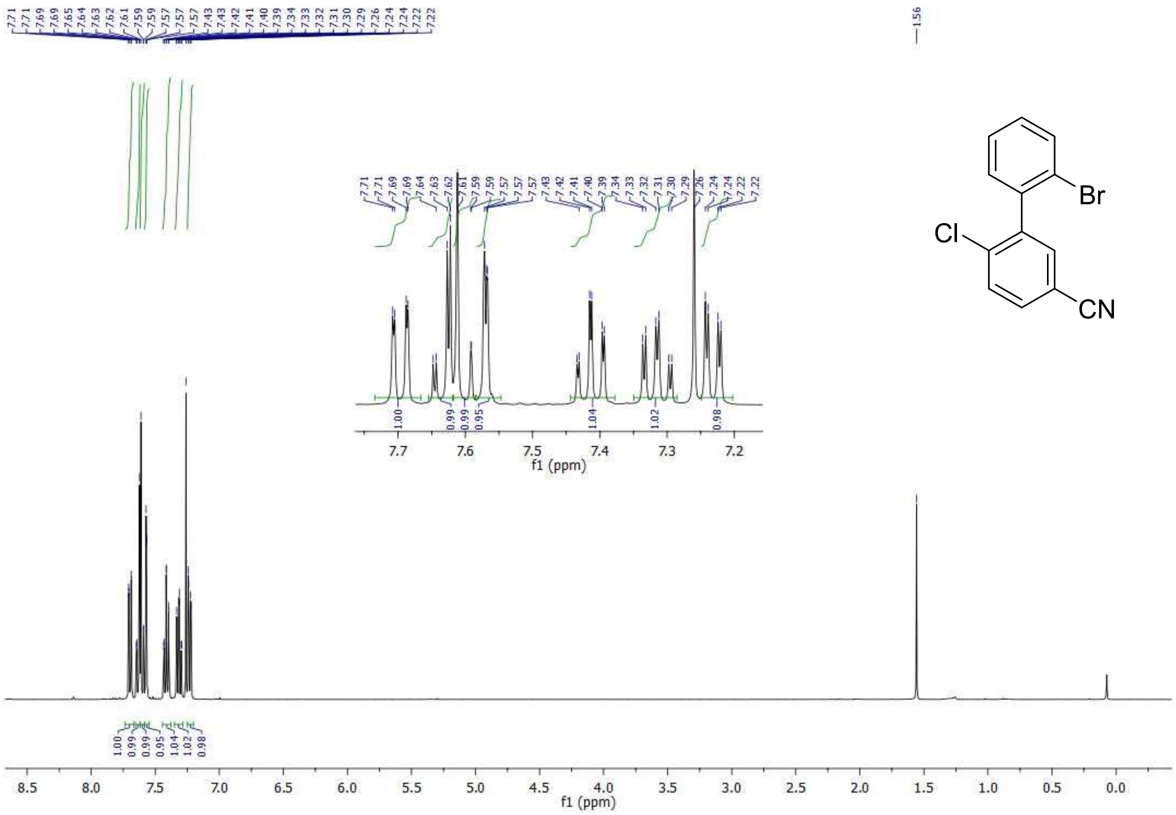

Figure S21. ^{1}H-NMR (400 MHz) spectrum of **2'-bromo-6-chloro-[1,1'-biphenyl]-3-carbonitrile** in $CDCl_3$.

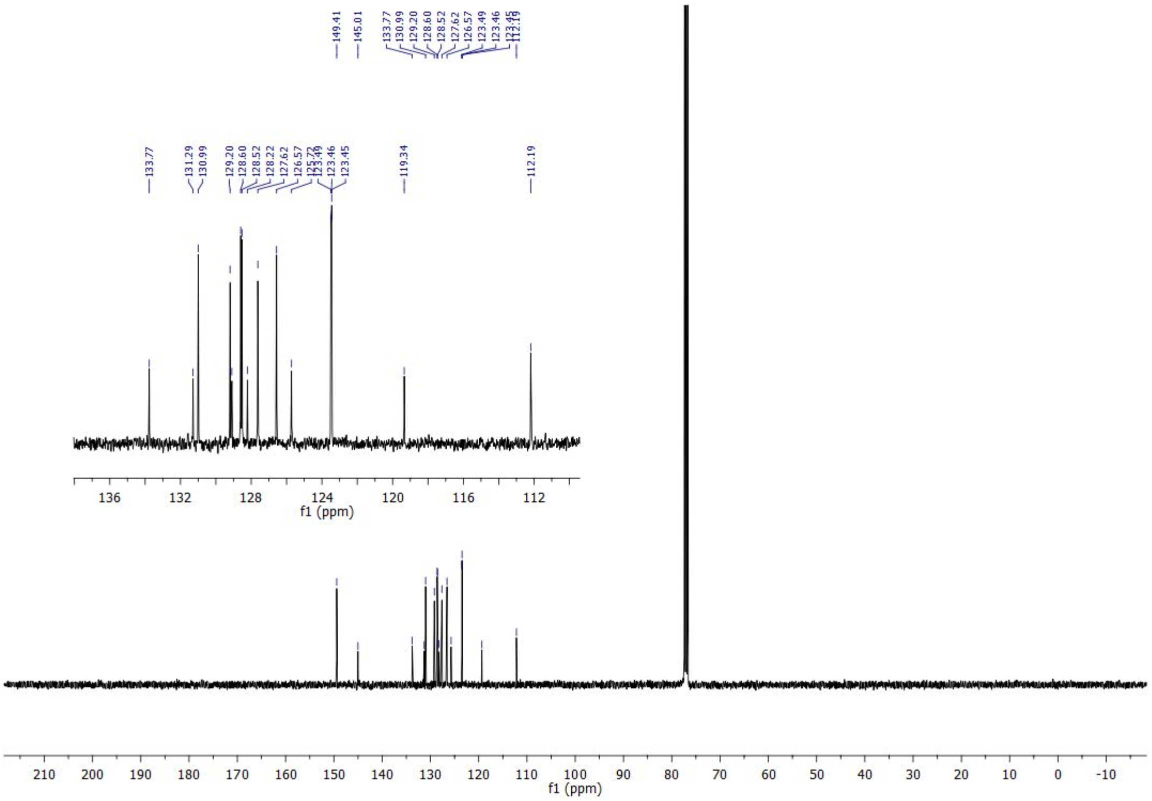

Figure S22. ^{13}C-NMR (101 MHz) spectrum of **2'-bromo-6-chloro-[1,1'-biphenyl]-3-carbonitrile** in $CDCl_3$.

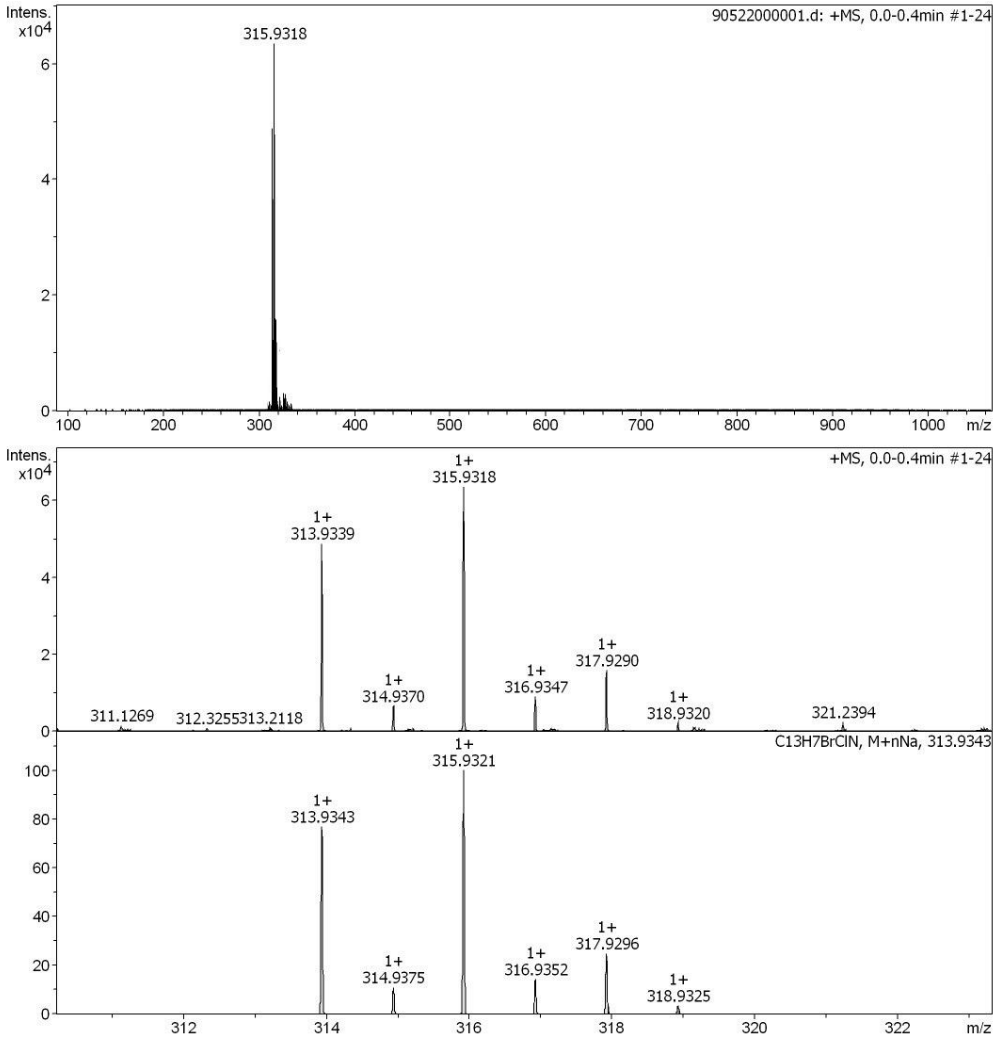

Figure S23. HRMS-ESI mass spectrum of **2'-bromo-6-chloro-[1,1'-biphenyl]-3-carbonitrile.**

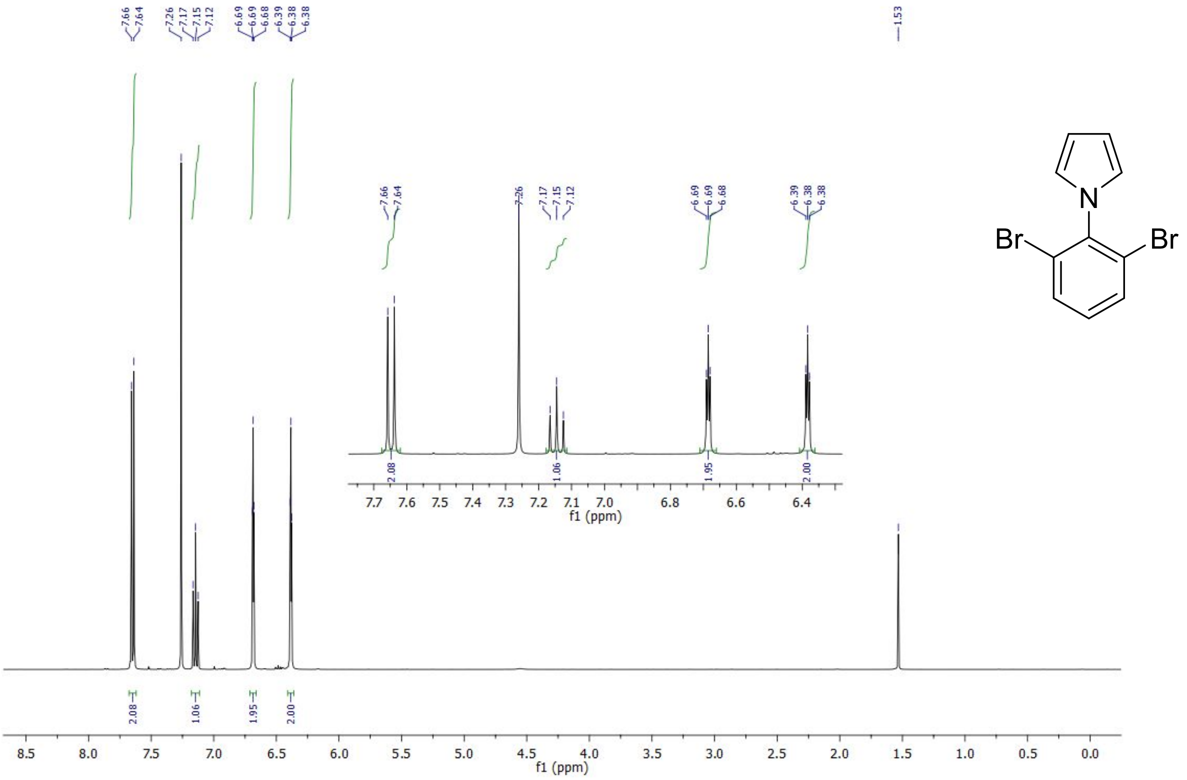

Figure S24. ^{1}H-NMR (600 MHz) spectrum of **1-(2,6-dibromophenyl)-1H-pyrrole** in $CDCl_3$.

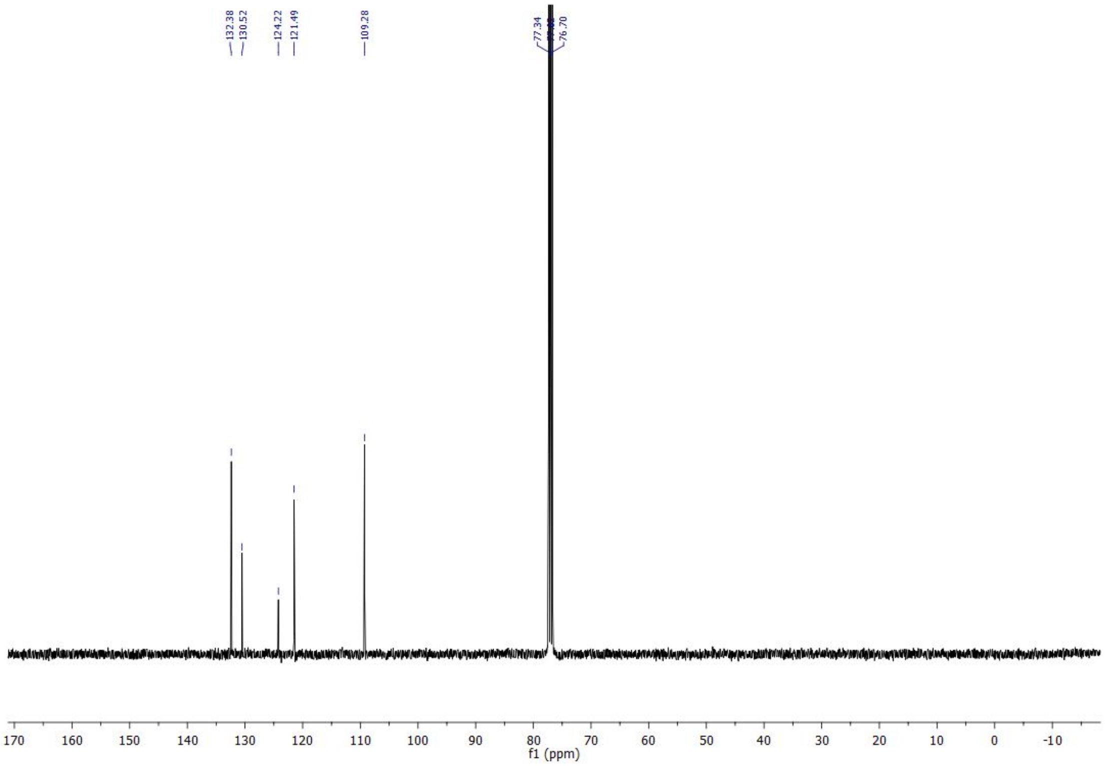

Figure S25. ^{13}C-NMR (151 MHz) spectrum of **1-(2,6-dibromophenyl)-1H-pyrrole** in $CDCl_3$.

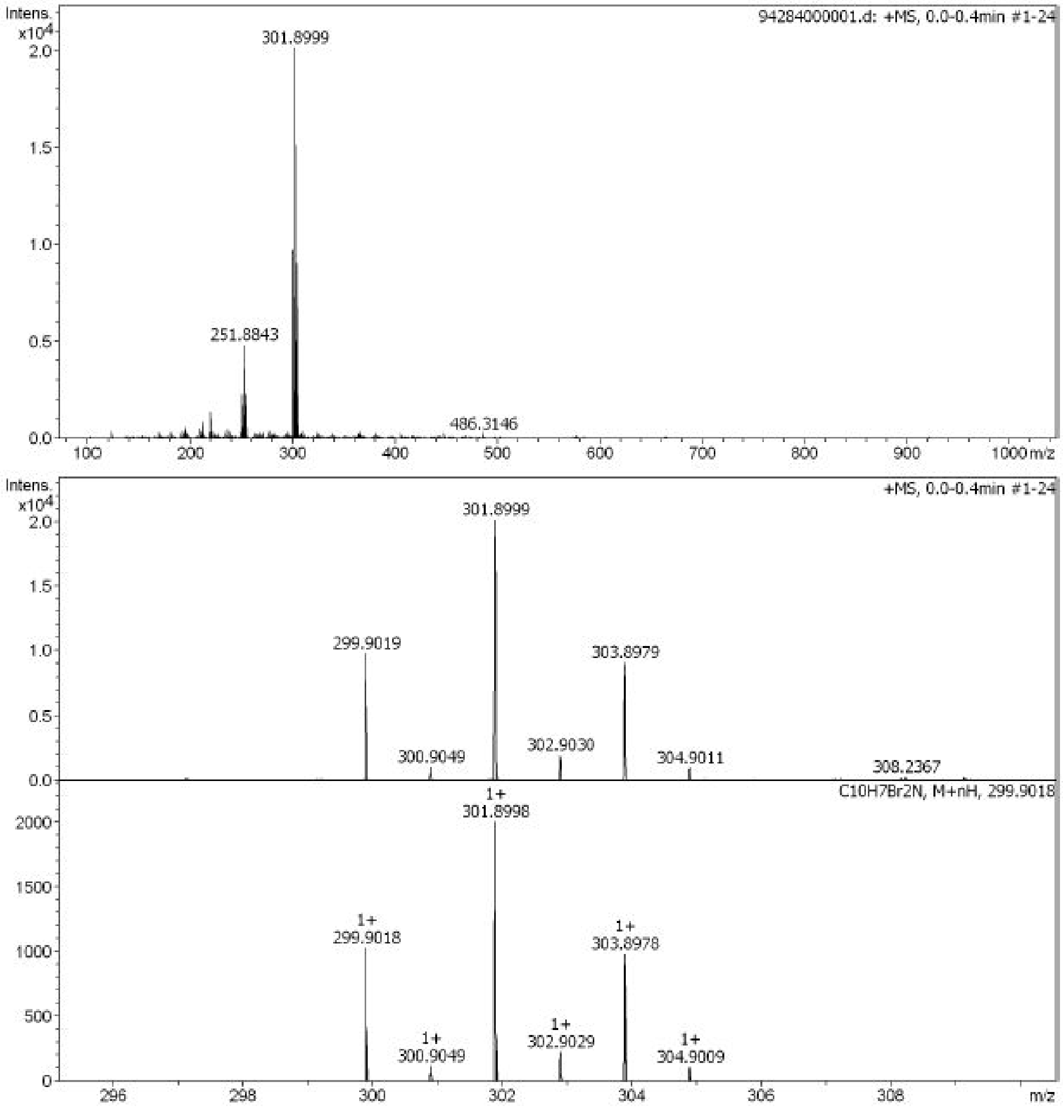

Figure S26. HRMS-ESI mass spectrum of **1-(2,6-dibromophenyl)-1H-pyrrole**.

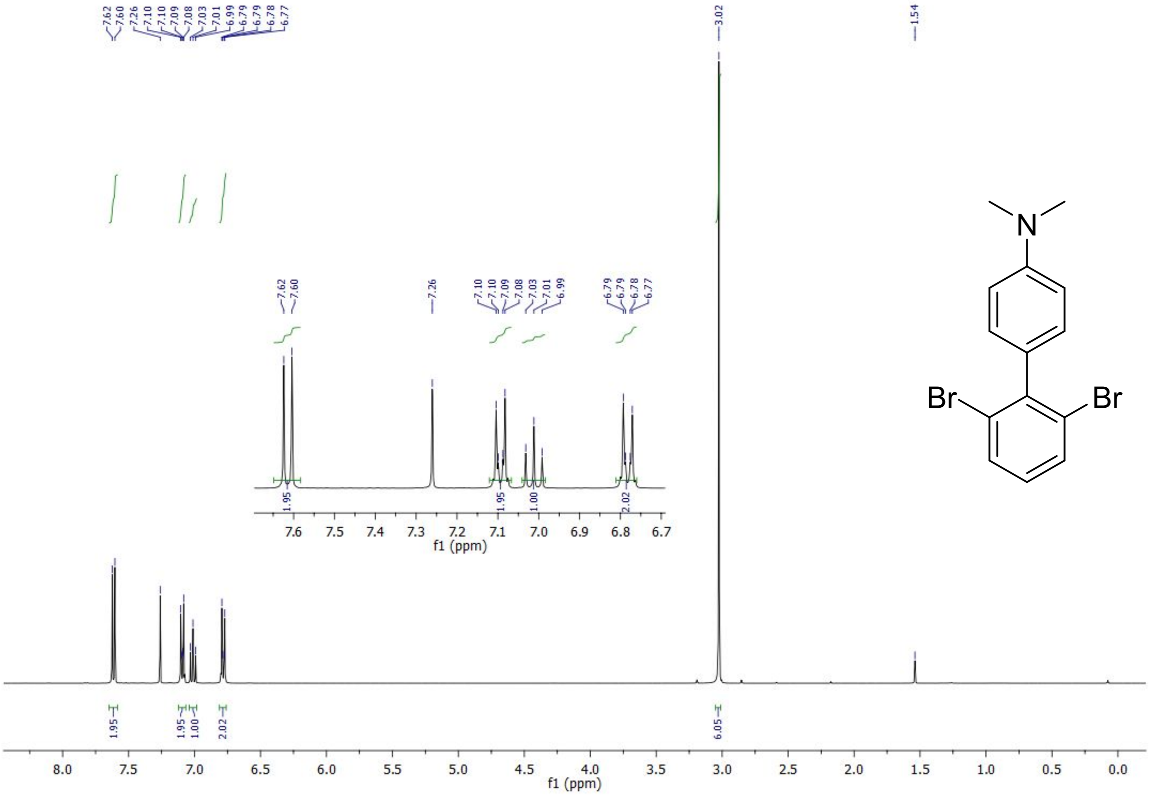

Figure S27. ^{1}H-NMR (600 MHz) spectrum of **2',6'-dibromo-N,N-dimethyl-[1,1'-biphenyl]-4-amine** in $CDCl_3$.

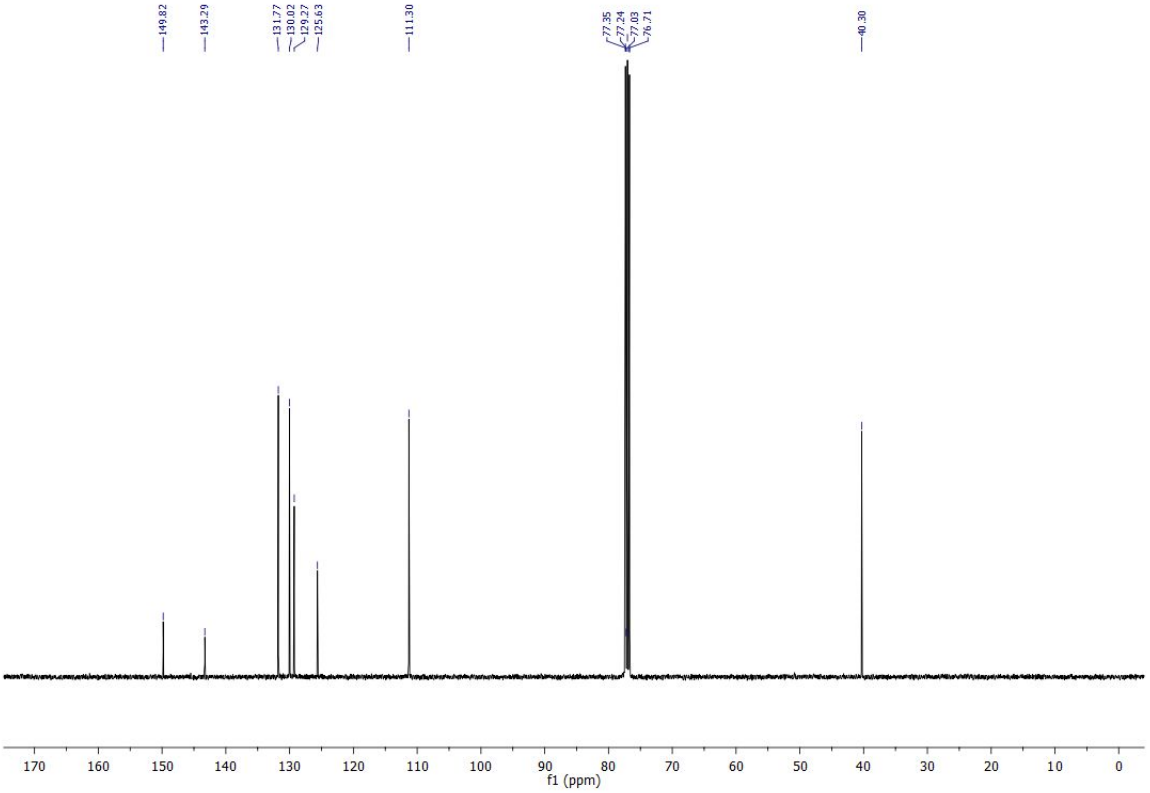

Figure S28. ^{13}C-NMR (151 MHz) spectrum of **2',6'-dibromo-N,N-dimethyl-[1,1'-biphenyl]-4-amine** in $CDCl_3$.

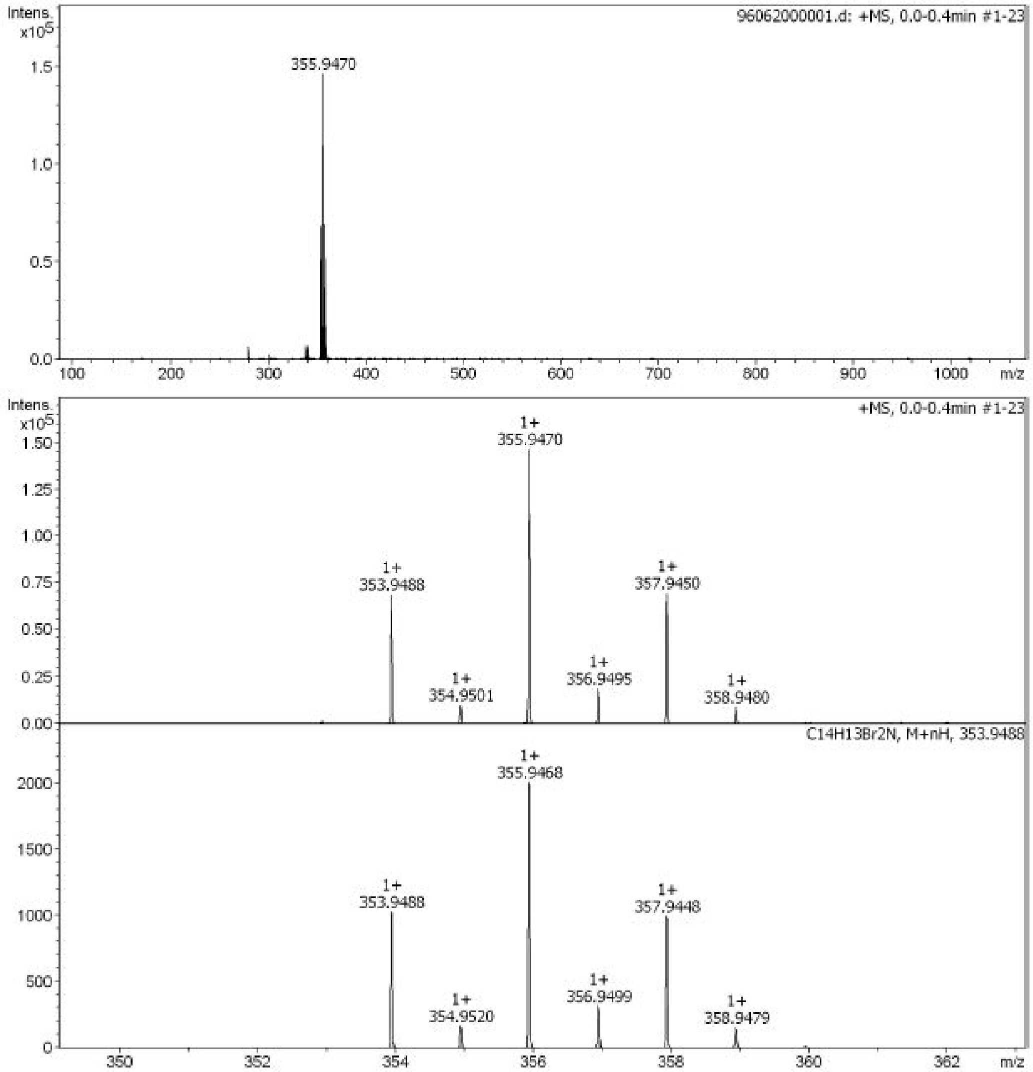

Figure S29. HRMS-ESI mass spectrum of **2',6'-dibromo-N,N-dimethyl-[1,1'-biphenyl]-4-amine**.

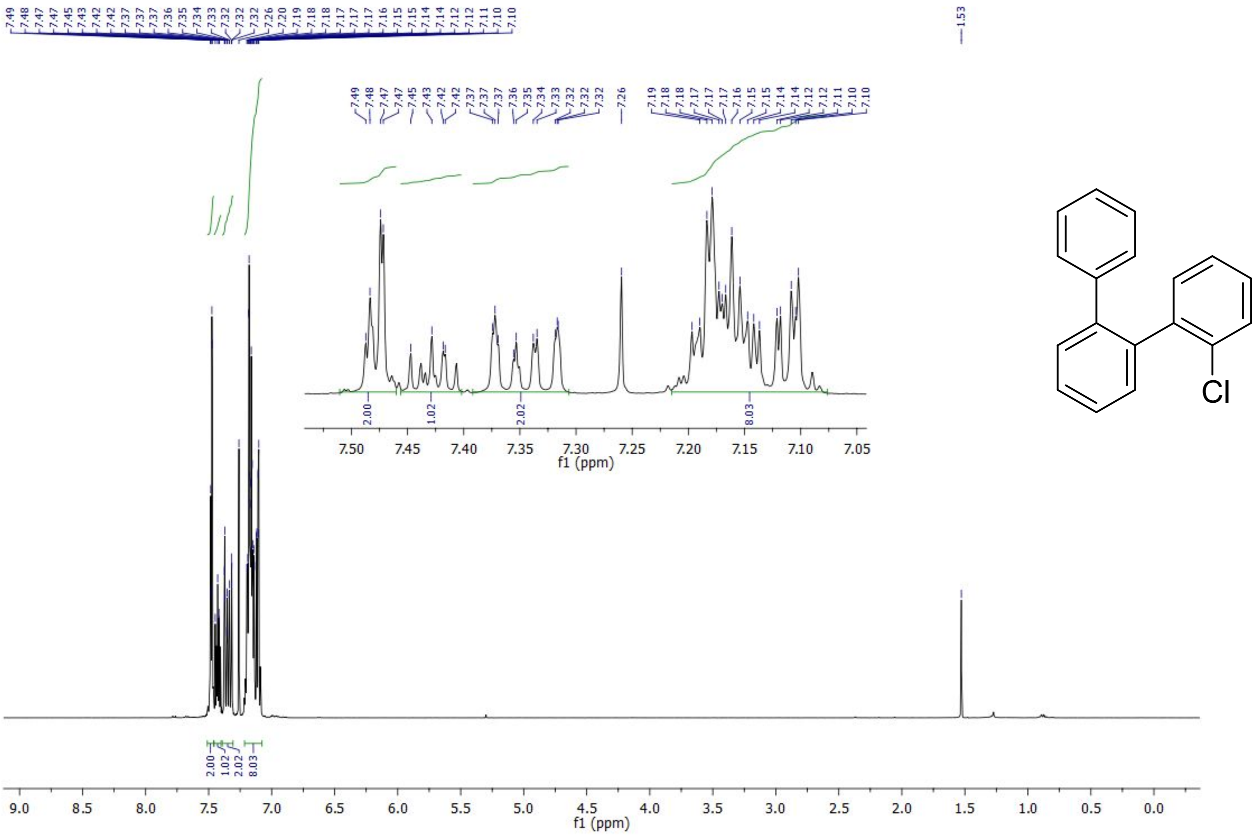

Figure S30. ^{1}H-NMR (600 MHz) spectrum of **1a** in $CDCl_3$.

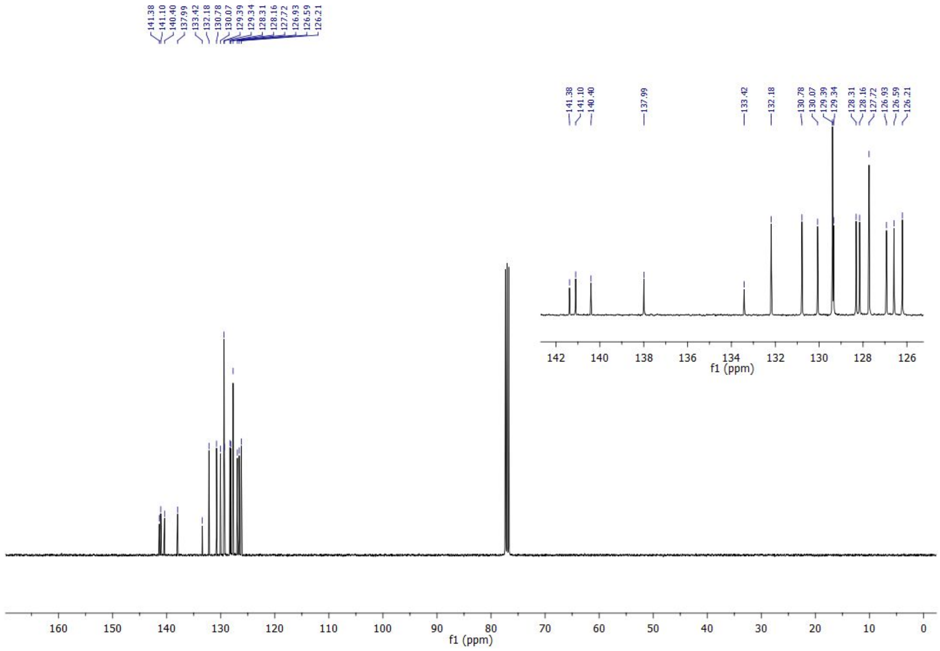

Figure S31. ^{13}C-NMR (151 MHz) spectrum of **1a** in $CDCl_3$.

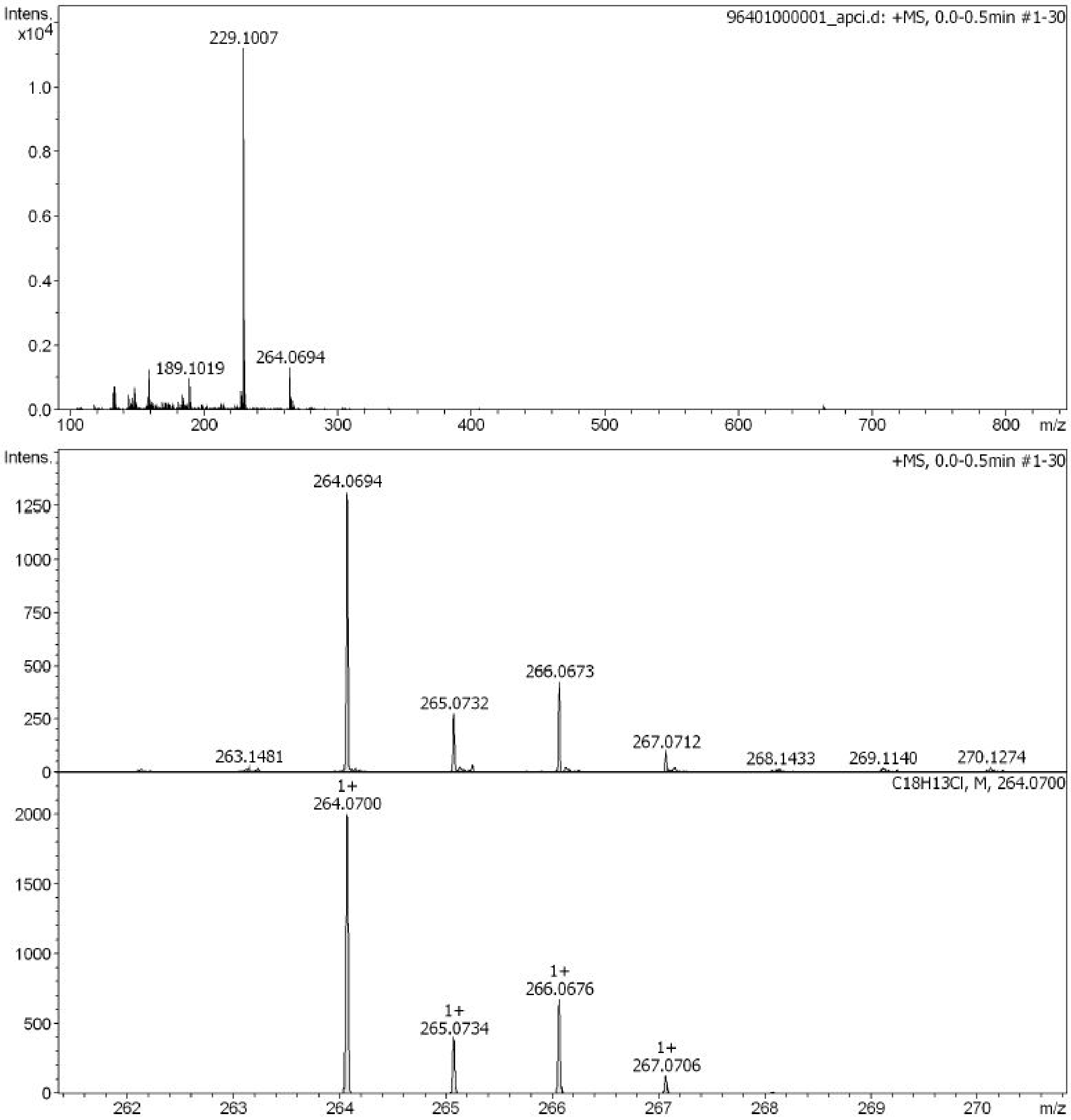

Figure S32. HRMS-APCI TOF mass spectrum of **1a**.

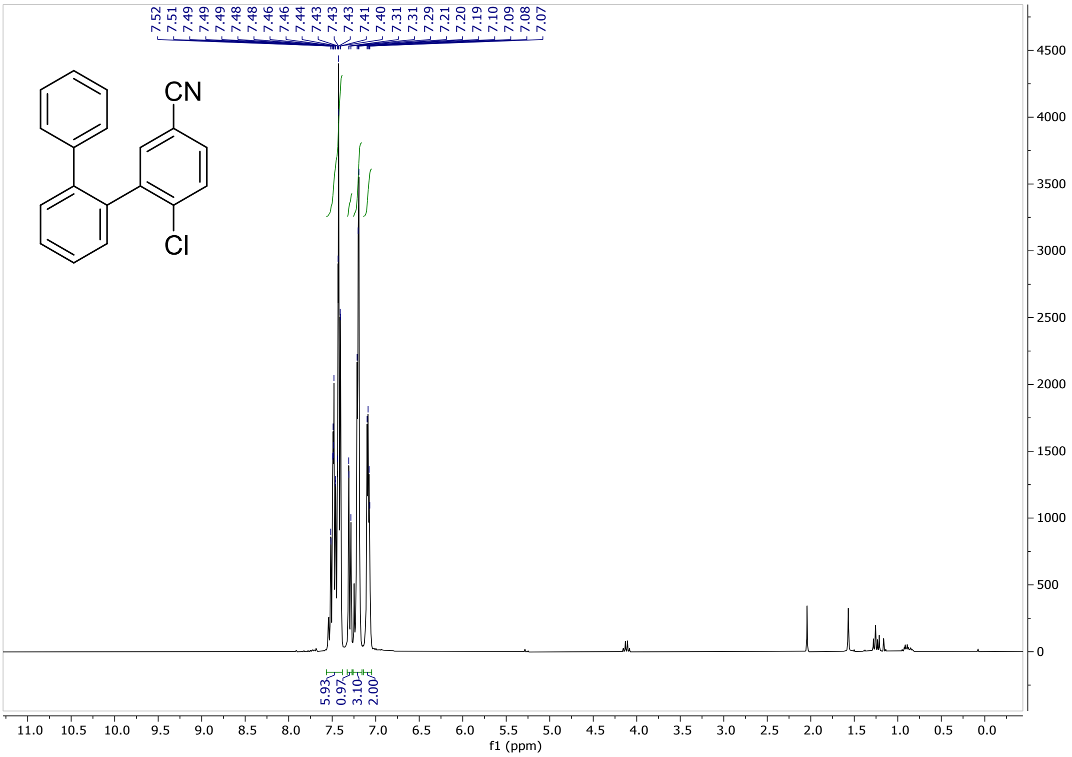

Figure S33. ^{1}H-NMR (300 MHz) spectrum of **1** in $CDCl_3$.

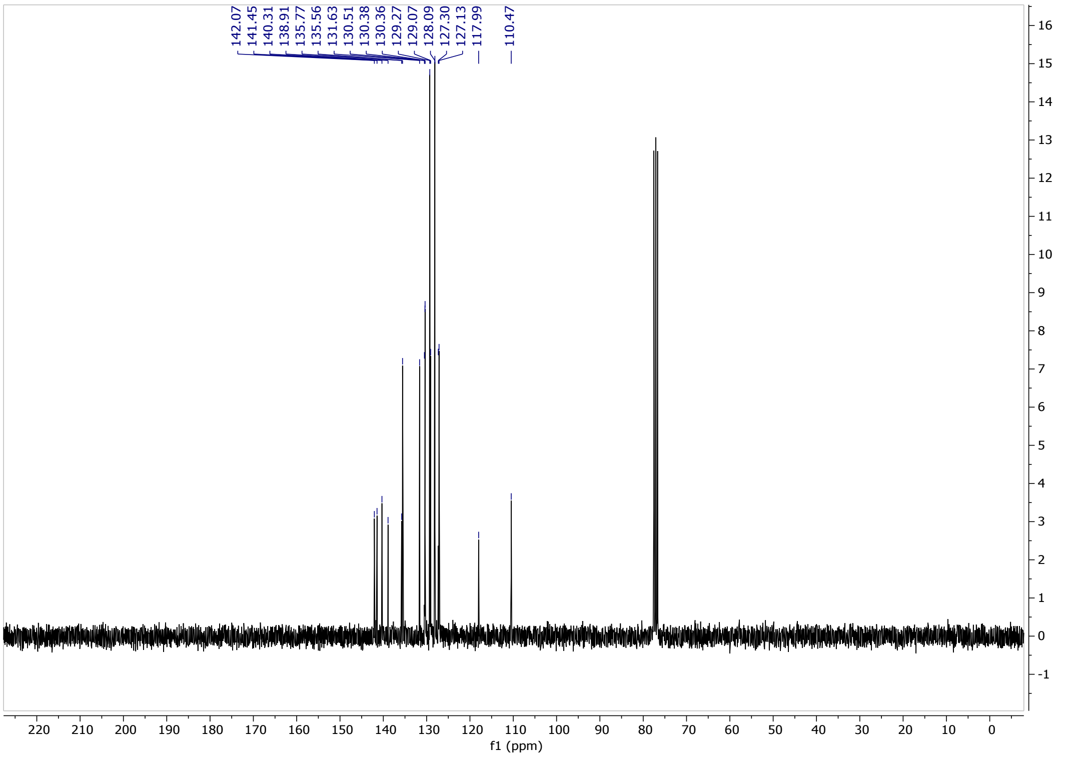

Figure S34. ^{13}C-NMR (75 MHz) spectrum of **1** in $CDCl_3$.

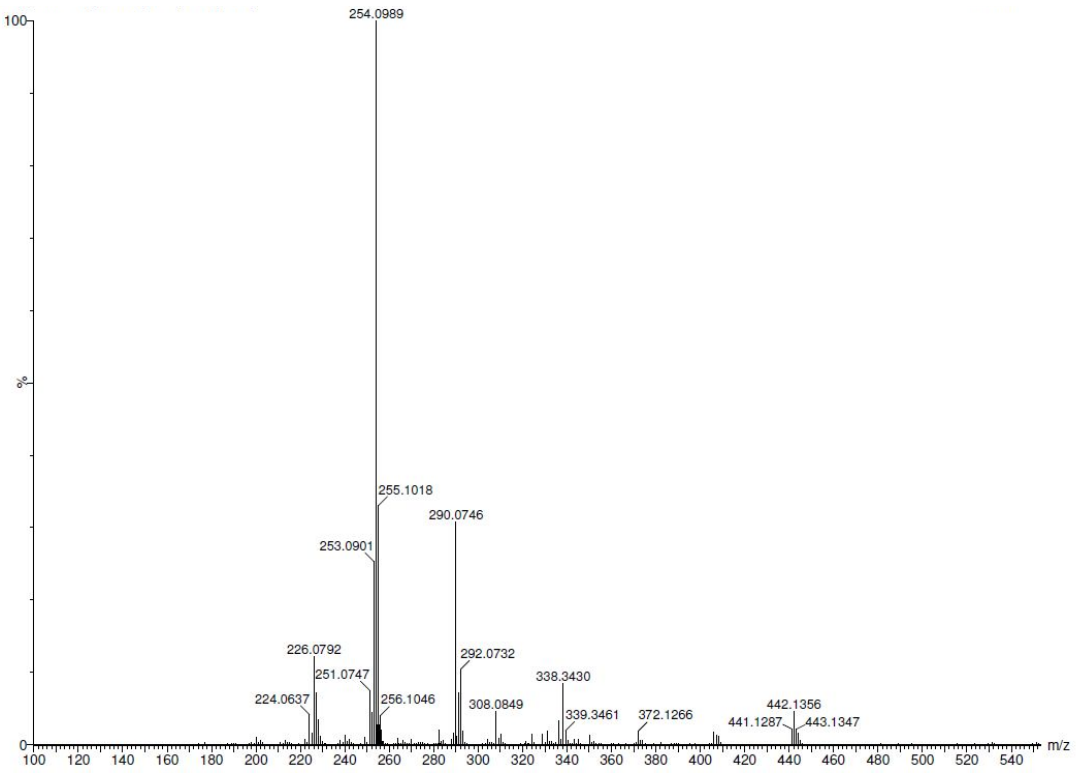

Figure S35. HRMS-APCI TOF mass spectrum of **1**.

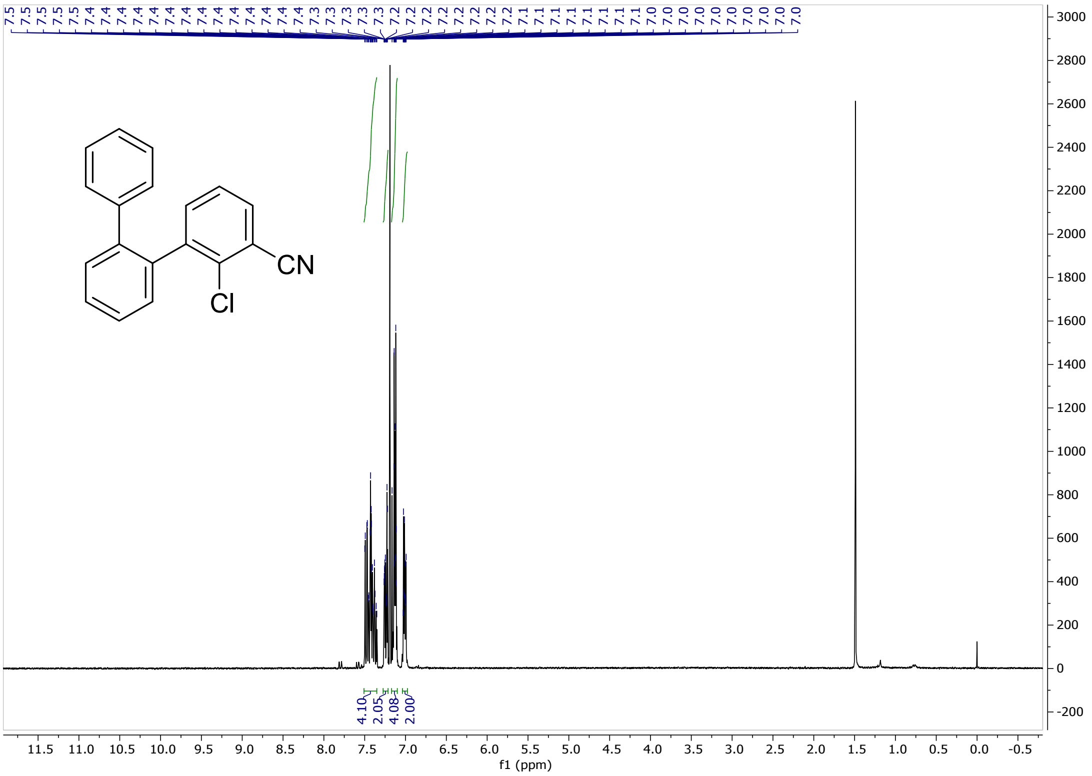

Figure S36. ^{1}H-NMR (300 MHz) spectrum of **3a** in $CDCl_3$.

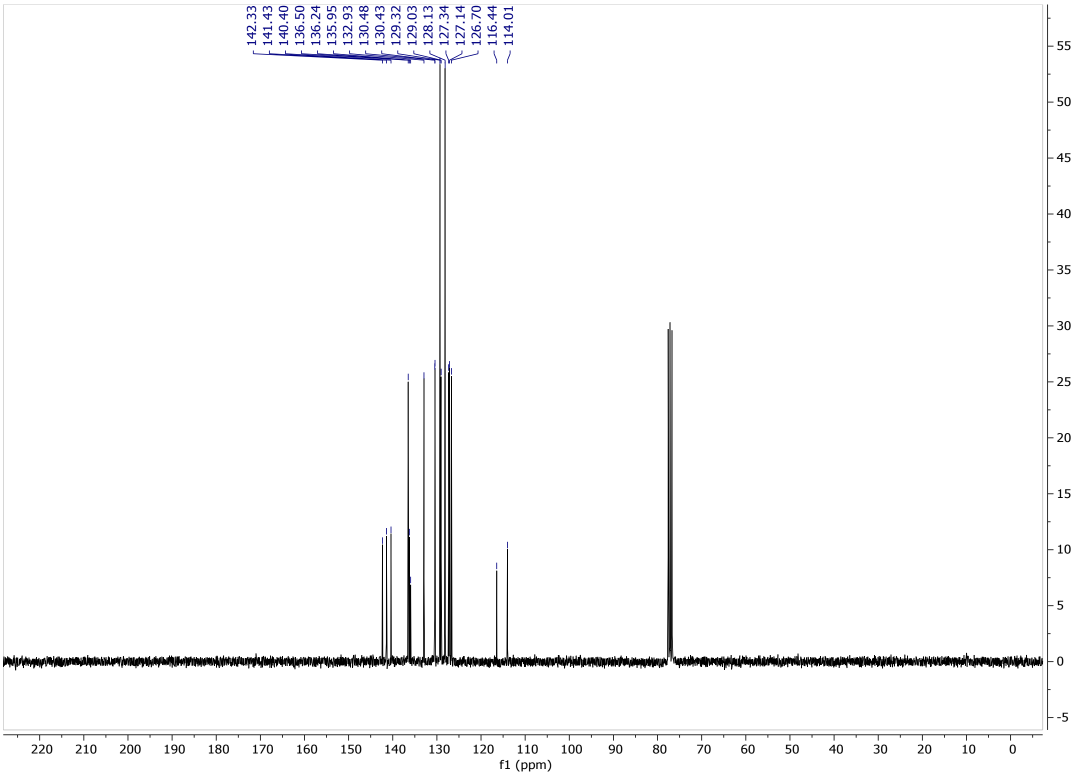

Figure S37. ^{13}C-NMR (75 MHz) spectrum of **3a** in $CDCl_3$.

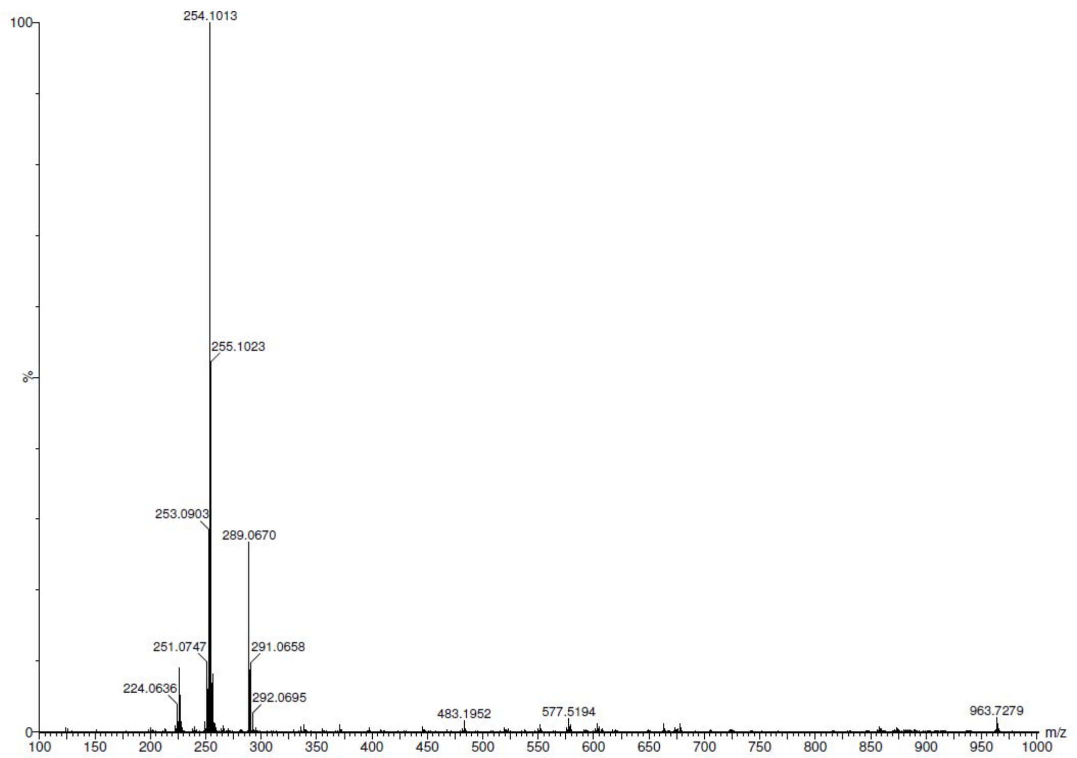

Figure S38. HRMS-APCI TOF mass spectrum of **3a**.

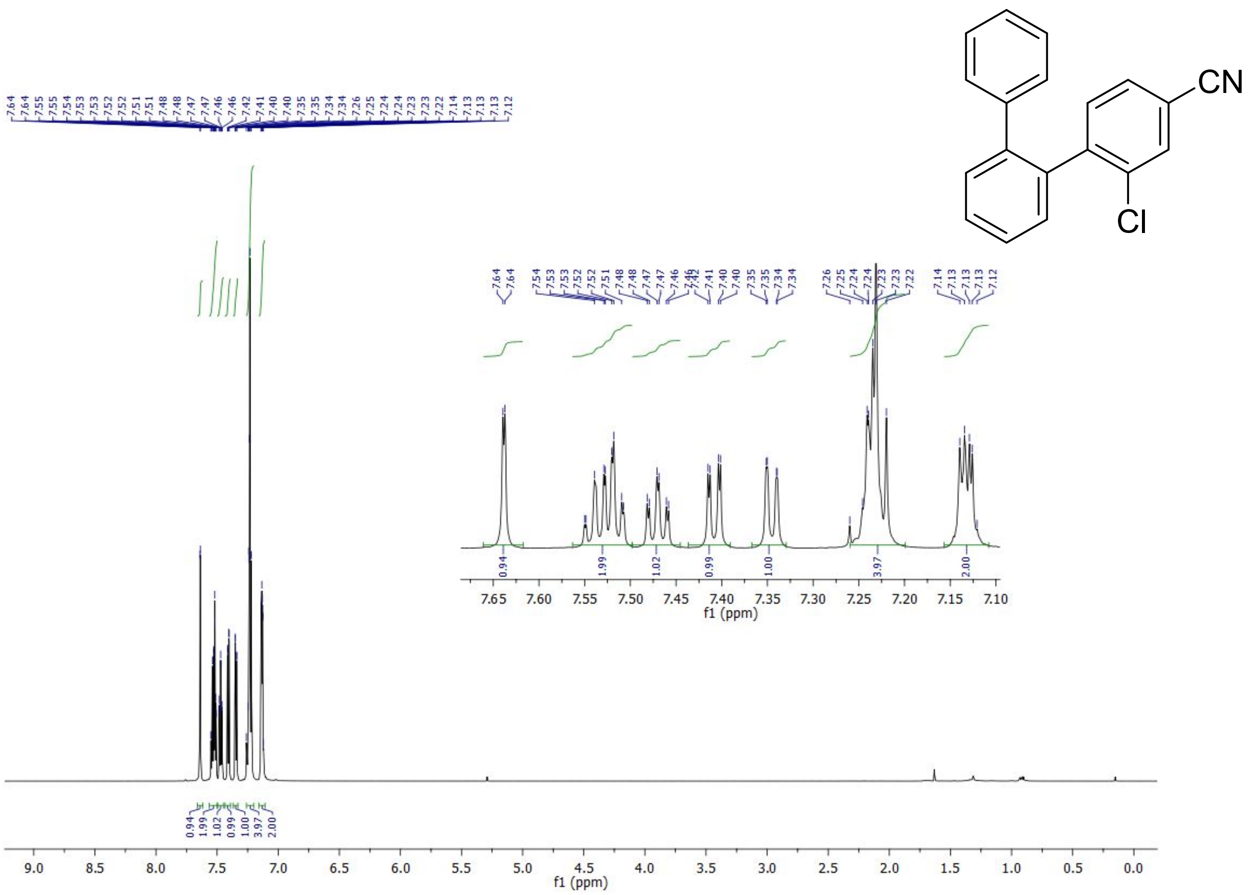

Figure S39. ^{1}H-NMR (700 MHz) spectrum of **3b** in $CDCl_3$.

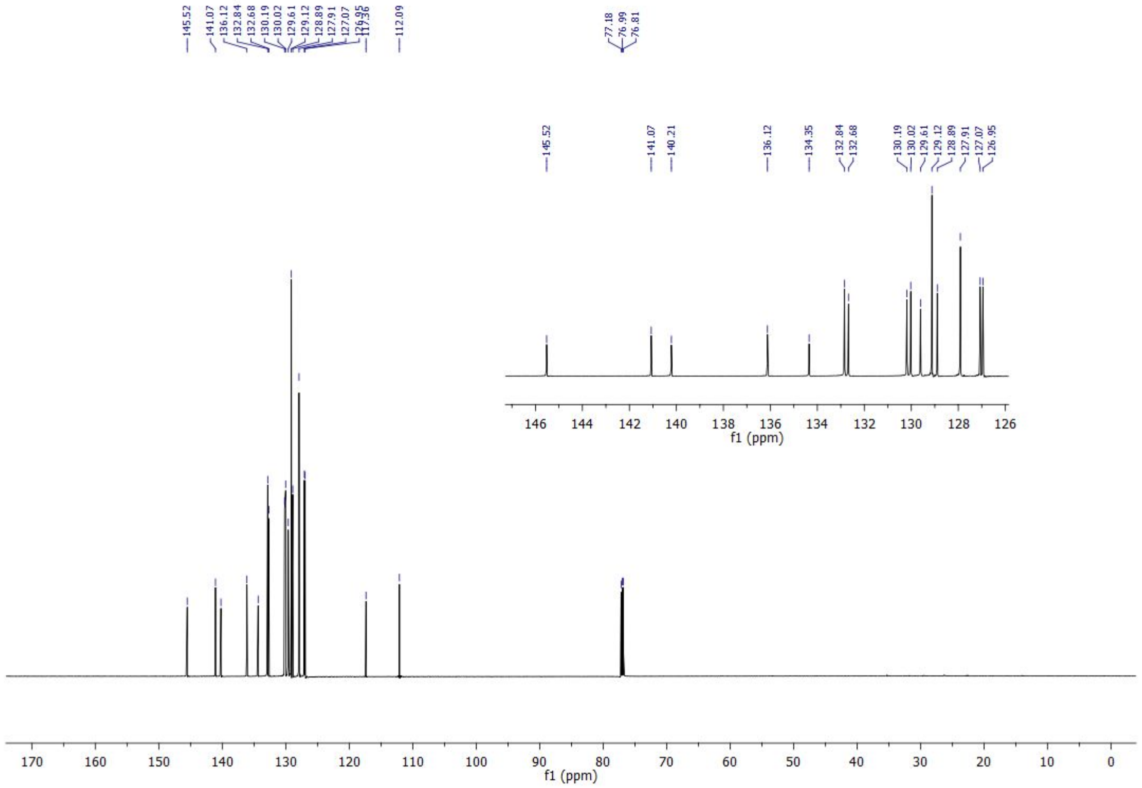

Figure S40. ^{13}C-NMR (176 MHz) spectrum of **3b** in $CDCl_3$.

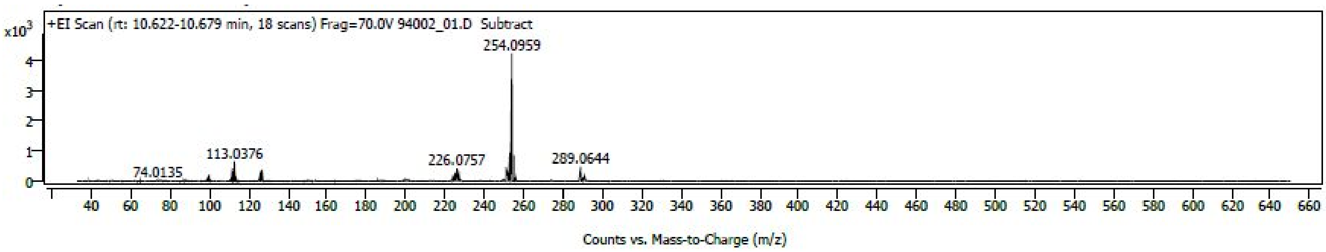

Figure S41. HRGC-MS mass spectrum of **3b.**

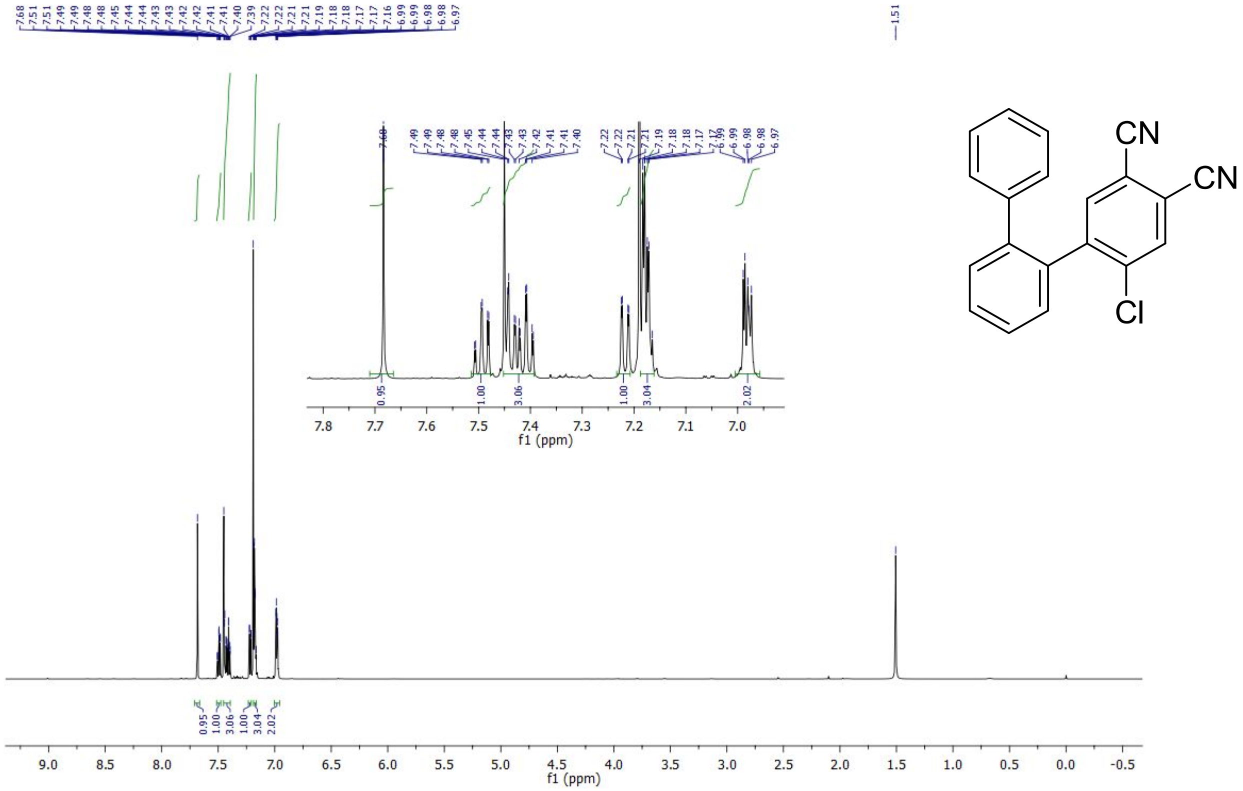

Figure S42. ^{1}H-NMR (600 MHz) spectrum of **3c** in $CDCl_3$.

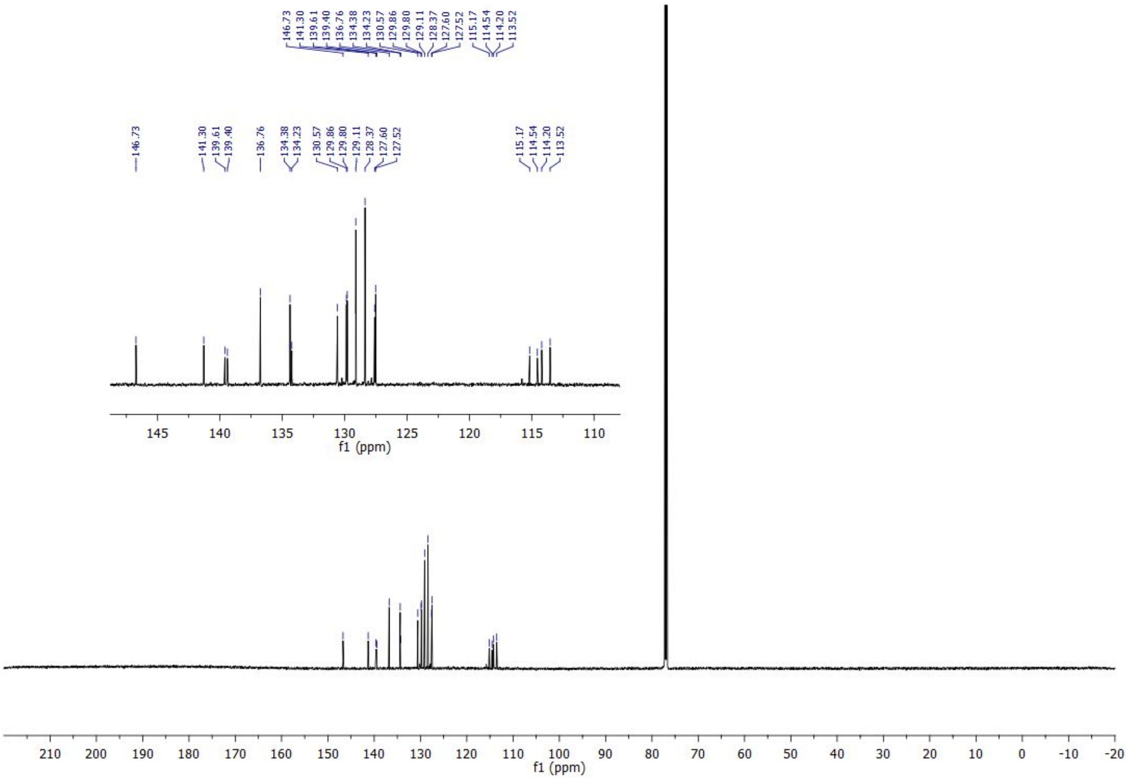

Figure S43. ^{13}C-NMR (151 MHz) spectrum of **3c** in $CDCl_3$.

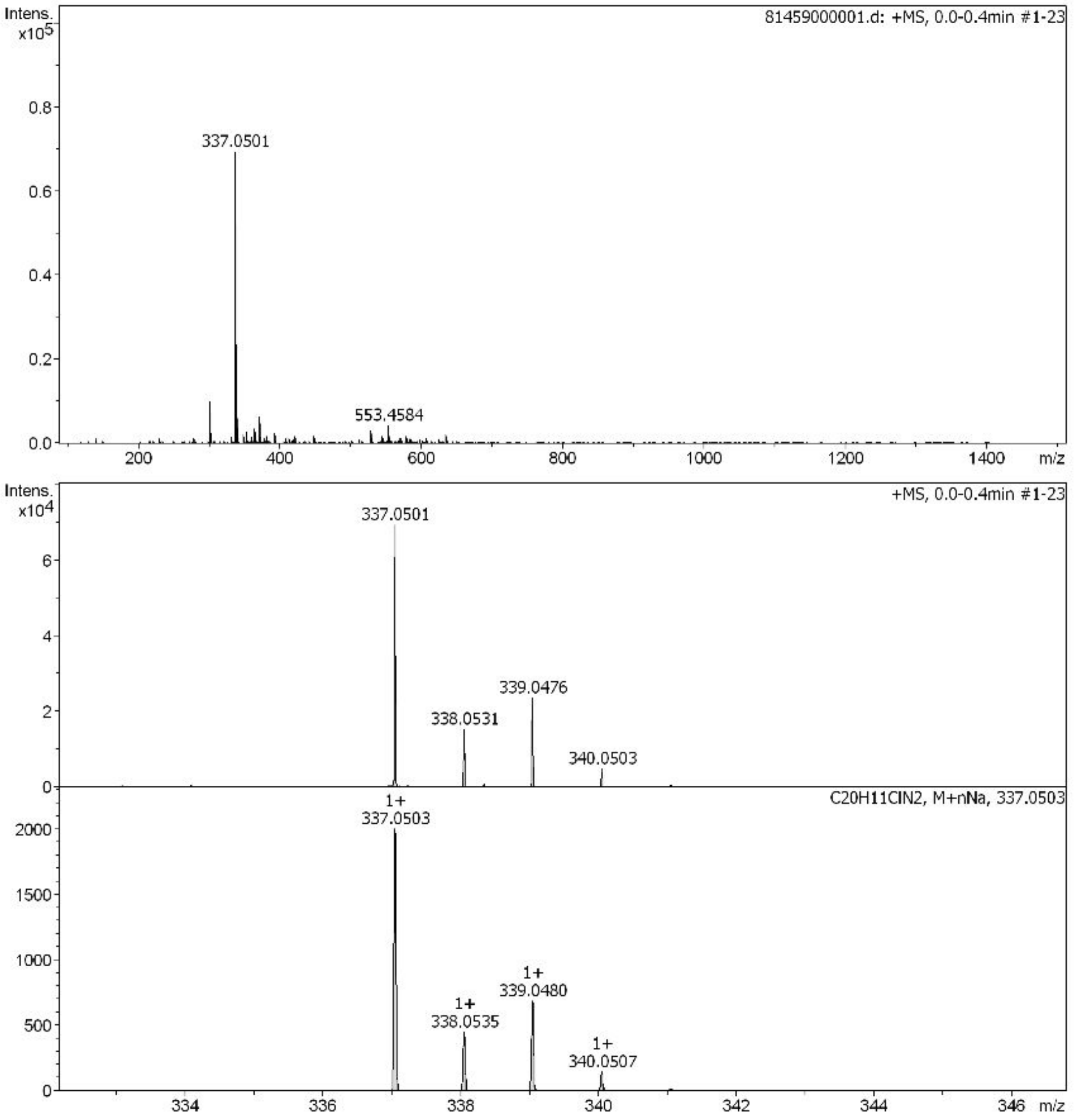

Figure S44. HRMS-APCI TOF mass spectrum of **3c**.

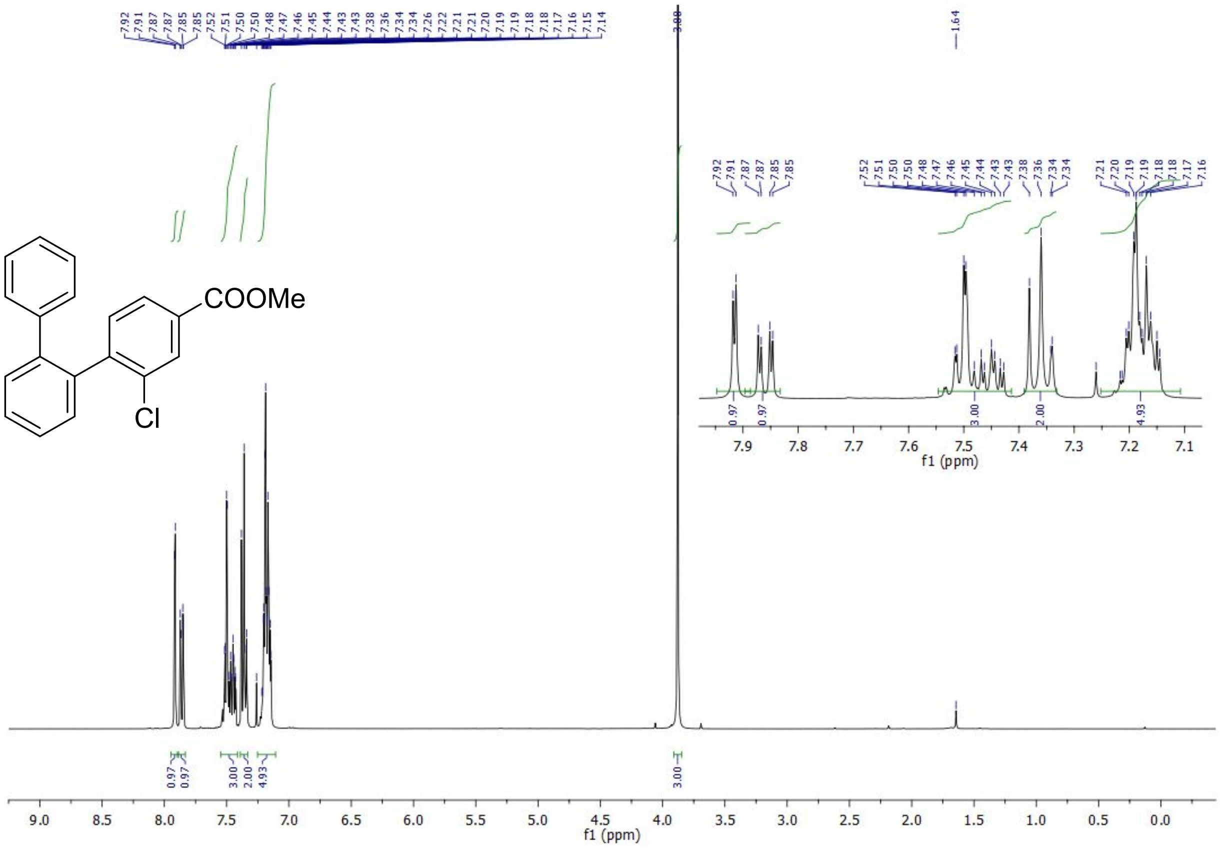

Figure S45. ^{1}H-NMR (600 MHz) spectrum of **3d** in $CDCl_3$.

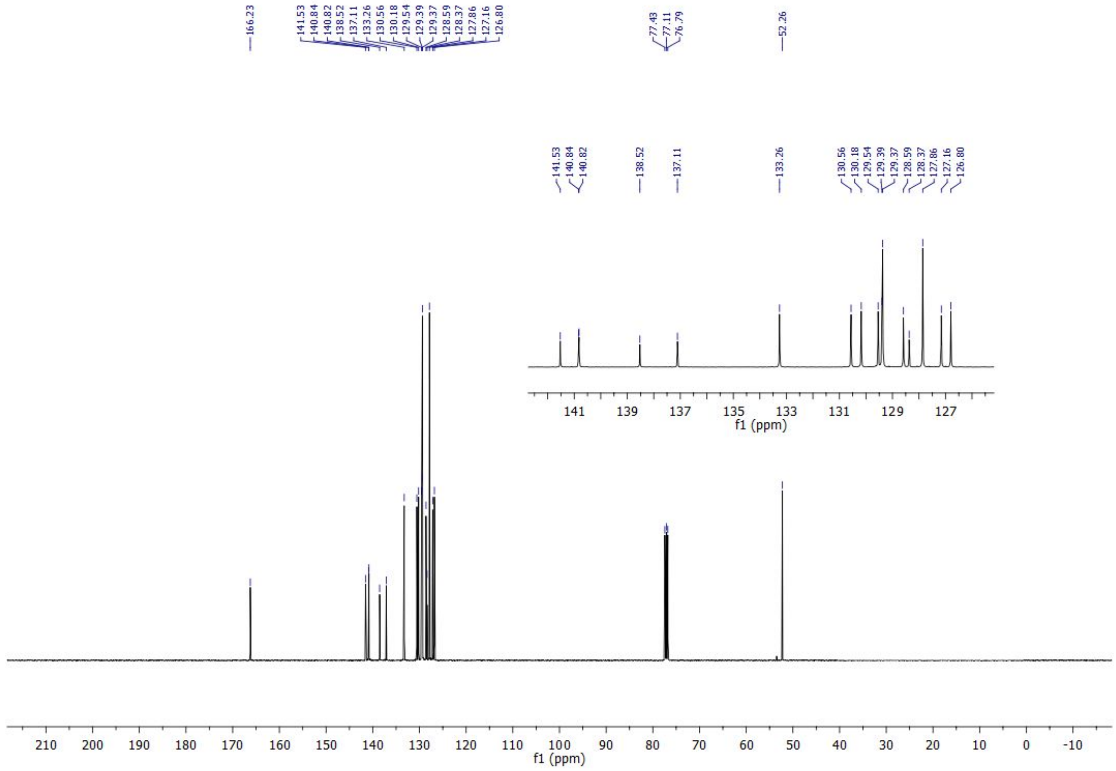

Figure S46. ^{13}C-NMR (151 MHz) spectrum of **3d** in $CDCl_3$.

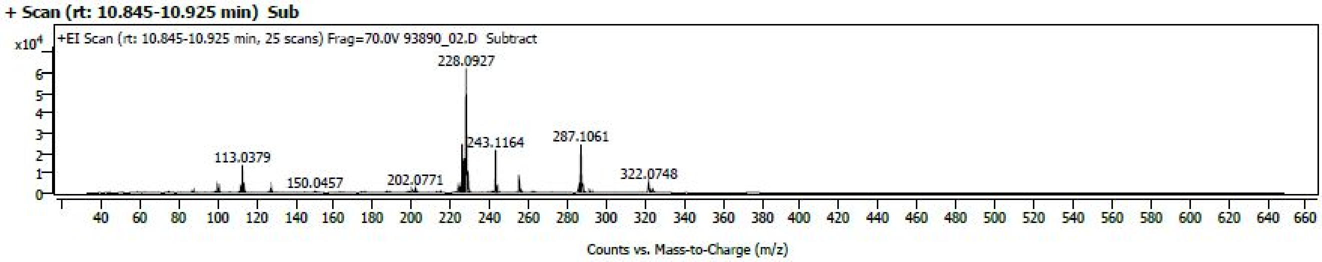

Figure S48. HRGC-MS mass spectrum of **3d**.

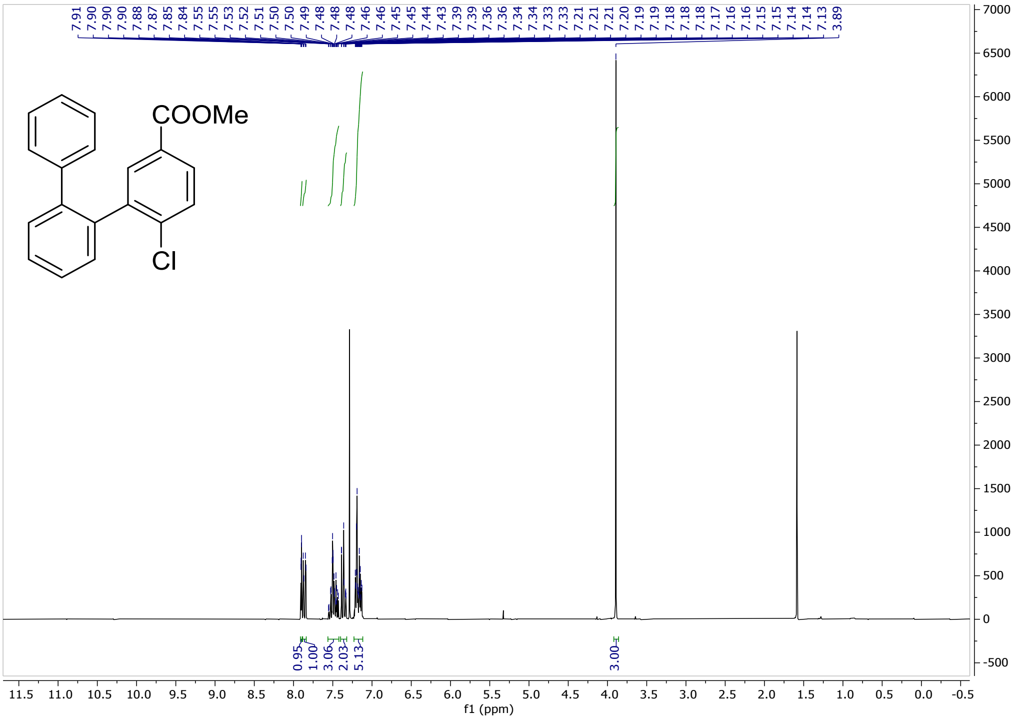

Figure S48. ^{1}H-NMR (300 MHz) spectrum of **3e** in $CDCl_3$.

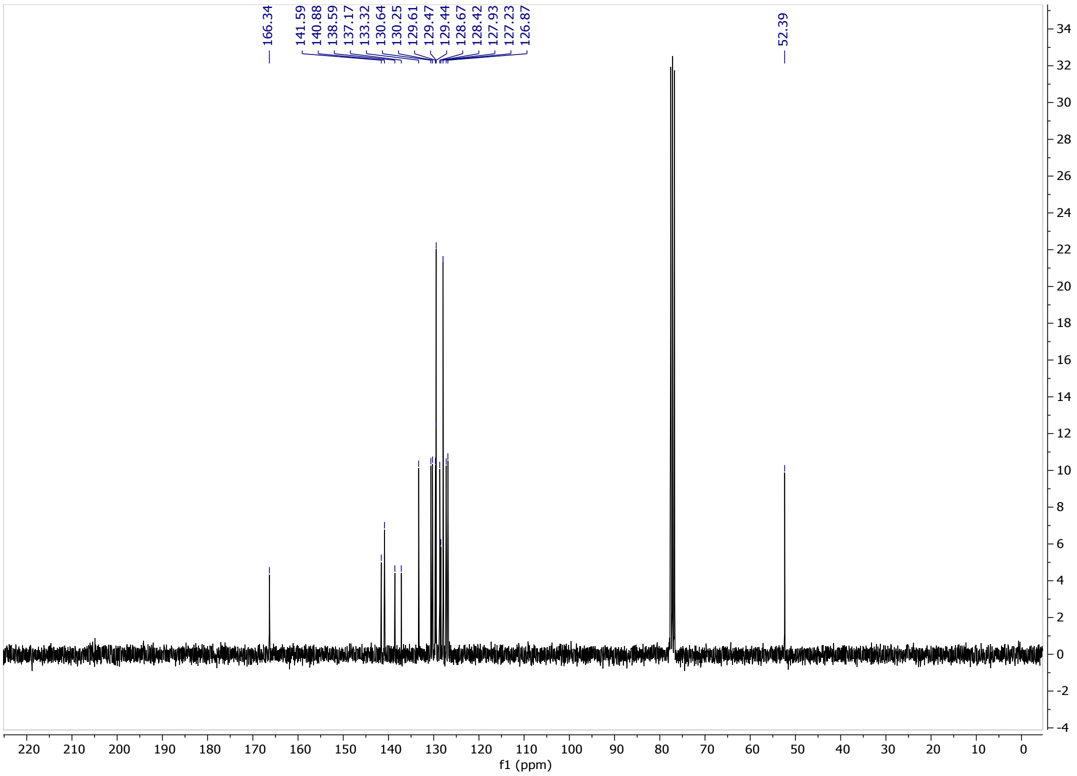

Figure S49. ^{13}C-NMR (75 MHz) spectrum of **3e** in $CDCl_3$.

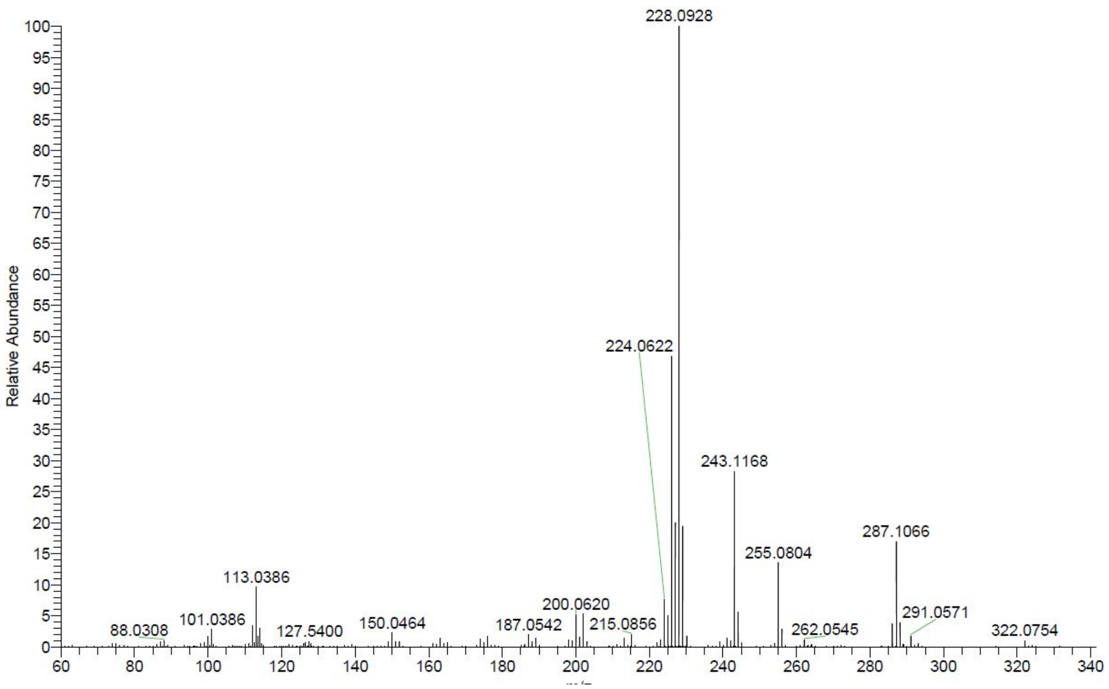

Figure S50. HRMS-APCI TOF mass spectrum of **3e**.

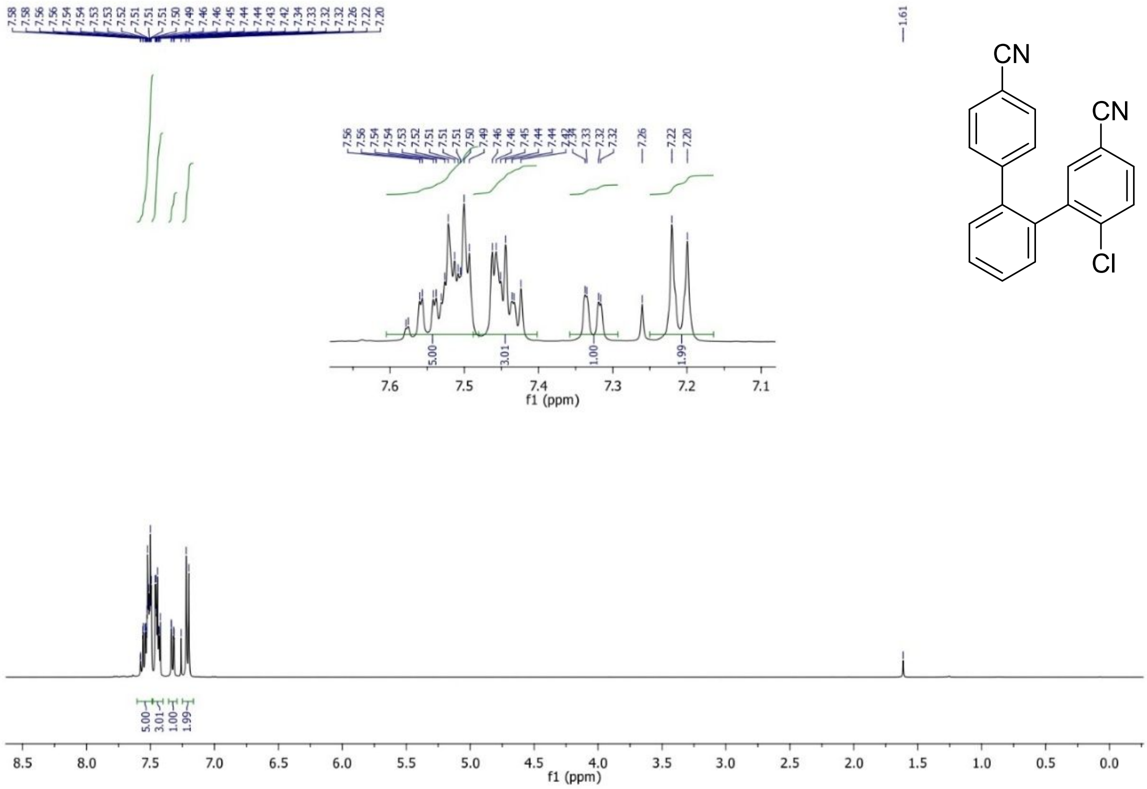

Figure S51. ^{1}H-NMR (600 MHz) spectrum of **3f** in $CDCl_3$.

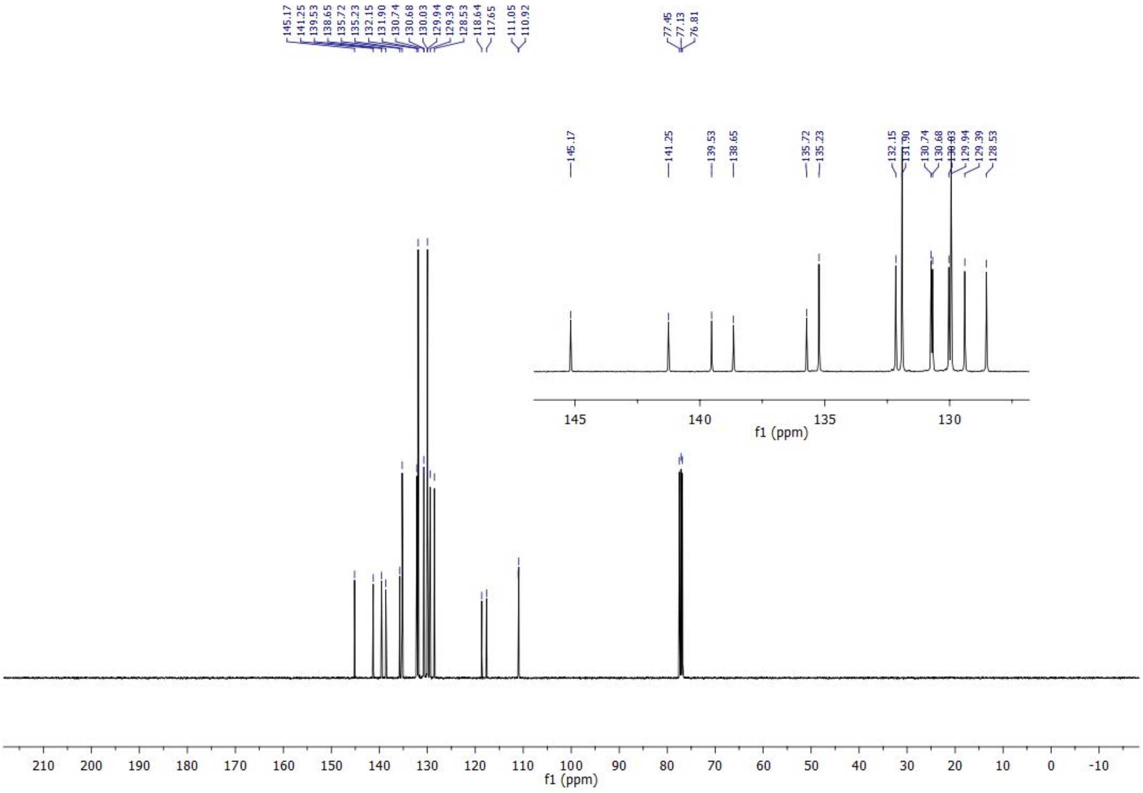

Figure S52. ^{13}C-NMR (151 MHz) spectrum of **3f** in $CDCl_3$.

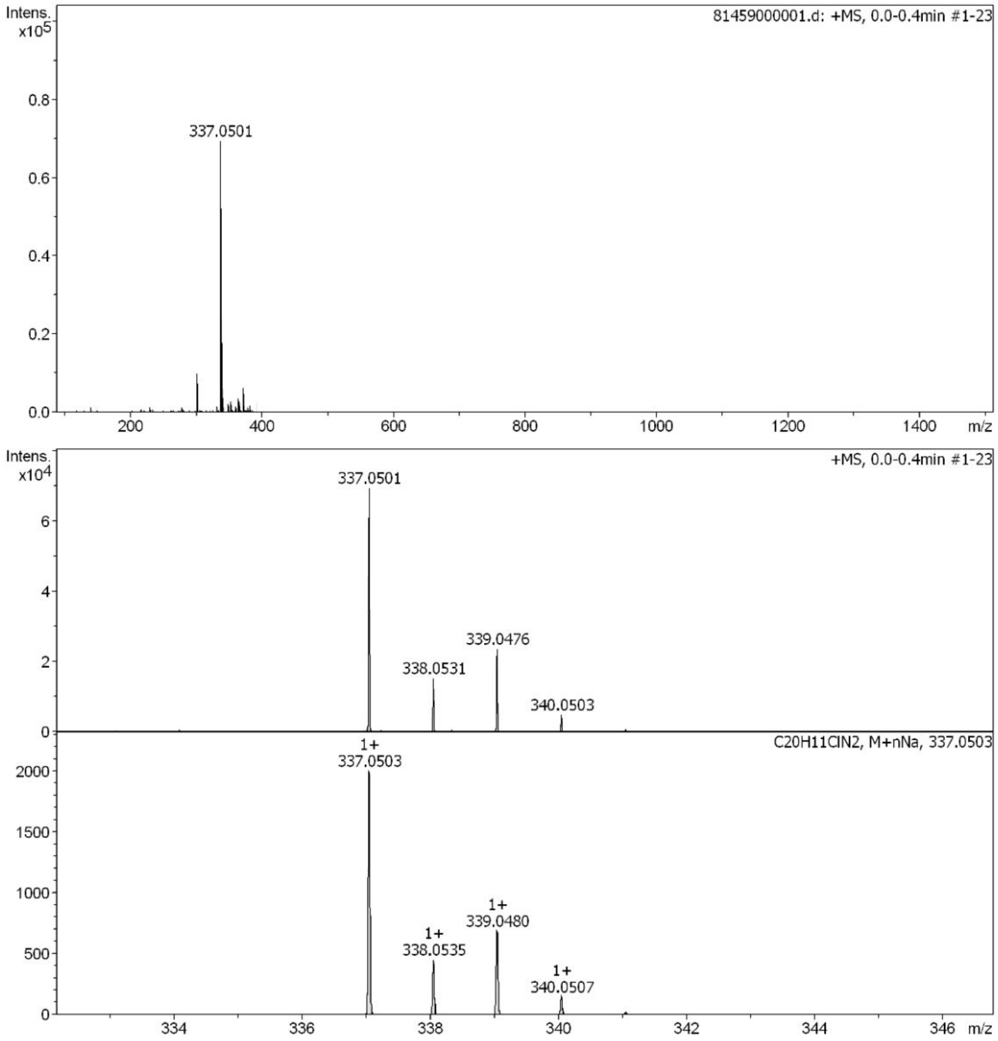

Figure S53. HRMS-ESI mass spectrum of **3f.**

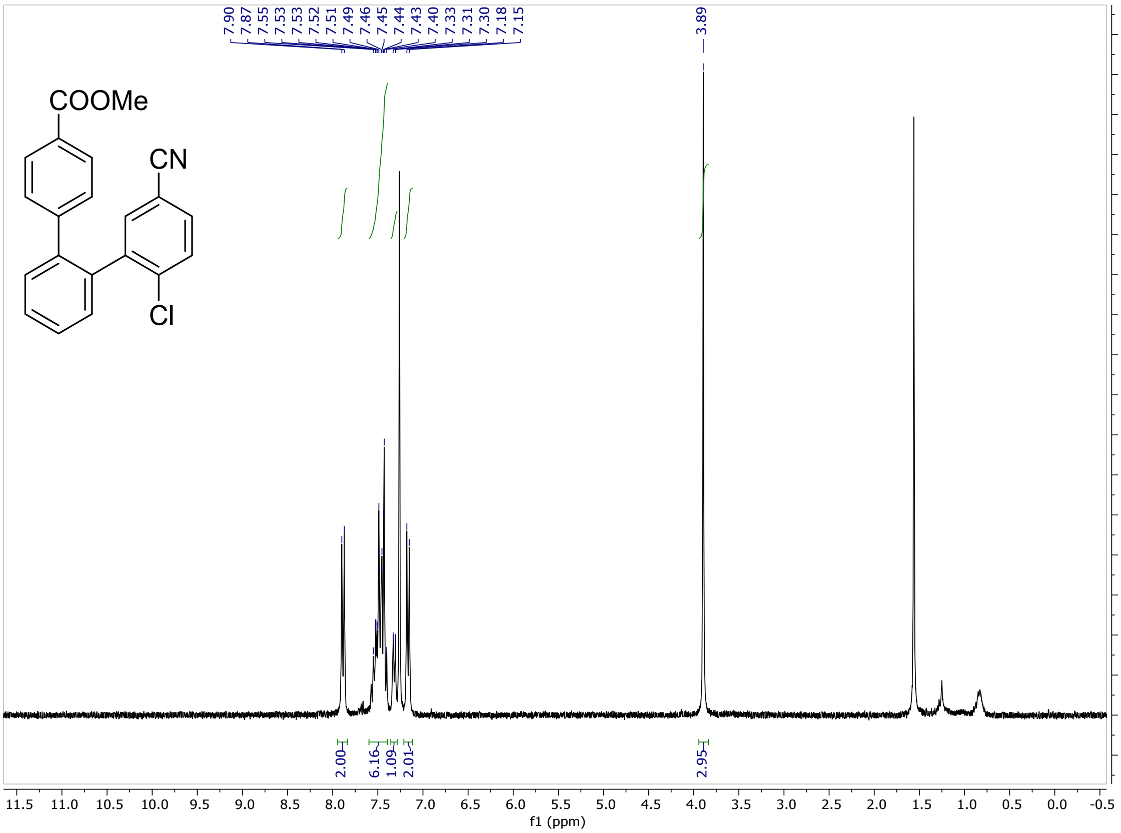

Figure S9. ^{1}H-NMR (300 MHz) spectrum of **3g** in $CDCl_3$.

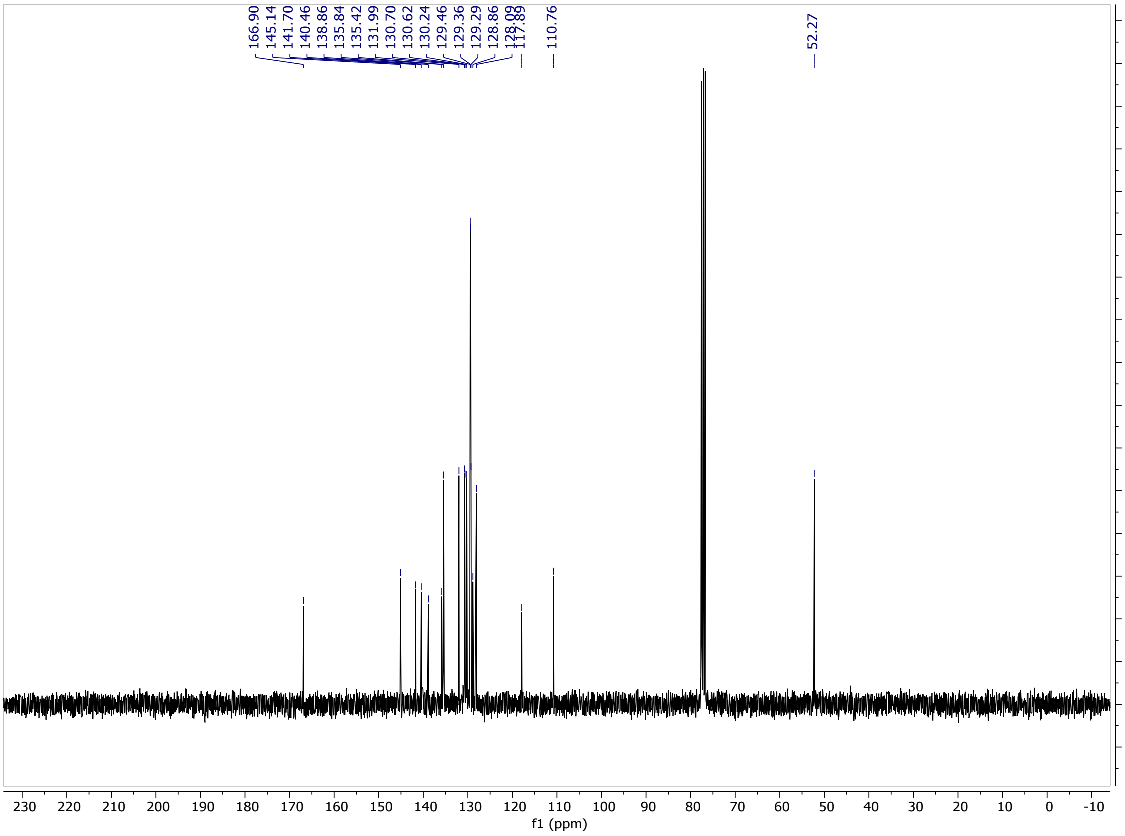

Figure S55. ^{13}C-NMR (75 MHz) spectrum of **3g** in $CDCl_3$.

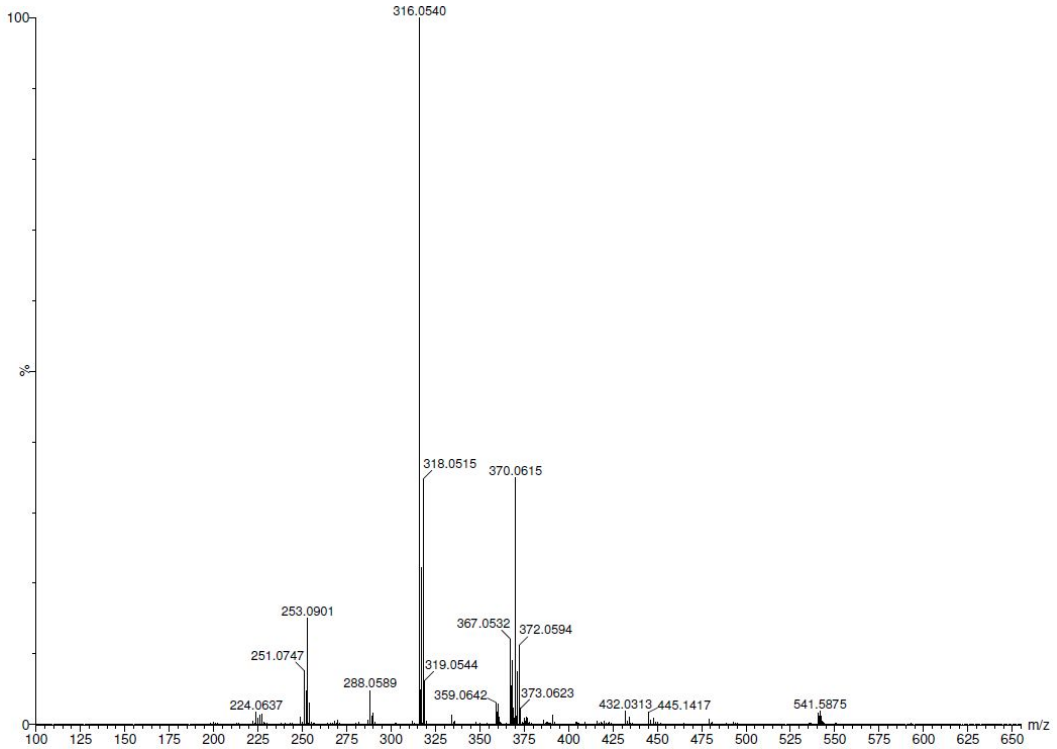

Figure S56. HRMS-APCI TOF mass spectrum of **3g**.

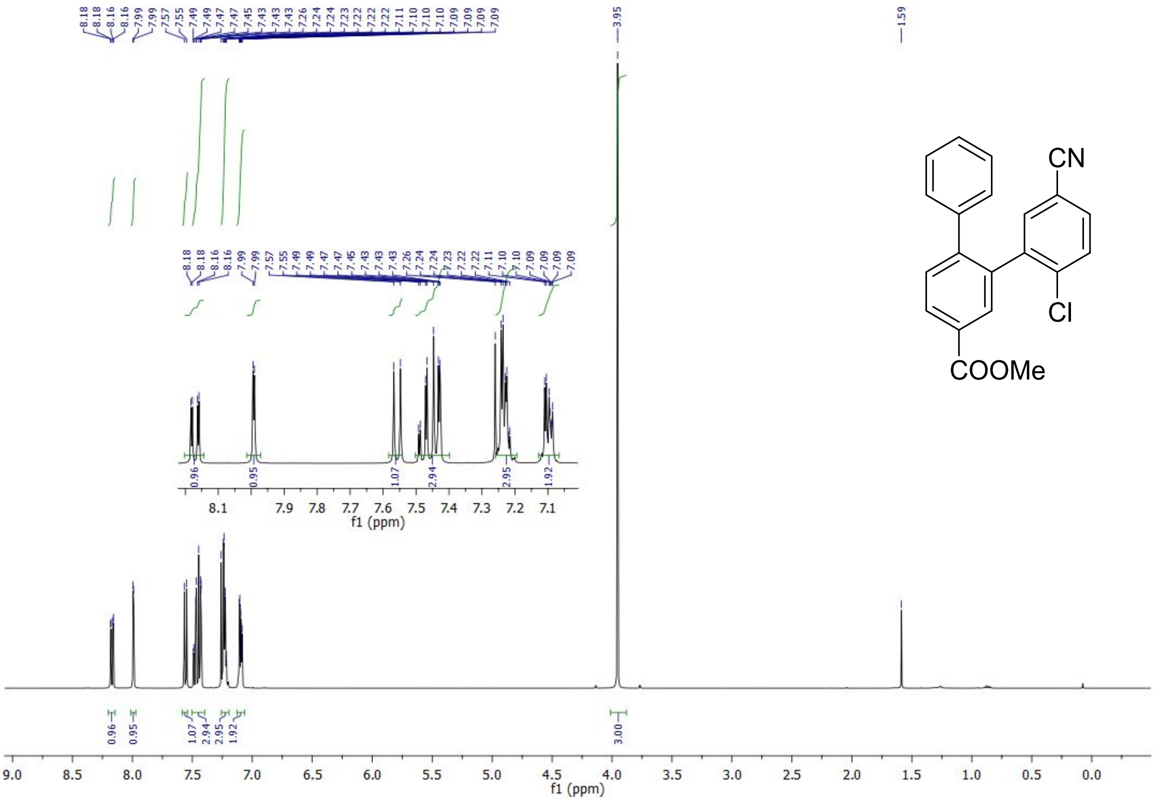

Figure S57. ^{1}H-NMR (600 MHz) spectrum of **3j** in $CDCl_3$.

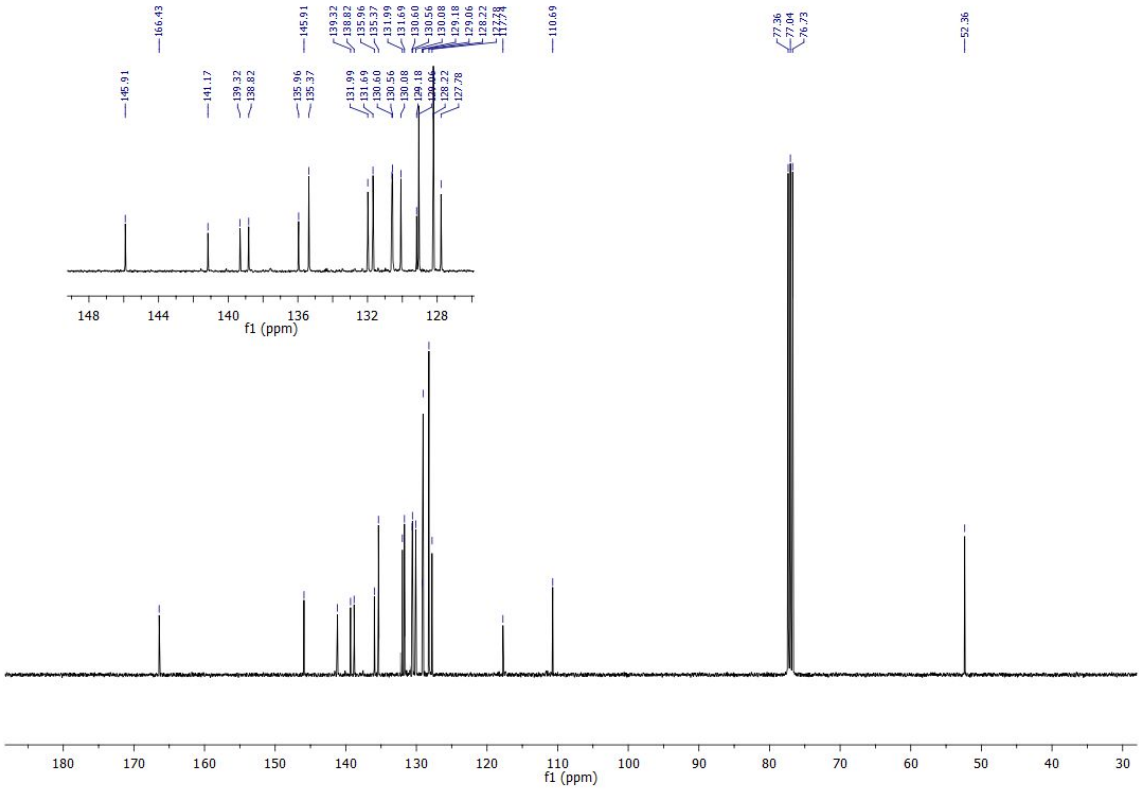

Figure S58. ^{13}C-NMR (151 MHz) spectrum of **3j** in $CDCl_3$.

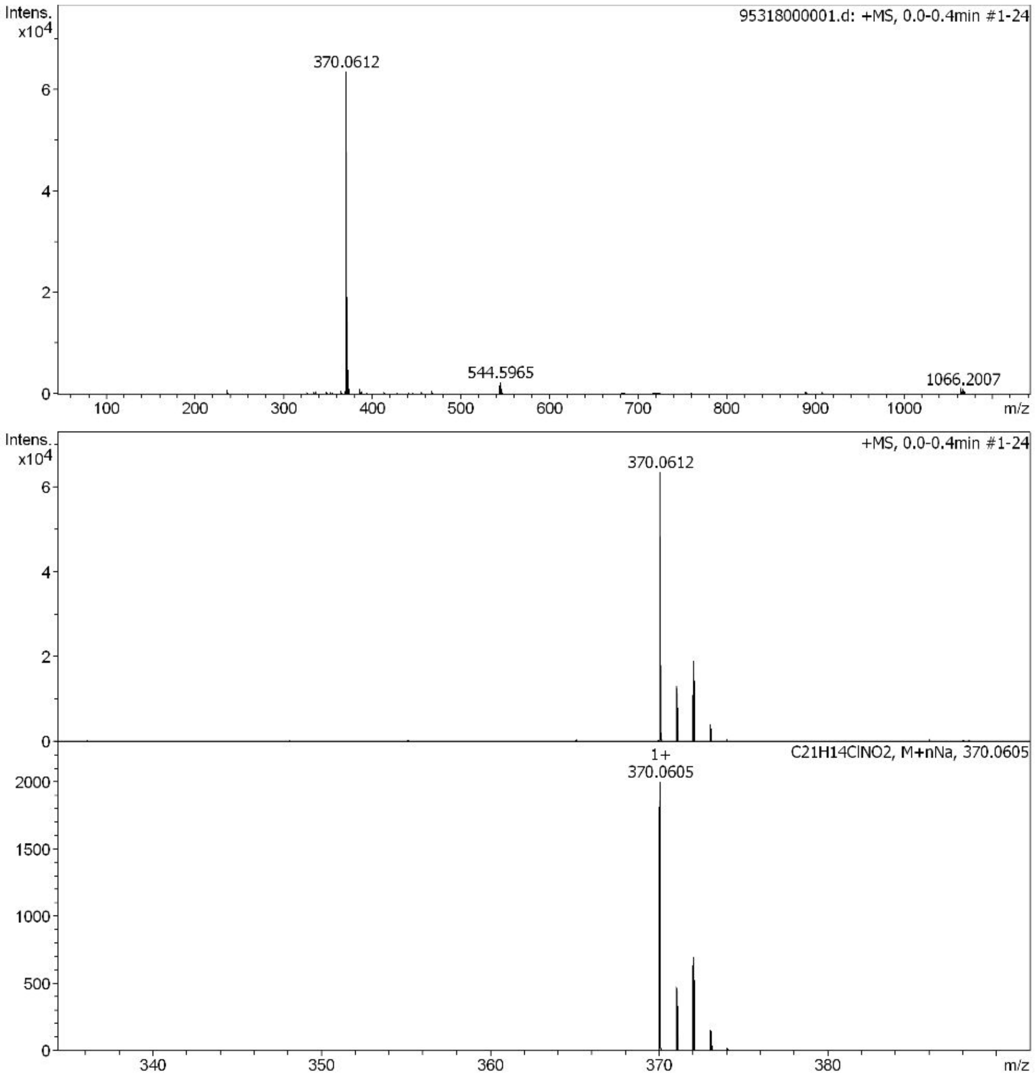

Figure S59. HRMS-ESI mass spectrum of **3j**.

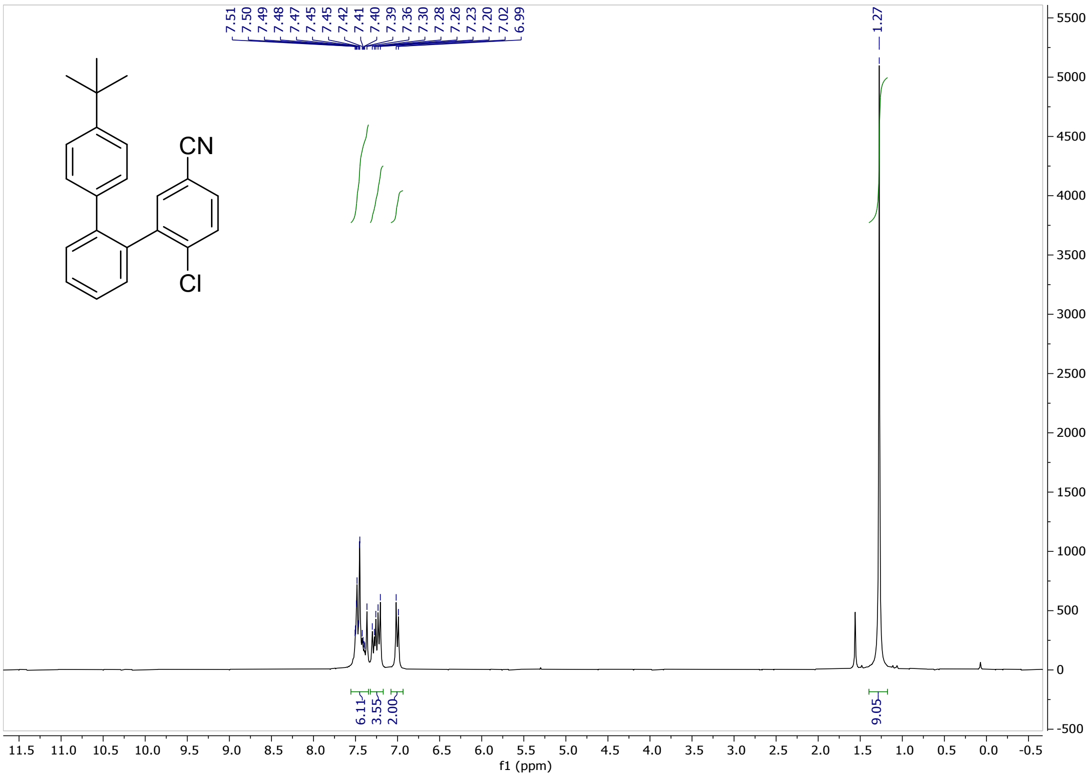

Figure S60. ^{1}H-NMR (300 MHz) spectrum of **3k** in $CDCl_3$.

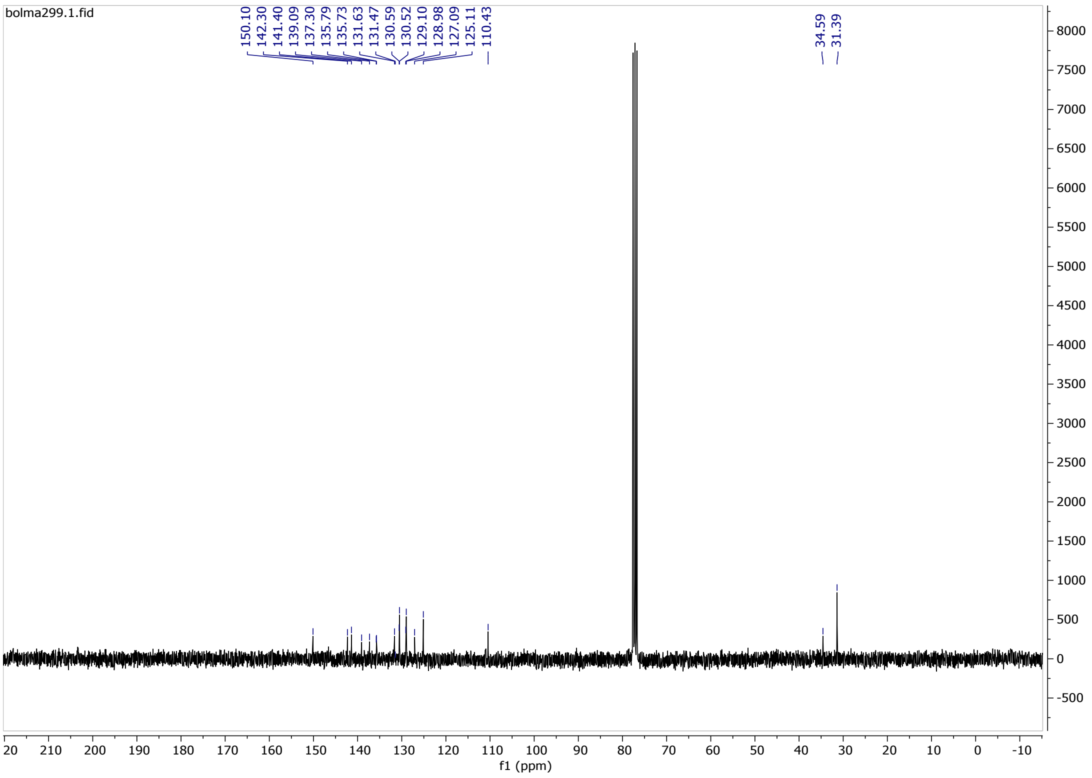

Figure S61. ^{13}C-NMR (75 MHz) spectrum of **3k** in $CDCl_3$.

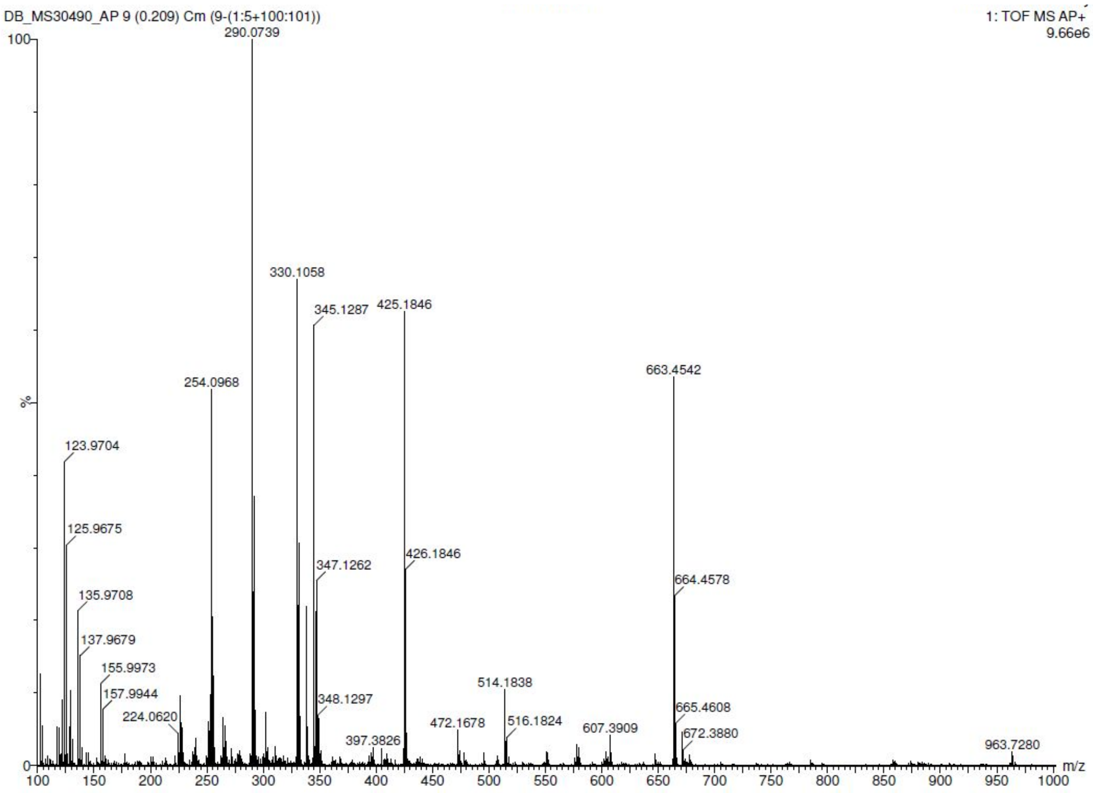

Figure S62. HRMS-APCI TOF mass spectrum of **3k**.

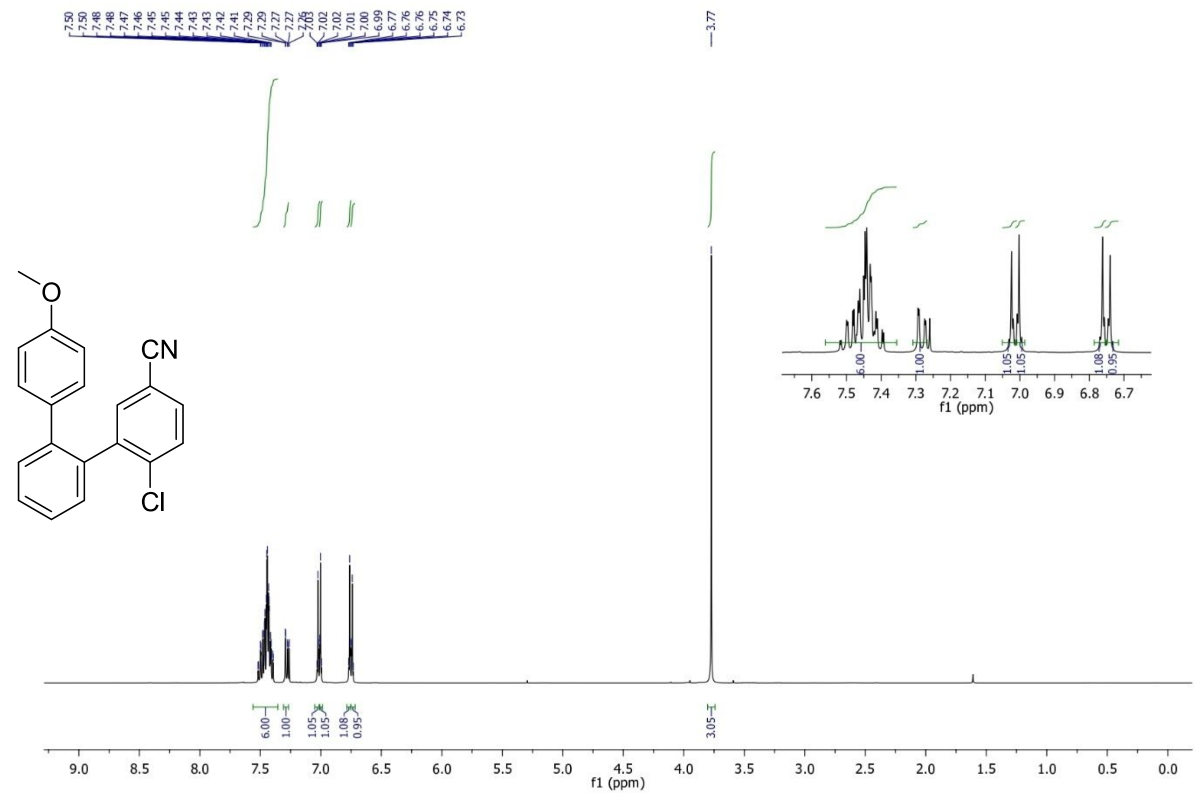

Figure S63. ^{1}H-NMR (600 MHz) spectrum of **3l** in $CDCl_3$.

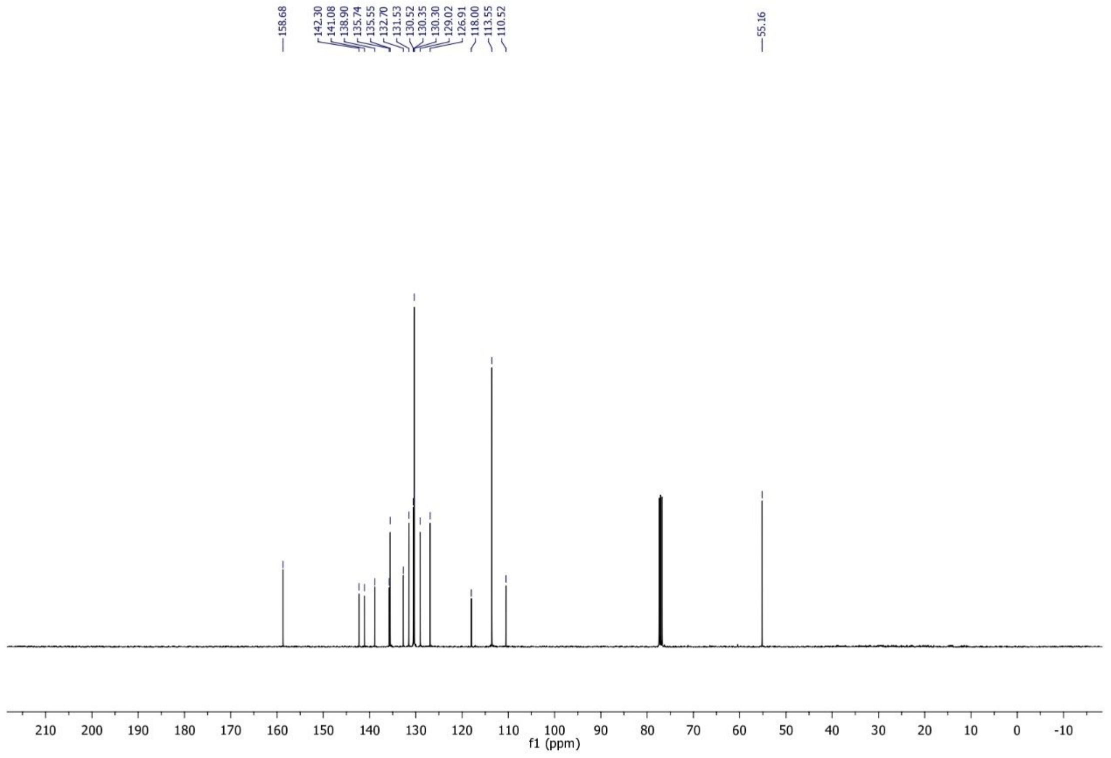

Figure S64. ^{13}C-NMR (151 MHz) spectrum of **3l** in $CDCl_3$.

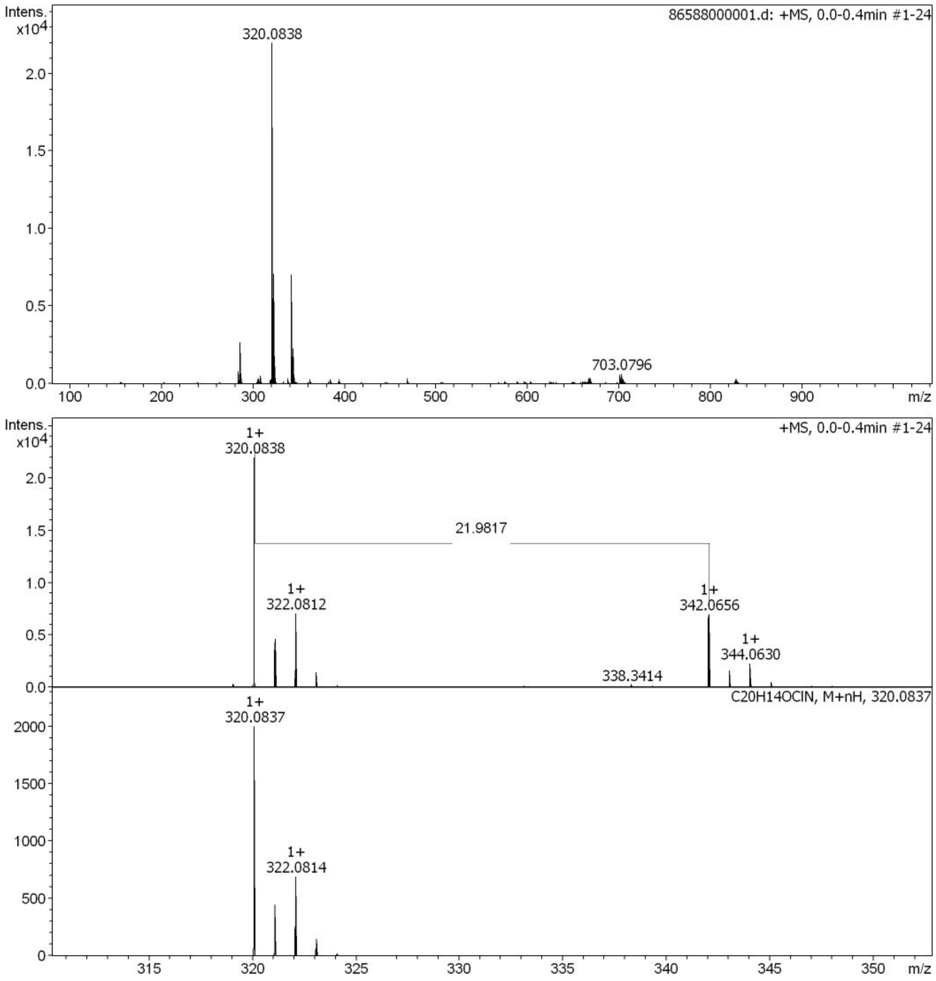

Figure S65. HRMS-ESI mass spectrum of **3l.**

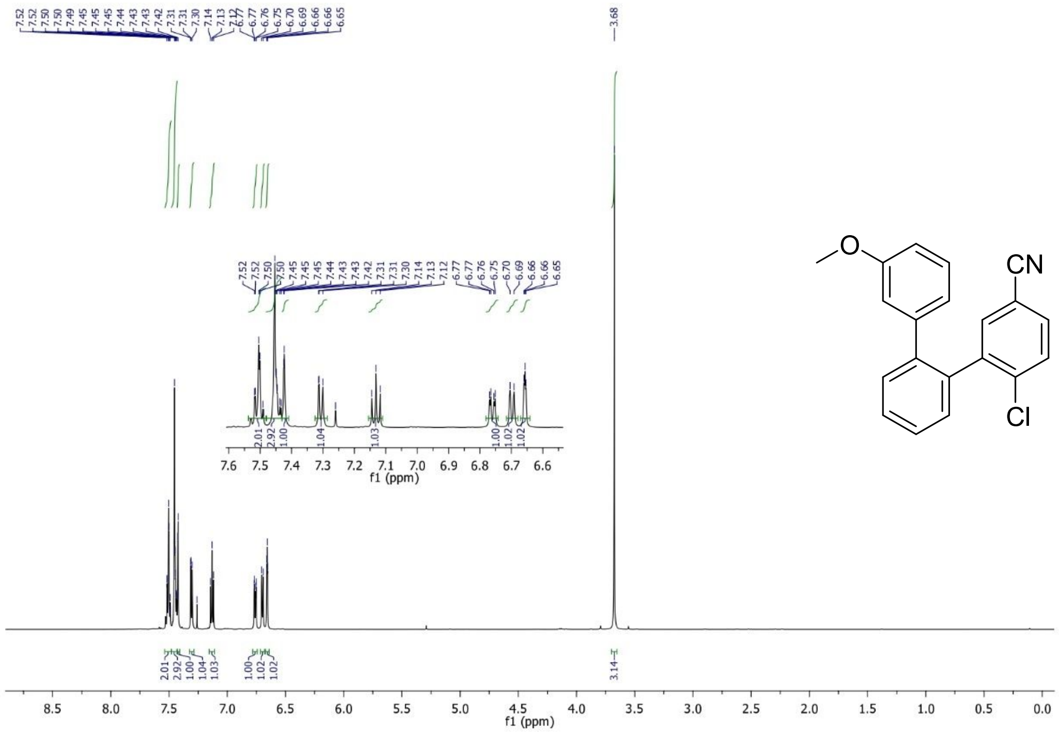

Figure S66. ^{1}H-NMR (600 MHz) spectrum of **3m** in $CDCl_3$.

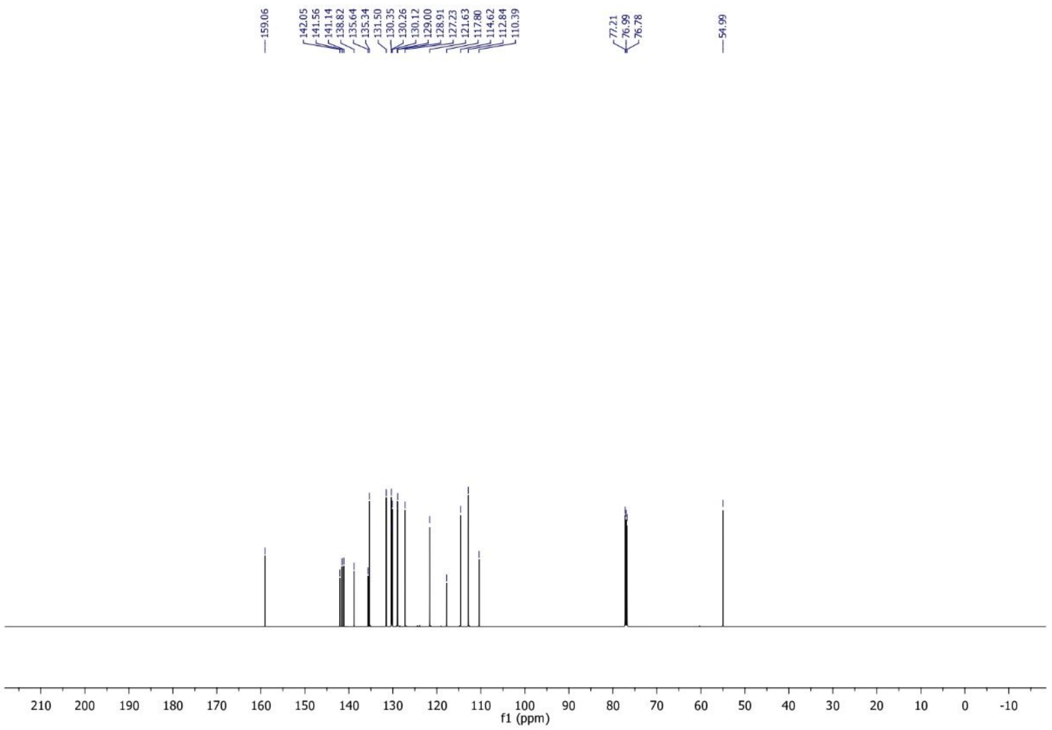

Figure S67. ^{13}C-NMR (151 MHz) spectrum of **3m** in $CDCl_3$.

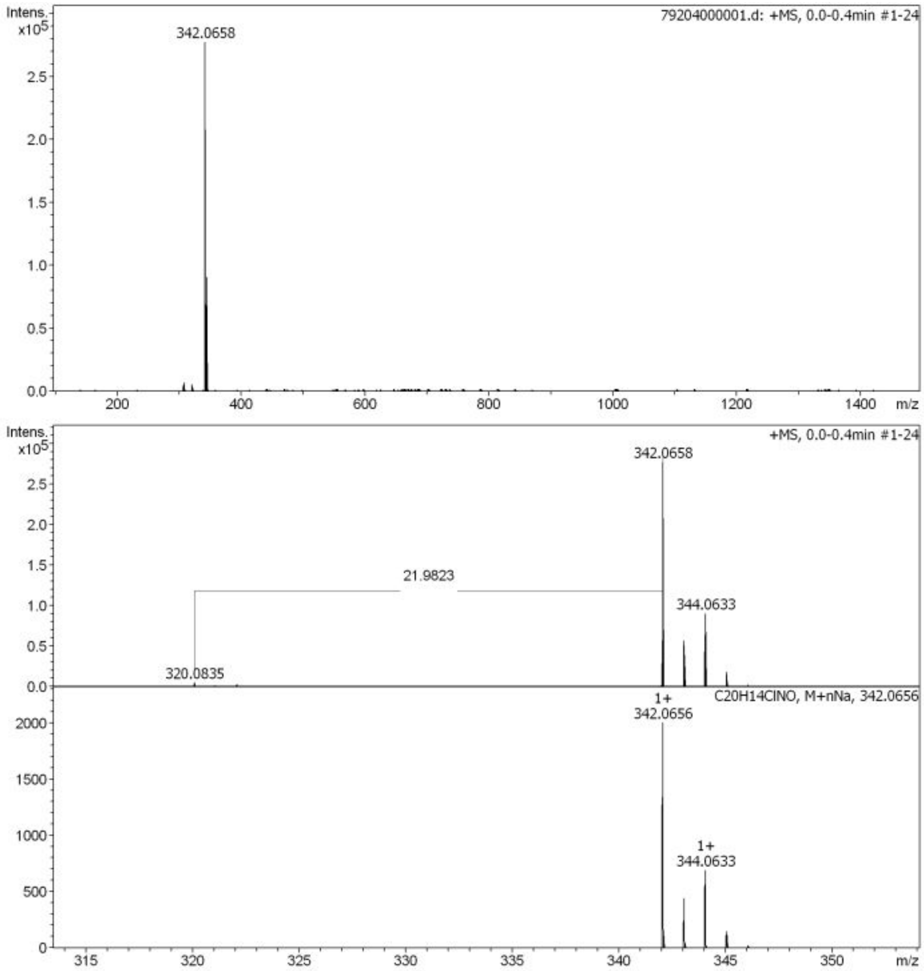

Figure S68. HRMS-ESI mass spectrum of **3m.**

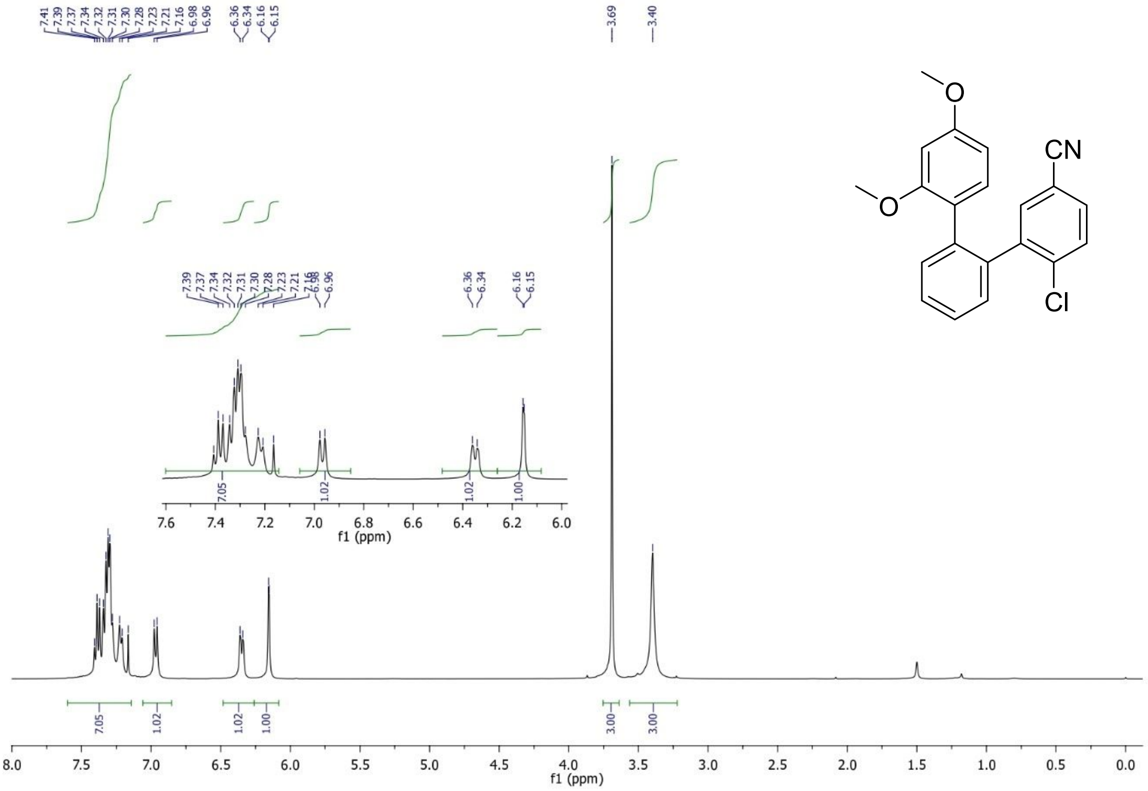

Figure S69. ^{1}H-NMR (600 MHz) spectrum of **3n** in $CDCl_3$.

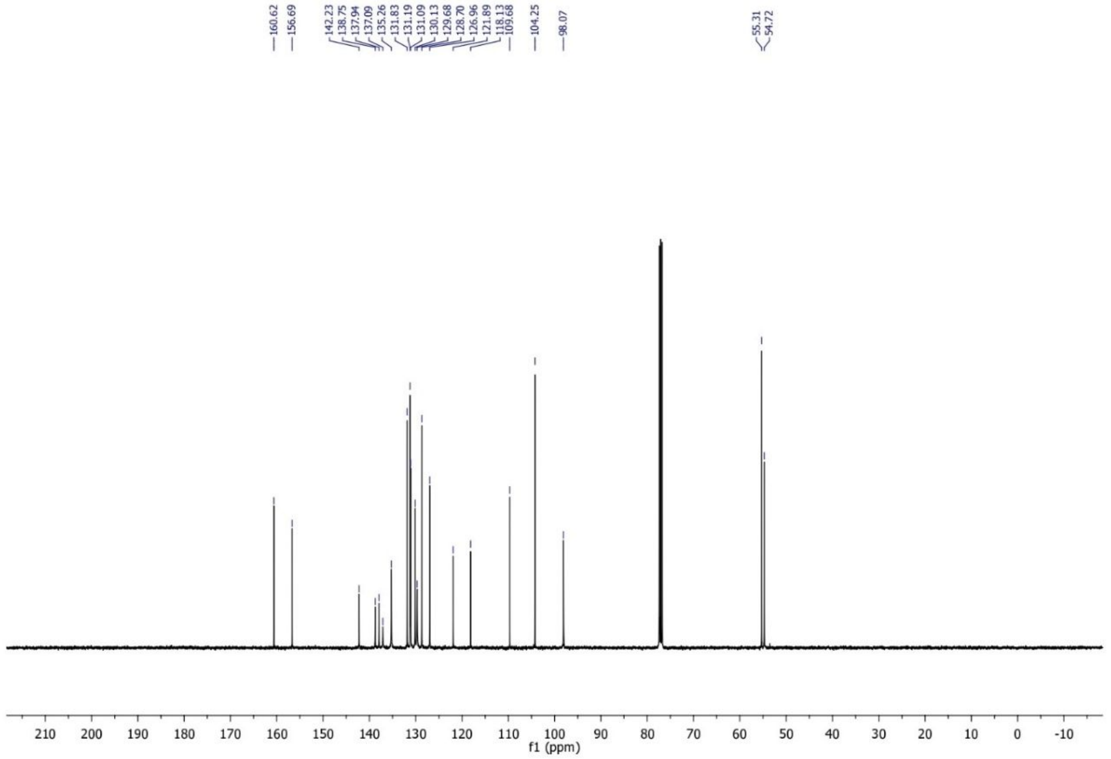

Figure S70. ^{13}C-NMR (151 MHz) spectrum of **3n** in $CDCl_3$.

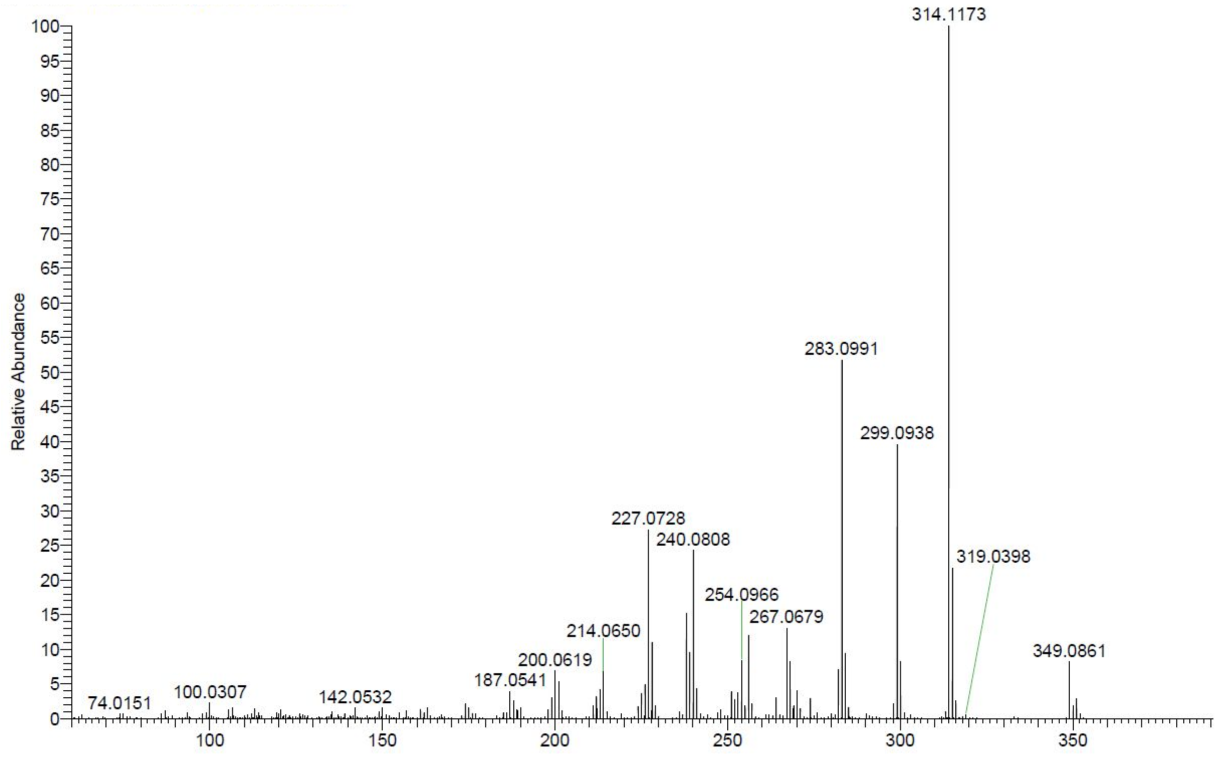

Figure S71. HRMS-ESI mass spectrum of **3n.**

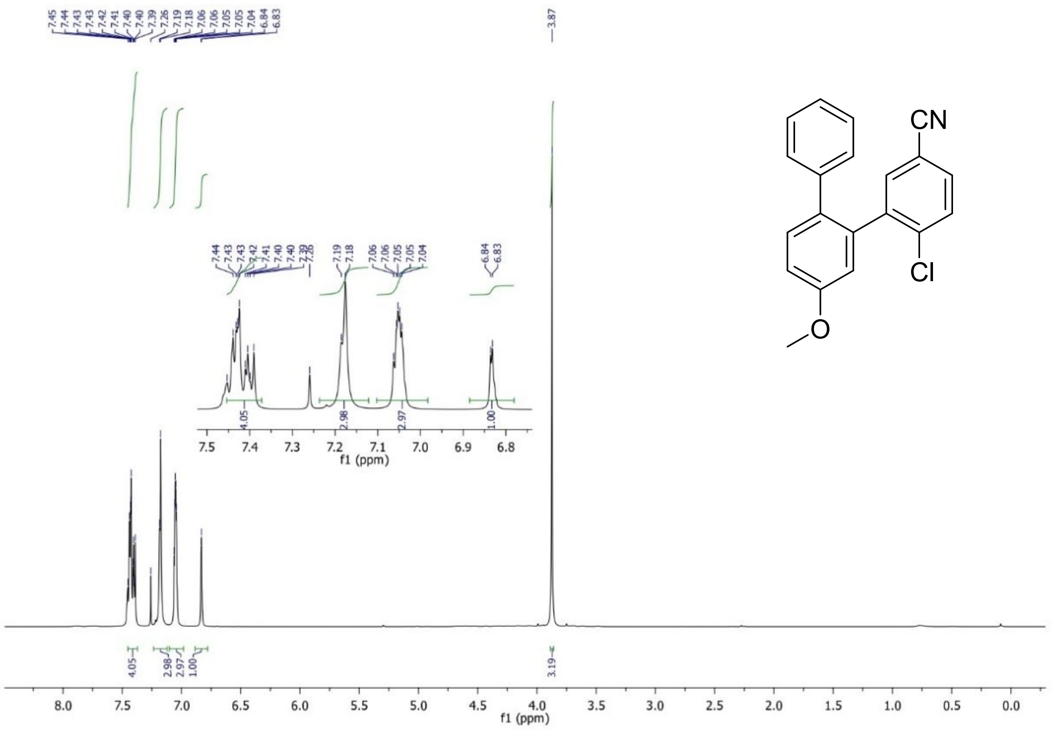

Figure S72. ^{1}H-NMR (600 MHz) spectrum of **3o** in $CDCl_3$.

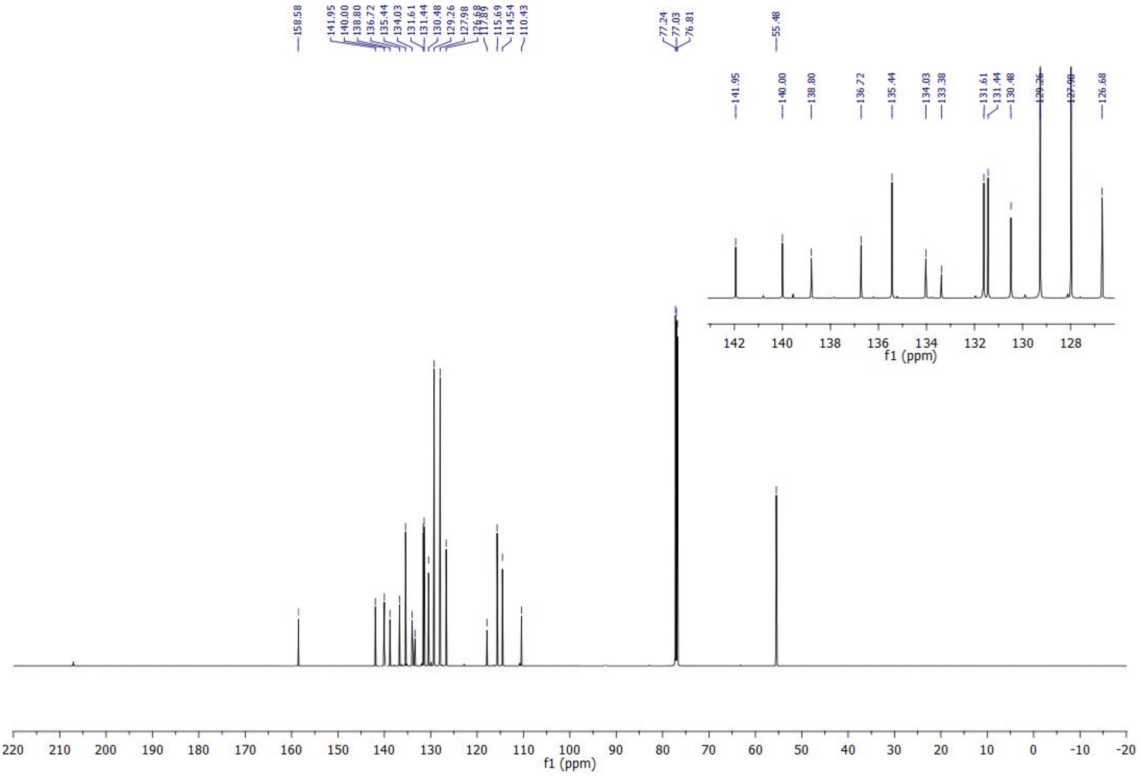

Figure S73. ^{13}C-NMR (151 MHz) spectrum of **3o** in $CDCl_3$.

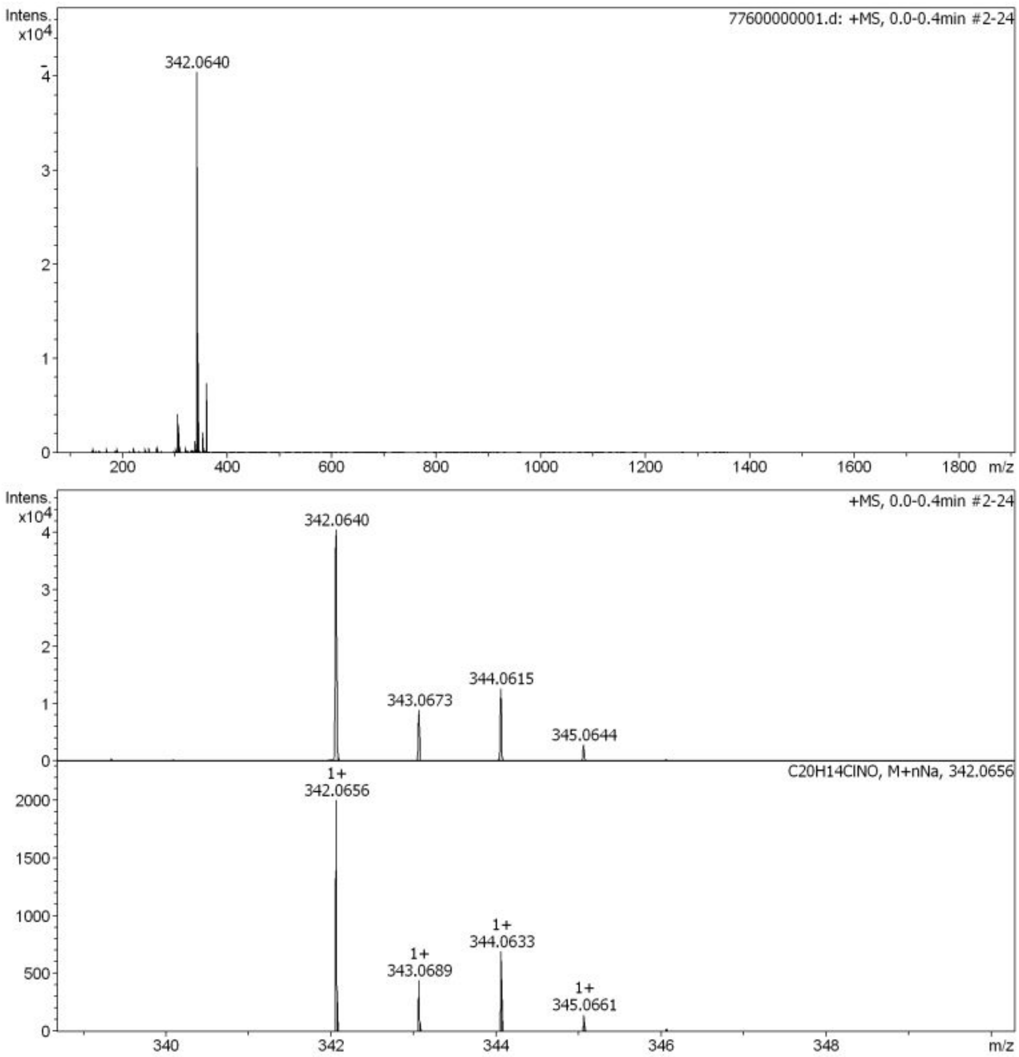

Figure S74. HRMS-ESI mass spectrum of **3o.**

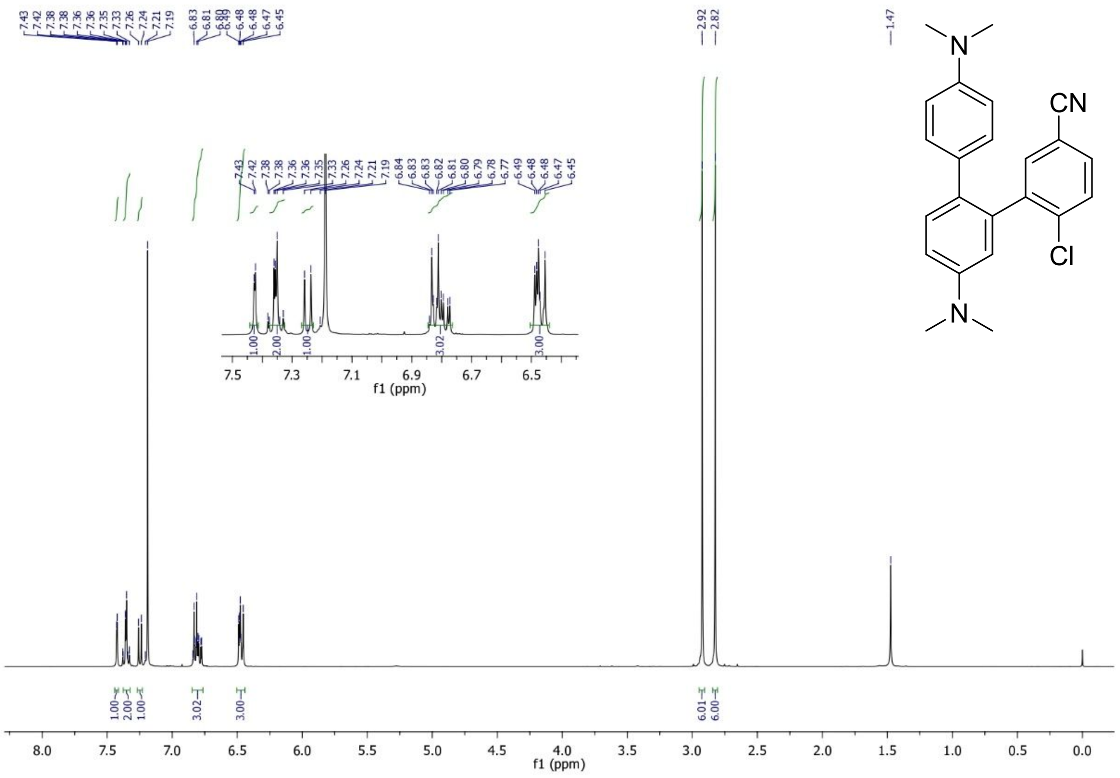

Figure S75. ^{1}H-NMR (600 MHz) spectrum of **3p** in $CDCl_3$.

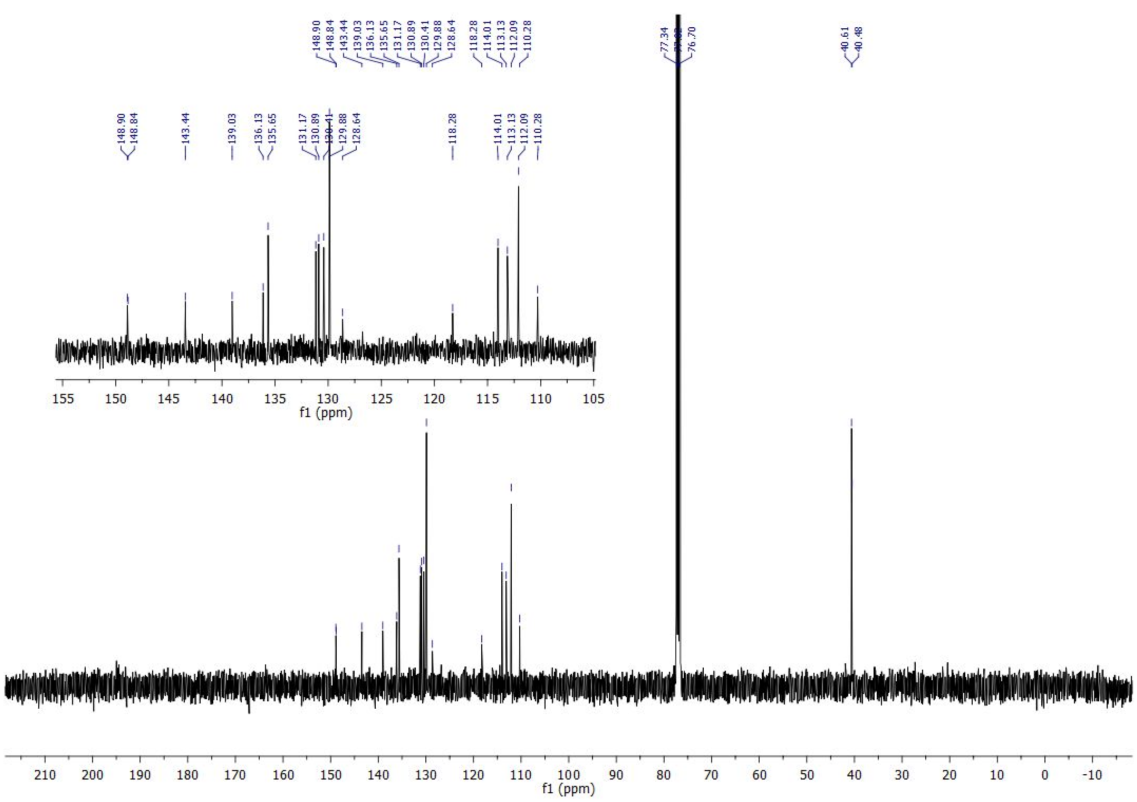

Figure S76. ^{13}C-NMR (151 MHz) spectrum of **3p** in $CDCl_3$.

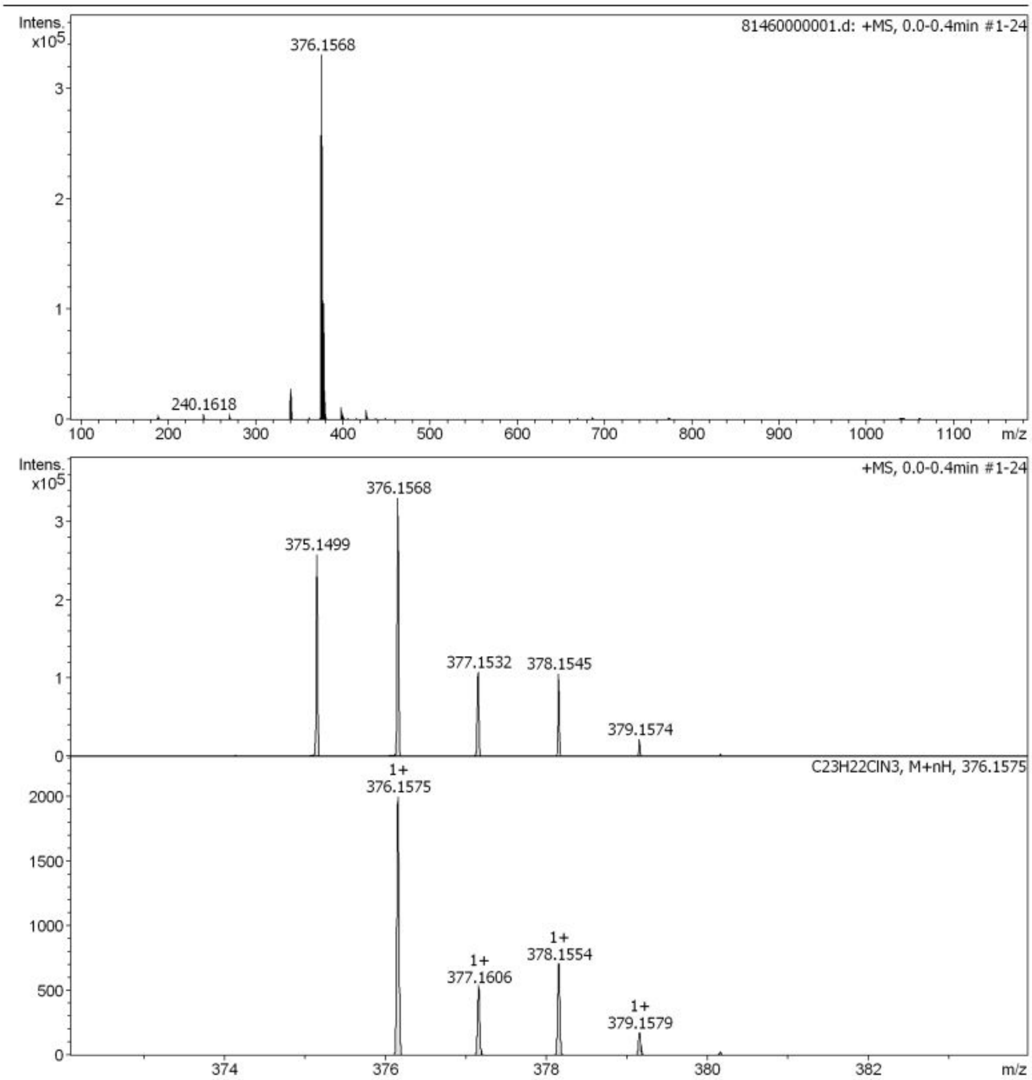

Figure S77. HRMS-ESI mass spectrum of **3p.**

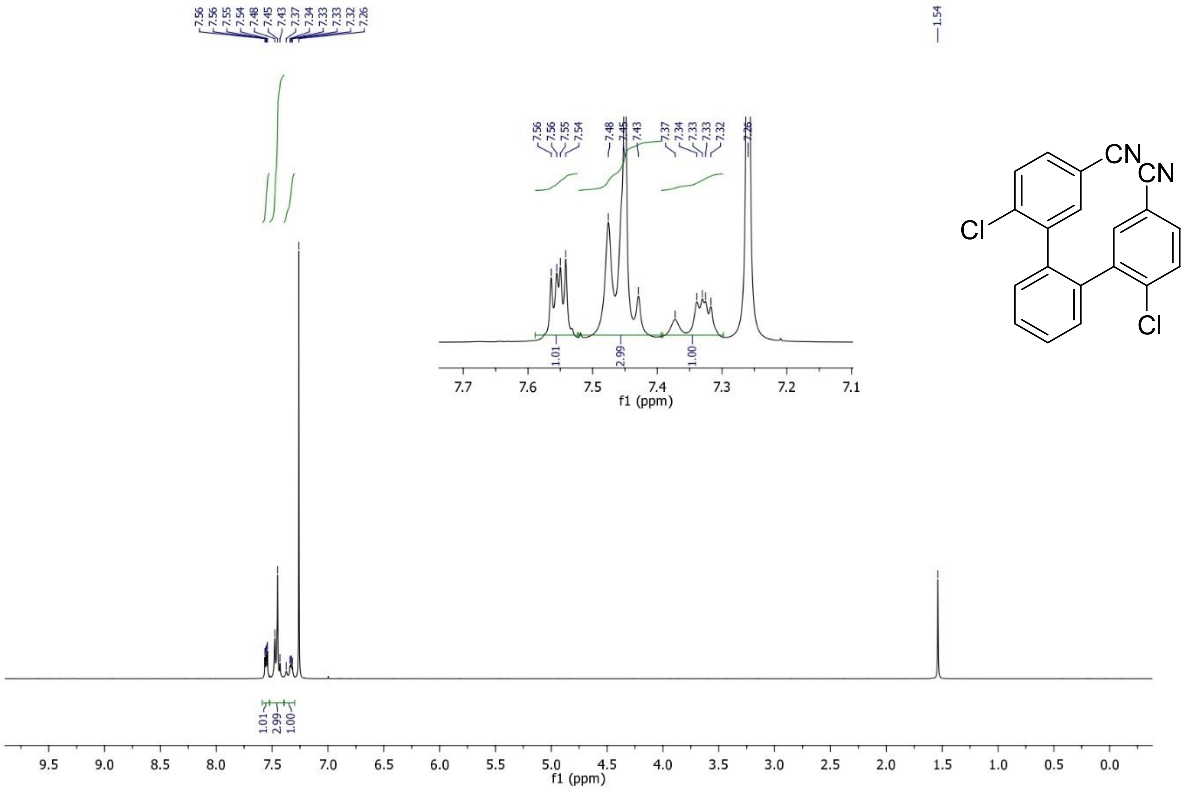

Figure S78. ^{1}H-NMR (600 MHz) spectrum of **3q** in $CDCl_3$.

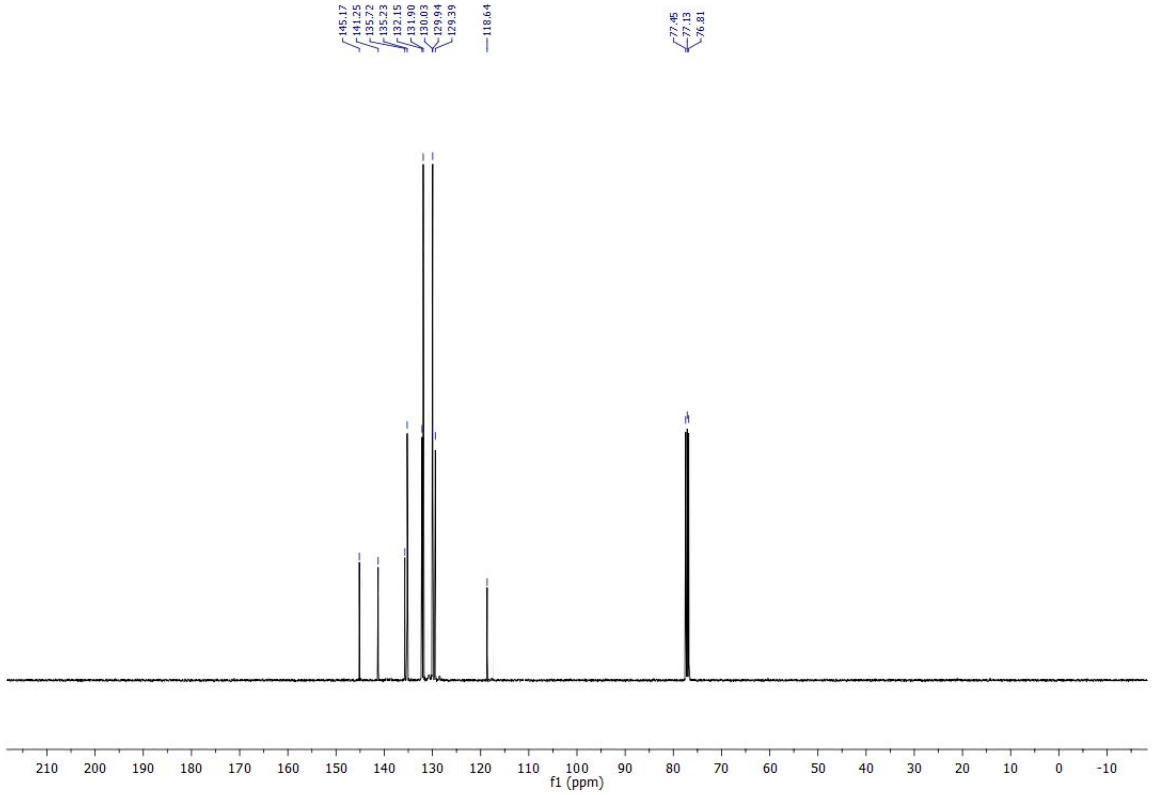

Figure S79. ^{13}C-NMR (151 MHz) spectrum of **3q** in $CDCl_3$.

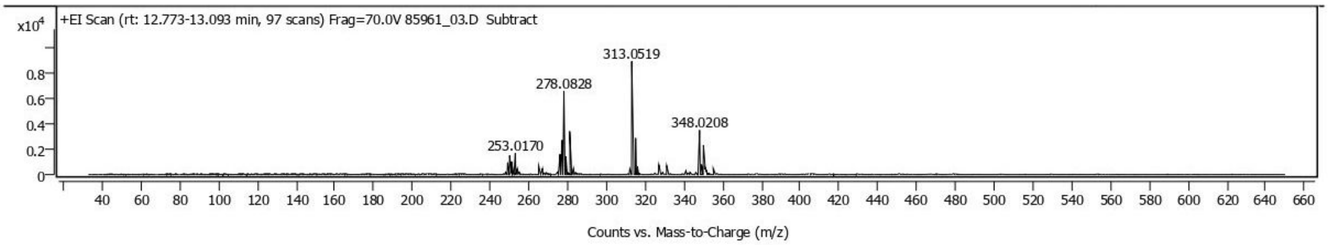

Figure S80. HRGC-MS mass spectrum of **3q**.

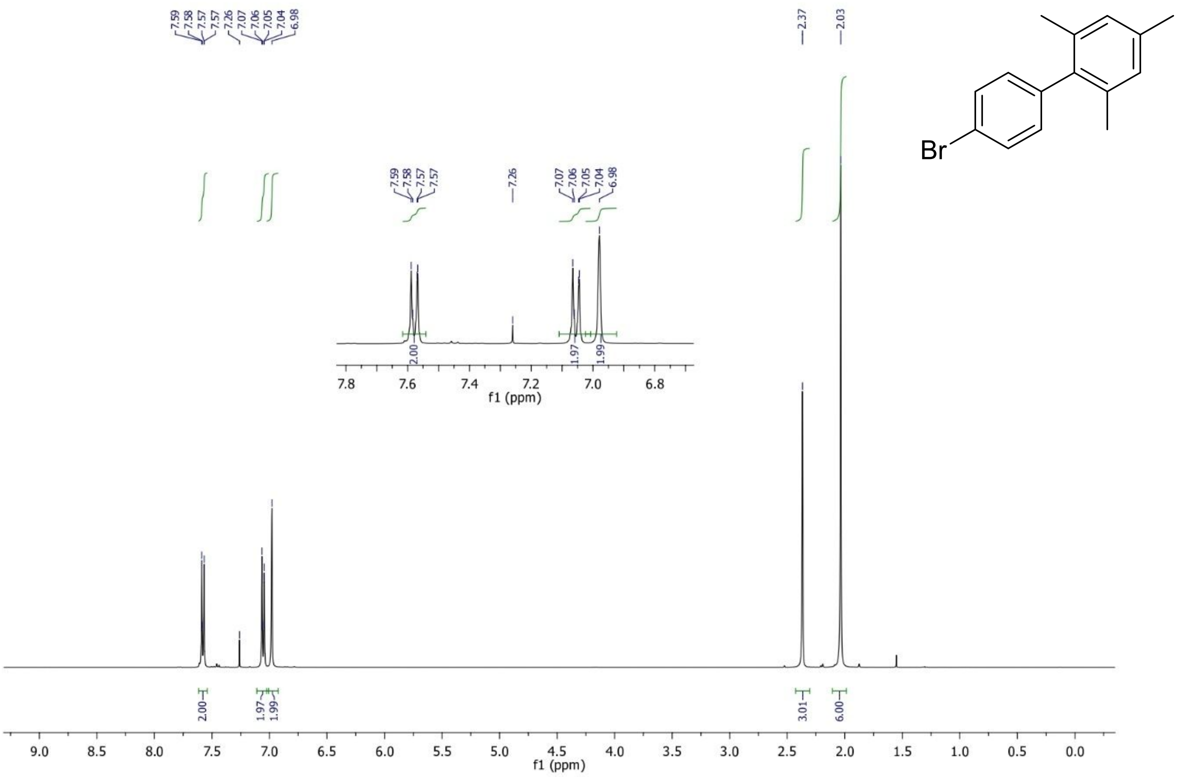

Figure S81. ^{1}H-NMR (600 MHz) spectrum of **4'-bromo-2,4,6-trimethyl-1,1'-biphenyl** in $CDCl_3$.

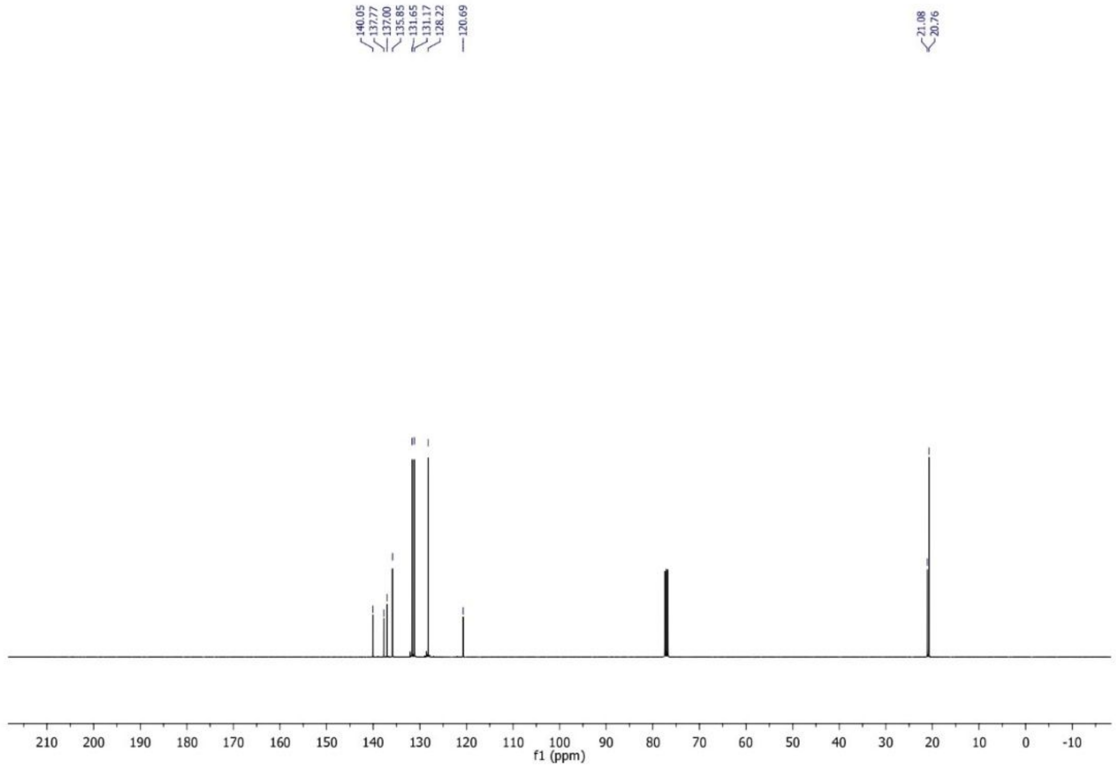

Figure S82. ^{13}C-NMR (151 MHz) spectrum of **4'-bromo-2,4,6-trimethyl-1,1'-biphenyl** in $CDCl_3$.

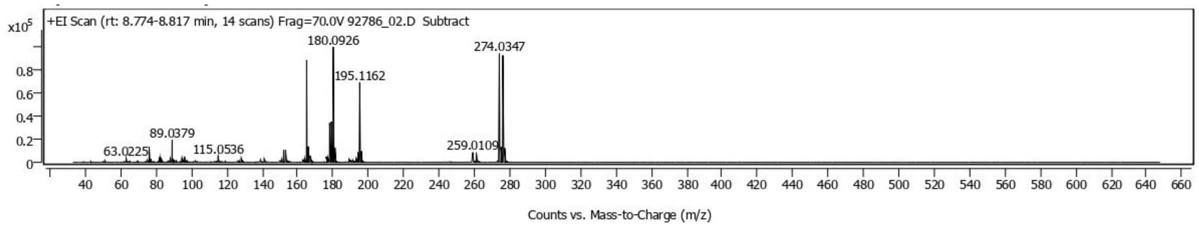

Figure S83. HRGC-MS mass spectrum of **4'-bromo-2,4,6-trimethyl-1,1'-biphenyl.**

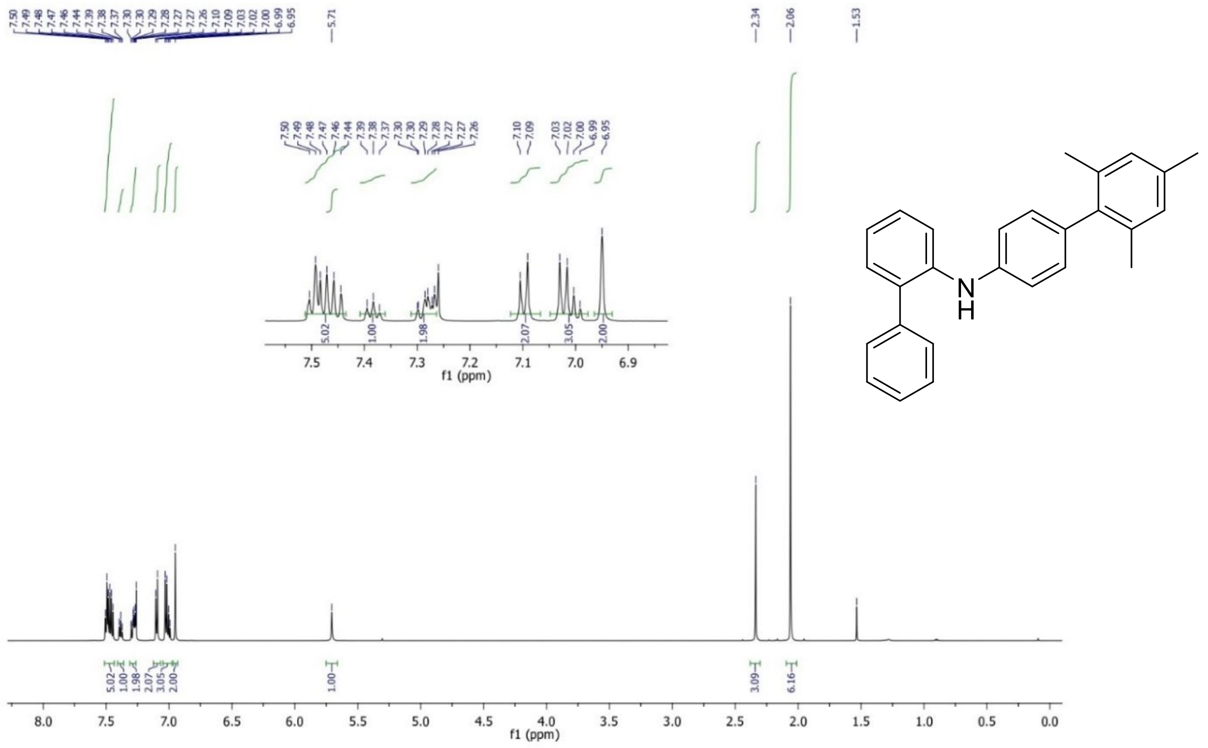

Figure S84. ^{1}H-NMR (600 MHz) spectrum of **N-(2',4',6'-trimethyl-[1,1'-biphenyl]-4-yl)-[1,1'-biphenyl]-2-amine** in $CDCl_3$.

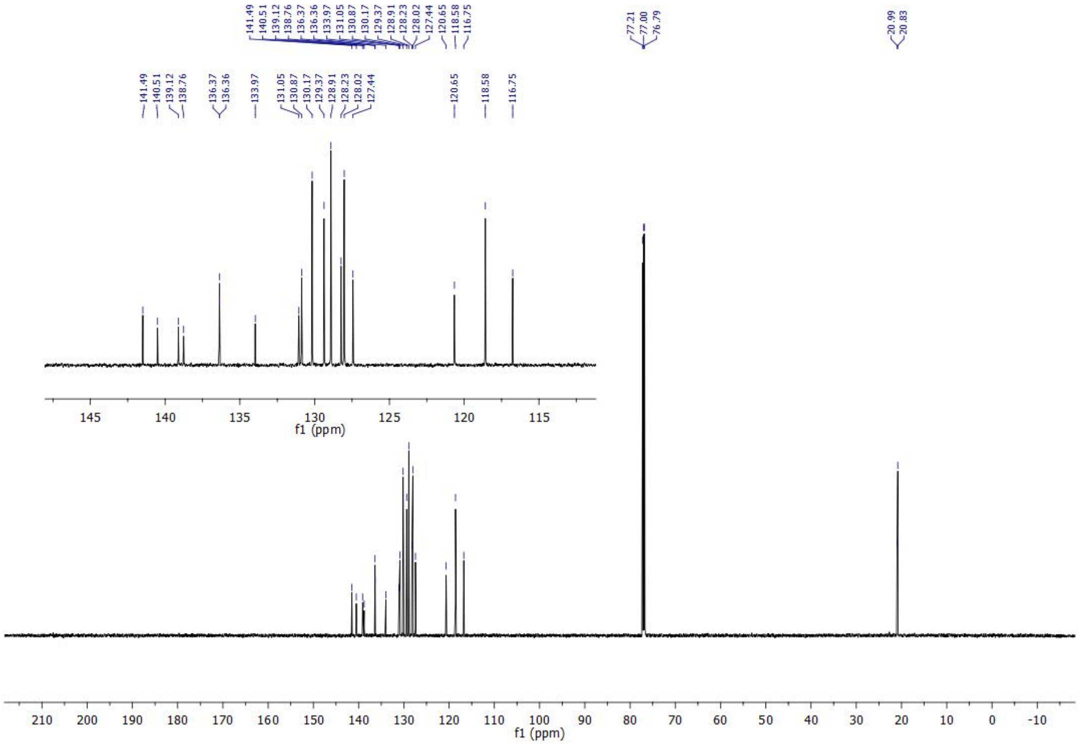

Figure S85. ^{13}C-NMR (151 MHz) spectrum of **N-(2',4',6'-trimethyl-[1,1'-biphenyl]-4-yl)-[1,1'-biphenyl]-2-amine** in $CDCl_3$.

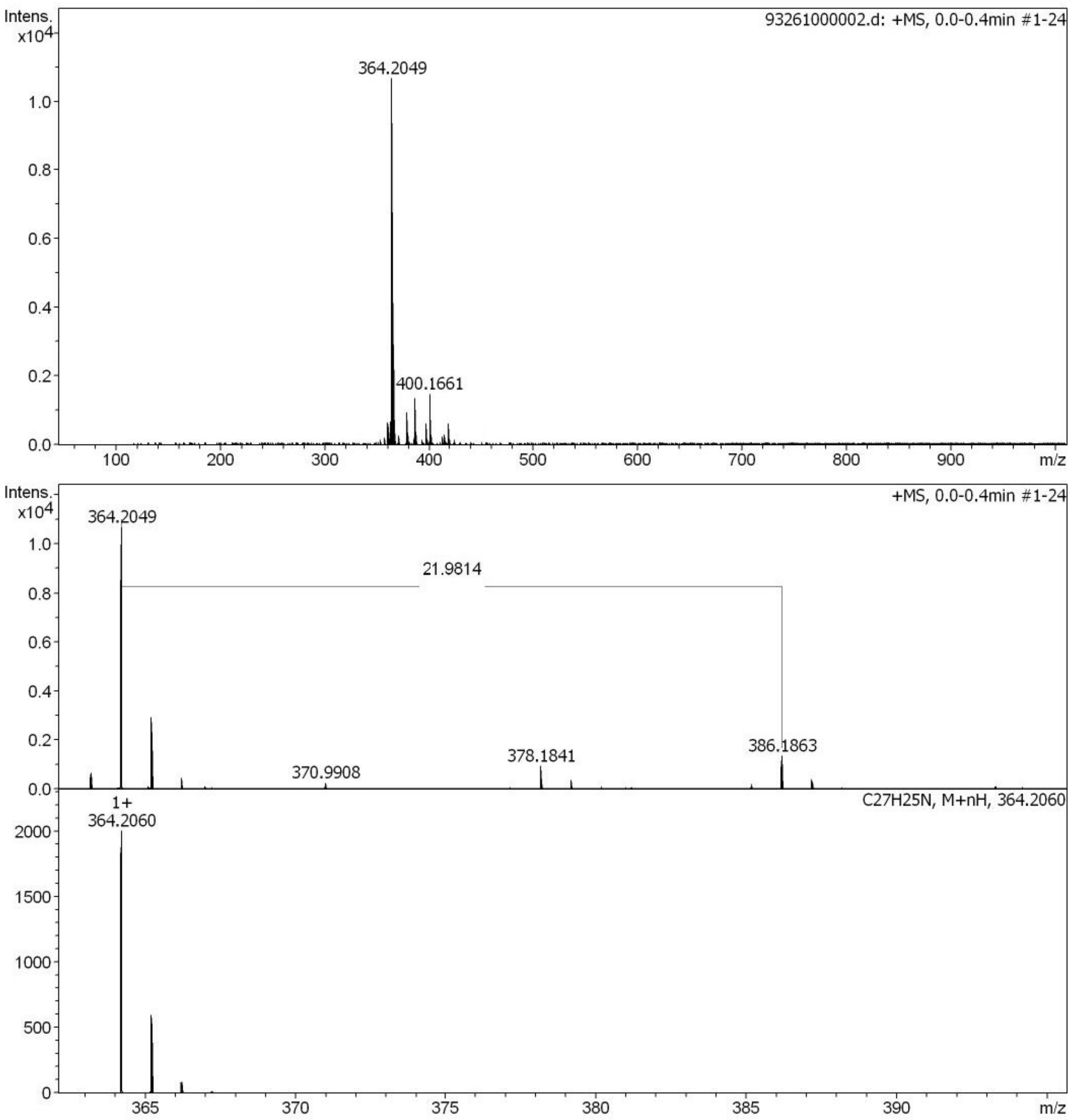

Figure S86. HRMS-Maldi-TOF mass spectrum of **N-(2',4',6'-trimethyl-[1,1'-biphenyl]-4-yl)-[1,1'-biphenyl]-2-amine.**

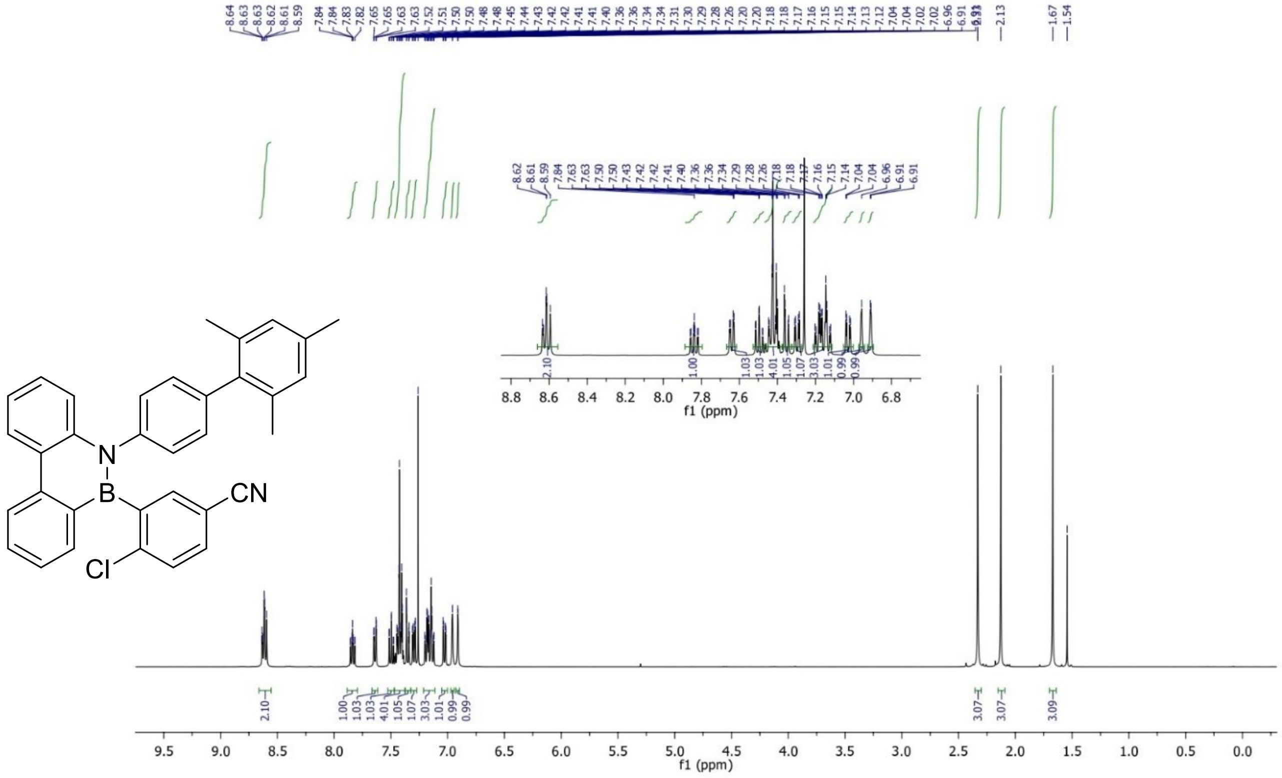

Figure S87. ^{1}H-NMR (600 MHz) spectrum of **3r** in $CDCl_3$.

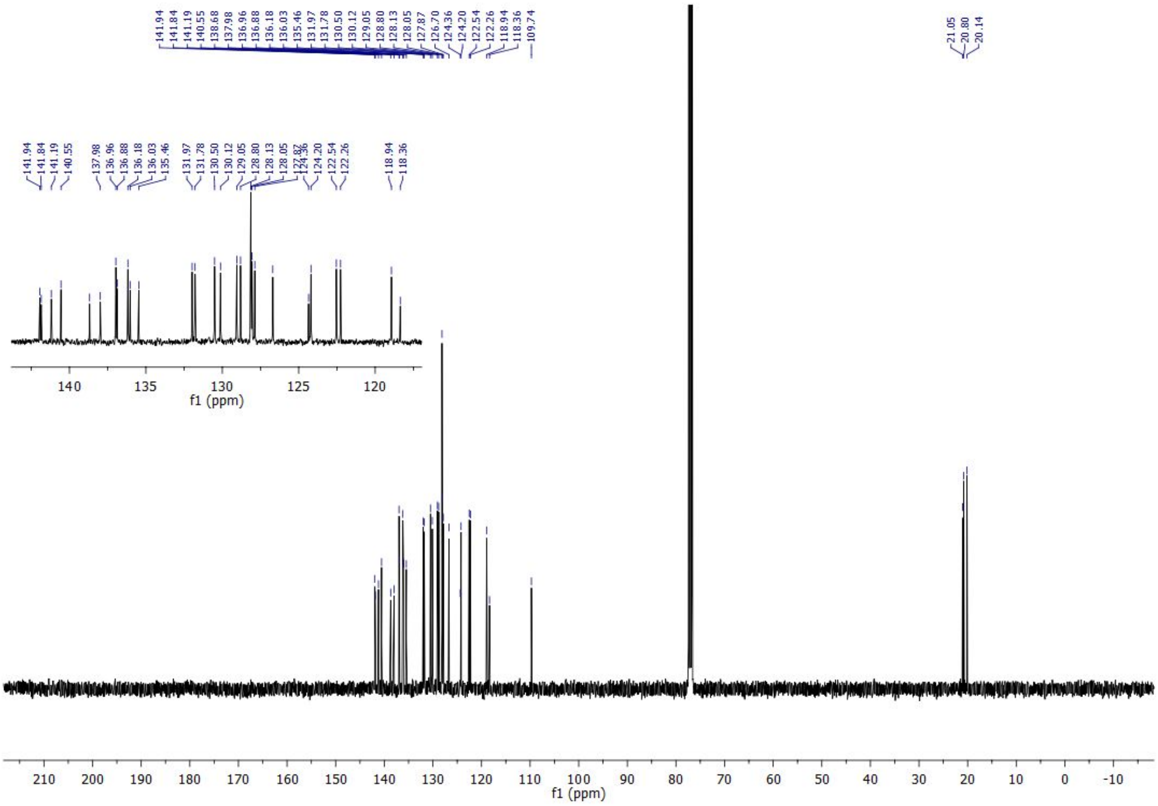

Figure S88. ^{13}C-NMR (151 MHz) spectrum of **3r** in $CDCl_3$.

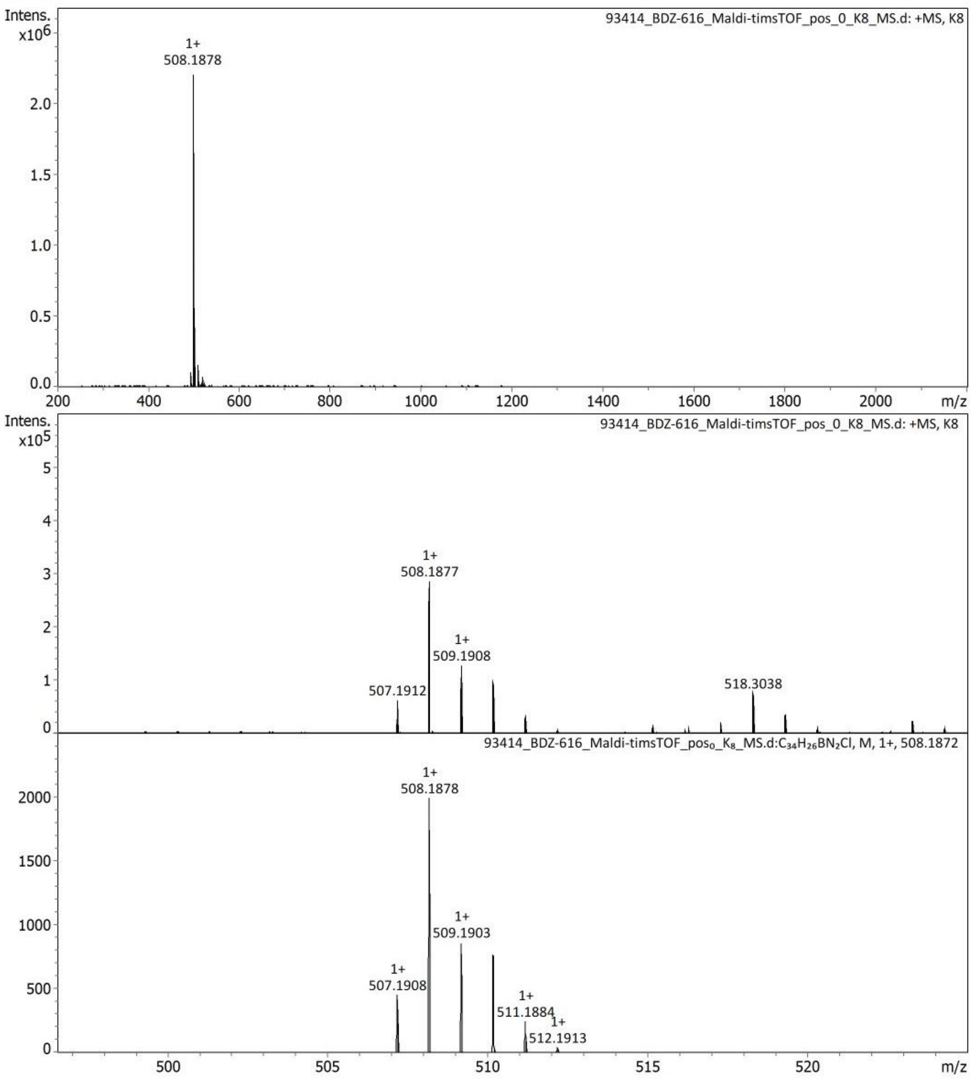

Figure S89. HRMS-Maldi TOF mass spectrum of **3r.**

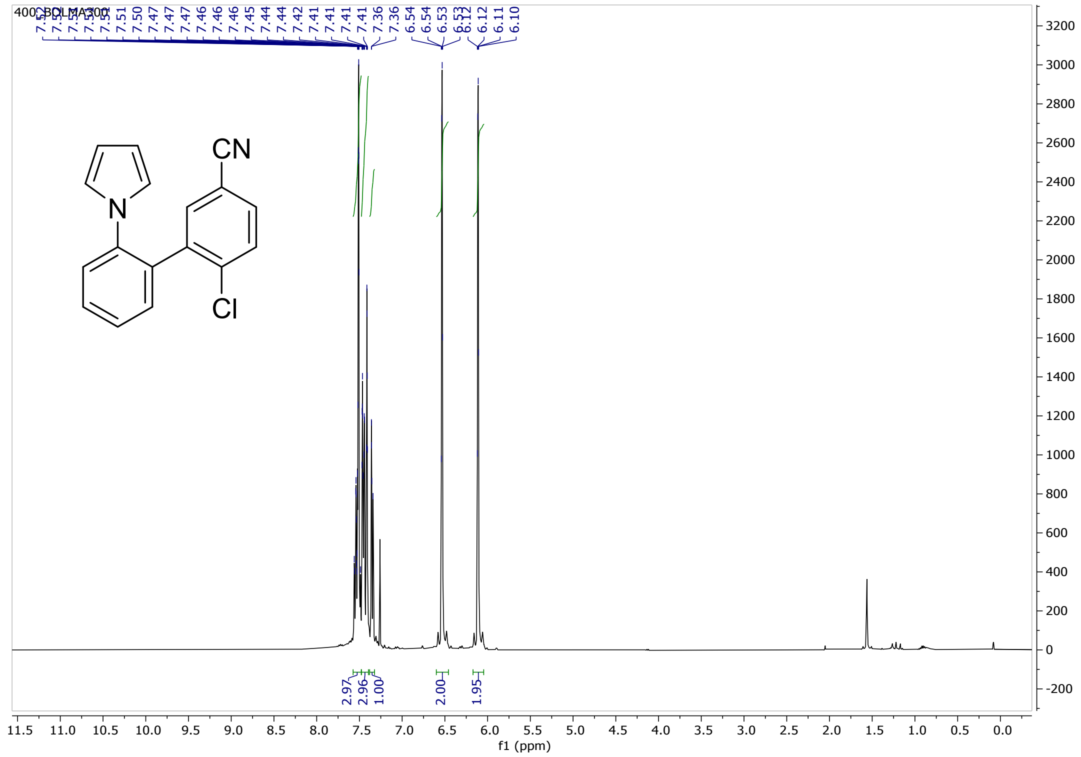

Figure S90. ^{1}H-NMR (300 MHz) spectrum of **5a** in $CDCl_3$.

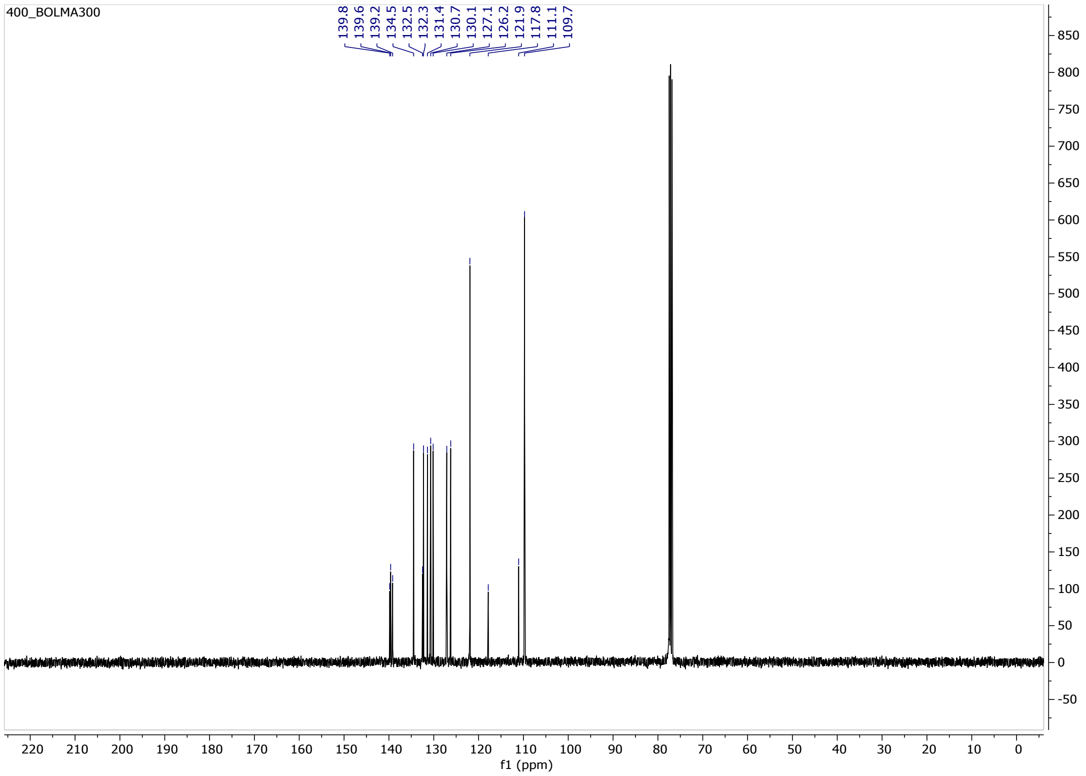

Figure S91. ^{13}C-NMR (75 MHz) spectrum of **5a** in $CDCl_3$.

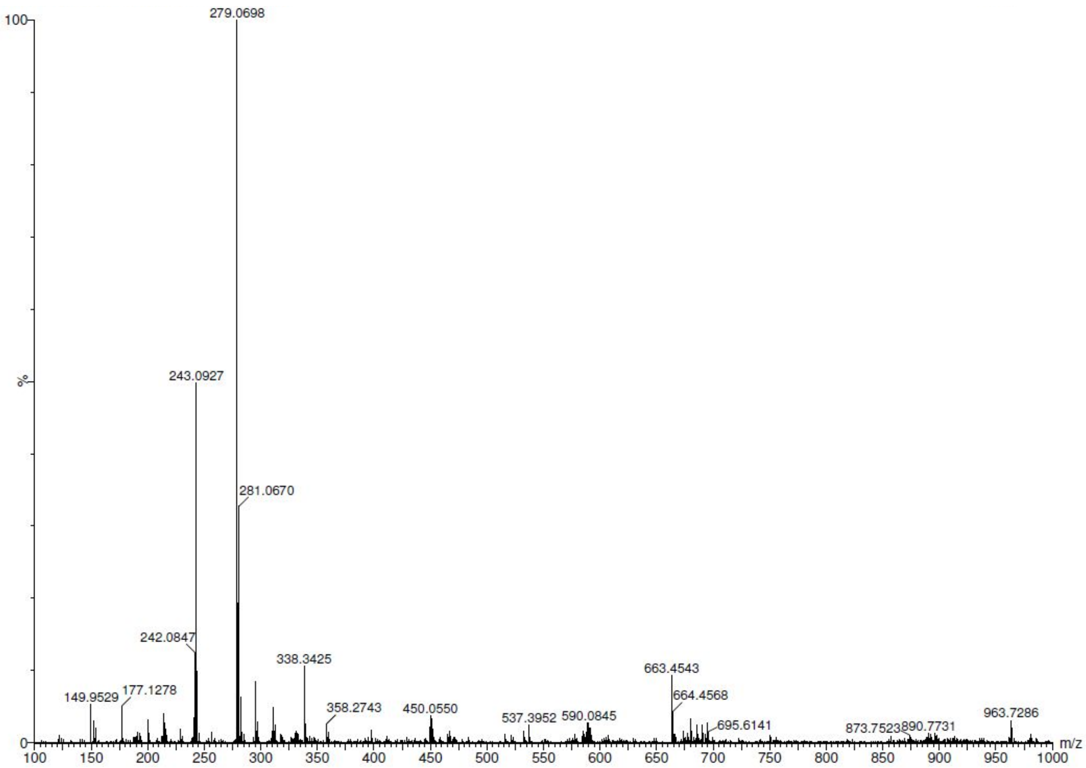

Figure S92. HRMS- APCI TOF mass spectrum of **5a**.

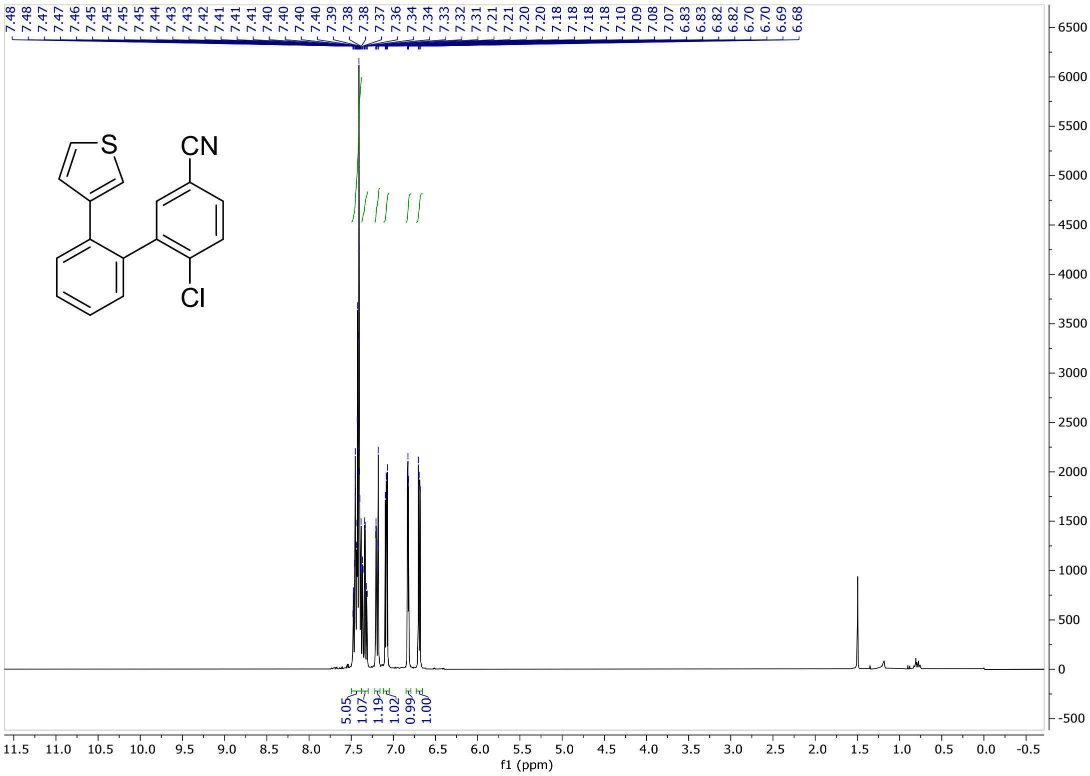

Figure S93. ^{1}H-NMR (300 MHz) spectrum of **5b** in $CDCl_3$.

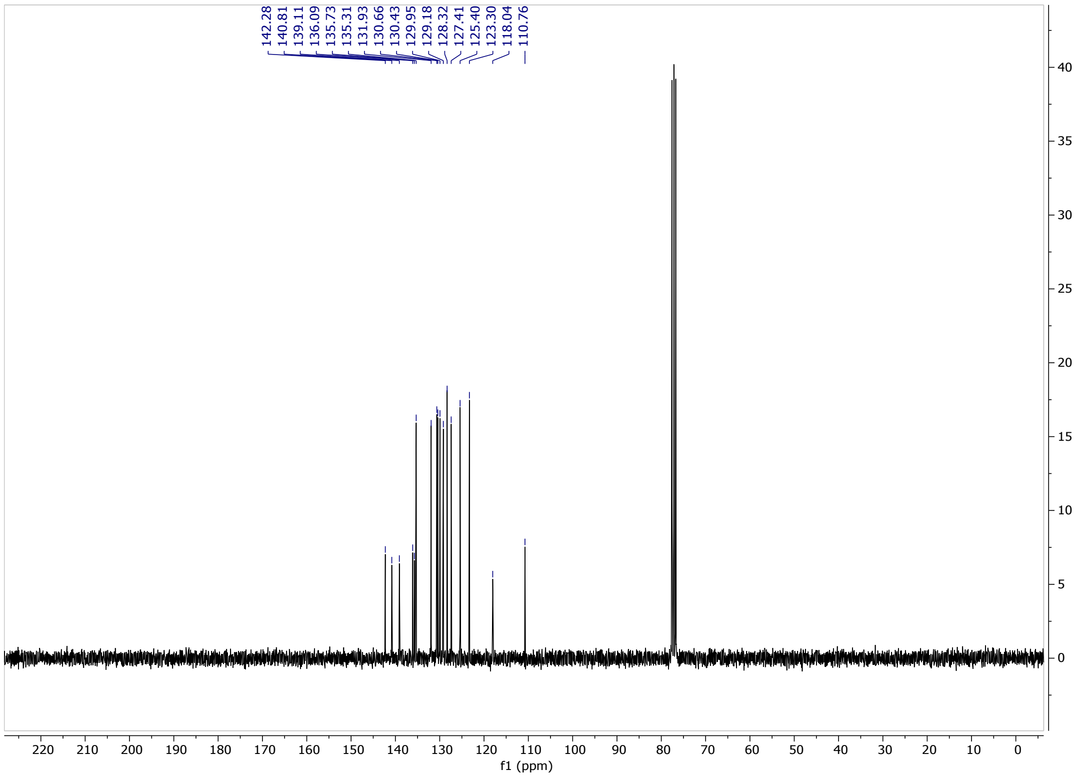

Figure S94. ^{13}C-NMR (75 MHz) spectrum of **5b** in $CDCl_3$.

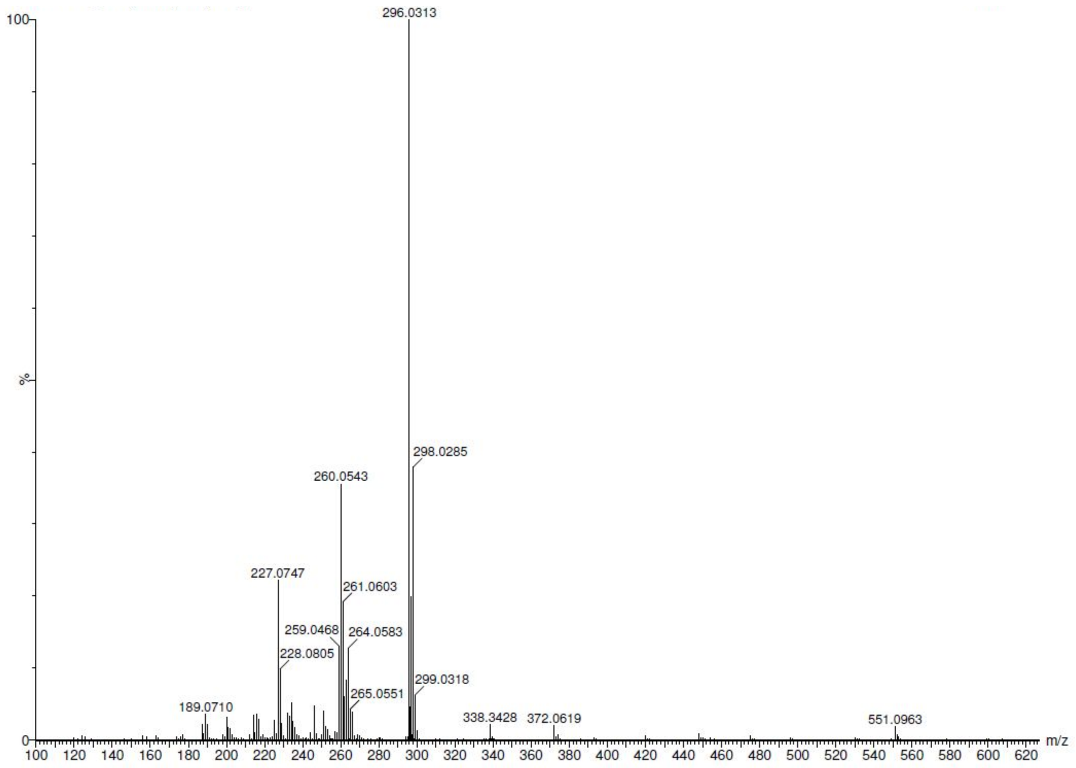

Figure S95. HRMS-APCI TOF mass spectrum of **5b**.

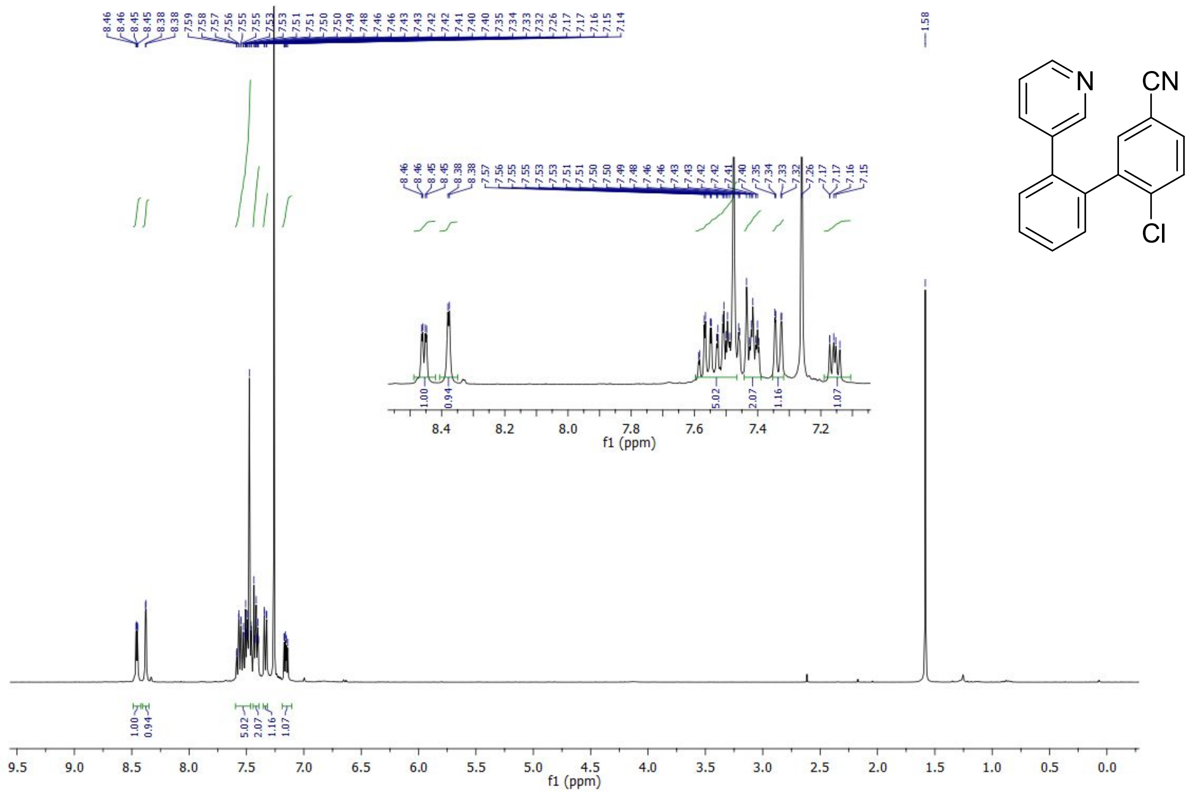

Figure S96. ^{1}H-NMR (600 MHz) spectrum of **5c** in $CDCl_3$.

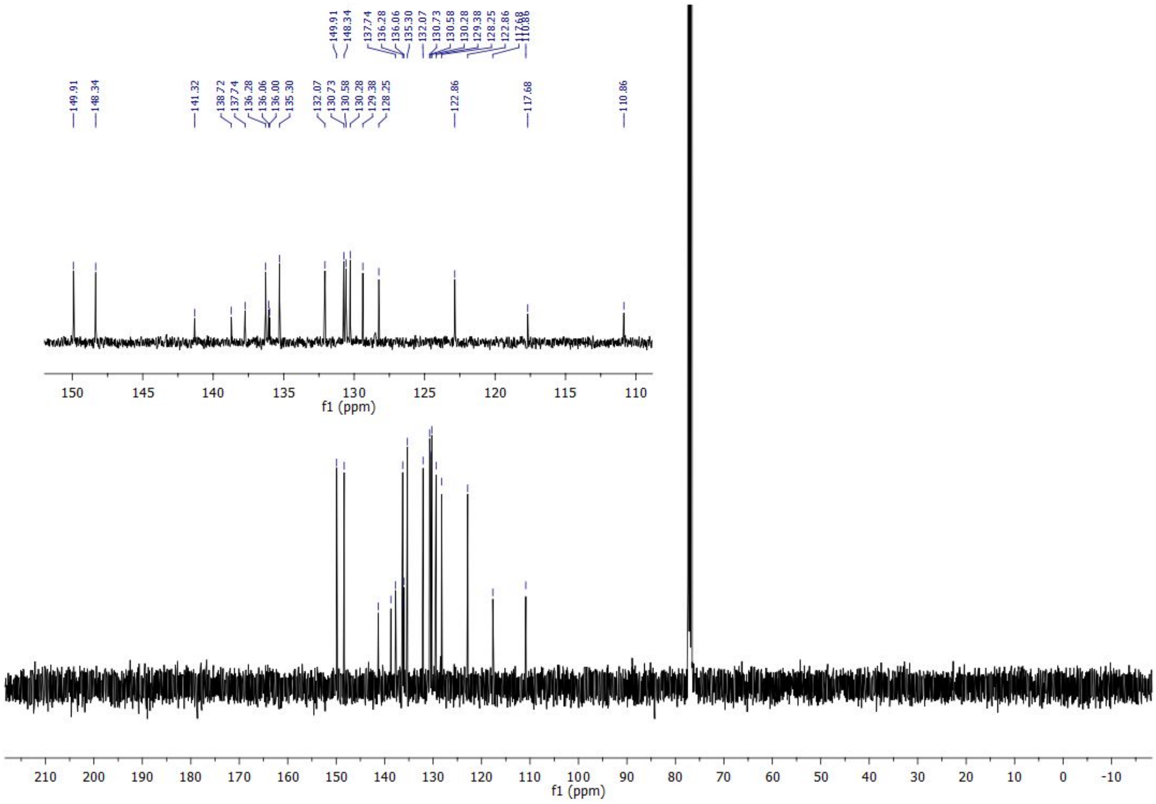

Figure S97. ^{13}C-NMR (151 MHz) spectrum of **5c** in $CDCl_3$.

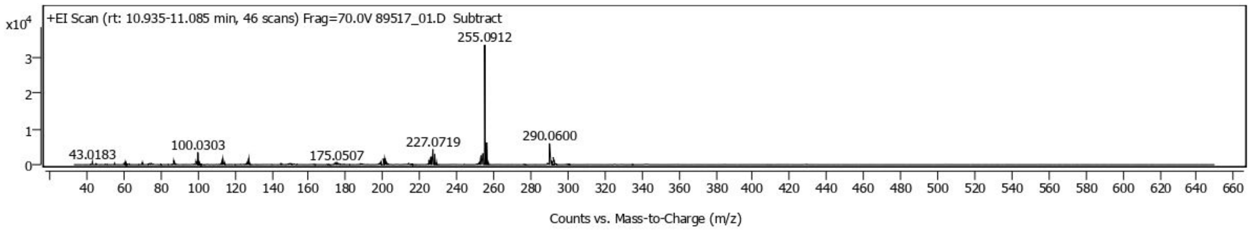

Figure S98.HRGC-MS mass spectrum of **5c**.

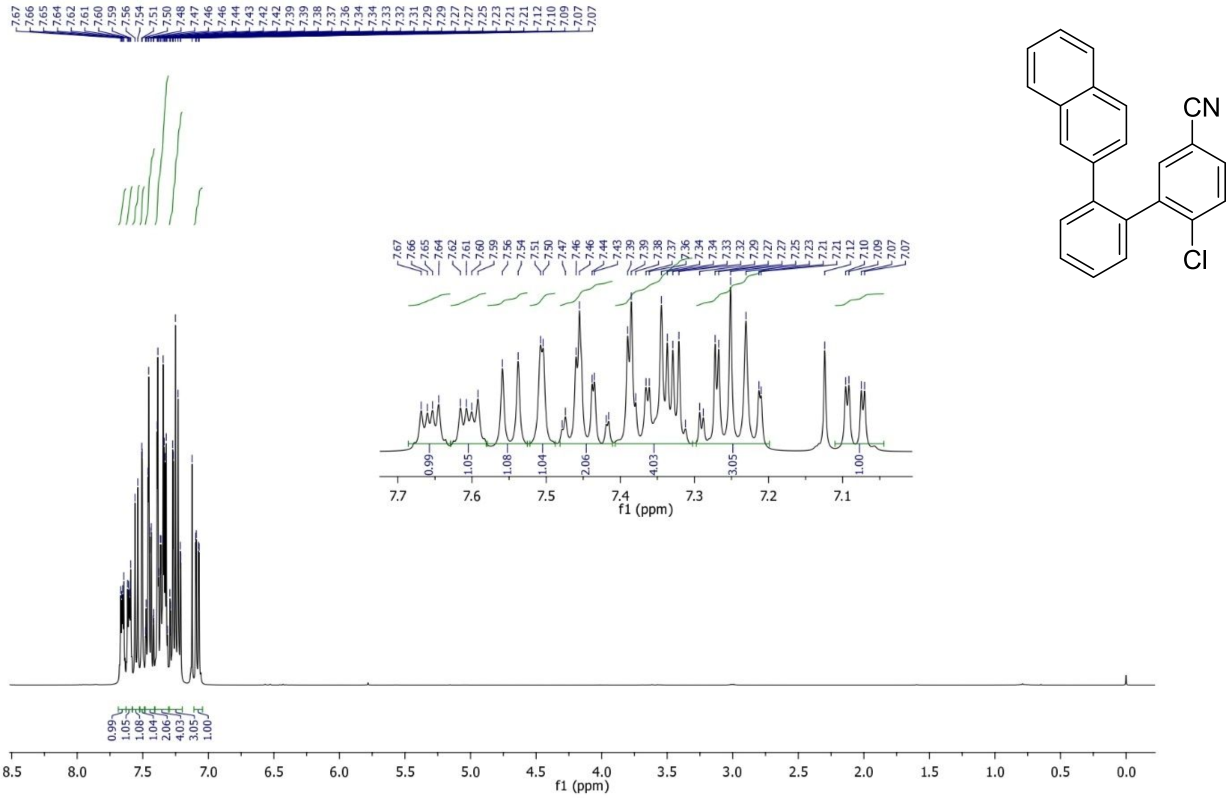

Figure S99. ^{1}H-NMR (600 MHz) spectrum of **7a** in $CDCl_3$.

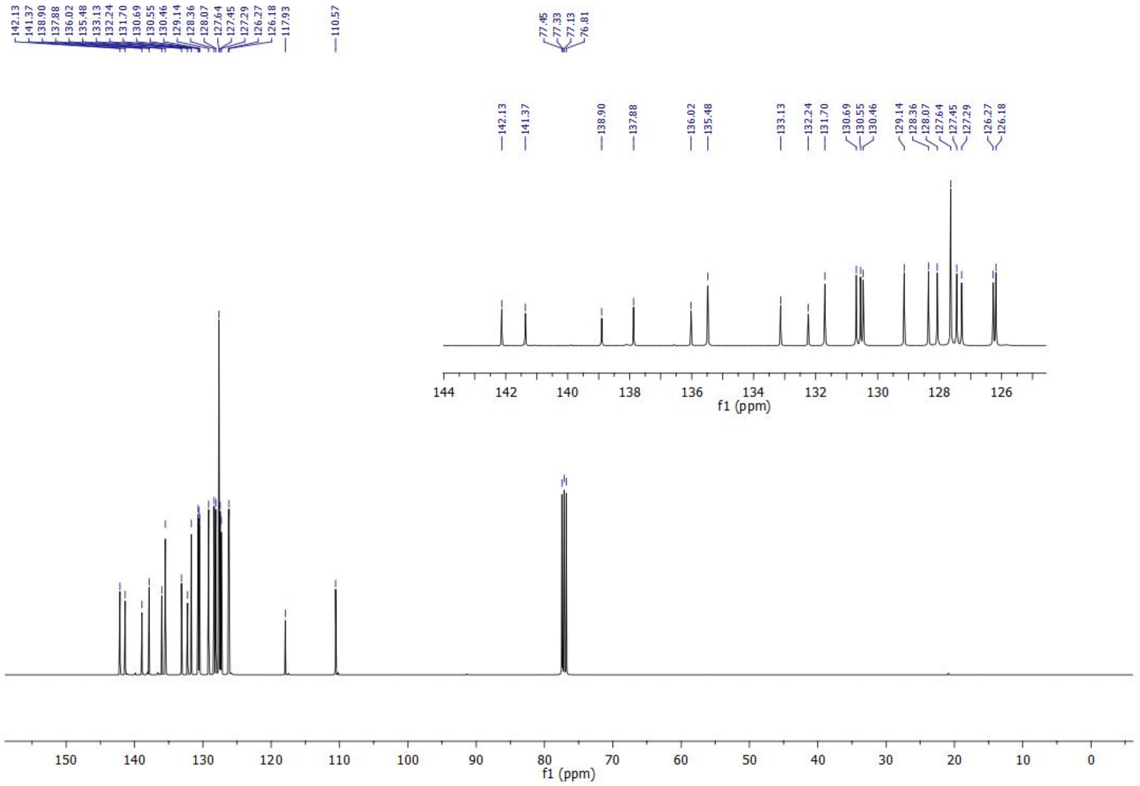

Figure S100. ^{13}C-NMR (151 MHz) spectrum of **7a** in $CDCl_3$.

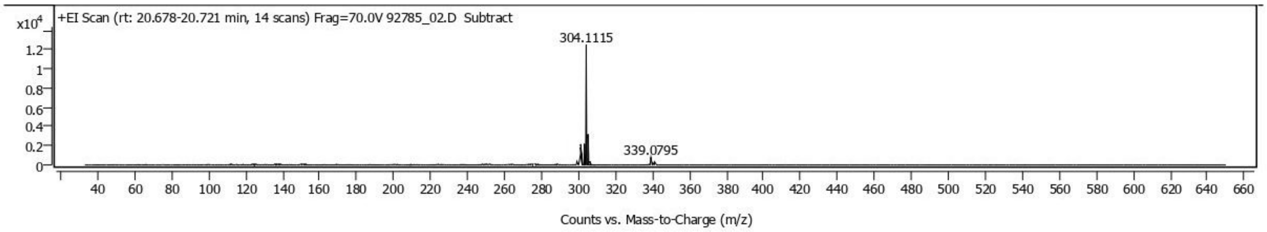

Figure S101. HRGC-MS mass spectrum of **7a.**

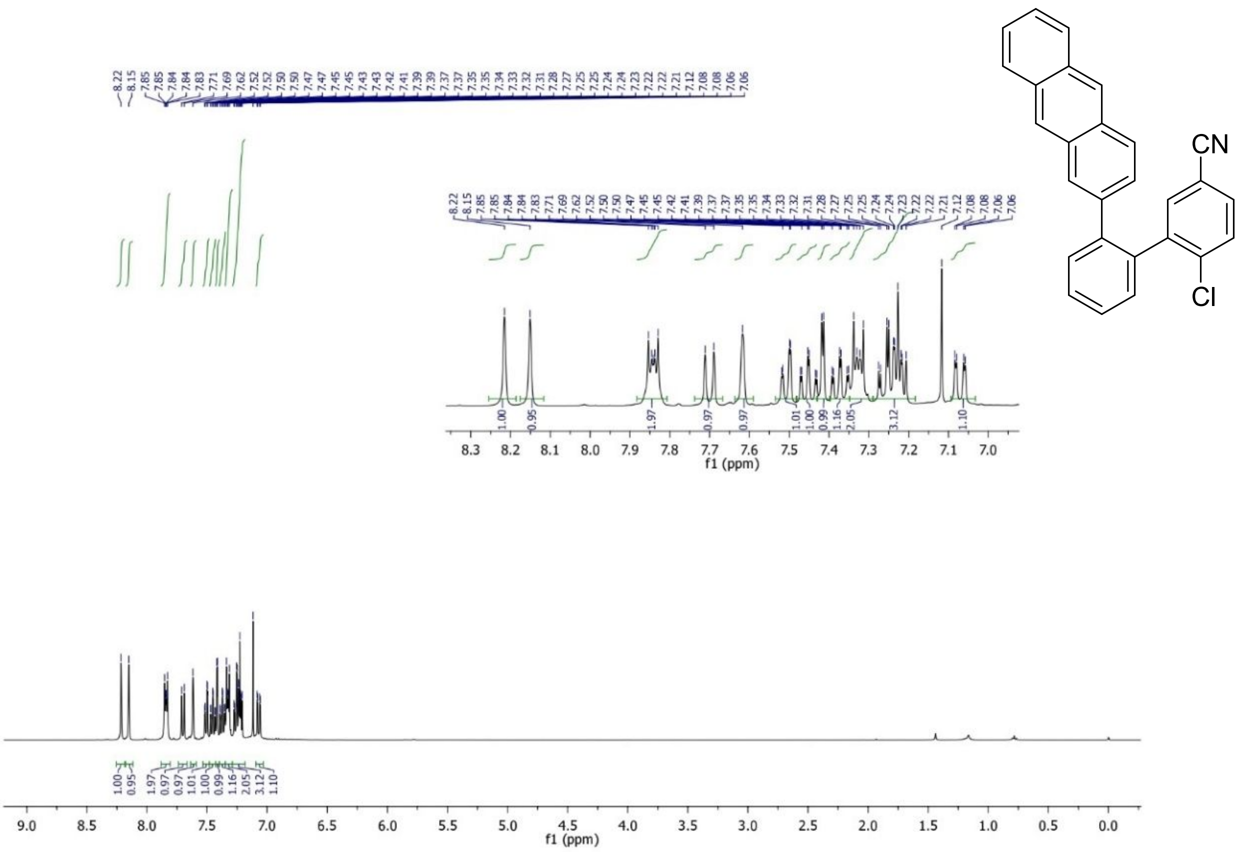

Figure S102. ^{1}H-NMR (600 MHz) spectrum of **7b** in $CDCl_3$.

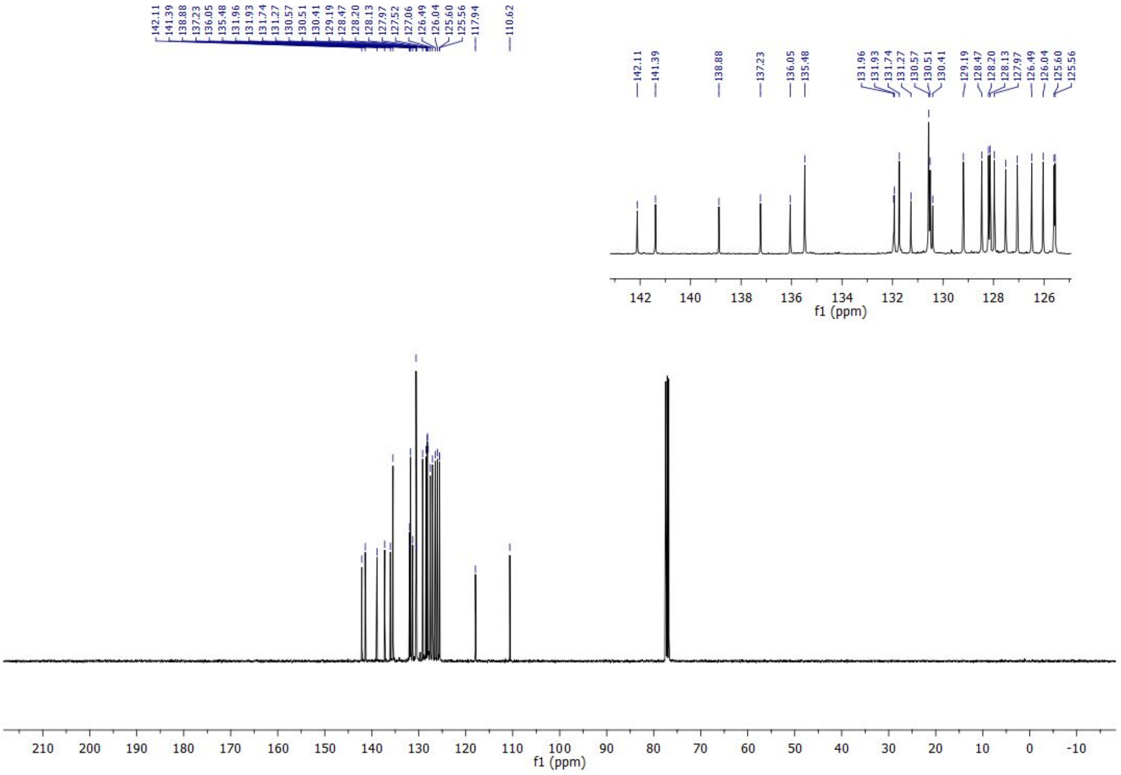

Figure S103. ^{13}C-NMR (151 MHz) spectrum of **7b** in $CDCl_3$.

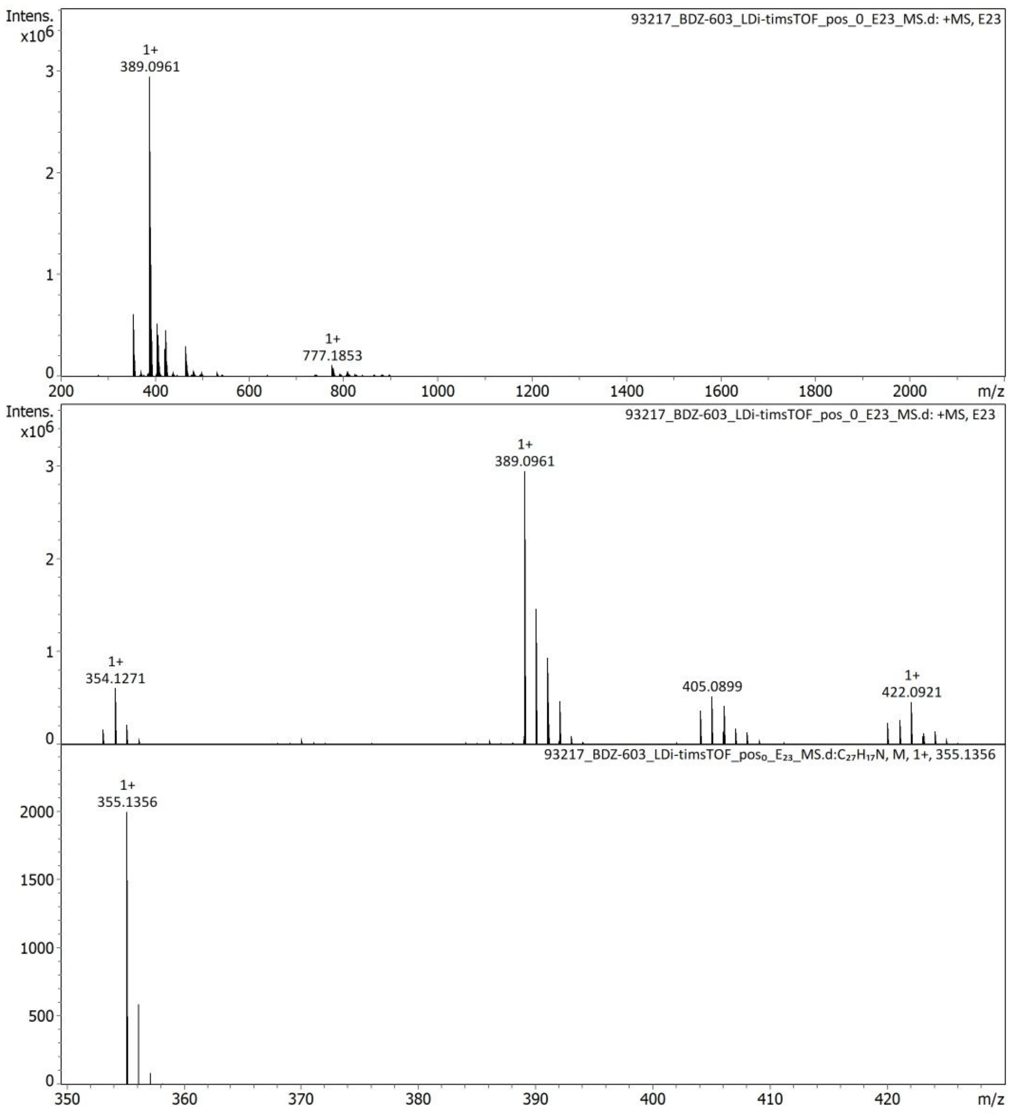

Figure S104. HRMS-LD-TOF mass spectrum of **7b.**

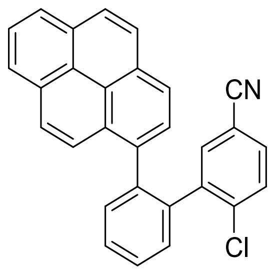

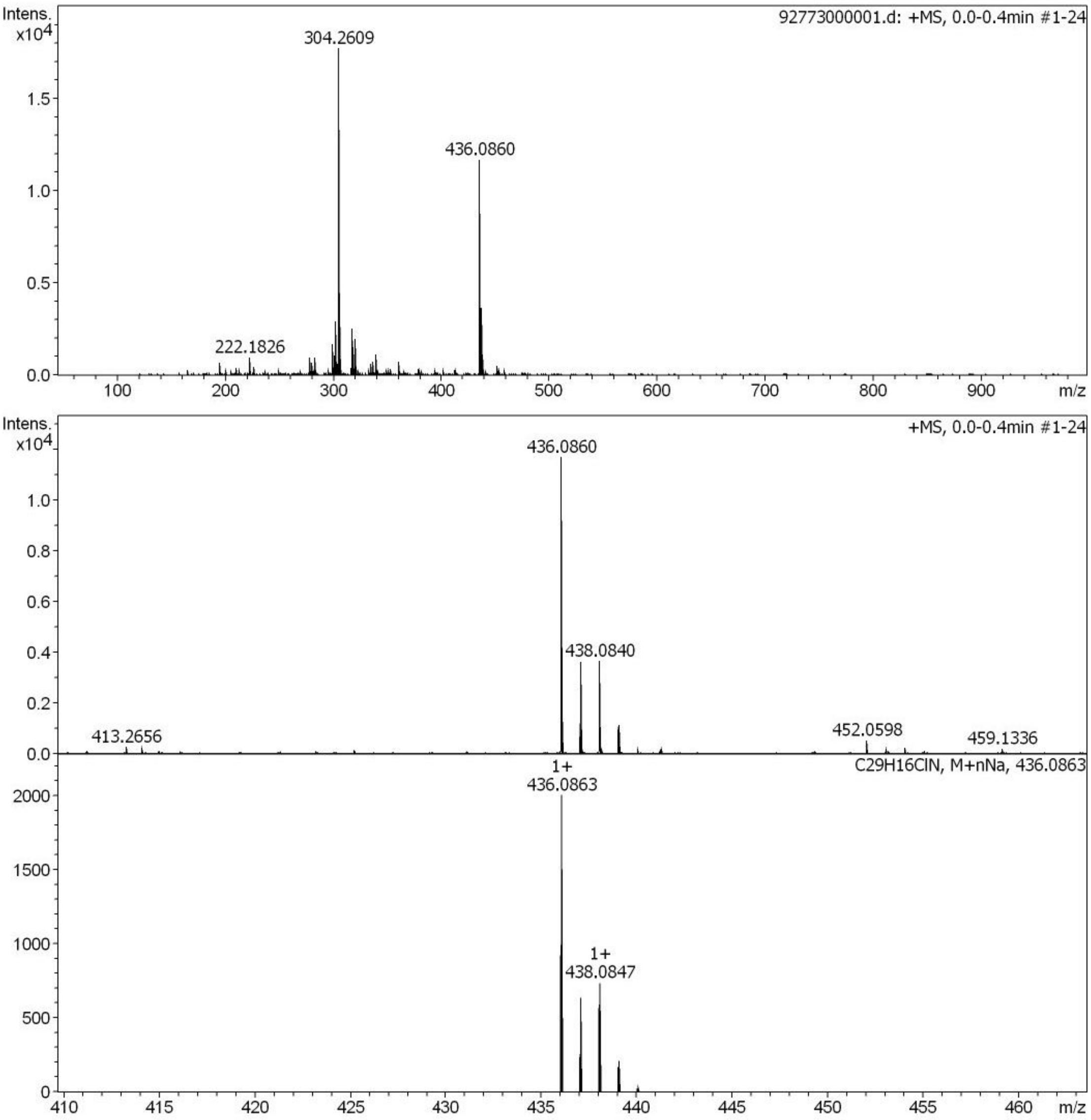

Figure S105. HRMS-Maldi TOF mass spectrum of **7c.**

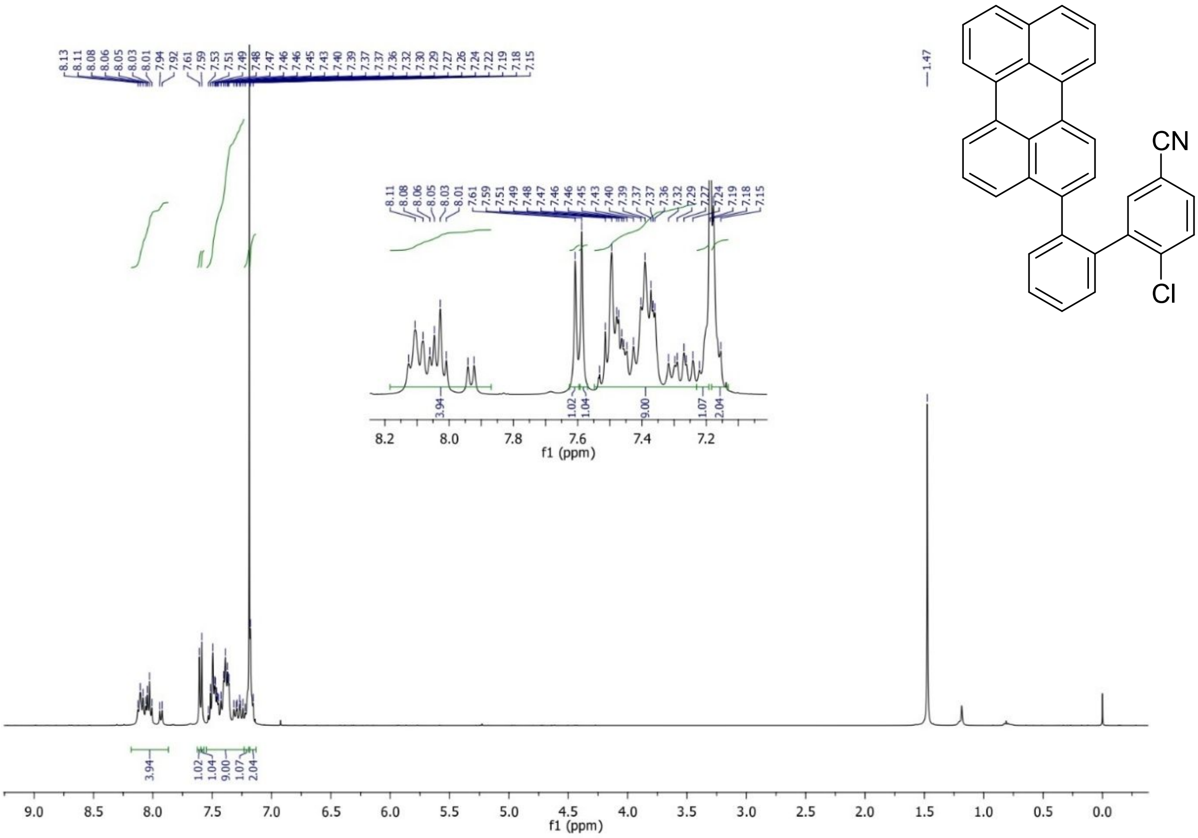

Figure S106. ^{1}H-NMR (600 MHz) spectrum of **7d** in $CDCl_3$.

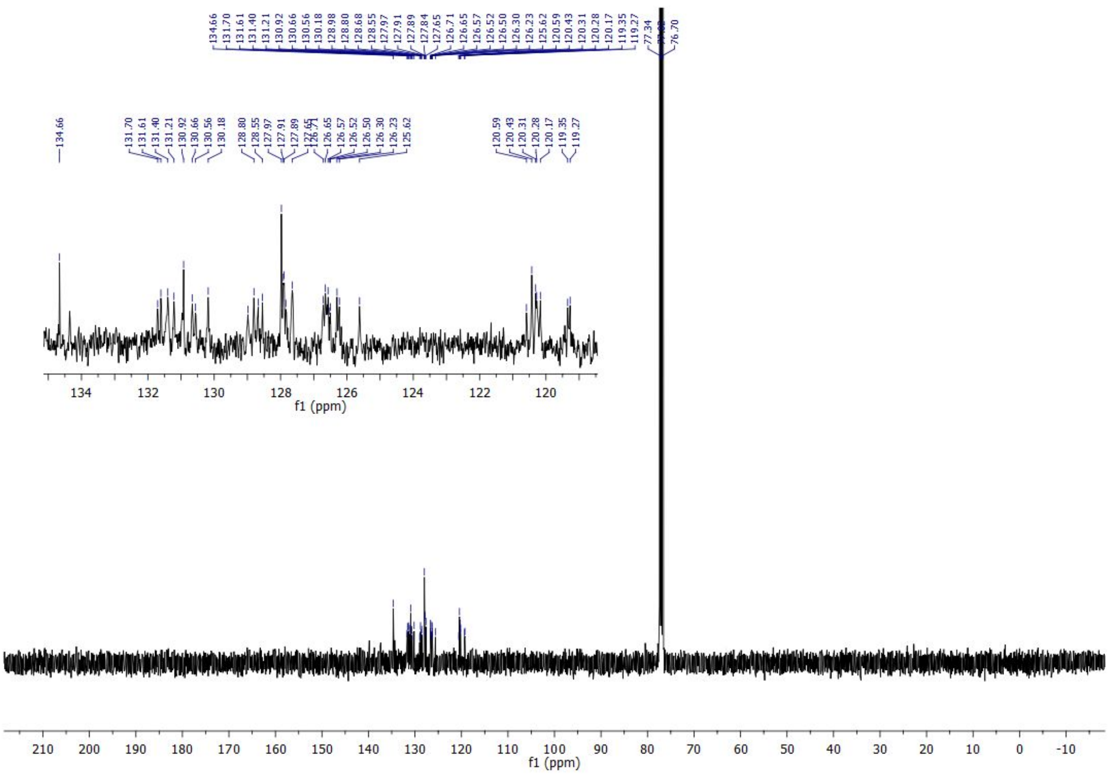

Figure S107. ^{13}C-NMR (151 MHz) spectrum of **7d** in $CDCl_3$.

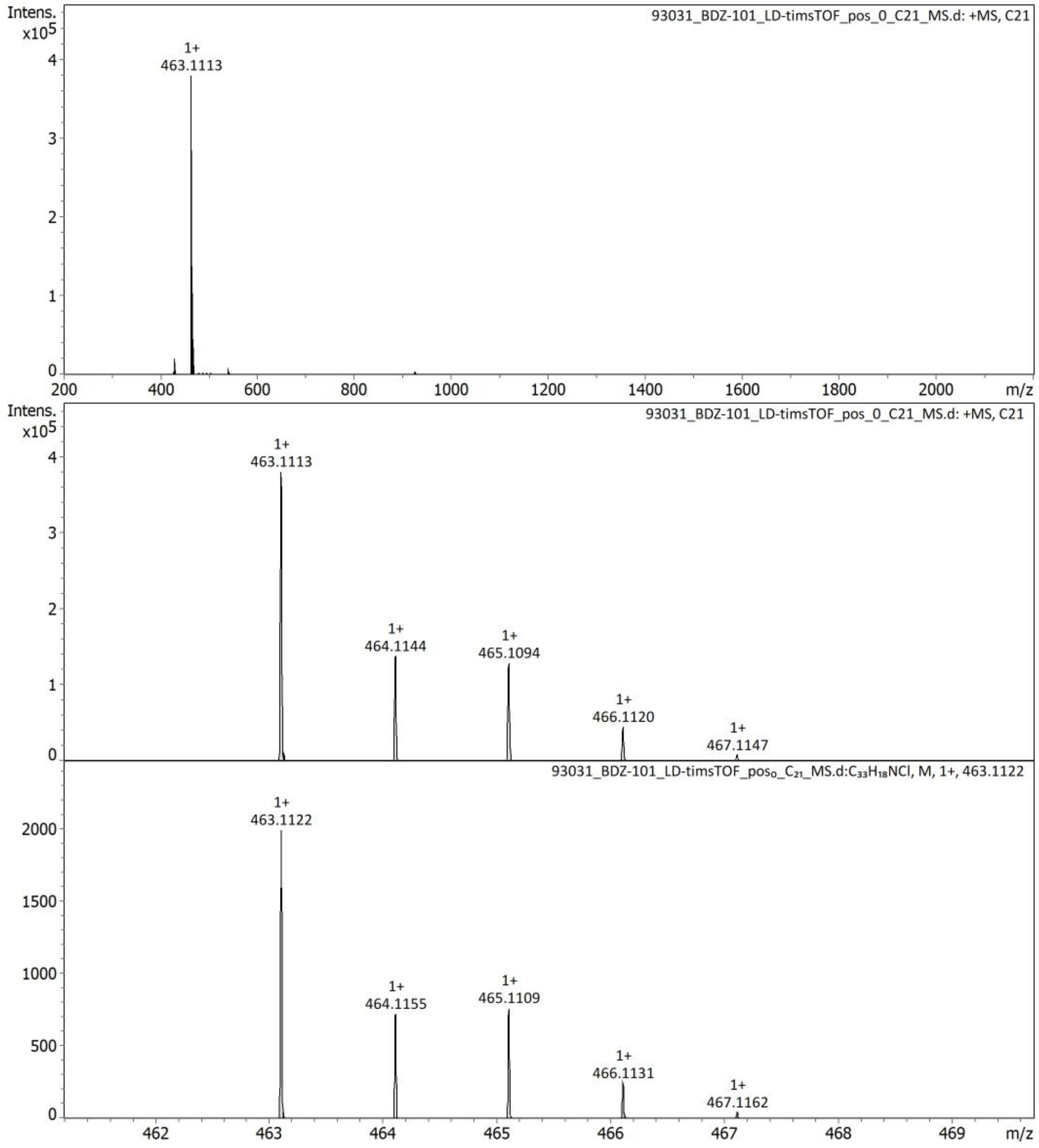

Figure S108. HRMS-MALDI-TOF mass spectrum of **7d.**

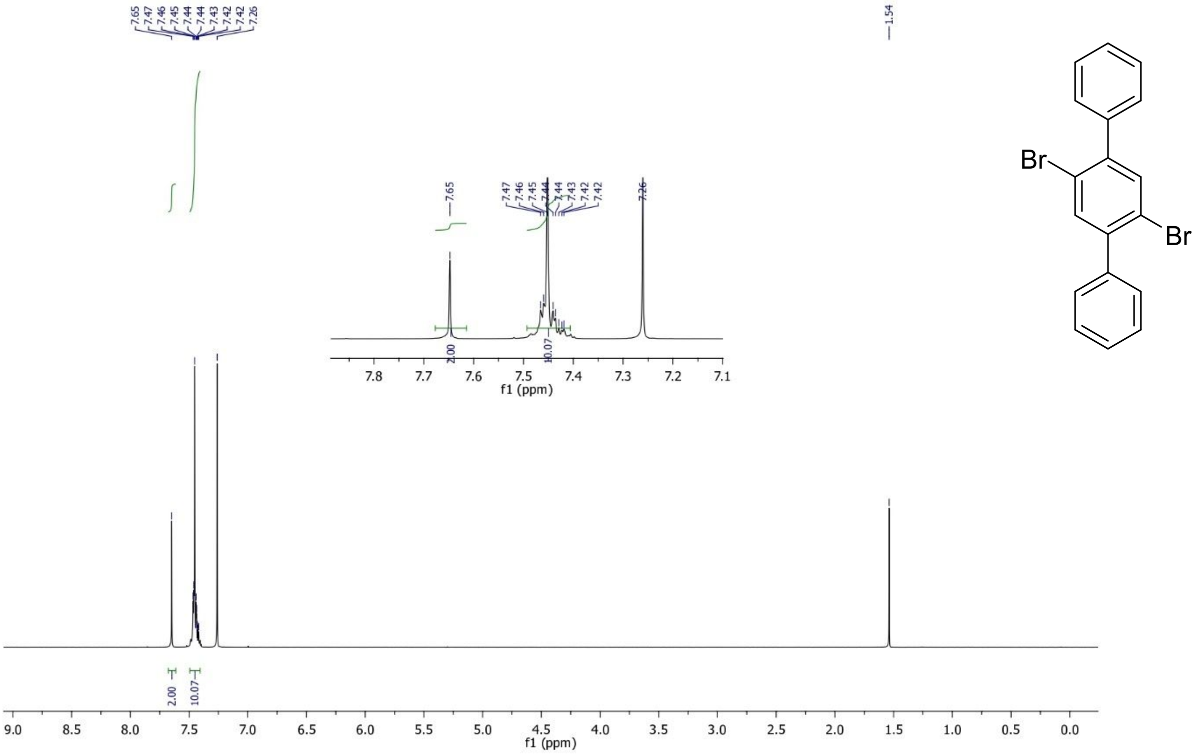

Figure S109. ^{1}H-NMR (600 MHz) spectrum of **2',5'-dibromo-1,1':4',1''-terphenyl** in $CDCl_3$.

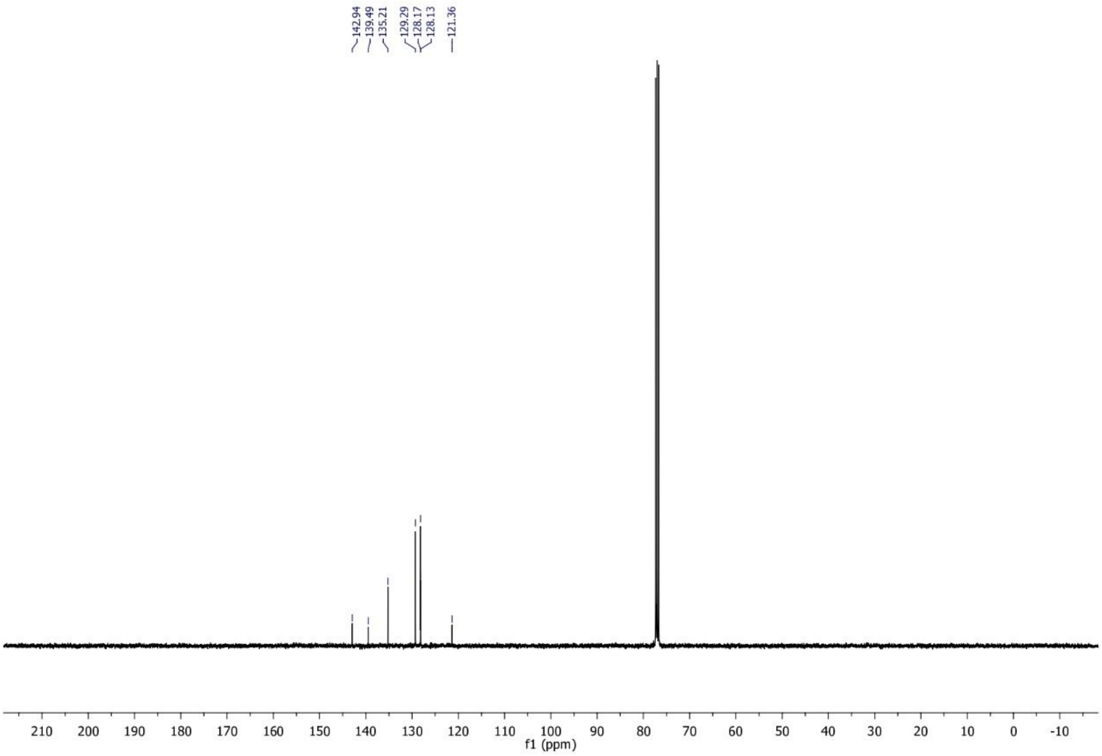

Figure S110. ^{13}C-NMR (151 MHz) spectrum of **2',5'-dibromo-1,1':4',1''-terphenyl** in $CDCl_3$.

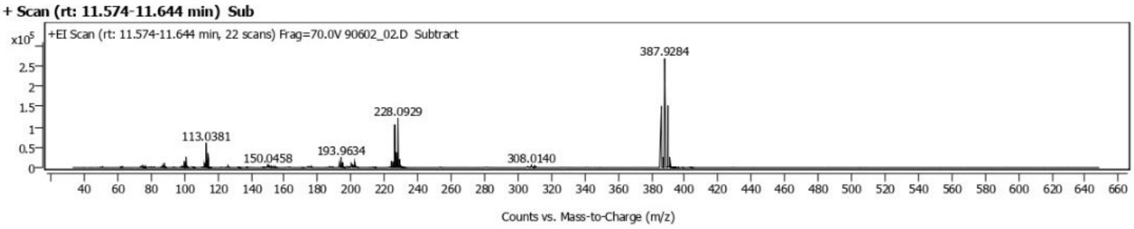

Figure S111. HRGC-MS mass spectrum of **2',5'-dibromo-1,1':4',1''-terphenyl.**

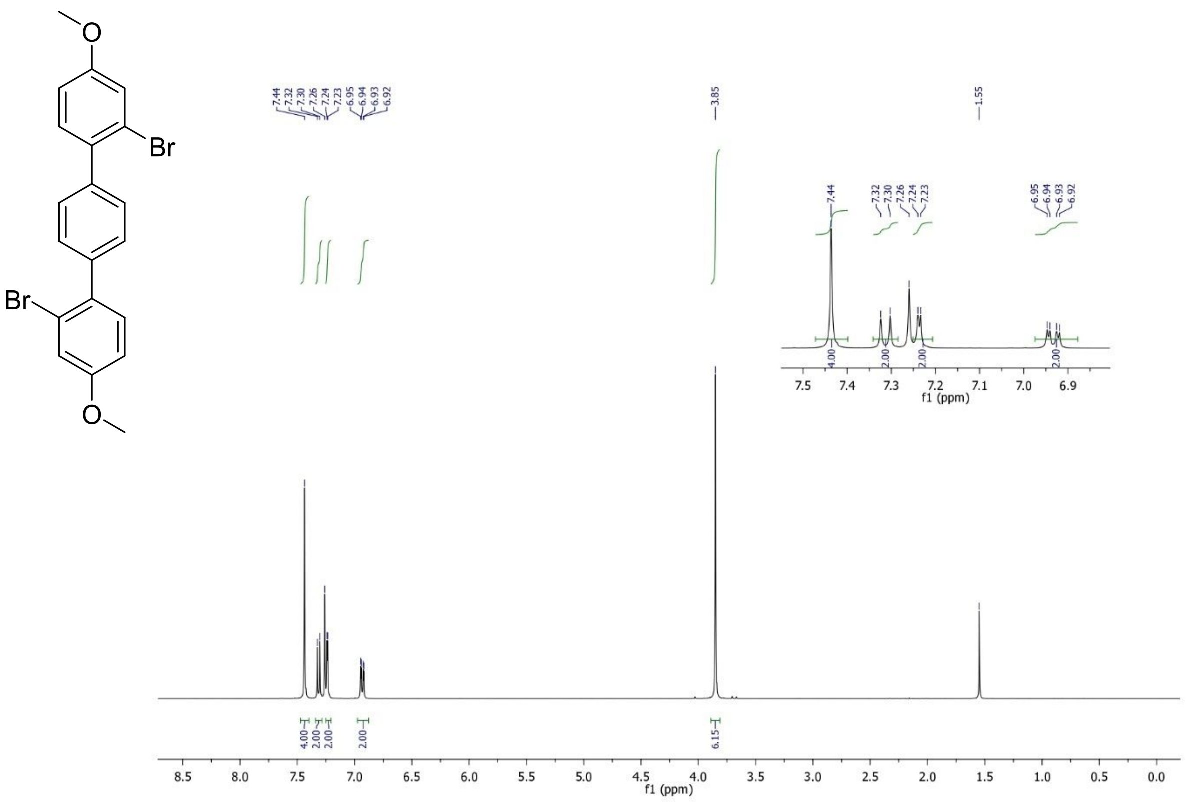

Figure S112. ^{1}H-NMR (600 MHz) spectrum of **2,2''-dibromo-4,4''-dimethoxy-1,1':4',1''-terphenyl** in $CDCl_3$.

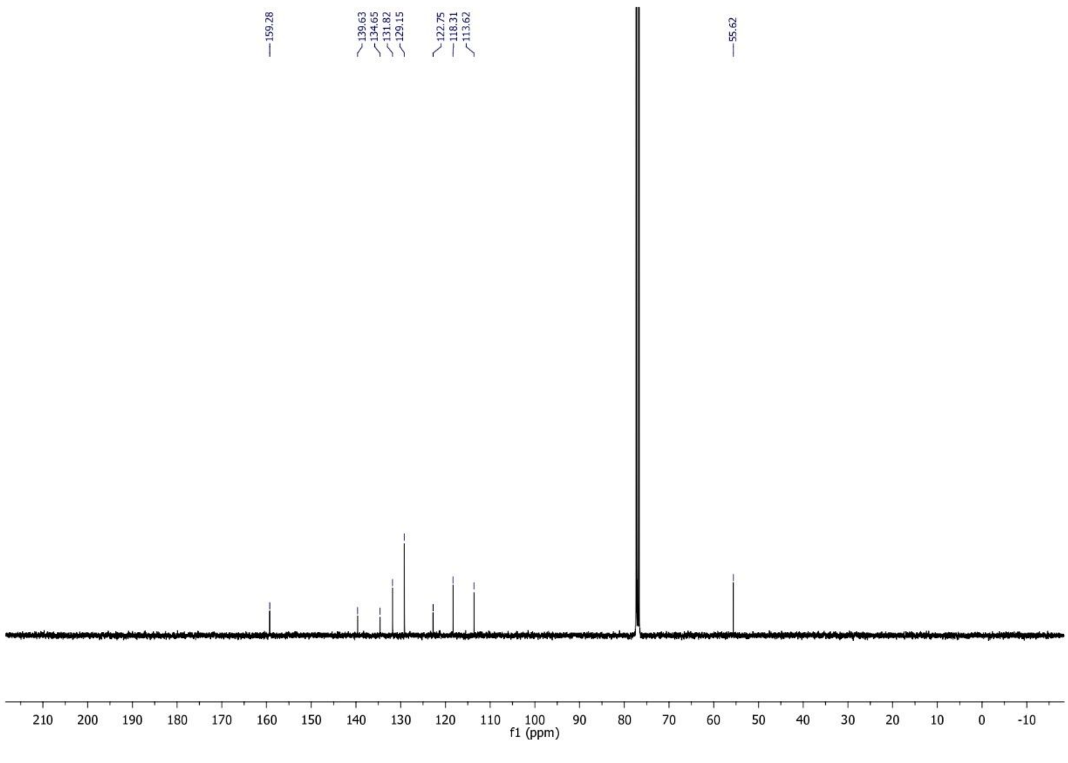

Figure S113. ^{13}C-NMR (151 MHz) spectrum of **2,2''-dibromo-4,4''-dimethoxy-1,1':4',1''-terphenyl** in $CDCl_3$.

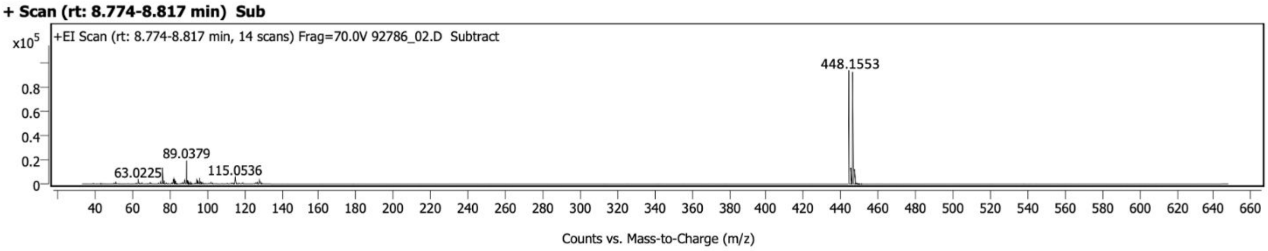

Figure S114. HRMS-ESI mass spectrum of **2,2''-dibromo-4,4''-dimethoxy-1,1':4',1''-terphenyl.**

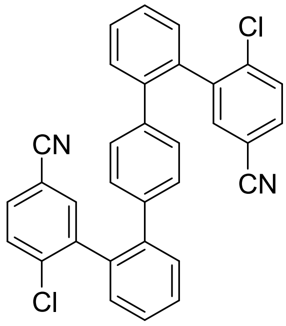

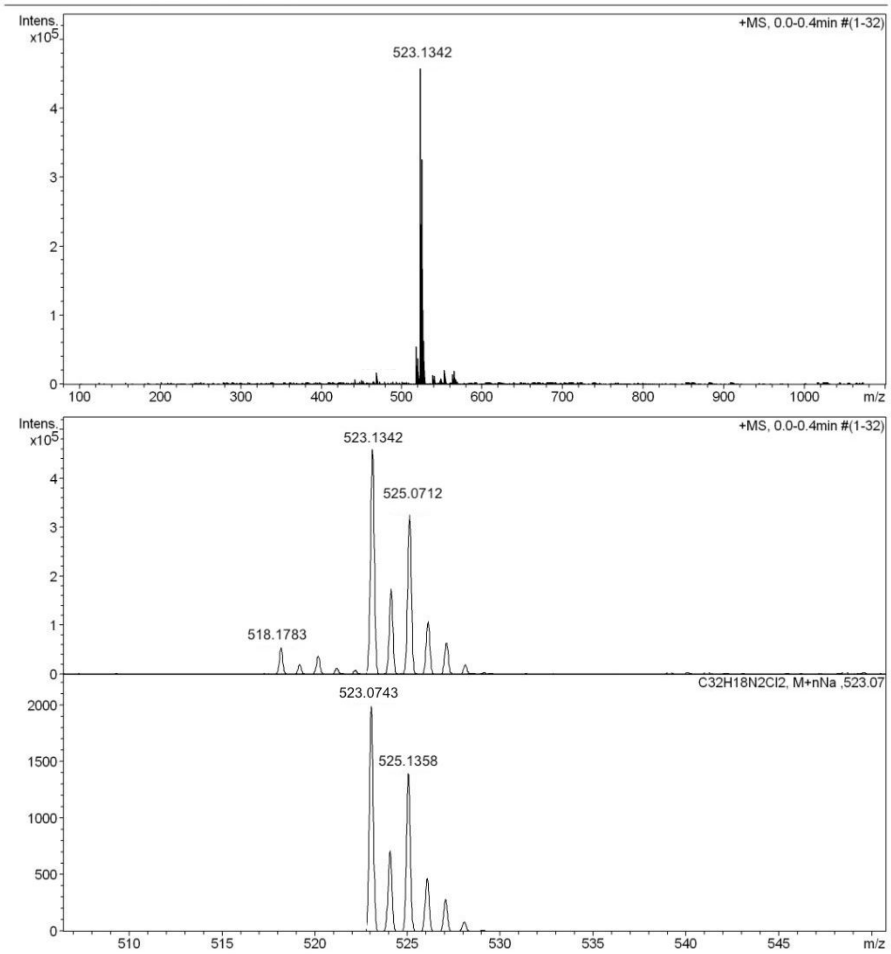

Figure S115. HRMS-ESI mass spectrum of **9a.**

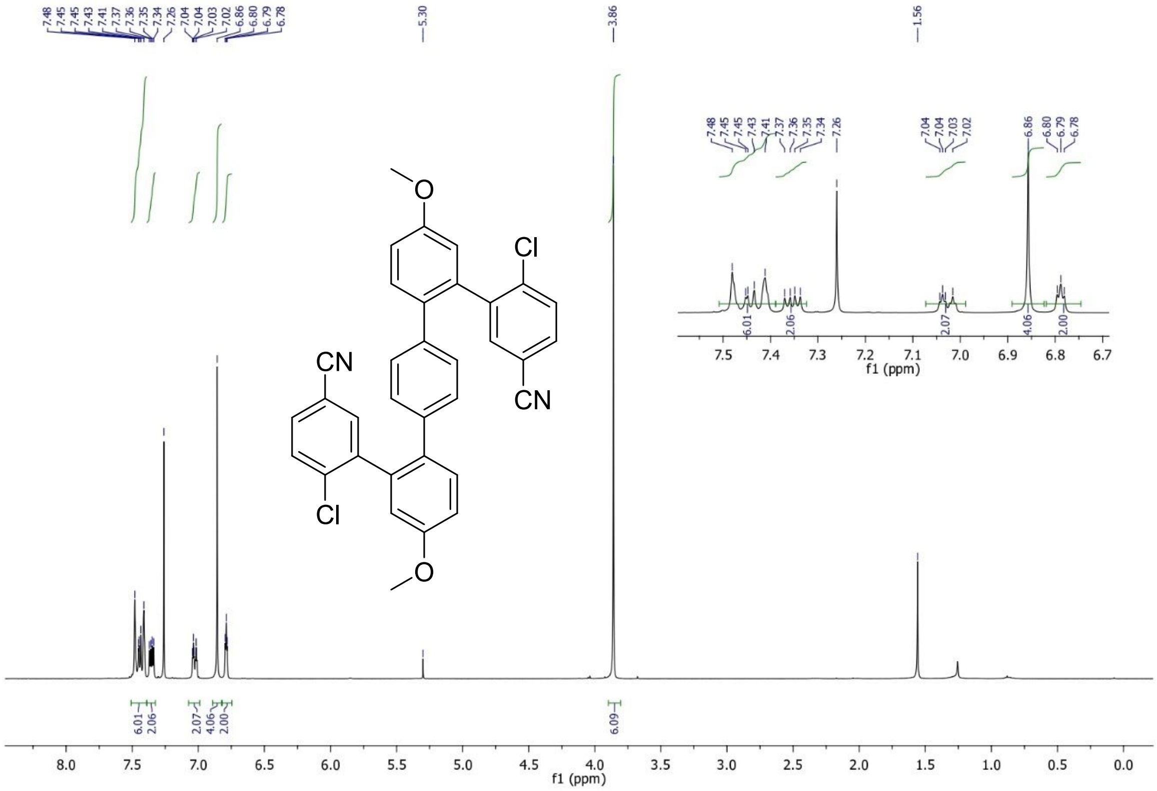

Figure S116. ^{1}H-NMR (600 MHz) spectrum of **9b** in $CDCl_3$.

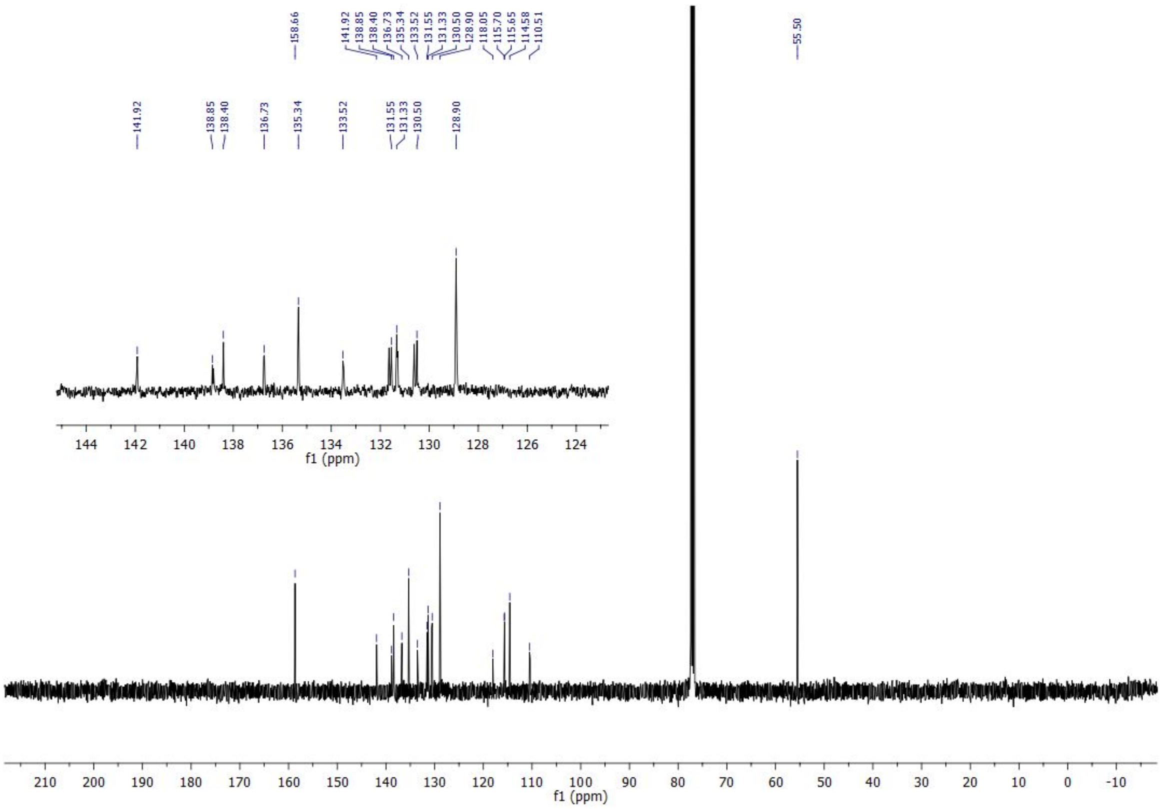

Figure S117. ^{13}C-NMR (151 MHz) spectrum of **9b** in $CDCl_3$.

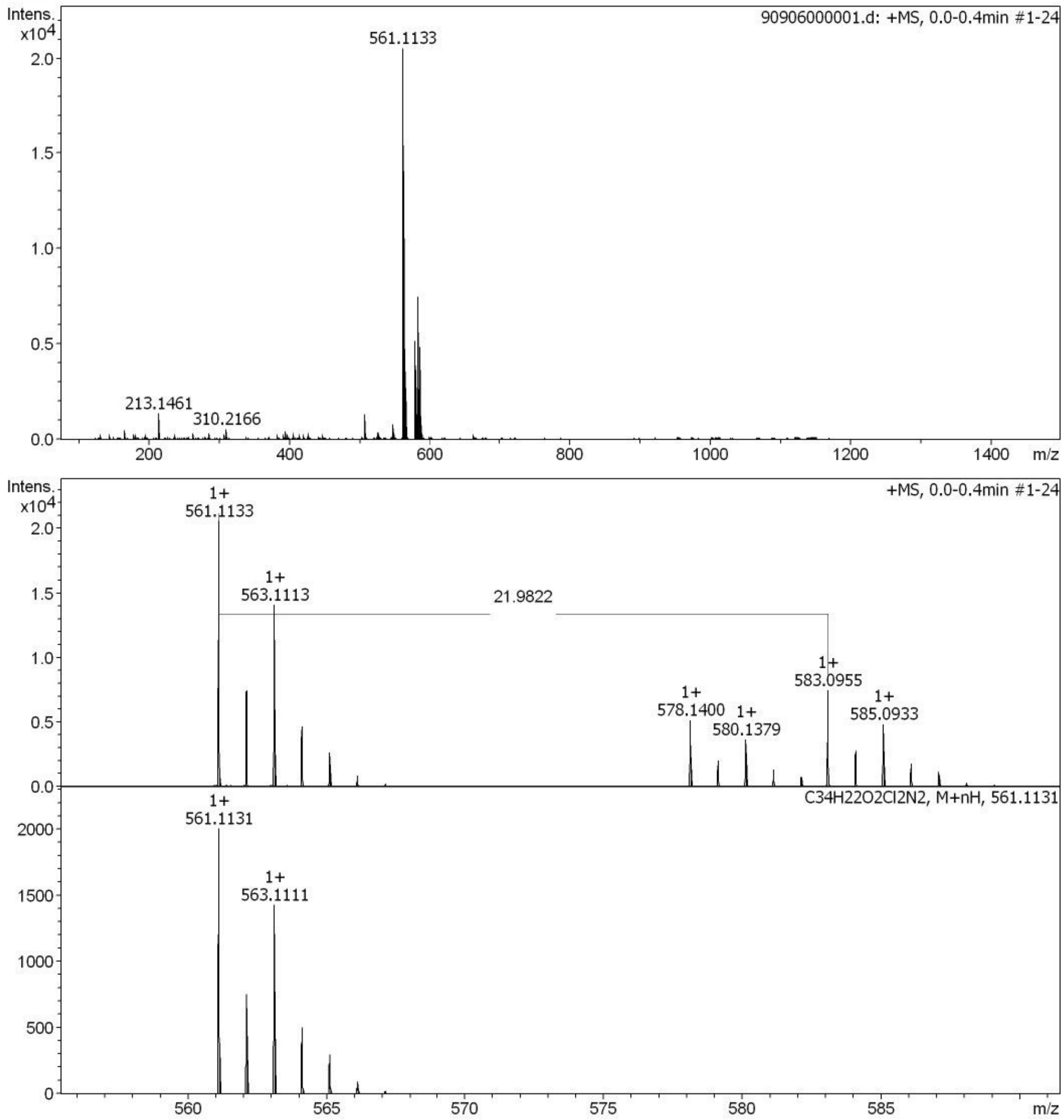

Figure S118. HRMS-ESI mass spectrum of **9b.**

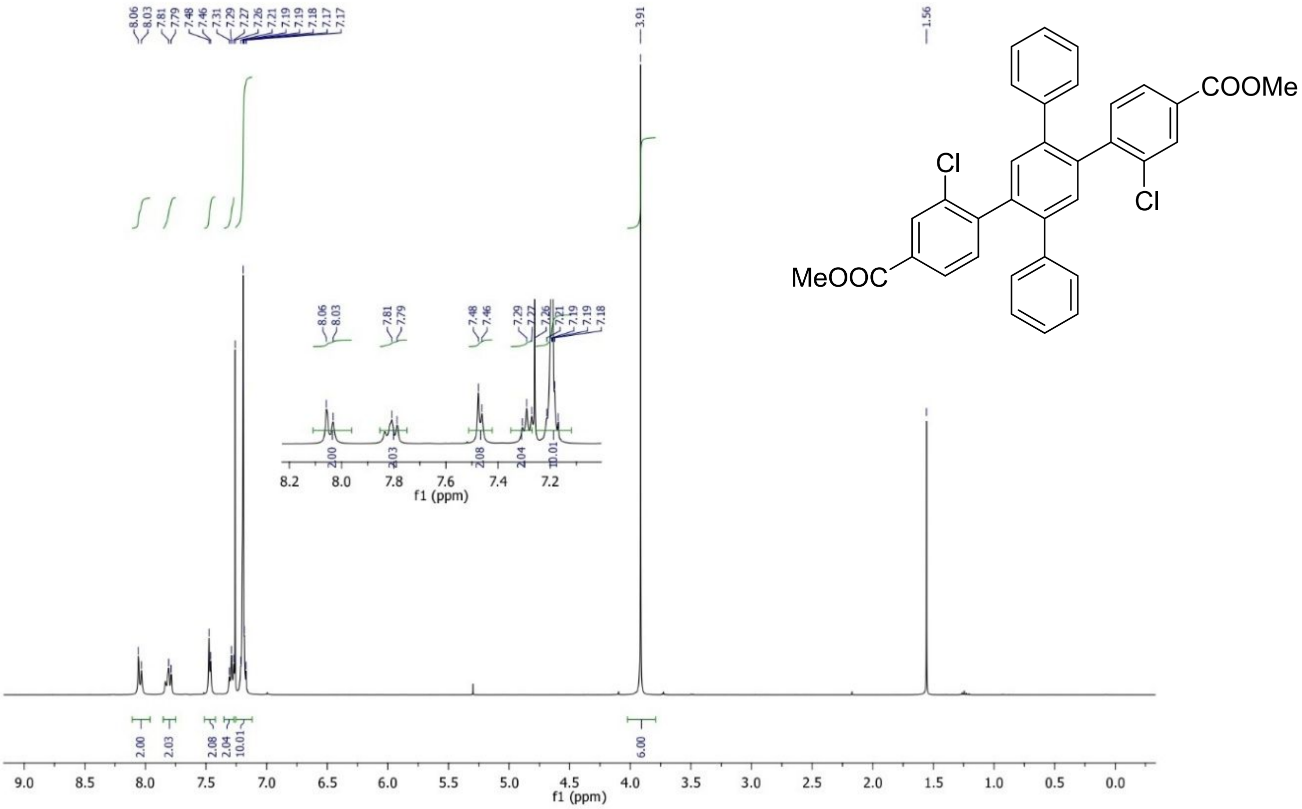

Figure S119. ^{1}H-NMR (600 MHz) spectrum of **10a** in $CDCl_3$.

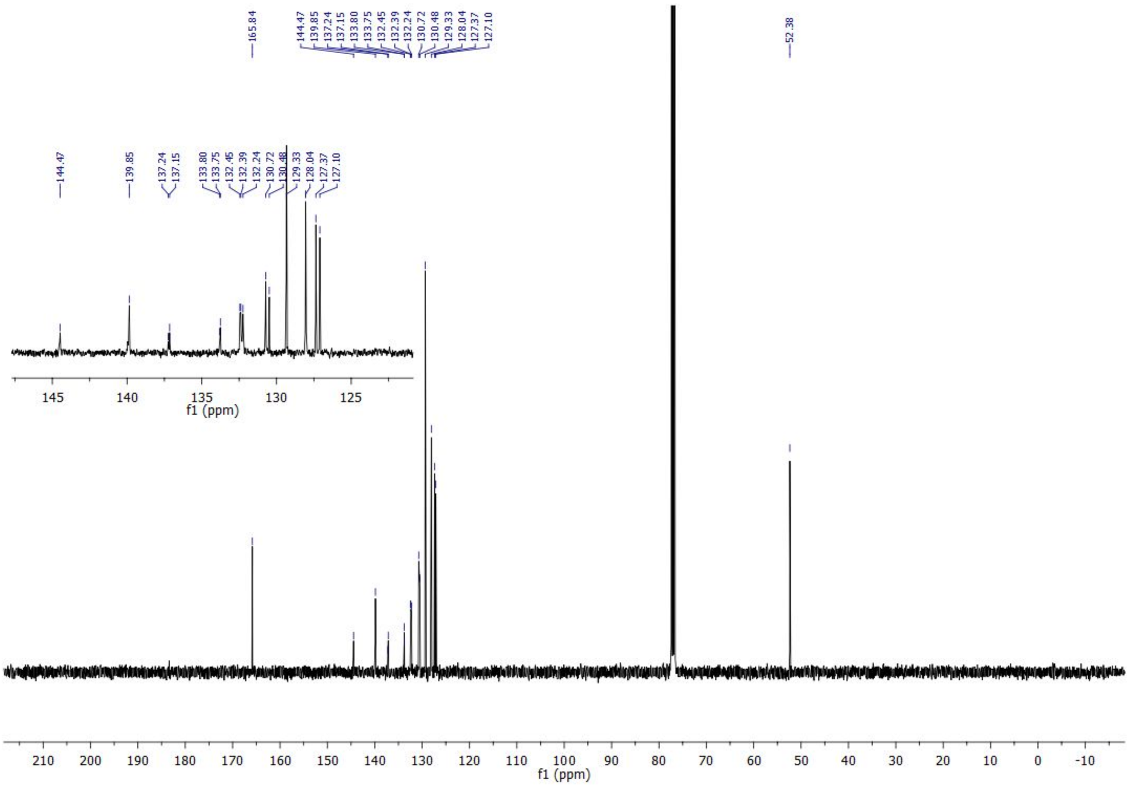

Figure S120. ^{13}C-NMR (151 MHz) spectrum of **10a** in $CDCl_3$.

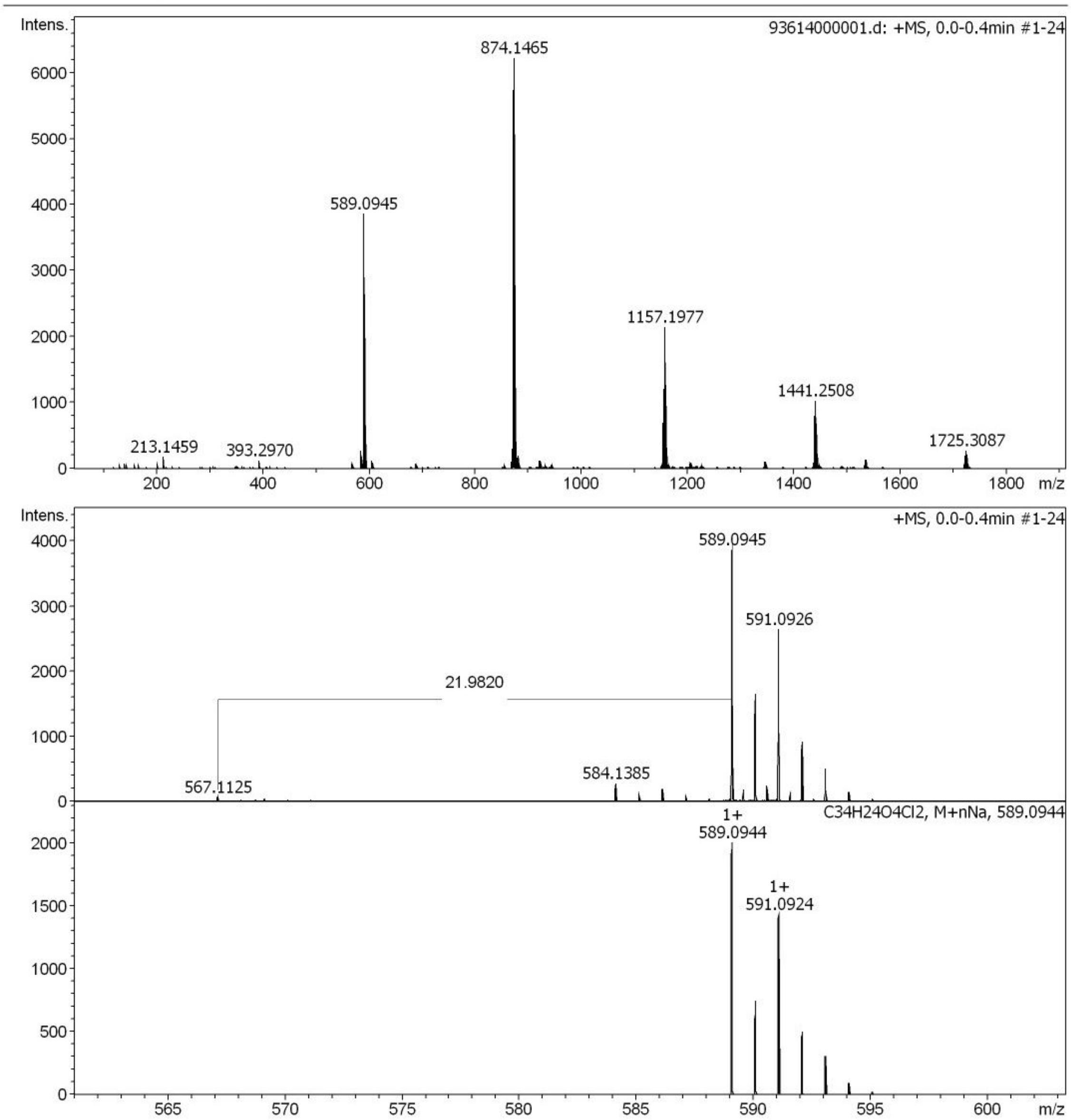

Figure S121. HRMS-ESI mass spectrum of **10a.**

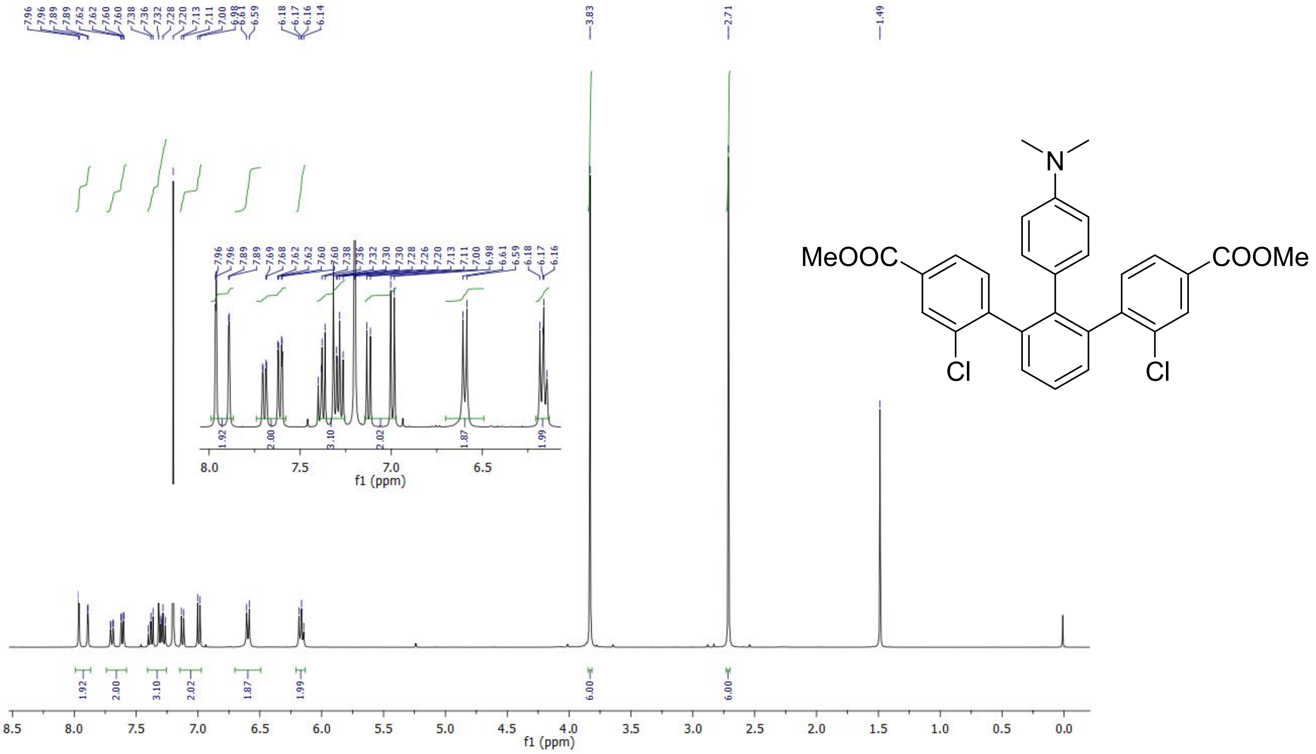

Figure S122. ^{1}H-NMR (600 MHz) spectrum of **12a** in $CDCl_3$.

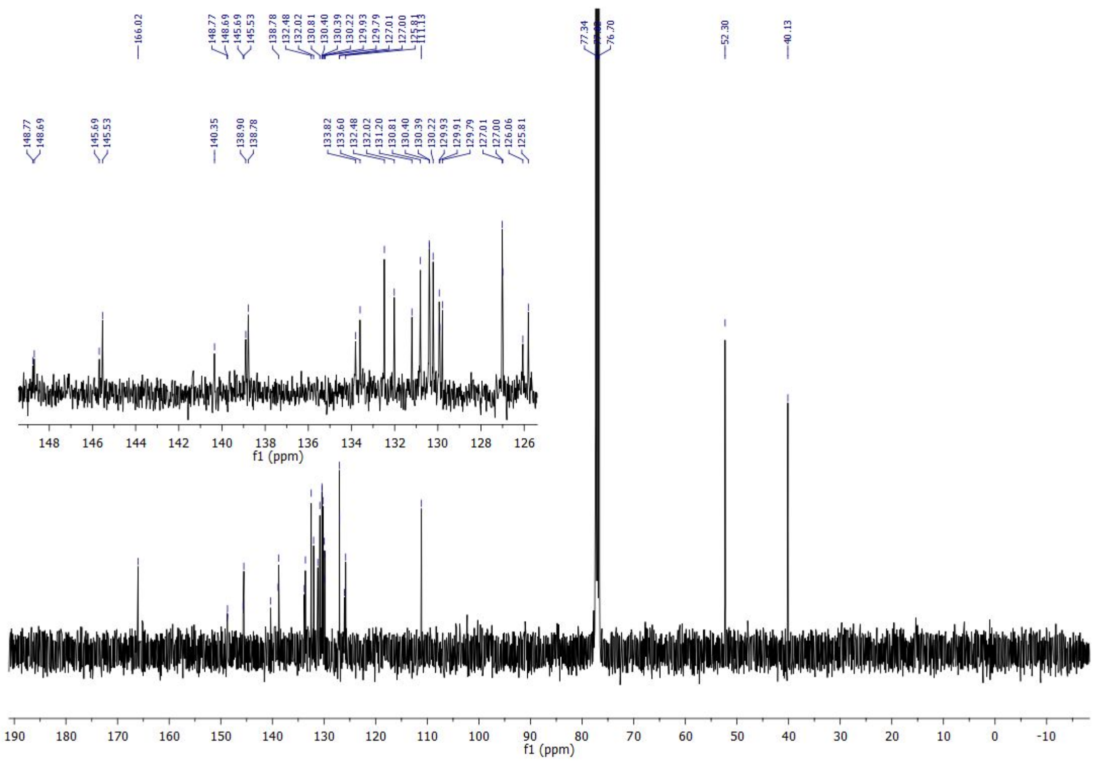

Figure S123. ^{13}C-NMR (151 MHz) spectrum of **12a** in $CDCl_3$.

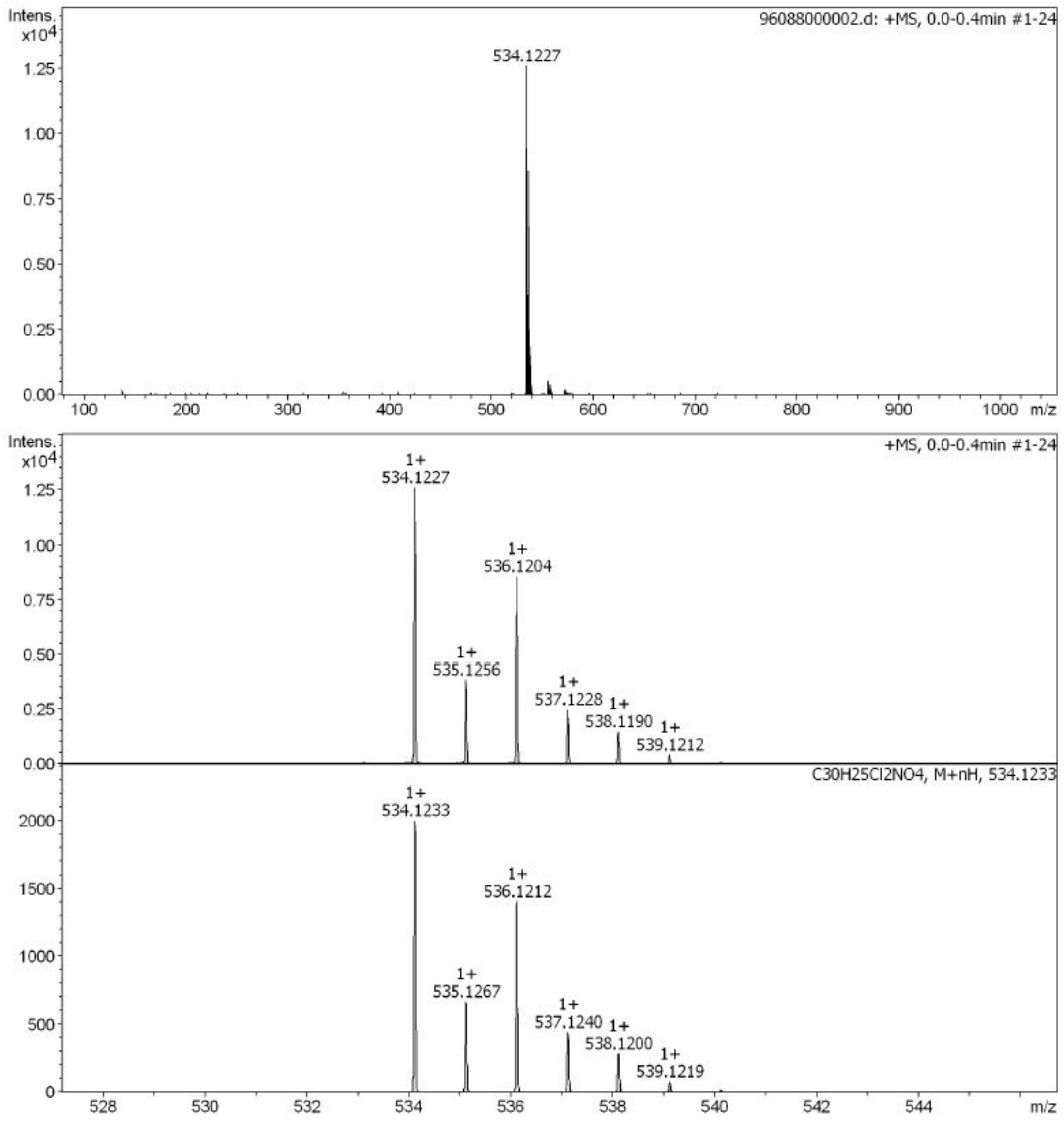

Figure S124. HRMS-ESI mass spectrum of **12a.**

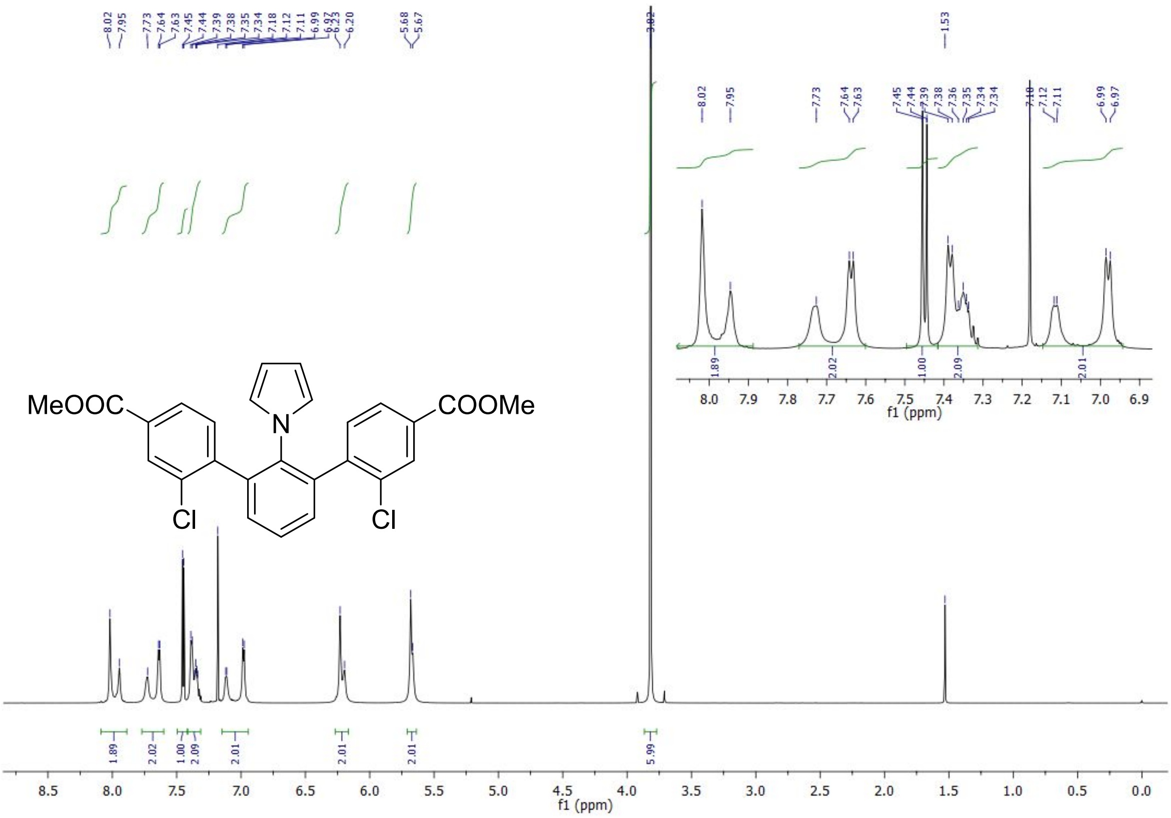

Figure S125. ^{1}H-NMR (600 MHz) spectrum of **12b** in $CDCl_3$.

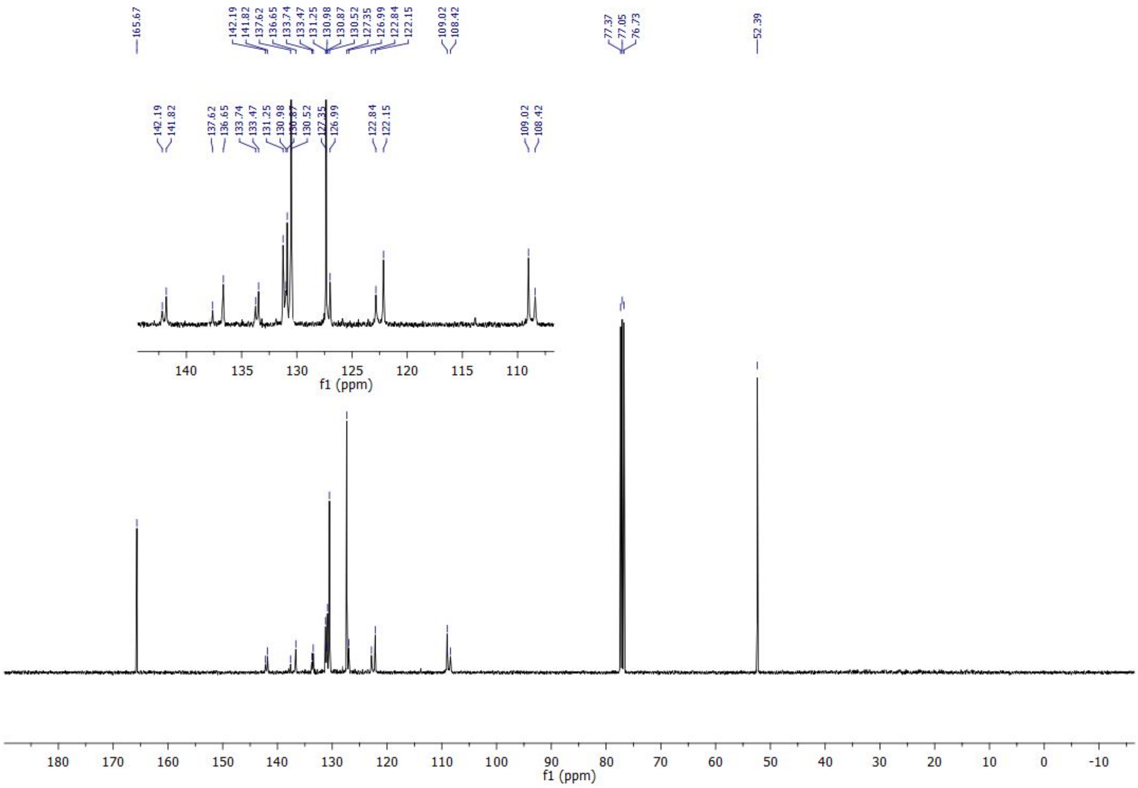

Figure S126. ^{13}C-NMR (151 MHz) spectrum of **12b** in $CDCl_3$.

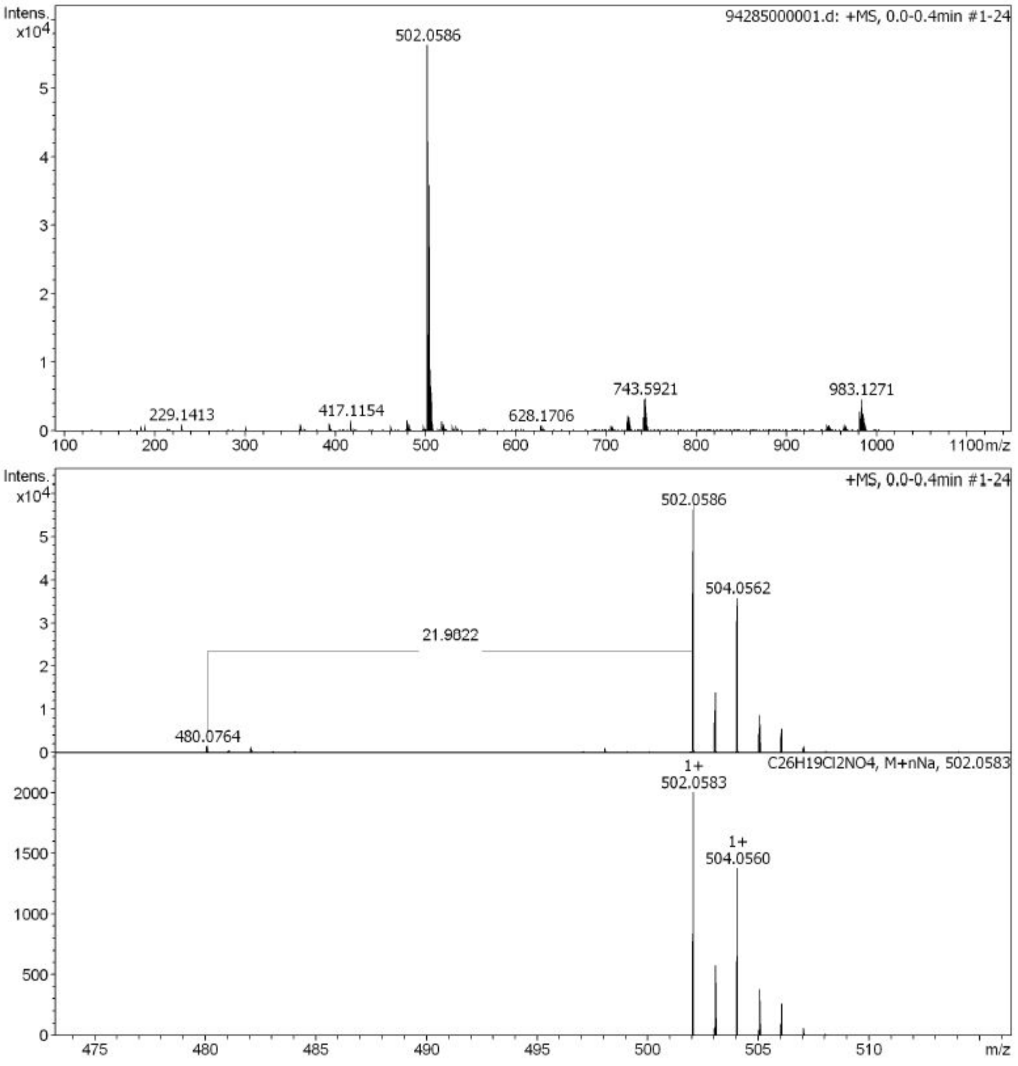

Figure S127. HRMS-ESI mass spectrum of **12b.**

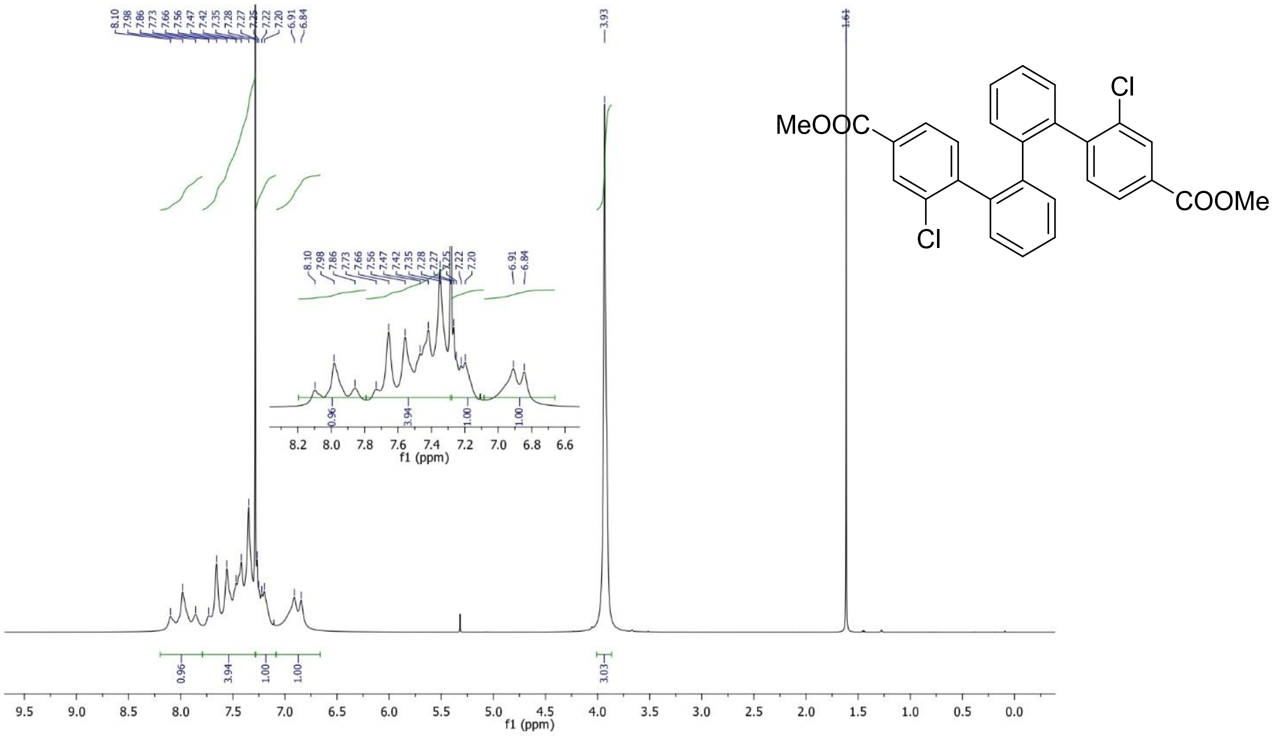

Figure S128. ^{1}H-NMR (600 MHz) spectrum of **13a** in $CDCl_3$.

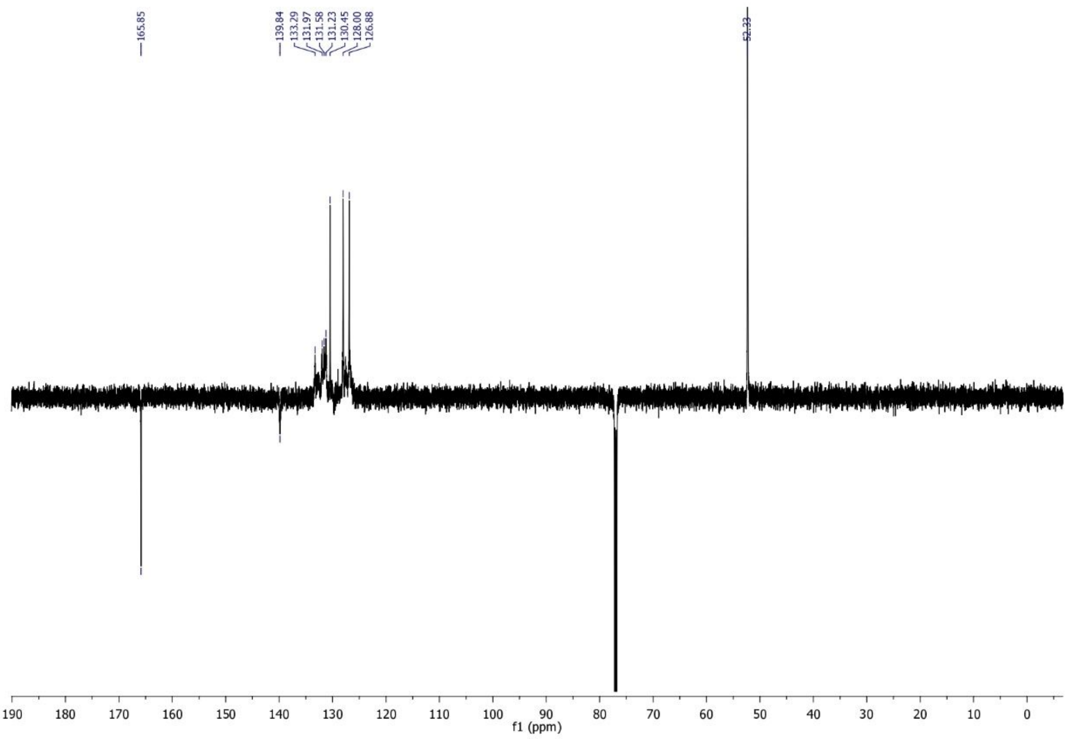

Figure S129. ^{13}C-NMR (151 MHz) spectrum of **13a** in $CDCl_3$ (recorded at 50°C).

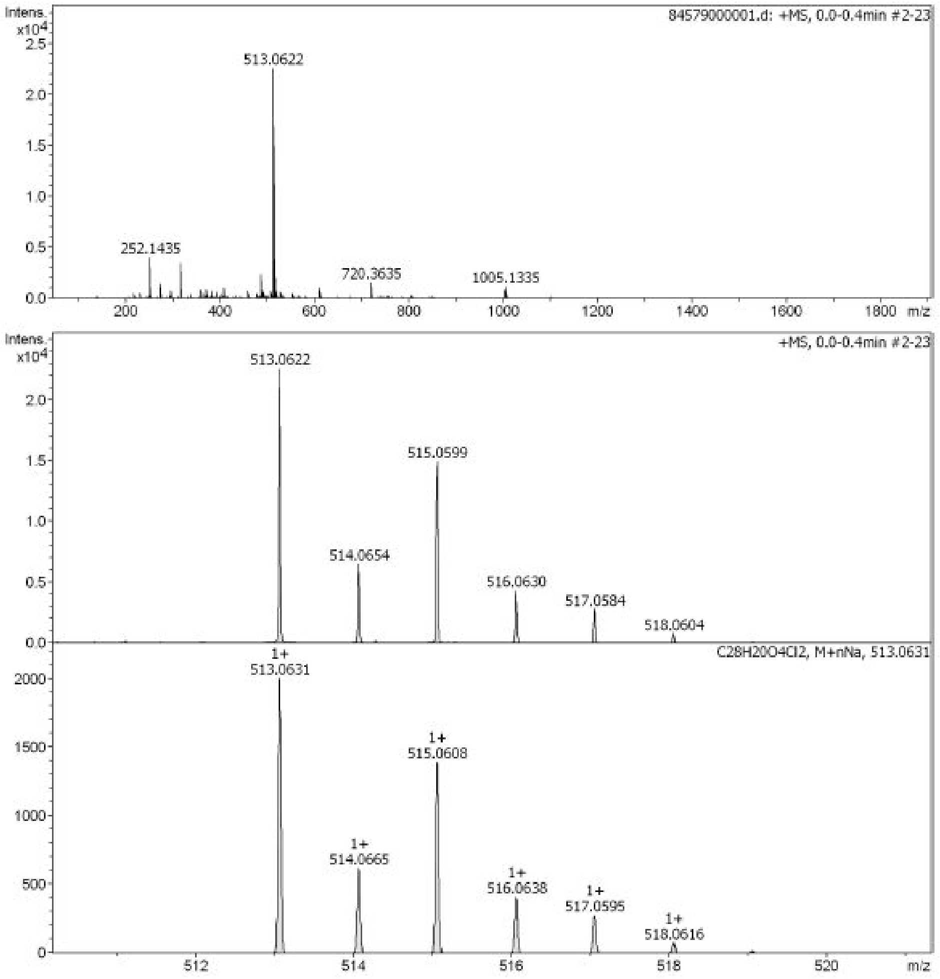

Figure S130. HRMS-LD-TOF mass spectrum of **13a**.

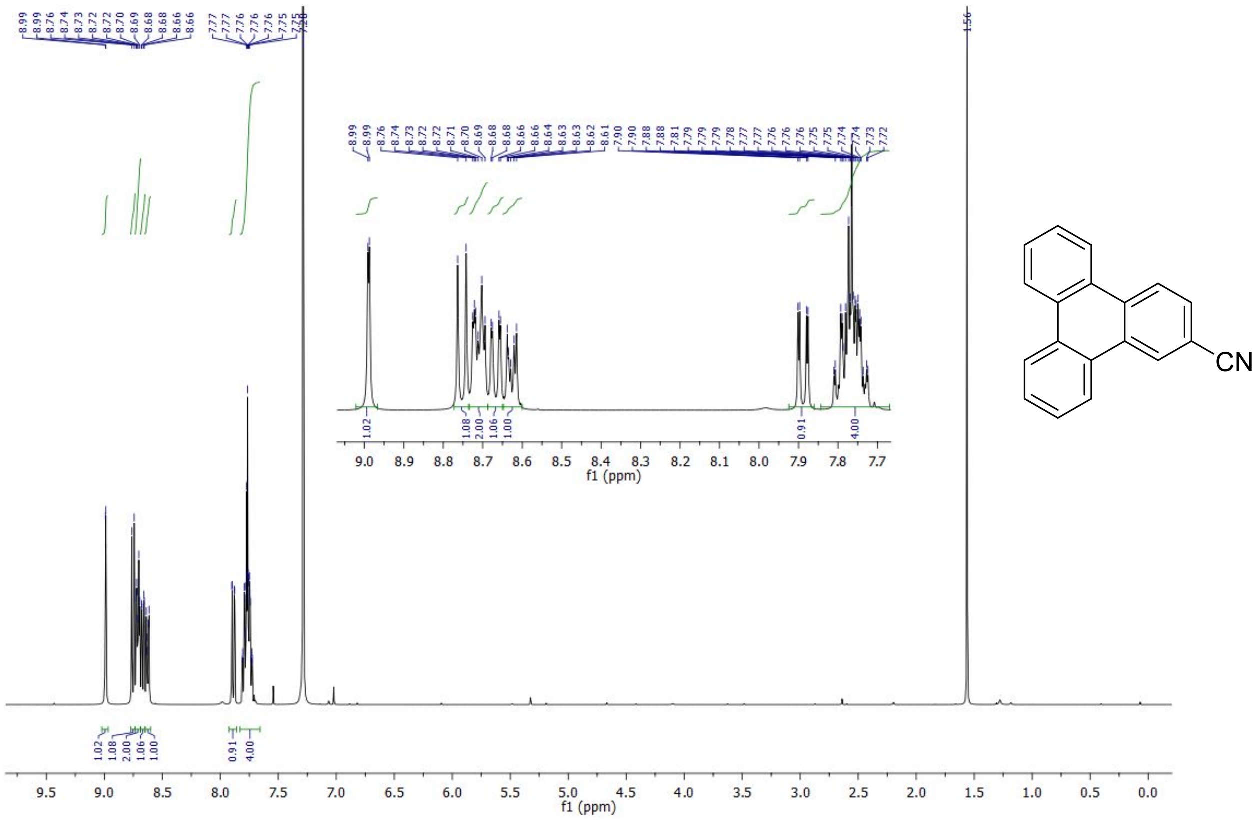

Figure S131. ^{1}H-NMR (600 MHz) spectrum of **2** and **4b** in $CDCl_3$.

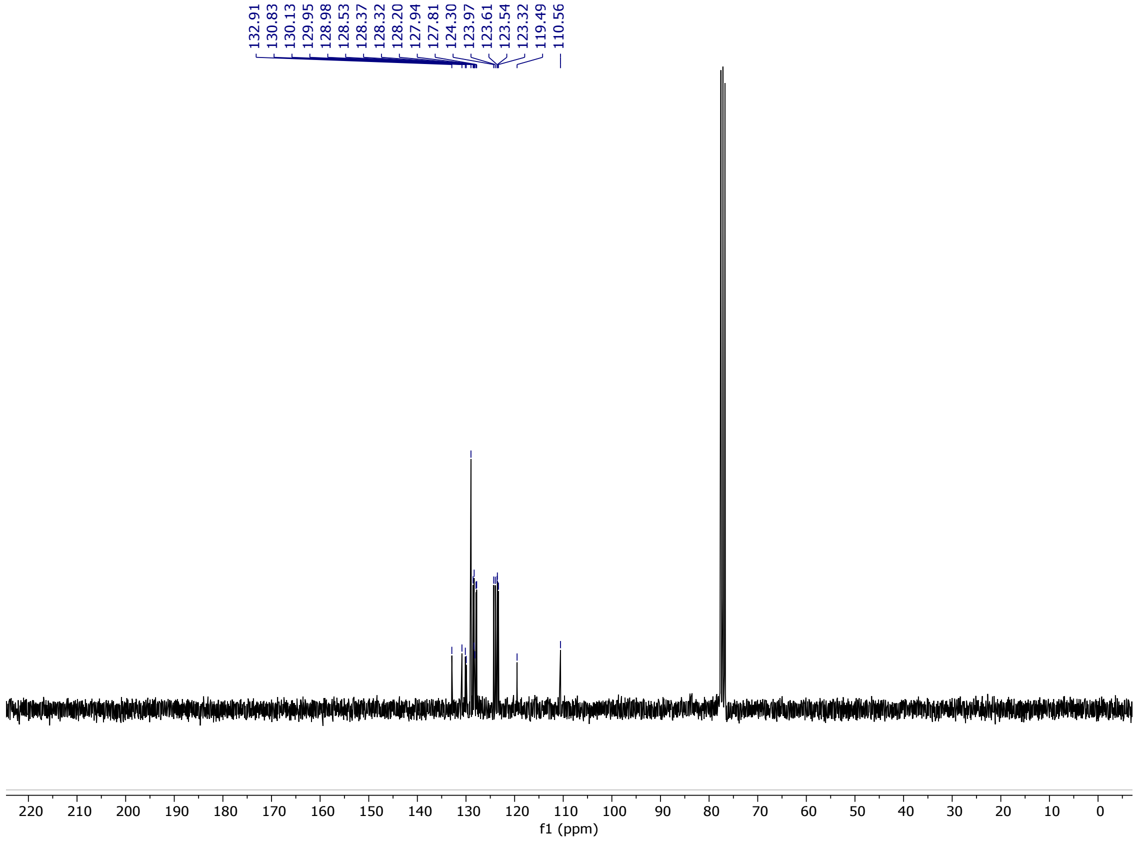

Figure S132. ^{13}C-NMR (75 MHz) spectrum of **2** and **4b** in $CDCl_3$.

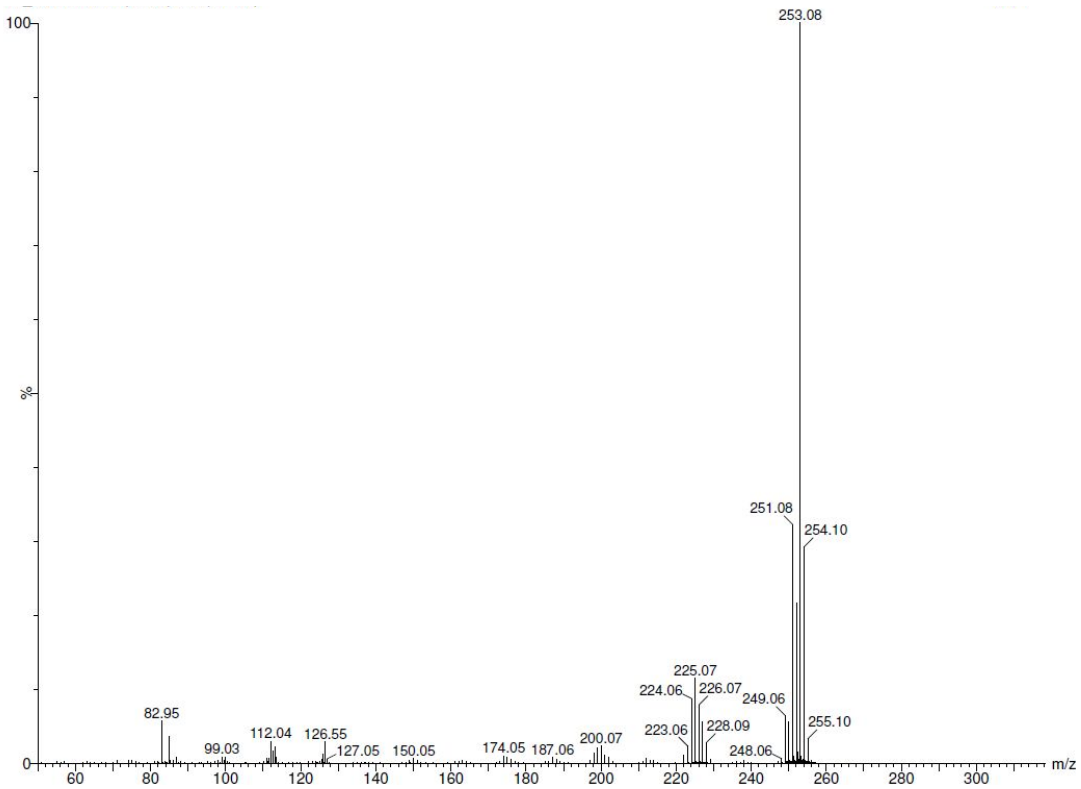

Figure S133. LRMS-ESI mass spectrum of **2** and **4b**.

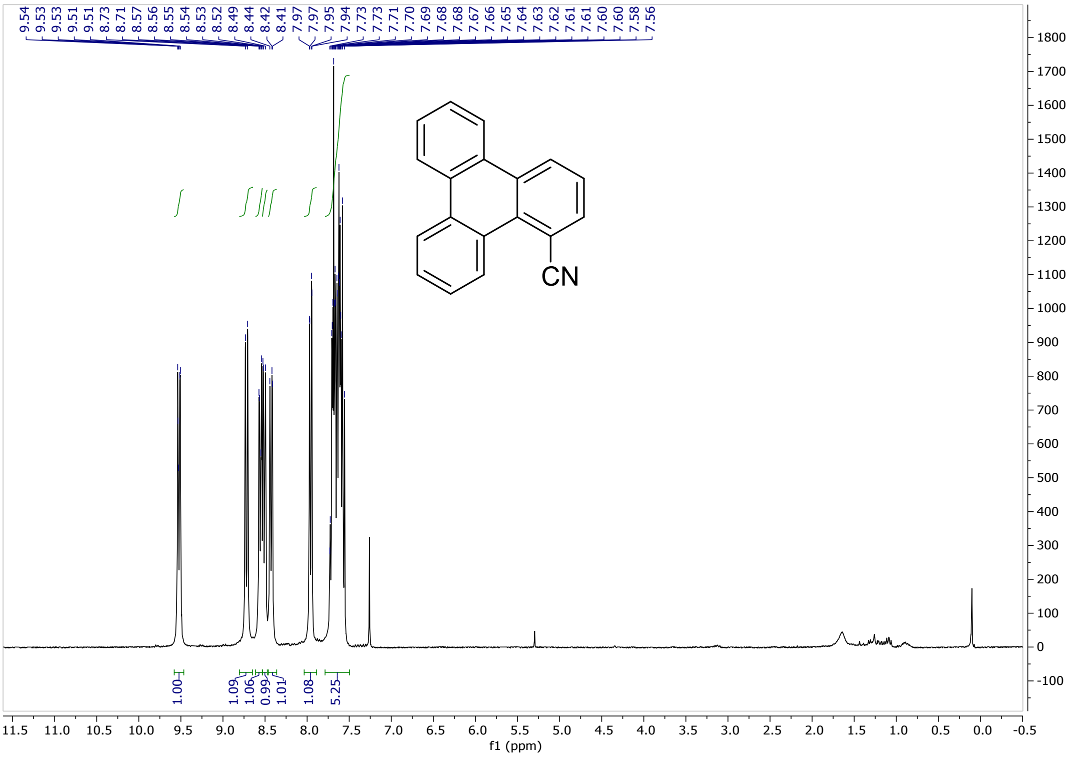

Figure S134. ^{1}H-NMR (300 MHz) spectrum of **4a** in $CDCl_3$.

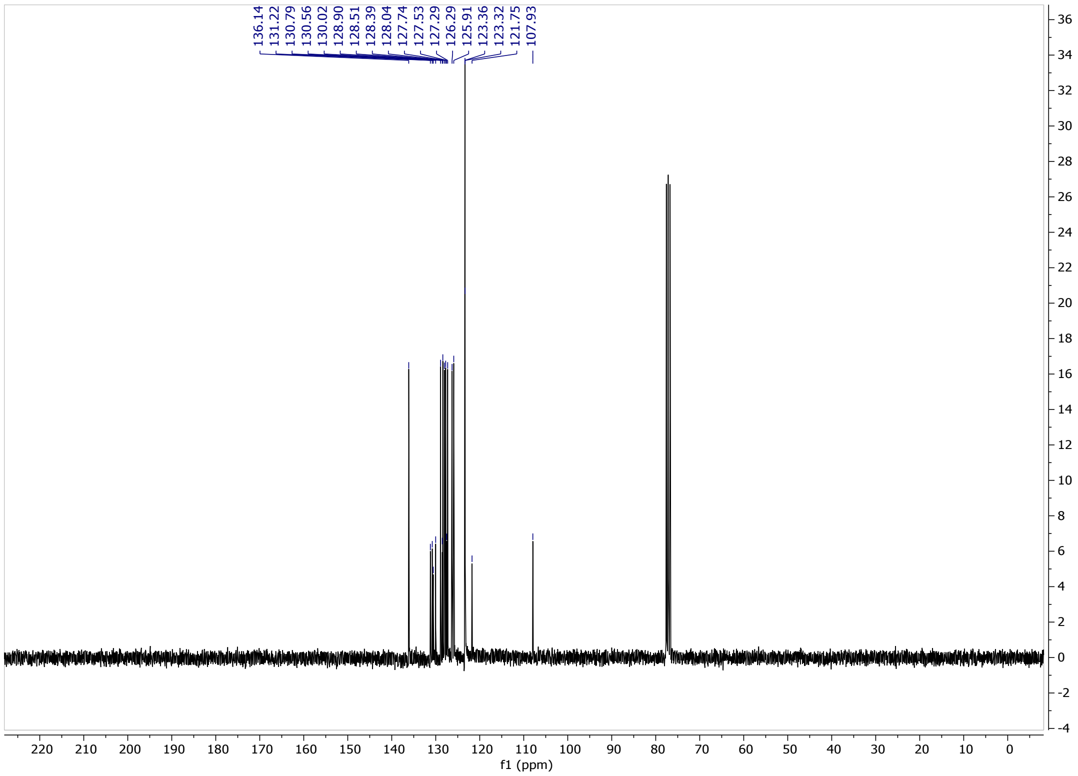

Figure S135. ^{13}C-NMR (75 MHz) spectrum of **4a** in $CDCl_3$.

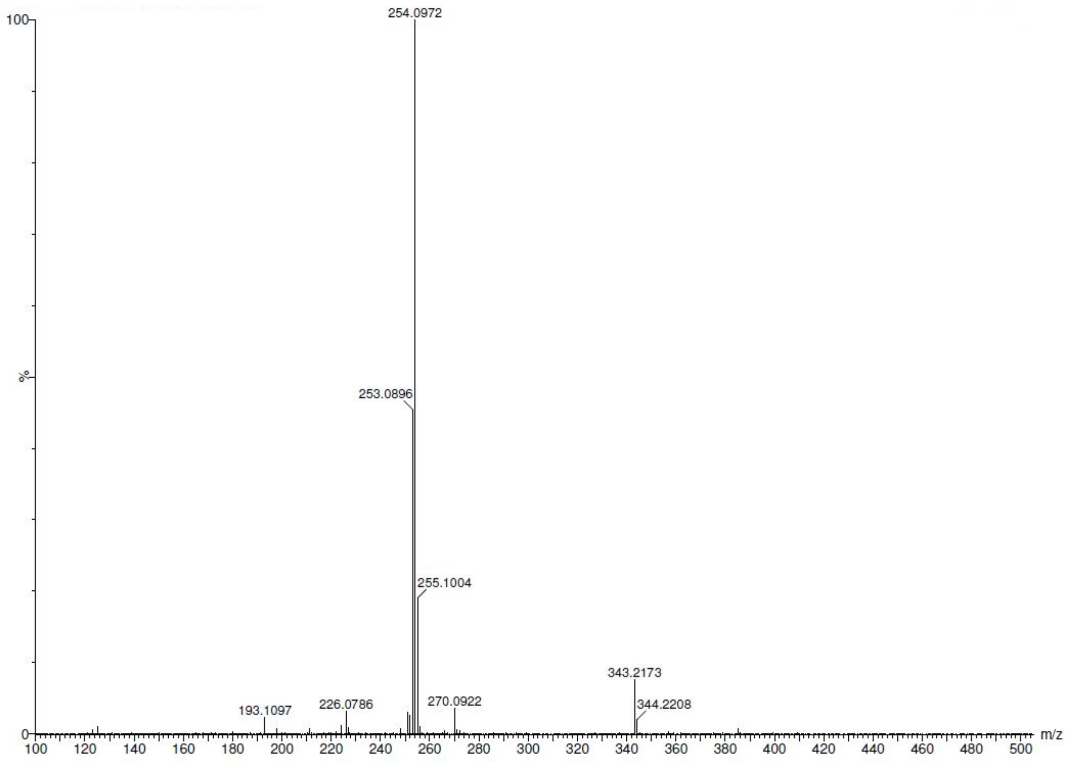

Figure S136. HRMS-APCI TOF mass spectrum of **4a**.

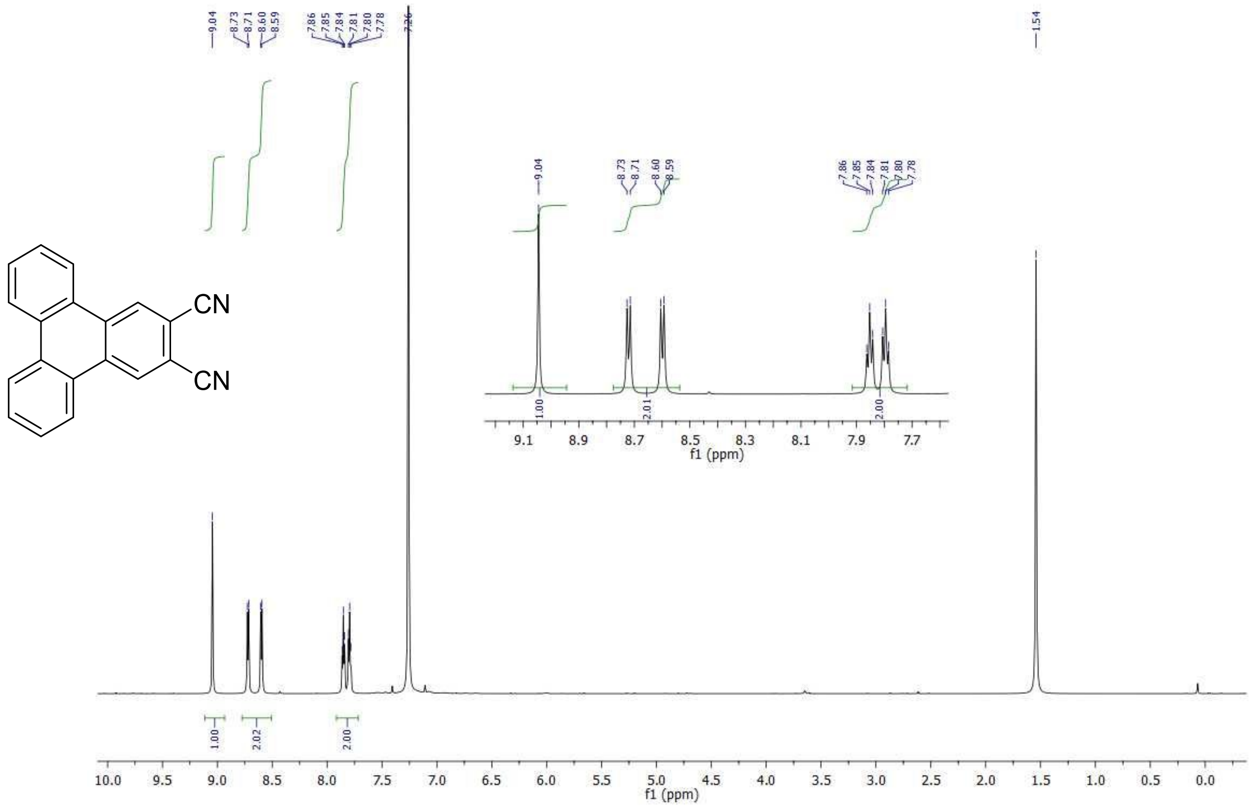

Figure S137. ^{1}H-NMR (600 MHz) spectrum of **4c** in $CDCl_3$.

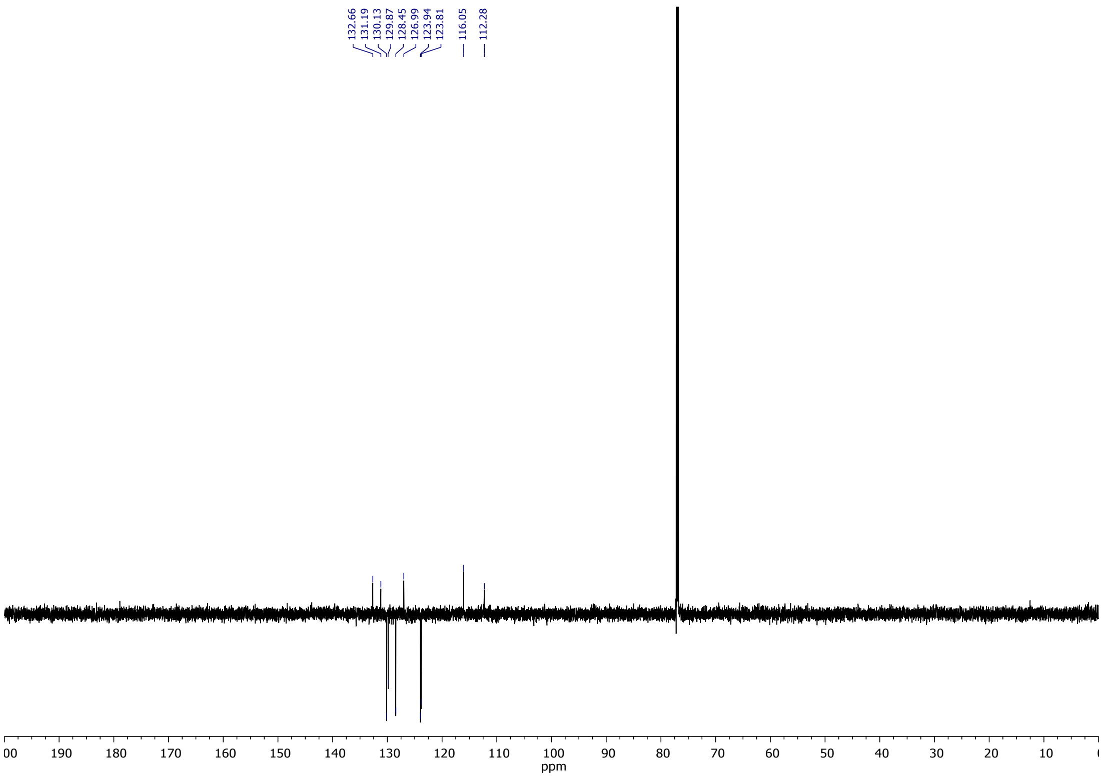

Figure S138. ^{13}C-NMR (151 MHz) spectrum of **4c** in $CDCl_3$.

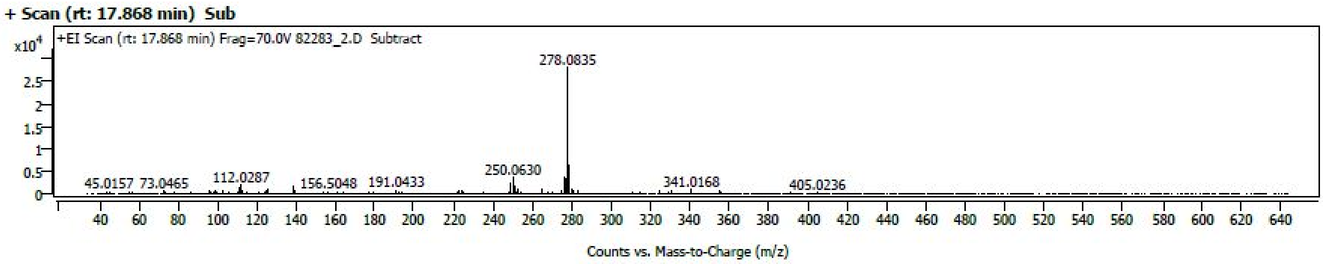

Figure S139. HRGC-MS mass spectrum of **4c**.

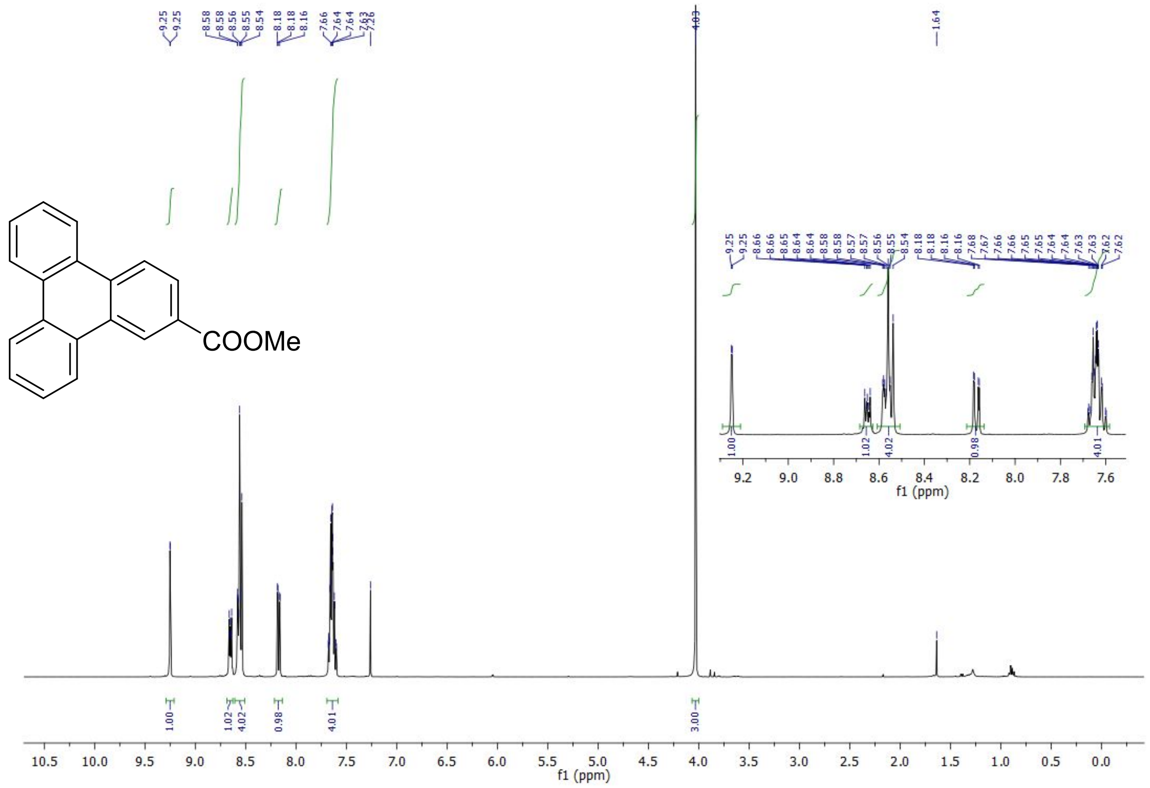

Figure S140. ^{1}H-NMR (600 MHz) spectrum of **4d** and **4e** in $CDCl_3$.

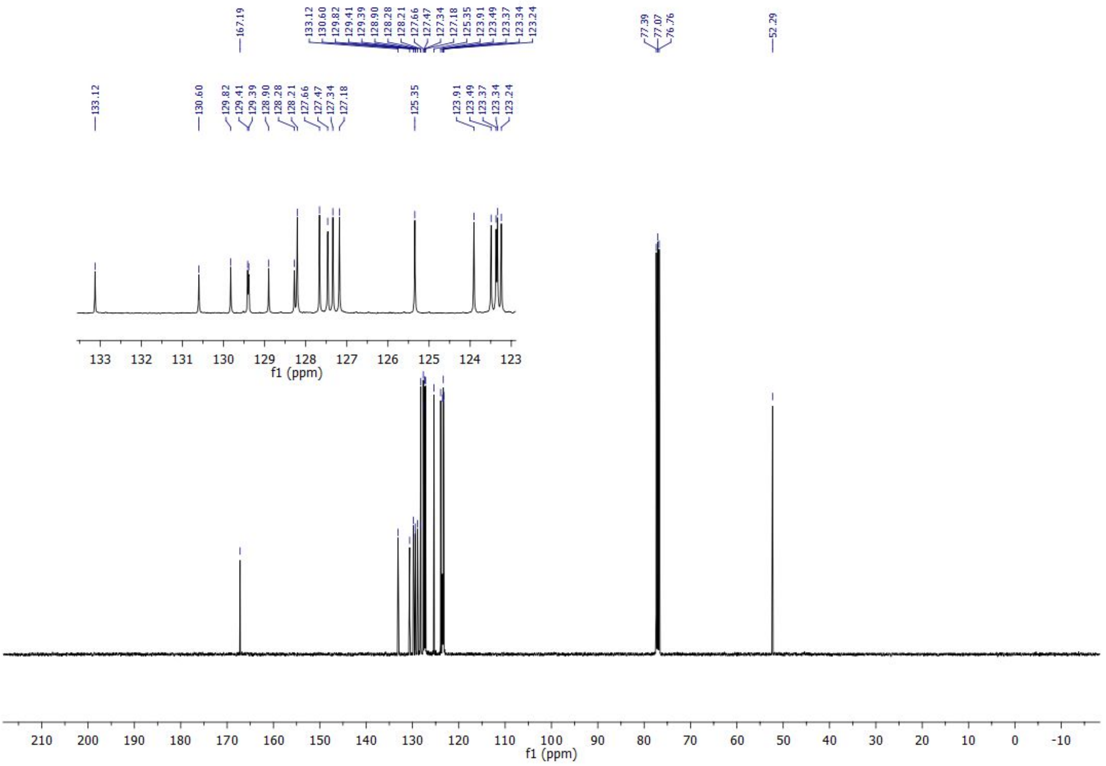

Figure S141. ^{13}C-NMR (151 MHz) spectrum of **4d** and **4e** in $CDCl_3$.

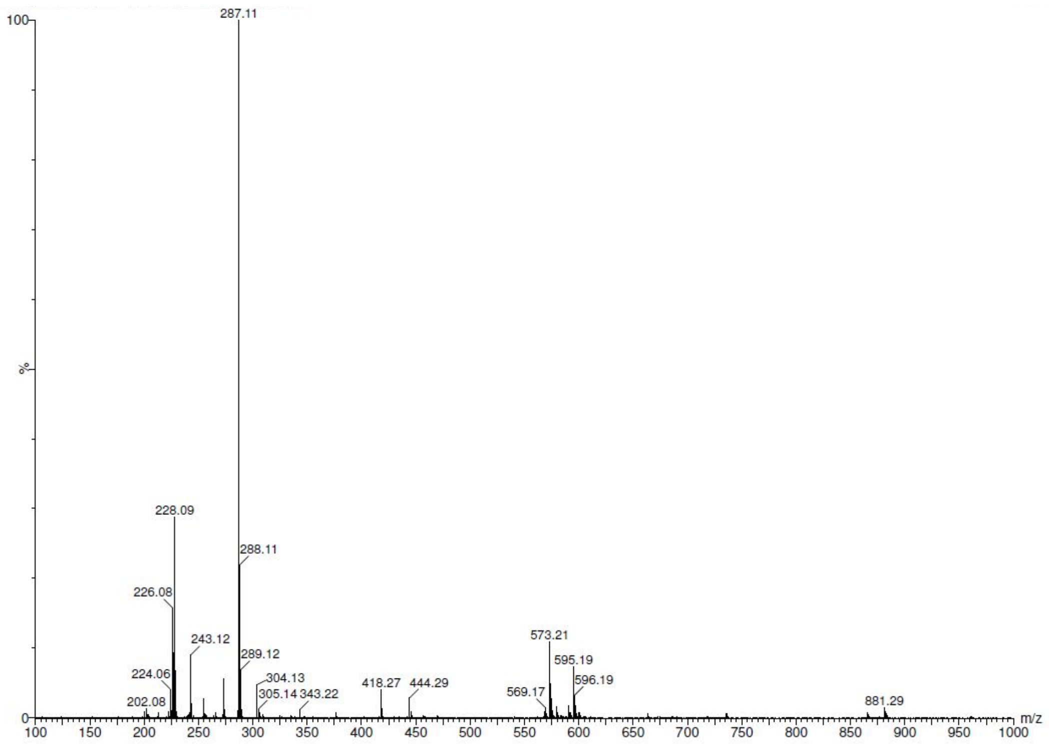

Figure S142. LRMS-ESI-TOF mass spectrum of **4d** and **4e**.

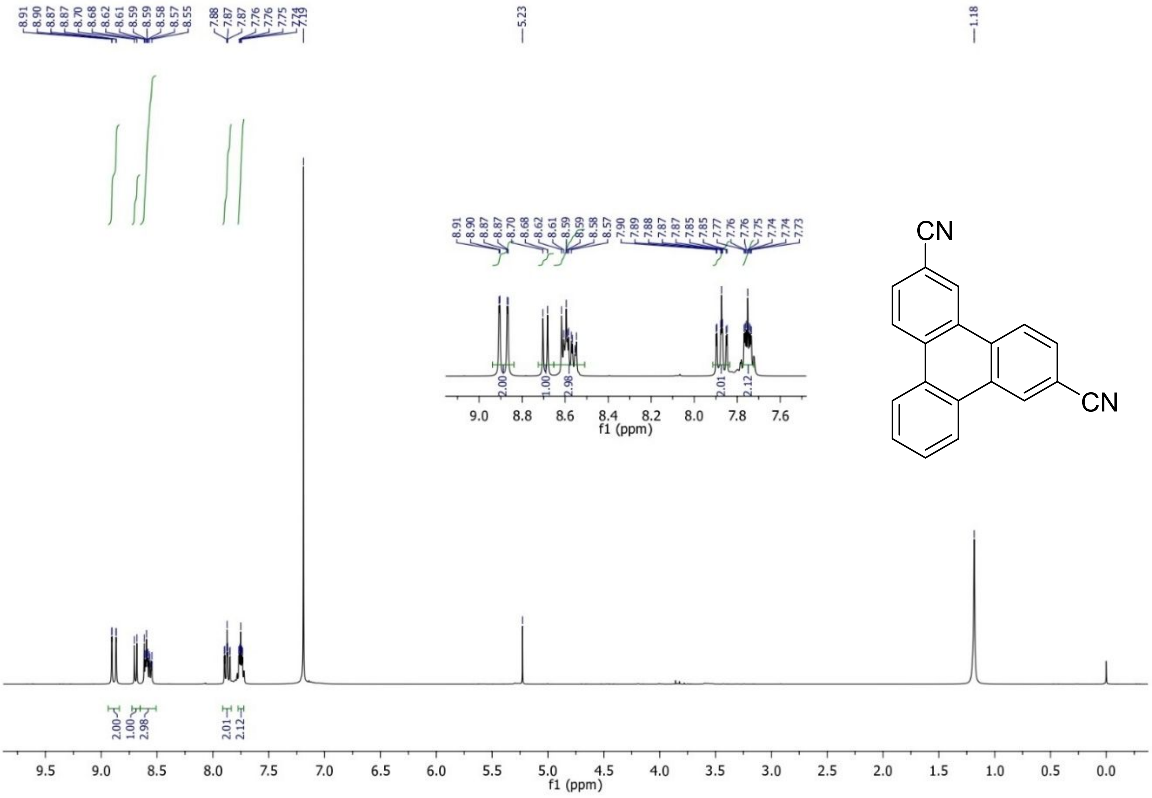

Figure S143. ^{1}H-NMR (600 MHz) spectrum of **4f** in $CDCl_3$.

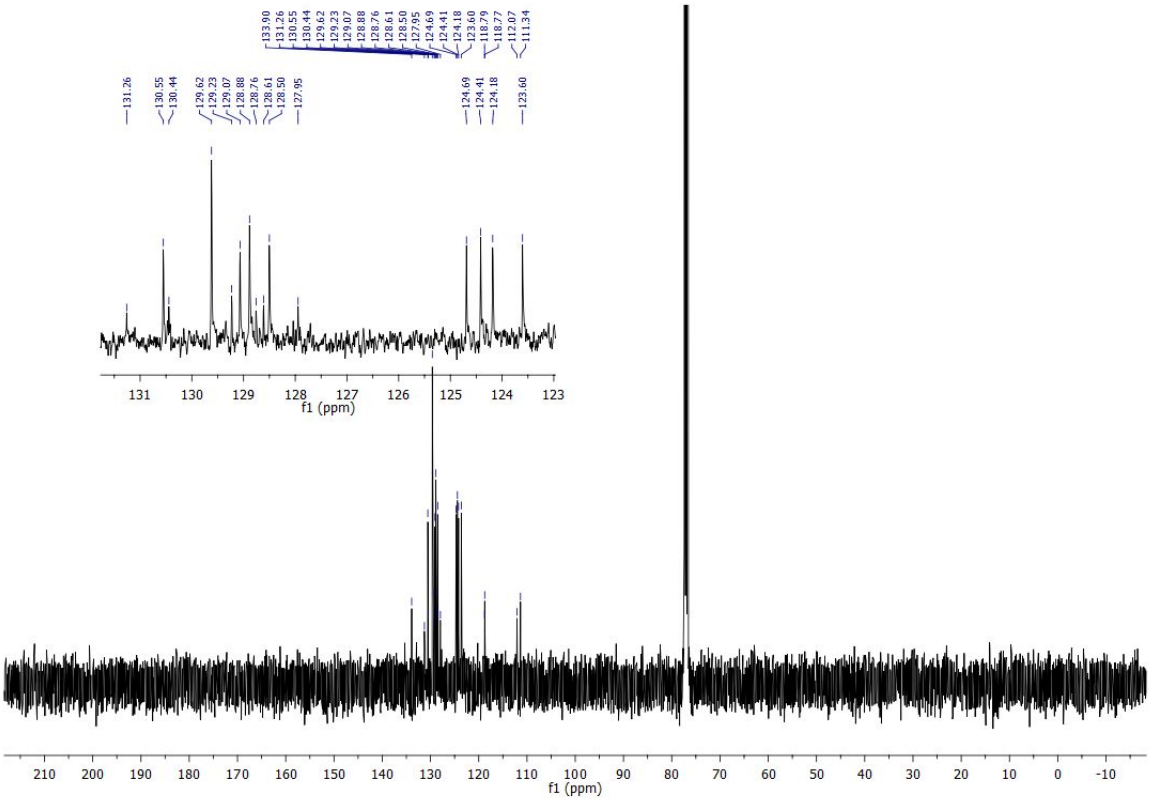

Figure S144. ^{13}C-NMR (151 MHz) spectrum of **4f** in $CDCl_3$.

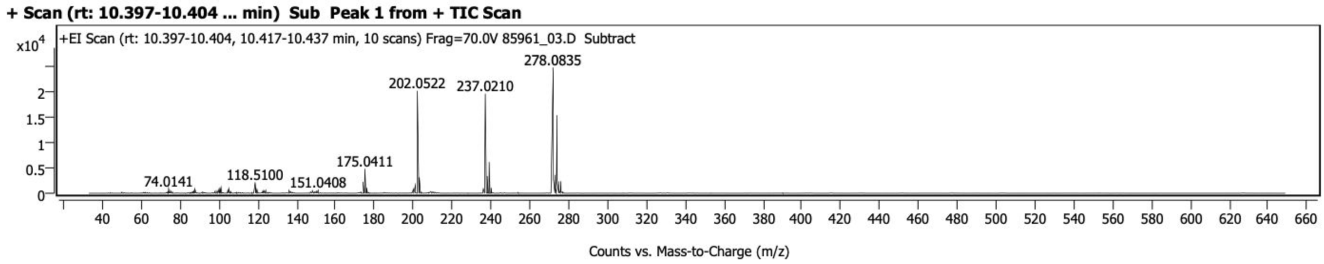

Figure S145. HRGC-MS mass spectrum of **4f.**

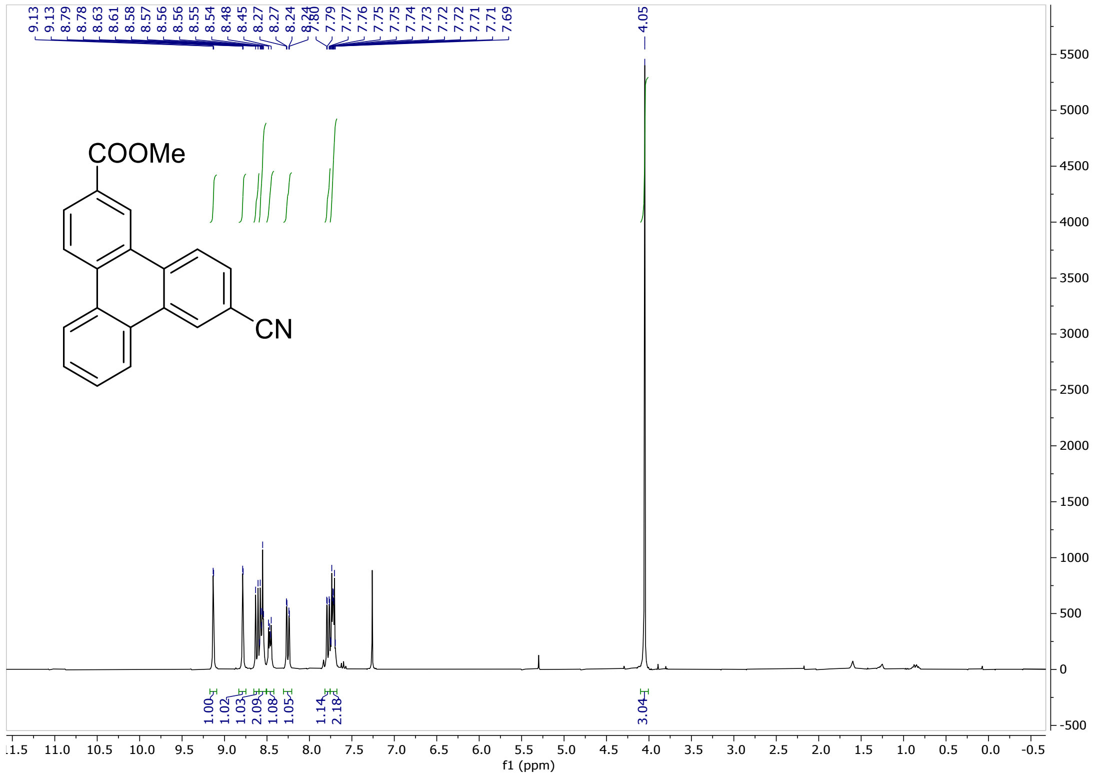

Figure S10. ^{1}H-NMR (300 MHz) spectrum of **4g** in $CDCl_3$.

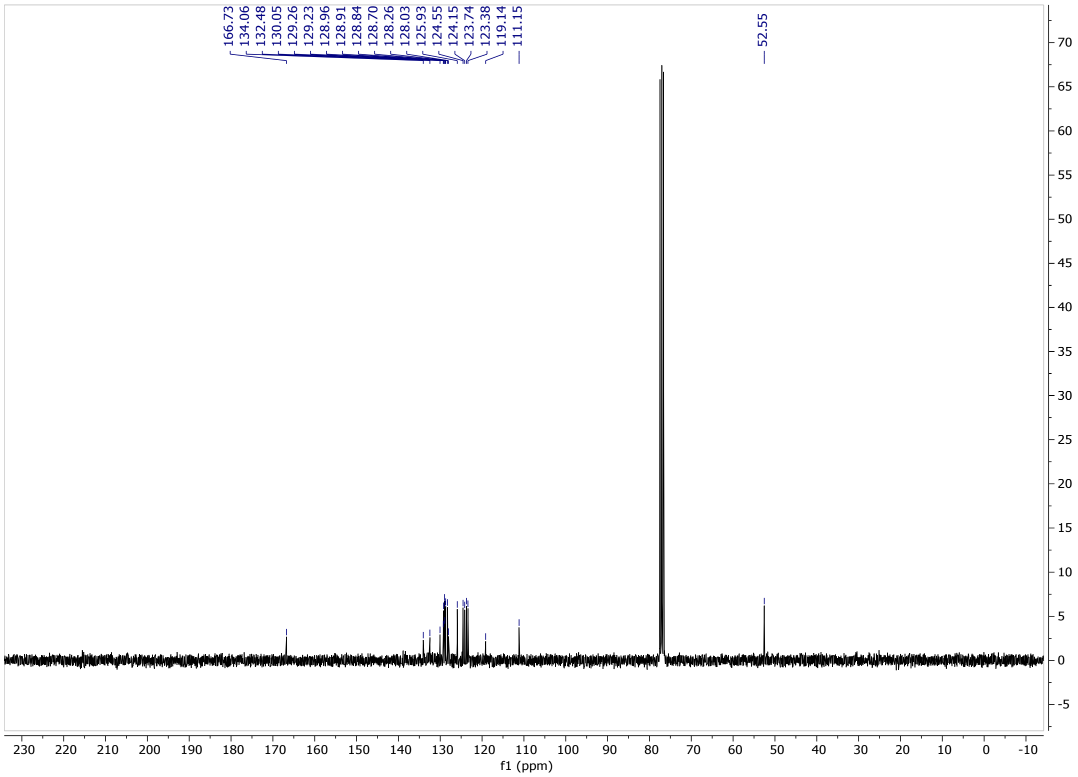

Figure S11. ^{13}C-NMR (75 MHz) spectrum of **4g** in $CDCl_3$.

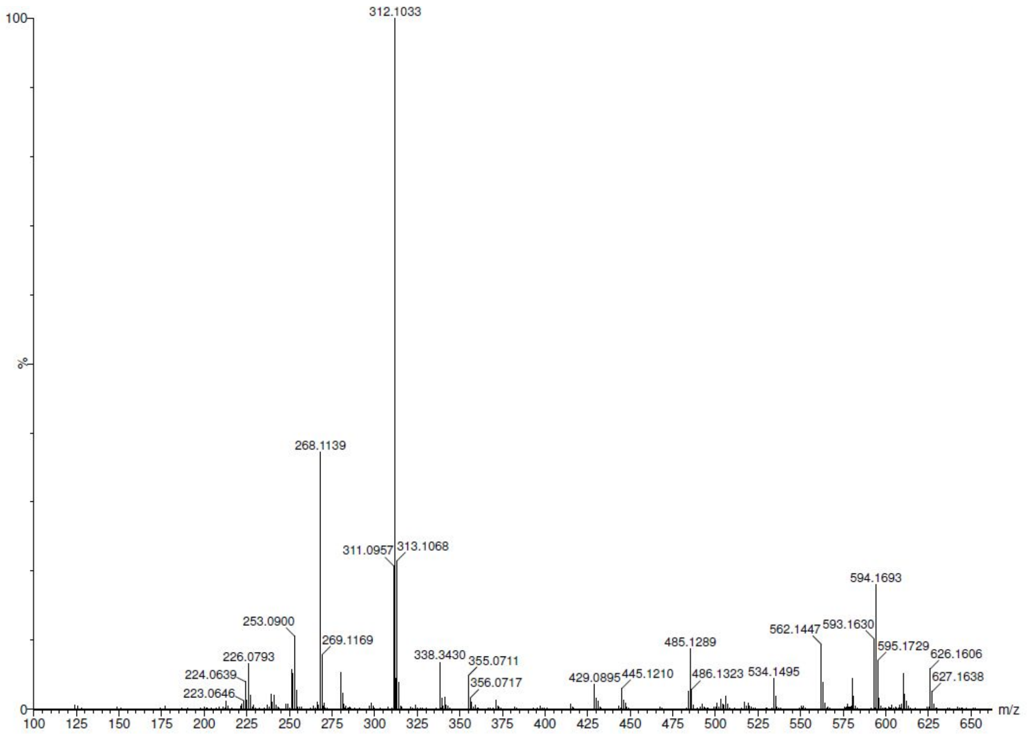

Figure S12. HRMS-APCI TOF mass spectrum of **4g**.

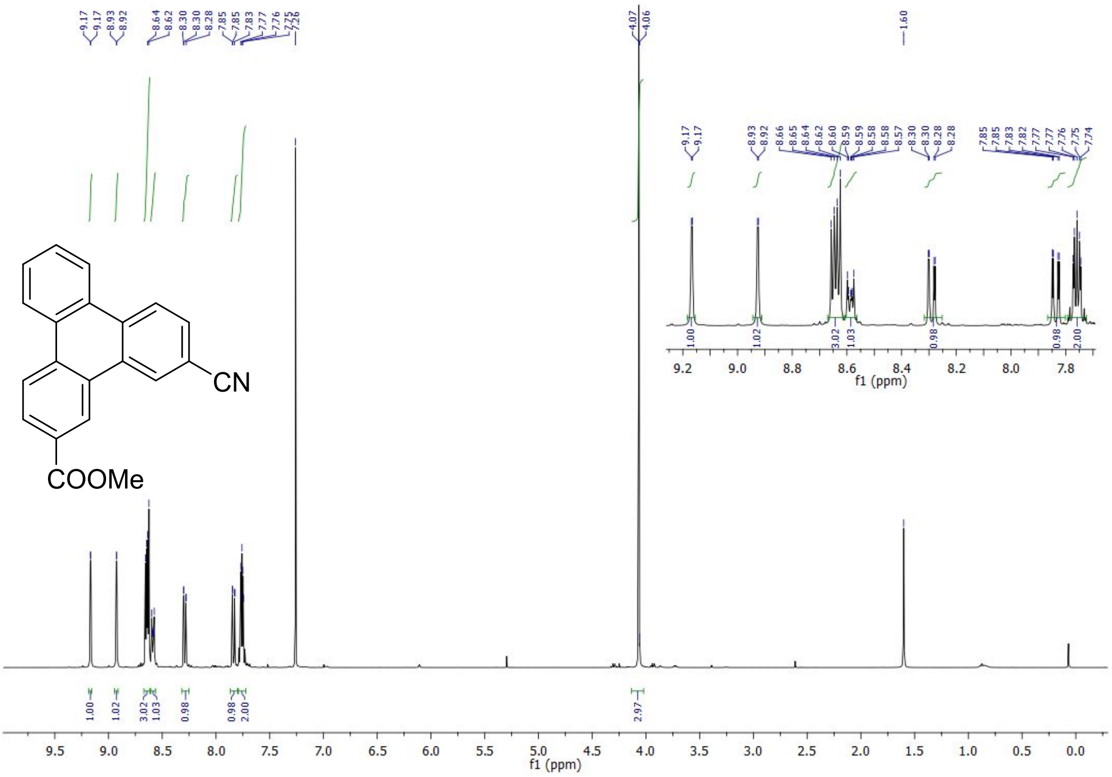

Figure S149. ^{1}H-NMR (600 MHz) spectrum of **4j** in $CDCl_3$.

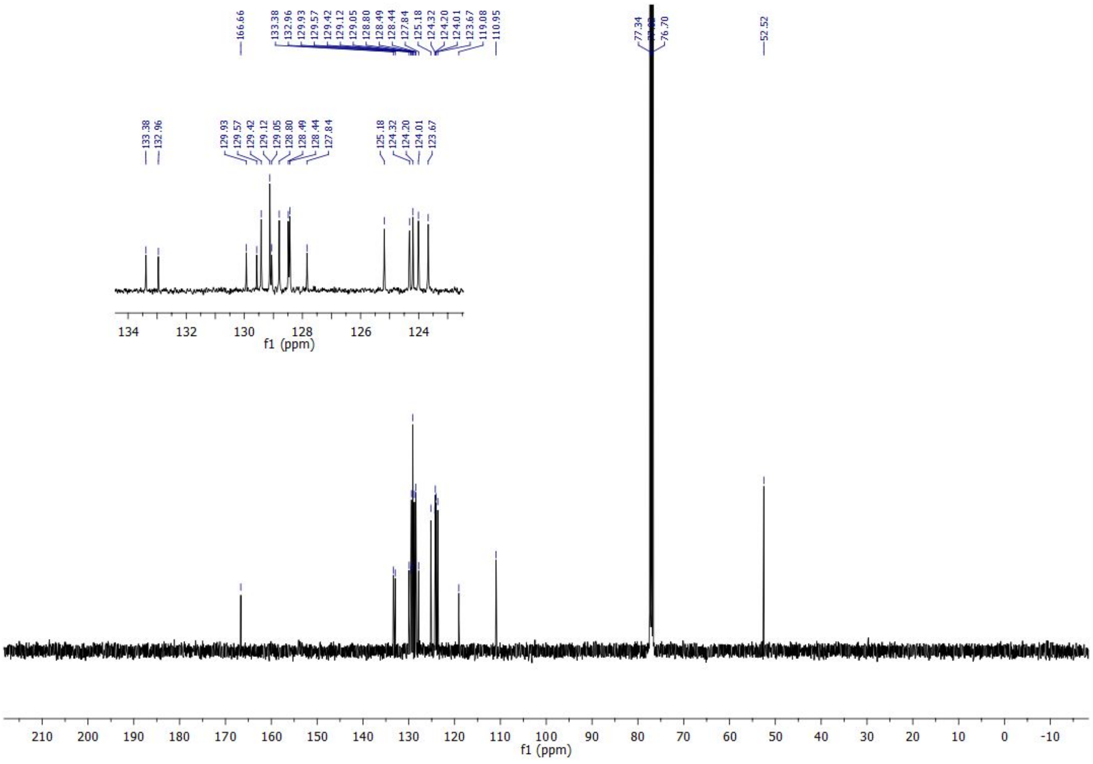

Figure S150. ^{13}C-NMR (151 MHz) spectrum of **4j** in $CDCl_3$.

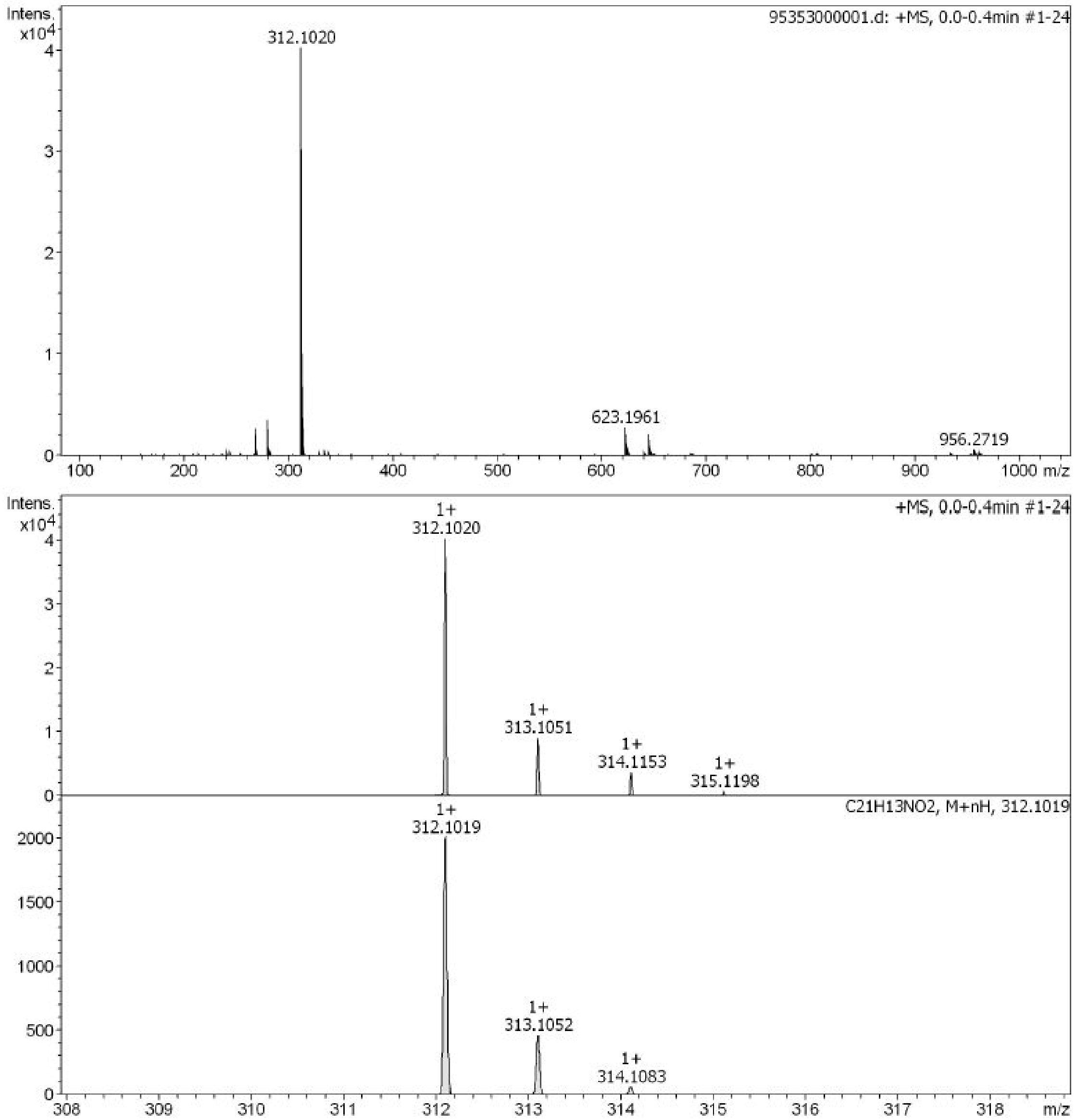

Figure S151. HRMS-LD-TOF mass spectrum of **4j**.

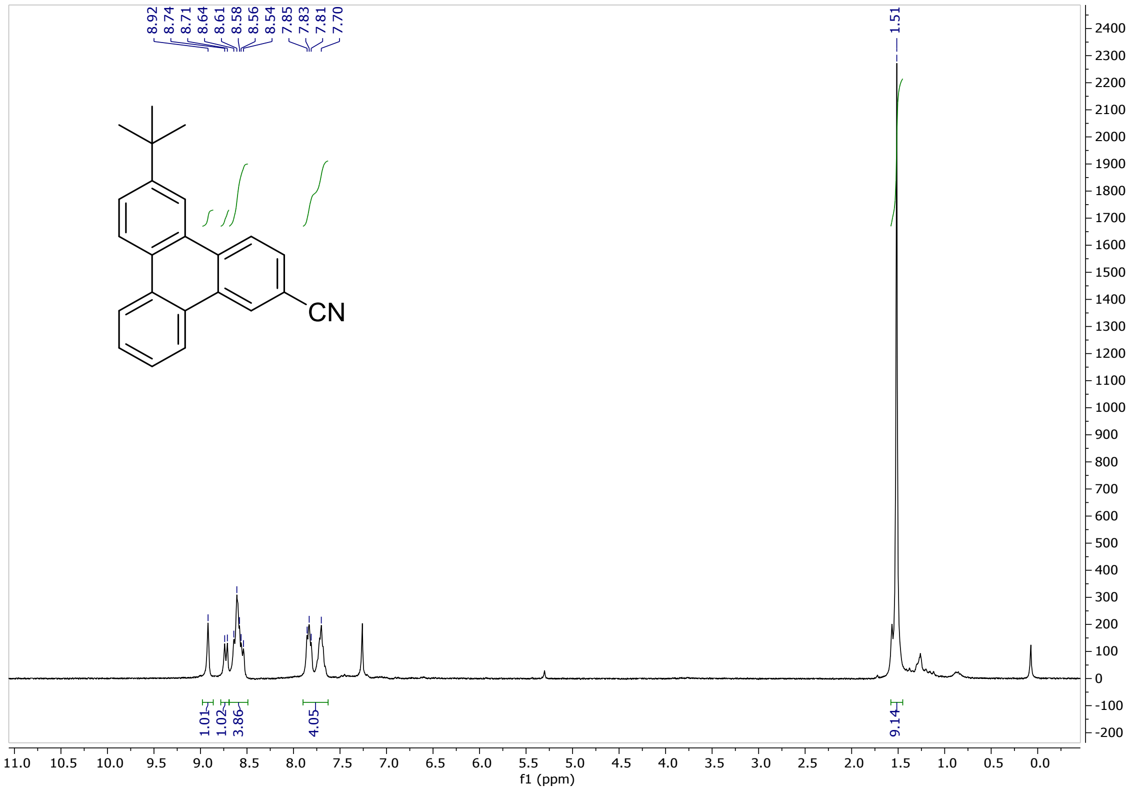

Figure S152. ^{1}H-NMR (300 MHz) spectrum of **4k** in $CDCl_3$.

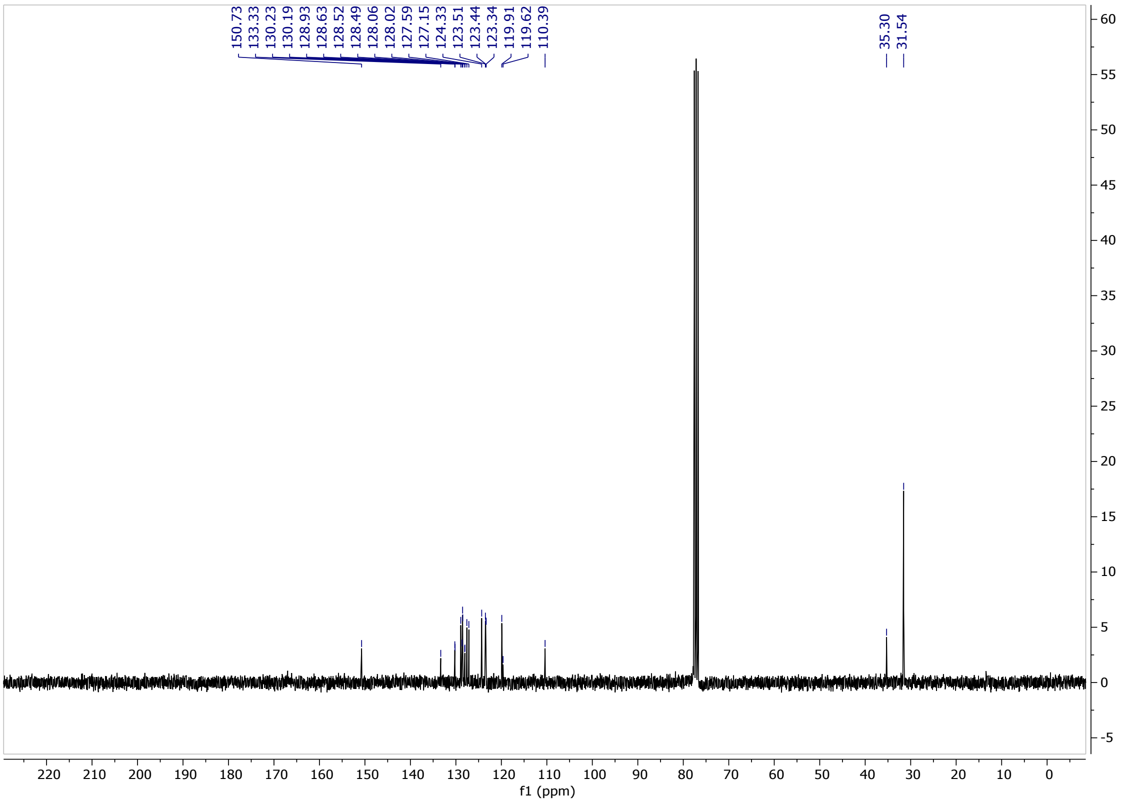

Figure S153. ^{13}C-NMR (75 MHz) spectrum of **4k** in $CDCl_3$.

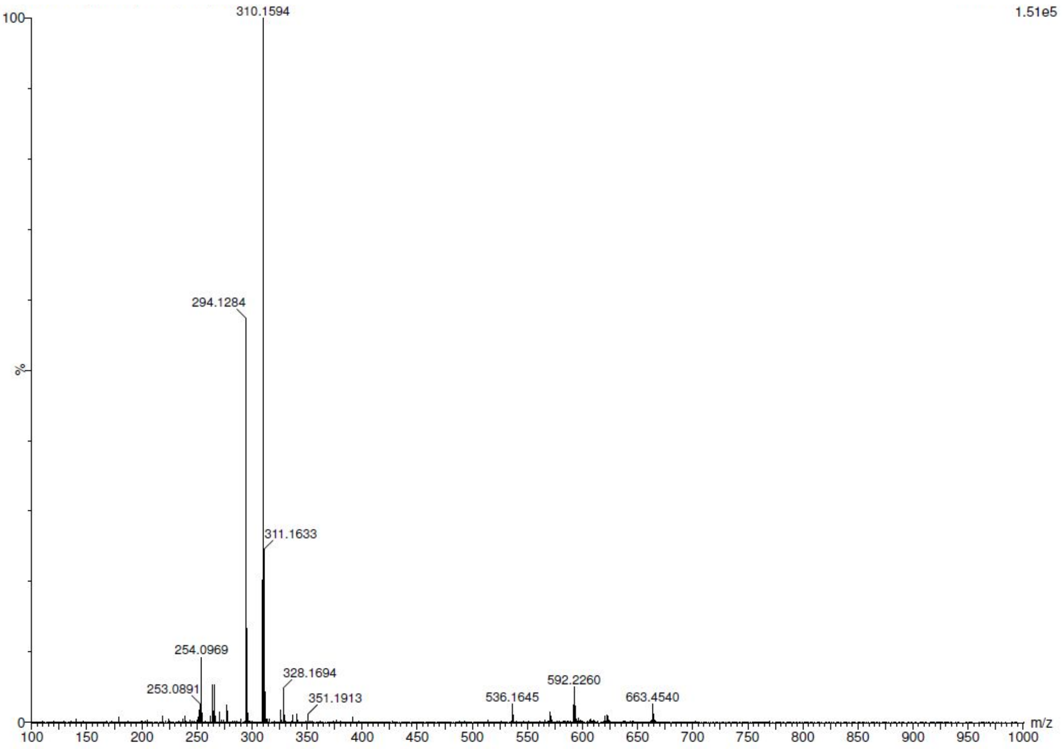

Figure S154. HRMS-APCI TOF mass spectrum of **4k**.

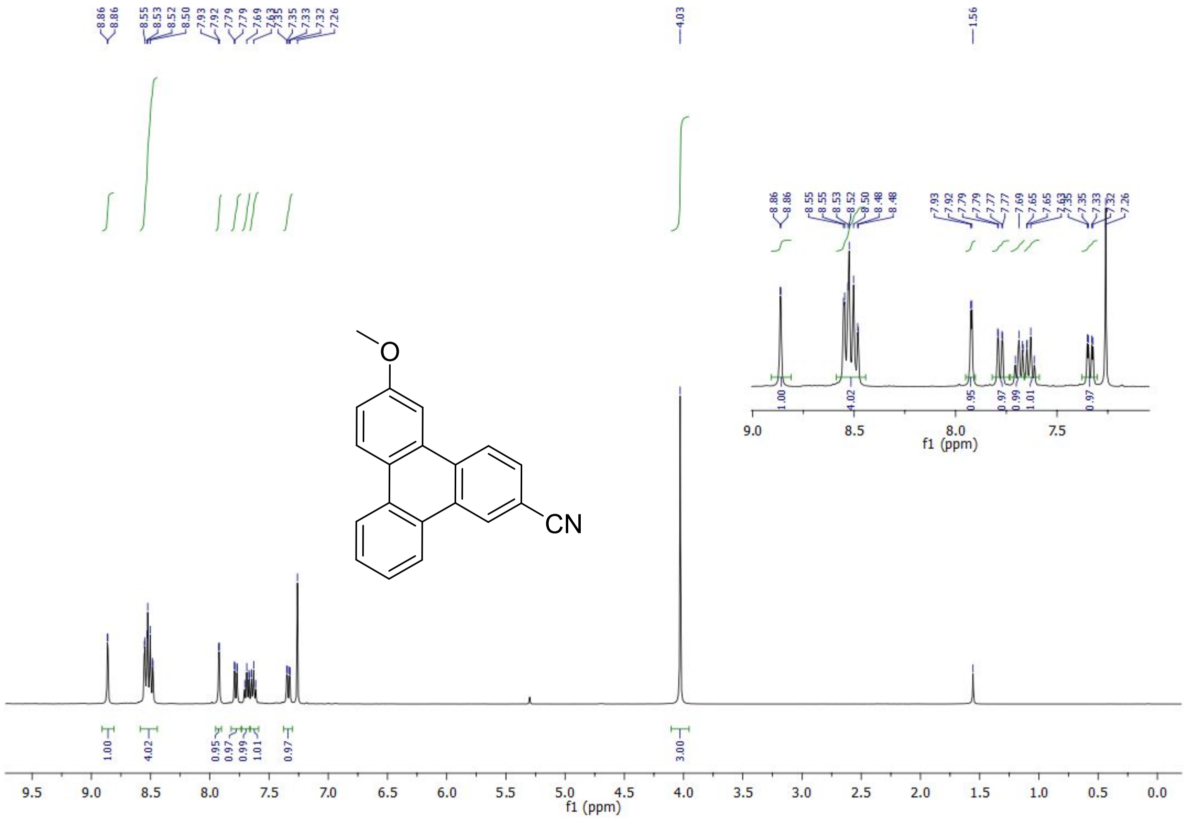

Figure S155. ^{1}H-NMR (600 MHz) spectrum of **4l** in $CDCl_3$.

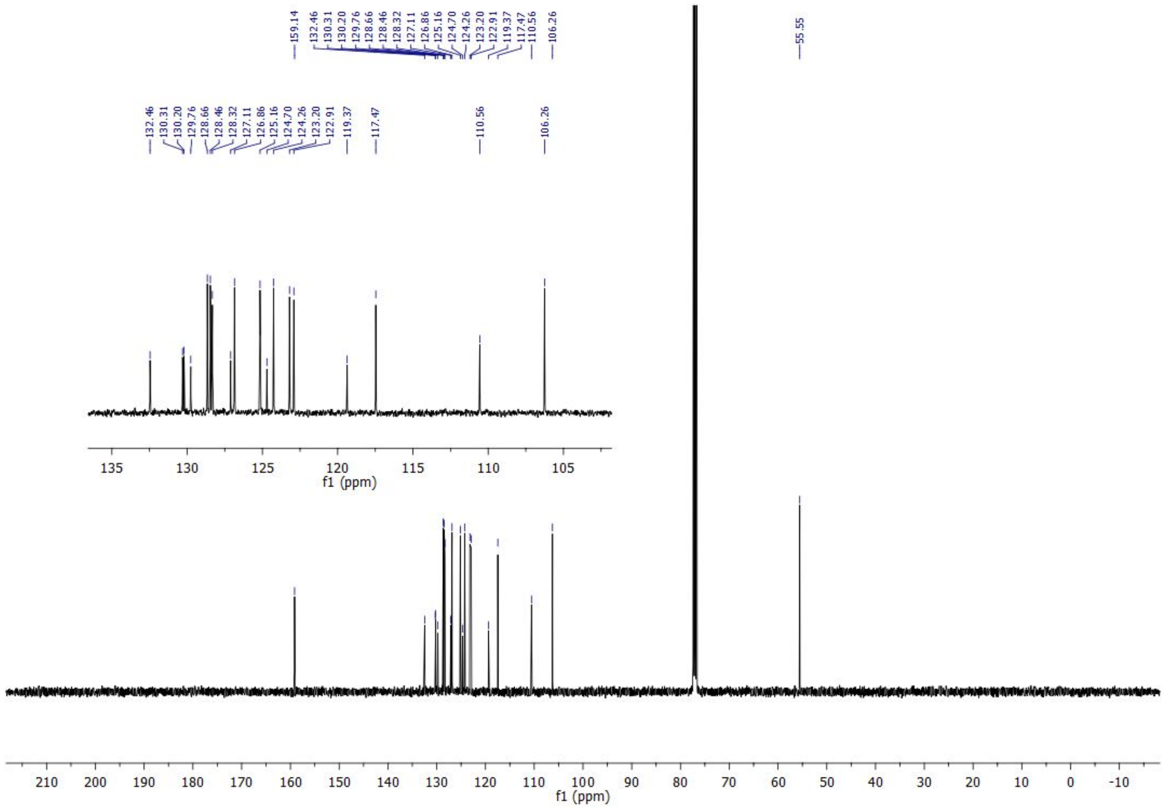

Figure S156. ^{13}C-NMR (151 MHz) spectrum of **4l** in $CDCl_3$.

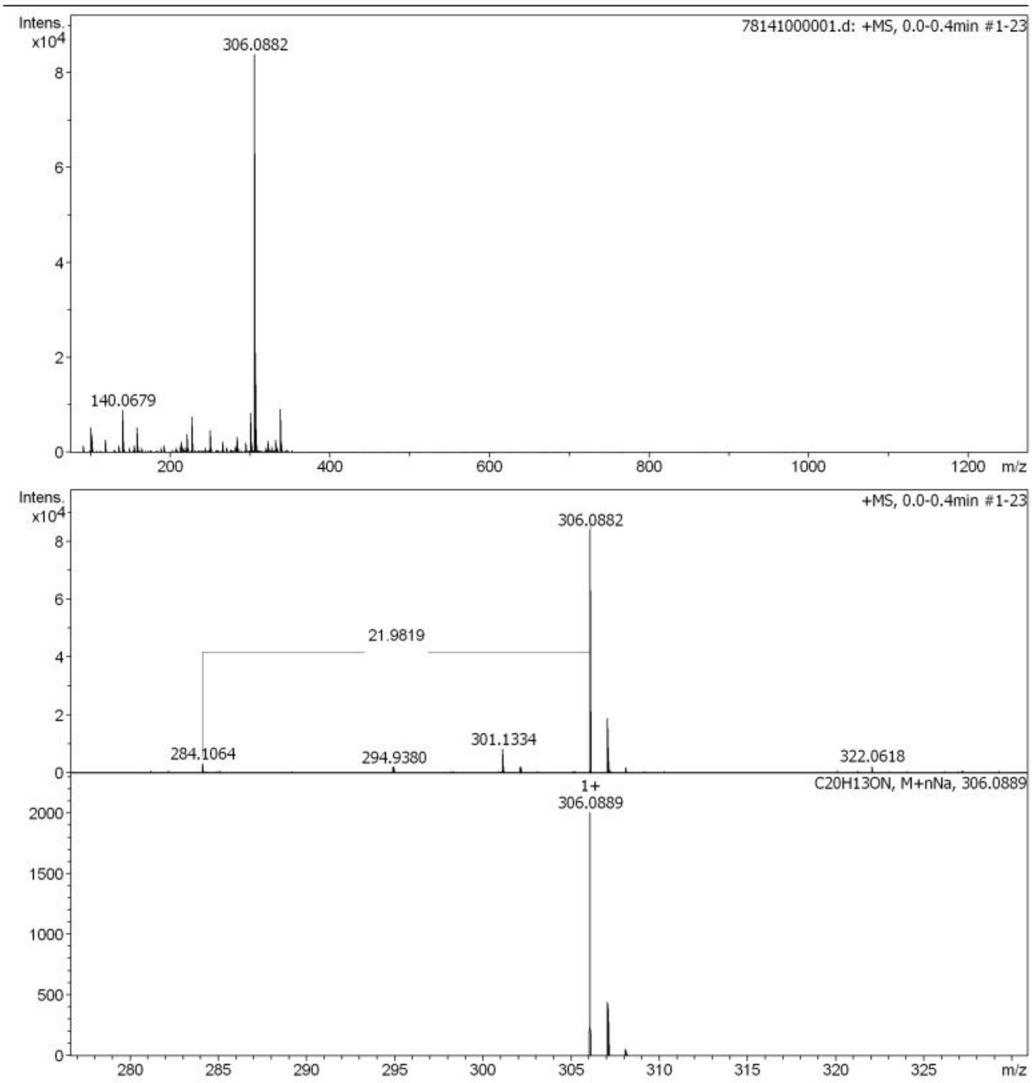

Figure S157. HRMS-ESI mass spectrum of **4l.**

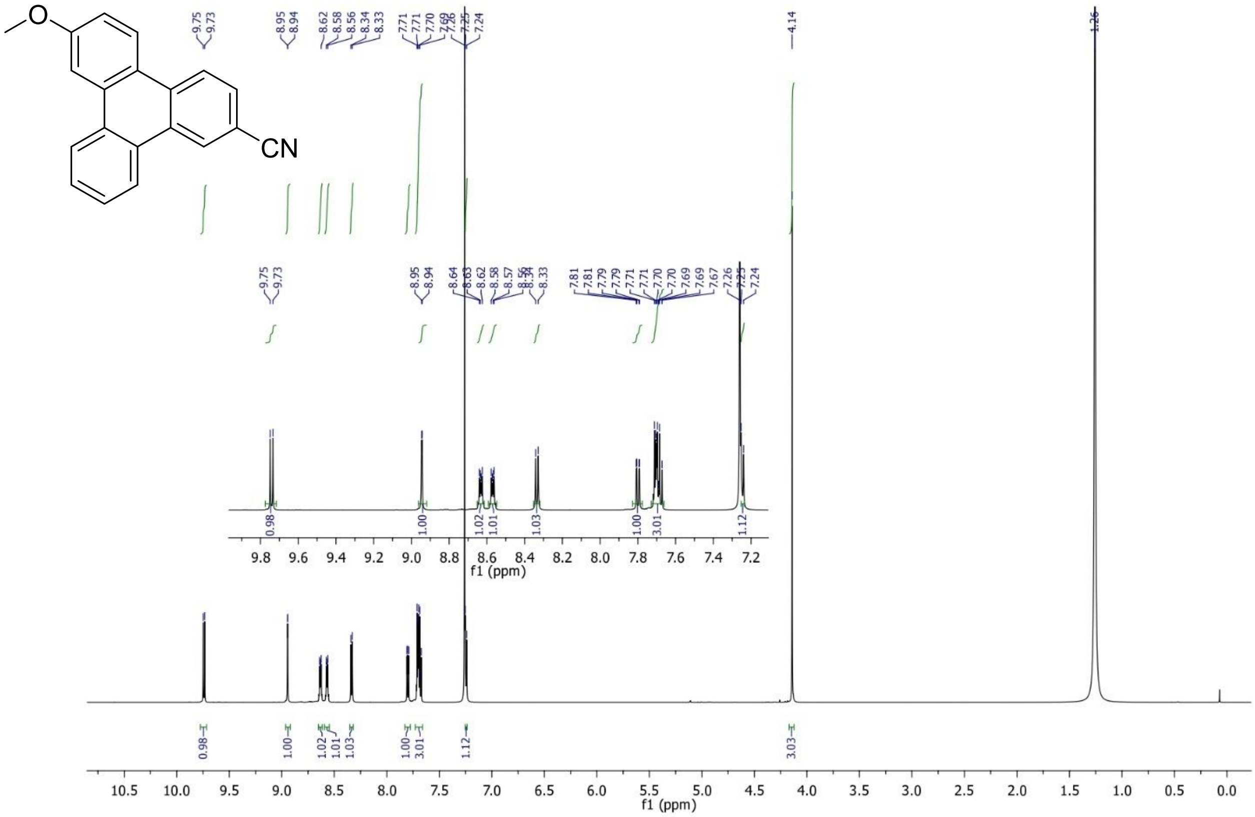

Figure S158. ^{1}H-NMR (600 MHz) spectrum of **4m** in $CDCl_3$.

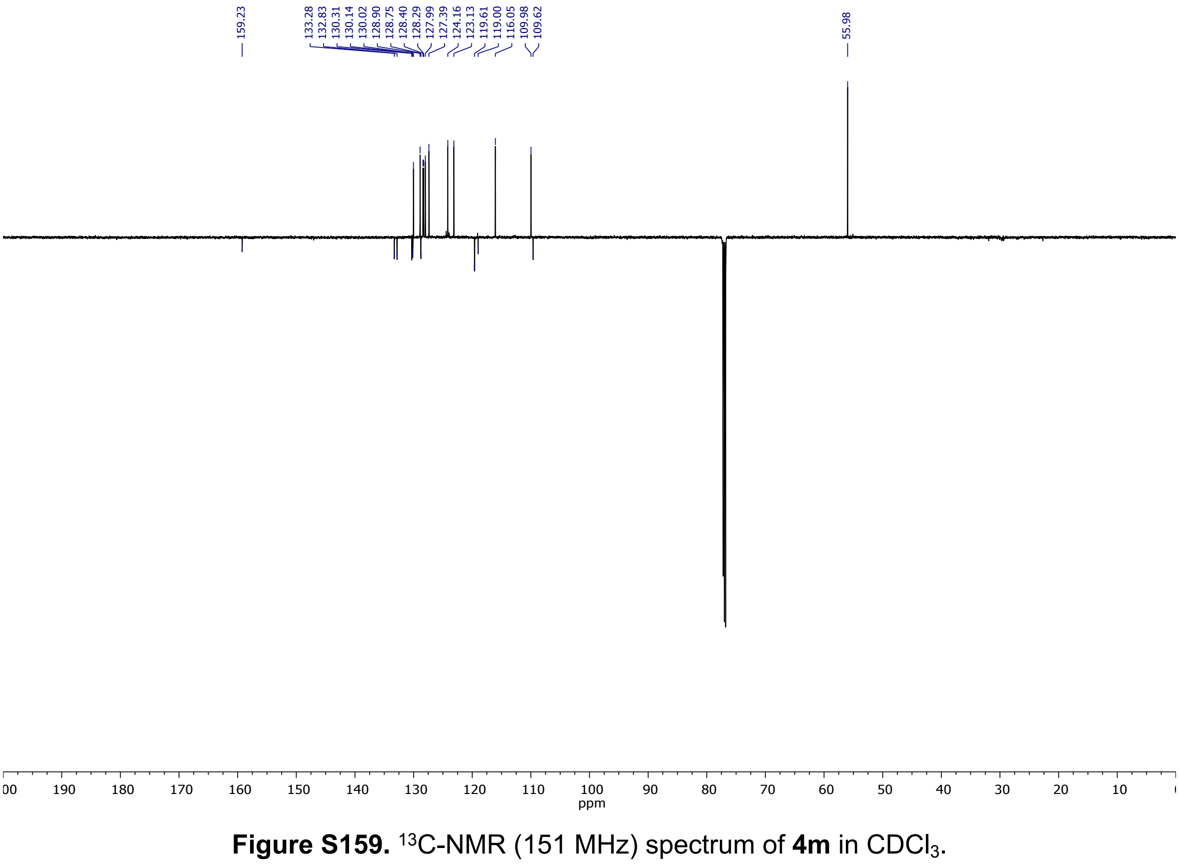

Figure S159. ^{13}C-NMR (151 MHz) spectrum of **4m** in $CDCl_3$.

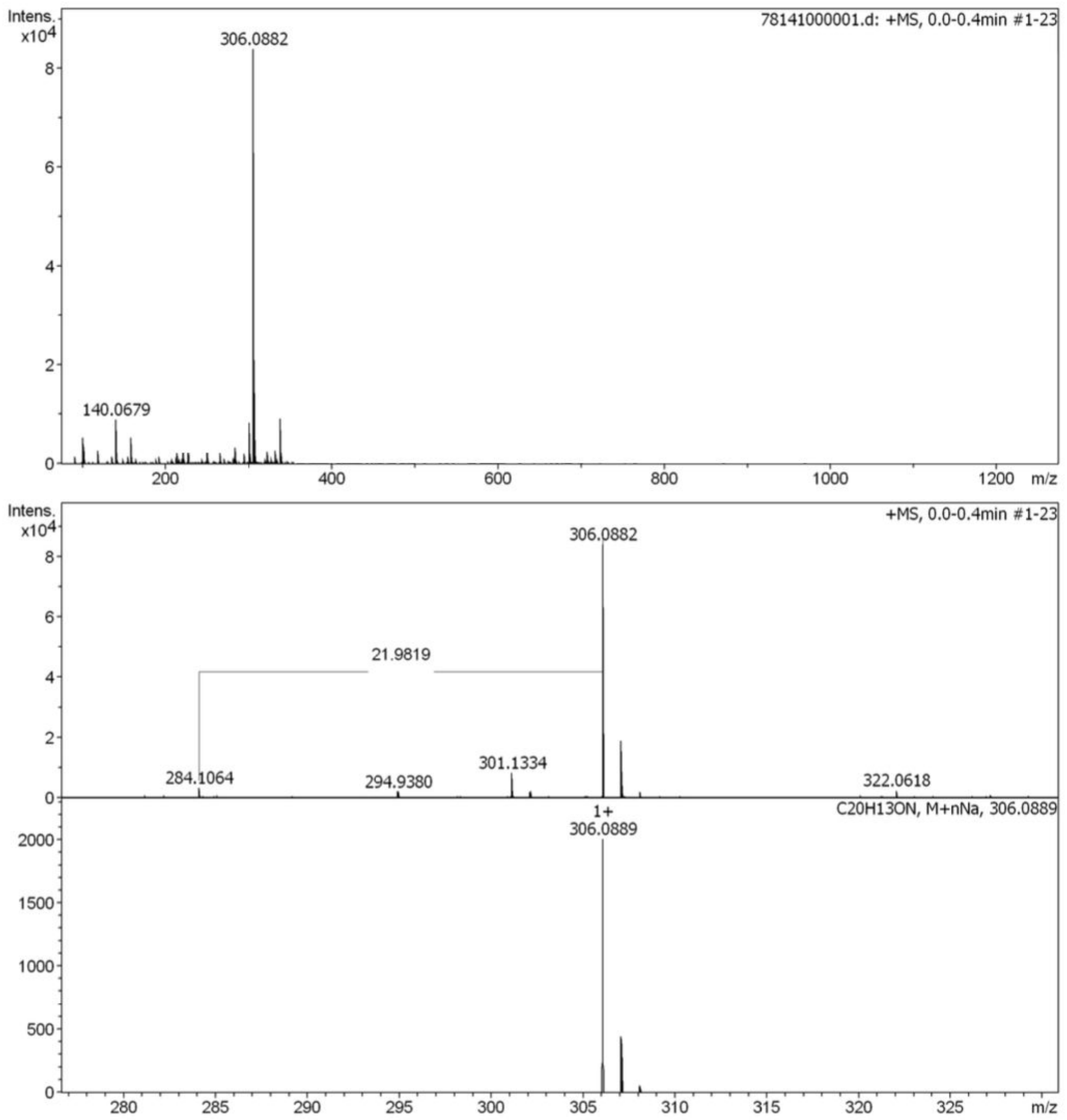

Figure S160. HRMS-ESI mass spectrum of **4m**.

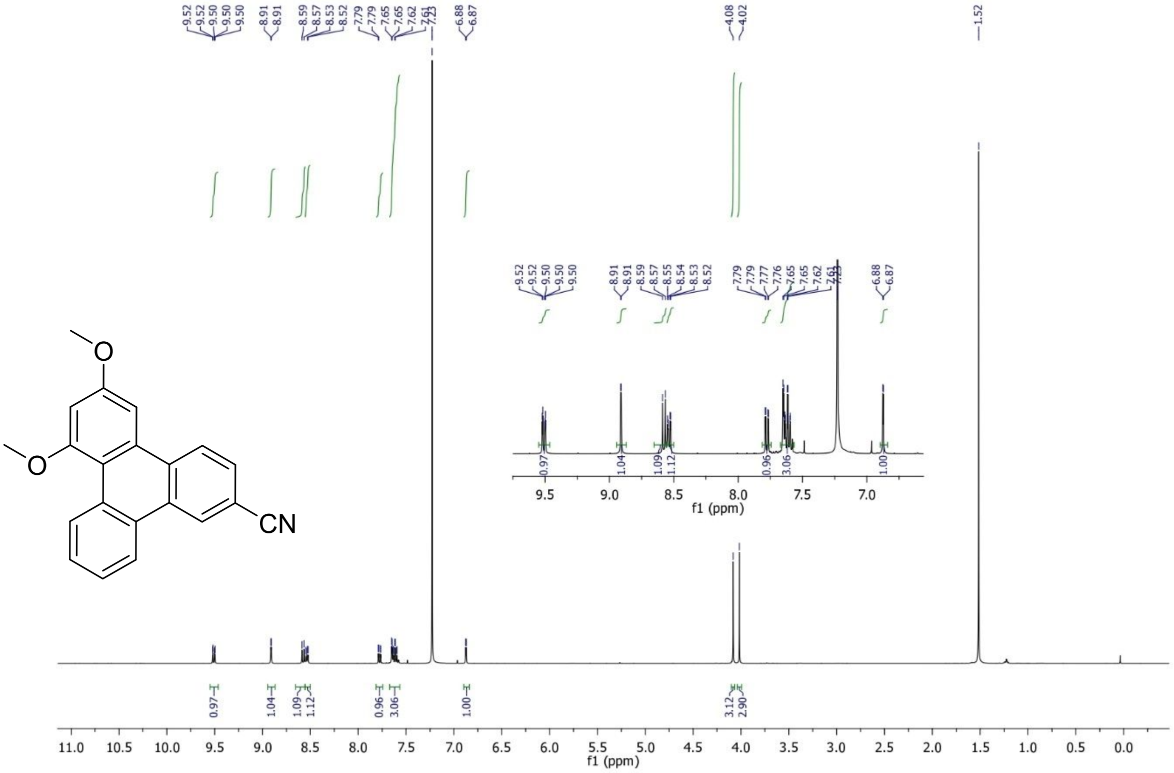

Figure S161. ^{1}H-NMR (600 MHz) spectrum of **4n** in $CDCl_3$.

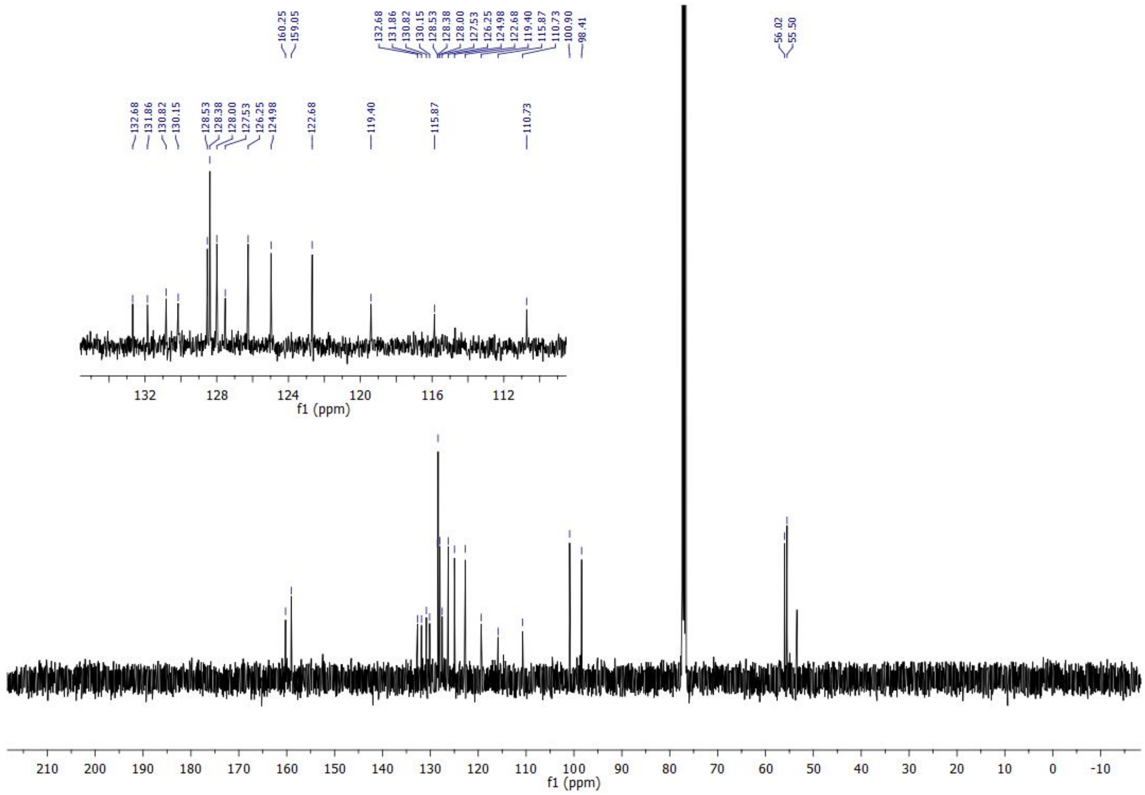

Figure S162. ^{13}C-NMR (151 MHz) spectrum of **4n** in $CDCl_3$.

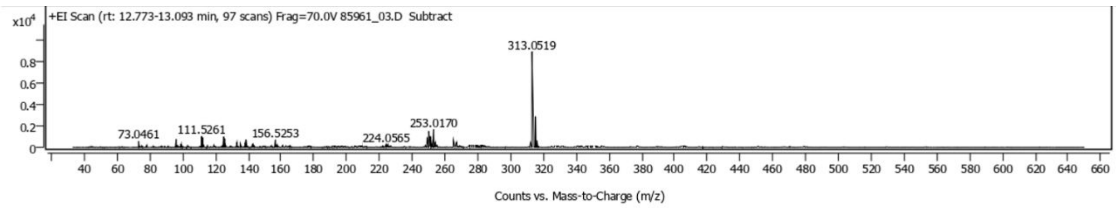

Figure S163. HRGC-MS mass spectrum of **4n**.

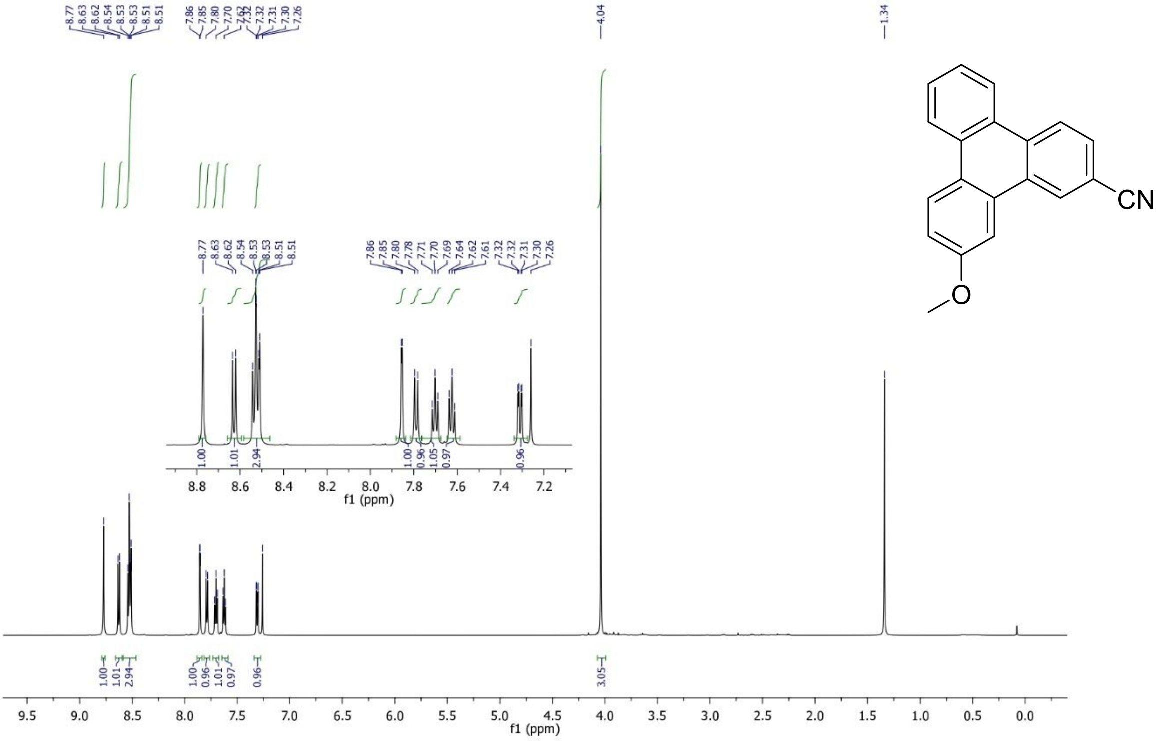

Figure S164. ^{1}H-NMR (600 MHz) spectrum of **4o** in $CDCl_3$.

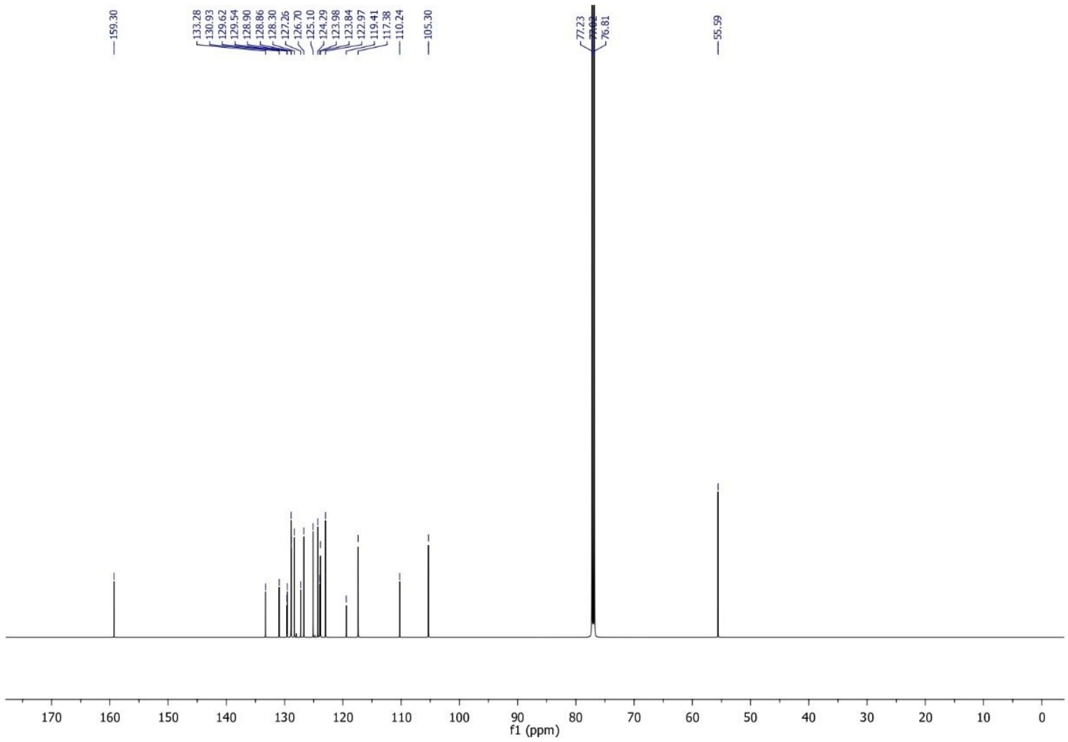

Figure S165. ^{13}C-NMR (151 MHz) spectrum of **4o** in $CDCl_3$.

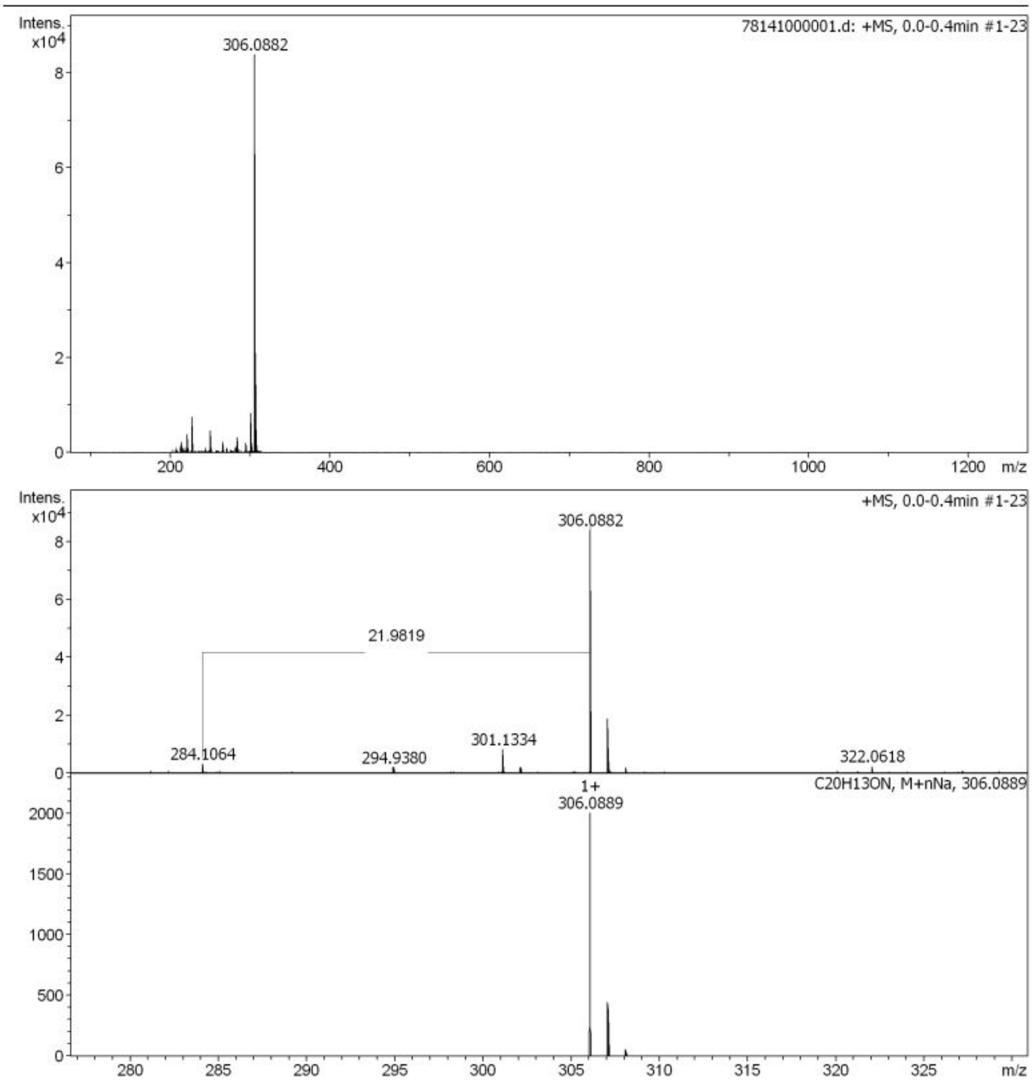

Figure S166. HRMS-ESI mass spectrum of **4o**.

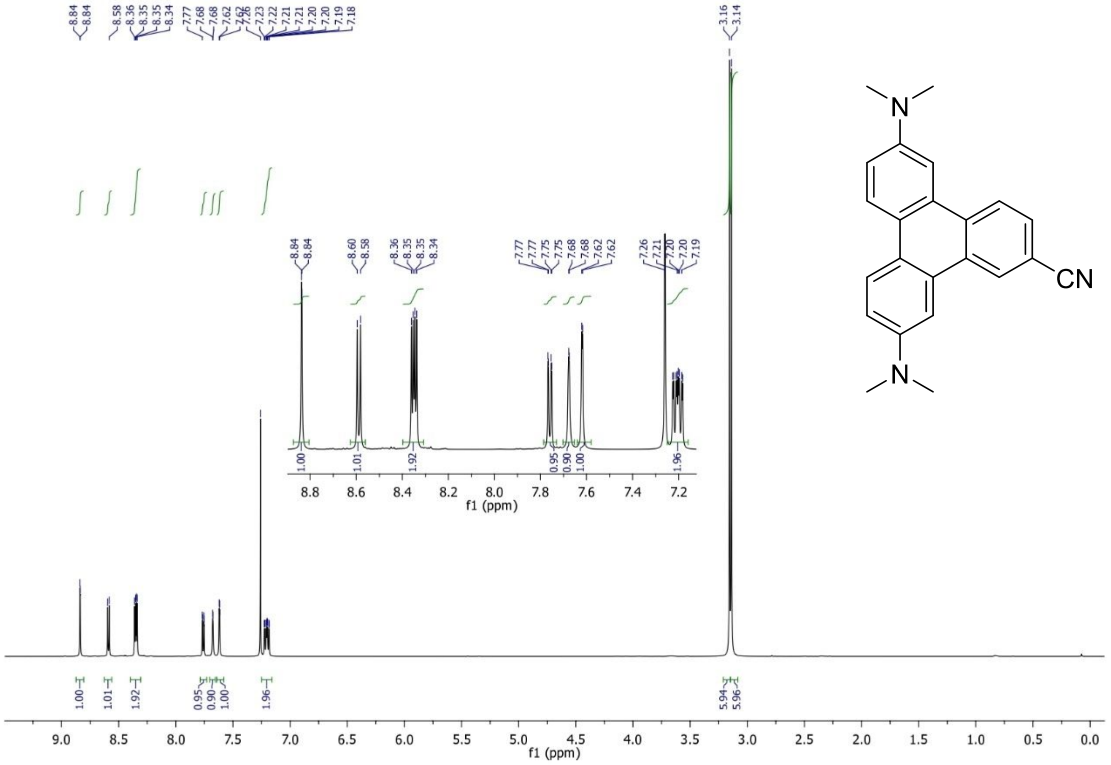

Figure S167. ^{1}H-NMR (600 MHz) spectrum of **4p** in $CDCl_3$.

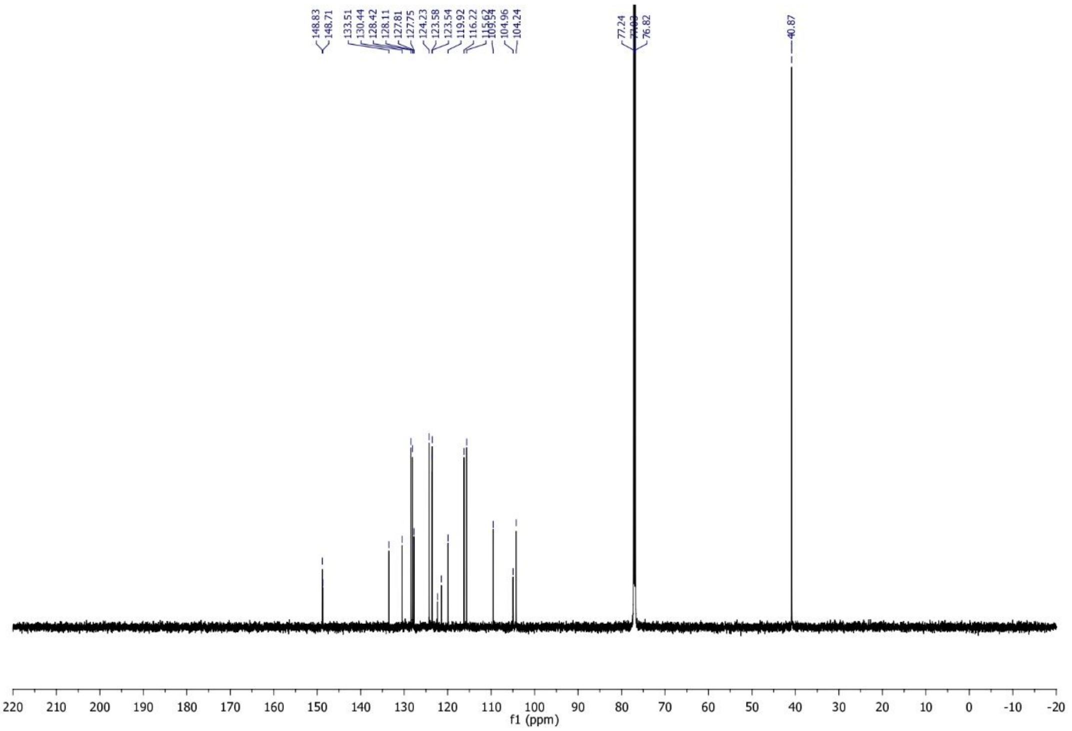

Figure S168. ^{13}C-NMR (151 MHz) spectrum of **4p** in $CDCl_3$.

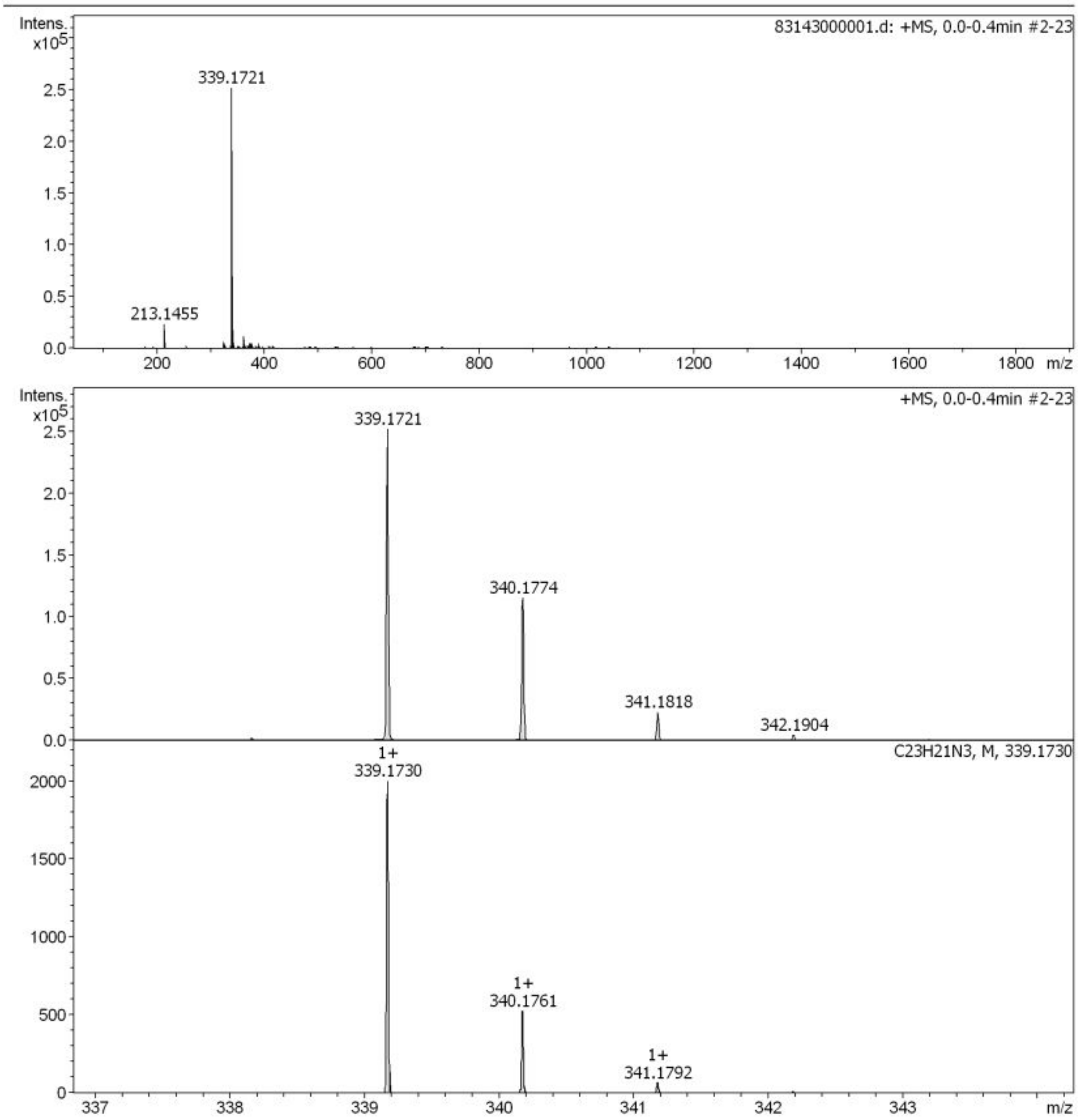

Figure S169. HRMS-ESI mass spectrum of **4p**.

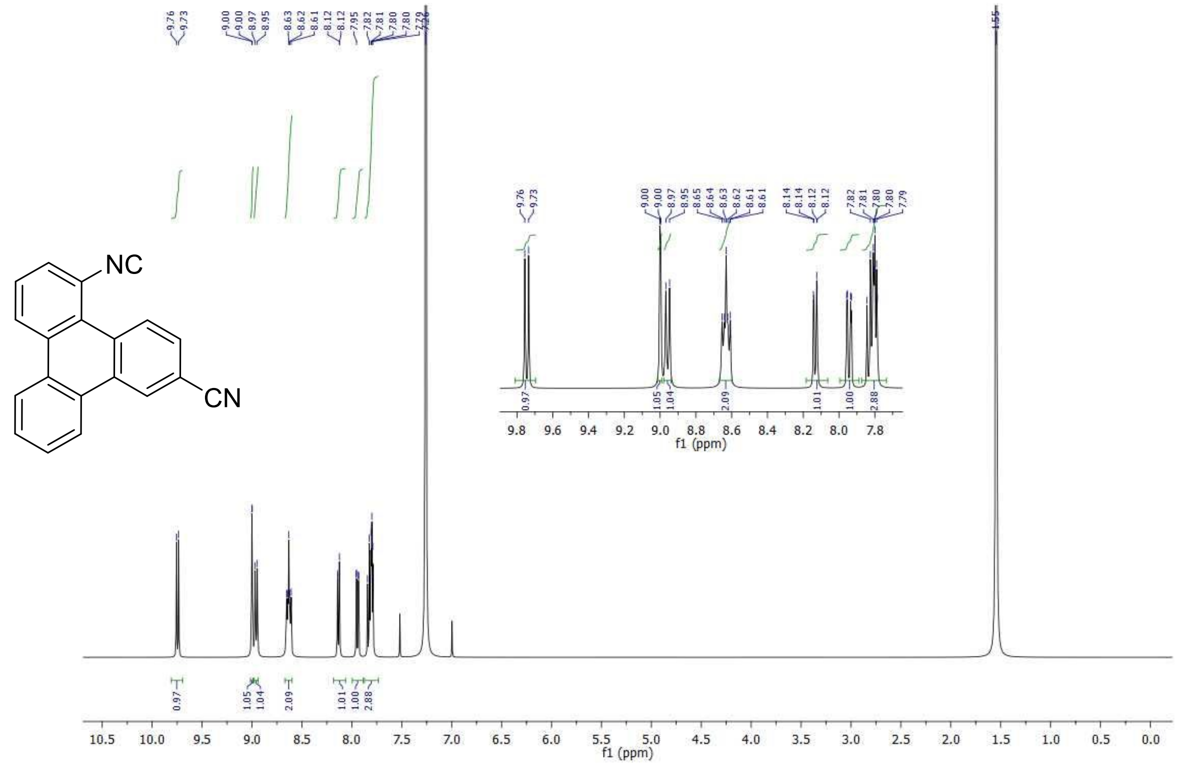

Figure S170. ^{1}H-NMR (600 MHz) spectrum of **4q** in $CDCl_3$.

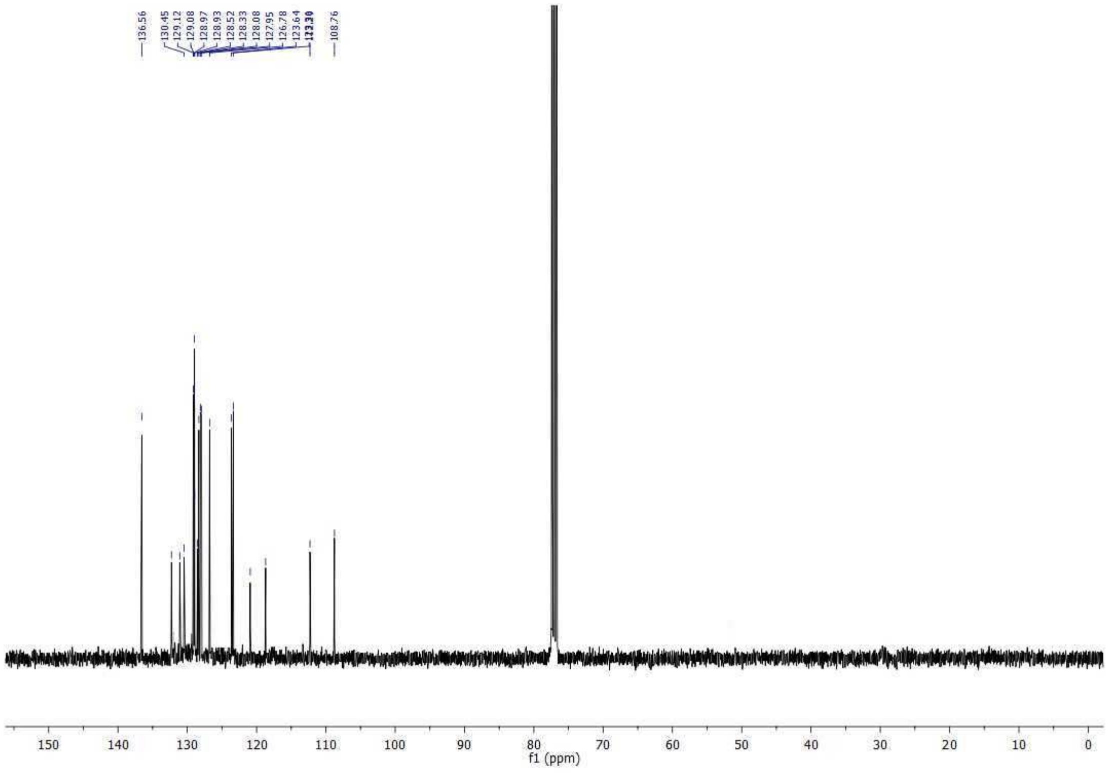

Figure S171. ^{13}C-NMR (151 MHz) spectrum of **4q** in $CDCl_3$.

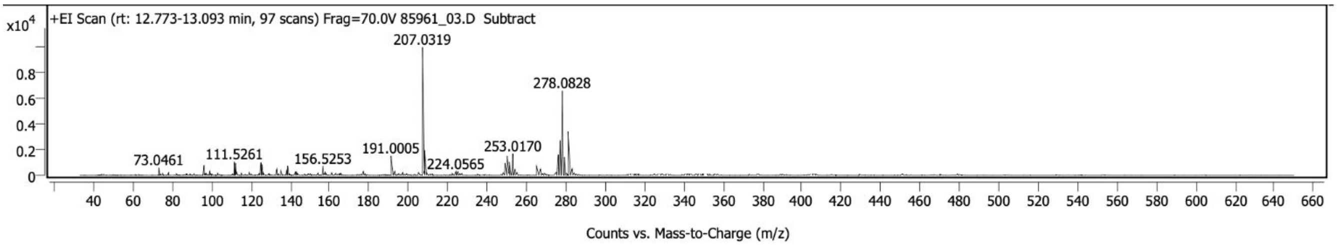

Figure S172. HRGC-MS mass spectrum of **4q.**

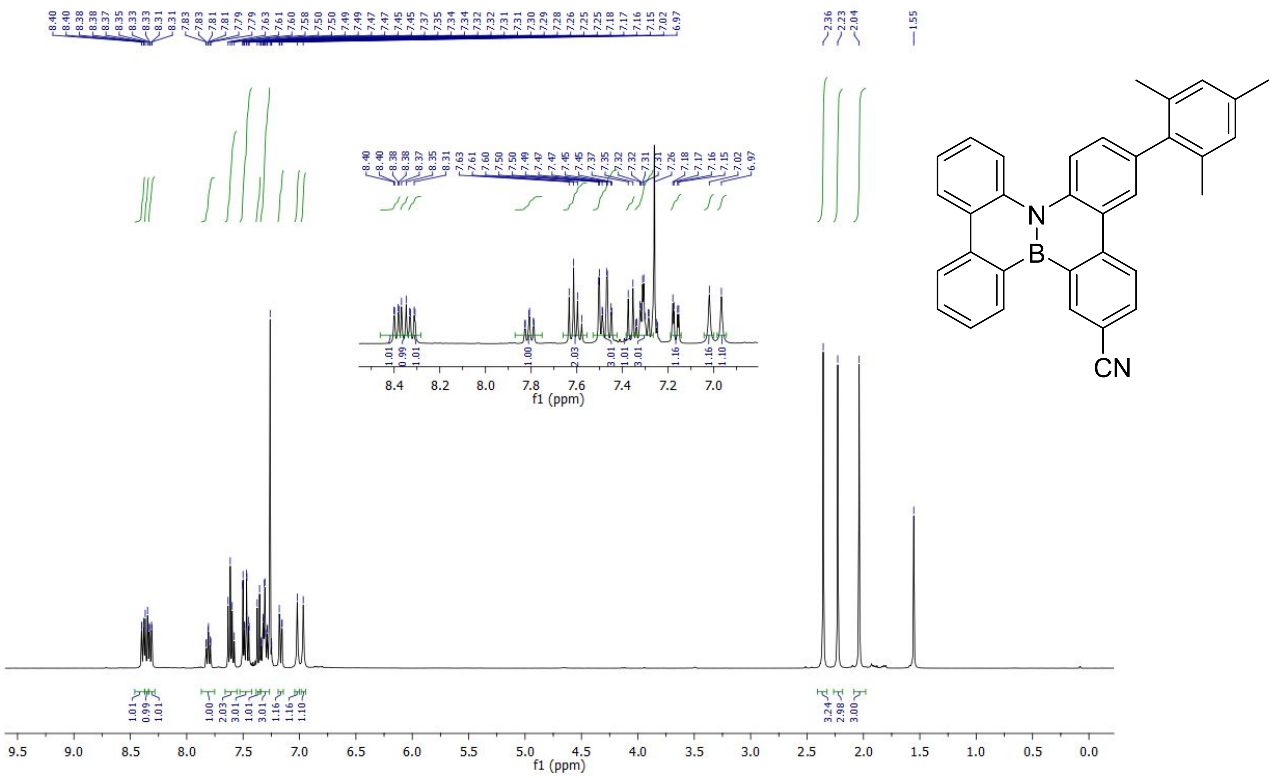

Figure S173. ^{1}H-NMR (600 MHz) spectrum of **4r** in $CDCl_3$.

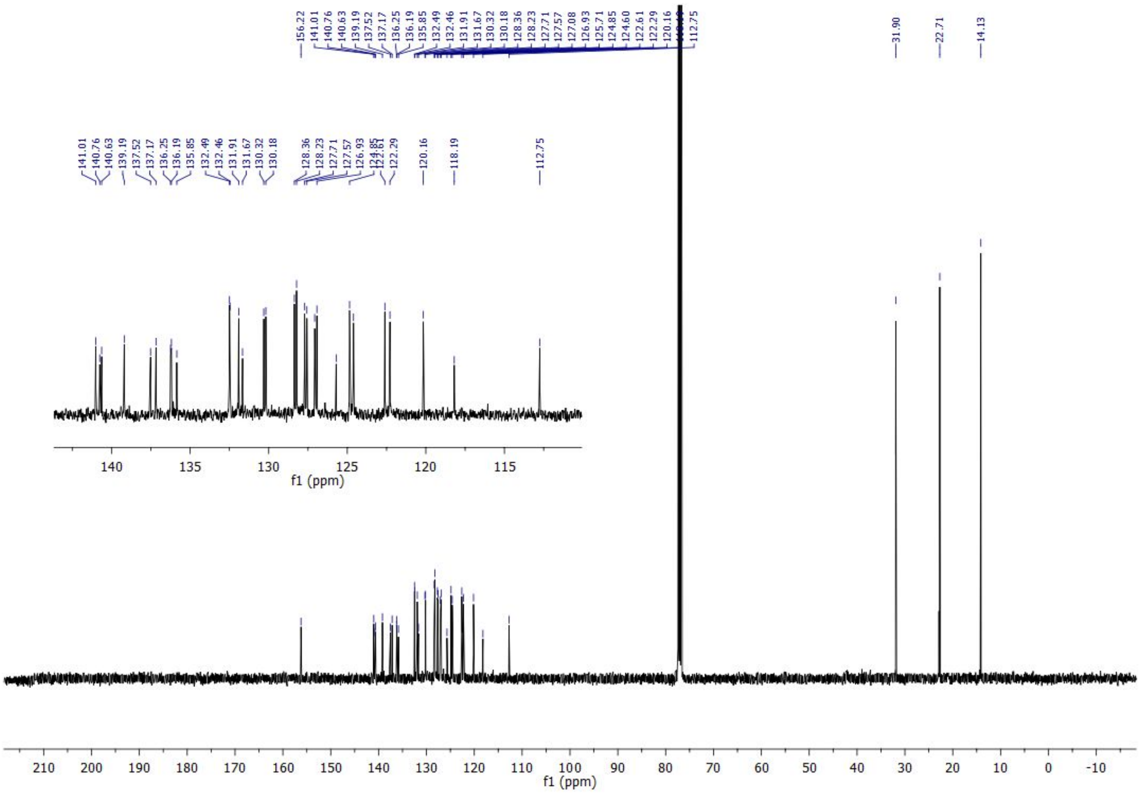

Figure S174. ^{13}C-NMR (151 MHz) spectrum of **4r** in $CDCl_3$.

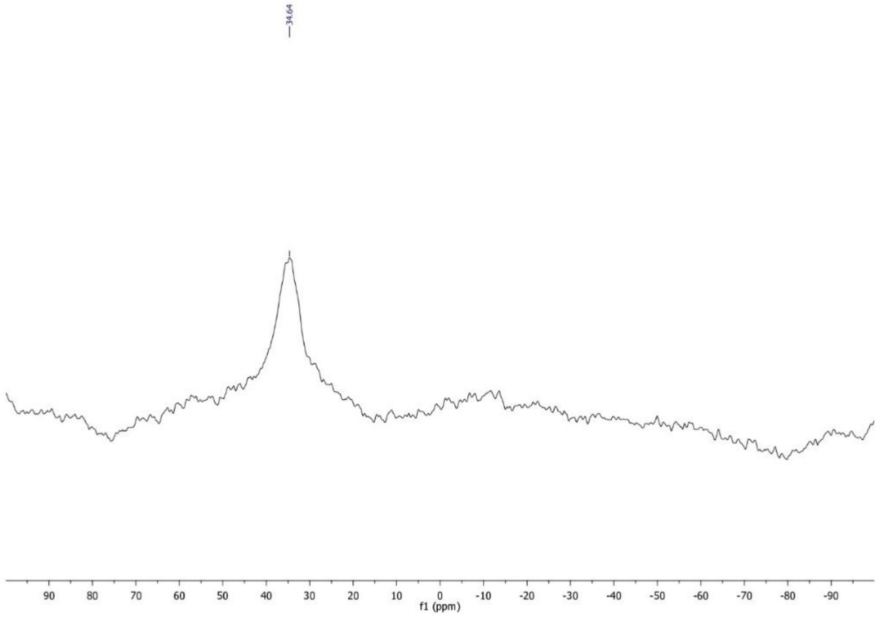

Figure S175. ^{11}B-NMR (193 MHz) spectrum of **4r** in $CDCl_3$.

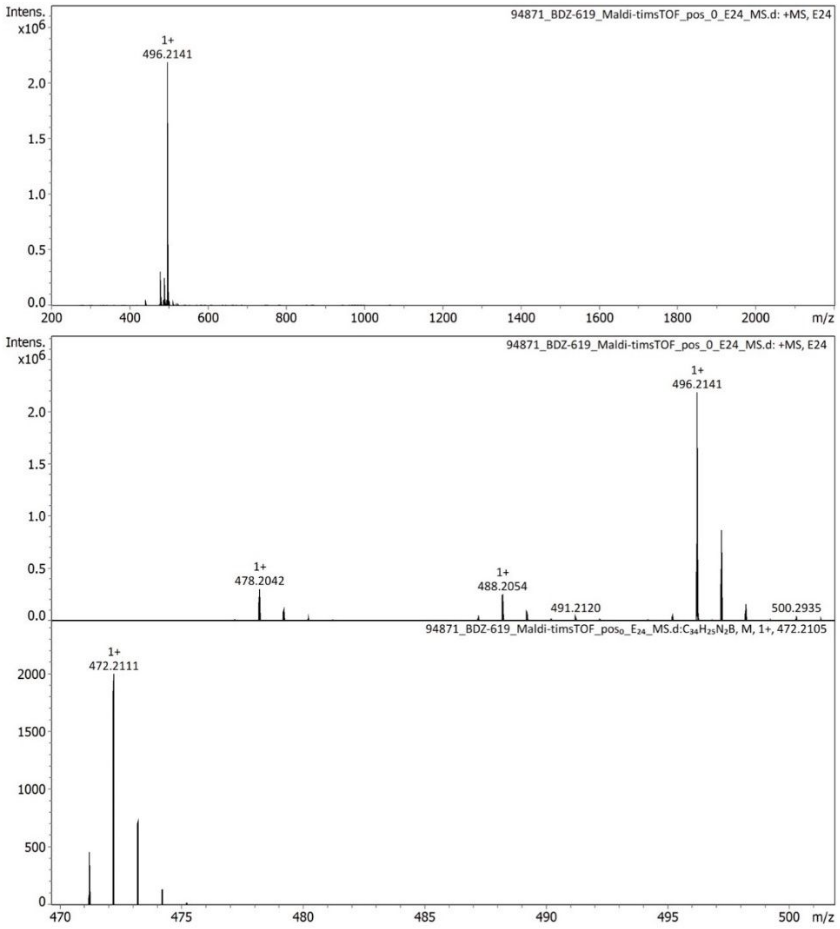

Figure S176. HRMS-LD-TOF mass spectrum of **4r.**

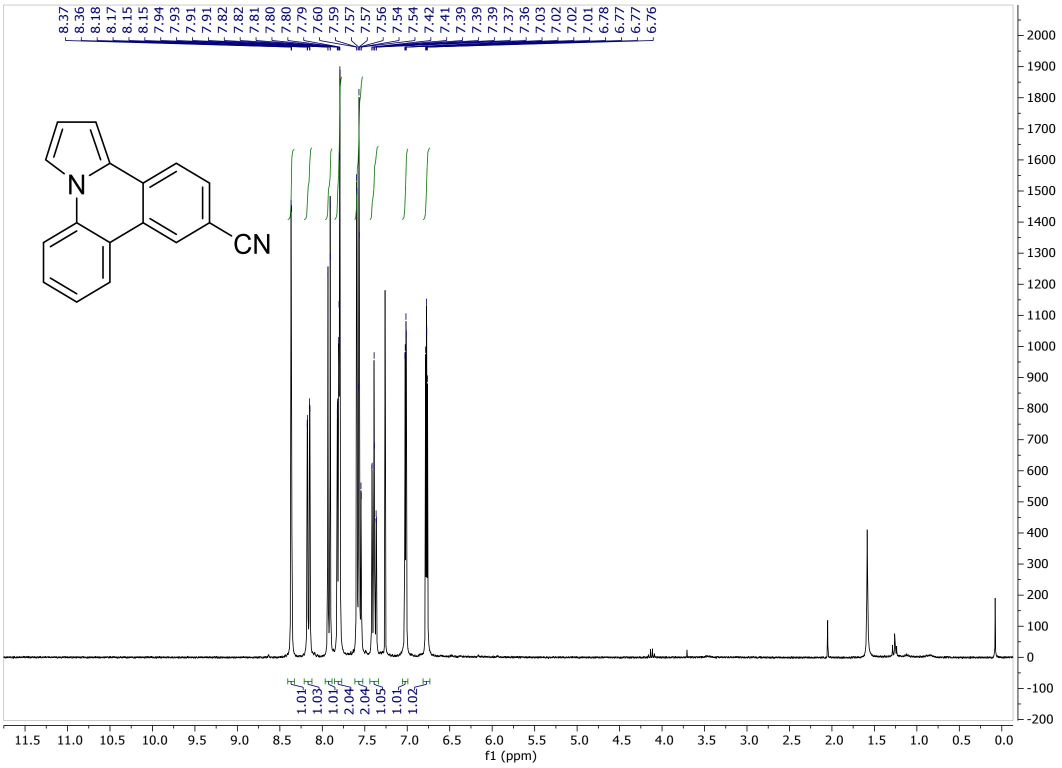

Figure S177. ^{1}H-NMR (300 MHz) spectrum of **6a** in $CDCl_3$.

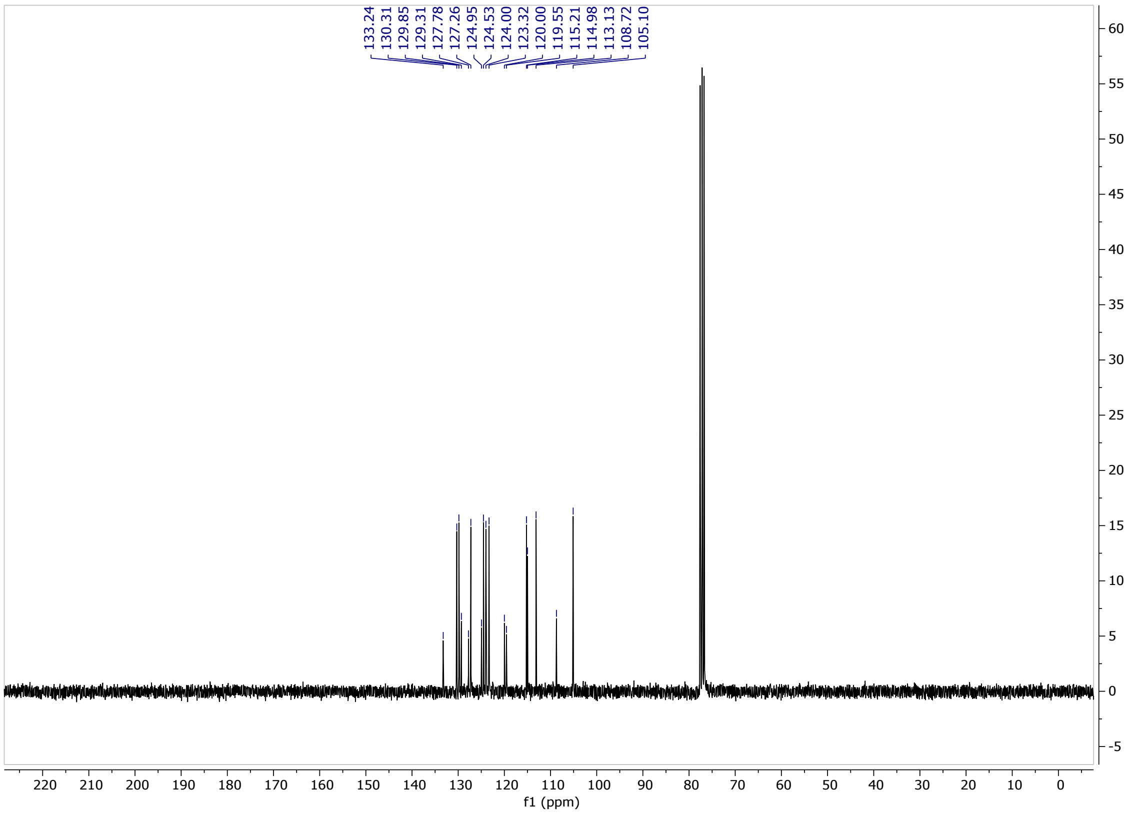

Figure S178. ^{13}C-NMR (75 MHz) spectrum of **6a** in $CDCl_3$.

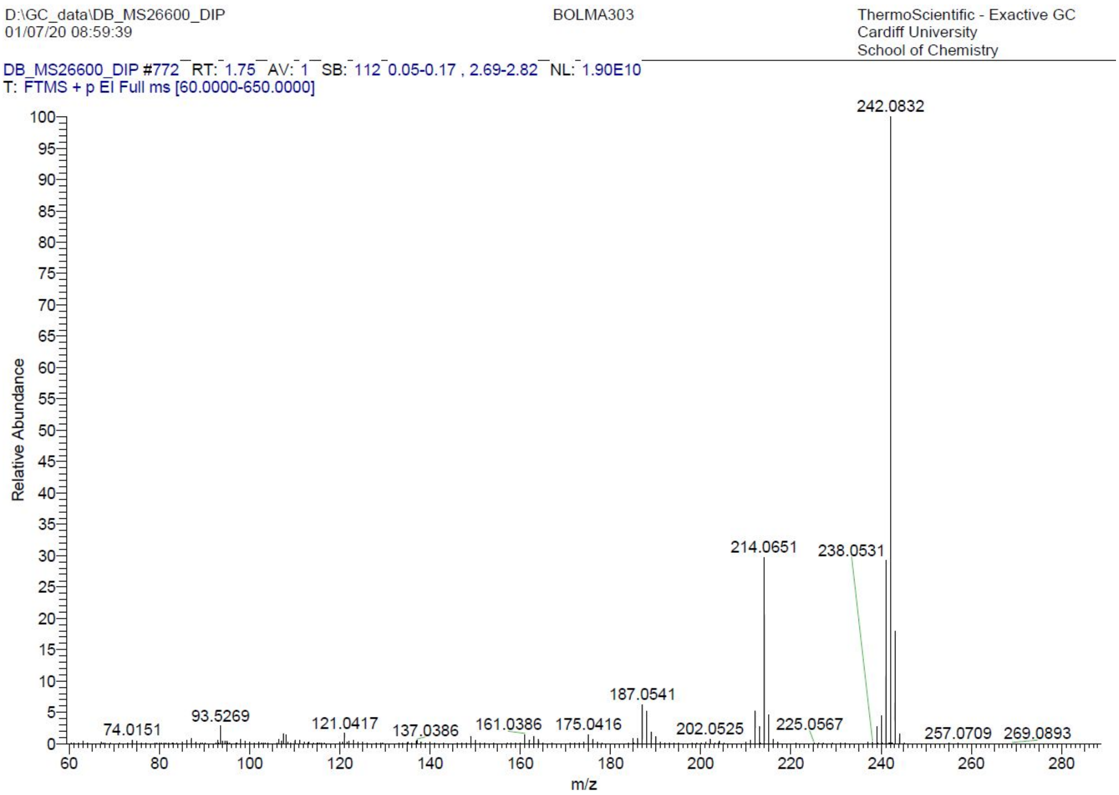

Figure S179. HRMS-ESI-TOF mass spectrum of **6a**.

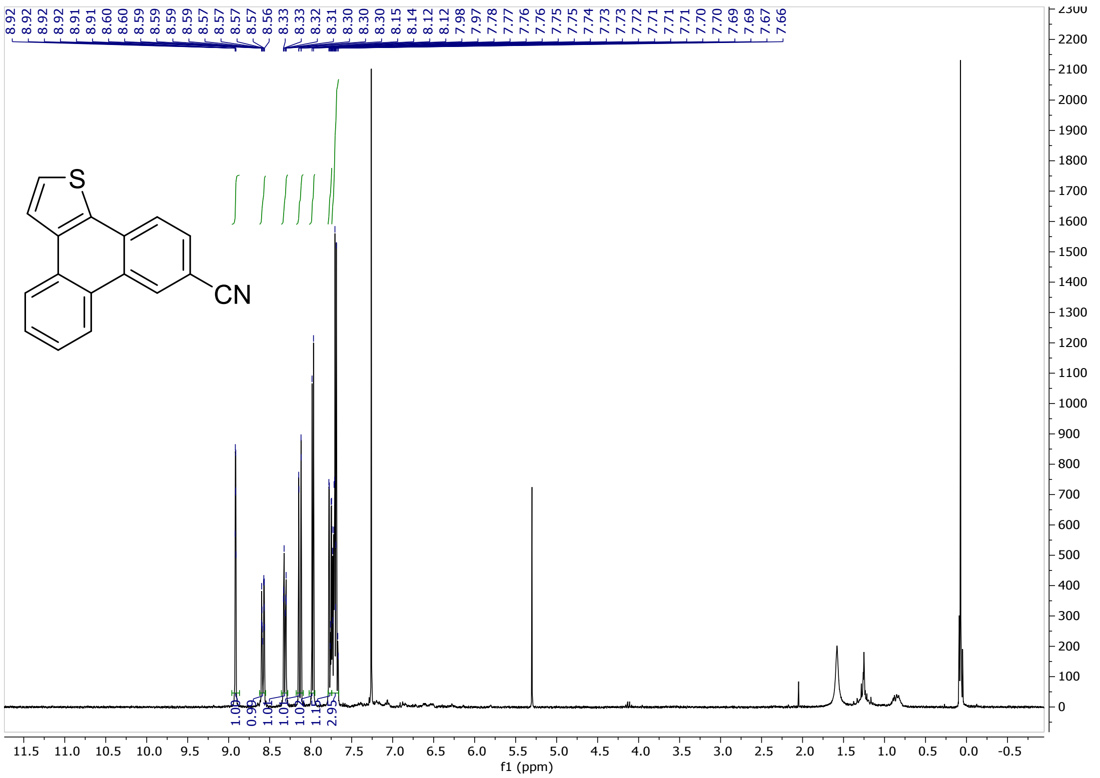

Figure S180. ^{1}H-NMR (300 MHz) spectrum of **6b** in $CDCl_3$.

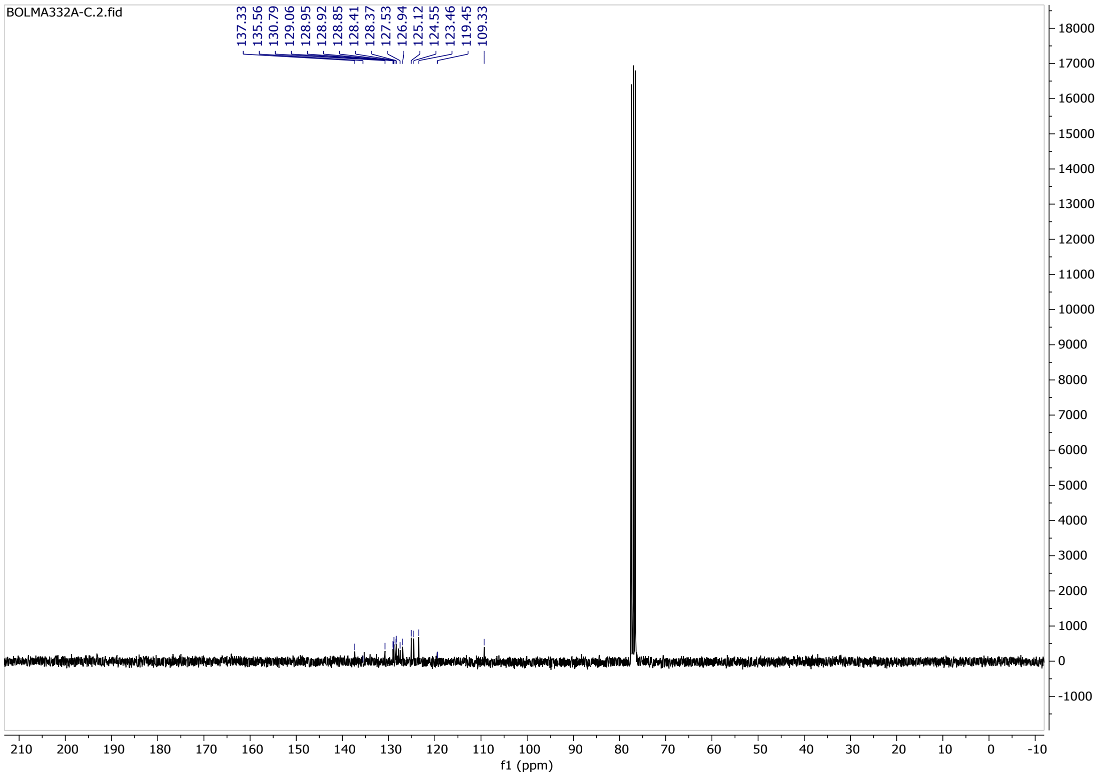

Figure S181. ^{13}C-NMR (75 MHz) spectrum of **6b** in $CDCl_3$.

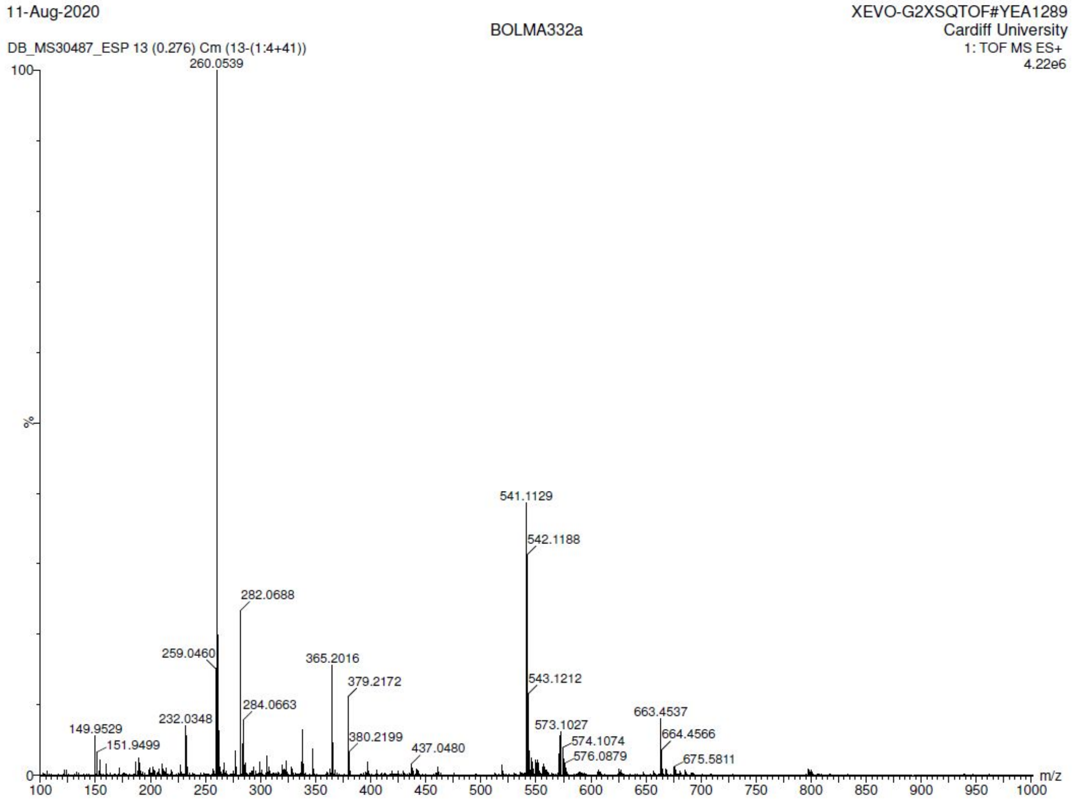

Figure S182. HRMS-ESI-TOF mass spectrum of **6b**.

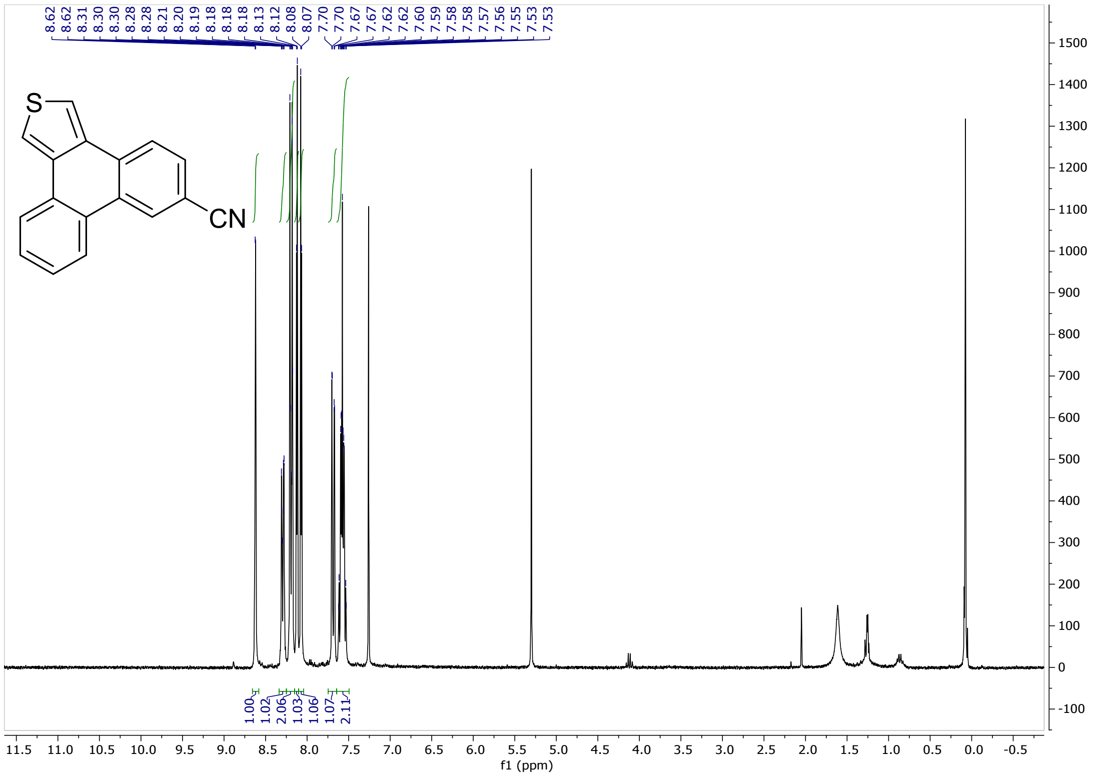

Figure S183. ^{1}H-NMR (300 MHz) spectrum of **6b´** in $CDCl_3$.

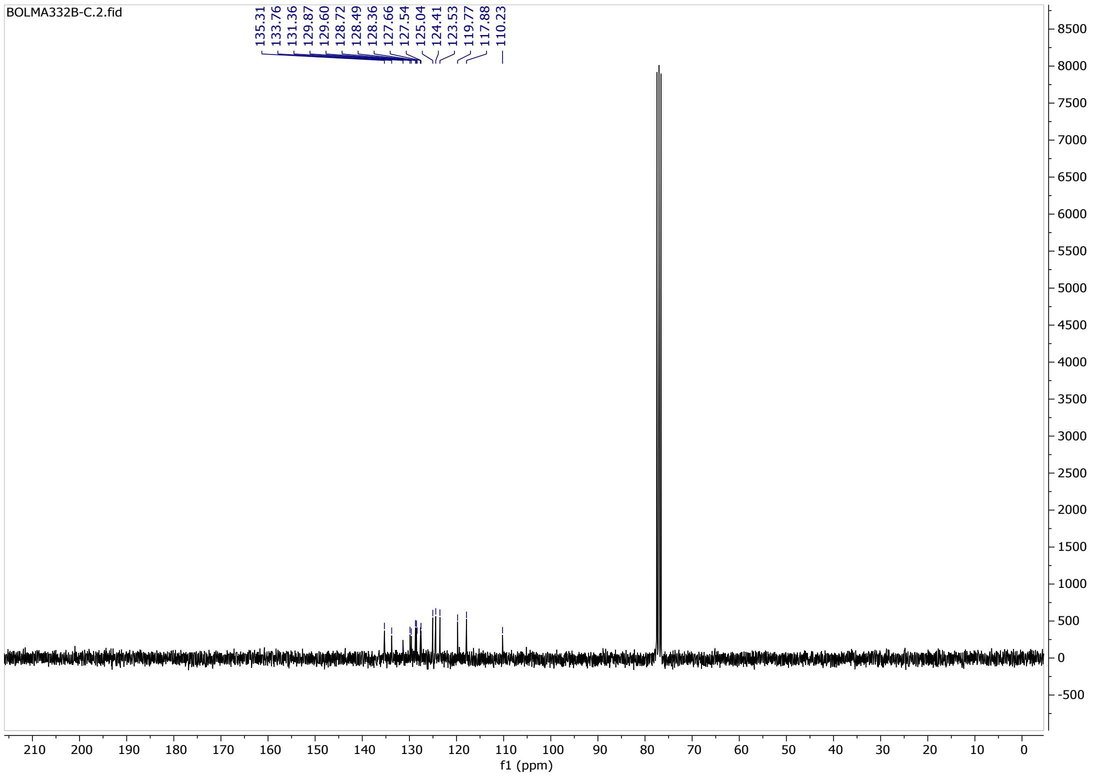

Figure S184. ^{13}C-NMR (75 MHz) spectrum of **6b´** in $CDCl_3$.

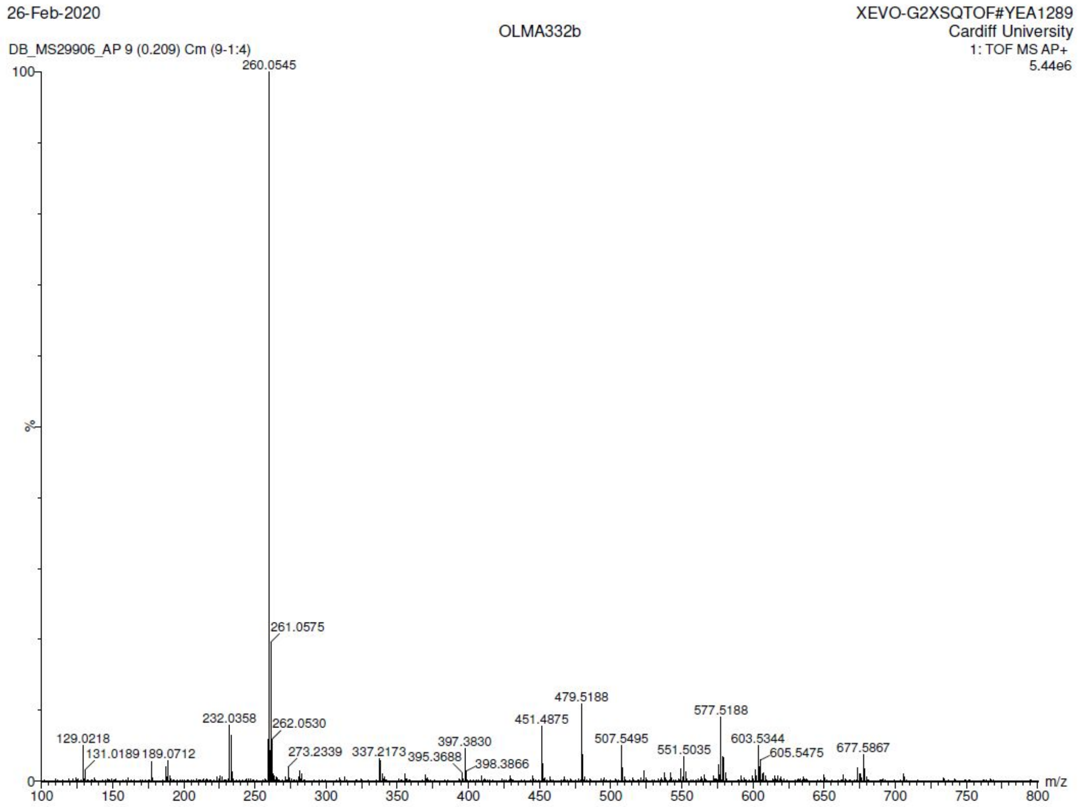

Figure S185. HRMS-APCI TOF mass spectrum of **6b´**.

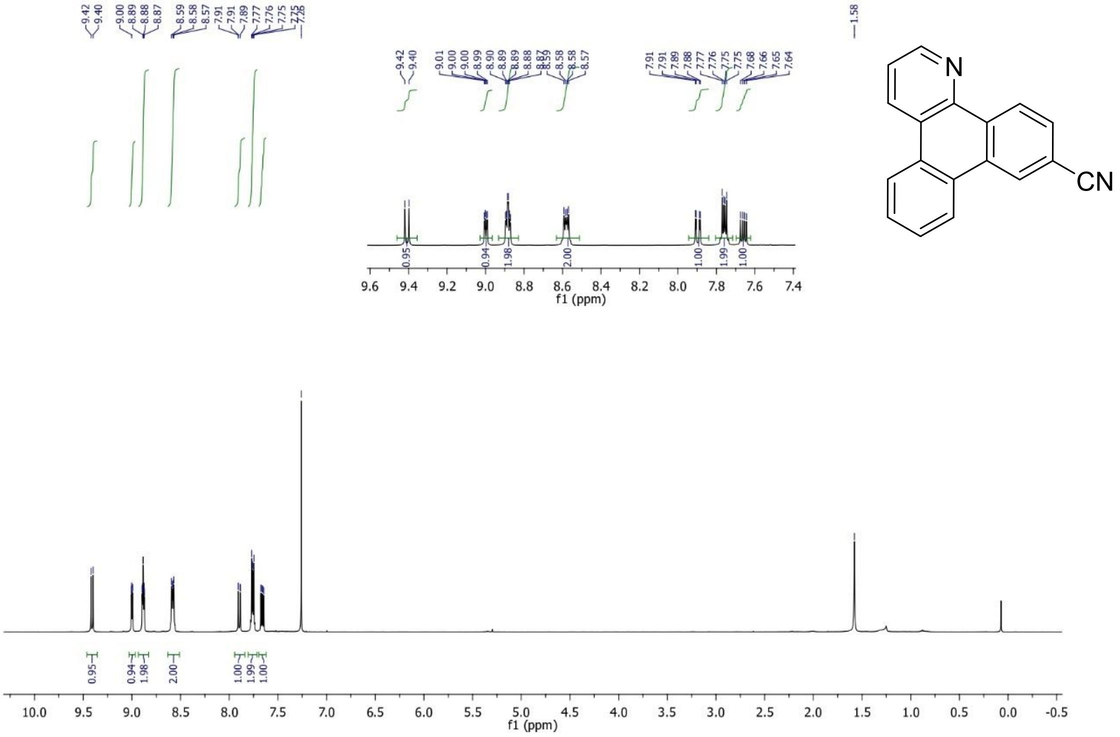

Figure S186. ^{1}H-NMR (600 MHz) spectrum of **6c** in $CDCl_3$.

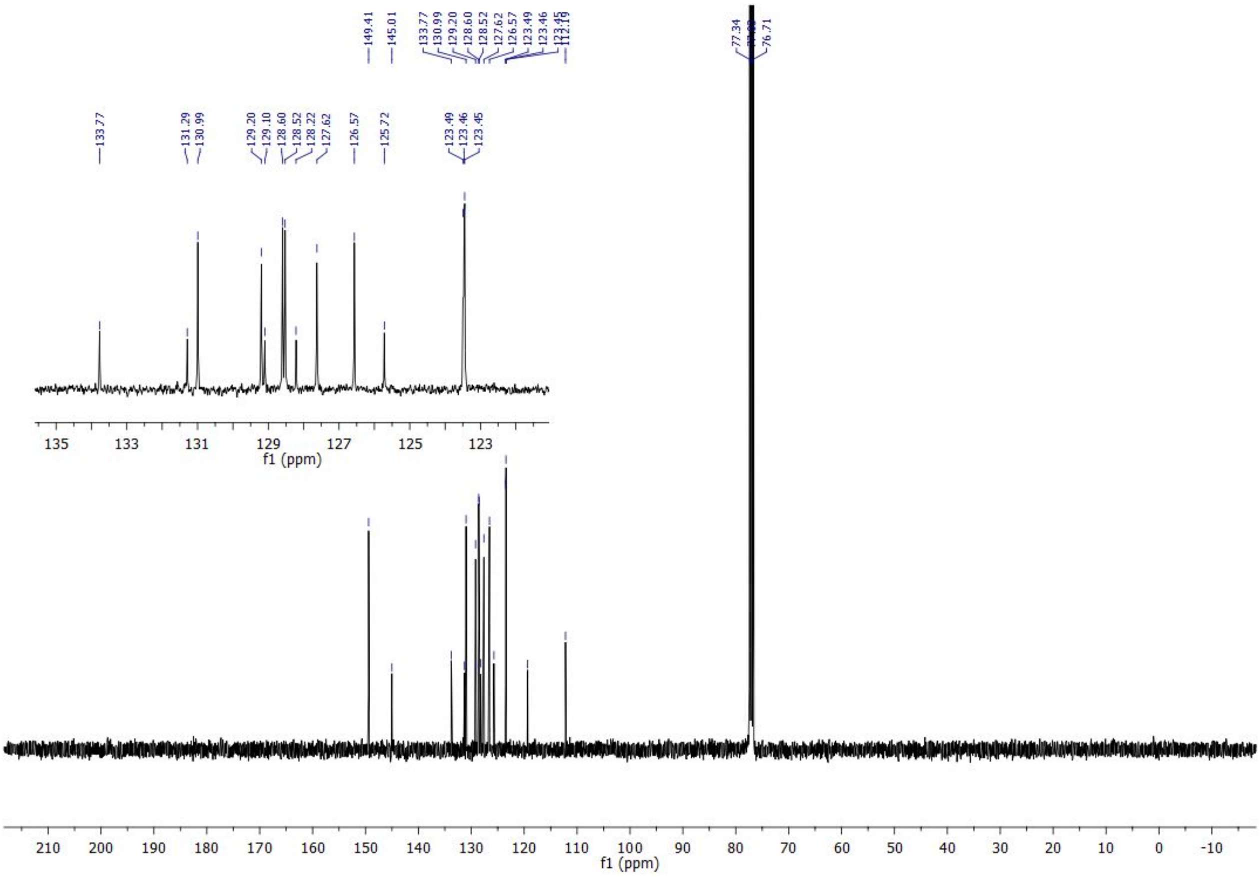

Figure S187. ^{13}C-NMR (151 MHz) spectrum of **6c** in $CDCl_3$.

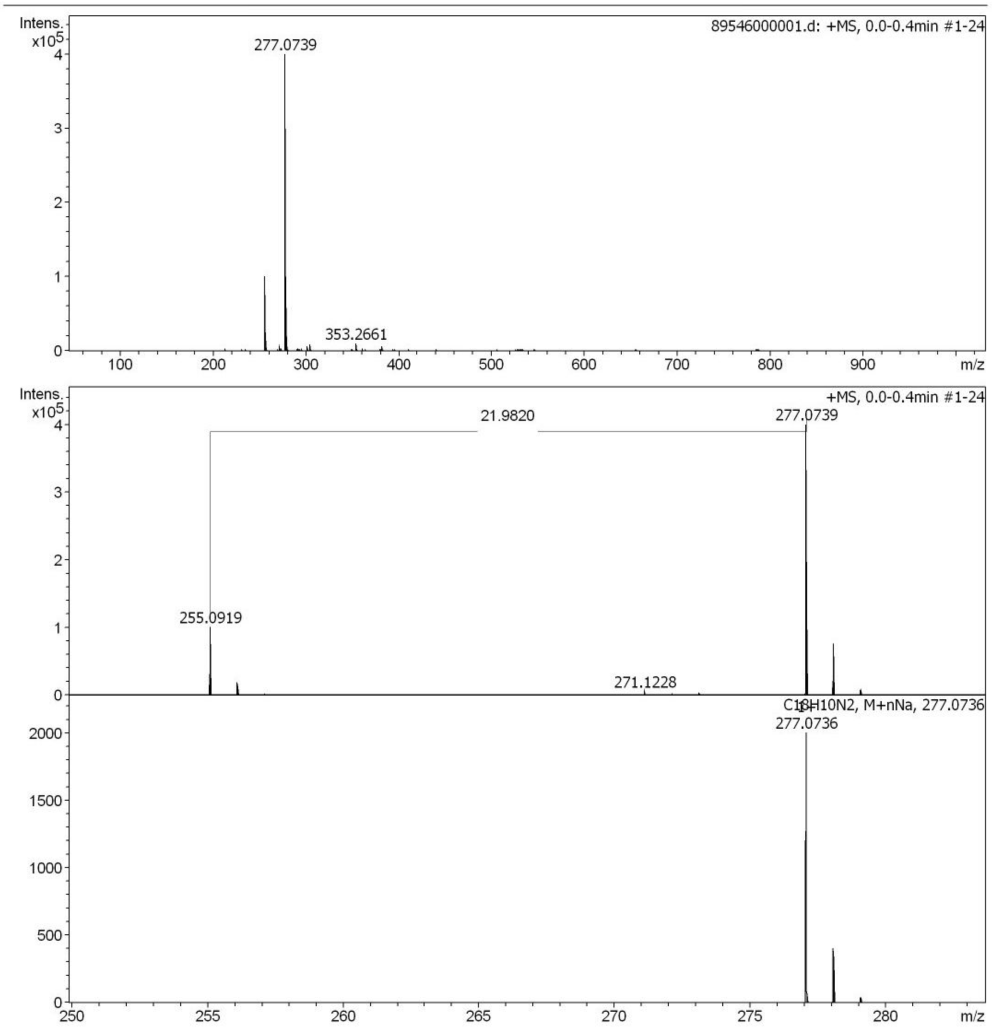

Figure S188. HRMS-ESI mass spectrum of **6c.**

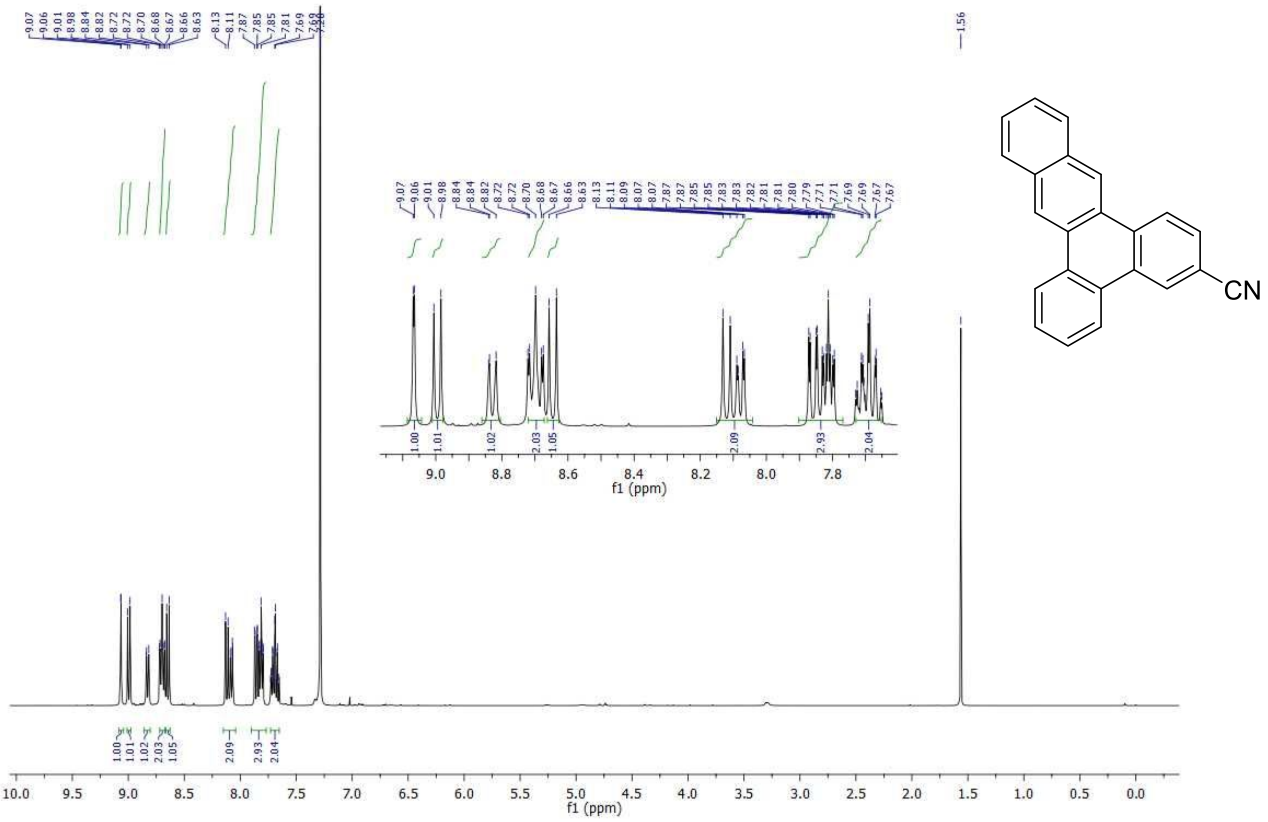

Figure S189. ^{1}H-NMR (600 MHz) spectrum of **8a** in $CDCl_3$.

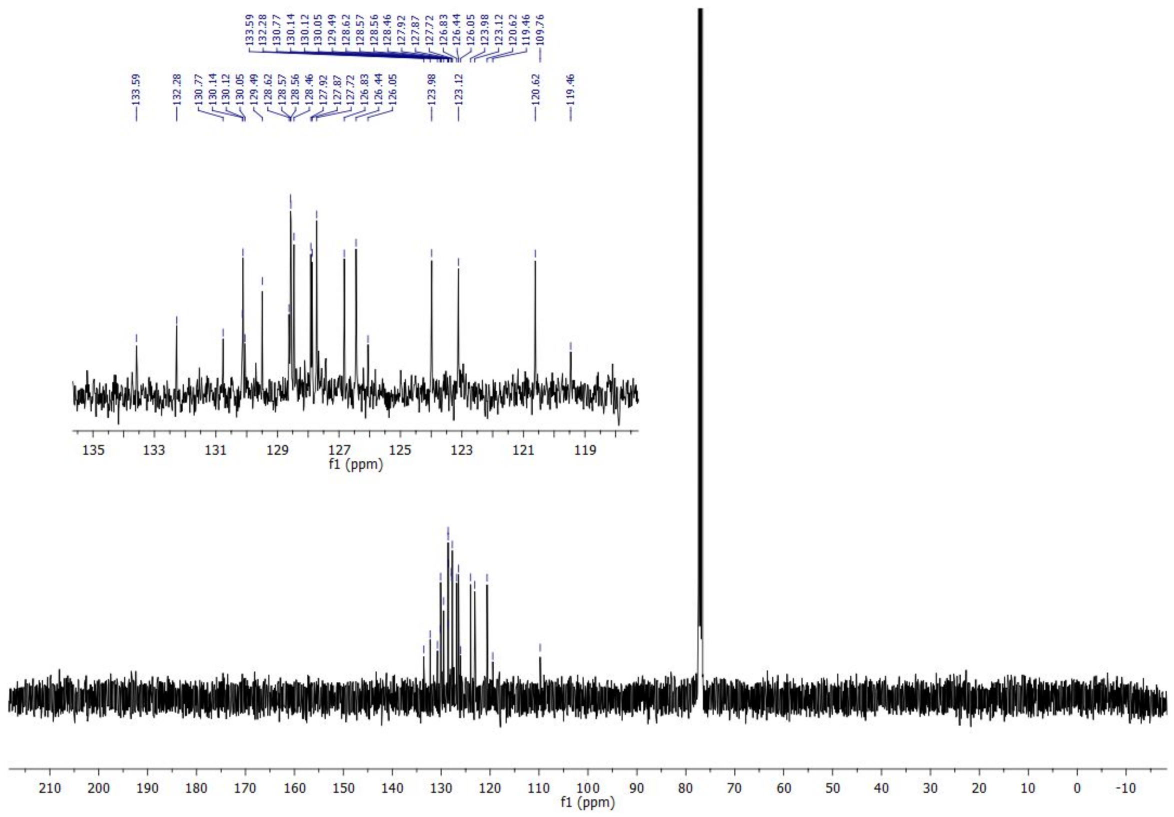

Figure S190. ^{13}C-NMR (151 MHz) spectrum of **8a** in $CDCl_3$.

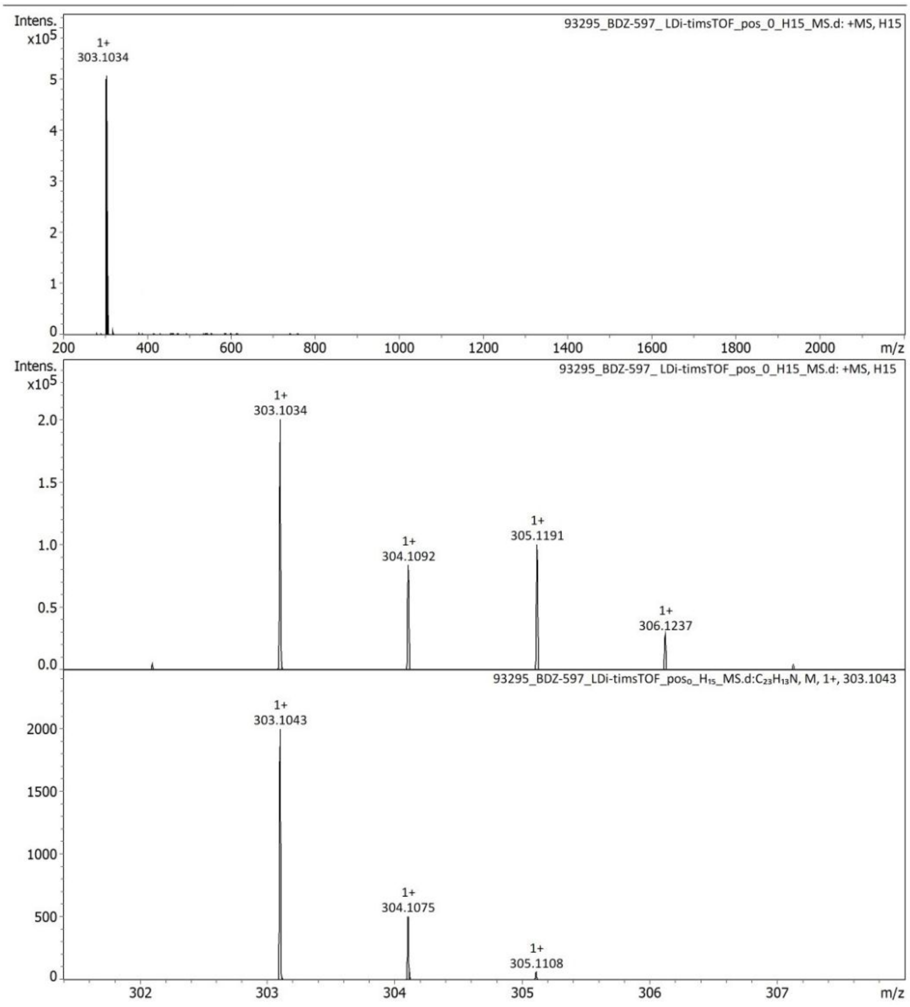

Figure S191. HRMS-LD-TOF mass spectrum of **8a.**

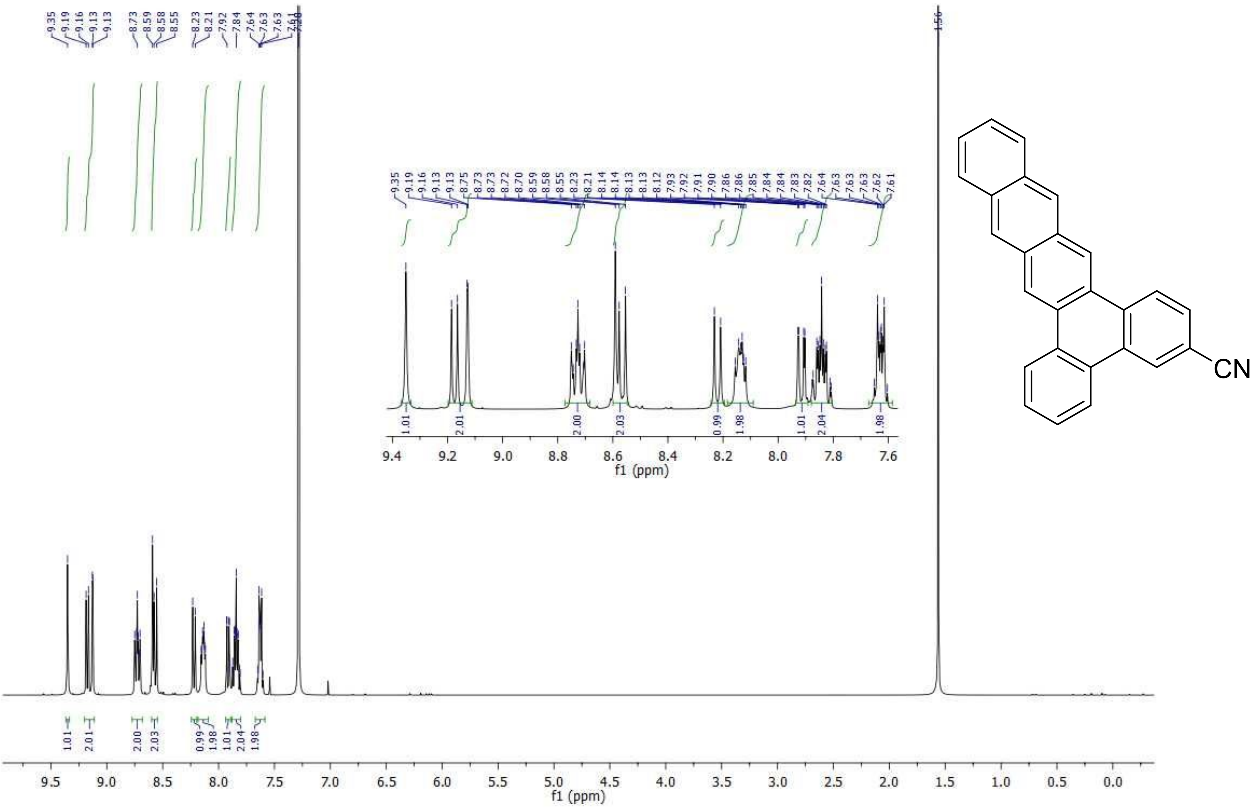

Figure S192. ^{1}H-NMR (600 MHz) spectrum of **8b** in $CDCl_3$.

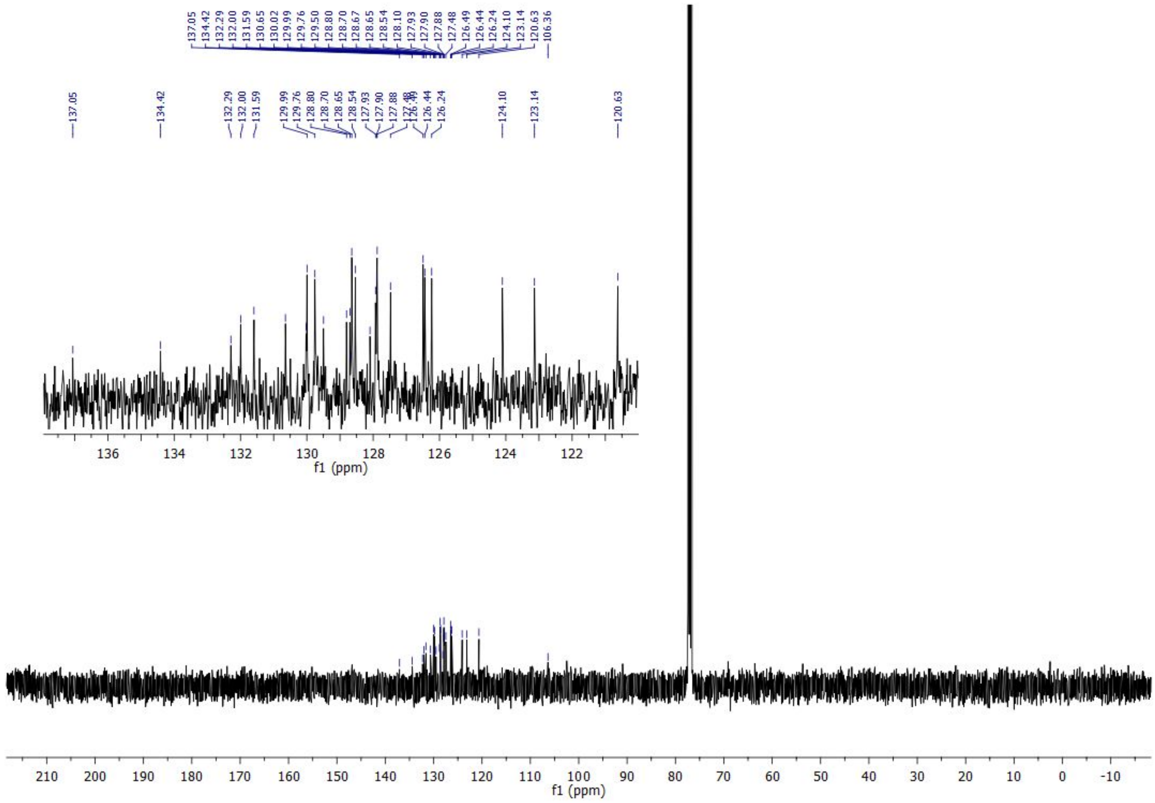

Figure S193. ^{13}C-NMR (151 MHz) spectrum of **8b** in $CDCl_3$.

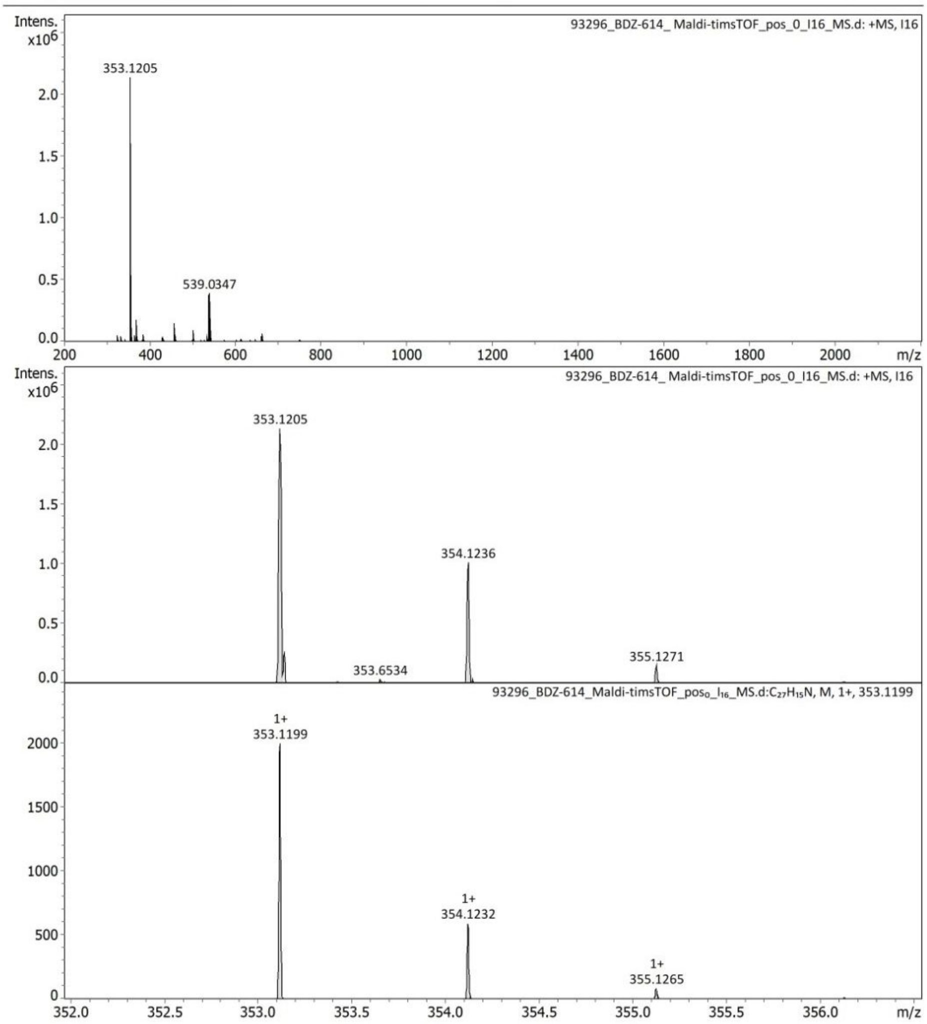

Figure S194. HRMS-LD-TOF mass spectrum of **8b.**

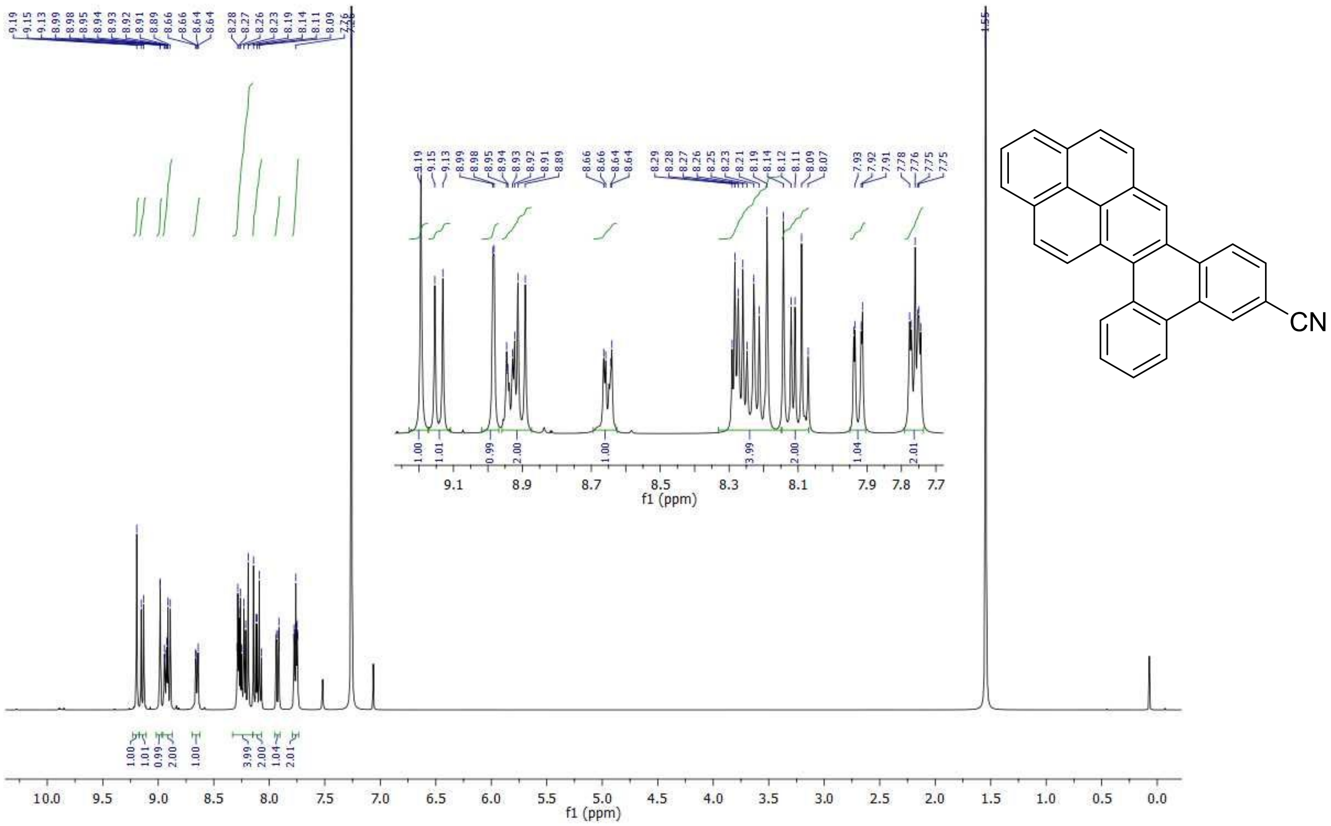

Figure S195. ^{1}H-NMR (600 MHz) spectrum of **8c** in $CDCl_3$.

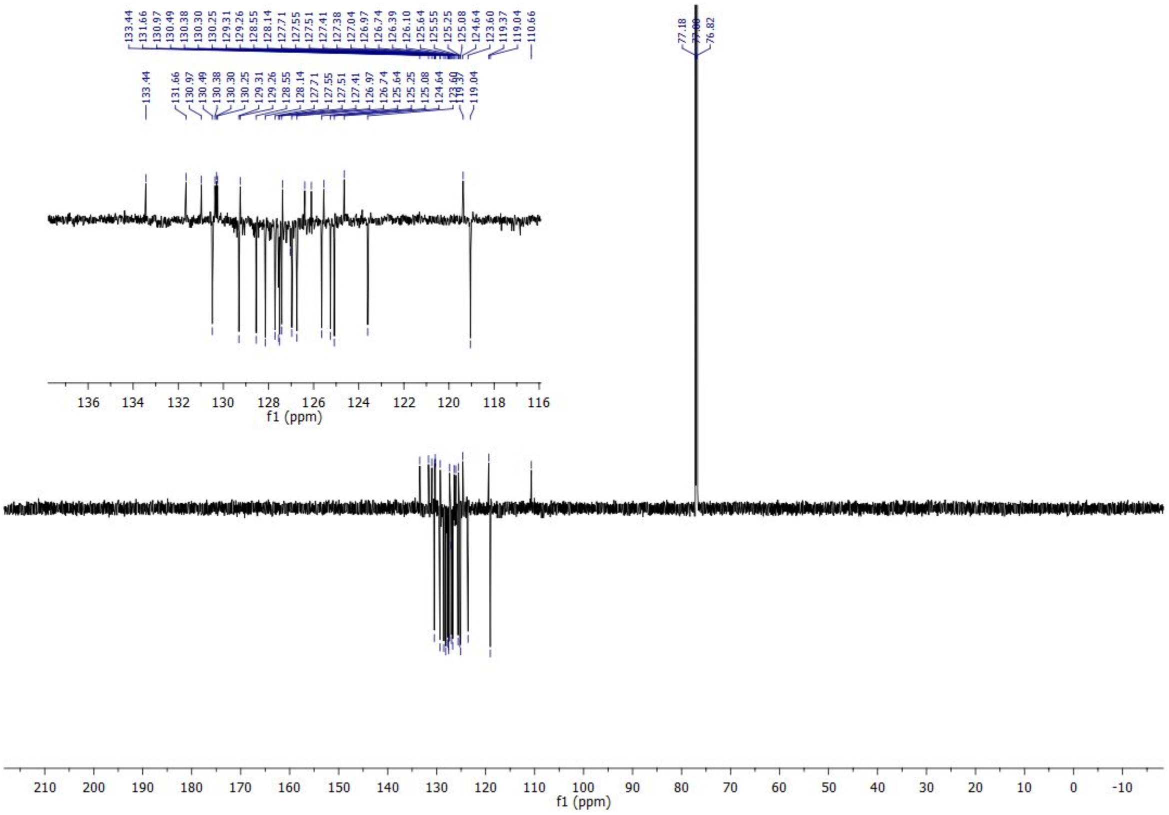

Figure S196. ^{13}C-NMR (151 MHz) spectrum of **8c** in $CDCl_3$.

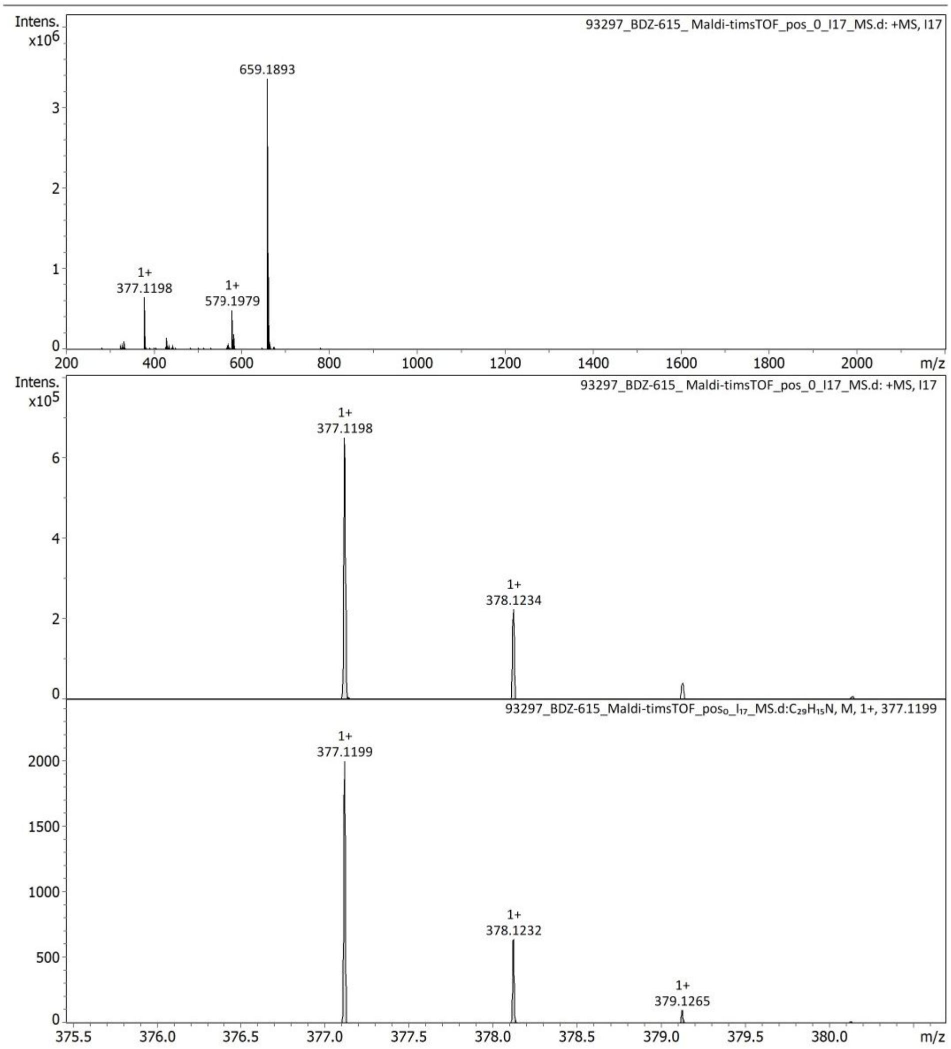

Figure S197. HRMS-Maldi-TOF mass spectrum of **8c.**

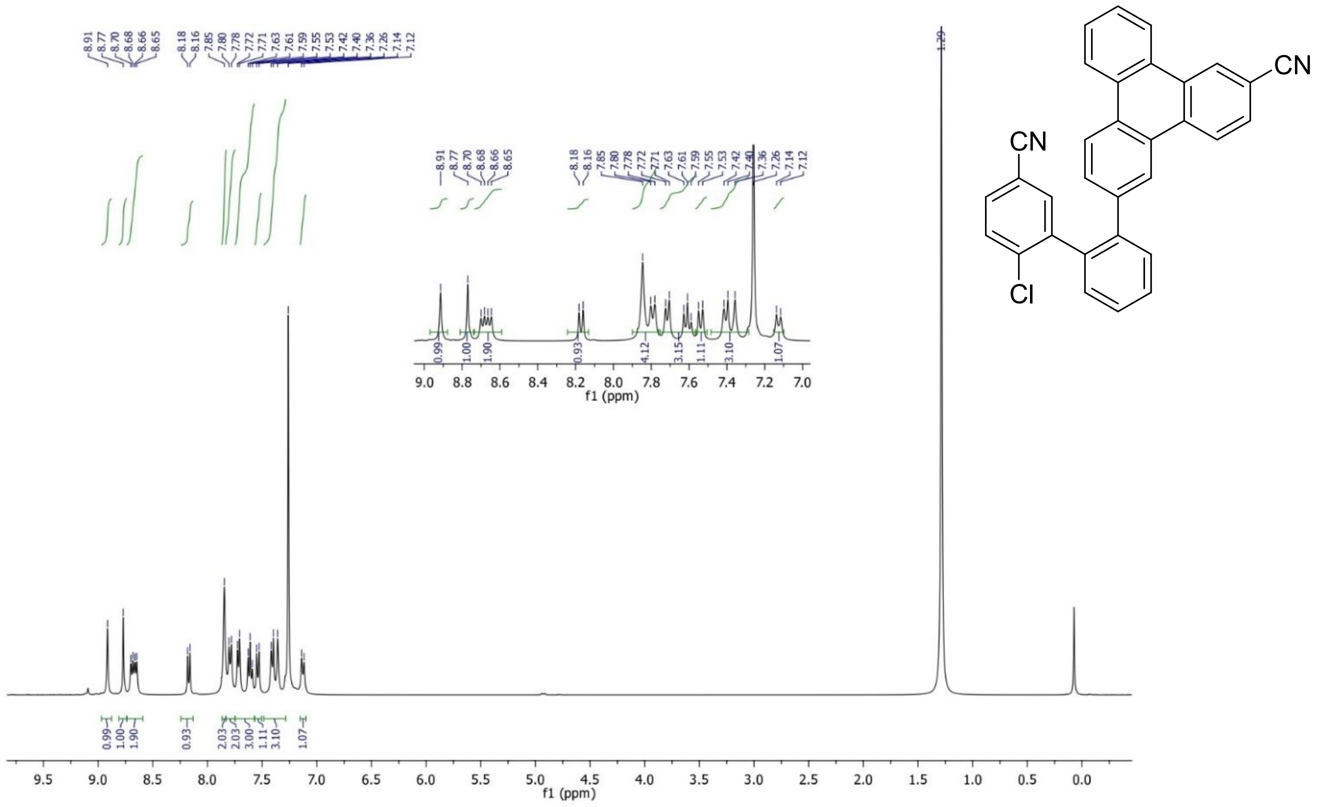

Figure S198. ^{1}H-NMR (600 MHz) spectrum of **11a** in $CDCl_3$.

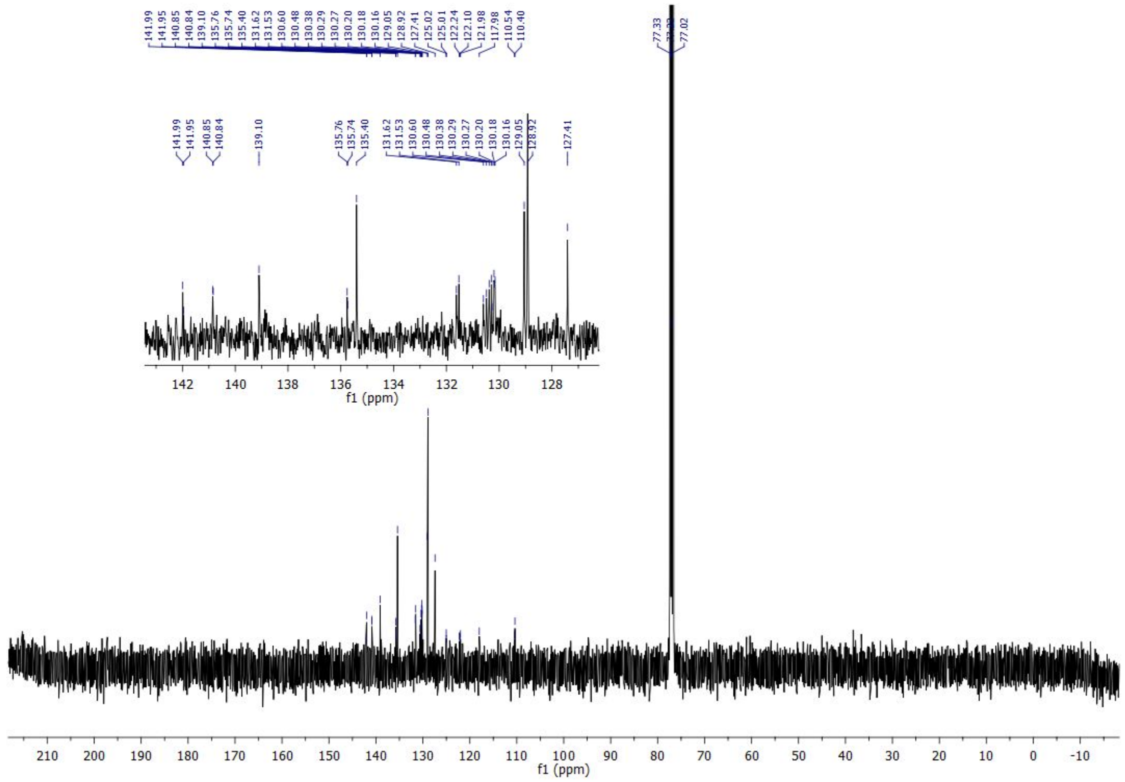

Figure S199. ^{13}C-NMR (151 MHz) spectrum of **11a** in $CDCl_3$.

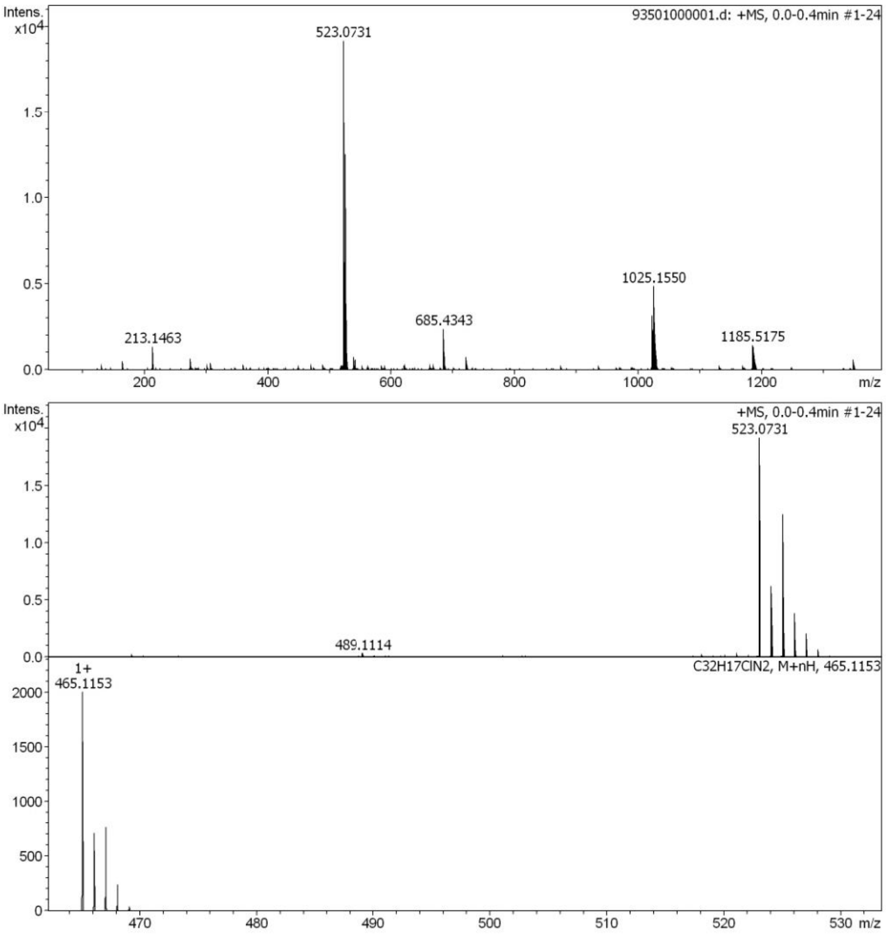

Figure S200. HRMS-ESI mass spectrum of **11a.**

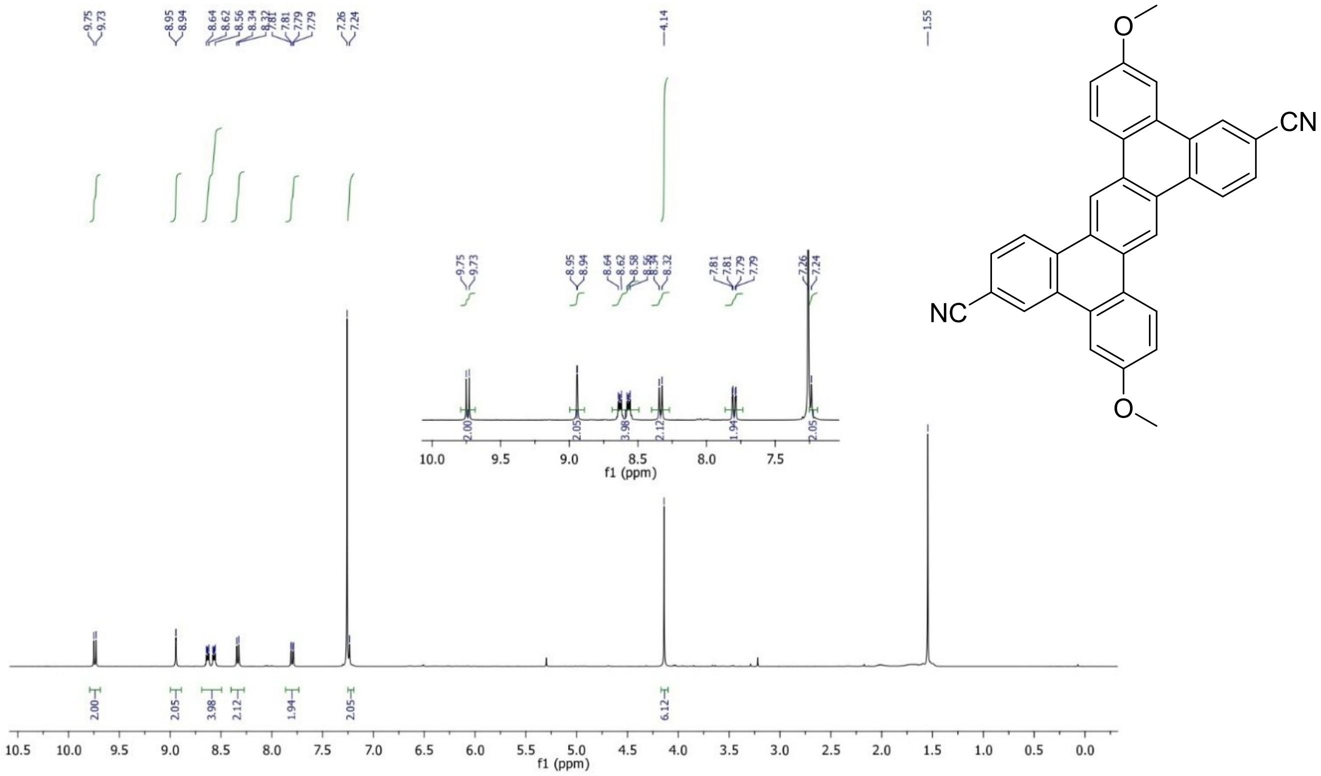

Figure S201. ^{1}H-NMR (600 MHz) spectrum of **11b** in $CDCl_3$.

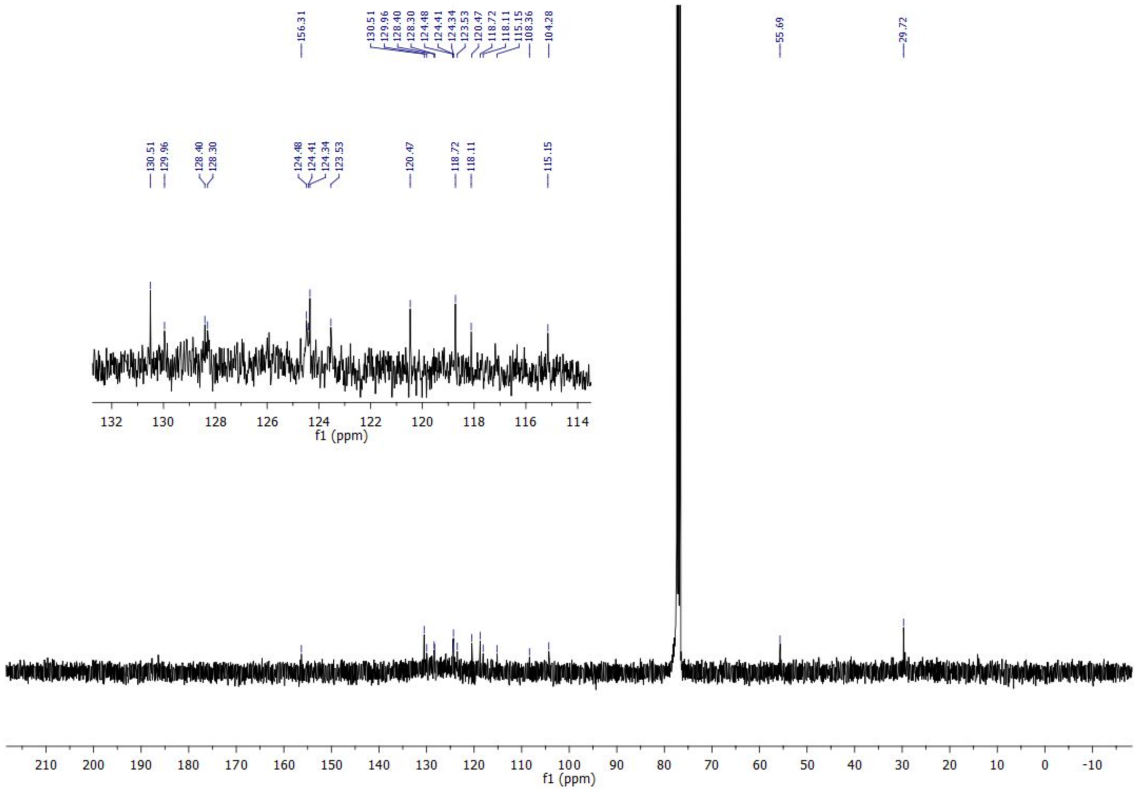

Figure S202. ^{13}C-NMR (151 MHz) spectrum of **11b** in $CDCl_3$.

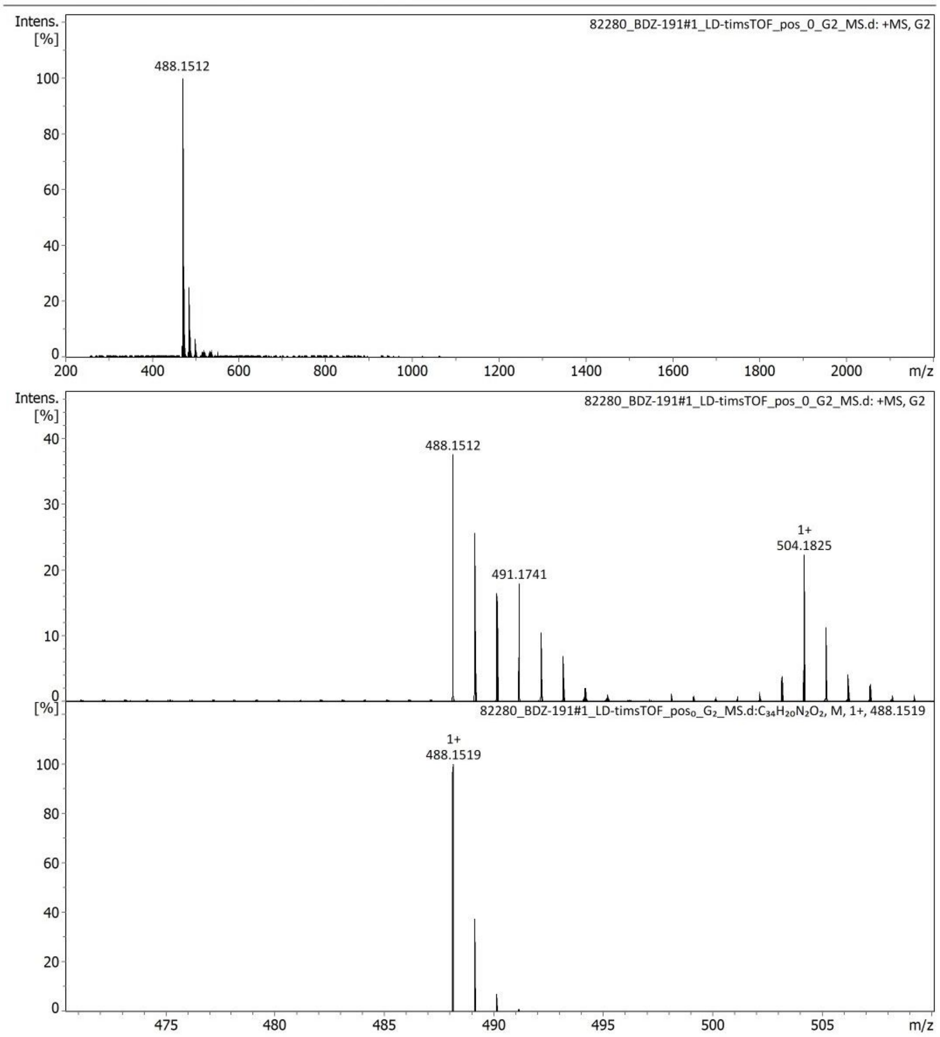

Figure S203. HRMS-LD-TOF mass spectrum of **11b.**

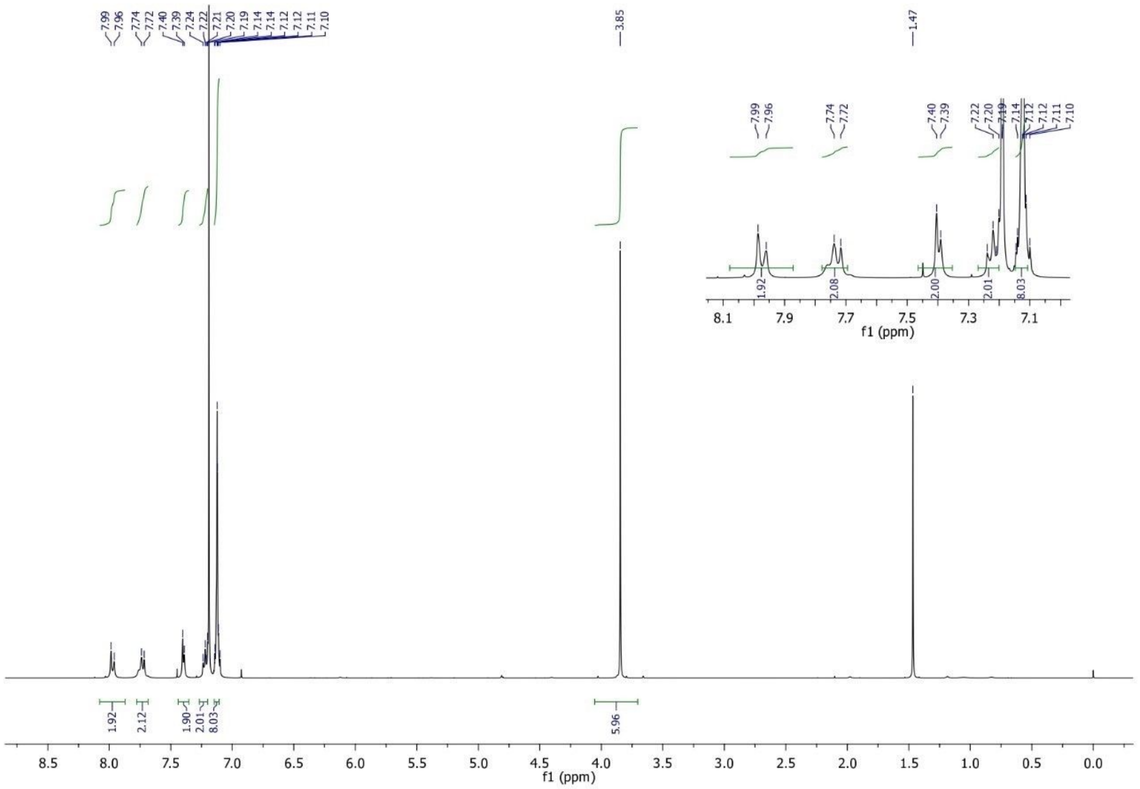

Figure S204. ^{1}H-NMR (600 MHz) spectrum of **10a** in $CDCl_3$.

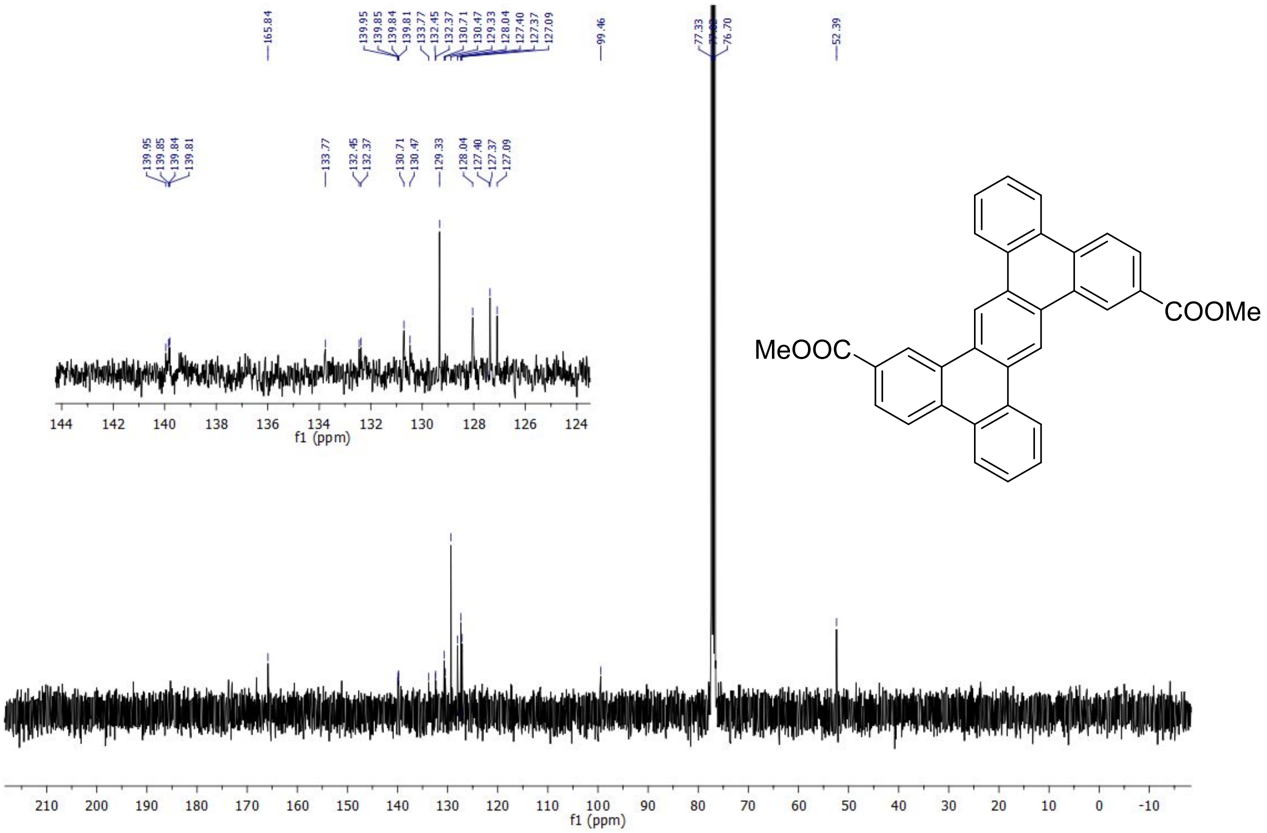

Figure S205. ^{13}C-NMR (151 MHz) spectrum of **10a** in $CDCl_3$.

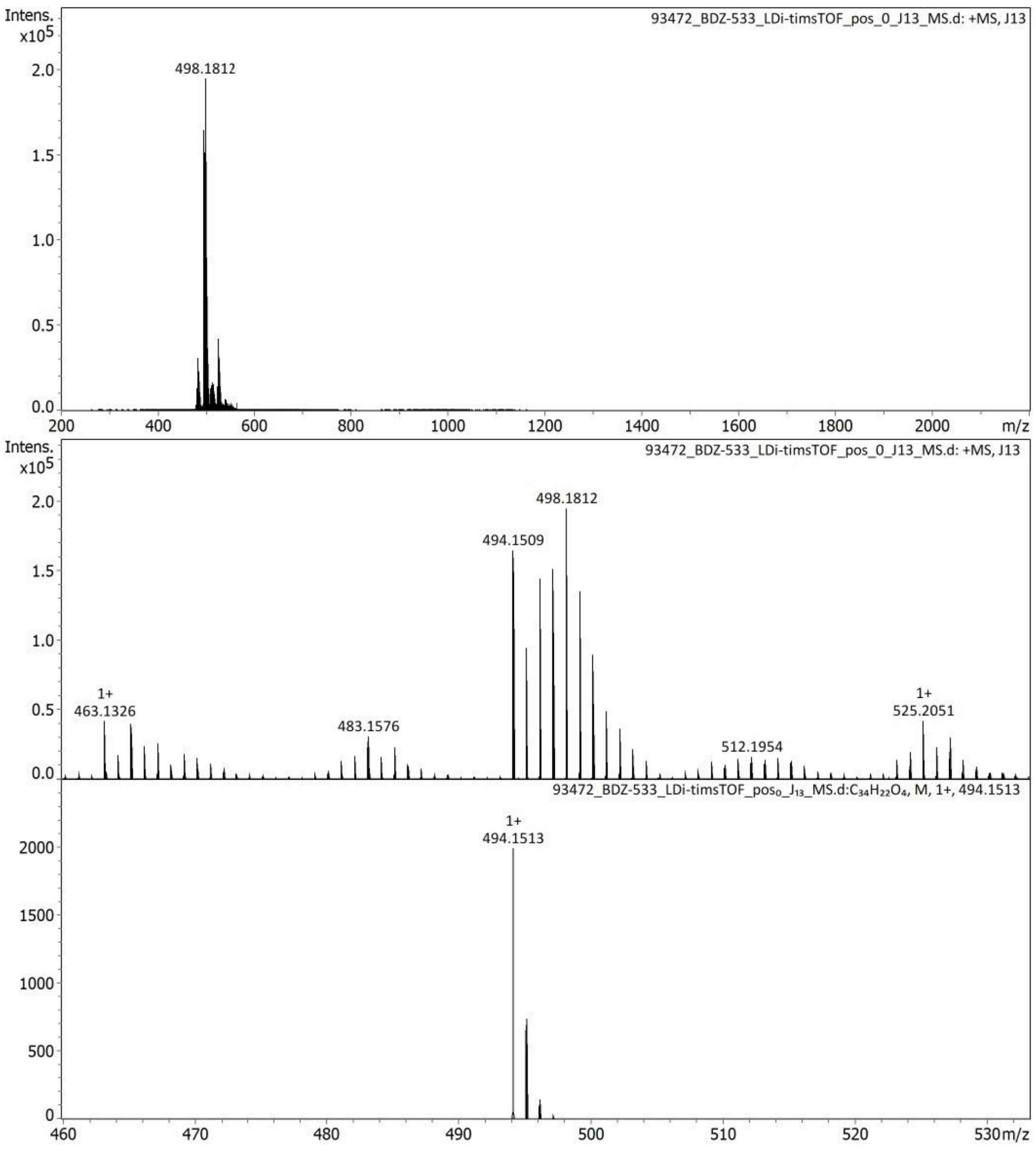

Figure S206. HRMS-LD-TOF mass spectrum of **10a.**

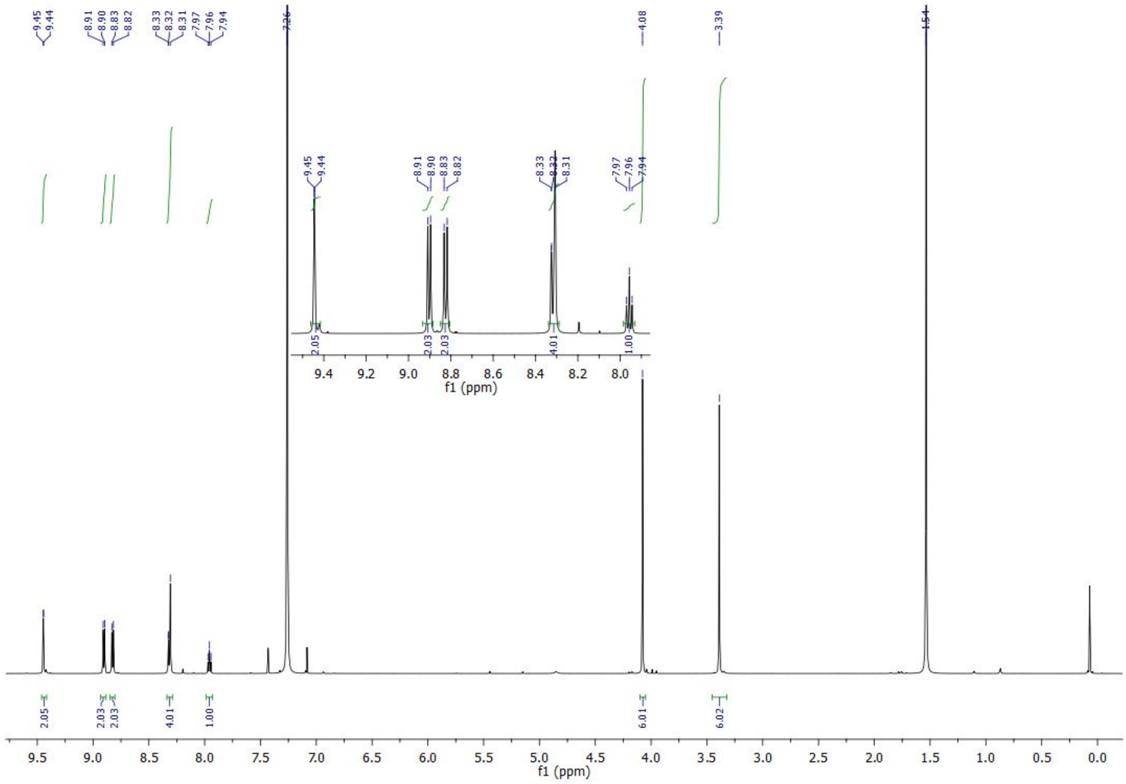

Figure S207. ^{1}H-NMR (600 MHz) spectrum of **14a** in $CDCl_3$.

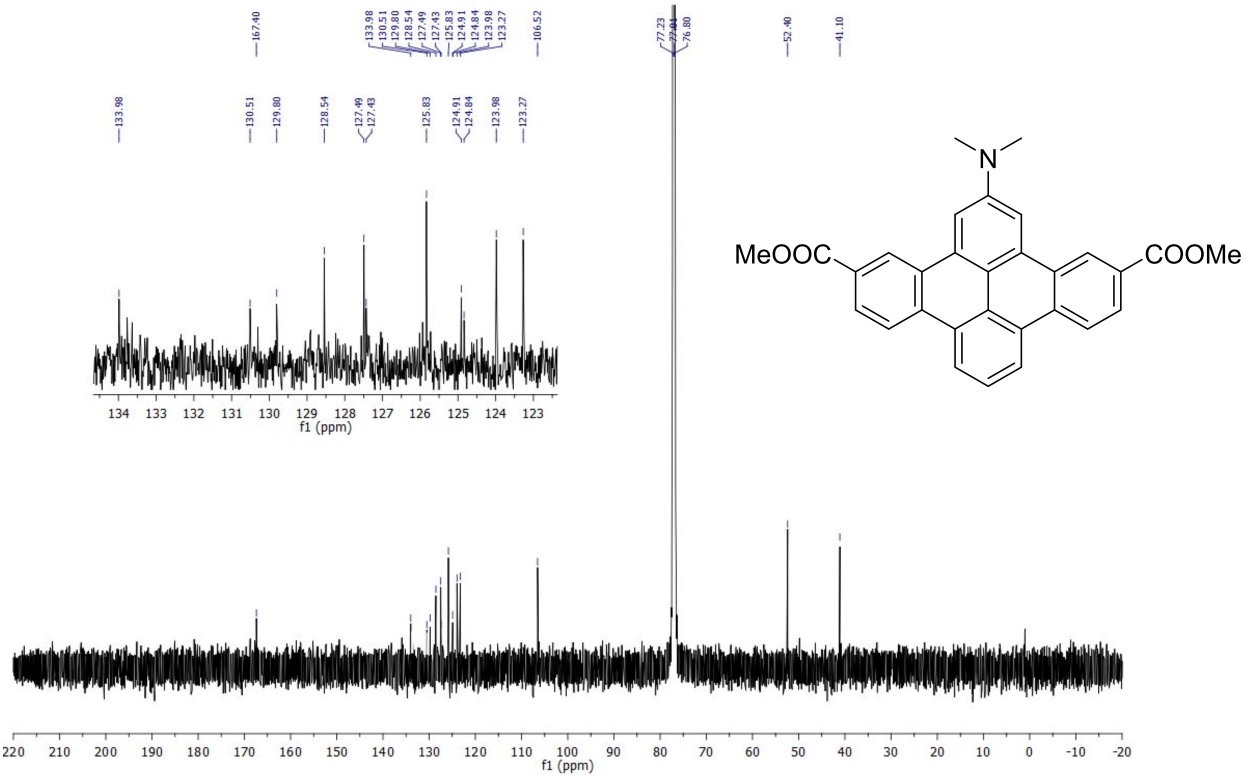

Figure S208. ^{13}C-NMR (151 MHz) spectrum of **14a** in $CDCl_3$.

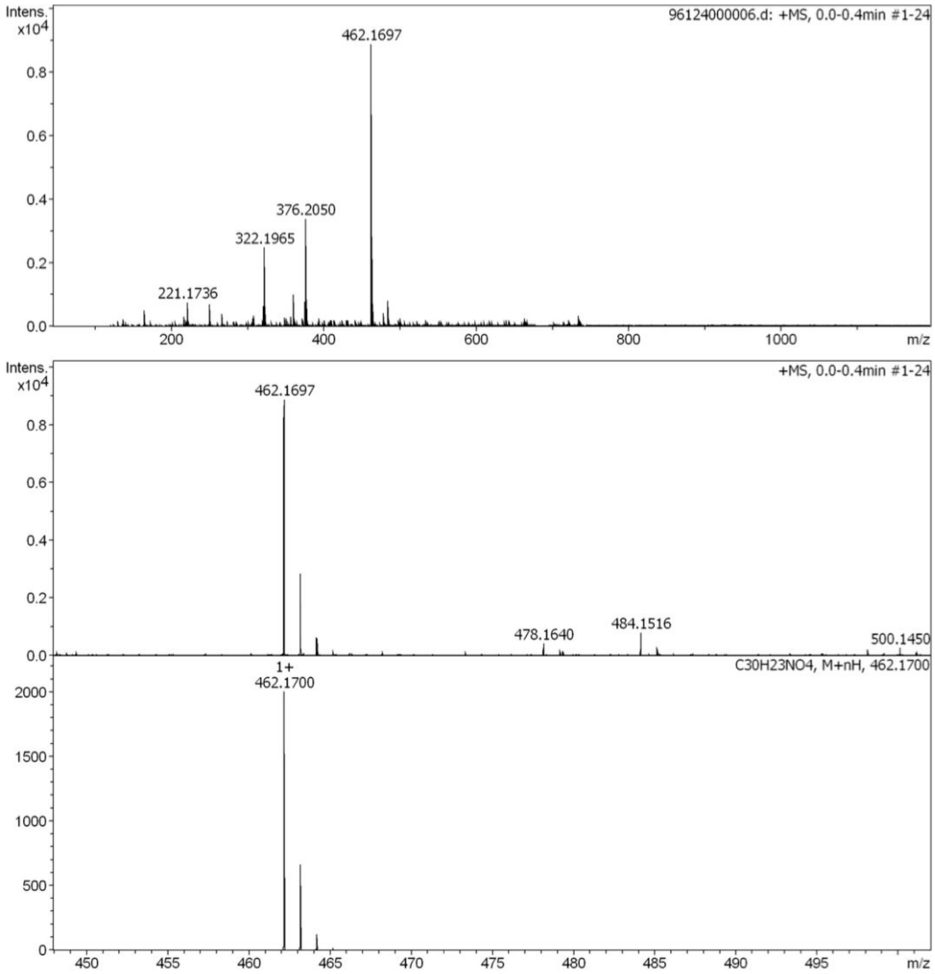

Figure S209. HRMS-LD-TOF mass spectrum of **14a.**

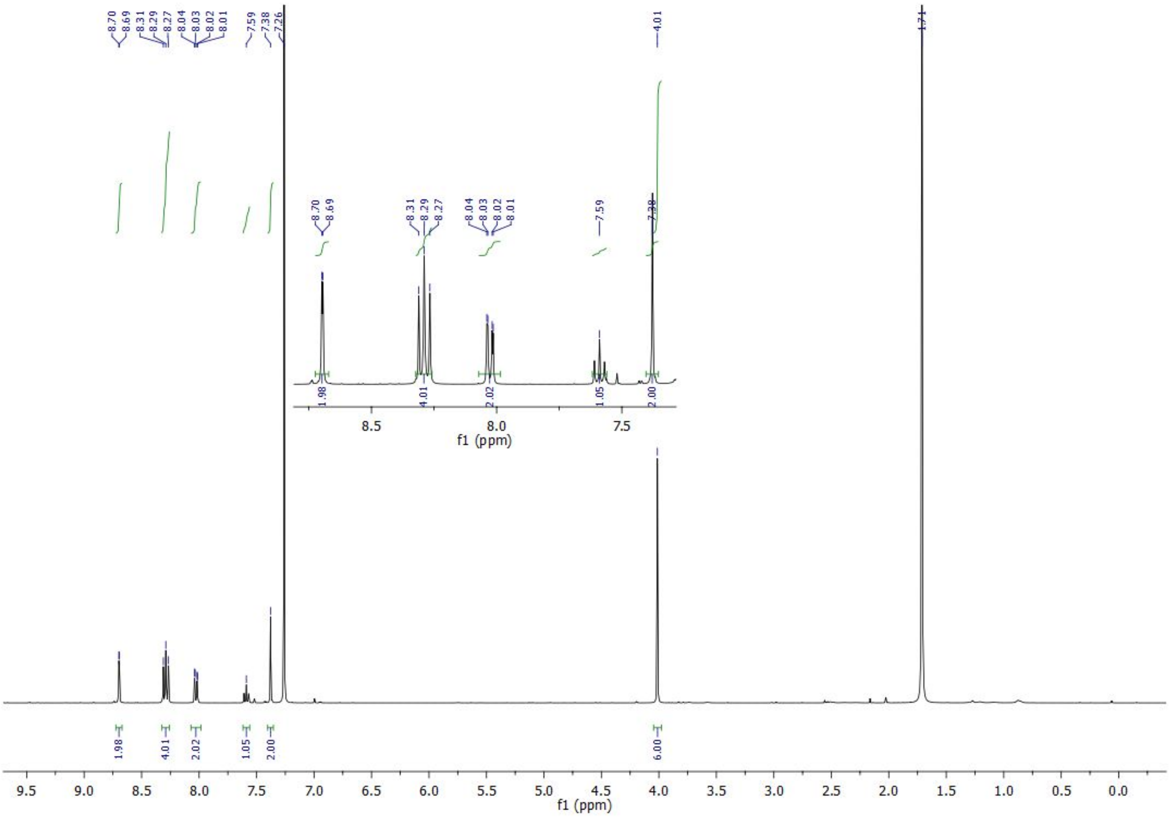

Figure S210. ^{1}H-NMR (600 MHz) spectrum of **14b** in $CDCl_3$.

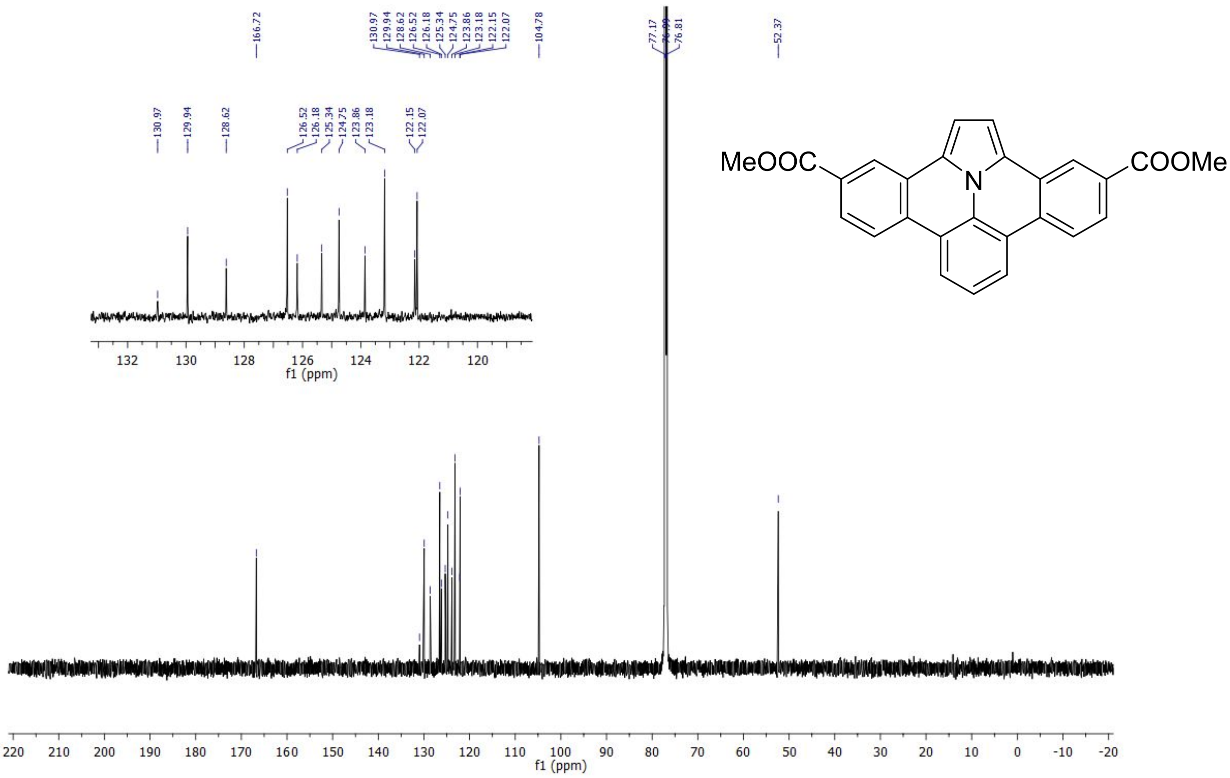

Figure S211. ^{13}C-NMR (151 MHz) spectrum of **14b** in $CDCl_3$.

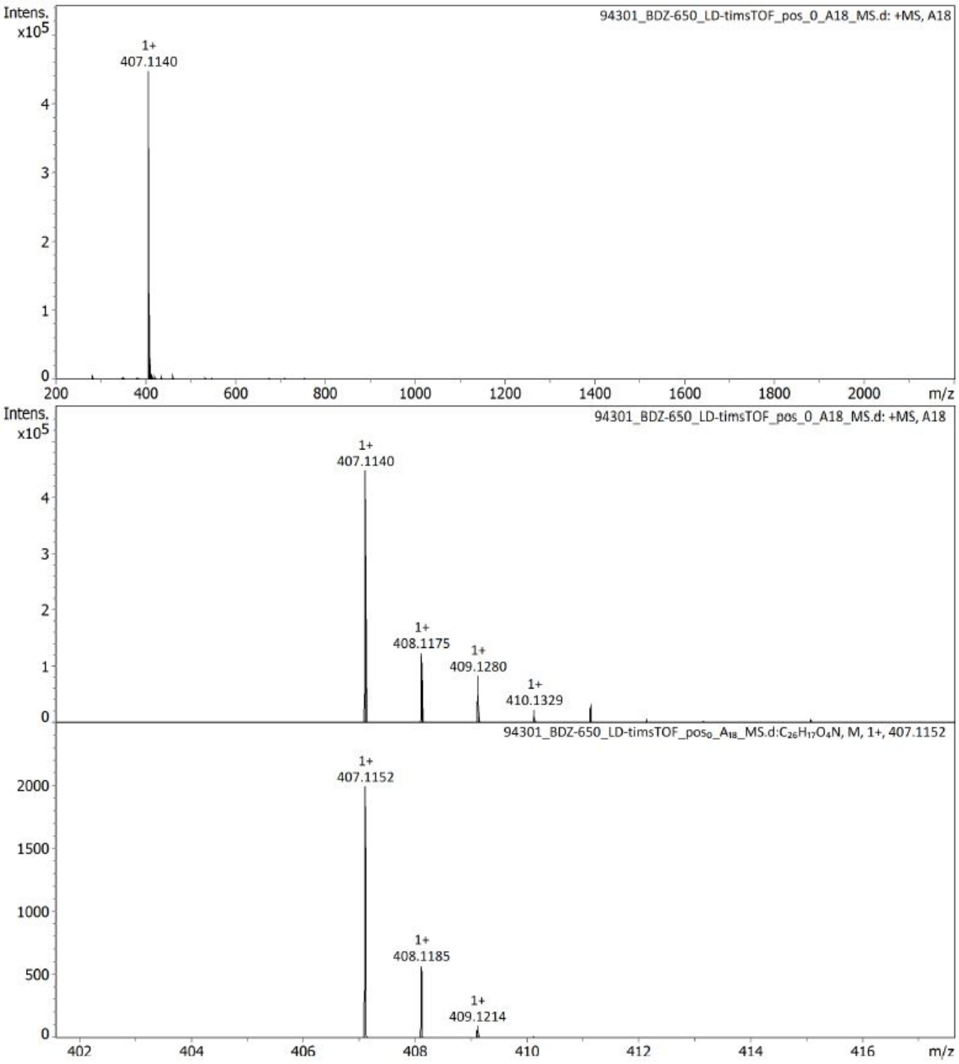

Figure S212. HRMS-LD-TOF mass spectrum of **14b.**

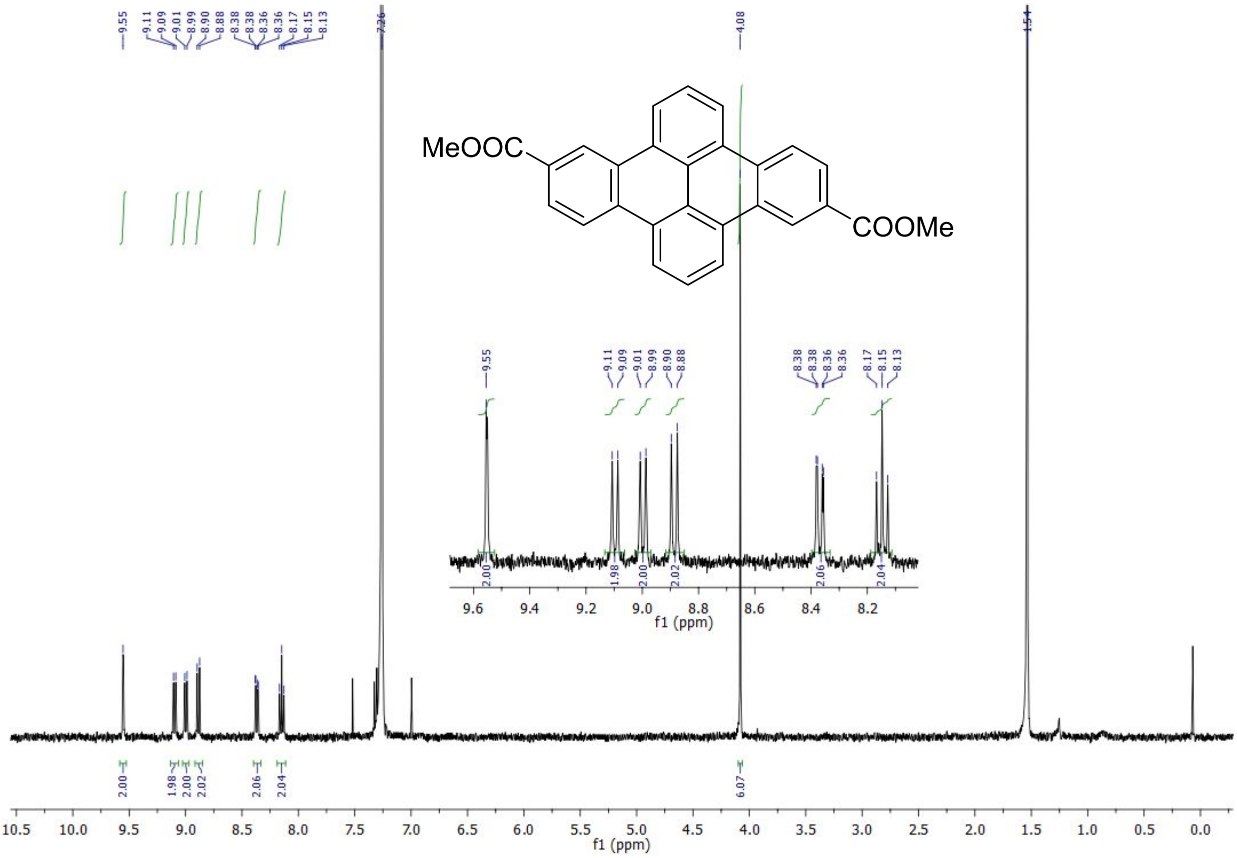

Figure S213. ^{1}H-NMR (700 MHz) spectrum of **14c** in $CDCl_3$.

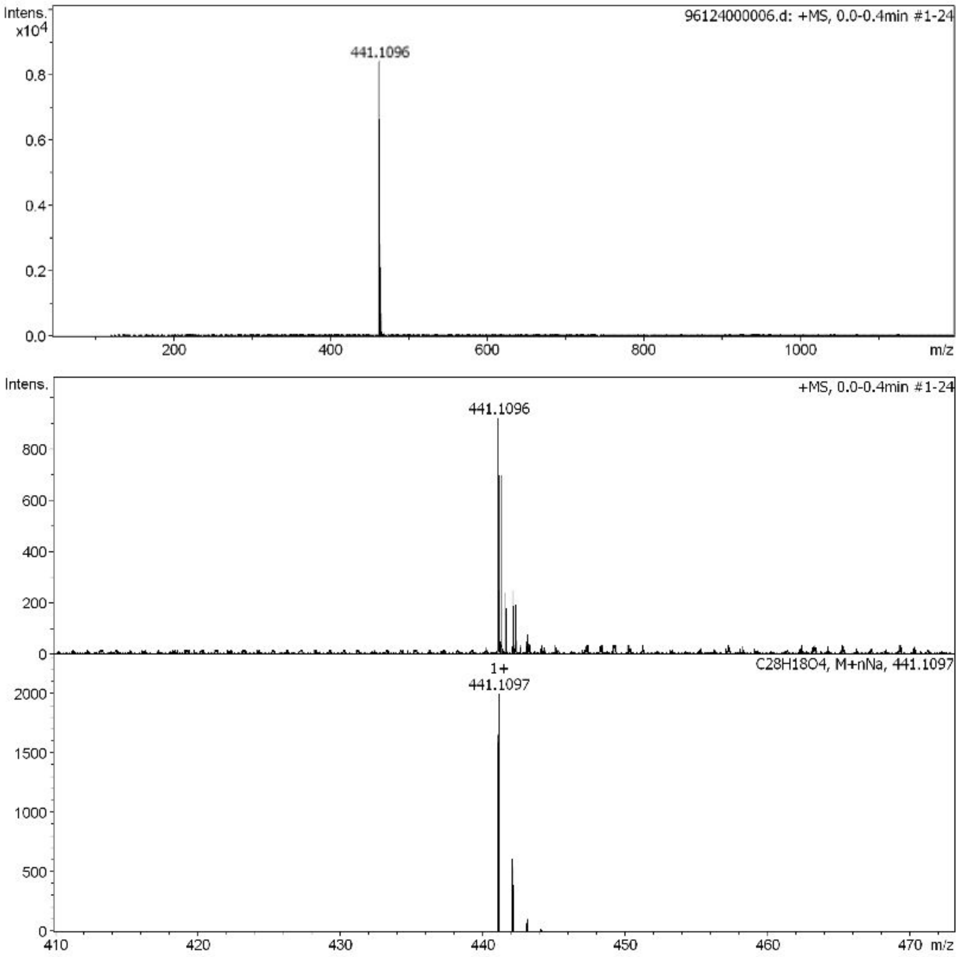

Figure S214. HRMS-LD-TOF mass spectrum of **14c.**

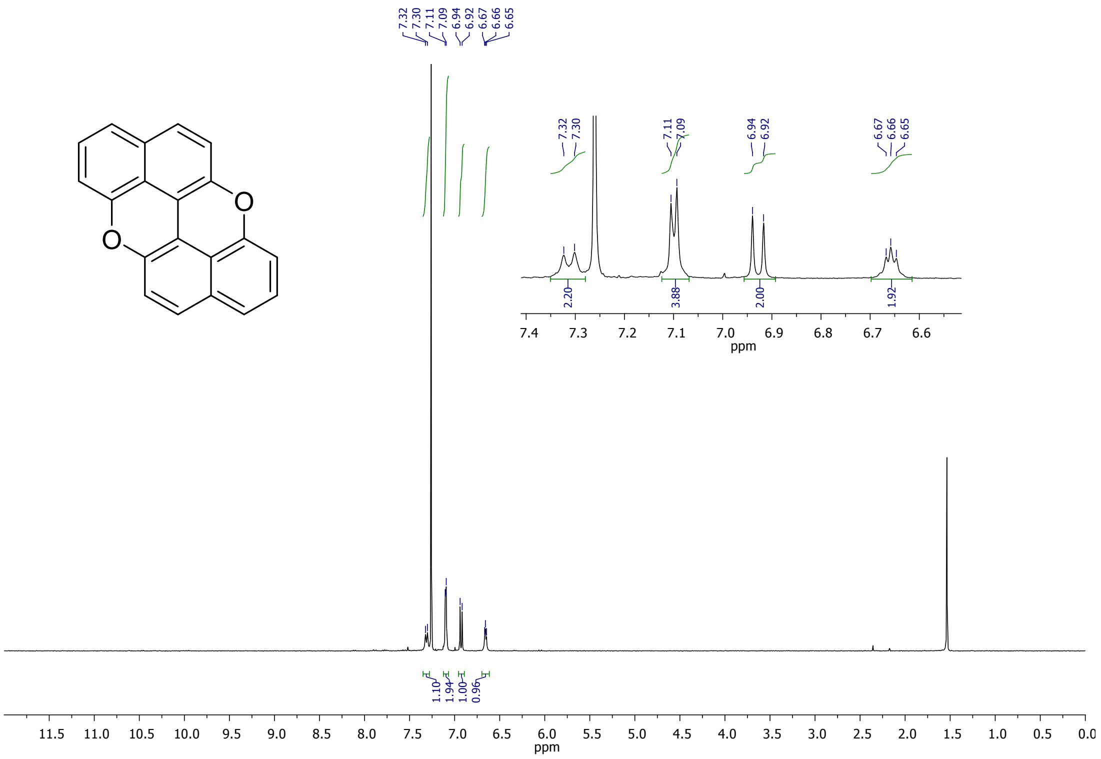

Figure S215. ^{1}H-NMR (400 MHz) spectrum of **PXX** in $CDCl_3$.

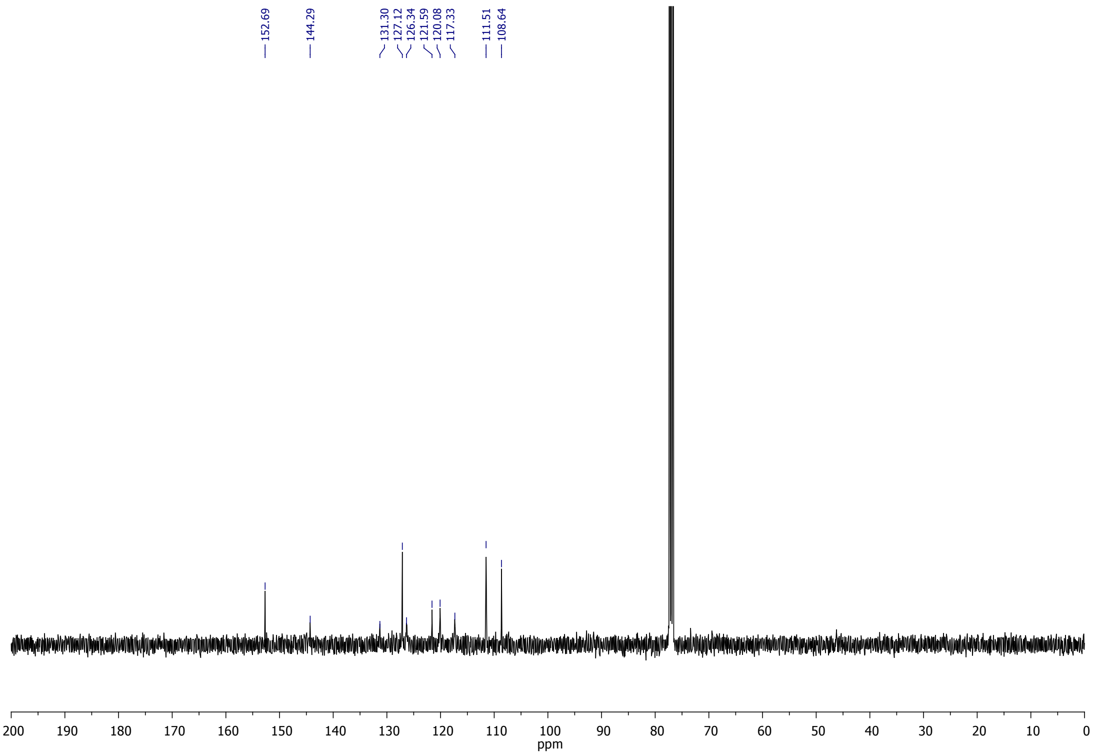

Figure S216. ^{13}C-NMR (101 MHz) spectrum of **PXX** in $CDCl_3$.

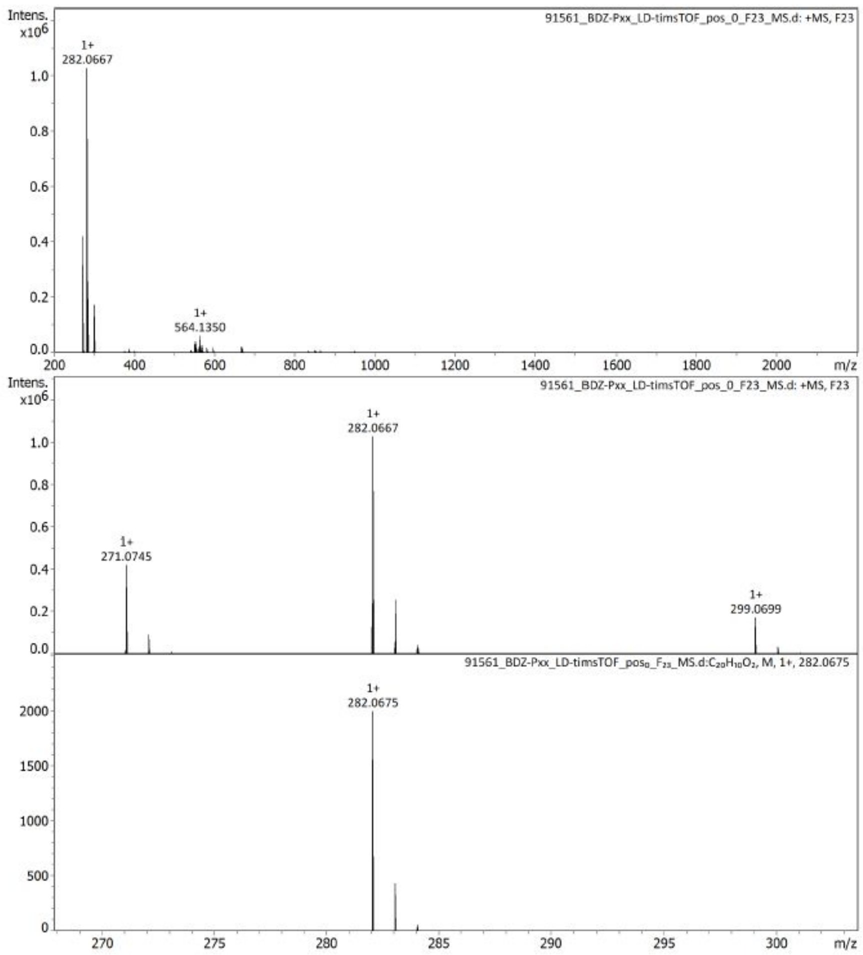

Figure S217. HRMS-LD-TOF mass spectrum of **PXX.**

3. Photophysical characterizations and Stern-Volmer Analysis

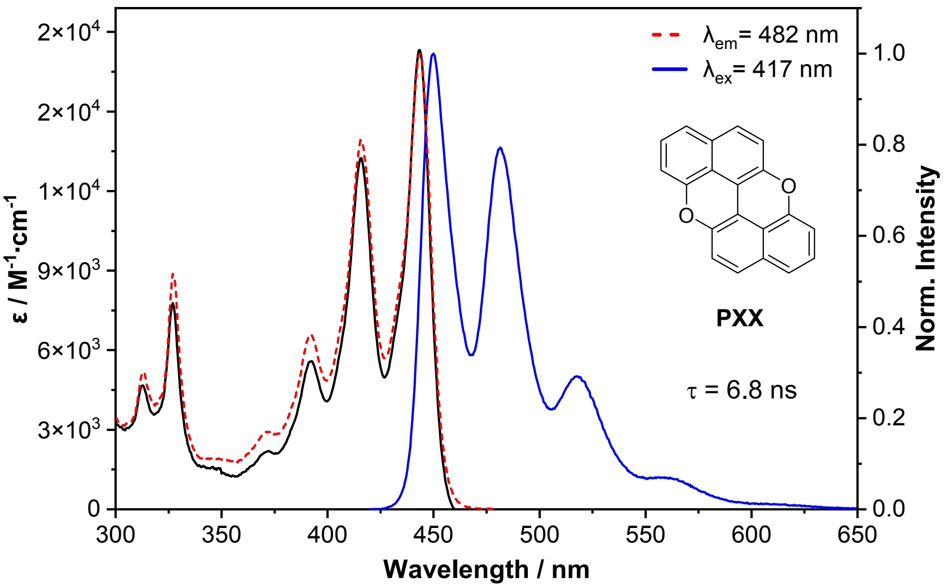

Figure S218. Absorption (black), excitation (dashed, red) and emission (blue) spectra of **PXX** (5.4×10^{-6} M) in DMSO.

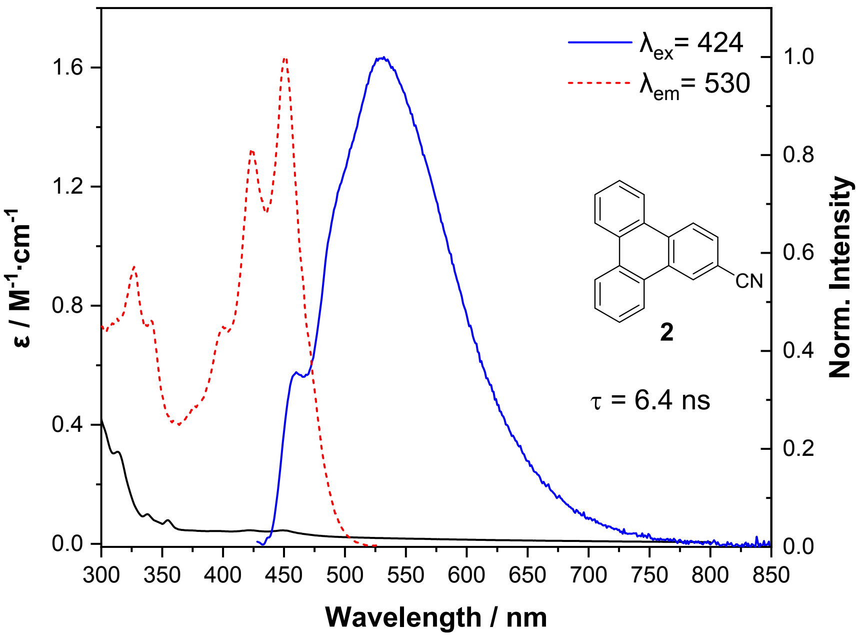

Figure S219. Absorption (black), excitation (dashed, red) and emission (blue) spectra of **2** (3.6×10^{-3} M) in DMSO.

Fluorophore	$\tau_{Fluorophore}$ (ns)	Quencher	$\tau_{Quencher}$ (ns)	Solvent	k_q (M^{-1} s^{-1})
PXX	6.8	1	16.5	DMSO	2.2×10^{10}
PXX	6.8	1a	14.9	DMSO	No quenching

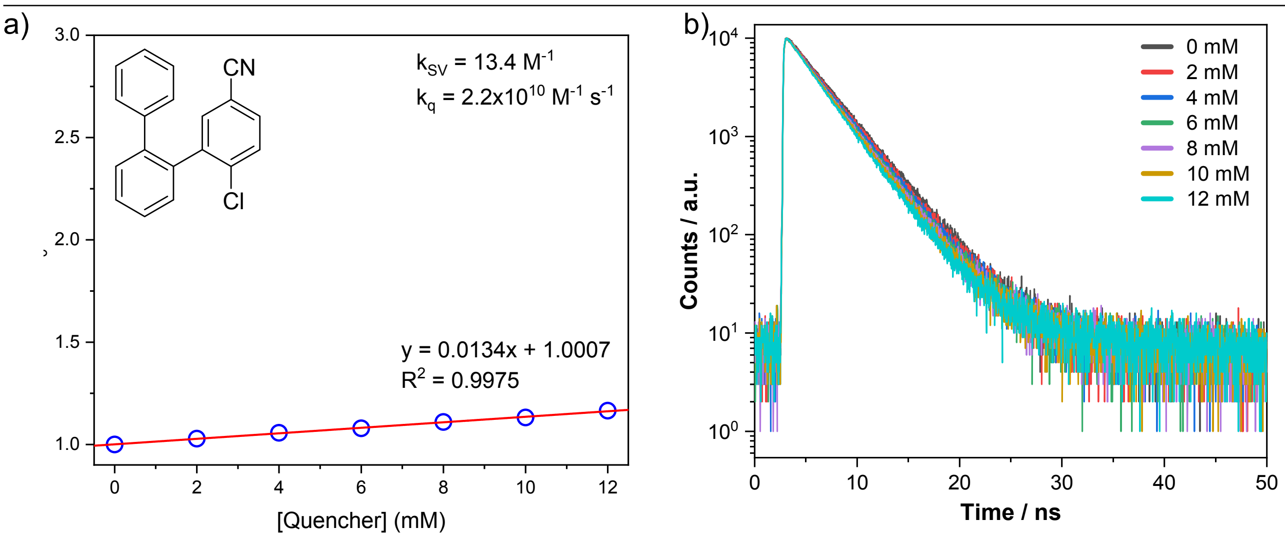

Figure S220 a) Fluorescence decay of **PXX** (0.01 mM) in DMSO, in the presence of increasing concentration of **1** (0 – 12 mM); λ_{ex}= 405.6 nm, λ_{em}= 450 nm. b) Stern-Volmer plot.

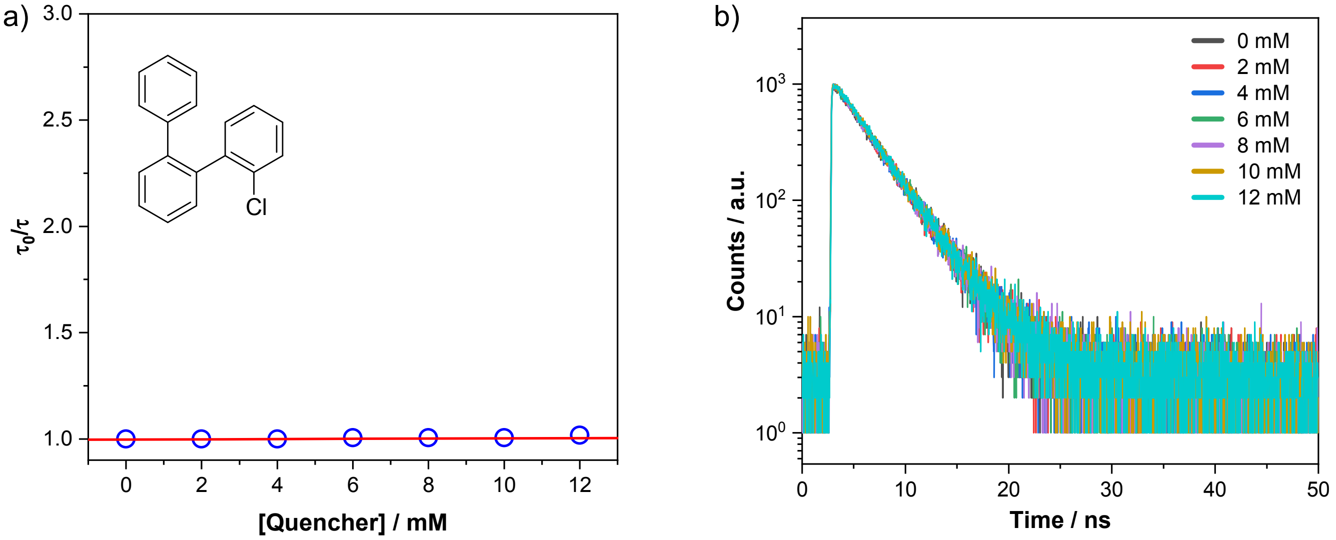

Figure S221 a) Fluorescence decay of PXX (0.01 mM) in DMSO, in the presence of increasing concentration of **1a** (0 – 12 mM); λ_{ex}= 405.6 nm, λ_{em}= 450 nm. b) Stern-Volmer plot.

4. Electrochemical Analysis

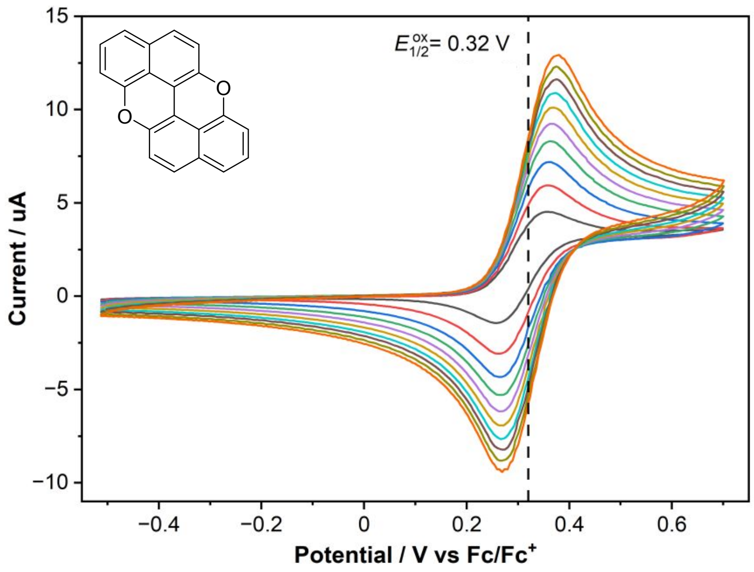

Figure S222. Cyclic voltammetry of **PXX** (0.5 mM) in CH_2Cl_2 at different scan rates.

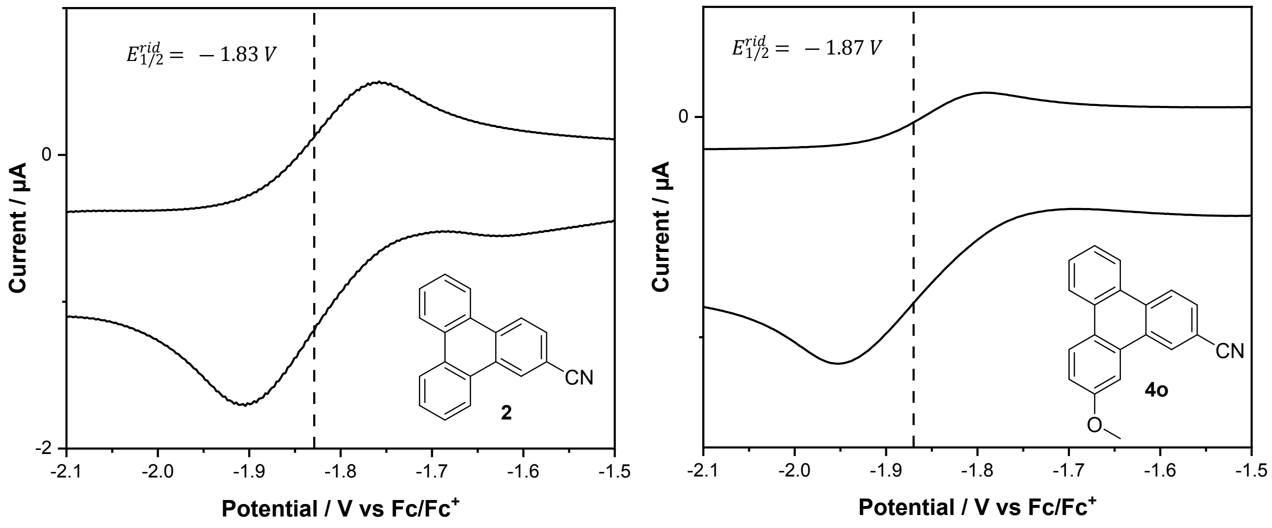

Figure S223. Cyclic voltammetry of **2** (0.5 mM, left) and **4o** (0.5 mM, right) in CH_2Cl_2 at 10 mVs. Zoom of the -2.1/-1.5 V window done for clarification.

5. Crystallographic data

Datablock: bdz-683_short_axis

Bond precision: C-C = 0.0188 A Wavelength=0.71073

Cell:	a=3.8257(11)	b=21.347(3)	c=17.274(4)
	alpha=90	beta=92.91(2)	gamma=90
Temperature:	100 K		

	Calculated	Reported
Volume	1408.9(6)	1408.9(6)
Space group	P 21/n	P 1 21/n 1
Hall group	-P 2yn	-P 2yn
Moiety formula	C21 H13 N O2	C21 H13 N O2
Sum formula	C21 H13 N O2	C21 H13 N O2
Mr	311.32	311.32
Dx,g cm-3	1.468	1.468
Z	4	4
Mu (mm-1)	0.095	0.095
F000	648.0	648.0
F000'	648.29	
h,k,lmax	3,19,15	4,26,44
Nref	1111	15955
Tmin,Tmax	0.982,0.997	0.139,0.745
Tmin'	0.960	

Correction method= # Reported T Limits: Tmin=0.139 Tmax=0.745
AbsCorr = MULTI-SCAN

Data completeness= 14.361 Theta(max)= 18.824

R(reflections)= 0.0724(6930) wR2(reflections)= 0.2111(15955)

S = 0.914 Npar= 225

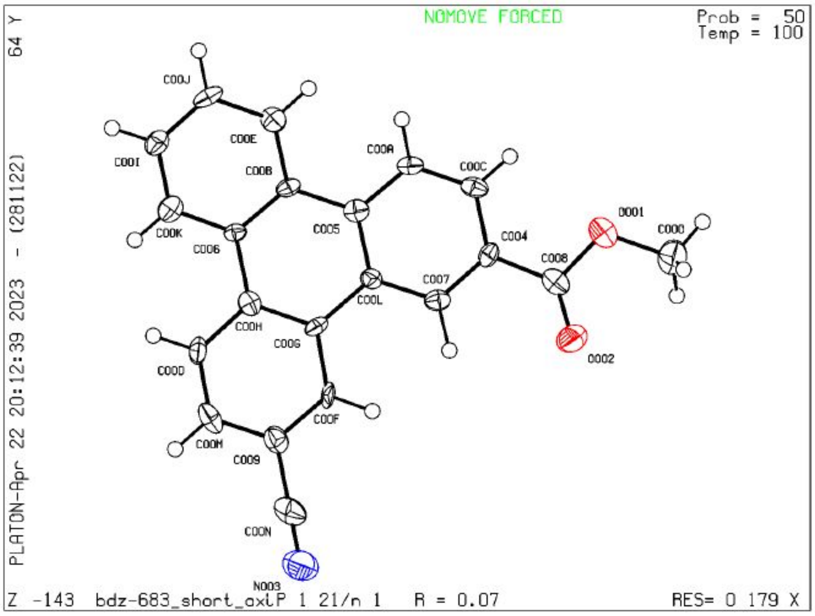

Figure S224. Crystal data and structure refinement for **4j.**

Datablock: bdz532

```
Bond precision:   C-C = 0.0017 A           Wavelength=0.71073

Cell:            a=9.0688(6)        b=9.2098(7)       c=9.4451(6)
                 alpha=92.205(6)    beta=98.297(5)    gamma=110.187(5)
Temperature:     100 K

                  Calculated                   Reported
Volume            729.35(9)                    729.35(9)
Space group       P -1                         P -1
Hall group        -P 1                         -P 1
Moiety formula    C21 H15 N O2                 C21 H15 N O2
Sum formula       C21 H15 N O2                 C21 H15 N O2
Mr                313.34                       313.34
Dx,g cm-3         1.427                        1.427
Z                 2                            2
Mu (mm-1)         0.092                        0.092
F000              328.0                        328.0
F000'             328.14
h,k,lmax          11,11,12                     11,11,11
Nref              3272                         3073
Tmin,Tmax         0.987,0.995                  0.802,0.986
Tmin'             0.986

Correction method= # Reported T Limits: Tmin=0.802 Tmax=0.986
AbsCorr = MULTI-SCAN

Data completeness= 0.939           Theta(max)= 27.267

                                                 wR2(reflections)=
R(reflections)= 0.0390( 2257)                    0.1092( 3073)

S = 0.998                  Npar= 219
```

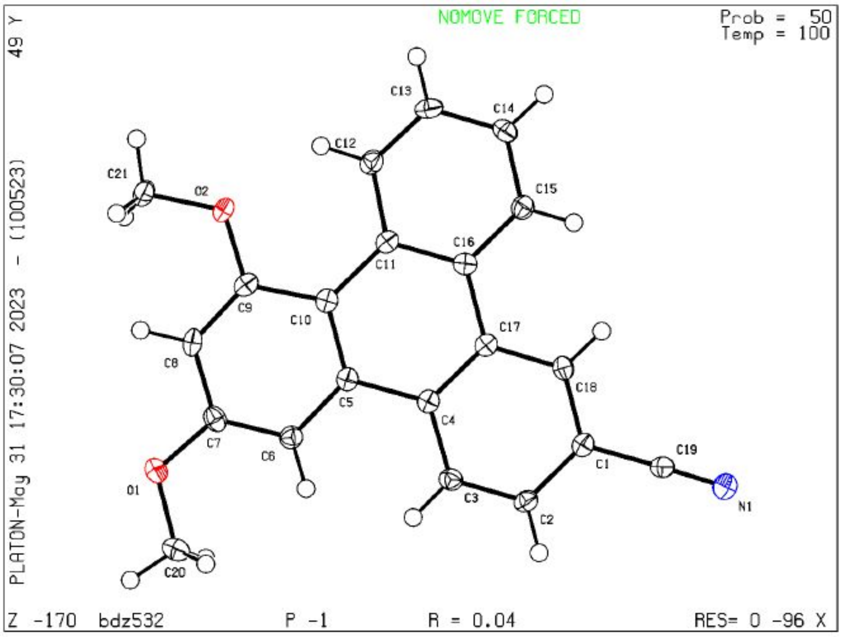

Figure S225. Crystal data and structure refinement for **4n.**

Datablock: PXX241A

Bond precision: C-C = 0.0097 A Wavelength=0.62000

Cell:	a=3.8410(8)	b=17.816(4)	c=35.542(7)
	alpha=90	beta=91.27(3)	gamma=90
Temperature:	100 K		

	Calculated	Reported
Volume	2431.6(9)	2431.6(8)
Space group	P 21/c	P 21/c
Hall group	-P 2ybc	-P 2ybc
Moiety formula	C28 H18 O4, 0.5(C20 H10 O2)	C20 H10 O2, C56 H36 O8
Sum formula	C38 H23 O5	C76 H46 O10
Mr	559.56	1119.13
Dx,g cm-3	1.528	1.529
Z	4	2
Mu (mm-1)	0.076	0.076
F000	1164.0	1164.0
F000'	1164.32	
h,k,lmax	4,20,41	4,20,41
Nref	4033	4009
Tmin,Tmax	0.995,0.998	0.578,0.745
Tmin'	0.992	

Correction method= # Reported T Limits: Tmin=0.578 Tmax=0.745
AbsCorr = MULTI-SCAN

Data completeness= 0.994 Theta(max)= 21.123

R(reflections)= 0.0963(1604) wR2(reflections)= 0.2896(4009)

S = 1.019 Npar= 391

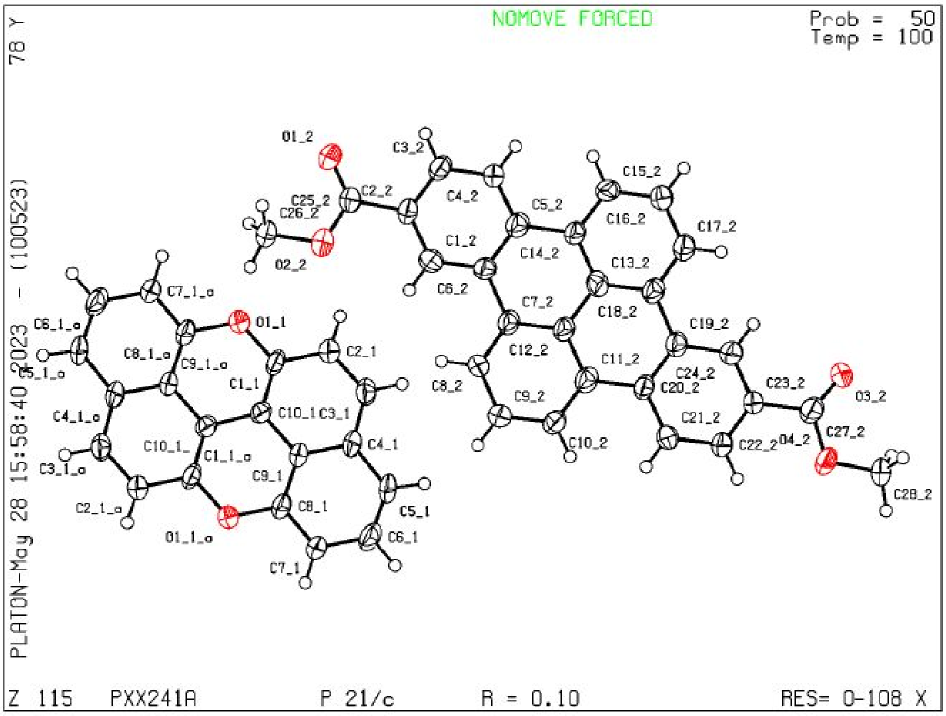

Figure S226. Crystal data and structure refinement for cocrystal **14c/PXX.**

Datablock: PXXa

Bond precision:	C-C = 0.0017 A		Wavelength=0.62000
Cell:	a=15.824(3)	b=4.943(1)	c=16.077(3)
	alpha=90	beta=90.84(3)	gamma=90
Temperature:	100 K		

	Calculated	Reported
Volume	1257.4(4)	1257.4(4)
Space group	C 2/c	C 2/c
Hall group	-C 2yc	-C 2yc
Moiety formula	C20 H10 O2	C20 H10 O2
Sum formula	C20 H10 O2	C20 H10 O2
Mr	282.28	282.28
Dx,g cm-3	1.491	1.491
Z	4	4
Mu (mm-1)	0.072	0.072
F000	584.0	584.0
F000'	584.15	
h,k,lmax	26,8,26	26,7,25
Nref	3044	2726
Tmin,Tmax	0.996,0.999	
Tmin'	0.993	

Correction method= Not given

Data completeness= 0.896 Theta(max)= 31.087

R(reflections)= 0.0557(1632) wR2(reflections)= 0.1767(2726)

S = 1.022 Npar= 100

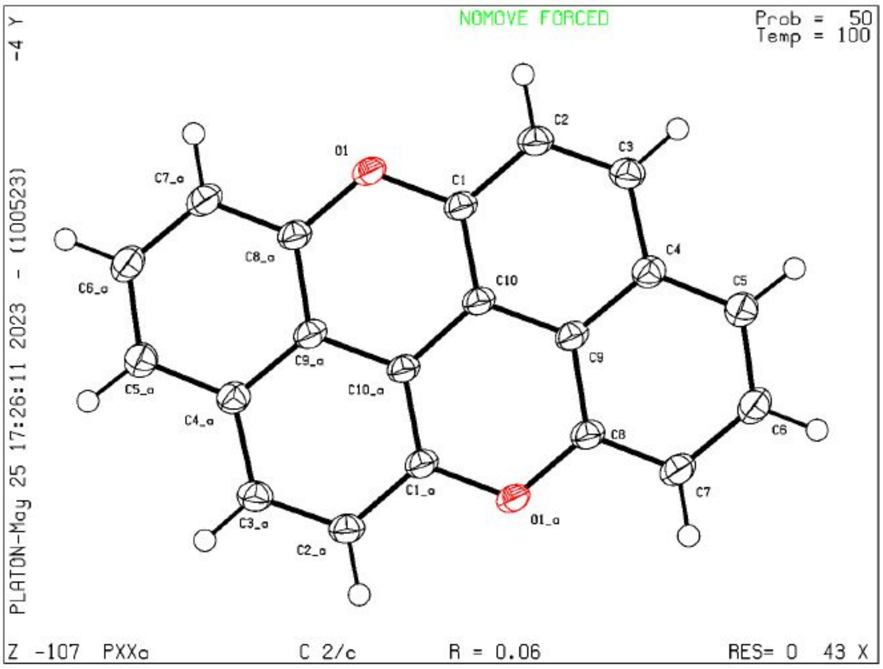

Figure S227. Crystal data and structure refinement for **PXX.**

Datablock: PXXb

Bond precision: C-C = 0.0020 A Wavelength=0.62000

Cell:	a=3.763(1)	b=7.552(2)	c=21.221(4)
	alpha=90	beta=92.29(3)	gamma=90
Temperature:	100 K		

	Calculated	Reported
Volume	602.6(3)	602.6(2)
Space group	P 21/c	P 21/c
Hall group	-P 2ybc	-P 2ybc
Moiety formula	C20 H10 O2	C20 H10 O2
Sum formula	C20 H10 O2	C20 H10 O2
Mr	282.28	282.28
Dx,g cm-3	1.556	1.556
Z	2	2
Mu (mm-1)	0.075	0.075
F000	292.0	292.0
F000'	292.08	
h,k,lmax	6,12,35	6,12,32
Nref	2915	2617
Tmin,Tmax	0.996,0.999	
Tmin'	0.993	

Correction method= Not given

Data completeness= 0.898 Theta(max)= 31.069

R(reflections)= 0.0641(1514) wR2(reflections)= 0.2175(2617)

S = 1.043 Npar= 101

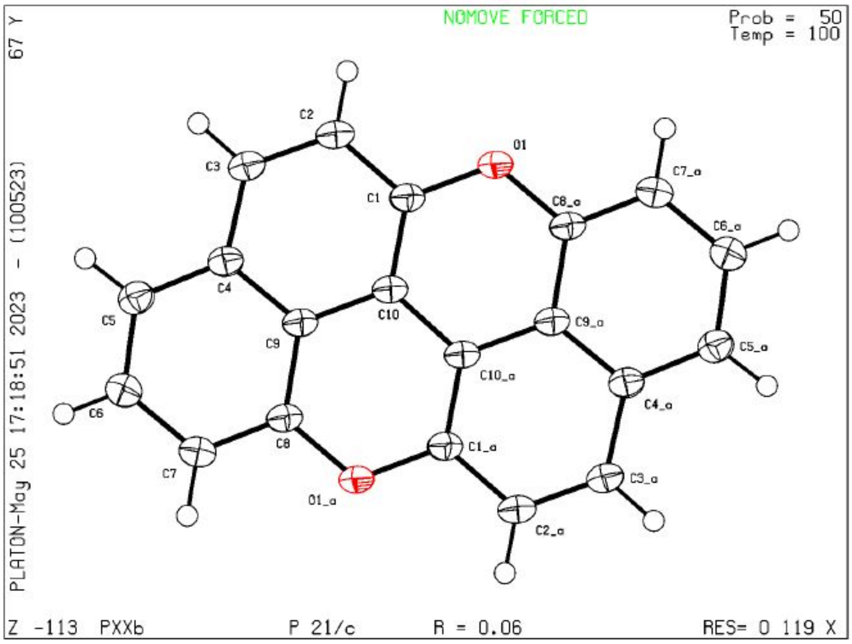

Figure S228. Crystal data and structure refinement for **PXX.**

6. Supplementary data

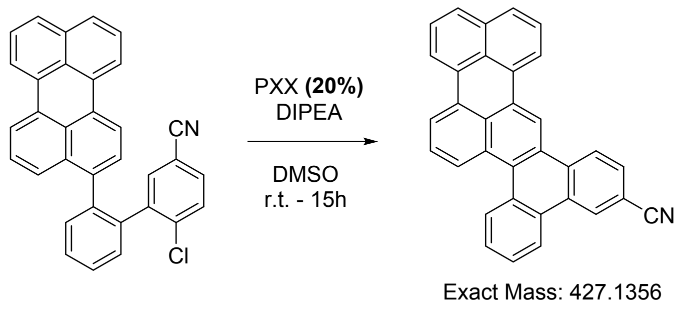

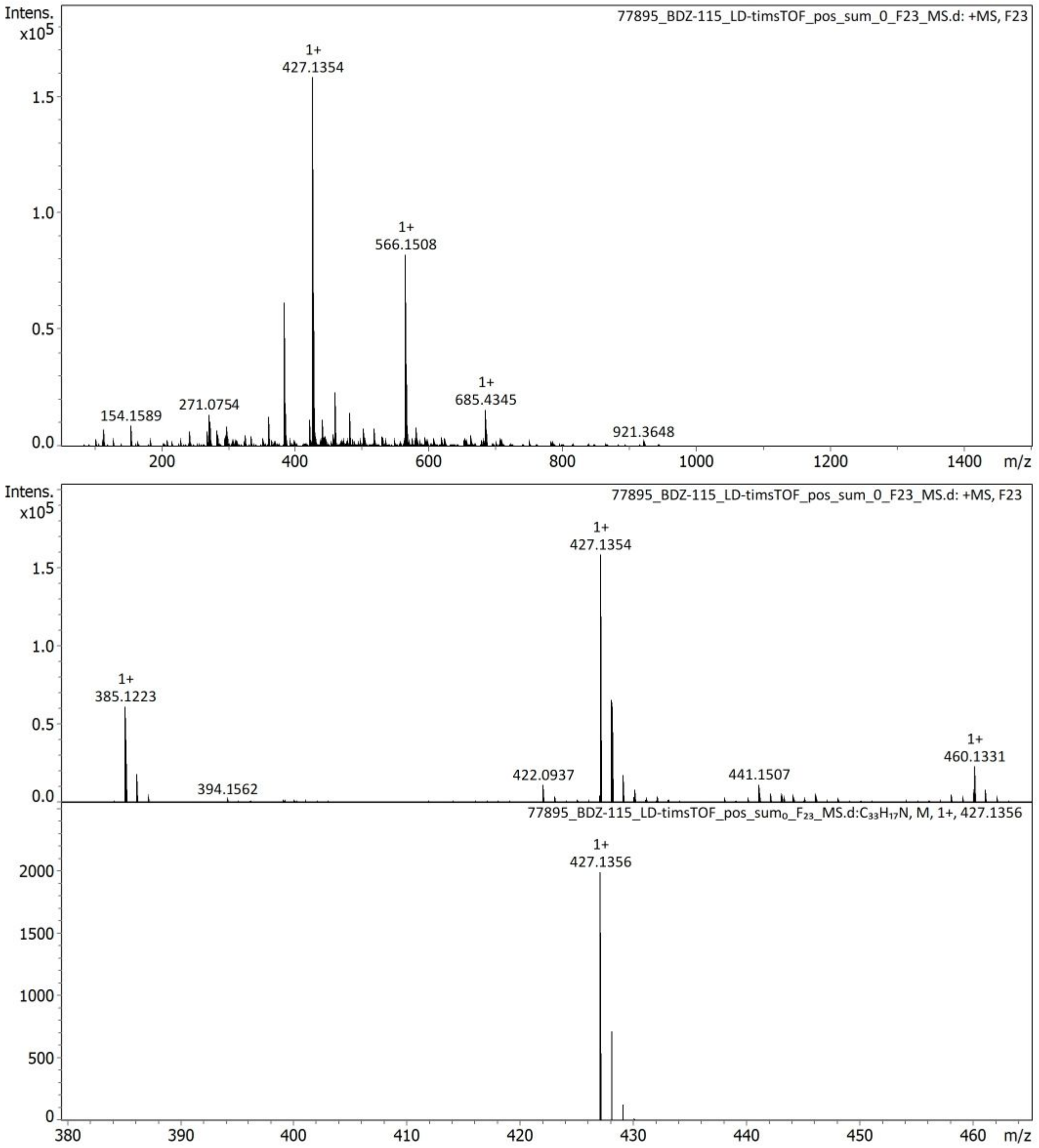

Figure S229. HRMS-ESI mass spectrum of **8d** (crude, 20% cat. charge).

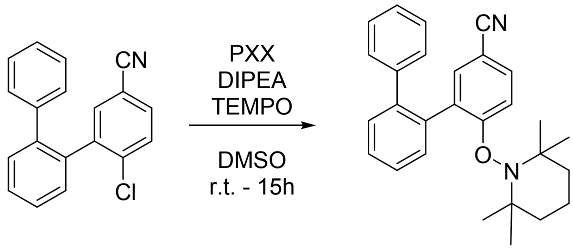

Exact Mass: 411.2431

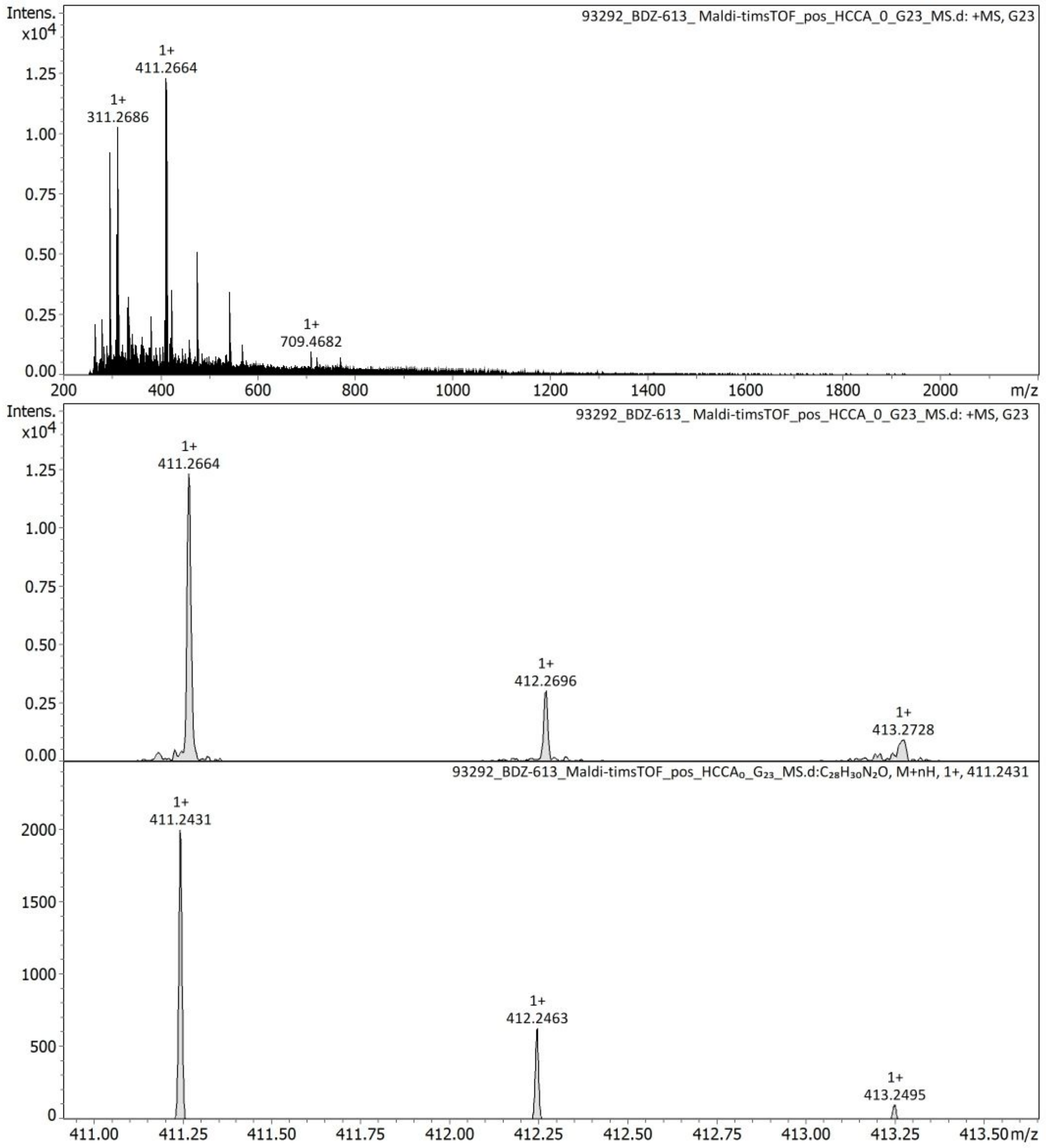

Figure S230. HRMS-MALDI-TOF mass spectrum of trapped adduct **2HTEMPO**.

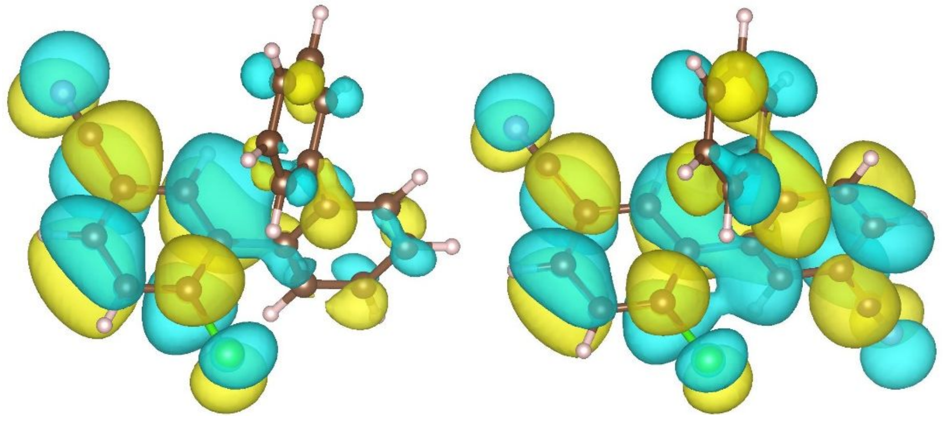

1 (-1.862 eV) **3h** (-2.234 eV)

Figure S231. LUMOs of product **1** (left) and **3h** (right) with respective energies *vs.* vacuum.

Preliminary DFT calculations to model the LUMO orbital of the given precursor has been performed. As show, the LUMO of both reference **1** and targeted **3h** precursors have been calculated. The geometries were optimized with MMFF94 force field and the orbitals calculated with DFT method (level of theory: B3LYP – Base set: 6-311G**). While molecule **1** depicts the LUMO mainly localized on the 1-chloro-4-cyano aryl moiety, oligoarylene precursor **3h** bearing the CN moiety on the linker unit, features a LUMO localized across the linker and the radical acceptor moieties. This likely suggests that the radical anion is highly delocalized and more stable, which might prevent the dissociative electron transfer from occurring, i.e. the formation of the Cl^- anion and aryl radical moiety. As one can see from the minimized model, we can exclude that the π-π stacking interaction plays a role in the stabilization of the anionic species.

7. References

[1] Kabsch, W. XDS. *Acta Crystallog.* Section D, **2010**, *66*, 125–132.

[2] Sheldrick, G. M. SADABS Version 2014/5, University of Göttingen, Germany, **2014**.

[3] Sheldrick, G. M. SHELXT – Integrated space-group and crystal-structure determination. *Acta Crystallog*. Section A, **2015**, *71*, 3-8.

[4] Sheldrick, G. M. SHELXT – Integrated space-group and crystal-structure determination. *Acta Crystallog*. Section C, **2015**, *71*, 3-8.

[5] Emsley, P.; Lohkamp, B. Features and development of Coot. *Acta Crystallog.* Section D, **2010**, *66*, 486-501.

[6] Farrugia, L. WinGX and ORTEP for Windows: an update. *J. Appl. Crystallogr.* **2012**, *45*, 849-854.

[7] Macrae, C. F.; Sovago, I. Mercury 4.0: from visualization to analysis, design and prediction. *J. Appl. Crystallogr.* **2020**, *53*, 226-235.

[8] Le Saux, E.; Zanini, M.; Melchiorre, P. Photochemical Organocatalytic Benzylation of Allylic C–H Bonds. *J. Am. Chem. Soc.* **2022**, *144*, 1113–1118.

[9] Wang, T.-F.; Lin, C.-L.; Chen, C.-N.; Wang, T.-C. Easily Accessible 2-(2-Bromophenyl)-4,4,5,5-Tetramethyl-[1,3,2]Dioxaborolane for Suzuki-Miyaura Reactions. *J. Chinese Chem. Soc*. **2007**, *54*, 811–816.

[10] Shimizu, M.; Nagao, I.; Tomioka, Y.; Kadowaki, T.; Hiyama, T. Palladium-catalyzed double cross-coupling reaction of 1,2-bis(pinacolatoboryl)alkenes and -arenes with 2,2′ -dibromobiaryls: annulative approach to functionalized polycyclic aromatic hydrocarbons. *Tetrahedron* **2011**, *67*, 8014-8026.

[11] Qiao, H.; Deng, Y.; Peng, R.; Wang, G.; Yuan, J.; Tan, S. Effect of π-spacers and anchoring groups on the photovoltaic performances of ullazine-based dyes. *RSC Adv.* **2016**, *6*, 70046–70055.

[12] Pratap, R.; Parrish, D.; Gunda, P.; Venkataraman, D.; Lakshman, M. K. Influence of Biaryl Phosphine Structure on C−N and C−C Bond Formation. *J. Am. Chem. Soc.* **2009**, *131*, 12240–12249.

[13] Daigle, M.; Picard-Lafond, A.; Soligo, E.; Morin, J. F. Regioselective Synthesis of Nanographenes by Photochemical Cyclodehydrochlorination. *Angew. Chem. Int. Ed.* **2016**, *55*, 2042–2047.

[14] Wipf, P.; Jung, J. Formal Total Synthesis of (+)-Diepoxin σ. *J. Org. Chem.* **2000**, *65,* 6319–6337.

[15] Wehrmann, C. M.; Charlton, R. T.; Chen. M. S. A Concise Synthetic Strategy for Accessing Ambient Stable Bisphenalenyls toward Achieving Electroactive Open-Shell π-Conjugated Materials *J. Am. Chem. Soc*. **2019**, *141*, 3240–3248.

[16] Tsukamoto, T.; Dong, G. Catalytic Dehydrogenative Cyclization of o-Teraryls under pH-Neutral and Oxidant-Free Conditions. *Angew. Chem. Int. Ed.* **2020**, *59*, 15249–15253.

[17] Iwasaki, M.; Iino, S.; Nishihara, Y. Palladium-Catalyzed Annulation of o-Iodobiphenyls with o-Bromobenzyl Alcohols: Synthesis of Functionalized Triphenylenes via C–C and C–H Bond Cleavages. *Org. Lett.* **2013**, *15*, 5326–5329.

[18] Kamei, T.; Uryu, M.; Shimada, T. Cu-Catalyzed Aerobic Oxidative C-H/C-O Cyclization of 2,2′ -Binaphthols: Practical Synthesis of PXX Derivatives. *Org. Lett.* **2017**, *19*, 2714–2717.
